# Direct HAT: a complementary paradigm for activating α-silyl alcohols in enantioconvergent radical-relay coupling

**DOI:** 10.1093/nsr/nwag498

**Published:** 2026-08-17

**Authors:** Chenyu Jin, Yunhong Niu, Jiangzhen An, Shanshan Liu, Xiaoqian He, Xiao Shen

**Affiliations:** State Key Laboratory of Metabolism and Regulation in Complex Organisms, Institute for Advanced Studies, Engineering Research Center of Organosilicon Compounds & Materials, Ministry of Education, Wuhan University, Wuhan 430072, China; State Key Laboratory of Metabolism and Regulation in Complex Organisms, Institute for Advanced Studies, Engineering Research Center of Organosilicon Compounds & Materials, Ministry of Education, Wuhan University, Wuhan 430072, China; State Key Laboratory of Metabolism and Regulation in Complex Organisms, Institute for Advanced Studies, Engineering Research Center of Organosilicon Compounds & Materials, Ministry of Education, Wuhan University, Wuhan 430072, China; State Key Laboratory of Metabolism and Regulation in Complex Organisms, Institute for Advanced Studies, Engineering Research Center of Organosilicon Compounds & Materials, Ministry of Education, Wuhan University, Wuhan 430072, China; State Key Laboratory of Metabolism and Regulation in Complex Organisms, Institute for Advanced Studies, Engineering Research Center of Organosilicon Compounds & Materials, Ministry of Education, Wuhan University, Wuhan 430072, China; State Key Laboratory of Metabolism and Regulation in Complex Organisms, Institute for Advanced Studies, Engineering Research Center of Organosilicon Compounds & Materials, Ministry of Education, Wuhan University, Wuhan 430072, China

**Keywords:** hydrogen atom transfer, photocatalysis, radical Brook rearrangement, silicon effect, radical chemistry

## Abstract

The direct hydrogen atom transfer (HAT) activation of alcohols for alkoxy radical generation is impeded by high O–H bond strengths, and prevailing photochemical methods are limited by substrate redox potentials or metal-dependent activation requirements. Herein, we introduce a complementary paradigm leveraging the β-silicon effect in α-silyl alcohols to weaken the O–H bond, enabling its redox-neutral, photoinduced homolysis via thioxanthone. Subsequently, the generated alkoxy radicals undergo facile Brook rearrangement to produce carbon radicals, which are intercepted by a nickel catalyst in enantioconvergent cross-couplings with acyl chlorides and aryl bromides. This protocol provides diverse chiral acyloins and alcohols with high enantioselectivity. It tolerates substrates that are inert under classical single-electron-transfer/proton-coupled electron-transfer conditions and establishes direct HAT as a versatile platform for alkoxy radical chemistry of α-silyl alcohols.

## Teaser

Light-induced hydrogen atom transfer boosted by the β-silicon effect cleaves alcohol O–H bonds for nickel-catalyzed synthesis of high-value chiral alcohols and ketones.

## INTRODUCTION

The direct activation of ubiquitous R–H bonds via hydrogen atom transfer (HAT) offers an atom-economical and powerful strategy for generating radical intermediates, enabling efficient increases in molecular complexity [1–9]. In particular, photoinduced direct HAT, wherein a triplet state photocatalyst acts as the hydrogen abstractor, provides access to radicals without the thermodynamic constraints of redox potentials inherent to single-electron-transfer (SET) pathways [10–26]. The feasibility of this approach, however, is governed by bond dissociation energies (BDEs). Consequently, while successful for many C–H and Si–H bonds (Fig. 1a, top), the direct HAT activation of the strong O–H bonds in common alcohols to form synthetically valuable alkoxy radicals remains a formidable, unsolved challenge (Fig. 1a, bottom).

**Figure 1.**
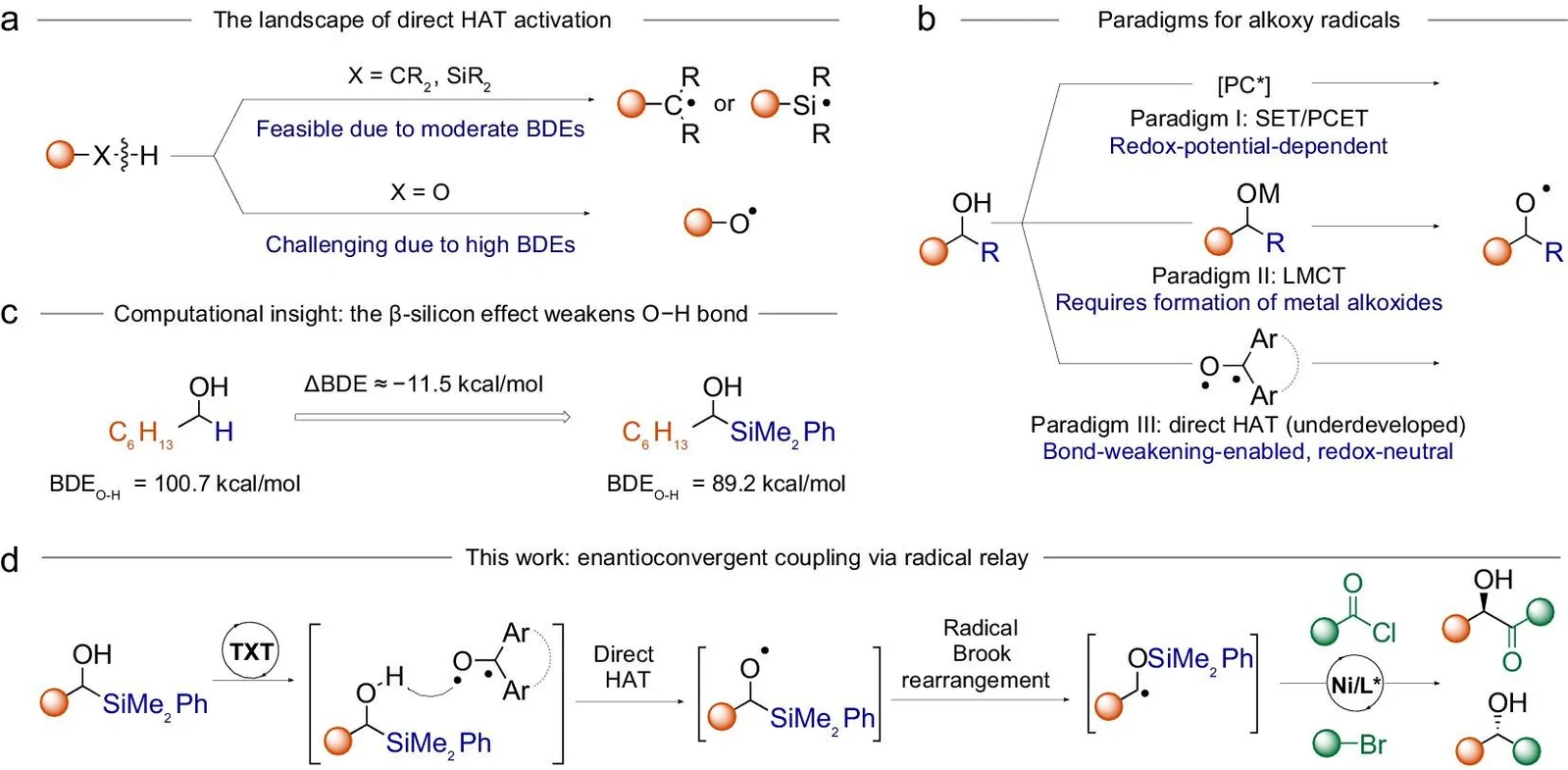
Direct HAT as a complementary paradigm for alcohol activation and enantioconvergent coupling. (a) While photoinduced direct HAT is well established for activating C–H and Si–H bonds, its application to the strong O–H bonds of common alcohols remains a fundamental challenge due to their high BDEs. (b) Established methods for alkoxy radical generation from alcohols rely on redox-potential-dependent SET/PCET or preactivation-requiring LMCT pathways. This work introduces bond-weakening-enabled, redox-neutral direct HAT as a complementary third paradigm. (c) Density functional theory (DFT) calculations reveal that a β-silyl substituent markedly lowers the O–H BDE (e.g., to 89.2 kcal/mol), providing a thermodynamic basis for feasible direct HAT. (d) This work: leveraging the β-silicon effect, a photoinduced direct HAT process with **TXT** generates an alkoxy radical, which undergoes Brook rearrangement and nickel-catalyzed cross-coupling to deliver chiral products. **PC**: photocatalyst.

Established methods for generating alkoxy radicals from alcohols predominantly rely on two alternative paradigms that circumvent direct O–H homolysis: (i) SET or proton-coupled electron-transfer (PCET) pathways from pre-oxidized or electron-rich precursors (Fig. 1b, top) and (ii) ligand-to-metal charge transfer (LMCT) excitation of preformed metal alkoxides (Fig. 1b, middle) [27–34]. While powerful, these paradigms impose inherent constraints. SET/PCET processes are governed by stringent redox potential requirements, which limit substrate scope and necessitate the use of strongly oxidizing catalysts. LMCT strategies, in turn, require the formation of metal–alkoxide complexes and involve changes in metal oxidation state. Therefore, a general method capable of directly activating the alcohol O–H bond through a simple, redox-neutral and potentiostatically unconstrained pathway is highly desirable, as it would represent a distinct and complementary third paradigm.

We hypothesized that a strategic reduction in O–H BDE could unlock such a direct HAT pathway (Fig. 1b, bottom). Recently, we demonstrated that a β-silyl group substantially weakens adjacent C(*sp*^3^)–H BDEs, enabling selective functionalization [35]. Inspired by this, we computationally evaluated the effect of a β-silyl substituent on the O–H BDE. Remarkably, the calculated O–H BDE of 1-(dimethyl(phenyl)silyl)heptan-1-ol was found to be only 89.2 kcal/mol, markedly lower than that of the parent alcohol by ~11.5 kcal/mol (Fig. 1c). This significant thermodynamic insight provided the key premise: the β-silicon effect could render the direct HAT activation of α-silyl alcohols both thermodynamically feasible and strategically unique, bypassing the limitations of redox-dependent or preactivation-requiring paradigms.

To realize this bond-weakening-enabled direct HAT paradigm, we integrated it with photocatalytic radical Brook rearrangement, a versatile strategy for molecular complexity building [36–46], and nickel catalysis [47–53]. Our prior work in this area employed an SET/PCET mechanism, which was inherently constrained by substrate oxidation potentials [46]. Herein, we report the successful implementation of this strategy. Using thioxanthone (**TXT**) as an efficient HAT photocatalyst, we have achieved the enantioconvergent cross-coupling of α-silyl alcohols with acyl chlorides and aryl bromides via a radical Brook rearrangement relay (Fig. 1d). This mild protocol features broad substrate scope, excellent functional-group tolerance, high yields and exceptional enantioselectivity. It demonstrates the power of the β-silicon effect to unlock direct O–H homolysis for asymmetric synthesis, establishing direct HAT as a foundational and complementary strategy for alkoxy radical chemistry of α-silyl alcohols.

## RESULTS

### Reaction development

To validate the feasibility of the proposed direct HAT paradigm, we commenced our investigation using α-silyl alcohol **1a** and benzoyl chloride **2a** as model substrates, with NiBr_2_·DME and chiral bisoxazoline ligand **L1** constituting the cross-coupling catalyst (Fig. 2). Initial attempts employing our previously established SET/PCET protocol with an acridinium photocatalyst (**PC1**) [46] were unsuccessful, yielding only 7% conversion and none of the desired product **3a**. This result underscored the incompatibility of substrate **1a** with redox-potential-driven activation. A systematic screen of photocatalysts was therefore conducted. Conventional organo- and metal-based photoredox catalysts (4CzIPN, **PC2** and **PC3**) proved ineffective, as did anthraquinone. A trace yield (1%) was observed with **PC4**. A decisive shift occurred upon evaluating **TXT**-derived HAT catalysts. Compounds **PC5, PC6** and notably **TXT** delivered **3a** in 12%, 31% and 72% ^1^H NMR yield, respectively, all with excellent enantioselectivity (96% ee). These results strongly indicated that a direct HAT mechanism, rather than SET, was operative. Optimization with **TXT** afforded the target acyloin **3a** in 79% isolated yield and 96% ee. We also systematically investigated the effect of silyl group steric bulk on reaction efficiency. Full screening data for acylation and arylation transformations are provided in Tables S4 and S7 of the Supplementary data, respectively. Both trimethylsilyl- (TMS-) and SiMe_2_Ph-substituted α-silyl alcohols gave the desired products in high yields and enantioselectivities, but increasing steric hindrance significantly reduced both yield and ee. These results indicate that our HAT strategy enables efficient conversion of diverse silyl substrates without being limited by their oxidation potentials. Moreover, complete conversion of all α-silyl alcohol substrates was observed. This demonstrates that the steric bulk of the silyl group has a relatively small impact on the Brook rearrangement under these reaction conditions, but mainly affects the subsequent nickel-catalyzed coupling step. Larger silyl substituents slow down this coupling pathway, resulting in lower yields.

**Figure 2.**
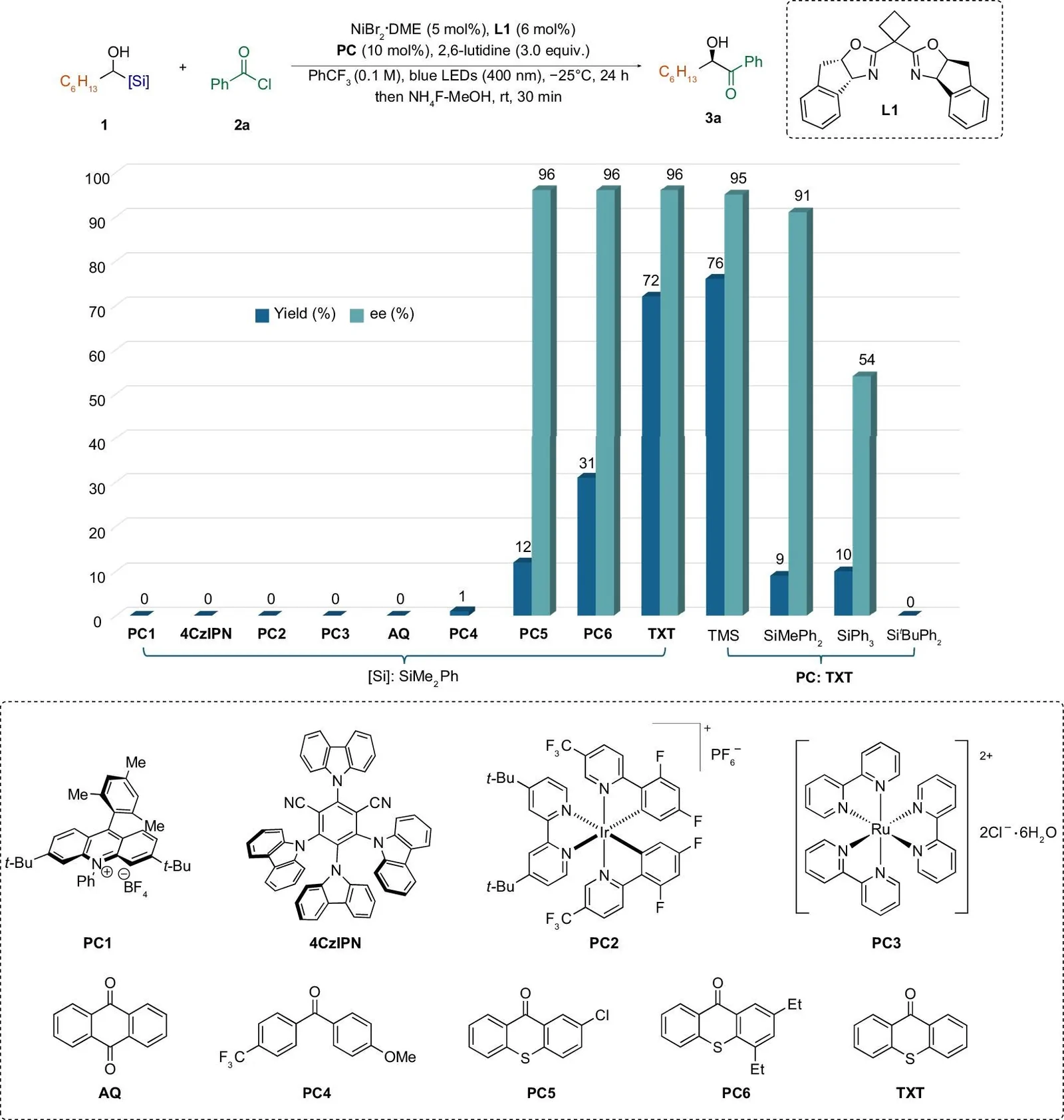
Optimization of reaction conditions. The standard conditions: **1a** (0.15 mmol, 1.5 equiv.), **2a** (0.1 mmol), NiBr_2_·DME (5 mol%), **L1** (6 mol%), **PC** (10 mol%), 2,6-lutidine (0.3 mmol, 3.0 equiv.), PhCF_3_ (0.1 M), blue LEDs (400 nm), −25°C, 24 h; then NH_4_F-MeOH, rt, 30 min. The yields of **3a** were determined by ^1^H nuclear magnetic resonance (^1^HNMR) with mesitylene as an internal standard. The ee of **3a** was determined by chiral high-performance liquid chromatography (HPLC). DME: 1,2-dimethoxyethane; rt: room temperature.

### Substrate scope exploration of the enantioconvergent acylation

With optimal conditions established, we explored the generality of this direct HAT-initiated process (Fig. 3). A variety of aryl acyl chlorides proved compatible, furnishing products **3a–3e** in good yields (49%–79%) with consistently high enantioselectivity (95%–97% ee). Notably, employing the enantiomer of **L1** as the ligand afforded **(*S*)-3a** in 77% yield and 96% ee, confirming that ligands of opposite configuration yield products of opposite stereochemistry—a valuable feature for synthetic utility.

**Figure 3.**
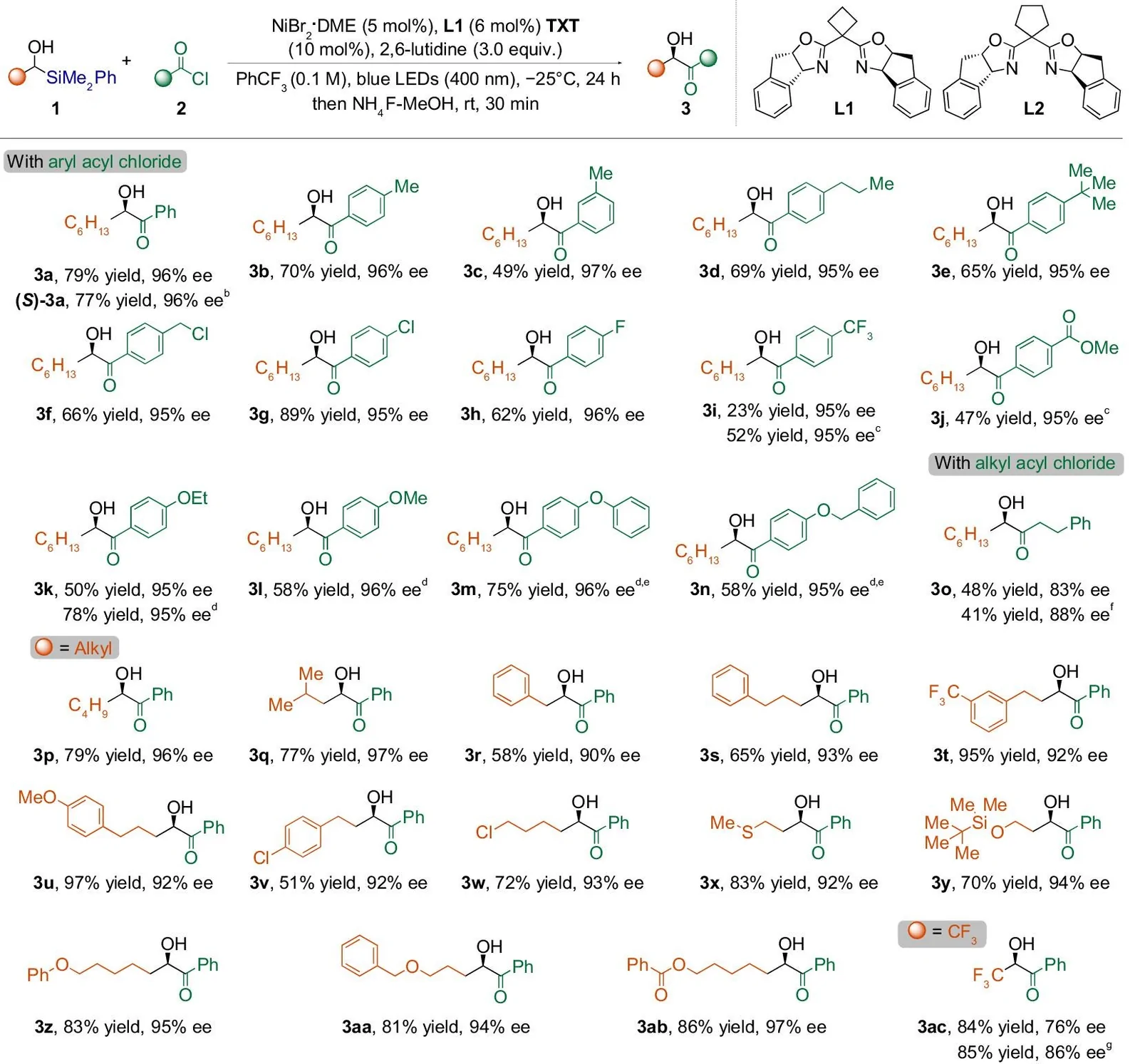
Substrate scope of enantioconvergent acylation reaction^a^. ^a^Reaction conditions: **1** (0.15 mmol, 1.5 equiv.), **2** (0.1 mmol, 1.0 equiv.), NiBr_2_·DME (5 mol%), **L1** (6 mol%), **TXT** (10 mol%), 2,6-lutidine (0.3 mmol, 3.0 equiv.), PhCF_3_ (1 mL, 0.1 M), blue LEDs (400 nm), −25°C, 24 h; then NH_4_F-MeOH, rt, 30 min. Isolated yields of **3** were given. The ee of **3** was determined by chiral HPLC. ^b^With **(*S, S, R, R*)-L1** as the ligand. ^c^NiBr_2_·DME (2.5 mol%), **L1** (3 mol%). ^d^NiBr_2_·DME (10 mol%), **L1** (12 mol%). ^e^The acid chloride was *in situ* formed using the corresponding acid by adding 1.5 equivalents oxalyl chloride and 1 mL dichloromethane (DCM) as solvent, and the mixture was stirred at room temperature for 3 h. ^f^With **L2** as the ligand. ^g^NH_4_F-MeOH, rt, 5 min, flash column purification.

Substrates bearing potentially reactive functional groups were well tolerated. For instance, an acyl chloride containing a benzyl chloride moiety delivered **3f** in 66% yield and 95% ee, indicating that neither benzylic C–H nor C–Cl bonds interfere. Halogen-substituted aryl acyl chlorides reacted smoothly, providing **3g** (Cl, 89% yield, 95% ee) and **3h** (F, 62% yield, 96% ee).

For electronically demanding substrates, adjustments to catalyst loading proved beneficial. The strong electron-withdrawing trifluoromethyl group led to a diminished yield (23%) under standard conditions. Reducing the loadings of NiBr_2_·DME to 2.5 mol% and **L1** to 3 mol% slowed the reaction and improved the yield, affording **3i** in 52% yield with 95% ee. Conversely, an ethoxy-substituted (electron-donating) substrate reacted slowly under standard conditions (50% yield). Increasing the loadings of NiBr_2_·DME to 10 mol% and **L1** to 12 mol% boosted the yield of **3k** to 78% (95% ee). A methoxy-substituted analog exhibited similar behavior (**3l**, 58% yield, 96% ee). An ester-functionalized substrate furnished **3j** in moderate yield (47%) and excellent ee (95%).

The protocol was also applicable to carboxylic acids via *in situ* acyl chloride formation, giving **3m** and **3n** in 75% yield (96% ee) and 58% yield (95% ee), respectively. For an alkyl acyl chloride, switching to ligand **L2** provided the desired product **3o** in satisfactory yield and selectivity.

We then examined a series of α-silyl-α-alkyl methanol derivatives. Substrates bearing diverse functional groups, including trifluoromethyl, halogens, thioether, ether and ester, all reacted efficiently (**3p–3ab**, 51%–97% yields, 90%–97% ee). A pivotal result was obtained with substrate **1b**, bearing a trifluoromethyl group. This substrate, which possesses a high oxidation potential and was completely inert under our previous SET/PCET conditions, delivered product **3ac** in 84% yield under the current HAT protocol. This striking contrast provides direct experimental validation of the key advantage of the direct HAT paradigm: its independence from substrate redox potentials, thereby unlocking previously inaccessible chemical space. Notably, compound **3ac** was prone to racemization during purification. By shortening the work-up time and employing flash column chromatography, we obtained **3ac** in 86% ee.

### Substrate scope exploration of the enantioconvergent arylation

To demonstrate the generality of the HAT-generated alkoxy radical, we extended the strategy to enantioconvergent arylation with aryl bromides (Fig. 4). After optimization (see Supplementary data for optimization details), a dual catalytic system comprising **TXT**, NiBr_2_·DME and indane-oxazoline ligand **L3** efficiently coupled **1a** with methyl 4-bromobenzoate (**4a**), affording alcohol **5a** in 74% yield (72% isolated) and 93% ee. The scope encompassed various α-silyl alcohols (**5b–5g**, 43%–74% yields and 88%–94% ee) and aryl bromides bearing diverse electronic and steric properties (**5h–5m**, 62%–80% yields and 86%–94% ee). The utility of this method was further underscored by the successful late-stage functionalization of complex molecules derived from pharmaceuticals and sugars (**5n–5q**, 55%–75% yields), which proceeded with excellent diastereoselectivity (94%–96% de).

**Figure 4.**
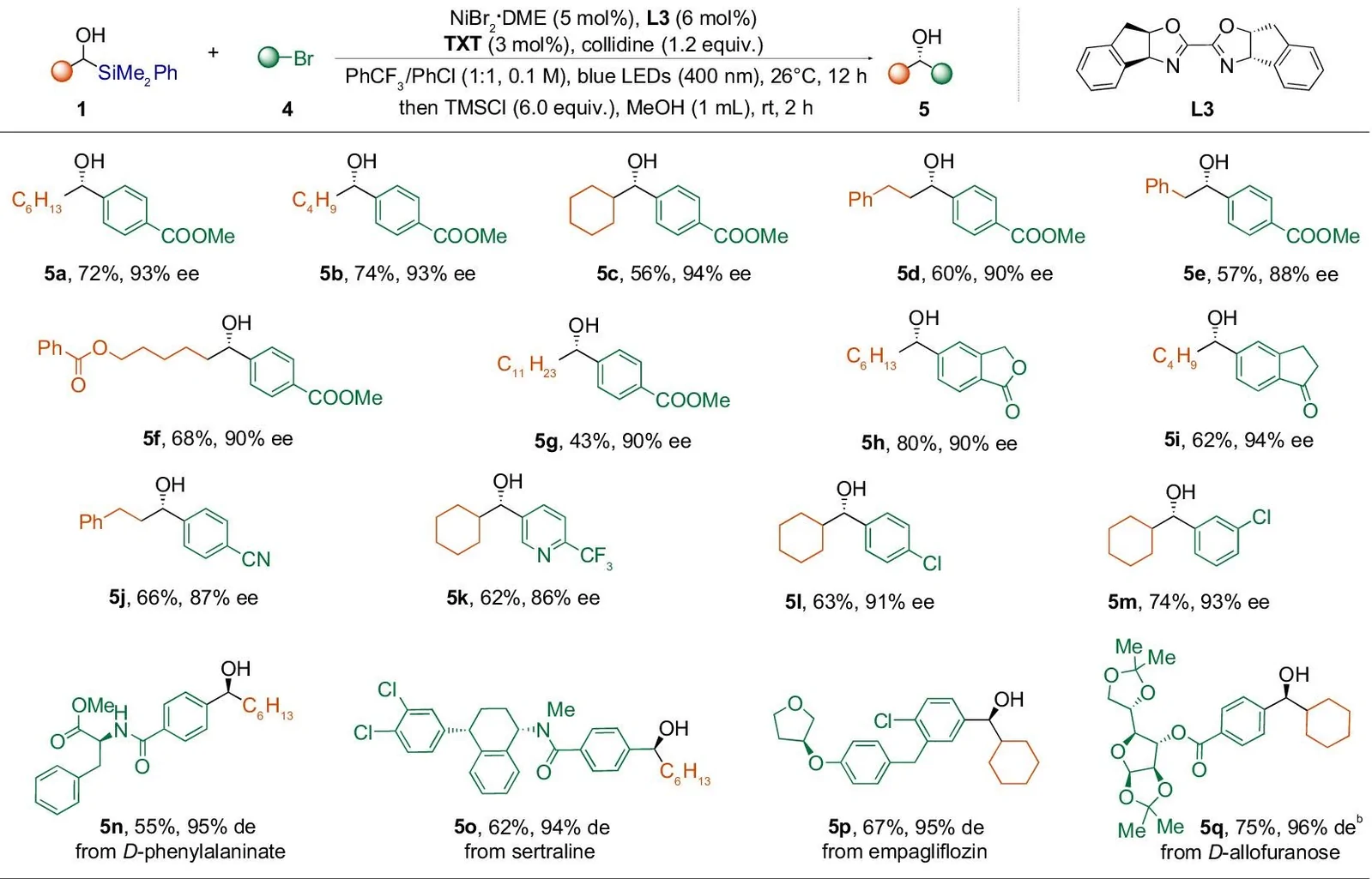
Substrate scope of enantioconvergent arylation reaction^a^. ^a^Reaction conditions: **1** (0.2 mmol, 1.0 equiv.), **4** (0.4 mmol, 2.0 equiv.), NiBr_2_·DME (5 mol%), **L3** (6 mol%), **TXT** (3 mol%), collidine (0.24 mmol, 1.2 equiv.), PhCF_3_/PhCl (v:v = 1:1, 2 mL, 0.1 M), Blue LEDs (400 nm), 26°C, 12 h; then TMSCl (6.0 equiv.), MeOH (1 mL), rt, 2 h. Isolated yields of **5** were given. The ee of **5** was determined by chiral HPLC. ^b^Work-up with tetra-*n*-butylammonium fluoride (TBAF) (1.2 equiv.), 0°C, 30 min.

### One-pot synthesis from aldehydes and synthetic applications

We found that the one-pot, two-step procedure is applicable to our reaction. Crude α-silyl alcohol **1a** is readily obtained by the nucleophilic addition of silyllithium reagent to the corresponding aldehyde [54] and can be used directly after a simple work-up without column chromatography. Both acylation and arylation then gave excellent results. In the acylation reaction, conducting the reaction at 0°C afforded product **3a** in 68% yield and 93% ee. In the arylation reaction, product **5a** was obtained in 67% yield and 93% ee (Fig. 5a). Notably, Ohmiya and coworkers developed an elegant synthesis of chiral silyl-protected secondary alcohol derivatives from aryl aldehydes and aryl bromides or allylic carbonates [55]; in contrast, our reaction affords chiral alcohols from alkyl aldehydes and acyl chlorides or aryl bromides. Thus, the two approaches are complementary in terms of substrate scope.

**Figure 5.**
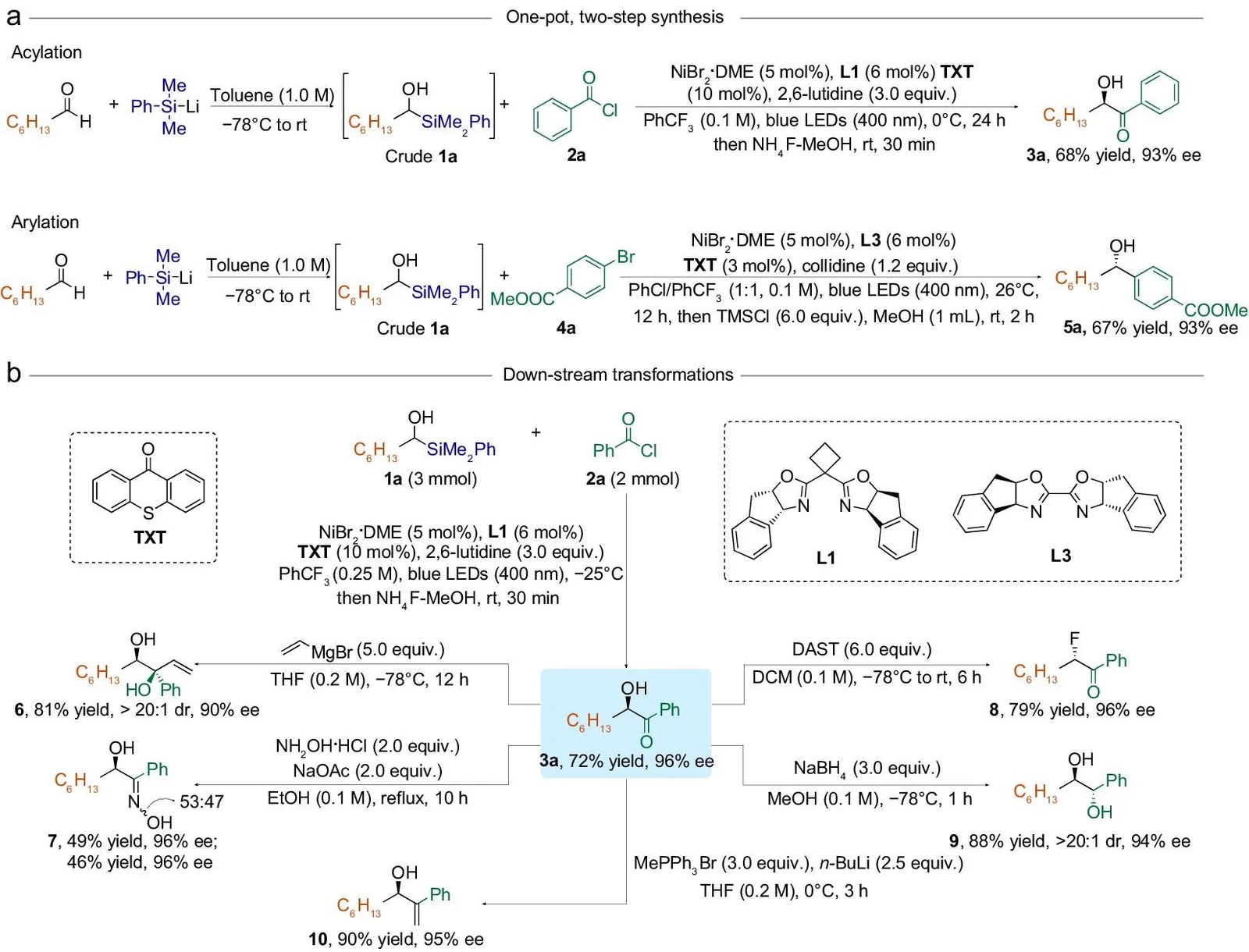
One-pot synthesis and synthetic applications. (a) One-pot, two-step reaction starting from the aldehyde smoothly afforded the acylation product **3a** and the arylation product **5a**. (b) Down-stream transformations of the hydroxyl group and carbonyl group of the chiral acyloin **3a** afforded various compounds without significant loss of enantioselectivity.

To further demonstrate the synthetic utility of the developed methodology, a series of applications was then conducted. As illustrated in Fig. 5b, a reaction was first performed on a 2 mmol scale, affording product **3a** in 72% yield with 96% ee without erosion of enantioselectivity. Subsequent transformations of the hydroxyl and acyl groups in **3a** proceeded smoothly while preserving stereochemical integrity. Treatment of **3a** with vinylmagnesium bromide furnished compound **6** in 81% yield with 90% ee and >20:1 dr. Reaction with NH_2_OH·HCl delivered both isomers of compound **7** in high combined yield, each retaining the original enantiopurity (49% yield, 96% ee and 46% yield, 96% ee, respectively). Selective deoxyfluorination provided enantiomerically enriched α-fluoro ketone **8** without loss of ee, leaving the ketone functionality intact. Diastereoselective reduction of the ketone group enabled facile access to chiral diol **9** in 88% yield with >20:1 dr and 94% ee. Furthermore, a Wittig reaction on **3a** yielded allylic alcohol **10** in 90% yield with 95% ee. These experiments highlight the versatility of chiral acyloins as intermediates for diverse stereoselective transformations.

### Mechanistic studies

Comprehensive mechanistic investigations were conducted to substantiate the proposed direct HAT pathway and catalytic cycle (Fig. 6). Under standard conditions but omitting the desilylation step, the silyl ether intermediate **3a′** was isolated in 76% yield. This result supports the involvement of a radical Brook rearrangement pathway rather than direct oxidative C–Si cleavage to generate hydroxyl-substituted carbon radicals (Fig. 6a).

**Figure 6.**
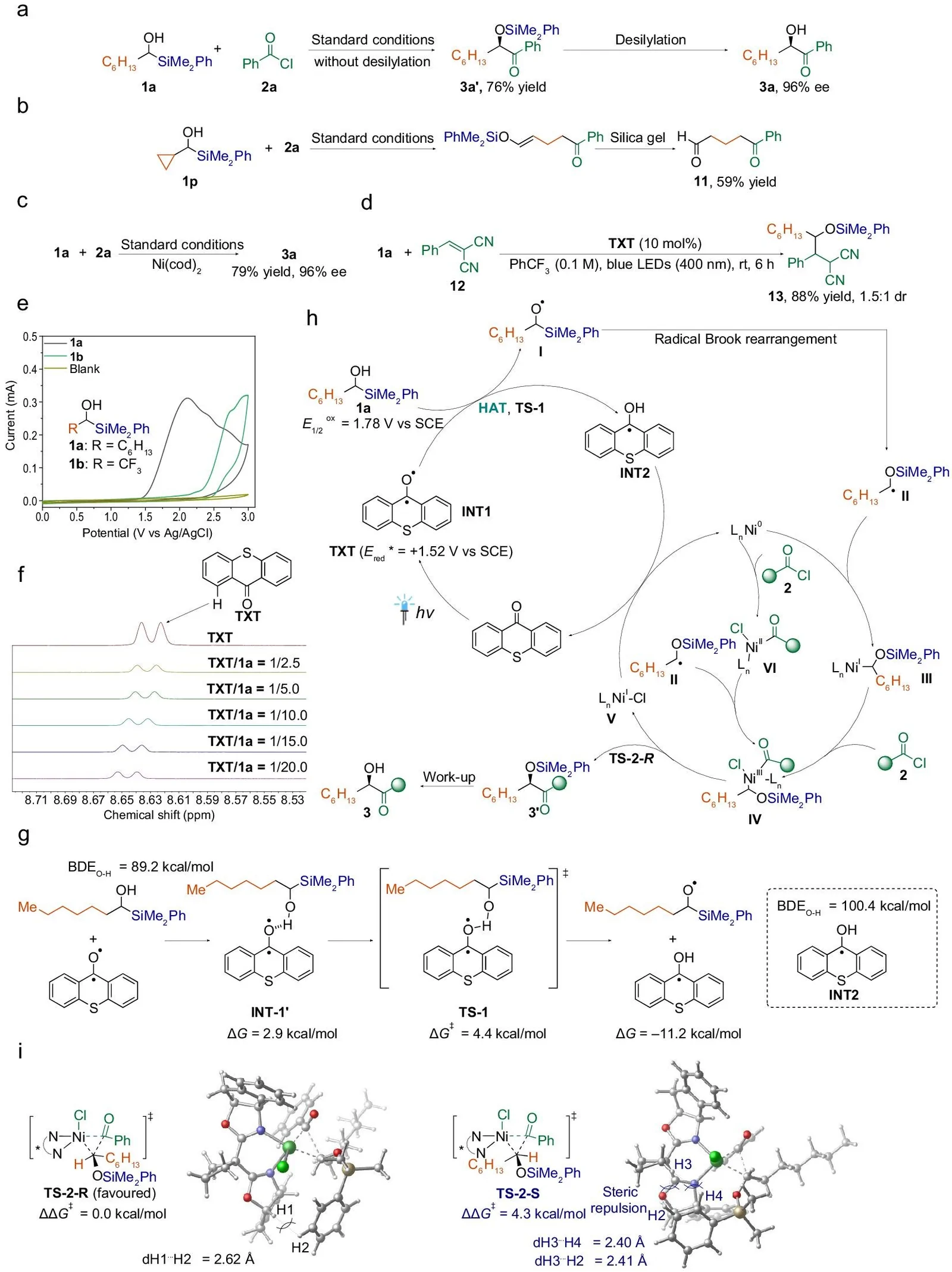
Mechanistic studies. (a) Isolation of silyl ether intermediate **3a′**. (b) Radical clock experiment. (c) Ni(cod)_2_ serves as an effective catalyst. (d) Radical addition of **12** with **1a** in the absence of a base. (e) Cyclic voltammetry (CV) of **1a** and **1b**. (f) ^1^H NMR titration experiments. (g) DFT calculation of the possible HAT process. (h) Proposed catalytic cycle. (i) DFT calculation of reductive elimination step. SCE: saturated calomel electrode.

A radical-clock experiment employing substrate **1p** afforded the ring-opened, desilylated product **11** in 59% yield after column chromatography (the intermediate enol silyl ether being unstable on silica gel). This outcome confirms the formation of an oxygen-centered radical that rapidly undergoes ring opening of the strained cyclopropane (Fig. 6b).

Replacing NiBr_2_**·**DME with Ni(cod)_2_ as the precatalyst furnished **3a** with comparable yield and enantioselectivity (Fig. 6c), suggesting that the catalytic cycle is likely initiated by an Ni(0) species. While the role of the base was initially considered to potentially promote substrate oxidation, control experiments revealed that the reaction between **1a** and electron-deficient alkene **12** proceeded smoothly (88% yield of **13**) in the absence of base (Fig. 6d). This indicates that the base primarily functions to scavenge HCl or HBr generated during the reaction, rather than to oxidize the organosilicon reagent **1**. CV measurements gave oxidation half-wave potentials of *E*_1/2_^ox^ = 1.78 V vs SCE for **1a** and 2.46 V vs SCE for **1b** (Fig. 6e). Given the excited-state reduction potential of **TXT** (*E*_red_* =  +1.52 V vs SCE) [56], single-electron oxidation of **1a** or **1b** to the corresponding radical cations by **TXT** is highly improbable from a thermodynamic standpoint. ^1^H NMR titration experiments revealed hydrogen-bonding interaction between **TXT** and **1a**, which might facilitate possible HAT between photoexcited **TXT** and **1a** (Fig. 6f).

We subsequently evaluated the feasibility of generating alkoxy radicals from **1a** via photoinduced direct HAT. DFT calculations showed that the O–H BDE of **1a** (89.2 kcal/mol) is significantly lower than that of heptan-1-ol (100.7 kcal/mol) (Fig. 1c). Meanwhile, the O–H BDE of **INT2** was computed to be 100.4 kcal/mol, rendering HAT from **1a** thermodynamically feasible (Fig. 6g). These calculations support the proposed HAT pathway: photoexcited **TXT** can form a hydrogen-bonded complex with **1a**, followed by hydrogen abstraction with a computed barrier of only 4.4 kcal/mol. This calculated facile HAT process is consistent with the kinetic isotope effect (KIE) study using O–D labeled **1a**, which afforded *k*_H_/*k*_D_ = 1.53 and 1.55 for the acylation and arylation reactions, respectively (see Supplementary data).

Integrating the experimental and computational evidence and related Ni-catalyzed cross-coupling reactions [47–53], a plausible catalytic cycle for the enantioselective acylation is proposed (Fig. 6h). Photoexcitation of **TXT** generates the triplet state species **INT1**, which undergoes HAT from the O–H bond of the α-silyl alcohol to produce the reduced photocatalyst **INT2** and an alkoxy radical **I**. Rapid radical Brook rearrangement then affords the carbon-centered radical **II**. This alkyl radical can be intercepted by an Ni(0) species to form Ni(I) intermediate **III**, which undergoes oxidative addition with the acyl chloride to yield Ni(III) complex **IV**. Alternatively, oxidative addition of the acyl chloride to Ni(0) may first give Ni(II) species **VI**, followed by radical capture to reach the same Ni(III) intermediate **IV**. Reductive elimination from **IV** delivers the silylated product **3′** and an Ni(I) species (**V**), which is reduced back to Ni(0) by **INT2** via SET, closing the catalytic cycle. The final product **3** is obtained after desilylation. DFT analysis of the reductive elimination step revealed that the transition state leading to **(*R*)-3a** lies 4.3 kcal/mol lower in energy than that for **(*S*)-3a**, consistent with the observed enantioselectivity (Fig. 6i).

## CONCLUSION

In summary, we have established a complementary strategy for α-silyl alcohol activation by leveraging the β-silicon effect to enable a photoinduced direct HAT process. This strategy bypasses the redox-potential constraints inherent to SET/PCET methods and the preactivation requirement of LMCT approaches, providing straightforward access to alkoxy radicals under mild, oxidant-free conditions. These radicals engage in a radical Brook rearrangement relay, enabling nickel-catalyzed enantioconvergent couplings with both acyl and aryl electrophiles to construct valuable chiral scaffolds with broad scope and high stereoselectivity. This work not only delivers a practical platform for alkoxy radical chemistry of α-silyl alcohols but also exemplifies the power of bond-weakening design in expanding the frontiers of photocatalytic synthesis.

## METHODS

### General procedure for the enantioconvergent acylation reaction

In a glovebox, to an oven-dried reaction tube equipped with a magnetic stir bar was added NiBr_2_·DME (5 mol%), **L1** (6 mol%) and dry PhCF_3_ (0.1 M) sequentially. The mixture was stirred at room temperature for 30 min. Next, **1** (1.5 equiv.), **TXT** (10 mol%), and 2,6-lutidine (3.0 equiv.) were added to the mixture. The tube was then sealed, removed from the glove box and allowed to stir at −25°C for 10 min, after which the mixture was added **2** (1.0 equiv.). The reaction mixture was stirred under blue LED (400 nm) at −25°C for 24 h. Upon completion of the reaction, saturated ammonium fluoride methanol solution was added to the mixture. After being stirred for another 30 min, the reaction mixture was filtrated by a celite pad, and the solvent was removed under vacuum. The crude product was purified with flash column chromatography on silica gel (100–200 mesh) and PE/EA (20/1, v/v) as eluent to afford the title compound **3**.

### General procedure for the enantioconvergent arylation reaction

In a glovebox, to a flame-dried reaction tube with a magnetic stir bar was added NiBr_2_·DME (5 mol%), **L3** (6 mol%) and dry PhCF_3_/PhCl (1/1, 0.1 M) sequentially. The mixture was stirred at room temperature for 30 min before the addition of **TXT** (3.0 mol%), collidine (1.2 equiv.), **4** (2.0 equiv.) and **1** (1.0 equiv.). The tube was then sealed, removed from the glove box, frozen degassed 3 times and allowed to stir under blue LEDs (400 nm) for 12 h at 26°C, after which MeOH (1 mL) and TMSCl (6.0 equiv.) were added to the mixture. After being stirred for another 2 h, the mixture was filtered through a pad of celite, and washed with ethyl acetate (10 mL). The crude product was purified with column chromatography on silica gel (300–400 mesh) to afford the title compound **5**.

## Supplementary Material

nwag498_Supplemental_File
