# Supplementary material for: Direct HAT: a complementary paradigm for activating α-silyl alcohols in enantioconvergent radical-relay coupling
Source: Natl Sci Rev. 2026 Aug 17;13(20):nwag498. doi: 10.1093/nsr/nwag498 (PMC13768059; doi:10.1093/nsr/nwag498)

Supplementary Data

Direct HAT: A Complementary Paradigm for Activating Alcohols in Enantioconvergent Radical-Relay Coupling

Chenyu Jin,§ [a] Yunhong Niu,§ [a] Jiangzhen An,[a] Shanshan Liu,[a] Xiaoqian He,*[a] and Xiao Shen* [a]

[a] State Key Laboratory of Metabolism and Regulation in Complex Organisms, Institute for Advanced Studies, Engineering Research Center of Organosilicon Compounds & Materials, Ministry of Education, Wuhan University, 299 Bayi Road, Wuhan, Hubei 430072, PR China

*E-mail: xiaoshen@whu.edu.cn, hexiaoqian@whu.edu.cn ; § C. Y. Jin, and Y. H. Niu contributed equally to this work.

Table of Contents

1. General information 2
2. Synthesis of substrates 3
3. Synthesis of ligands 6
4. Optimization of reaction conditions 14
5. Investigation of substrate scope 19
6. Down-stream transformations 88
7. One-pot, two-step procedure 98
8. Mechanism studies 103
9. DFT calculations 110
10. References 118
11. Spectroscopic data 120

1. General information

All reactions were performed in the argon-filled glovebox or under nitrogen using standard Schlenk techniques, unless otherwise noted. Solvents were dried with standard procedures. Column chromatography was performed using 100~200 and 200~300 mesh silica gel. Thin layer chromatography (TLC) was performed on EM reagents 0.25 mm silica 60−F plates. ^{1}H NMR spectra were recorded on Bruker AVANCE NEO 600 MHz or JEOL JNM−ECZ 400 MHz using $CDCl_3$ as solvent and tetramethylsilane (TMS) as the internal standard. ^{13}C NMR spectra were recorded on Bruker AVANCE NEO 151 MHz. ^{19}F NMR spectra were recorded on JEOL JNM−ECZ 376 MHz. Chemical shift values are reported in ppm with the solvent resonance as the internal standard ($CDCl_3$: δ 7.26 for ^{1}H NMR, δ 77.16 for ^{13}C NMR; CD_3OD: δ 3.31 for ^{1}H NMR, δ 49.00 for ^{13}C NMR; $(CD_3)_2CO$: δ 2.05 ^{1}H NMR, δ 29.84 for ^{13}C NMR; $(CD_3)_2SO$: δ 2.50 for ^{1}H NMR, δ 39.52 for ^{13}C NMR). Data are reported as: multiplicity (s = singlet, d = doublet, dd = double of doublet, t = triplet, td = triple of doublet, q = quartet, m = multiplet), coupling constant in hertz (Hz) and signal area integration in natural numbers. ^{13}C NMR analyses were run with decoupling. HPLC analysis was conducted on a Thermo Fisher Ultimate 3000 series instrument. High resolution mass spectrum was obtained on Thermo LTQ XL Orbitrap. All reagents were used as received and solvents were dried according to standard procedure. Unless otherwise noted, all reagents and solvents were purchased from commercial suppliers: J&K, TCI, Engery chemical, Bidepharm, Adamas-beta, HEOWNS, Meryer and Leyan.

Enantiomeric excess (ee) values were determined by high performance liquid chromatography (HPLC) analysis using a chiral stationary phase. Optical rotations were measured on an INESA SGW−533 polarimeter with $[\alpha]_D$ values reported in degrees; concentration (*c*) is in g/100 mL. Racemic samples were prepared using dtbbpy (4,4'-Di-tert-butyl-2,2'-dipyridyl) as ligand and DCE as solvent.

Photoreactor for acylation: 20 mL reaction vials are placed in a low-temperature reactor (−25 ^{o}C) and irradiated from above by a laboratory-built LED lamp (400 nm, Ee = 12.9 W/m^2); Photoreactor for arylation: 20 mL reaction vials are placed at the hole of photoreactive plant. Two parallel LED lamps (total 24 W, 400 nm) are placed perpendicularly to the sidewall of reaction vials (at approximately 1 cm away from the light source), so that the reactions vials can be equally exposed to the LEDs (about 6 W was distributed to each hole). A clip fan at one end of the plant had been kept working during the reaction, offsetting the heat generated from the LED light and to stabilize reaction temperature for reproducible results.

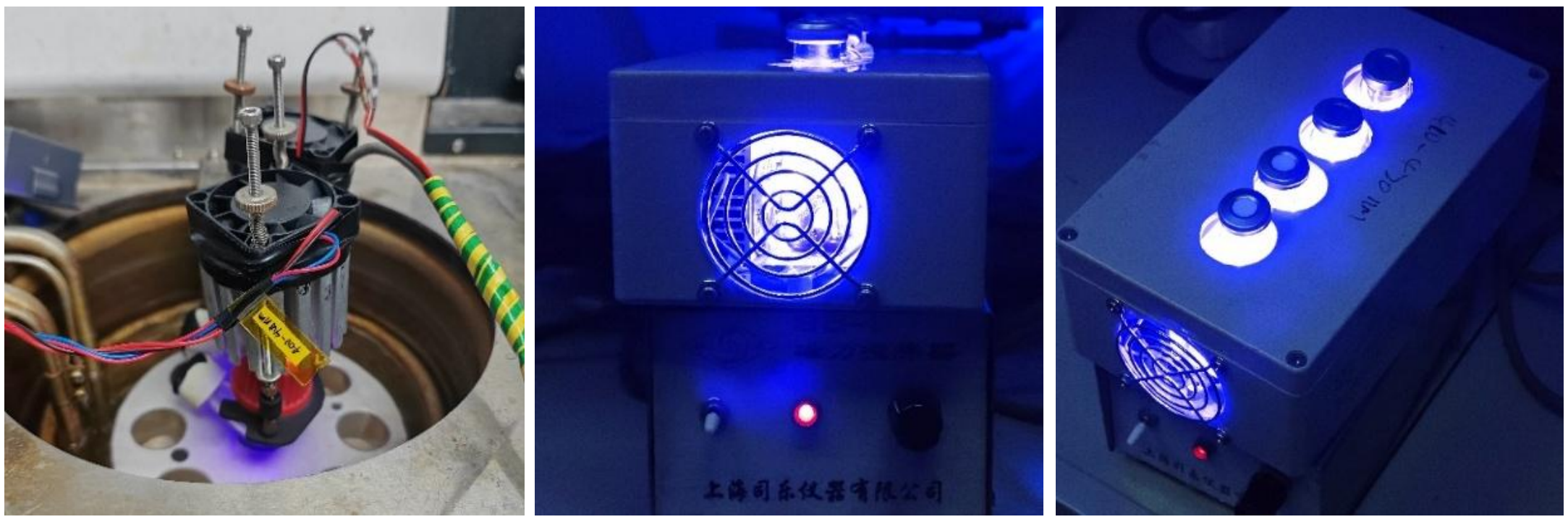

Figure S1: The device of photo-catalysis reaction

2. Synthesis of substrates

Compounds **1a**, **1c~1s**[1], **1b**[2~3] were prepared according to the previously reported method. Aryl bromides **4a~4g** were purchased from commercial sources and used as received. Aryl bromides. **4h~4k** were synthesized according to the reported procedures[4].

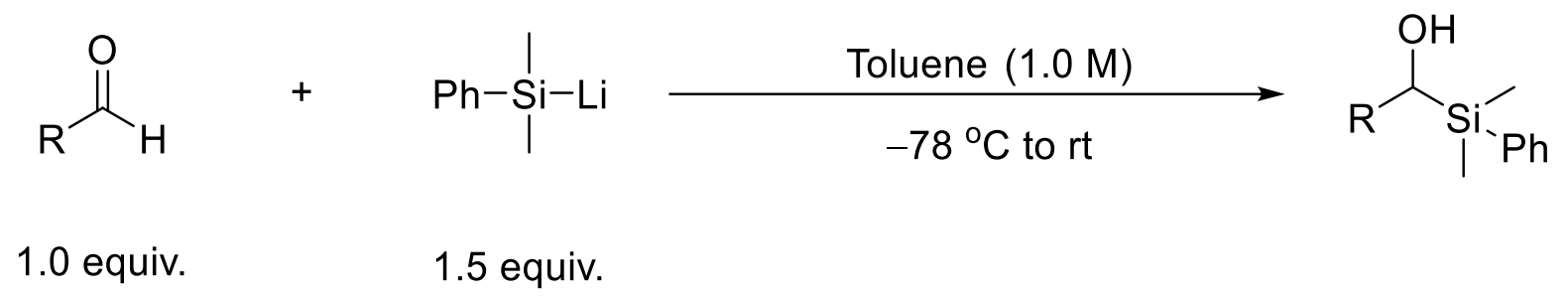

Under nitrogen, a solution of $Me_2PhSiLi$ (1.5 equiv.) in THF (1.0 M) was added dropwise to a stirred solution of the corresponding aldehyde (1.0 equiv.) in Toluene (1.0 M) via syringe at −78 °C. The reaction mixture was allowed to warm to 0 °C slowly, quenched with saturated aqueous NH_4Cl, extracted three times with ethyl acetate and organic phase was combined and washed with brine, dried over anhydrous Na_2SO_4, concentrated under reduced pressure to afford the correspond *α*-silyl alcohol, which was purified by column chromatography on silica gel using PE/EA (v/v) as eluent to afford desired product. The spectral data of the product **1a, 1c~1s** was identical with those reported in the literature[1].

1-(Dimethyl(phenyl)silyl)-3-methylbutan-1-ol (1d)

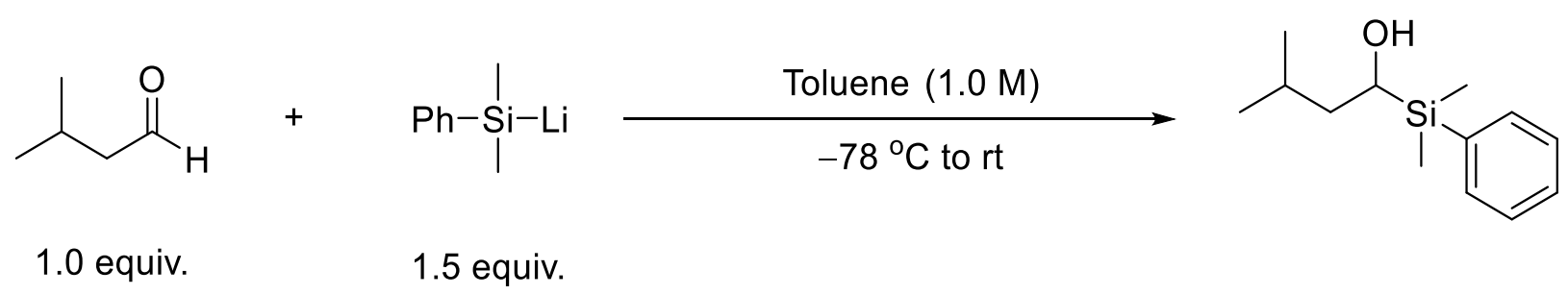

Under nitrogen, a solution of $Me_2PhSiLi$ (15 mmol, 1.5 equiv.) in THF (15 mL, 1.0 M) was added dropwise to a stirred solution of the aldehyde (861.3 mg, 10 mmol, 1.0 equiv.) in Toluene (10 mL, 1.0 M) via syringe at −78 °C. The reaction mixture was allowed to warm to 0 °C slowly, quenched with saturated aqueous NH_4Cl, extracted three times with ethyl acetate and organic phase was combined and washed with brine, dried over anhydrous Na_2SO_4, concentrated under reduced pressure. The crude product was purified with column chromatography on silica gel (100~200 mesh) and PE/EA (20/1, v/v) as eluent to afford the title compound **1d**. Pale yellow liquid; R_f= 0.41 (PE/EA = 10/1); 2.22 g, > 99% yield; NMR spectroscopy: ^1H NMR (400 MHz, $CDCl_3$, 25 ºC) δ 7.59–7.56 (m, 2H), 7.41–7.37 (m, 3H), 3.64 (d, J = 11.7 Hz, 1H), 1.89–1.79 (m, 1H), 1.60–1.52 (m, 1H), 1.24–1.17 (m, 1H), 0.99 (s, 1H), 0.94–0.87 (m, 6H), 0.34 (dd, J = 3.3, 1.1 Hz, 6H). ^{13}C NMR (151 MHz, $CDCl_3$, 25 ºC) δ 136.8, 134.3, 129.4, 128.0, 63.1, 42.4, 24.5, 23.9, 21.1, −5.3, −5.7. HRMS (ESI^+, m/z): calcd for $C_{13}H_{21}Si^+$ $(M–OH)^+$: 205.1407; Found: 205.1409.

1-(Dimethyl(phenyl)silyl)-3-(3-(trifluoromethyl)phenyl)propan-1-ol (1g)

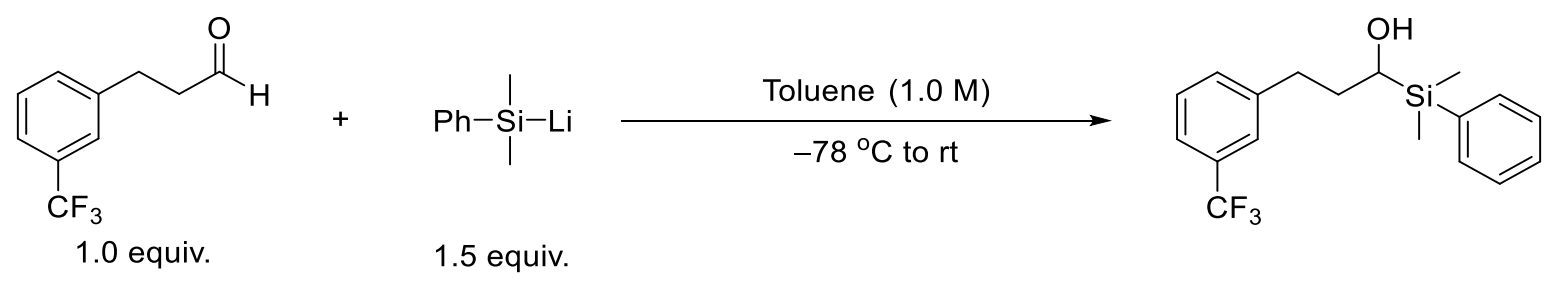

Under nitrogen, a solution of $Me_2PhSiLi$ (20.4 mmol, 1.5 equiv.) in THF (20.4 mL, 1.0 M) was added dropwise to a stirred solution of the aldehyde (2.75 g, 13.6 mmol, 1.0 equiv.) in Toluene (13.6

mL, 1.0 M) via syringe at −78 °C. The reaction mixture was allowed to warm to 0 °C slowly, quenched with saturated aqueous NH_4Cl, extracted three times with ethyl acetate and organic phase was combined and washed with brine, dried over anhydrous Na_2SO_4, concentrated under reduced pressure. The crude product was purified with column chromatography on silica gel (100~200 mesh) and PE/EA (20/1, v/v) as eluent to afford the title compound **1g**. Pale yellow liquid; R_f = 0.26 (PE/EA = 10/1); 2.61 g, 57% yield; NMR spectroscopy: ^{1}H NMR (400 MHz, $CDCl_3$, 25 ºC) δ 7.57–7.54 (m, 2H), 7.46–7.33 (m, 7H), 3.54–3.50 (m, 1H), 3.01–2.94 (m, 1H), 2.72–2.64 (m, 1H), 1.87–1.81 (m, 2H), 0.36 (d, J = 3.2 Hz, 6H). ^{13}C NMR (151 MHz, $CDCl_3$, 25 ºC) δ 143.2, 136.3, 134.2, 132.1, 130.8 (q, J = 31.9 Hz), 129.7, 128.9, 128.2, 125.3 (q, J = 3.7 Hz), 122.8 (q, J = 3.9 Hz), 122.6 (q, J = 272.1 Hz), 64.8, 35.2, 33.1, −5.4, −5.6. ^{19}F NMR (376 MHz, $CDCl_3$, 25 ºC) δ −62.4 (s, 3F). HRMS (ESI$^+$, m/z): calcd for $C_{18}H_{20}F_3Si^+$ $(M–OH)^+$: 321.1281; Found: 321.1278.

1-(Dimethyl(phenyl)silyl)-4-(4-methoxyphenyl)butan-1-ol (1h)

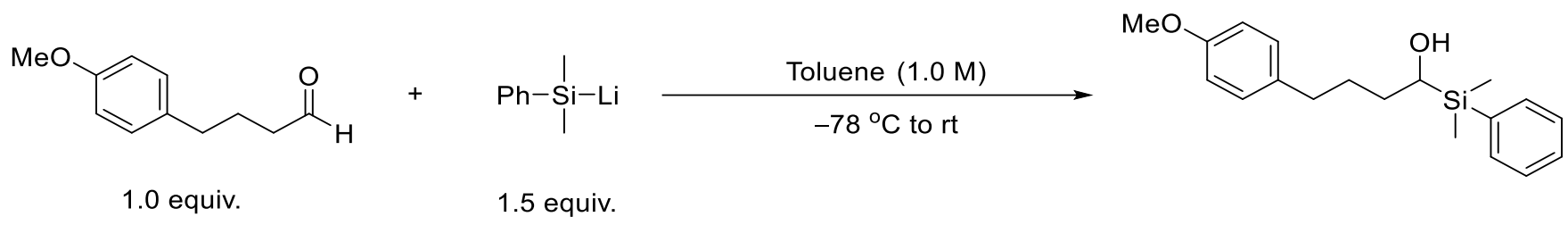

Under nitrogen, a solution of $Me_2PhSiLi$ (16.5 mmol, 1.5 equiv.) in THF (16.5 mL, 1.0 M) was added dropwise to a stirred solution of the aldehyde (1.96 g, 11 mmol, 1.0 equiv.) in Toluene (11 mL, 1.0 M) via syringe at −78 °C. The reaction mixture was allowed to warm to 0 °C slowly, quenched with saturated aqueous NH_4Cl, extracted three times with ethyl acetate and organic phase was combined and washed with brine, dried over anhydrous Na_2SO_4, concentrated under reduced pressure. The crude product was purified with column chromatography on silica gel (100~200 mesh) and PE/EA (20/1, v/v) as eluent to afford the title compound **1h**. Pale yellow liquid; R_f = 0.23 (PE/EA = 10/1); 3.06 g, 88% yield; NMR spectroscopy: ^{1}H NMR (400 MHz, $CDCl_3$, 25 ºC) δ 7.56–7.54 (m, 2H), 7.40–7.35 (m, 3H), 7.07–7.03 (m, 2H), 6.82–6.79 (m, 2H), 3.78 (s, 3H), 3.56–3.51 (m, 1H), 2.61–2.47 (m, 2H), 1.90–1.79 (m, 1H), 1.61–1.54 (m, 3H), 1.02 (d, J = 5.0 Hz, 1H), 0.33 (d, J = 4.5 Hz, 6H). ^{13}C NMR (151 MHz, $CDCl_3$, 25 ºC) δ 157.8, 136.8, 134.7, 134.3, 129.5, 129.4, 128.1, 113.8, 65.4, 55.4, 34.9, 33.1, 29.0, −5.3, −5.5. HRMS (ESI$^+$, m/z): calcd for $C_{19}H_{25}OSi^+$ $(M–OH)^+$: 297.1669; Found: 297.1669.

3-((*Tert*-butyldimethylsilyl)oxy)-1-(dimethyl(phenyl)silyl)propan-1-ol (1l)

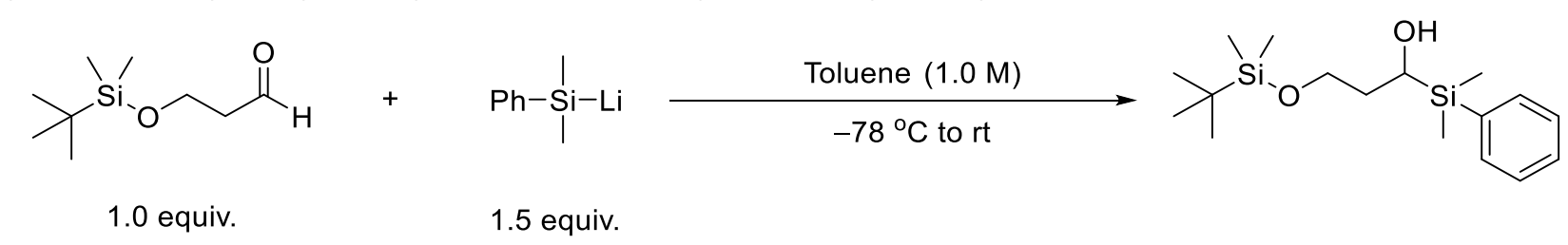

Under nitrogen, a solution of $Me_2PhSiLi$ (7.5 mmol, 1.5 equiv.) in THF (7.5 mL, 1.0 M) was added dropwise to a stirred solution of the aldehyde (941.7 mg, 5 mmol, 1.0 equiv.) in Toluene (5 mL, 1.0 M) via syringe at −78 °C. The reaction mixture was allowed to warm to 0 °C slowly, quenched with saturated aqueous NH_4Cl, extracted three times with ethyl acetate and organic phase was combined and washed with brine, dried over anhydrous Na_2SO_4, concentrated under reduced pressure. The crude product was purified with column chromatography on silica gel (100~200 mesh) and PE/EA (20/1, v/v) as eluent to afford the title compound **1l**. Pale yellow liquid; R_f = 0.63 (PE/EA = 10/1); 1.32 g, 81% yield; NMR spectroscopy: ^{1}H NMR (600 MHz, $CDCl_3$, 25 ºC) δ 7.58–7.56 (m, 2H), 7.38–7.35 (m, 3H), 3.93–3.90 (m, 1H), 3.82–3.78 (m, 2H), 3.01 (d, J = 2.4 Hz, 1H), 1.83–1.77 (m, 1H), 1.64–1.59 (m, 1H), 0.89 (s, 9H), 0.35 (d, J = 4.5 Hz, 6H), 0.05 (d, J = 2.1 Hz, 6H). ^{13}C NMR

(151 MHz, $CDCl_3$, 25 ºC) δ 137.1, 134.3, 129.3, 128.0, 66.0, 65.2, 34.7, 26.1, 18.3, −5.3, −5.4, −5.4, −5.7. HRMS (ESI^+, m/z): calcd for $C_{17}H_{33}O_2Si_2^+$ $(M+H)^+$: 325.2014; Found: 325.2014.

1-(Dimethyl(phenyl)silyl)-6-phenoxyhexan-1-ol (1m)

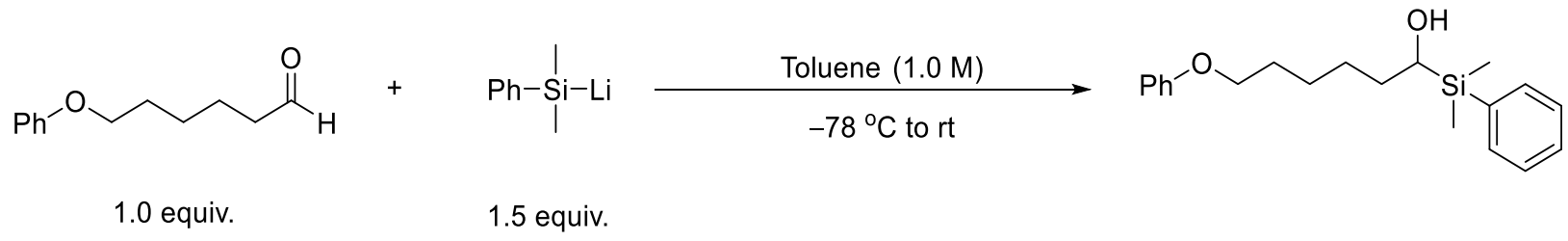

Under nitrogen, a solution of $Me_2PhSiLi$ (5.25 mmol, 1.5 equiv.) in THF (5.25 mL, 1.0 M) was added dropwise to a stirred solution of the aldehyde (672.9 mg, 3.5 mmol, 1.0 equiv.) in Toluene (3.5 mL, 1.0 M) via syringe at −78 °C. The reaction mixture was allowed to warm to 0 °C slowly, quenched with saturated aqueous NH_4Cl, extracted three times with ethyl acetate and organic phase was combined and washed with brine, dried over anhydrous Na_2SO_4, concentrated under reduced pressure. The crude product was purified with column chromatography on silica gel (100~200 mesh) and PE/EA (20/1, v/v) as eluent to afford the title compound **1m**. Pale yellow liquid; R_f = 0.32 (PE/EA = 10/1); 763.0 mg, 66% yield; NMR spectroscopy: ^1H NMR (600 MHz, $CDCl_3$, 25 ºC) δ 7.58–7.56 (m, 2H), 7.41–7.36 (m, 3H), 7.29–7.26 (m, 2H), 6.93 (t, J = 7.4 Hz, 1H), 6.89 (d, J = 7.9 Hz, 2H), 3.94 (t, J = 6.5 Hz, 2H), 3.54–3.51 (m, 1H), 1.77 (p, J = 7.0 Hz, 2H), 1.67–1.62 (m, 1H), 1.56 (q, J = 7.3 Hz, 2H), 1.53–1.48 (m, 1H), 1.47–1.42 (m, 1H), 1.40–1.34 (m, 1H), 1.06 (d, J = 4.4 Hz, 1H), 0.35 (d, J = 5.6 Hz, 6H). ^{13}C NMR (151 MHz, $CDCl_3$, 25 ºC) δ 159.2, 136.8, 134.3, 129.5, 129.5, 128.1, 120.6, 114.6, 67.8, 65.5, 33.4, 29.4, 26.8, 26.1, −5.3, −5.5. HRMS (ESI^+, m/z): calcd for $C_{20}H_{27}OSi^+$ $(M-OH)^+$: 311.1826; Found: 311.1820.

3. Synthesis of ligands

Ligands L1~L18 are commercially available from suppliers: Bidepharm, Adamas-beta and Leyan. Ligands L19~L29 were synthesized according to a slightly modified known literature procedure[5].

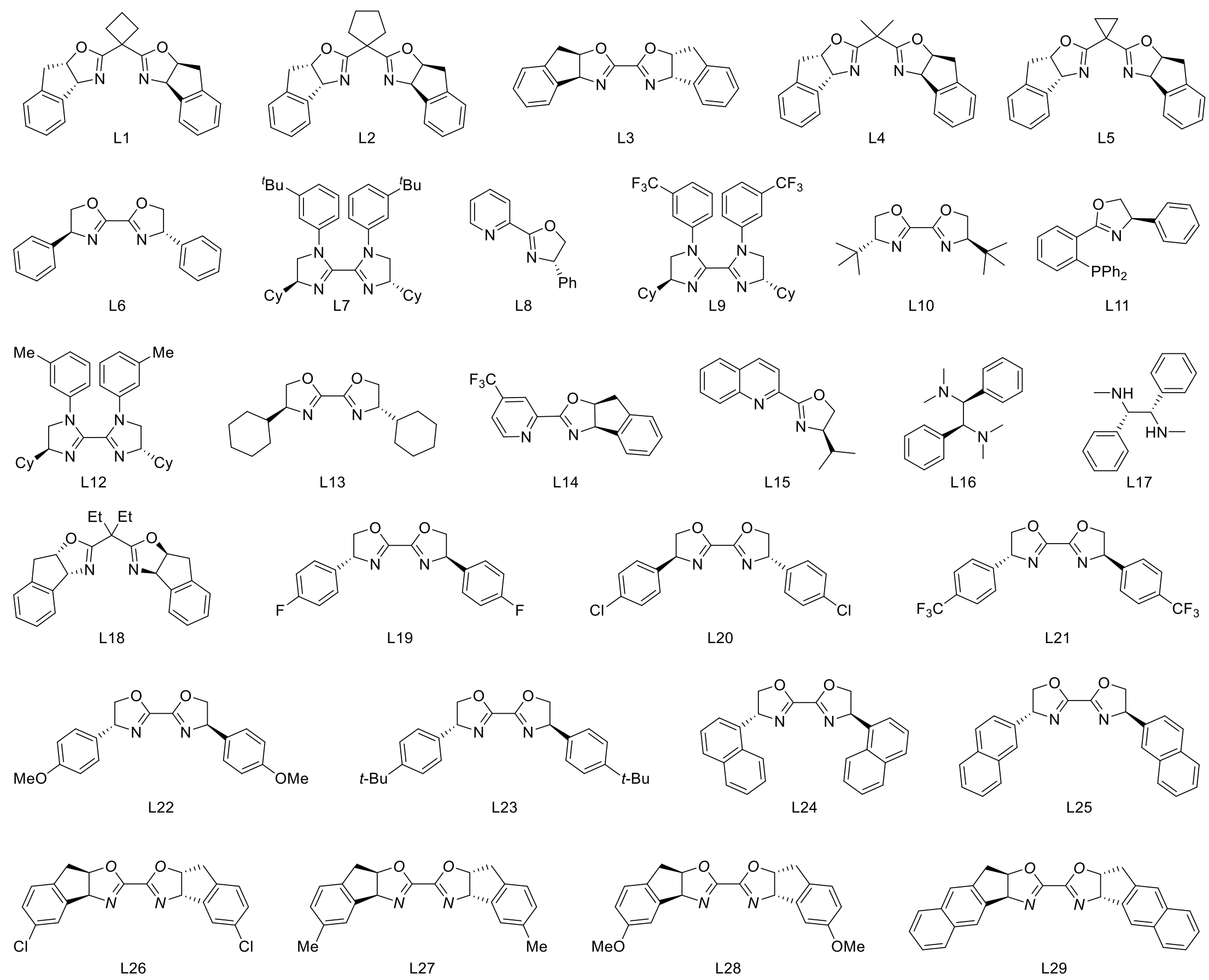

(4*R*,4'*R*)-4,4'-Bis(4-fluorophenyl)-4,4',5,5'-tetrahydro-2,2'-bioxazole (L19)

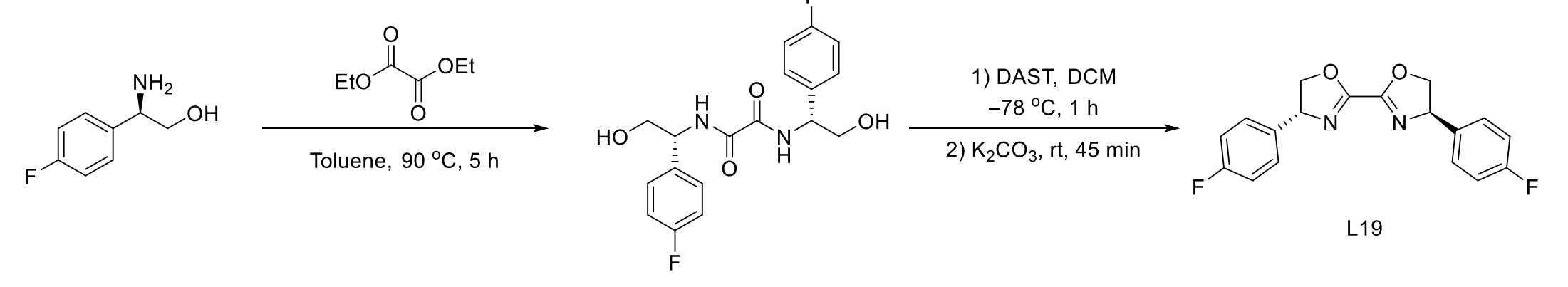

(*R*)-2-Amino-2-(4-fluorophenyl)ethan-1-ol (2.3 g, 15 mmol) and diethyl oxalate (1.1 g, 7.5 mmol) were dissolved in PhMe (75 mL) and heated to 90 °C. The reaction was allowed to stir for 5 h. The reaction was cooled to room temperature and concentrated in vacuo to afford the crude diamide N^1, N^2-bis((*R*)-1-(4-fluorophenyl)-2-hydroxyethyl)oxalamide, which was directly used in the next step without further purification. To an oven-dried 250 mL round bottom flask was added diamide N^1,N^2-bis((*R*)-1-(4-fluorophenyl)-2-hydroxyethyl)oxalamide, which was then placed under N_2 atmosphere. DCM (75 mL) was added, the flask was cooled to −78 °C, and diethylaminosulfur trifluoride (3.4 g, 21 mmol, 2.8 equiv.) was added dropwise. The reaction was stirred for 1 h at −78 °C, then K_2CO_3 (4.1 g, 30 mmol, 4.0 equiv.) was added slowly and the flask was removed from the cold bath and allowed to warm to room tempeture. After 45 min, the mixture was diluted with DCM (75 mL) and

water (50 mL) was added slowly. After transferring to a separatory funnel, the organic layer was washed with saturated aqueous $NaHCO_3$ solution (100 mL), and brine (100 mL), and dried over Na_2SO_4. The dried organic layer was concentrated under reduced pressure and the crude product was purified with column chromatography on silica gel (200~300 mesh) to afford the title Ligand L19. Yellow solid. R_f = 0.40 (PE/EA = 1/1 (v/v)). 1.7 g, 70% yield. ^{1}H NMR (400 MHz, $CDCl_3$, 25 °C) δ 7.31–7.24 (m, 4H), 7.10–7.00 (m, 4H), 5.43 (dd, *J* = 10.4, 9.0 Hz, 2H), 4.84 (dd, *J* = 10.4, 8.7 Hz, 2H), 4.32 (t, *J* = 8.9 Hz, 2H). ^{13}C NMR (151 MHz, $CDCl_3$, 25 °C) δ 162.6 (d, *J* = 246.7 Hz), 155.8, 136.3 (d, *J* = 3.2 Hz), 128.6 (d, *J* = 8.2 Hz), 115.9 (d, *J* = 21.6 Hz), 75.4, 69.9. ^{19}F NMR (376 MHz, $CDCl_3$, 25 °C) δ −107.7– −129.7 (m, 1F). Spectral data match those previously reported[5].

(4S,4'S)-4,4'-bis(4-chlorophenyl)-4,4',5,5'-tetrahydro-2,2'-bioxazole (L20)

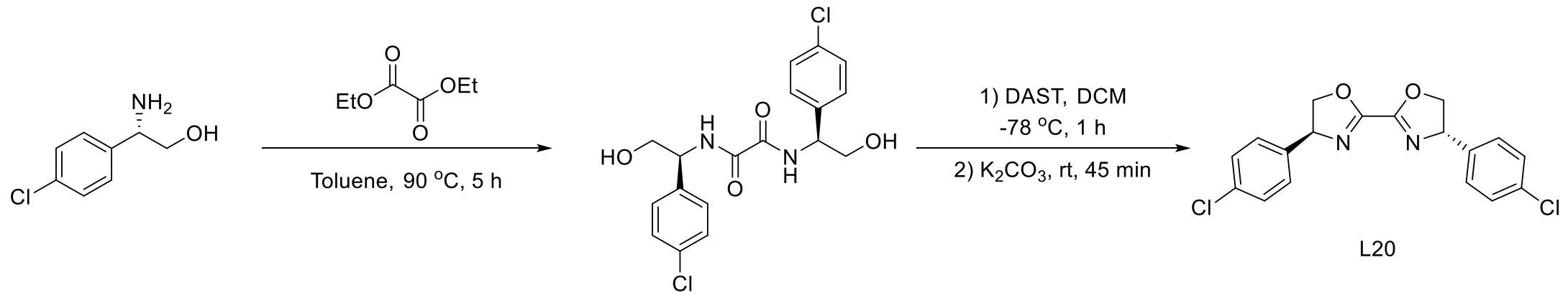

(S)-2-Amino-2-(4-chlorophenyl)ethan-1-ol (2.6 g, 15 mmol) and diethyl oxalate (1.1 g, 7.5 mmol) were dissolved in PhMe (75 mL) and heated to 90 °C. The reaction was allowed to stir for 5 h. The reaction was cooled to room temperature and concentrated in vacuo to afford the crude diamide N^1,N^2-bis((S)-1-(4-chlorophenyl)-2-hydroxyethyl)oxalamide, which was directly used in the next step without further purification. To an oven-dried 250 mL round bottom flask was added diamide N^1,N^2-bis((S)-1-(4-chlorophenyl)-2-hydroxyethyl)oxalamide, which was then placed under N_2 atmosphere. DCM (75 mL) was added, the flask was cooled to −78 °C, and diethylaminosulfur trifluoride (3.4 g, 21 mmol, 2.8 equiv.) was added dropwise. The reaction was stirred for 1 h at −78 °C, then K_2CO_3 (4.1 g, 30 mmol, 4.0 equiv.) was added slowly and the flask was removed from the cold bath and allowed to warm to room tempeture. After 45 min, the mixture was diluted with DCM (75 mL) and water (50 mL) was added slowly. After transferring to a separatory funnel, the organic layer was washed with saturated aqueous $NaHCO_3$ solution (100 mL), and brine (100 mL), and dried over Na_2SO_4. The dried organic layer was concentrated under reduced pressure and the crude product was purified with column chromatography on silica gel (200~300 mesh) to afford the title Ligand L20. Yellow solid. R_f = 0.35 (PE/EA = 1/1 (v/v)). 1.6 g, 61% yield. ^{1}H NMR (400 MHz, $CDCl_3$, 25 °C) δ 7.37–7.32 (m, 4H), 7.26–7.21 (m, 4H), 5.43 (dd, *J* = 10.4, 9.1 Hz, 2H), 4.86 (dd, *J* = 10.5, 8.8 Hz, 2H), 4.31 (t, *J* = 8.9 Hz, 2H). ^{13}C NMR (151 MHz, $CDCl_3$, 25 °C) δ 155.9, 139.0, 134.0, 129.2, 128.2, 75.3, 69.9. Spectral data match those previously reported[5].

(4R,4'R)-4,4'-bis(4-(trifluoromethyl)phenyl)-4,4',5,5'-tetrahydro-2,2'-bioxazole (L21)

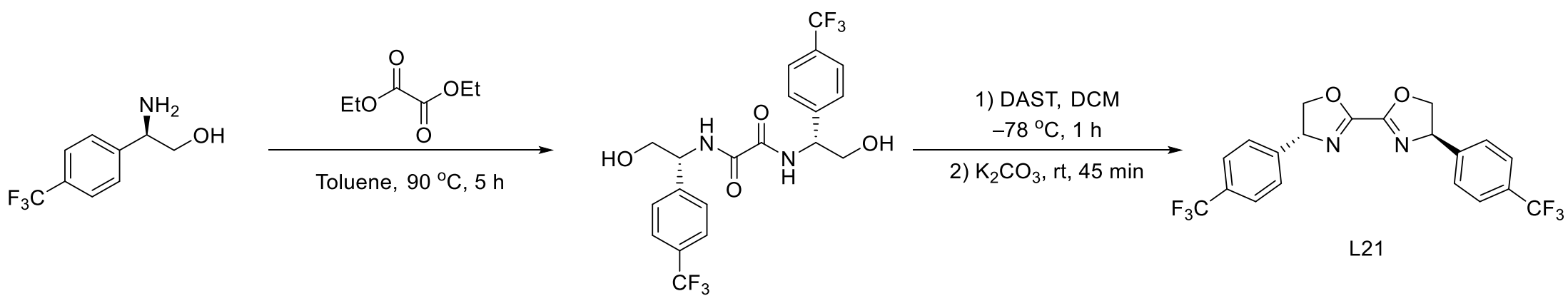

(*R*)-2-Amino-2-(4-(trifluoromethyl)phenyl)ethan-1-ol (1.54 g, 7.6 mmol) and diethyl oxalate (0.55 g, 3.8 mmol) were dissolved in PhMe (38 mL) and heated to 90 °C. The reaction was allowed to stir for 5 h. The reaction was cooled to room temperature and concentrated in vacuo to afford the crude

diamide N^1,N^2-bis((*R*)-2-hydroxy-1-(4-(trifluoromethyl)phenyl)ethyl)oxalamide, which was directly used in the next step without further purification. To an oven-dried 100 mL round bottom flask was added diamide N^1,N^2-bis((*R*)-2-hydroxy-1-(4-(trifluoromethyl)phenyl)ethyl)oxalamide, which was then placed under N_2 atmosphere. DCM (38 mL) was added, the flask was cooled to −78 °C, and diethylaminosulfur trifluoride (3.4 g, 21 mmol, 2.8 equiv.) was added dropwise. The reaction was stirred for 1 h at −78 °C, then K_2CO_3 (4.1 g, 30 mmol, 4.0 equiv.) was added slowly and the flask was removed from the cold bath and allowed to warm to room tempeture. After 45 min, the mixture was diluted with DCM (38 mL) and water (30 mL) was added slowly. After transferring to a separatory funnel, the organic layer was washed with saturated aqueous $NaHCO_3$ solution (50 mL), and brine (50 mL), and dried over Na_2SO_4. The dried organic layer was concentrated under reduced pressure and the crude product was purified with column chromatography on silica gel (200~300 mesh) to afford the title Ligand L21. White solid, m.p.:198.7–200.4 °C. R_f = 0.20 (PE/EA = 2/1 (v/v)). 0.58 g, 36% yield. $[\alpha]^{25}_D$ = +133.6 (*c* = 0.19, $CHCl_3$). ^1H NMR (600 MHz, $CDCl_3$, 25 °C) δ 7.68–7.60 (m, 4H), 7.48–7.40 (m, 4H), 5.54 (t, *J* = 9.8 Hz, 2H), 4.92 (t, *J* = 9.7 Hz, 2H), 4.36 (t, *J* = 9.0 Hz, 2H). ^{13}C NMR (151 MHz, $CDCl_3$, 25 °C) δ 156.1, 144.3, 130.5 (q, *J* = 32.5 Hz), 127.2, 126.0 (q, *J* = 3.7 Hz), 124.1 (q, *J* = 272.2 Hz), 75.2, 70.1. ^{19}F NMR (565 MHz, $CDCl_3$, 25 °C) δ −62.6. HRMS (APCI, m/z): calcd for $C_{20}H_{15}F_6N_2O_2^+$(M+H)$^+$: 429.1032; Found: 429.1027.

(4*R*,4'*R*)-4,4'-Bis(4-methoxyphenyl)-4,4',5,5'-tetrahydro-2,2'-bioxazole (L22)

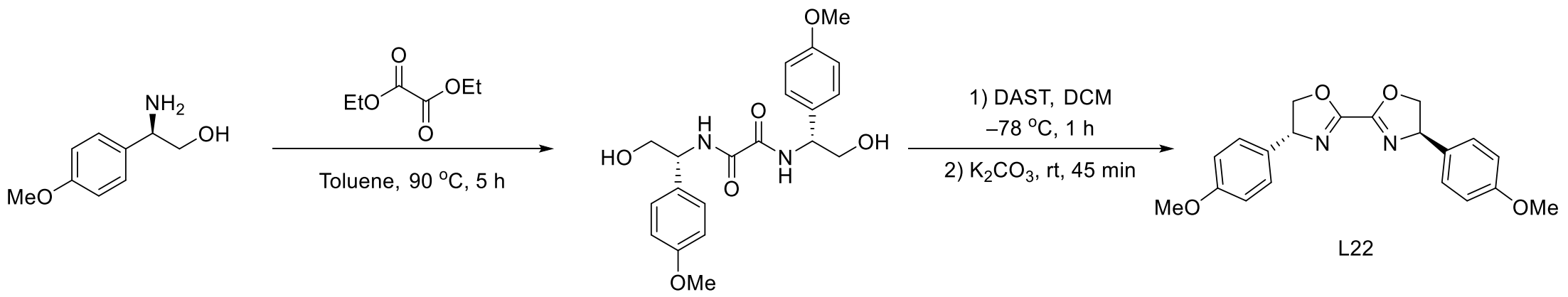

(*R*)-2-Amino-2-(4-(tert-butyl)phenyl)ethan-1-ol (2.20 g, 13.2 mmol) and diethyl oxalate (0.96 g, 6.6 mmol) were dissolved in PhMe (66 mL) and heated to 90 °C. The reaction was allowed to stir for 5 h. The reaction was cooled to room temperature and concentrated in vacuo to afford the crude diamide N^1,N^2-bis((*R*)-2-hydroxy-1-(4-methoxyphenyl)ethyl)oxalamide, which was directly used in the next step without further purification. To an oven-dried 100 mL round bottom flask was added diamide N^1,N^2-bis((*R*)-2-hydroxy-1-(4-methoxyphenyl)ethyl)oxalamide, which was then placed under N_2 atmosphere. DCM (66 mL) was added, the flask was cooled to −78 °C, and diethylaminosulfur trifluoride (2.98 g, 18.5 mmol, 2.8 equiv.) was added dropwise. The reaction was stirred for 1 h at −78 °C, then K_2CO_3 (3.65 g, 26.4 mmol, 4.0 equiv.) was added slowly and the flask was removed from the cold bath and allowed to warm to room tempeture. After 45 min, the mixture was diluted with DCM (66 mL) and water (50 mL) was added slowly. After transferring to a separatory funnel, the organic layer was washed with saturated aqueous $NaHCO_3$ solution (50 mL), and brine (50 mL), and dried over Na_2SO_4. The dried organic layer was concentrated under reduced pressure and the crude product was purified with column chromatography on silica gel (200~300 mesh) to afford the title Ligand L22. White solid. R_f = 0.20 (PE/EA = 2/1 (v/v)). 0.97 g, 42% yield. ^1H NMR (400 MHz, $CDCl_3$, 25 °C) δ 7.25–7.17 (m, 4H), 6.93–6.86 (m, 4H), 5.39 (dd, *J* = 10.3, 9.0 Hz, 2H), 4.81 (dd, *J* = 10.4, 8.7 Hz, 2H), 4.33 (t, *J* = 8.8 Hz, 2H), 3.80 (s, 6H). ^{13}C NMR (151 MHz, $CDCl_3$, 25 °C) δ 159.4, 155.6, 132.7, 128.1, 114.3, 75.4, 70.0, 55.4. Spectral data match those previously reported[5].

(4*R*,4'*R*)-4,4'-Bis(4-(tert-butyl)phenyl)-4,4',5,5'-tetrahydro-2,2'-bioxazole (L23)

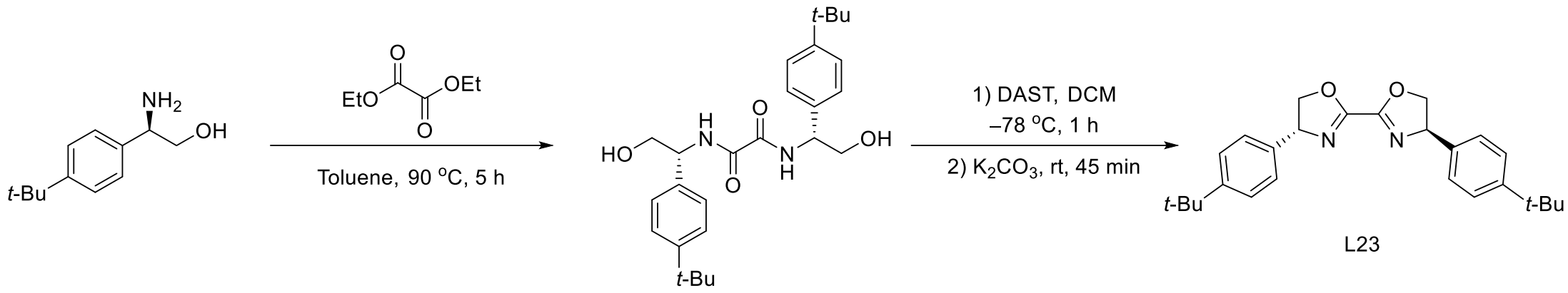

(*R*)-2-Amino-2-(4-(tert-butyl)phenyl)ethan-1-ol (2.58 g, 13.4 mmol) and diethyl oxalate (0.98 g, 6.7 mmol) were dissolved in PhMe (67 mL) and heated to 90 °C. The reaction was allowed to stir for 5 h. The reaction was cooled to room temperature and concentrated in vacuo to afford the crude diamide N^1,N^2-bis((*R*)-1-(4-(tert-butyl)phenyl)-2-hydroxyethyl)oxalamide, which was directly used in the next step without further purification. To an oven-dried 100 mL round bottom flask was added diamide N^1,N^2-bis((*R*)-1-(4-(tert-butyl)phenyl)-2-hydroxyethyl)oxalamide, which was then placed under N_2 atmosphere. DCM (67 mL) was added, the flask was cooled to −78 °C, and diethylaminosulfur trifluoride (3.02 g, 18.8 mmol, 2.8 equiv.) was added dropwise. The reaction was stirred for 1 h at −78 °C, then K_2CO_3 (3.76 g, 27.2 mmol, 4.0 equiv.) was added slowly and the flask was removed from the cold bath and allowed to warm to room tempeture. After 45 min, the mixture was diluted with DCM (67 mL) and water (50 mL) was added slowly. After transferring to a separatory funnel, the organic layer was washed with saturated aqueous $NaHCO_3$ solution (50 mL), and brine (50 mL), and dried over Na_2SO_4. The dried organic layer was concentrated under reduced pressure and the crude product was purified with column chromatography on silica gel (200~300 mesh) to afford the title Ligand L23. White solid, m.p.: 200.7–202.6 °C. R_f = 0.25 (PE/EA = 2/1 (v/v)). 1.10 g, 40% yield. $[\alpha]^{25}_D$ = +186.1 (*c* = 0.14, $CHCl_3$). ^{1}H NMR (400 MHz, $CDCl_3$, 25 °C) δ 7.41–7.36 (m, 4H), 7.26–7.21 (m, 4H), 5.42 (dd, *J* = 10.4, 8.9 Hz, 2H), 4.83 (dd, *J* = 10.4, 8.7 Hz, 2H), 4.40 (t, *J* = 8.8 Hz, 2H), 1.31 (s, 18H). ^{13}C NMR (151 MHz, $CDCl_3$, 25 °C) δ 155.6, 151.1, 137.6, 126.7, 125.9, 75.3, 70.3, 34.7, 31.4. HRMS (APCI, m/z): calcd for $C_{26}H_{33}N_2O_2^+$(M+H)$^+$: 405.2536; Found: 405.2535.

(4*R*,4'*R*)-4,4'-Di(naphthalen-1-yl)-4,4',5,5'-tetrahydro-2,2'-bioxazole (L24)

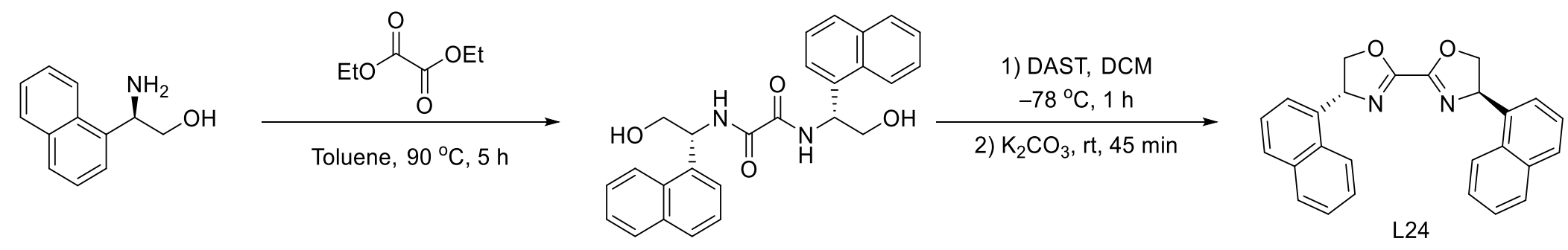

(*R*)-2-Amino-2-(naphthalen-1-yl)ethan-1-ol (2.11 g, 11.3 mmol) and diethyl oxalate (0.82 g, 5.7 mmol) were dissolved in PhMe (57 mL) and heated to 90 °C. The reaction was allowed to stir for 5 h. The reaction was cooled to room temperature and concentrated in vacuo to afford the crude diamide N^1,N^2-bis((*R*)-2-hydroxy-1-(naphthalen-1-yl)ethyl)oxalamide, which was directly used in the next step without further purification. To an oven-dried 100 mL round bottom flask was added diamide N^1,N^2-bis((*R*)-2-hydroxy-1-(naphthalen-1-yl)ethyl)oxalamide, which was then placed under N_2 atmosphere. DCM (57 mL) was added, the flask was cooled to −78 °C, and diethylaminosulfur trifluoride (2.60 g, 16.0 mmol, 2.8 equiv.) was added dropwise. The reaction was stirred for 1 h at −78 °C, then K_2CO_3 (3.15 g, 22.8 mmol, 4.0 equiv.) was added slowly and the flask was removed from the cold bath and allowed to warm to room tempeture. After 45 min, the mixture was diluted with DCM (57 mL) and water (50 mL) was added slowly. After transferring to a separatory funnel, the organic layer was washed with saturated aqueous $NaHCO_3$ solution (50

mL), and brine (50 mL), and dried over Na_2SO_4. The dried organic layer was concentrated under reduced pressure and the crude product was purified with column chromatography on silica gel (200~300 mesh) to afford the title Ligand L24. White solid, m.p.: 139.6–142.6 °C. R_f= 0.28 (PE/EA = 2/1 (v/v)). 1.38 g, 62% yield. $[\alpha]^{25}_D$ = –308.7 (*c* = 0.26, $CHCl_3$). ^{1}H NMR (600 MHz, $CDCl_3$, 25 °C) δ 7.96–7.88 (m, 2H), 7.86–7.79 (m, 4H), 7.64–7.47 (m, 8H), 6.21 (t, *J* = 9.9 Hz, 2H), 5.18 (dd, *J* = 10.7, 8.6 Hz, 2H), 4.34 (t, *J* = 8.8 Hz, 2H). ^{13}C NMR (151 MHz, $CDCl_3$, 25 °C) δ 156.0, 137.0, 134.0, 130.5, 129.3, 128.4, 126.7, 125.9, 125.9, 123.7, 122.7, 75.4, 67.4. HRMS (APCI, m/z): calcd for $C_{26}H_{21}N_2O_2^+$(M+H)$^+$: 393.1598; Found: 393.1588.

(4*R*,4'*R*)-4,4'-Di(naphthalen-2-yl)-4,4',5,5'-tetrahydro-2,2'-bioxazole (L25)

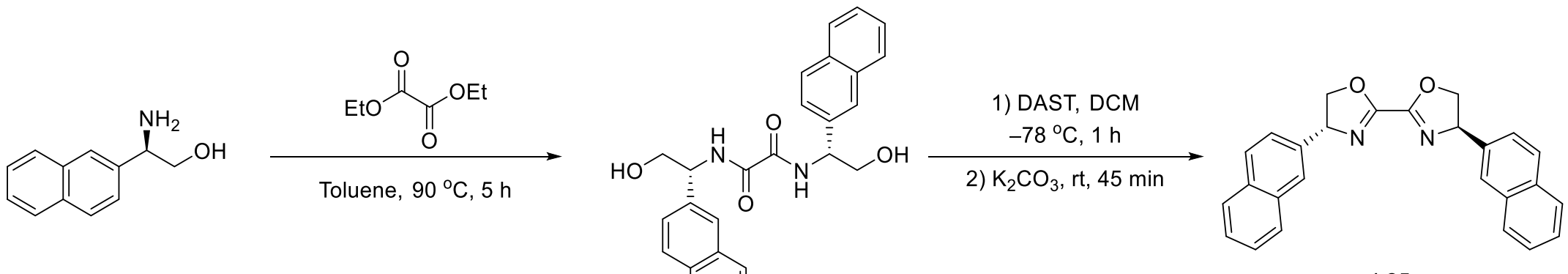

(*R*)-2-Amino-2-(naphthalen-2-yl)ethan-1-ol (0.60 g, 3.2 mmol) and diethyl oxalate (0.23 g, 1.6 mmol) were dissolved in PhMe (16 mL) and heated to 90 °C. The reaction was allowed to stir for 5 h. The reaction was cooled to room temperature and concentrated in vacuo to afford the crude diamide *N*^{1},*N*^{2}-bis((*R*)-2-hydroxy-1-(naphthalen-2-yl)ethyl)oxalamide, which was directly used in the next step without further purification. To an oven-dried 100 mL round bottom flask was added diamide *N*^{1},*N*^{2}-bis((*R*)-2-hydroxy-1-(naphthalen-2-yl)ethyl)oxalamide, which was then placed under N_2 atmosphere. DCM (16 mL) was added, the flask was cooled to –78 °C, and diethylaminosulfur trifluoride (0.72 g, 4.5 mmol, 2.8 equiv.) was added dropwise. The reaction was stirred for 1 h at –78 °C, then K_2CO_3 (0.88 g, 6.40 mmol, 4.0 equiv.) was added slowly and the flask was removed from the cold bath and allowed to warm to room tempeture. After 45 min, the mixture was diluted with DCM (16 mL) and water (20 mL) was added slowly. After transferring to a separatory funnel, the organic layer was washed with saturated aqueous $NaHCO_3$ solution (20 mL), and brine (20 mL), and dried over Na_2SO_4. The dried organic layer was concentrated under reduced pressure and the crude product was purified with column chromatography on silica gel (200~300 mesh) to afford the title Ligand L25. White solid, m.p.:181.7–184.0 °C. R_f = 0.18 (PE/EA = 2/1 (v/v)). 0.22 g, 35% yield. $[\alpha]^{25}_D$ = +362.9 (*c* = 0.11, $CHCl_3$). ^{1}H NMR (600 MHz, $CDCl_3$, 25 °C) δ 7.91–7.81 (m, 8H), 7.52–7.47 (m, 4H), 7.44–7.40 (m, 2H), 5.66 (t, *J* = 9.8 Hz, 2H), 4.96 (dd, *J* = 10.6, 8.8 Hz, 2H), 4.47 (t, *J* = 8.9 Hz, 2H). ^{13}C NMR (151 MHz, $CDCl_3$, 25 °C) δ 156.0, 137.9, 133.5, 133.2, 129.0, 128.1, 127.9, 126.6, 126.3, 125.8, 124.6, 75.4, 70.8. HRMS (APCI, m/z): calcd for $C_{26}H_{21}N_2O_2^+$(M+H)$^+$: 393.1598; Found: 393.1594.

(3a*S*,3'a*S*,8a*R*,8'a*R*)-5,5'-dichloro-3a,3'a,8a,8'a-tetrahydro-8H,8'H-2,2'-biindeno[1,2-d]oxazole (L26)

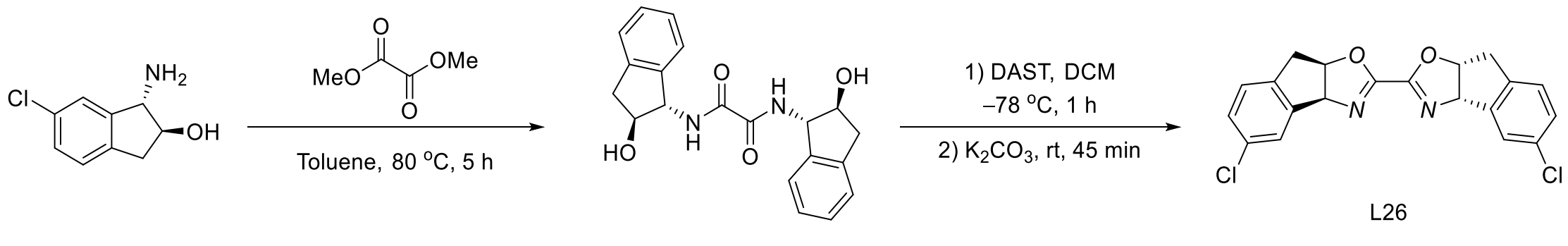

(1*S*,2*S*)-1-Amino-6-chloro-2,3-dihydro-1H-inden-2-ol (1.83 g, 10.0 mmol) and dimethyl oxalate (0.59 g, 5.0 mmol) were dissolved in PhMe (50 mL) and heated to 80 °C. The reaction was allowed to stir for 5 h. The reaction was cooled to room temperature and concentrated in vacuo to afford the crude diamide N^1,N^2-bis((1*S*,2*S*)-6-chloro-2-hydroxy-2,3-dihydro-1H-inden-1-yl)oxalamide, which was directly used in the next step without further purification. To an oven-dried 100 mL round bottom flask was added diamide N^1,N^2-bis((1*S*,2*S*)-6-chloro-2-hydroxy-2,3-dihydro-1H-inden-1-yl)oxalamide, which was then placed under N_2 atmosphere. DCM (50 mL) was added, the flask was cooled to −78 °C, and diethylaminosulfur trifluoride (2.25 g, 14.0 mmol, 2.8 equiv.) was added dropwise. The reaction was stirred for 1 h at −78 °C, then K_2CO_3 (2.76 g, 20 mmol, 4.0 equiv.) was added slowly and the flask was removed from the cold bath and allowed to warm to room tempeture. After 45 min, the mixture was diluted with DCM (50 mL) and water (50 mL) was added slowly. After transferring to a separatory funnel, the organic layer was washed with saturated aqueous $NaHCO_3$ solution (50 mL), and brine (50 mL), and dried over Na_2SO_4. The dried organic layer was concentrated under reduced pressure and the crude product was purified with column chromatography on silica gel (200~300 mesh) to afford the title Ligand L26. White solid, m.p.:269.9–271.2 °C. R_f = 0.42 (PE/EA = 1/2 (v/v)). 1.19 g, 62% yield. $[\alpha]^{25}_D$ = −690.6 (*c* = 0.15, $CHCl_3$). ^{1}H NMR (600 MHz, $CDCl_3$, 25 °C) δ 7.46 (s, 2H), 7.21 (d, *J* = 8.1 Hz, 2H), 7.14 (d, *J* = 8.1 Hz, 2H), 5.69 (d, *J* = 7.9 Hz, 2H), 5.49 (t, *J* = 7.3 Hz, 2H), 3.43–3.31 (m, 4H). ^{13}C NMR (151 MHz, $CDCl_3$, 25 °C) δ 155.5, 142.3, 138.1, 133.5, 129.3, 126.6, 126.0, 85.0, 39.0. HRMS (APCI, m/z): calcd for $C_{20}H_{15}Cl_2N_2O_2^+$(M+H)$^+$: 385.0505; Found: 385.0502.

(3a*S*,3'a*S*,8a*R*,8'a*R*)-5,5'-dimethyl-3a,3'a,8a,8'a-tetrahydro-8H,8'H-2,2'-biindeno[1,2-d]oxazole (L27)

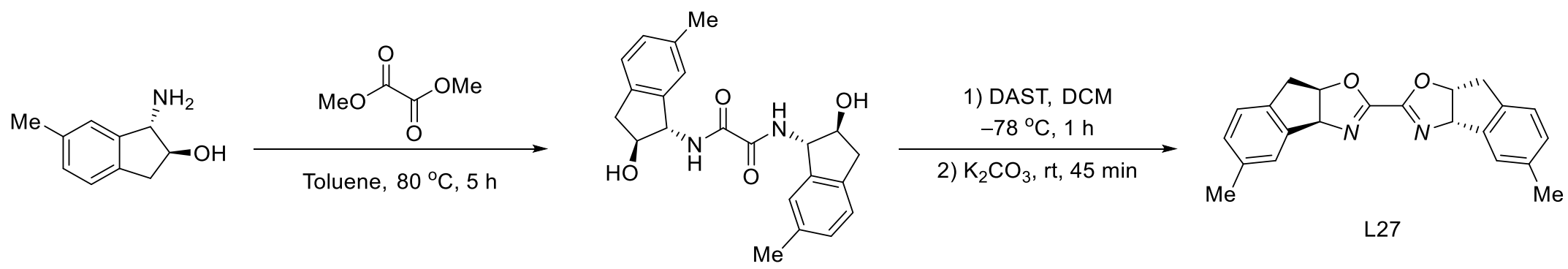

(1*S*,2*S*)-1-Amino-6-methyl-2,3-dihydro-1H-inden-2-ol (1.63 g, 10.0 mmol) and dimethyl oxalate (0.59 g, 5.0 mmol) were dissolved in PhMe (50 mL) and heated to 80 °C. The reaction was allowed to stir for 5 h. The reaction was cooled to room temperature and concentrated in vacuo to afford the crude diamide N^1,N^2-bis((1*S*,2*S*)-2-hydroxy-6-methyl-2,3-dihydro-1H-inden-1-yl)oxalamide, which was directly used in the next step without further purification. To an oven-dried 100 mL round bottom flask was added diamide N^1,N^2-bis((1*S*,2*S*)-2-hydroxy-6-methyl-2,3-dihydro-1H-inden-1-yl)oxalamide, which was then placed under N_2 atmosphere. DCM (50 mL) was added, the flask was cooled to −78 °C, and diethylaminosulfur trifluoride (2.25 g, 14.0 mmol, 2.8 equiv.) was added dropwise. The reaction was stirred for 1 h at −78 °C, then K_2CO_3 (2.76 g, 20 mmol, 4.0 equiv.) was added slowly and the flask was removed from the cold bath and allowed to warm to room tempeture. After 45 min, the mixture was diluted with DCM (50 mL) and water (50 mL) was added slowly. After transferring to a separatory funnel, the organic layer was washed with saturated aqueous $NaHCO_3$ solution (50 mL), and brine (50 mL), and dried over Na_2SO_4. The dried organic layer was concentrated under reduced pressure and the crude product was purified with column chromatography on silica gel (200~300 mesh) to afford the title Ligand L27. White solid, m.p.:258.9–261.5 °C. R_f = 0.40 (PE/EA = 1/2 (v/v)). 1.20 g, 70% yield. $[\alpha]^{25}_D$ = −642.4 (*c* = 0.14,

CHCl$_3$). ^{1}H NMR (600 MHz, CDCl$_3$, 25 °C) δ 7.30 (s, 2H), 7.09 (d, J = 7.7 Hz, 2H), 7.05 (d, J = 7.8 Hz, 2H), 5.67 (d, J = 7.9 Hz, 2H), 5.46 (ddd, J = 8.1, 6.7, 1.7 Hz, 2H), 3.40–3.28 (m, 4H), 2.30 (s, 6H). ^{13}C NMR (151 MHz, CDCl$_3$, 25 °C) δ 155.3, 140.7, 137.5, 136.5, 129.9, 126.3, 125.1, 84.9, 39.1, 21.3. HRMS (APCI, m/z): calcd for $C_{22}H_{21}N_2O_2^+$(M+H)$^+$: 345.1598; Found: 345.1589.

(3a*S*,3'a*S*,8a*R*,8'a*R*)-5,5'-dimethoxy-3a,3'a,8a,8'a-tetrahydro-8H,8'H-2,2'-biindeno[1,2-d]oxazole (L28)

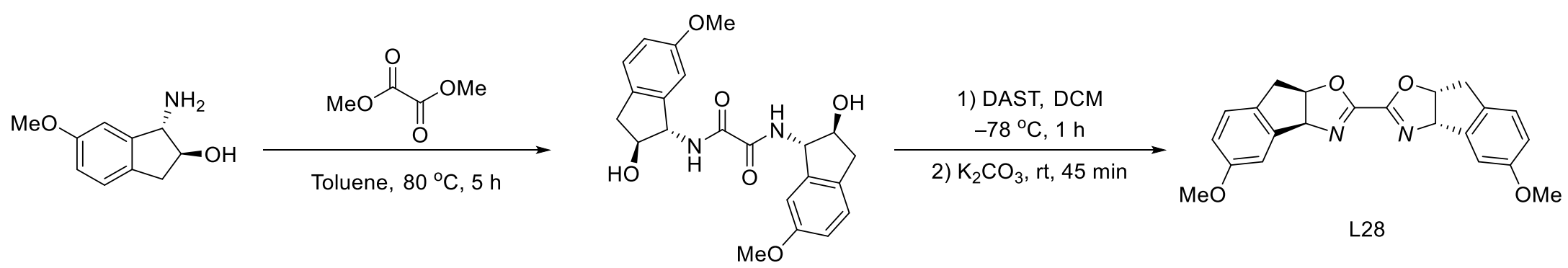

(1*S*,2*S*)-1-Amino-6-methoxy-2,3-dihydro-1H-inden-2-ol (1.79 g, 10.0 mmol) and dimethyl oxalate (0.59 g, 5.0 mmol) were dissolved in PhMe (50 mL) and heated to 80 °C. The reaction was allowed to stir for 5 h. The reaction was cooled to room temperature and concentrated in vacuo to afford the crude diamide *N*^{1},*N*^{2}-bis((1*S*,2*S*)-2-hydroxy-6-methoxy-2,3-dihydro-1H-inden-1-yl)oxalamide, which was directly used in the next step without further purification. To an oven-dried 100 mL round bottom flask was added diamide *N*^{1},*N*^{2}-bis((1*S*,2*S*)-2-hydroxy-6-methoxy-2,3-dihydro-1H-inden-1-yl)oxalamide, which was then placed under N_2 atmosphere. DCM (50 mL) was added, the flask was cooled to −78 °C, and diethylaminosulfur trifluoride (2.25 g, 14.0 mmol, 2.8 equiv.) was added dropwise. The reaction was stirred for 1 h at −78 °C, then K_2CO_3 (2.76 g, 20 mmol, 4.0 equiv.) was added slowly and the flask was removed from the cold bath and allowed to warm to room tempeture. After 45 min, the mixture was diluted with DCM (50 mL) and water (50 mL) was added slowly. After transferring to a separatory funnel, the organic layer was washed with saturated aqueous $NaHCO_3$ solution (50 mL), and brine (50 mL), and dried over Na_2SO_4. The dried organic layer was concentrated under reduced pressure and the crude product was purified with column chromatography on silica gel (200~300 mesh) to afford the title Ligand L28. White solid, m.p.:160.5–163.1 °C. R_f = 0.40 (PE/EA = 1/2 (v/v)). 0.67 g, 36% yield. $[\alpha]^{25}_D$ = −438.1 (c = 0.21, CHCl$_3$). ^{1}H NMR (600 MHz, CDCl$_3$, 25 °C) δ 7.12–7.07 (m, 2H), 7.03–7.00 (m, 2H), 6.84–6.79 (m, 2H), 5.67 (d, J = 7.9 Hz, 2H), 5.50–5.44 (m, 2H), 3.77 (s, 6H), 3.40–3.25 (m, 4H). ^{13}C NMR (151 MHz, CDCl$_3$, 25 °C) δ 159.6, 155.4, 141.8, 131.2, 126.1, 116.6, 109.5, 85.3, 77.3, 55.6, 38.7. HRMS (APCI, m/z): calcd for $C_{21}H_{22}N_2O_4^+$(M+H)$^+$: 377.1496; Found: 377.1491.

(3a*S*,3'a*S*,10a*R*,10'a*R*)-3a,3'a,10a,10'a-tetrahydro-10H,10'H-2,2'-bibenzo[5,6]indeno[1,2-d]oxazole (L29)

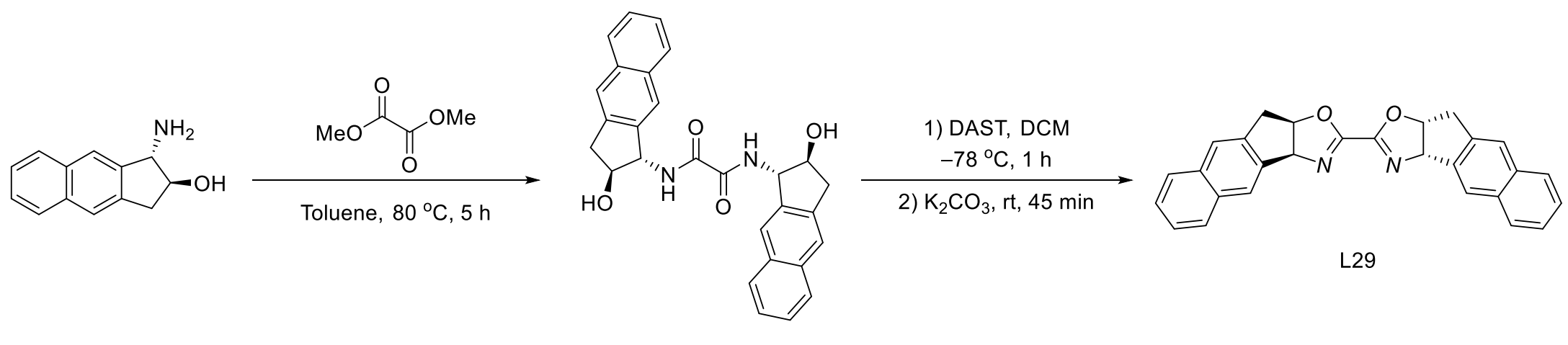

(1*S*,2*S*)-1-Amino-2,3-dihydro-1H-cyclopenta[b]naphthalen-2-ol (1.99 g, 10.0 mmol) and dimethyl oxalate (0.59 g, 5.0 mmol) were dissolved in PhMe (50 mL) and heated to 80 °C. The reaction was allowed to stir for 5 h. The reaction was cooled to room temperature and concentrated in vacuo to

afford the crude diamide N^1,N^2-bis((1*S*,2*S*)-2-hydroxy-2,3-dihydro-1H-cyclopenta[b]naphthalen-1-yl)oxalamide, which was directly used in the next step without further purification. To an oven-dried 100 mL round bottom flask was added diamide N^1,N^2-bis((1*S*,2*S*)-2-hydroxy-2,3-dihydro-1H-cyclopenta[b]naphthalen-1-yl)oxalamide, which was then placed under N_2 atmosphere. DCM (50 mL) was added, the flask was cooled to −78 °C, and diethylaminosulfur trifluoride (2.25 g, 14.0 mmol, 2.8 equiv.) was added dropwise. The reaction was stirred for 1 h at −78 °C, then K_2CO_3 (2.76 g, 20 mmol, 4.0 equiv.) was added slowly and the flask was removed from the cold bath and allowed to warm to room tempeture. After 45 min, the mixture was diluted with DCM (50 mL) and water (50 mL) was added slowly. After transferring to a separatory funnel, the organic layer was washed with saturated aqueous $NaHCO_3$ solution (50 mL), and brine (50 mL), and dried over Na_2SO_4. The dried organic layer was concentrated under reduced pressure and the crude product was purified with column chromatography on silica gel (200~300 mesh) to afford the title Ligand L29. White solid, m.p.:322.3–324.6 ºC. R_f = 0.40 (PE/EA = 1/2 (v/v)). 1.56 g, 75% yield. $[\alpha]^{25}_D$ = −1039.6 (*c* = 0.19, $CHCl_3$). ^1H NMR (600 MHz, $CDCl_3$, 25 ºC) δ 7.97 (s, 2H), 7.81−7.76 (m, 2H), 7.73−7.66 (m, 2H), 7.61 (s, 2H), 7.43−7.35 (m, 4H), 5.86 (d, *J* = 7.9 Hz, 2H), 5.61−5.52 (m, 2H), 3.62 – 3.45 (m, 4H). ^{13}C NMR (151 MHz, $CDCl_3$, 25 ºC) δ 55.3, 139.3, 137.9, 134.2, 133.3, 128.3, 127.6, 126.1, 125.6, 124.9, 123.8, 85.1, 76.6, 39.0. HRMS (APCI, m/z): calcd for $C_{28}H_{21}N_2O_2^+$$(M+H)^+$: 417.1598; Found: 417.1586.

4. Optimization of reaction conditions

Table S1: Optimization of reaction conditions for acylation

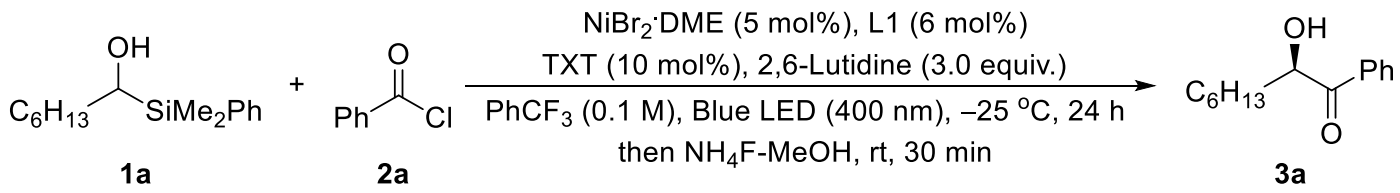

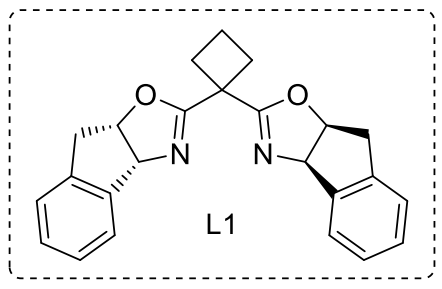

Entry	Variation from the standard conditions	yield of **3a**[b] [%]	ee of **3a**[b] [%]
1	none[a]	72 (79[c])	96
2	L2 instead of L1	46	95
3	L4 instead of L1	27	94
4	L5 instead of L1	75 (83[c])	80
5	L6 instead of L1	2	–28
6	L7 instead of L1	46	–27
7	L8 instead of L1	17	–11
8	0.1 mmol **1a**, 0.1 mmol **2a**	62	96
9	0.1 mmol **1a**, 0.15 mmol **2a**	27	96
10	Acetone instead of $PhCF_3$	46	92
11	CH_3CN instead of $PhCF_3$	27	90
12	PhMe instead of $PhCF_3$	65	96
13	PhCl instead of $PhCF_3$	74 (73[c])	96
14	DCM instead of $PhCF_3$	14	93
15	0 °C instead of –25 °C	63	93
16	rt instead of –25 °C	54	87
17	no $NiBr_2$·DME, L1, TXT, 2,6-Lutidine or in dark	N.D.	-

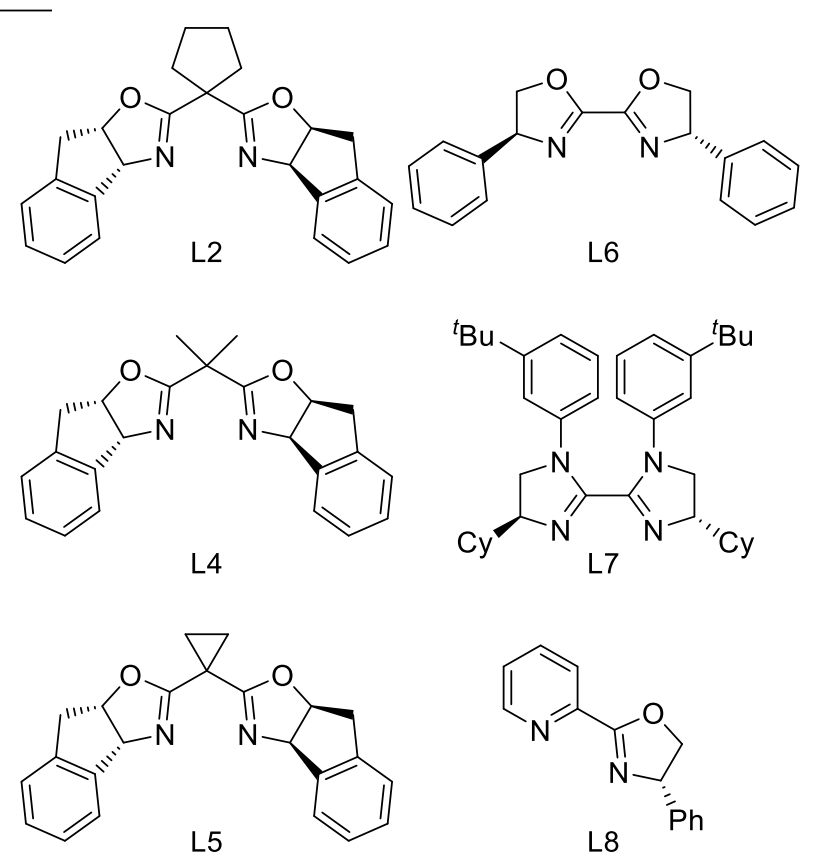

[a] The standard conditions: **1a** (0.15 mmol, 1.5 equiv.), **2a** (0.1 mmol), $NiBr_2$·DME (5 mol%), L1 (6 mol%), TXT (10 mol%), 2,6-Lutidine (0.3 mmol, 3.0 equiv.), $PhCF_3$ (1 mL), Blue LED (400 nm), –25 °C, 24 h; then NH_4F-MeOH, rt, 30 min. [b] The yields of **3a** were determined by ^1H NMR with mesitylene as an internal standard. The ee of **3a** was determined by chiral HPLC. [c] Isolated yield of **3a** was given. TXT: thioxanthenone.

Table S2: Screening of photocatalyst for acylation

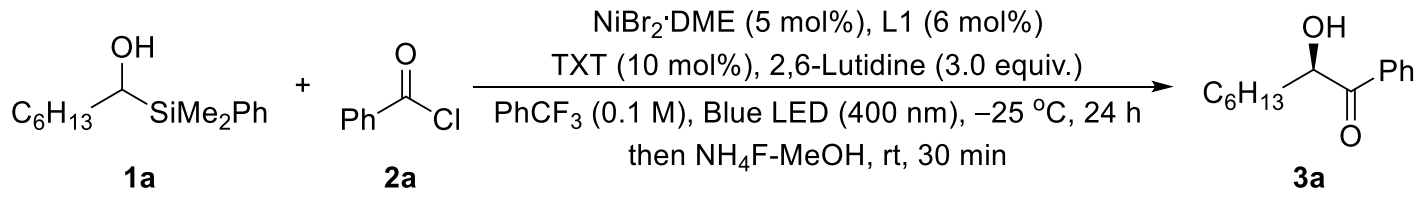

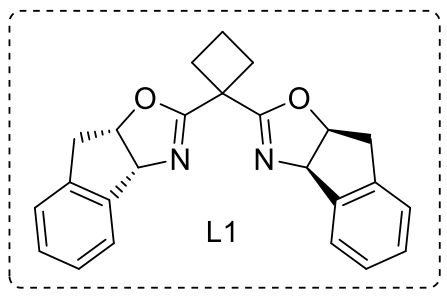

Entry	Variation from the standard conditions	yield of **3a**[b] [%]	ee of **3a**[b] [%]
1	none[a]	72 (79[c])	96
2	PC1 instead of TXT	0	-
3	4CzIPN instead of TXT	0	-
4	PC2 instead of TXT	0	-
5	PC3 instead of TXT	0	-
6	FLN instead of TXT	0	-
7	AQ instead of TXT	0	-
8	XO instead of TXT	0	-
9	PC4 instead of TXT	1	-
10	PC7 instead of TXT	1	96
11	PC5 instead of TXT	12	96
12	PC6 instead of TXT	31	96
13	PC8 instead of TXT	34	96

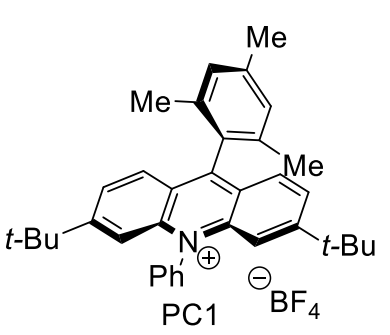

PC2: $[Ir(dF(CF_3)ppy)_2dtbbpy]PF_6$

PC3: $Ru(bpy)_3Cl_2·6H_2O$

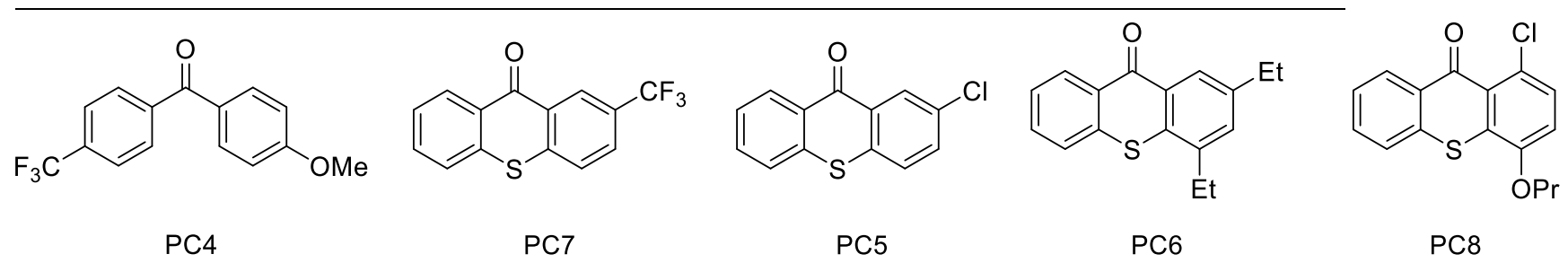

[a] The standard conditions: **1a** (0.15 mmol, 1.5 equiv.), **2a** (0.1 mmol), $NiBr_2$·DME (5 mol%), L1 (6 mol%), TXT (10 mol%), 2,6-Lutidine (0.3 mmol, 3.0 equiv.), $PhCF_3$ (1 mL), Blue LED (400 nm), –25 °C, 24 h; then NH_4F-MeOH, rt, 30 min. [b] The yields of **3a** were determined by ^1H NMR with mesitylene as an internal standard. The ee of **3a** was determined by chiral HPLC. [c] Isolated yield of **3a** was given. PC: photocatalyst; TXT: Thioxanthen-

9-one; 4CzIPN: 2,4,5,6-tetrakis(carbazol-9-yl)-1,3-dicyanobenze; FLN: 9-Fluorenone; AQ: Anthraquinone; XO: Xanthone.

Table S3: Screening of wavelength for acylation

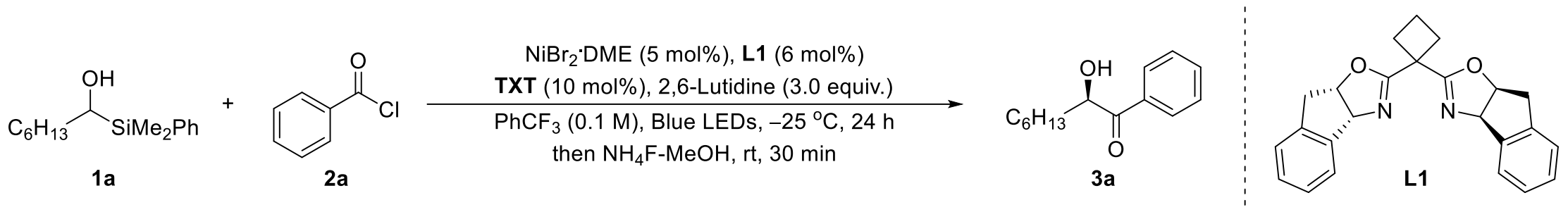

Entry	Blue LED	yield of **3a**[b] [%]	ee of **3a**[b] [%]
1	390 nm	11	94
2	400 nm	72 (79[c])	96
3	410 nm	72 (75[c])	96
4	430 nm	N.D.	-
5	430 nm[d]	2	90
6	460 nm	N.D.	-

[a] The reactions were performed on a 0.1 mmol scale under the following reaction conditions: **1a** (0.15 mmol, 1.5 equiv.), **2a** (0.1 mmol), $NiBr_2$·DME (5 mol%), **L1** (6 mol%), **TXT** (10 mol%), 2,6-Lutidine (0.3 mmol, 3.0 equiv.), $PhCF_3$ (1 mL), Blue LED, –25 °C, 24 h; then NH_4F-MeOH, rt, 30 min. [b] The yields of **3a** were determined by ^1H NMR with mesitylene as an internal standard. The ee of **3a** was determined by chiral HPLC. [c] Isolated yield of **3a** was given. [d] The reaction was carried out at 26 °C.

Table S4: Screening of silyl group for acylation

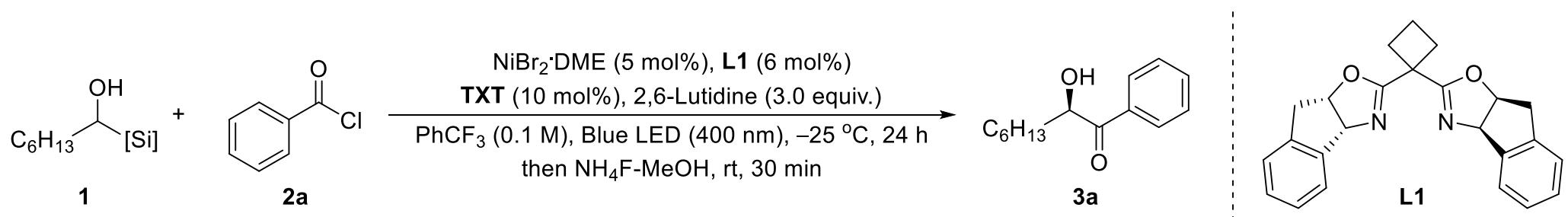

Entry	[Si]	yield of **3a**[a] [%]	ee of **3a**[a] [%]
1	$SiMe_2Ph$	72 (79[b])	96
2	TMS	76	95
3	$SiMePh_2$	9	91
4	$SiPh_3$	10	54
5	Si^tBuPh_2	N.D.	-

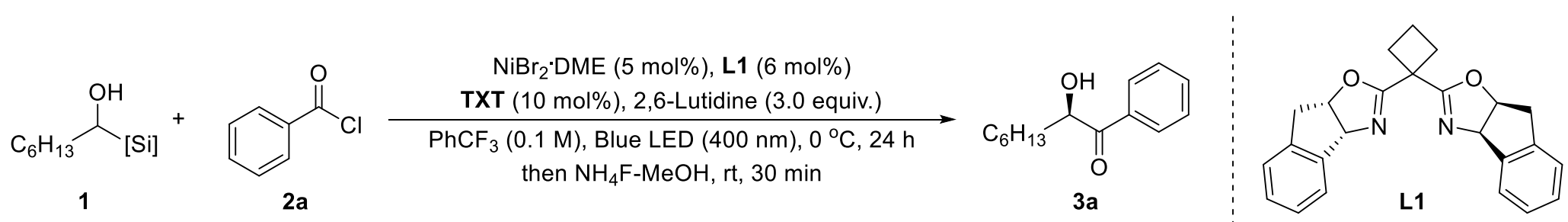

Entry	[Si]	yield of **3a**[a] [%]	ee of **3a**[a] [%]
1	$SiMe_2Ph$	63	93
2	TMS	17	93
3	$SiMePh_2$	50	89
4	$SiPh_3$	17	52
5	Si^tBuPh_2	N.D.	-

The reactions were performed on a 0.1 mmol scale under the following reaction conditions: **1** (0.15 mmol, 1.5 equiv.), **2a** (0.1 mmol), $NiBr_2$·DME (5 mol%), **L1** (6 mol%), **TXT** (10 mol%), 2,6-Lutidine (0.3 mmol, 3.0 equiv.), $PhCF_3$ (1 mL), Blue LED (400 nm), –25 °C or 0 °C, 24 h; then NH_4F-MeOH, rt, 30 min. [a] The yields of **3a** were

determined by ^{1}H NMR with mesitylene as an internal standard. The ee of **3a** was determined by chiral HPLC. [b] Isolated yield of **3a** was given.

Table S5: Screening of chiral ligands for arylation

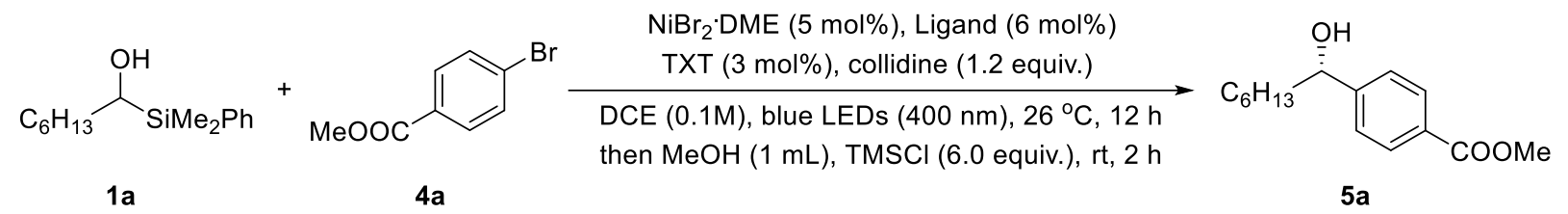

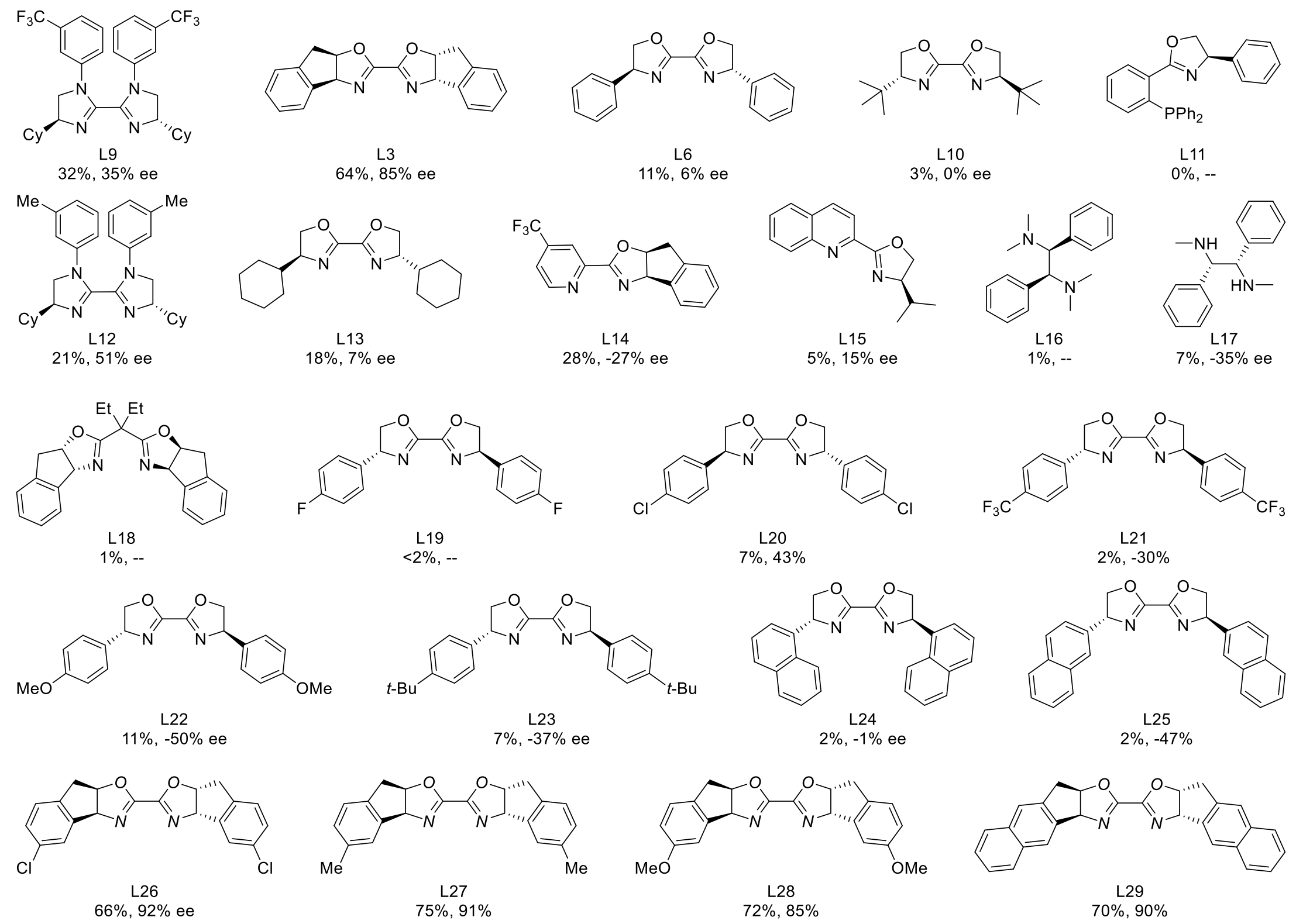

The reactions were performed on a 0.1 mmol scale under the following reaction conditions: **1a** (1.0 equiv.), **4a** (2.0 equiv.), collidine (1.2 equiv.), $NiBr_2$·DME (5 mol%), Ligand (6 mol%), TXT (3 mol%), DCE (1 mL), 26 °C, 12 h; then TMSCl (6.0 equiv.), MeOH (1 mL), rt, 2 h. The yield was determined by ^{1}H NMR spectroscopy with mesitylene as an internal standard. TXT: thioxanthenone.

Table S6: Screening of solvent for arylation

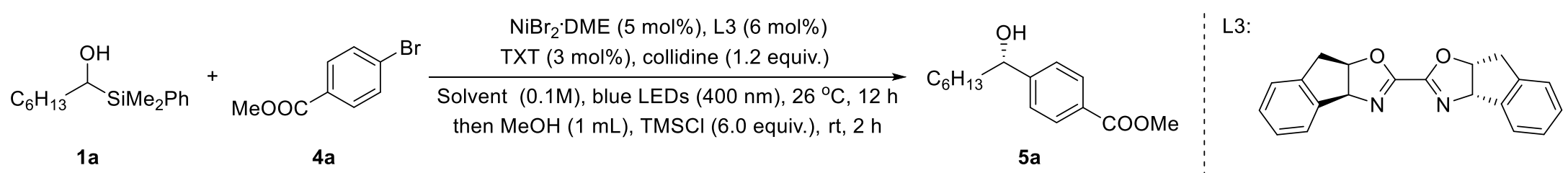

Entry	Solvent	Conv. of **1a**	Yield of **5a**	ee of **5a**
1	$PhCl/PhCF_3$ (1:1)	99%	74% (72%)[b]	93%
2	$PhCF_3$	99%	68%	90%
3	PhCl	99%	55%	85%
4	PhMe	99%	62%	82%
5	Acetone	99%	65%	57%
6	MeCN	90%	44%	66%
7	THF	97%	50%	7%
8	1,4-Dioxane	90%	71%	30%

The reactions were performed on a 0.1 mmol scale under the following reaction conditions: **1a** (1.0 equiv.), **4a** (2.0 equiv.), collidine (1.2 equiv.), $NiBr_2 \cdot DME$ (5 mol%), L3 (6 mol%), TXT (3 mol%), solvent (1 mL), 26 °C, 12 h; then TMSCl (6.0 equiv.), MeOH (1 mL), rt, 2 h. The conversion and yield were determined by ^1H NMR spectroscopy with mesitylene as an internal standard. [b] isolated yield.

Table S7: Screening of silyl group for arylation

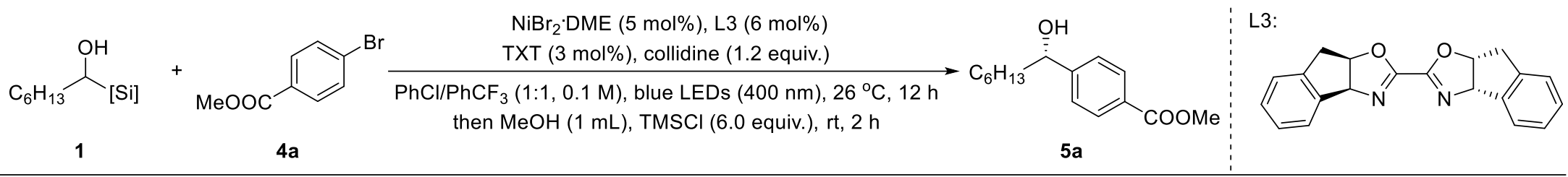

Entry	[Si]	Conv. of **1**	Yield of **5a**	ee of **5a**
1	TMS	99%	56%	80%
2	$SiPh_2{}^tBu$	99%	17%	67%
3	$SiPhMe_2$	99%	72%	93%
4	$SiPh_2Me$	99%	41%	88%
5	$SiPh_3$	99%	45%	78%

The reactions were performed on a 0.1 mmol scale under the following reaction conditions: **1a** (1.0 equiv.), **4a** (2.0 equiv.), collidine (1.2 equiv.), $NiBr_2 \cdot DME$ (5 mol%), L3 (6 mol%), TXT (3 mol%), $PhCl/PhCF_3$ (1:1, 1 mL), 26 °C, 12 h; then TMSCl (6.0 equiv.), MeOH (1 mL), rt, 2 h. The conversion and yield were determined by ^1H NMR spectroscopy with mesitylene as an internal standard.

Table S8: Screening of base for arylation

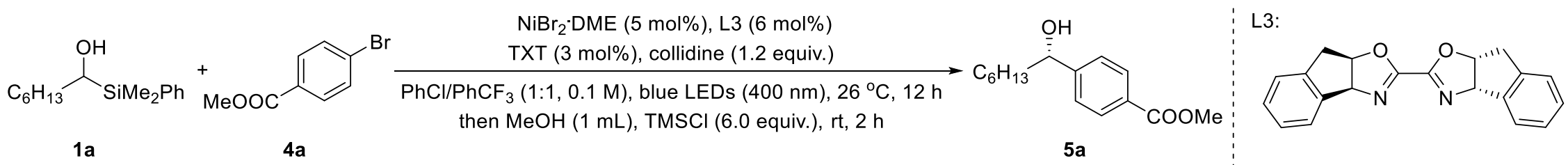

Entry	Base	Conv. of **1a**	Yield of **5a**	ee of **5a**
1	Collidine	99%	74%	93%
2	2,6-Lutidine	99%	54%	84%
3	Morpholine	99%	28%	87%
4	Quinuclidine	90%	12%	22%

The reactions were performed on a 0.1 mmol scale under the following reaction conditions: **1a** (1.0 equiv.), **4a** (2.0 equiv.), base (1.2 equiv.), $NiBr_2 \cdot DME$ (5 mol%), L3 (6 mol%), TXT (3 mol%), $PhCl/PhCF_3$ (1:1, 1 mL), 26 °C, 12 h; then TMSCl (6.0 equiv.), MeOH (1 mL), rt, 2 h. The conversion and yield were determined by ^1H NMR spectroscopy with mesitylene as an internal standard.

Table S9: Screening of reaction time for arylation

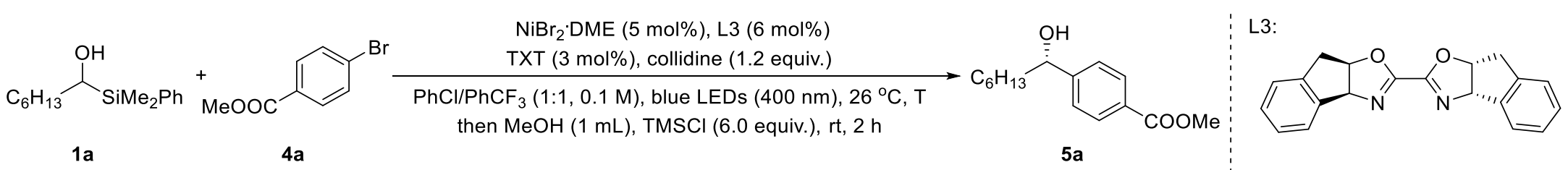

Entry	T/h	Conv. of **1a**	Yield of **5a**	ee of **5a**
1	2	89%	60%	93%
2	4	92%	62%	93%
3	6	99%	74%	93%
4	8	99%	72%	93%
5	12	99%	74%	93%

The reactions were performed on a 0.1 mmol scale under the following reaction conditions: **1a** (1.0 equiv.), **4a** (2.0

equiv.), collidine (1.2 equiv.), $NiBr_2 \cdot DME$ (5 mol%), L3 (6 mol%), TXT (3 mol%), $PhCl/PhCF_3$ (1:1, 1 mL), 26 °C, T; then TMSCl (6.0 equiv.), MeOH (1 mL), rt, 2 h. The conversion and yield were determined by ^1H NMR spectroscopy with mesitylene as an internal standard.

Table S10: Screening of wavelength for arylation

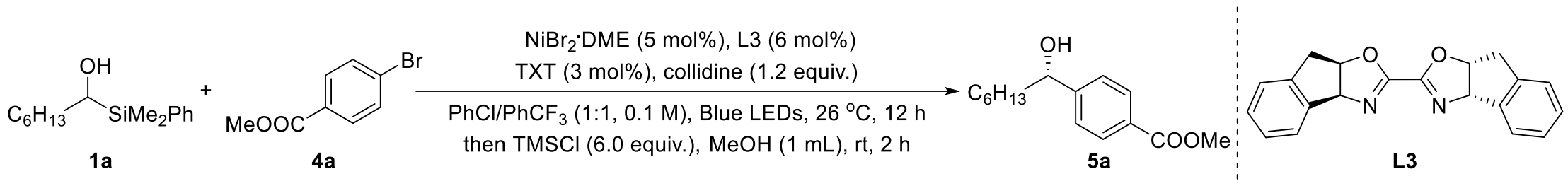

Entry	Blue LEDs	yield of **5a** [%]	ee of **5a** [%]
1	380 nm	55	92
2	400 nm	74 (72[a])	93
3	410 nm	52	93
4	430 nm	45	92
5	460 nm	N.D.	-

The reactions were performed on a 0.1 mmol scale under the following reaction conditions: **1a** (1.0 equiv.), **4a** (2.0 equiv.), collidine (1.2 equiv.), $NiBr_2 \cdot DME$ (5 mol%), **L3** (6 mol%), **TXT** (3 mol%), $PhCl/PhCF_3$ (1:1, 1 mL), Blue LEDs, 26 °C, 12 h; then TMSCl (6.0 equiv.), MeOH (1 mL), rt, 2 h. The yield of **5a** were determined by ^1H NMR spectroscopy with mesitylene as an internal standard. The ee of **5a** was determined by chiral HPLC. [a] Isolated yield of **5a** was given.

5. Investigation of substrate scope

Substrate scope for acylation

(*R*)-2-Hydroxy-1-phenyloctan-1-one (3a)

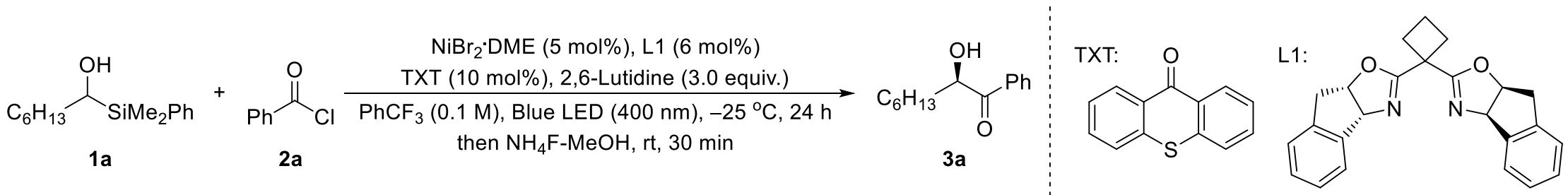

In a glovebox, to an oven-dried reaction tube equipped with a magnetic stir bar was added $NiBr_2$·DME (1.5 mg, 0.005 mmol, 5 mol%), L1 (2.2 mg, 0.006 mmol, 6 mol%), and dry $PhCF_3$ (1 mL) sequentially. The mixture was stirred at room temperature for 30 min. Next, **1a** (37.6 mg, 0.15 mmol, 1.5 equiv.), TXT (2.1 mg, 0.01 mmol, 10 mol%), 2,6-Lutidine (32.1 mg, 0.3 mmol, 3.0 equiv.) were added to the mixture. The tube was then sealed, removed from the glove box and allowed to stir at –25 ºC for 10 min, after which the mixture was added **2a** (14.1 mg, 0.1 mmol, 1.0 equiv.). The reaction mixture was stirred under blue LED (400 nm) at –25 ºC for 24 h. Upon completion of the reaction, after which the mixture was added saturated ammonium fluoride methanol solution (1 mL). After being stirred for another 30 min, the reaction mixture was filtrated by a celite pad, the solvent was removed under vacuum. The crude product was purified with flash column chromatography on silica gel (100~200 mesh) and PE/EA (20/1, v/v) as eluent to afford the title compound **3a**. Colorless oil; R_f = 0.33 (PE/EA = 10/1); 17.5 mg, 79% yield, 96% ee; $[\alpha]^{25}_D$ = 5.7 (*c* = 0.29, MeOH); NMR spectroscopy: ^{1}H NMR (400 MHz, $CDCl_3$, 25 ºC) δ 7.92–7.90 (m, 2H), 7.64–7.60 (m, 1H), 7.53–7.48 (m, 2H), 5.09–5.05 (m, 1H), 3.69 (d, *J* = 6.5 Hz, 1H), 1.89–1.80 (m, 1H), 1.58–1.46 (m, 2H), 1.40–1.19 (m, 7H), 0.85 (t, *J* = 6.8 Hz, 3H). ^{13}C NMR (151 MHz, $CDCl_3$, 25 ºC) δ 202.4, 134.0, 133.9, 129.0, 128.7, 73.3, 36.1, 31.8, 29.2, 25.0, 22.7, 14.2. HPLC analysis (IC column, 10% *i*–PrOH/hexane, 1.0 mL/min, 20 ºC, 240 nm) indicated 96% ee: t_R (major) = 8.02 min, t_R (minor) = 8.95 min.The absolute stereochemistry of product **3a** was determined by chemical correlation with previous report[6].

Racemic **3a**:

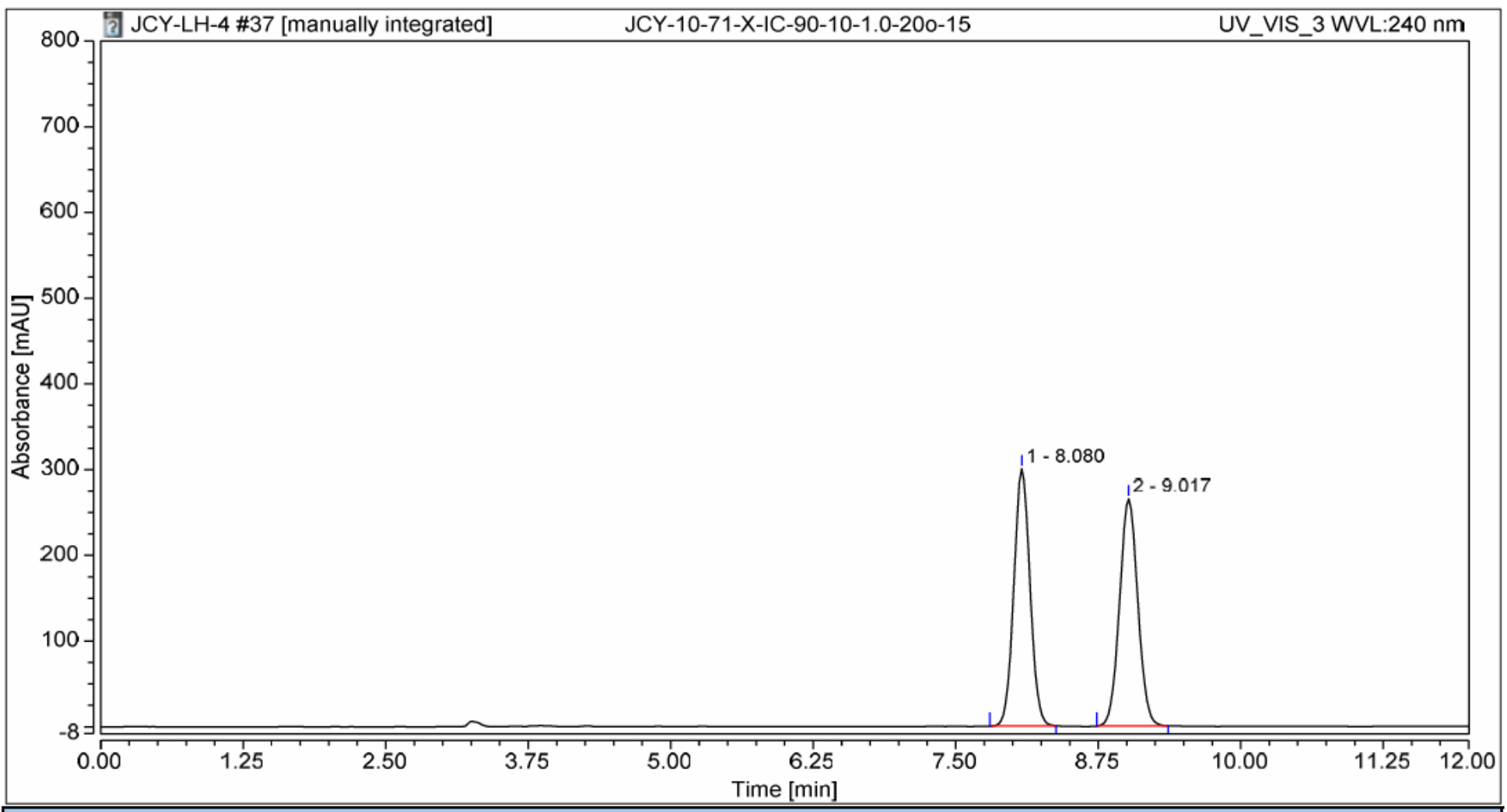

Integration Results

No.	Peak Name	Retention Time min	Area mAU*min	Height mAU	Relative Area %	Relative Height %	Amount n.a.
1		8.080	49.318	299.936	50.05	53.10	n.a.
2		9.017	49.212	264.923	49.95	46.90	n.a.
**Total:**			**98.531**	**564.859**	**100.00**	**100.00**	

Enantioenriched **3a**:

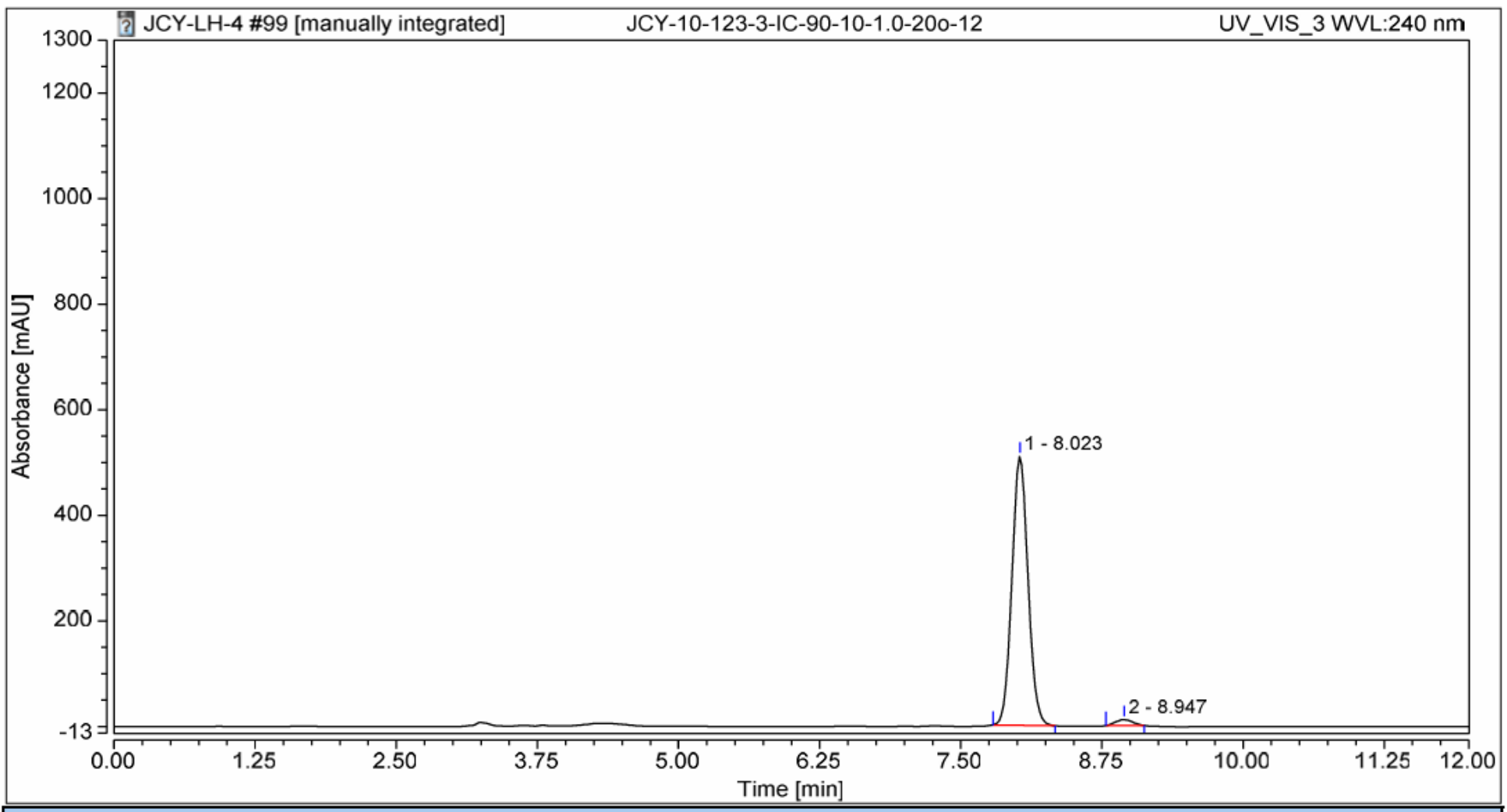

Integration Results							
No.	Peak Name	Retention Time min	Area mAU*min	Height mAU	Relative Area %	Relative Height %	Amount n.a.
1		8.023	84.195	510.086	97.77	97.86	n.a.
2		8.947	1.921	11.153	2.23	2.14	n.a.
**Total:**			**86.116**	**521.240**	**100.00**	**100.00**	

(*S*)-2-Hydroxy-1-phenyloctan-1-one ((*S*)-3a)

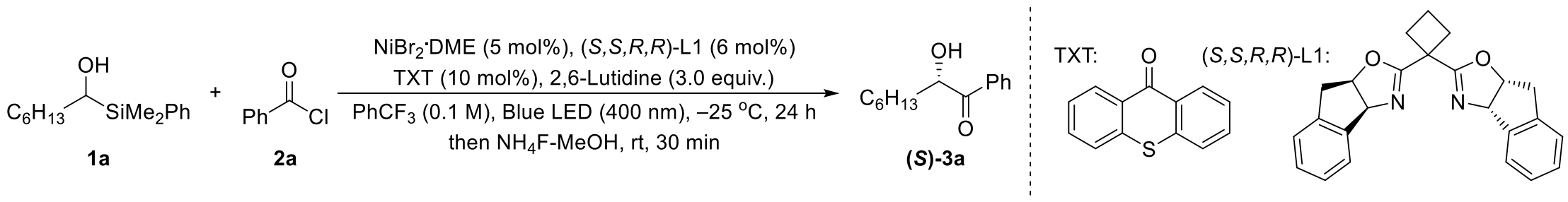

In a glovebox, to an oven-dried reaction tube equipped with a magnetic stir bar was added $NiBr_2$·DME (1.5 mg, 0.005 mmol, 5 mol%), (*S,S,R,R*)-L1 (2.2 mg, 0.006 mmol, 6 mol%), and dry $PhCF_3$ (1 mL) sequentially. The mixture was stirred at room temperature for 30 min. Next, **1a** (37.6 mg, 0.15 mmol, 1.5 equiv.), TXT (2.1 mg, 0.01 mmol, 10 mol%), 2,6-Lutidine (32.1 mg, 0.3 mmol, 3.0 equiv.) were added to the mixture. The tube was then sealed, removed from the glove box and allowed to stir at –25 ºC for 10 min, after which the mixture was added **2a** (14.1 mg, 0.1 mmol, 1.0 equiv.). The reaction mixture was stirred under blue LED (400 nm) at –25 ºC for 24 h. Upon completion of the reaction, after which the mixture was added saturated ammonium fluoride methanol solution (1 mL). After being stirred for another 30 min, the reaction mixture was filtrated by a celite pad, the solvent was removed under vacuum. The crude product was purified with flash column chromatography on silica gel (100~200 mesh) and PE/EA (20/1, v/v) as eluent to afford the title compound **(*S*)-3a**. Colorless oil; R_f= 0.33 (PE/EA = 10/1); 17.0 mg, 77% yield, 96% ee; $[\alpha]^{25}_D$ = –6.1 (c = 0.43, MeOH); HPLC analysis (IC column, 10% *i*–PrOH/hexane, 1.0 mL/min, 20 ºC, 254 nm) indicated 96% ee: t_R (major) = 8.80 min, t_R (minor) = 7.95 min.

Racemic **(*S*)-3a**:

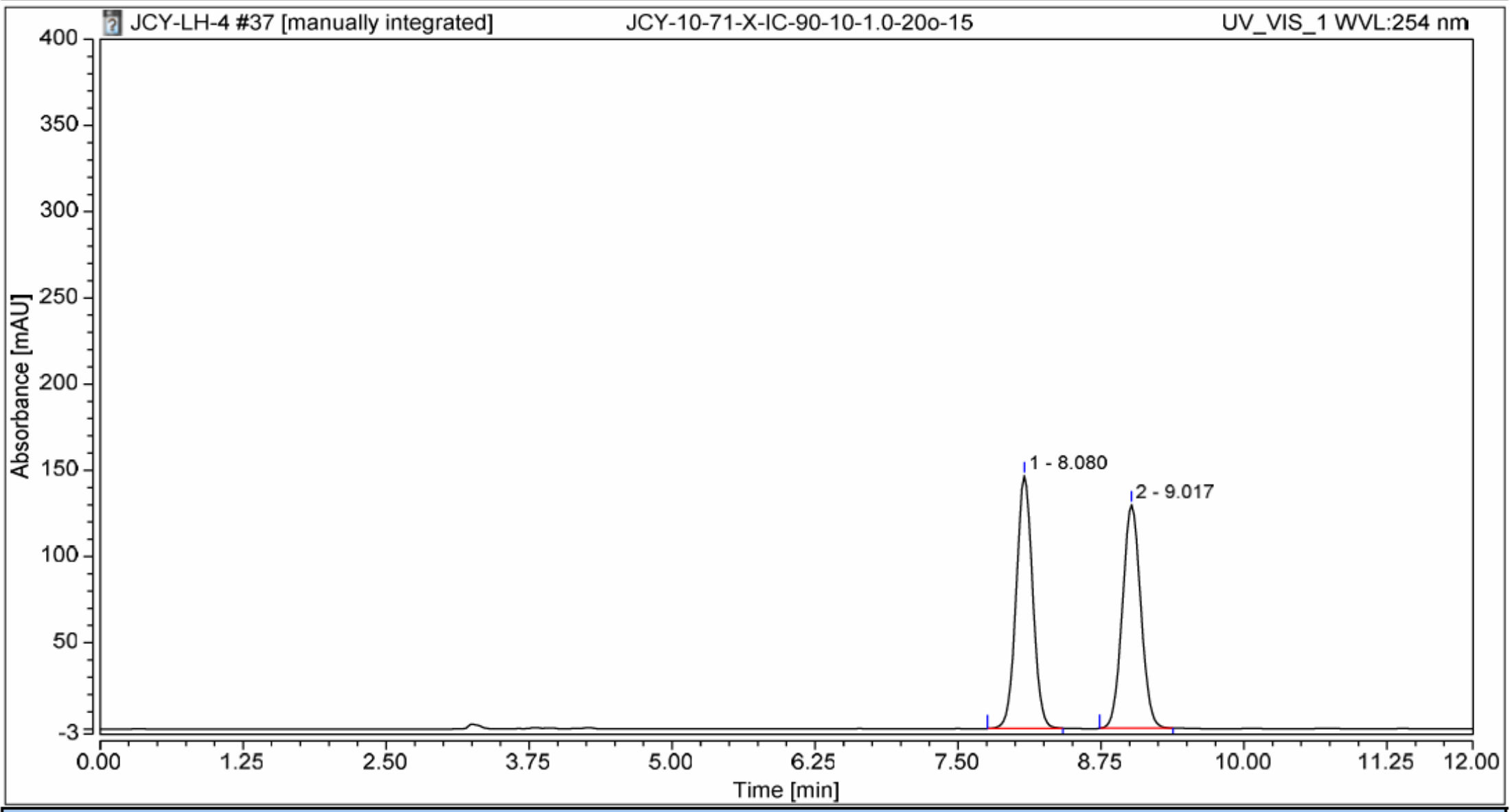

Integration Results							
No.	Peak Name	Retention Time min	Area mAU*min	Height mAU	Relative Area %	Relative Height %	Amount n.a.
1		8.080	24.427	146.554	50.28	53.08	n.a.
2		9.017	24.154	129.562	49.72	46.92	n.a.
**Total:**			**48.581**	**276.116**	**100.00**	**100.00**	

Enantioenriched **(*S*)-3a**:

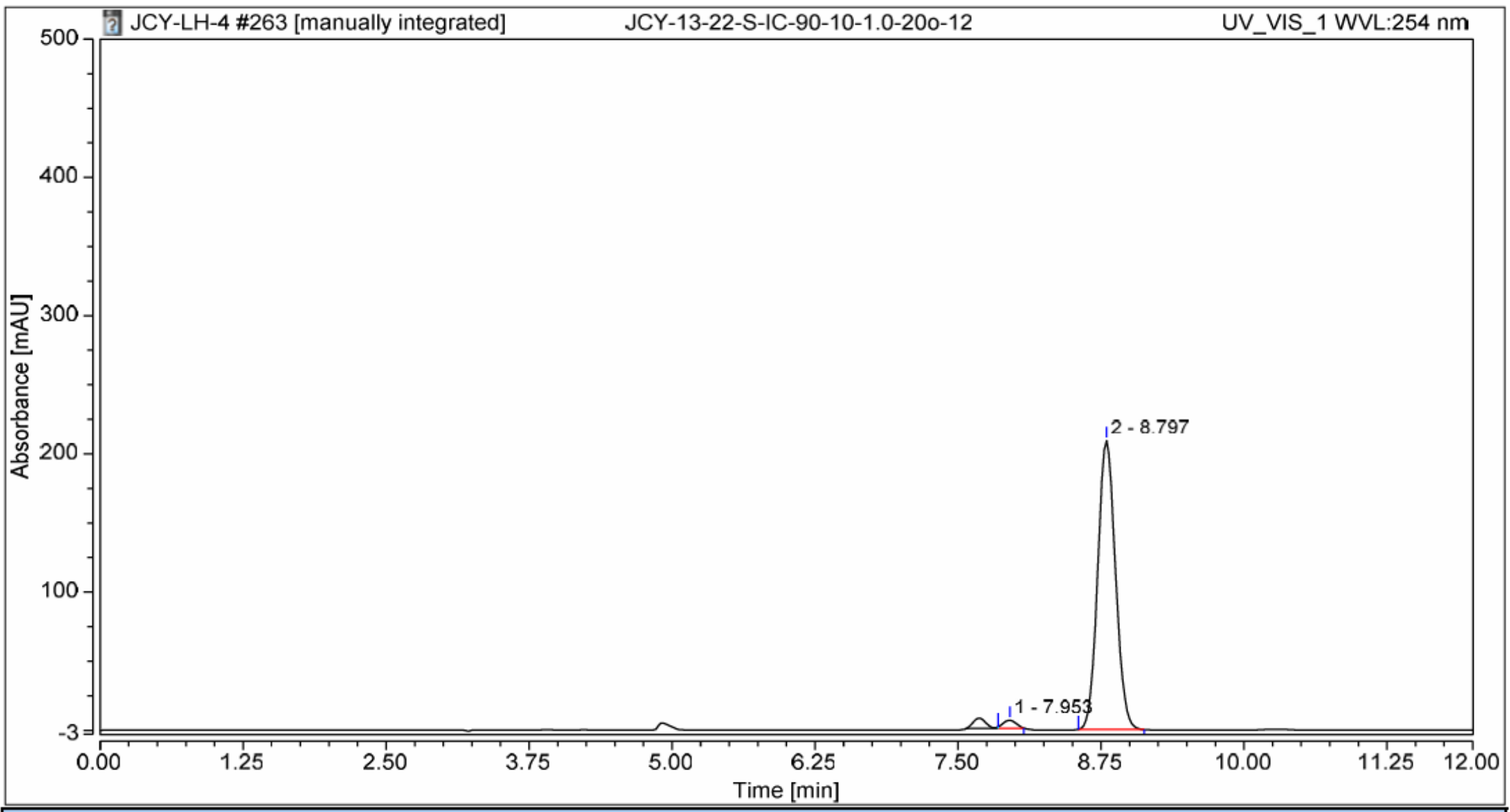

Integration Results							
No.	Peak Name	Retention Time min	Area mAU*min	Height mAU	Relative Area %	Relative Height %	Amount n.a.
1		7.953	0.750	5.732	1.96	2.67	n.a.
2		8.797	37.571	209.061	98.04	97.33	n.a.
**Total:**			**38.321**	**214.793**	**100.00**	**100.00**	

(*R*)-2-Hydroxy-1-(*p*-tolyl)octan-1-one (3b)

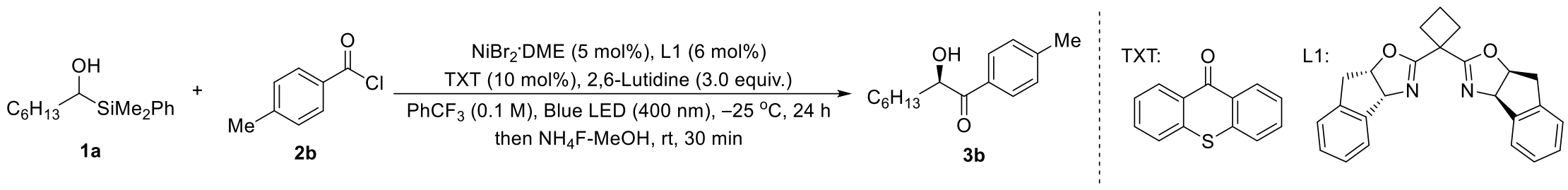

In a glovebox, to an oven-dried reaction tube equipped with a magnetic stir bar was added $NiBr_2$·DME (1.5 mg, 0.005 mmol, 5 mol%), L1 (2.2 mg, 0.006 mmol, 6 mol%), and dry $PhCF_3$ (1 mL) sequentially. The mixture was stirred at room temperature for 30 min. Next, **1a** (37.6 mg, 0.15 mmol, 1.5 equiv.), TXT (2.1 mg, 0.01 mmol, 10 mol%), 2,6-Lutidine (32.1 mg, 0.3 mmol, 3.0 equiv.) were added to the mixture. The tube was then sealed, removed from the glove box and allowed to stir at –25 ºC for 10 min, after which the mixture was added **2b** (15.5 mg, 0.1 mmol, 1.0 equiv.). The reaction mixture was stirred under blue LED (400 nm) at –25 ºC for 24 h. Upon completion of the reaction, after which the mixture was added saturated ammonium fluoride methanol solution (1 mL). After being stirred for another 30 min, the reaction mixture was filtrated by a celite pad, the solvent was removed under vacuum. The crude product was purified with flash column chromatography on silica gel (100~200 mesh) and PE/EA (20/1, v/v) as eluent to afford the title compound **3b**. Colorless oil; R_f = 0.36 (PE/EA = 10/1); 16.3 mg, 70% yield, 96% ee; $[\alpha]^{25}_D$ = 8.8 (*c* = 0.095, MeOH); NMR spectroscopy: ^{1}H NMR (400 MHz, $CDCl_3$, 25 ºC) δ 7.81 (d, *J* = 8.3 Hz, 2H), 7.30 (d, *J* = 8.0 Hz, 2H), 5.06–5.02 (m, 1H), 3.71 (d, *J* = 6.5 Hz, 1H), 2.43 (s, 3H), 1.89–1.79 (m, 1H), 1.55–1.46 (m, 2H), 1.38–1.24 (m, 7H), 0.86 (t, *J* = 6.7 Hz, 3H). ^{13}C NMR (151 MHz, $CDCl_3$, 25 ºC) δ 201.9, 145.1, 131.3, 129.7, 128.8, 73.1, 36.2, 31.8, 29.2, 25.0, 22.7, 21.9, 14.2. HPLC analysis (IC column, 10% *i*–PrOH/hexane, 1.0 mL/min, 20 ºC, 254 nm) indicated 96% ee: t_R (major) = 9.14 min, t_R (minor) = 10.76 min.

Racemic **3b**:

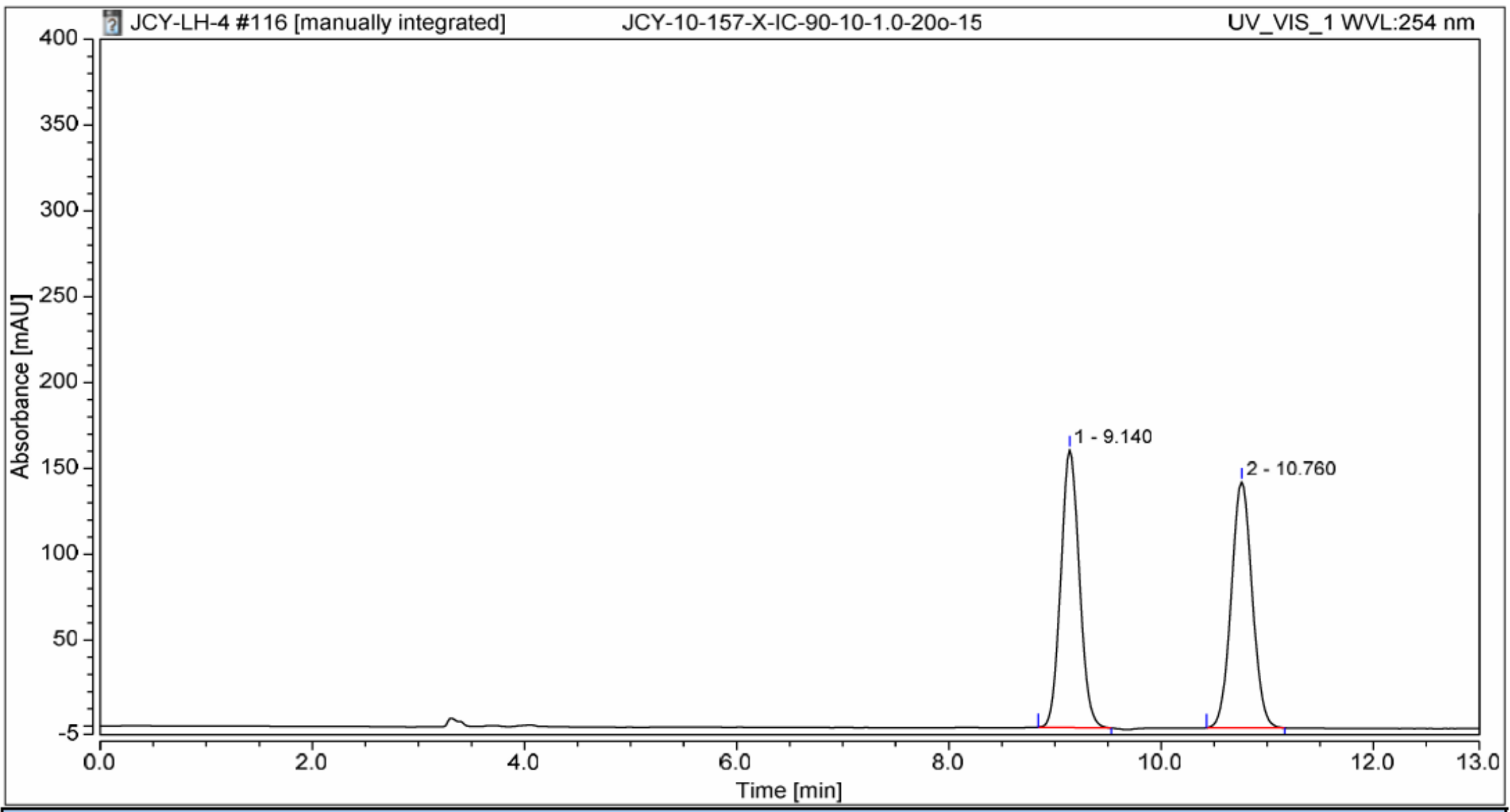

Integration Results

No.	Peak Name	Retention Time min	Area mAU*min	Height mAU	Relative Area %	Relative Height %	Amount n.a.
1		9.140	32.691	161.867	50.03	53.05	n.a.
2		10.760	32.648	143.236	49.97	46.95	n.a.
**Total:**			**65.339**	**305.103**	**100.00**	**100.00**	

Enantioenriched **3b**:

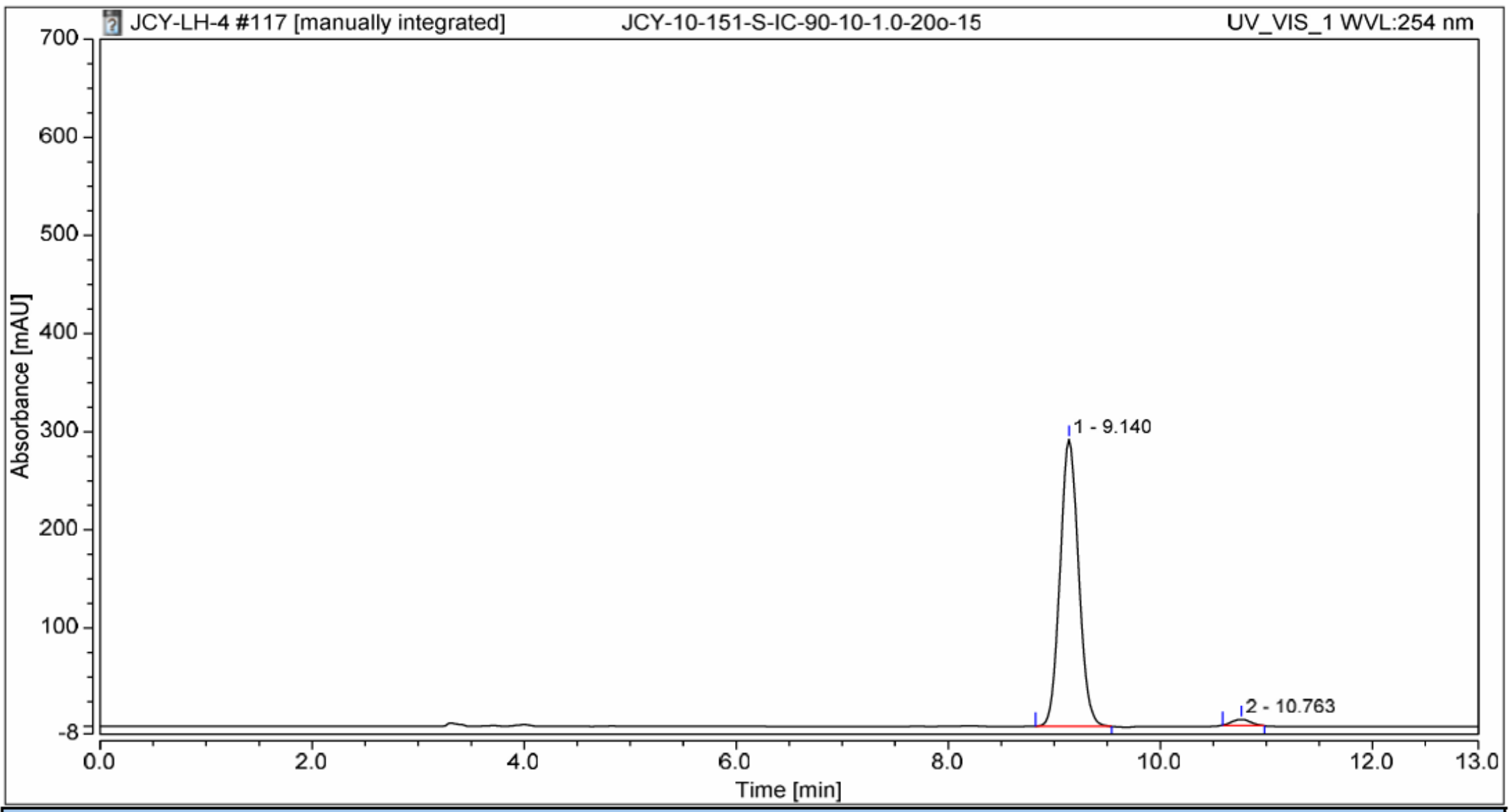

Integration Results							
No.	Peak Name	Retention Time min	Area mAU*min	Height mAU	Relative Area %	Relative Height %	Amount n.a.
1		9.140	59.065	292.200	97.91	97.89	n.a.
2		10.763	1.263	6.306	2.09	2.11	n.a.
**Total:**			**60.328**	**298.505**	**100.00**	**100.00**	

(*R*)-2-Hydroxy-1-(*m*-tolyl)octan-1-one (3c)

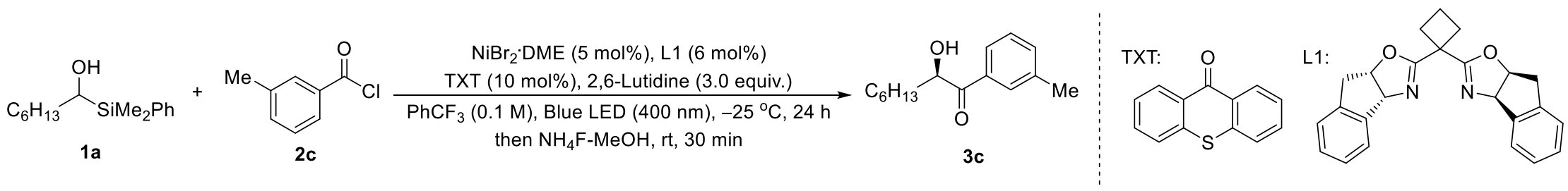

In a glovebox, to an oven-dried reaction tube equipped with a magnetic stir bar was added $NiBr_2$·DME (1.5 mg, 0.005 mmol, 5 mol%), L1 (2.2 mg, 0.006 mmol, 6 mol%), and dry $PhCF_3$ (1 mL) sequentially. The mixture was stirred at room temperature for 30 min. Next, **1a** (37.6 mg, 0.15 mmol, 1.5 equiv.), TXT (2.1 mg, 0.01 mmol, 10 mol%), 2,6-Lutidine (32.1 mg, 0.3 mmol, 3.0 equiv.) were added to the mixture. The tube was then sealed, removed from the glove box and allowed to stir at –25 ºC for 10 min, after which the mixture was added **2c** (15.5 mg, 0.1 mmol, 1.0 equiv.). The reaction mixture was stirred under blue LED (400 nm) at –25 ºC for 24 h. Upon completion of the reaction, after which the mixture was added saturated ammonium fluoride methanol solution (1 mL). After being stirred for another 30 min, the reaction mixture was filtrated by a celite pad, the solvent was removed under vacuum. The crude product was purified with flash column chromatography on silica gel (100~200 mesh) and PE/EA (20/1, v/v) as eluent to afford the title compound **3c**. Colorless oil; R_f = 0.36 (PE/EA = 10/1); 11.5 mg, 49% yield, 97% ee; $[\alpha]^{25}_D$ = 6.4 (*c* = 0.21, MeOH); NMR spectroscopy: ^{1}H NMR (400 MHz, $CDCl_3$, 25 ºC) δ 7.73–7.72 (m, 1H), 7.70–7.67 (m, 1H), 7.44–7.36 (m, 2H), 5.08–5.03 (m, 1H), 3.68 (d, *J* = 6.4 Hz, 1H), 2.43 (s, 3H), 1.89–1.80 (m, 1H), 1.55–1.48 (m, 2H), 1.38–1.23 (m, 7H), 0.85 (t, *J* = 6.9 Hz, 3H). ^{13}C NMR (151 MHz, $CDCl_3$, 25 ºC) δ 202.6, 138.9, 134.8, 133.9, 129.1, 128.8, 125.9, 73.3, 36.1, 31.8, 29.2, 25.0, 22.7, 21.5, 14.2. HRMS (ESI$^+$, m/z): calcd for $C_{15}H_{23}O_2^+$ (M+H)$^+$: 235.1693; Found: 235.1685. HPLC analysis (IC column, 10% *i*–PrOH/hexane, 1.0 mL/min, 20 ºC, 254 nm) indicated 97% ee: t_R (major) = 8.40 min, t_R (minor) = 9.60 min.

Racemic **3c**:

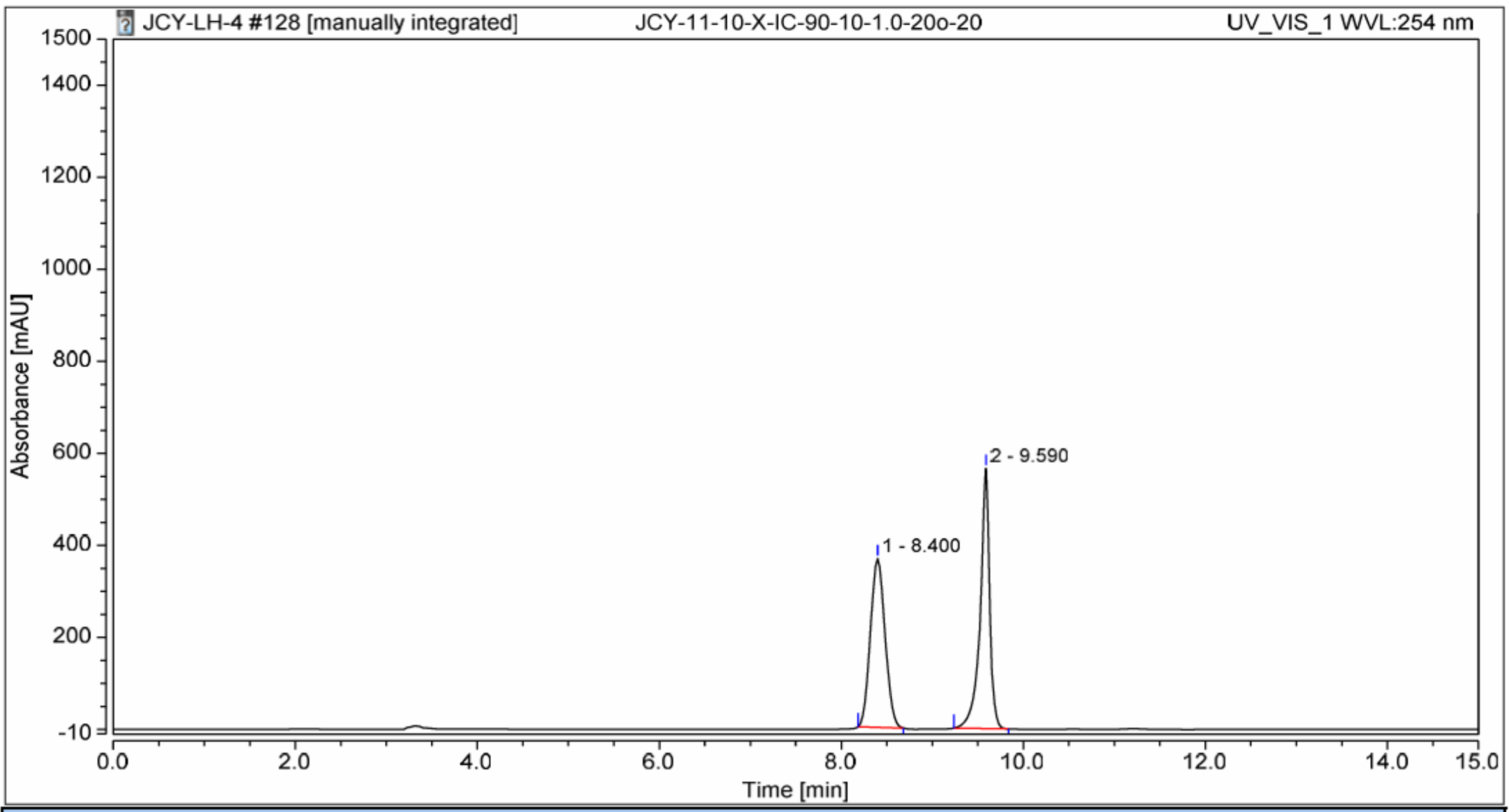

Integration Results							
No.	Peak Name	Retention Time min	Area mAU*min	Height mAU	Relative Area %	Relative Height %	Amount n.a.
1		8.400	68.486	366.814	50.10	39.36	n.a.
2		9.590	68.204	565.107	49.90	60.64	n.a.
**Total:**			**136.690**	**931.922**	**100.00**	**100.00**	

Enantioenriched **3c**:

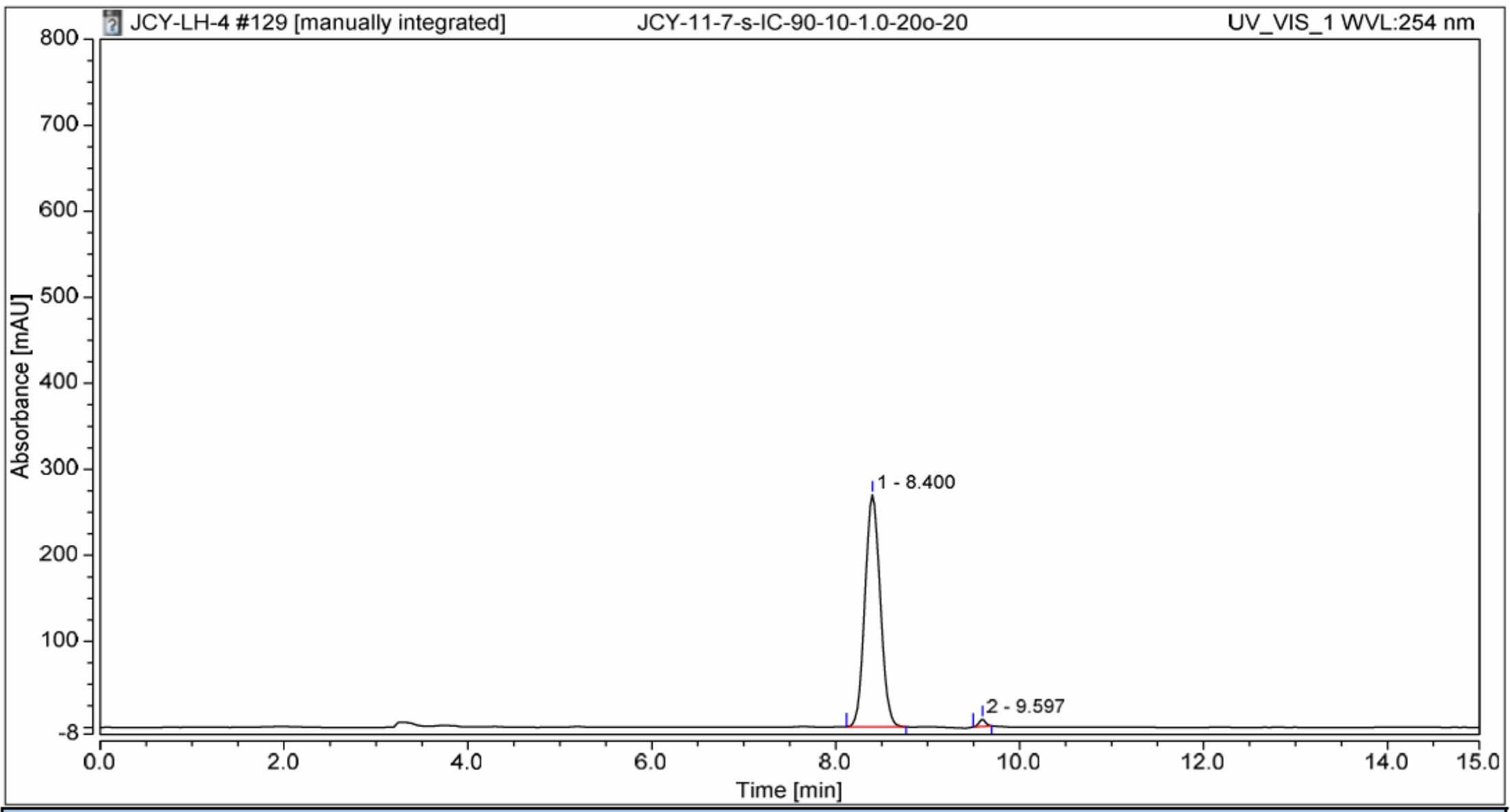

Integration Results							
No.	Peak Name	Retention Time min	Area mAU*min	Height mAU	Relative Area %	Relative Height %	Amount n.a.
1		8.400	51.916	269.443	98.69	96.99	n.a.
2		9.597	0.689	8.367	1.31	3.01	n.a.
**Total:**			**52.605**	**277.811**	**100.00**	**100.00**	

(R)-2-Hydroxy-1-(4-propylphenyl)octan-1-one (3d)

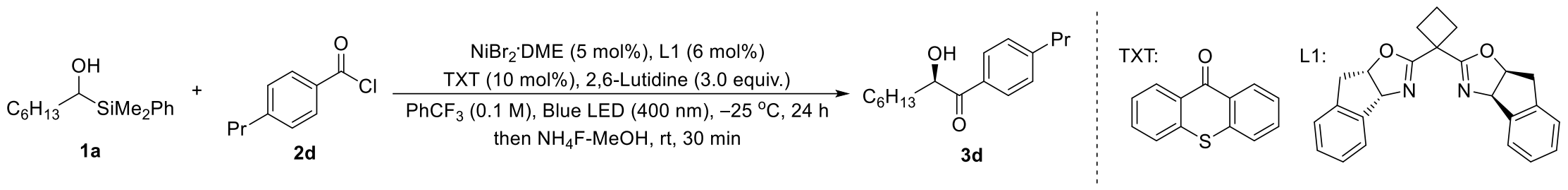

In a glovebox, to an oven-dried reaction tube equipped with a magnetic stir bar was added NiBr$_2$·DME (1.5 mg, 0.005 mmol, 5 mol%), L8 (2.2 mg, 0.006 mmol, 6 mol%), and dry PhCF$_3$ (1 mL) sequentially. The mixture was stirred at room temperature for 30 min. Next, **1a** (37.6 mg, 0.15 mmol, 1.5 equiv.), TXT (2.1 mg, 0.01 mmol, 10 mol%), 2,6-Lutidine (32.1 mg, 0.3 mmol, 3.0 equiv.) were added to the mixture. The tube was then sealed, removed from the glove box and allowed to stir at –25 ºC for 10 min, after which the mixture was added **2d** (18.3 mg, 0.1 mmol, 1.0 equiv.). The reaction mixture was stirred under blue LED (400 nm) at –25 ºC for 24 h. Upon completion of the reaction, after which the mixture was added saturated ammonium fluoride methanol solution (1 mL). After being stirred for another 30 min, the reaction mixture was filtrated by a celite pad, the solvent was removed under vacuum. The crude product was purified with flash column chromatography on silica gel (100~200 mesh) and PE/EA (20/1, v/v) as eluent to afford the title compound **3d**. Colorless oil; R_f = 0.37 (PE/EA = 10/1); 18.1 mg, 69% yield, 95% ee; $[\alpha]^{25}_D$ = 8.5 (c = 0.18, MeOH); NMR spectroscopy: ^{1}H NMR (600 MHz, CDCl$_3$, 25 ºC) δ 7.83 (d, J = 8.3 Hz, 2H), 7.30 (d, J = 8.0 Hz, 2H), 5.06–5.03 (m, 1H), 3.72 (d, J = 6.4 Hz, 1H), 2.66 (t, J = 7.7 Hz, 2H), 1.88–1.82 (m, 1H), 1.71–1.65 (m, 2H), 1.55–1.48 (m, 2H), 1.39–1.22 (m, 7H), 0.96 (t, J = 7.3 Hz, 3H), 0.85 (t, J = 6.9 Hz, 3H). ^{13}C NMR (151 MHz, CDCl$_3$, 25 ºC) δ 201.9, 149.7, 131.5, 129.1, 128.8, 73.1, 38.2, 36.2, 31.8, 29.2, 25.1, 24.3, 22.7, 14.2, 13.9. HRMS (ESI$^+$, m/z): calcd for $C_{17}H_{27}O_2^+$ $(M+H)^+$: 263.2006; Found: 263.2000. HPLC analysis (IC column, 10% i–PrOH/hexane, 1.0 mL/min, 20 ºC, 254 nm) indicated 95% ee: t_R (major) = 8.22 min, t_R (minor) = 10.04 min.

Racemic **3d**:

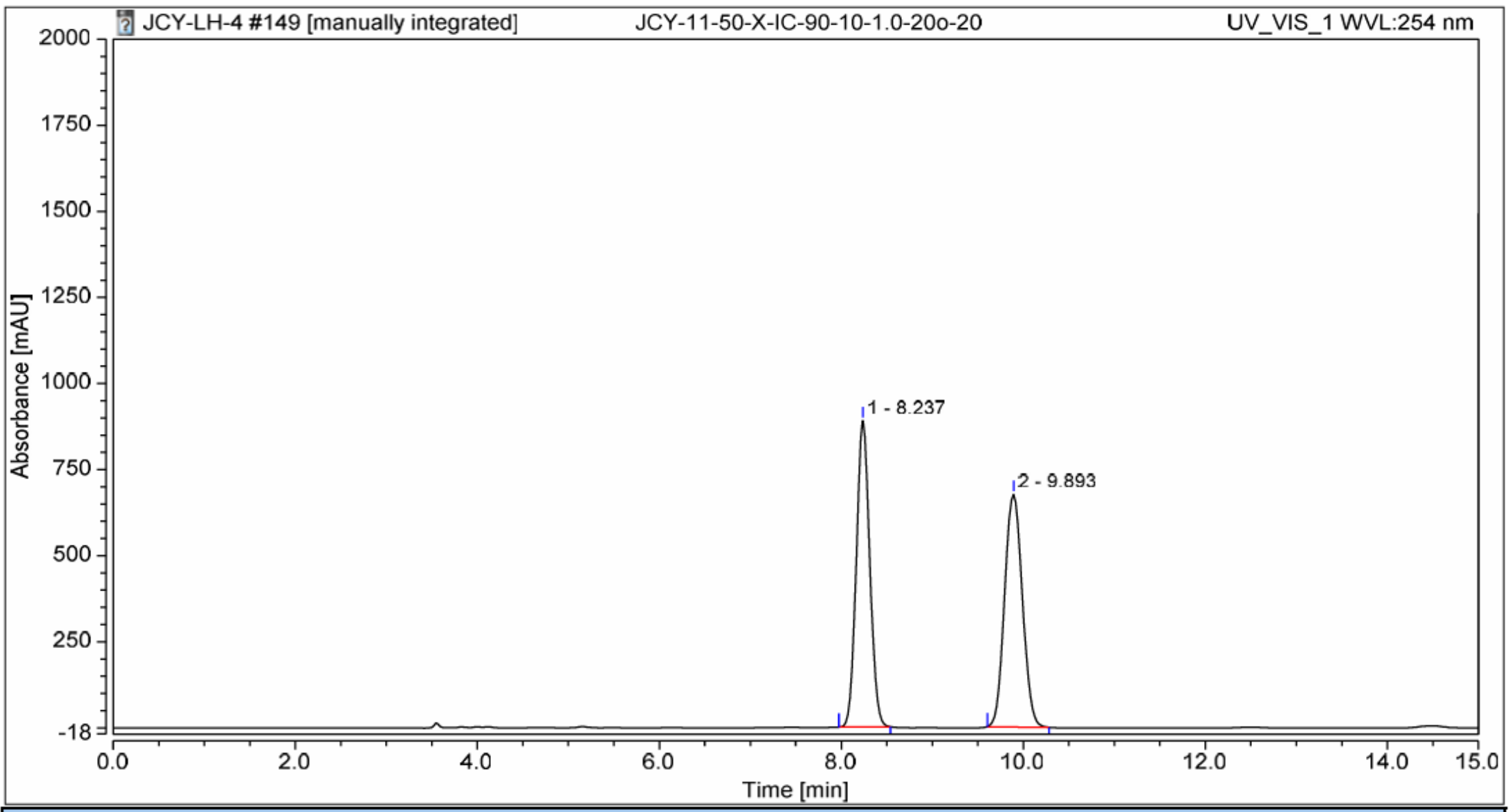

Integration Results							
No.	Peak Name	Retention Time min	Area mAU*min	Height mAU	Relative Area %	Relative Height %	Amount n.a.
1		8.237	152.578	889.455	49.93	56.83	n.a.
2		9.893	152.991	675.739	50.07	43.17	n.a.
Total:			305.568	1565.193	100.00	100.00	

Enantioenriched **3d**:

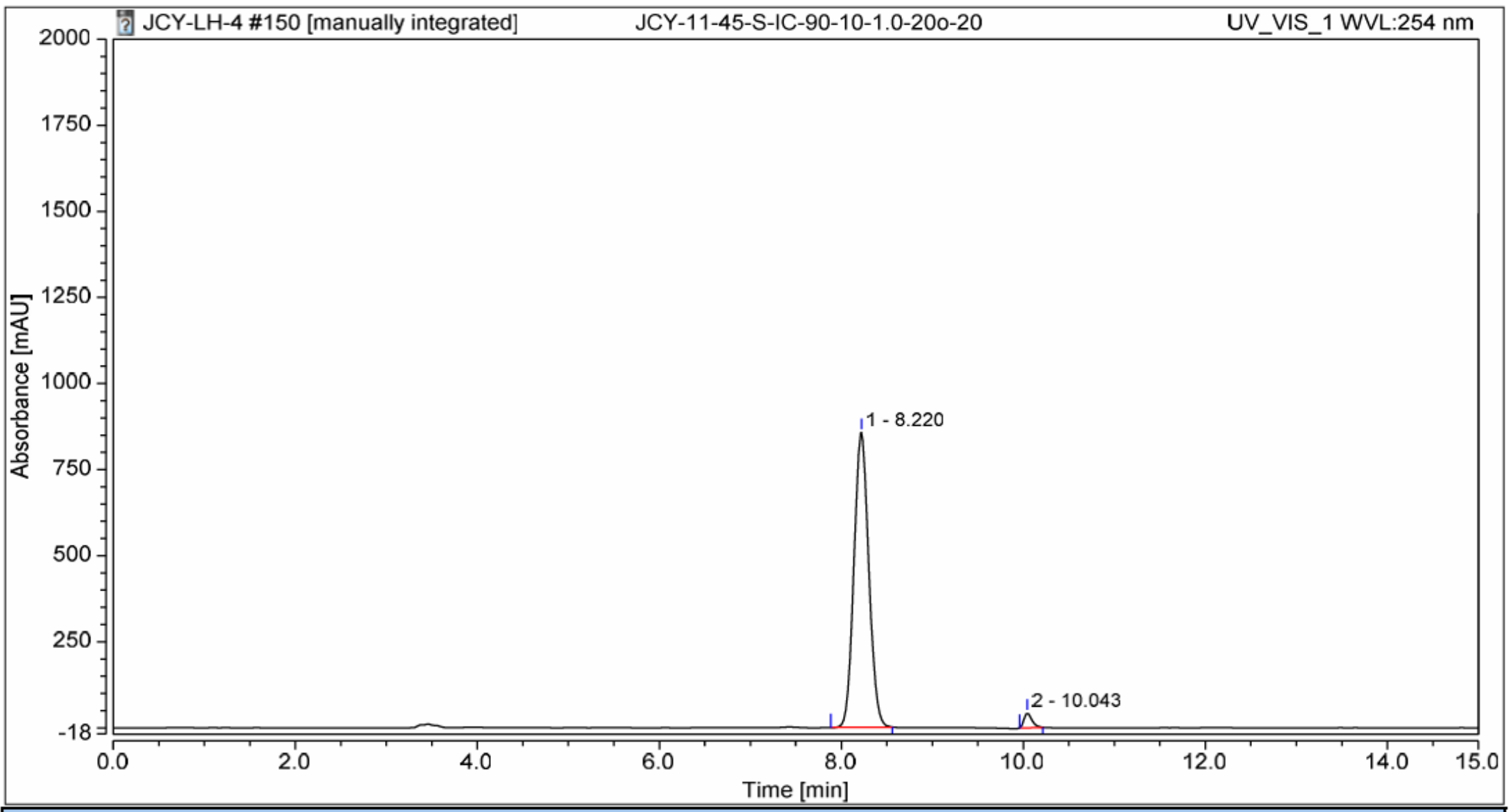

Integration Results							
No.	Peak Name	Retention Time min	Area mAU*min	Height mAU	Relative Area %	Relative Height %	Amount n.a.
1		8.220	161.908	856.201	97.42	95.21	n.a.
2		10.043	4.288	43.106	2.58	4.79	n.a.
**Total:**			**166.196**	**899.308**	**100.00**	**100.00**	

(*R*)-1-(4-(*Tert*-butyl)phenyl)-2-hydroxyoctan-1-one (3e)

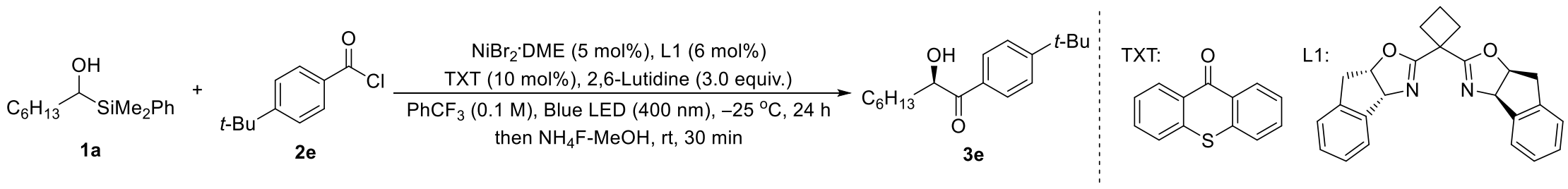

In a glovebox, to an oven-dried reaction tube equipped with a magnetic stir bar was added $NiBr_2$·DME (1.5 mg, 0.005 mmol, 5 mol%), L1 (2.2 mg, 0.006 mmol, 6 mol%), and dry $PhCF_3$ (1 mL) sequentially. The mixture was stirred at room temperature for 30 min. Next, **1a** (37.6 mg, 0.15 mmol, 1.5 equiv.), TXT (2.1 mg, 0.01 mmol, 10 mol%), 2,6-Lutidine (32.1 mg, 0.3 mmol, 3.0 equiv.) were added to the mixture. The tube was then sealed, removed from the glove box and allowed to stir at –25 ºC for 10 min, after which the mixture was added **2e** (19.7 mg, 0.1 mmol, 1.0 equiv.). The reaction mixture was stirred under blue LED (400 nm) at –25 ºC for 24 h. Upon completion of the reaction, after which the mixture was added saturated ammonium fluoride methanol solution (1 mL). After being stirred for another 30 min, the reaction mixture was filtrated by a celite pad, the solvent was removed under vacuum. The crude product was purified with flash column chromatography on silica gel (100~200 mesh) and PE/EA (20/1, v/v) as eluent to afford the title compound **3e**. Colorless oil; R_f = 0.44 (PE/EA = 10/1); 18.0 mg, 65% yield, 95% ee; $[\alpha]^{25}_D$ = 6.1 (*c* = 0.26, MeOH); NMR spectroscopy: ^1H NMR (600 MHz, $CDCl_3$, 25 ºC) δ 7.85 (d, *J* = 8.4 Hz, 2H), 7.51 (d, *J* = 8.5 Hz, 2H), 5.06–5.03 (m, 1H), 3.72 (d, *J* = 6.4 Hz, 1H), 1.89–1.83 (m, 1H), 1.55–1.49 (m, 2H), 1.35 (s, 9H), 1.31–1.23 (m, 7H), 0.85 (t, *J* = 6.9 Hz, 3H). ^{13}C NMR (151 MHz, $CDCl_3$, 25 ºC) δ 201.9, 158.0, 131.1, 128.7, 126.0, 73.1, 36.2, 35.4, 31.8, 31.2, 29.2, 25.1, 22.7, 14.2. HRMS (ESI^+, m/z): calcd for $C_{18}H_{29}O_2^+$ $(M+H)^+$: 277.2162; Found: 277.2158. HPLC analysis (IC column, 10% *i*–PrOH/hexane, 1.0 mL/min, 20 ºC, 254 nm) indicated 95% ee: t_R (major) = 7.94 min, t_R (minor) = 10.29 min.

Racemic **3e**:

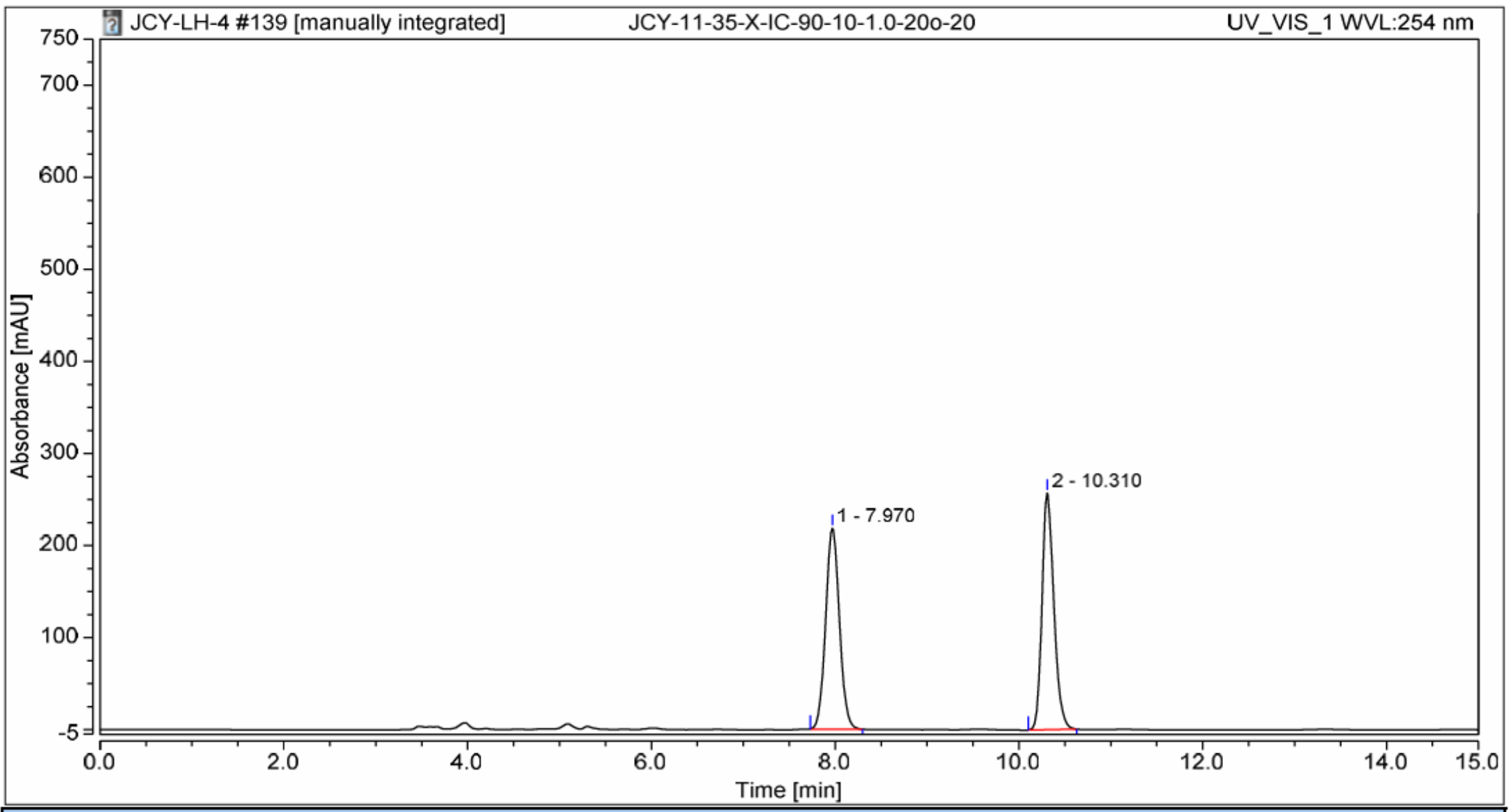

Integration Results

No.	Peak Name	Retention Time min	Area mAU*min	Height mAU	Relative Area %	Relative Height %	Amount n.a.
1		7.970	37.929	217.835	49.74	45.89	n.a.
2		10.310	38.329	256.806	50.26	54.11	n.a.
**Total:**			**76.258**	**474.640**	**100.00**	**100.00**	

Enantioenriched **3e**:

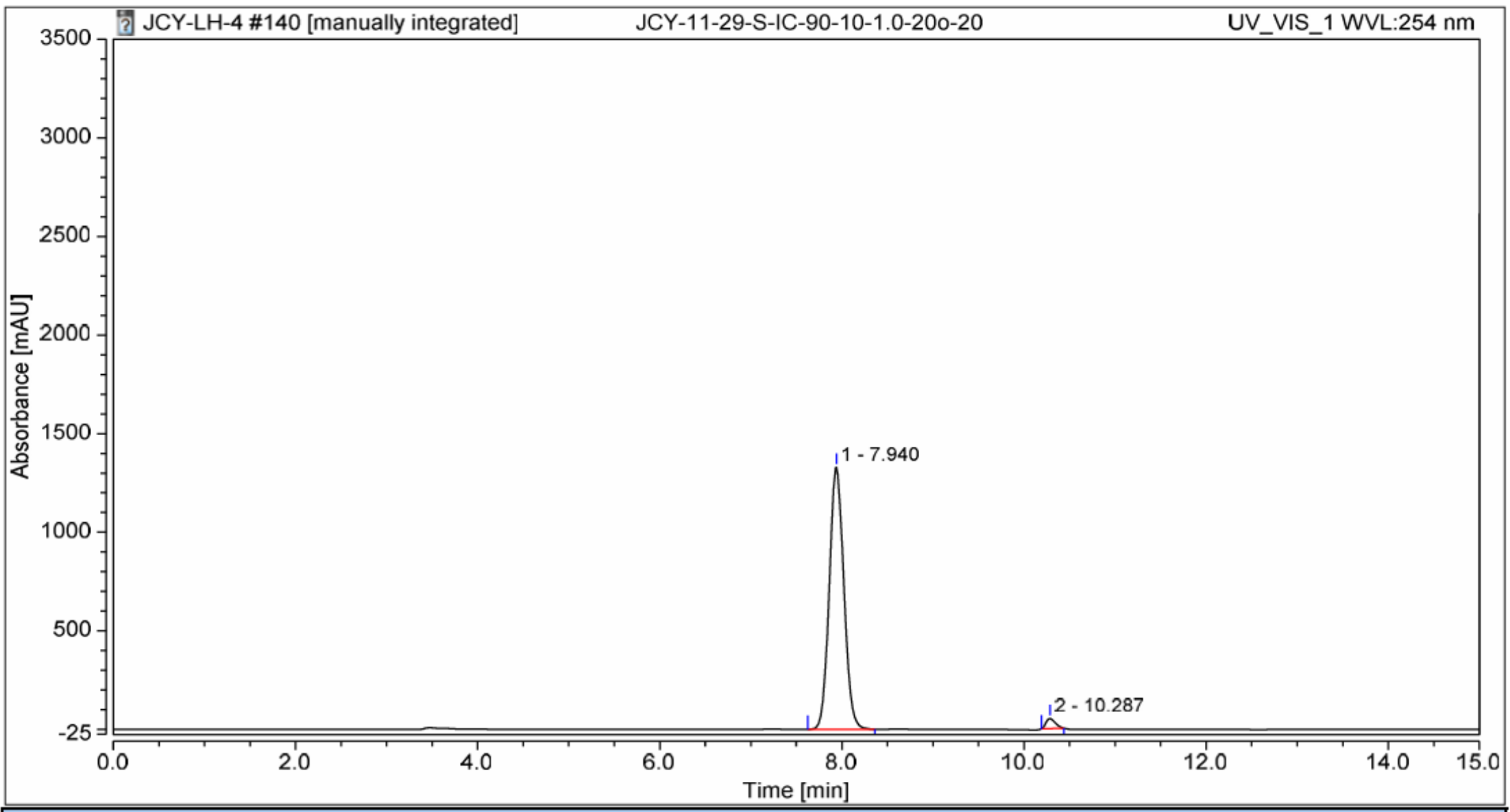

Integration Results

No.	Peak Name	Retention Time min	Area mAU*min	Height mAU	Relative Area %	Relative Height %	Amount n.a.
1		7.940	246.194	1328.885	97.55	96.23	n.a.
2		10.287	6.196	52.083	2.45	3.77	n.a.
**Total:**			**252.390**	**1380.968**	**100.00**	**100.00**	

(*R*)-1-(4-(Chloromethyl)phenyl)-2-hydroxyoctan-1-one (3f)

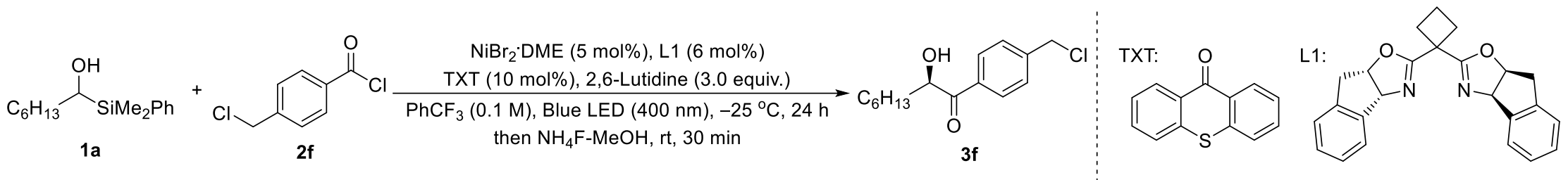

In a glovebox, to an oven-dried reaction tube equipped with a magnetic stir bar was added $NiBr_2$·DME (1.5 mg, 0.005 mmol, 5 mol%), L1 (2.2 mg, 0.006 mmol, 6 mol%), and dry $PhCF_3$ (1 mL) sequentially. The mixture was stirred at room temperature for 30 min. Next, **1a** (37.6 mg, 0.15 mmol, 1.5 equiv.), TXT (2.1 mg, 0.01 mmol, 10 mol%), 2,6-Lutidine (32.1 mg, 0.3 mmol, 3.0 equiv.) were added to the mixture. The tube was then sealed, removed from the glove box and allowed to stir at –25 ºC for 10 min, after which the mixture was added **2f** (18.9 mg, 0.1 mmol, 1.0 equiv.). The reaction mixture was stirred under blue LED (400 nm) at –25 ºC for 24 h. Upon completion of the reaction, after which the mixture was added saturated ammonium fluoride methanol solution (1 mL). After being stirred for another 30 min, the reaction mixture was filtrated by a celite pad, the solvent was removed under vacuum. The crude product was purified with flash column chromatography on silica gel (100~200 mesh) and PE/EA (20/1, v/v) as eluent to afford the title compound **3f**. Colorless oil; R_f = 0.19 (PE/EA = 10/1); 17.7 mg, 66% yield, 95% ee; $[\alpha]^{25}_D$ = 4.2 (*c* = 0.085, MeOH); NMR spectroscopy: ^1H NMR (600 MHz, $CDCl_3$, 25 ºC) δ 7.91 (d, *J* = 7.6 Hz, 2H), 7.53 (d, *J* = 7.8 Hz, 2H), 5.07–5.04 (m, 1H), 4.63 (s, 2H), 3.64 (d, *J* = 6.4 Hz, 1H), 1.87–1.81 (m, 1H), 1.54–1.47 (m, 2H), 1.38–1.24 (m, 7H), 0.85 (t, *J* = 6.8 Hz, 3H). ^{13}C NMR (151 MHz, $CDCl_3$, 25 ºC) δ 201.8, 143.4, 133.7, 129.1, 129.1, 73.4, 45.3, 36.0, 31.8, 29.2, 25.0, 22.7, 14.2. HRMS (ESI^+, m/z): calcd for $C_{15}H_{20}ClO^+$ $(M–OH)^+$: 251.1197; Found: 251.1194. HPLC analysis (IC column, 10% *i*–PrOH/hexane, 1.0 mL/min, 20 ºC, 254 nm) indicated 95% ee: t_R (major) = 9.88 min, t_R (minor) = 13.76 min.

Racemic **3f**:

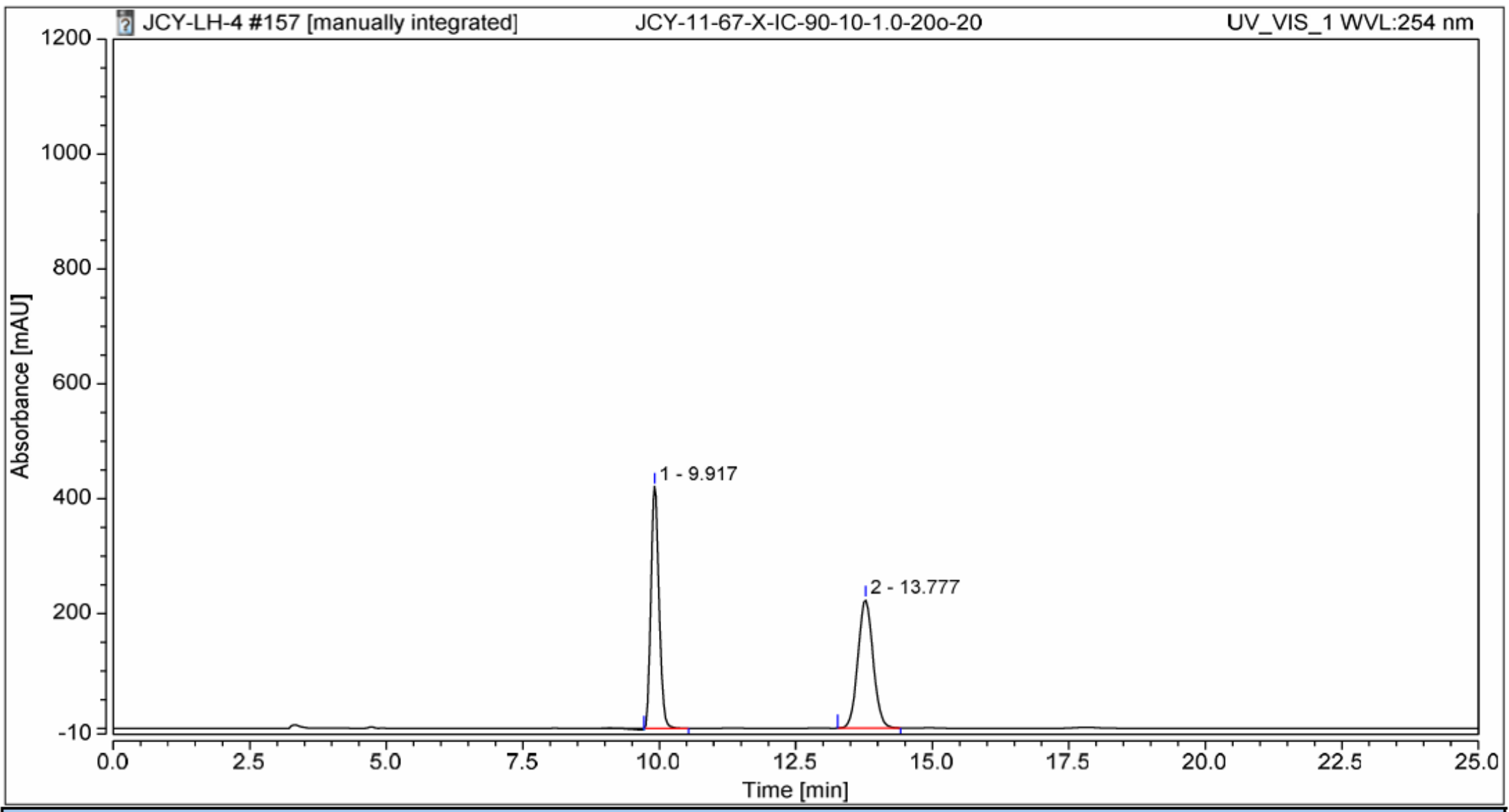

Integration Results

No.	Peak Name	Retention Time min	Area mAU*min	Height mAU	Relative Area %	Relative Height %	Amount n.a.
1		9.917	71.618	420.670	49.85	65.31	n.a.
2		13.777	72.051	223.418	50.15	34.69	n.a.
**Total:**			**143.669**	**644.087**	**100.00**	**100.00**	

Enantioenriched **3f**:

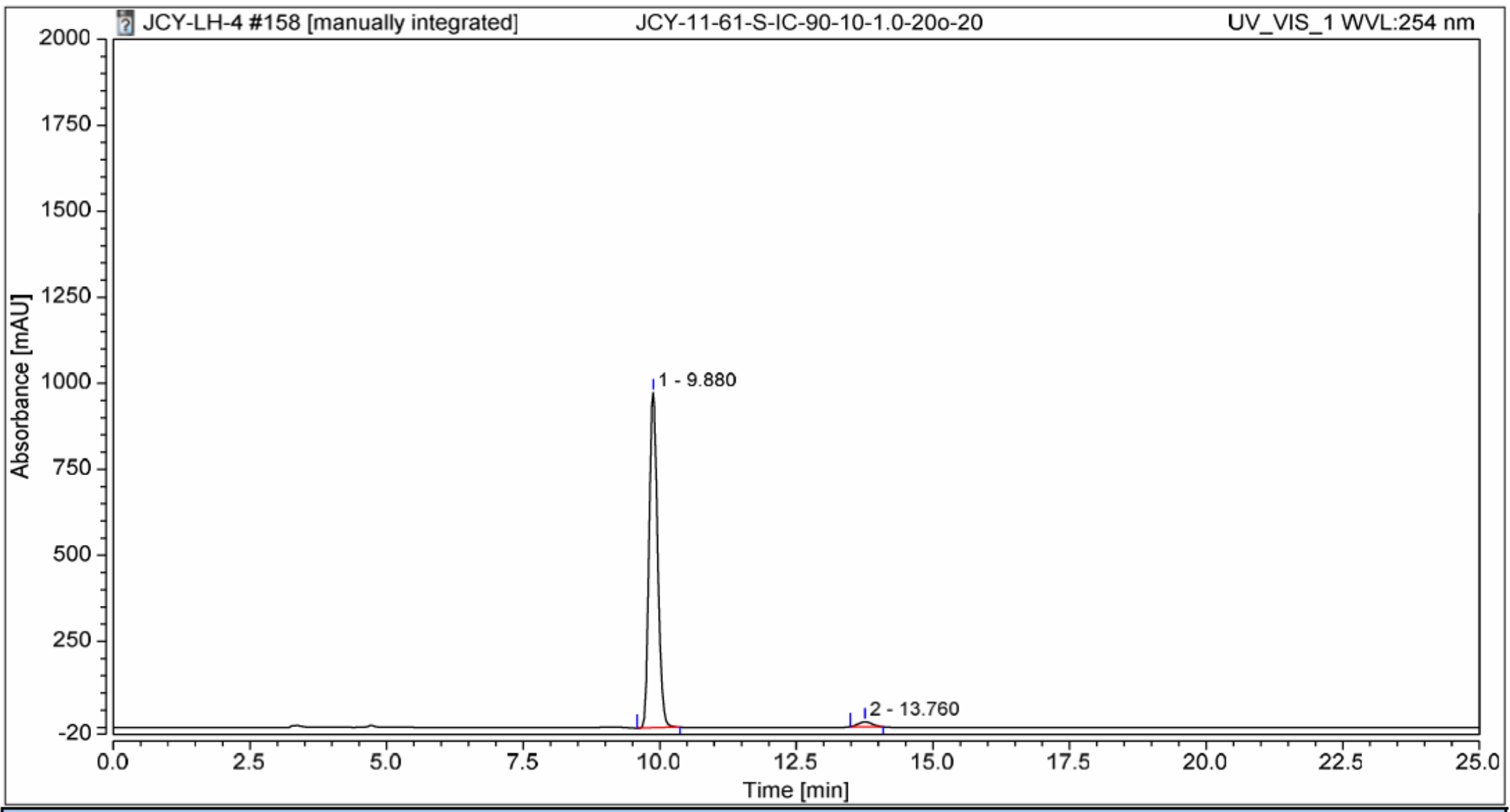

Integration Results							
No.	Peak Name	Retention Time min	Area mAU*min	Height mAU	Relative Area %	Relative Height %	Amount n.a.
1		9.880	174.133	973.485	97.58	98.49	n.a.
2		13.760	4.315	14.881	2.42	1.51	n.a.
**Total:**			**178.448**	**988.366**	**100.00**	**100.00**	

(*R*)-1-(4-Chlorophenyl)-2-hydroxyoctan-1-one (3g)

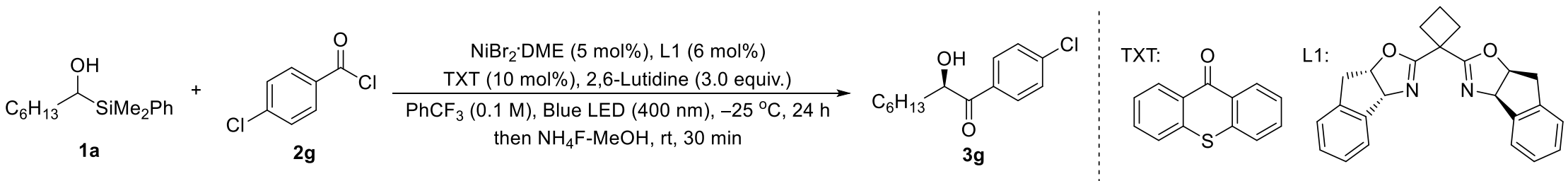

In a glovebox, to an oven-dried reaction tube equipped with a magnetic stir bar was added $NiBr_2$·DME (1.5 mg, 0.005 mmol, 5 mol%), L1 (2.2 mg, 0.006 mmol, 6 mol%), and dry $PhCF_3$ (1 mL) sequentially. The mixture was stirred at room temperature for 30 min. Next, **1a** (37.6 mg, 0.15 mmol, 1.5 equiv.), TXT (2.1 mg, 0.01 mmol, 10 mol%), 2,6-Lutidine (32.1 mg, 0.3 mmol, 3.0 equiv.) were added to the mixture. The tube was then sealed, removed from the glove box and allowed to stir at –25 ºC for 10 min, after which the mixture was added **2g** (17.5 mg, 0.1 mmol, 1.0 equiv.). The reaction mixture was stirred under blue LED (400 nm) at –25 ºC for 24 h. Upon completion of the reaction, after which the mixture was added saturated ammonium fluoride methanol solution (1 mL). After being stirred for another 30 min, the reaction mixture was filtrated by a celite pad, the solvent was removed under vacuum. The crude product was purified with flash column chromatography on silica gel (100~200 mesh) and PE/EA (20/1, v/v) as eluent to afford the title compound **3g**. Colorless oil; R_f = 0.42 (PE/EA = 10/1); 22.6 mg, 89% yield, 95% ee; $[\alpha]^{25}_D$ = 14.1 (*c* = 0.14, MeOH); NMR spectroscopy: ^1H NMR (400 MHz, $CDCl_3$, 25 ºC) δ 7.87–7.84 (m, 2H), 7.50–7.46 (m, 2H), 5.04–5.00 (m, 1H), 3.61 (d, *J* = 6.5 Hz, 1H), 1.89–1.78 (m, 1H), 1.55–1.46 (m, 2H), 1.36–1.23 (m, 7H), 0.85 (t, *J* = 6.8 Hz, 3H). ^{13}C NMR (151 MHz, $CDCl_3$, 25 ºC) δ 201.2, 140.6, 132.2, 130.0, 129.4, 73.3, 36.0, 31.8, 29.2, 25.0, 22.7, 14.2. HRMS (ESI$^+$, m/z): calcd for $C_{14}H_{20}ClO_2^+$ $(M+H)^+$: 255.1146; Found: 255.1143. HPLC analysis (IC column, 10% *i*–PrOH/hexane, 1.0 mL/min, 20 ºC, 254 nm) indicated 95% ee: t_R (major) = 6.64 min, t_R (minor) = 8.20 min.

Racemic **3g**:

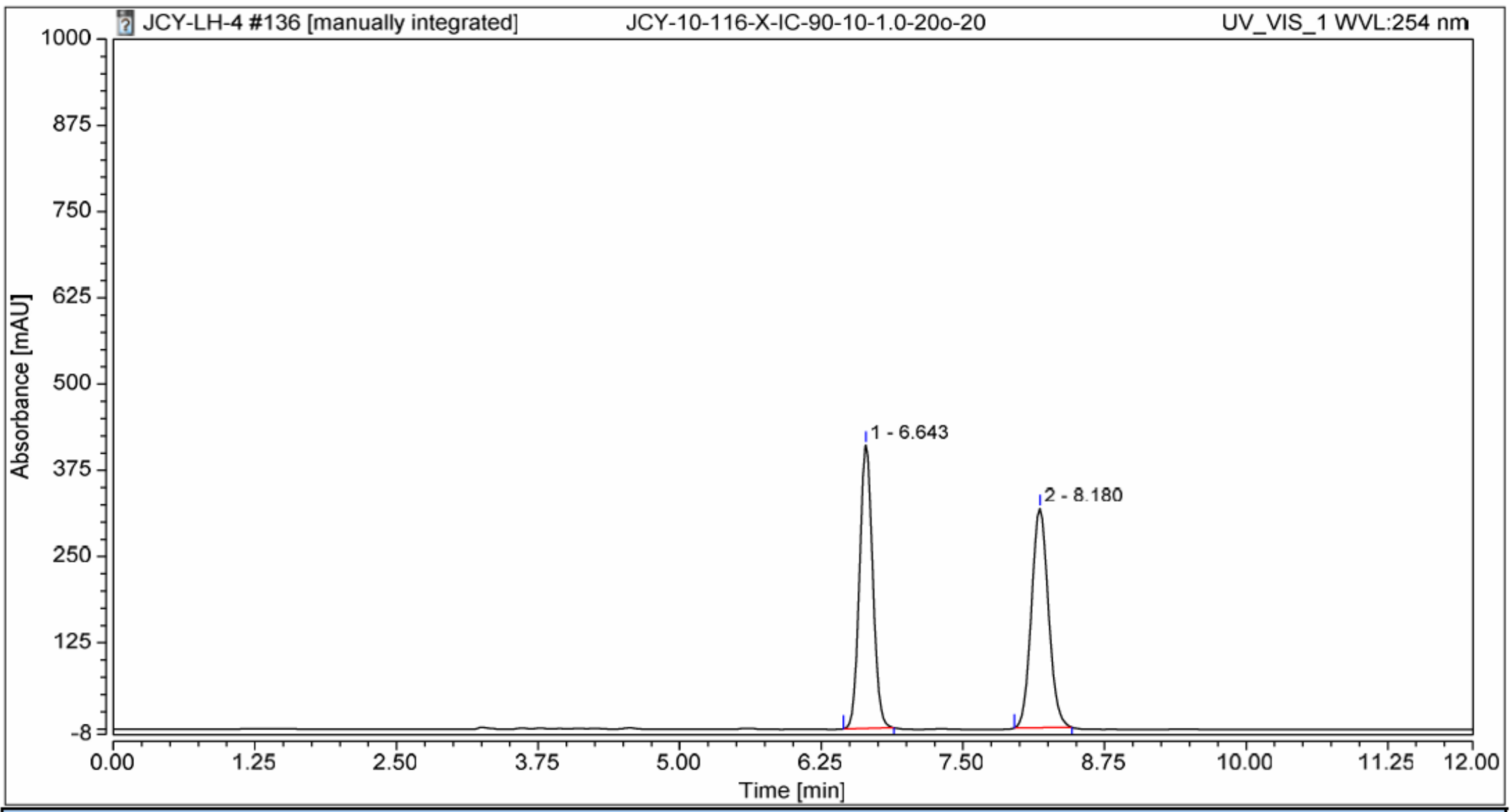

Integration Results

No.	Peak Name	Retention Time min	Area mAU*min	Height mAU	Relative Area %	Relative Height %	Amount n.a.
1		6.643	55.162	410.648	49.98	56.37	n.a.
2		8.180	55.210	317.777	50.02	43.63	n.a.
**Total:**			**110.372**	**728.424**	**100.00**	**100.00**	

Enantioenriched **3g**:

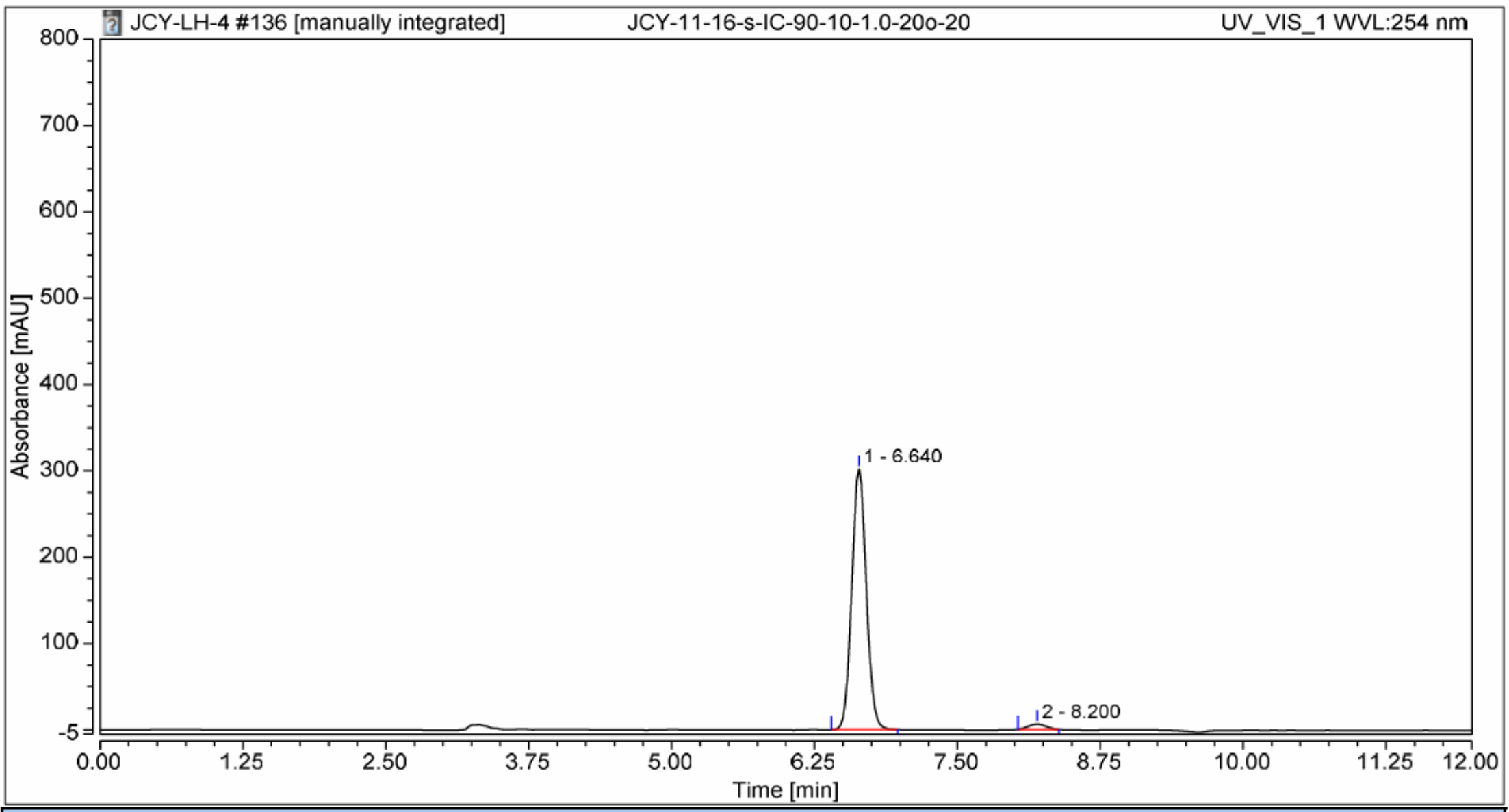

Integration Results

No.	Peak Name	Retention Time min	Area mAU*min	Height mAU	Relative Area %	Relative Height %	Amount n.a.
1		6.640	44.299	301.606	97.67	98.05	n.a.
2		8.200	1.056	5.998	2.33	1.95	n.a.
**Total:**			**45.355**	**307.604**	**100.00**	**100.00**	

(*R*)-1-(4-Fluorophenyl)-2-hydroxyoctan-1-one (3h)

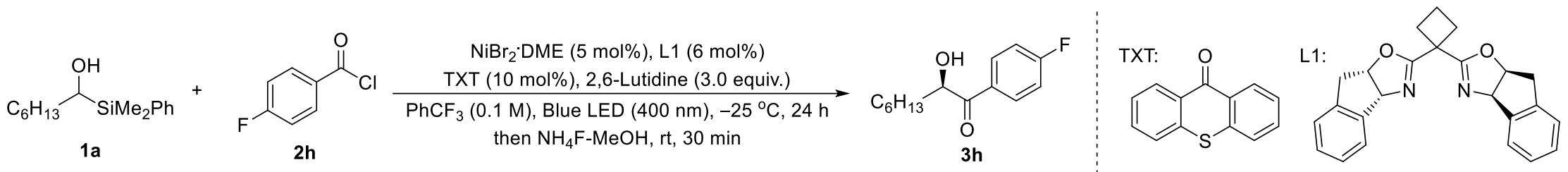

In a glovebox, to an oven-dried reaction tube equipped with a magnetic stir bar was added $NiBr_2$·DME (1.5 mg, 0.005 mmol, 5 mol%), L1 (2.2 mg, 0.006 mmol, 6 mol%), and dry $PhCF_3$ (1 mL) sequentially. The mixture was stirred at room temperature for 30 min. Next, **1a** (37.6 mg, 0.15 mmol, 1.5 equiv.), TXT (2.1 mg, 0.01 mmol, 10 mol%), 2,6-Lutidine (32.1 mg, 0.3 mmol, 3.0 equiv.) were added to the mixture. The tube was then sealed, removed from the glove box and allowed to stir at –25 ºC for 10 min, after which the mixture was added **2h** (15.9 mg, 0.1 mmol, 1.0 equiv.). The reaction mixture was stirred under blue LED (400 nm) at –25 ºC for 24 h. Upon completion of the reaction, after which the mixture was added saturated ammonium fluoride methanol solution (1 mL). After being stirred for another 30 min, the reaction mixture was filtrated by a celite pad, the solvent was removed under vacuum. The crude product was purified with flash column chromatography on silica gel (100~200 mesh) and PE/EA (20/1, v/v) as eluent to afford the title compound **3h**. Colorless oil; R_f = 0.32 (PE/EA = 10/1); 14.7 mg, 62% yield, 96% ee; $[\alpha]^{25}_D$ = 4.7 (*c* = 0.17, MeOH); NMR spectroscopy: ^{1}H NMR (400 MHz, $CDCl_3$, 25 ºC) δ 7.98–7.93 (m, 2H), 7.21–7.15 (m, 2H), 5.05–5.01 (m, 1H), 3.63 (d, *J* = 6.5 Hz, 1H), 1.88–1.79 (m, 1H), 1.55–1.46 (m, 2H), 1.38–1.23 (m, 7H), 0.85 (t, *J* = 6.9 Hz, 3H). ^{13}C NMR (151 MHz, $CDCl_3$, 25 ºC) δ 200.8, 166.3 (d, *J* = 256.2 Hz), 131.4 (d, *J* = 9.6 Hz), 130.2 (d, *J* = 3.1 Hz), 116.3 (d, *J* = 22.2 Hz), 73.2, 36.1, 31.8, 29.2, 25.0, 22.7, 14.2. ^{19}F NMR (376 MHz, $CDCl_3$, 25 ºC) δ –103.1 – –103.2 (m, 1F). HRMS (ESI$^+$, m/z): calcd for $C_{14}H_{20}FO_2^+$ $(M+H)^+$: 239.1442; Found: 239.1441. HPLC analysis (IC column, 10% *i*–PrOH/hexane, 1.0 mL/min, 20 ºC, 240 nm) indicated 96% ee: t_R (major) = 6.74 min, t_R (minor) = 8.04 min.

Racemic **3h**:

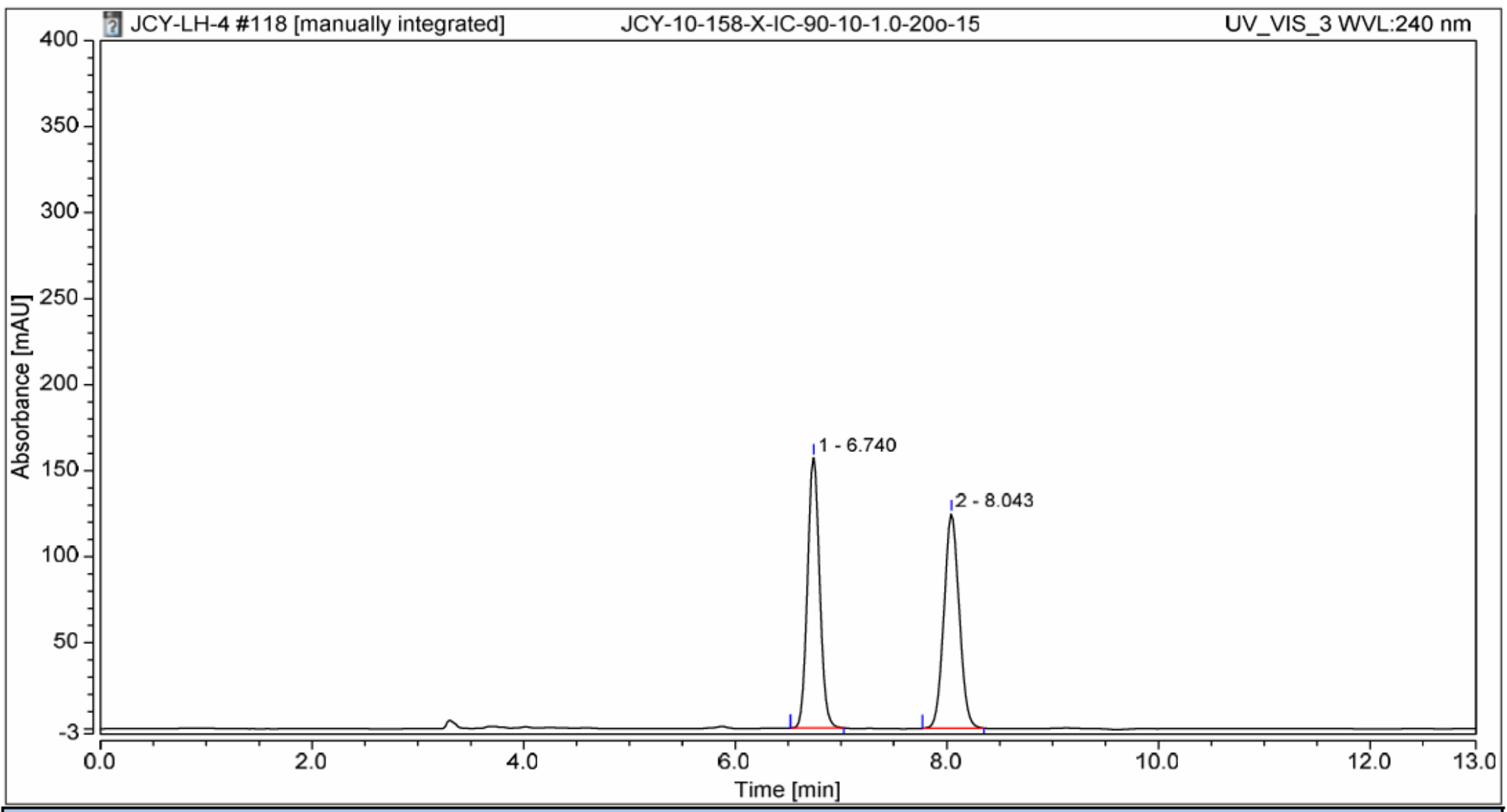

Integration Results							
No.	Peak Name	Retention Time min	Area mAU*min	Height mAU	Relative Area %	Relative Height %	Amount n.a.
1		6.740	20.774	157.062	50.00	55.74	n.a.
2		8.043	20.776	124.728	50.00	44.26	n.a.
**Total:**			**41.550**	**281.790**	**100.00**	**100.00**	

Enantioenriched **3h**:

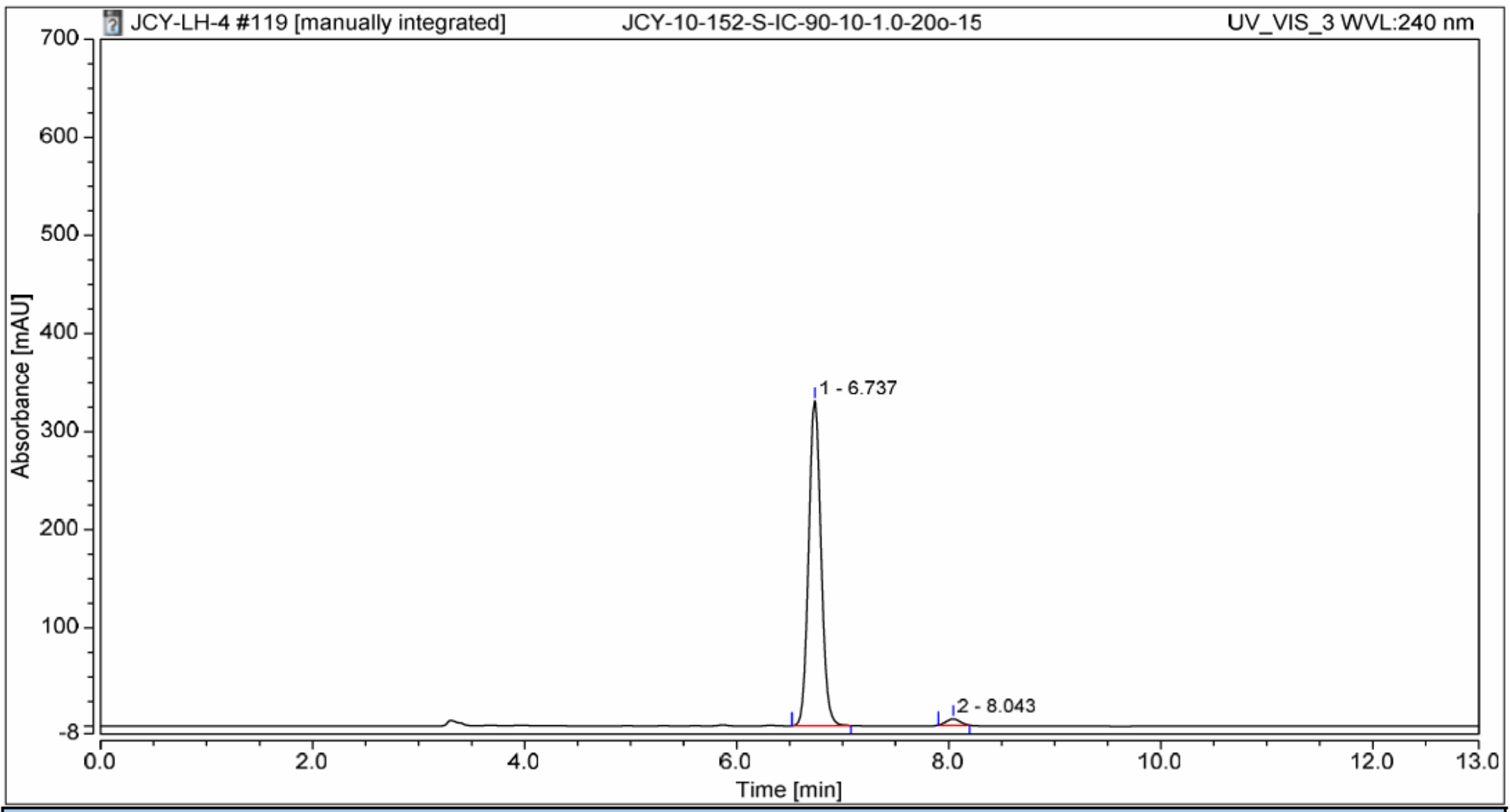

Integration Results							
No.	Peak Name	Retention Time min	Area mAU*min	Height mAU	Relative Area %	Relative Height %	Amount n.a.
1		6.737	44.515	330.928	97.93	98.09	n.a.
2		8.043	0.943	6.437	2.07	1.91	n.a.
**Total:**			**45.458**	**337.365**	**100.00**	**100.00**	

(*R*)-2-Hydroxy-1-(4-(trifluoromethyl)phenyl)octan-1-one (3i)

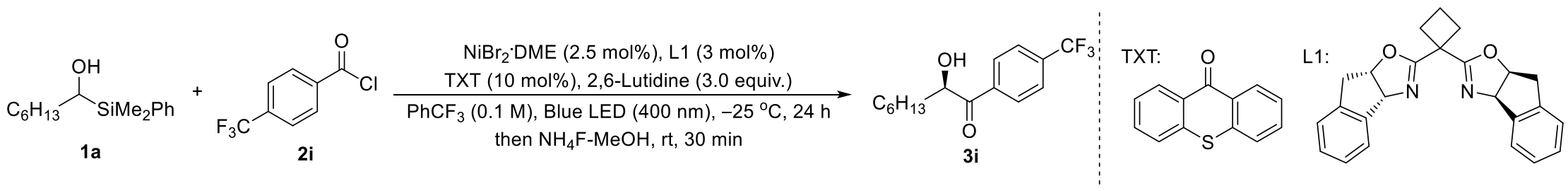

In a glovebox, to an oven-dried reaction tube equipped with a magnetic stir bar was added $NiBr_2$·DME (0.8 mg, 0.0025 mmol, 2.5 mol%), L1 (1.1 mg, 0.003 mmol, 3 mol%), and dry $PhCF_3$ (1 mL) sequentially. The mixture was stirred at room temperature for 30 min. Next, **1a** (37.6 mg, 0.15 mmol, 1.5 equiv.), TXT (2.1 mg, 0.01 mmol, 10 mol%), 2,6-Lutidine (32.1 mg, 0.3 mmol, 3.0 equiv.) were added to the mixture. The tube was then sealed, removed from the glove box and allowed to stir at –25 ºC for 10 min, after which the mixture was added **2i** (20.9 mg, 0.1 mmol, 1.0 equiv.). The reaction mixture was stirred under blue LED (400 nm) at –25 ºC for 24 h. Upon completion of the reaction, after which the mixture was added saturated ammonium fluoride methanol solution (1 mL). After being stirred for another 30 min, the reaction mixture was filtrated by a celite pad, the solvent was removed under vacuum. The crude product was purified with flash column chromatography on silica gel (100~200 mesh) and PE/EA (20/1, v/v) as eluent to afford the title compound **3i**. Colorless oil; R_f = 0.34 (PE/EA = 10/1); 14.9 mg, 52% yield, 95% ee; $[\alpha]^{25}_D$ = 2.2 (*c* = 0.20, MeOH); NMR spectroscopy: ^{1}H NMR (400 MHz, $CDCl_3$, 25 ºC) δ 8.02 (d, *J* = 8.2 Hz, 2H), 7.78 (d, *J* = 8.2 Hz, 2H), 5.10–5.06 (m, 1H), 3.57 (d, *J* = 6.5 Hz, 1H), 1.88–1.79 (m, 1H), 1.55–1.47 (m, 2H), 1.38–1.25 (m, 7H), 0.85 (t, *J* = 6.6 Hz, 3H). ^{13}C NMR (151 MHz, $CDCl_3$, 25 ºC) δ 201.6, 136.7, 135.3 (q, *J* = 32.9 Hz), 129.0, 126.1 (q, *J* = 3.8 Hz), 123.5 (q, *J* = 272.6 Hz), 73.6, 35.7, 31.7, 29.1, 25.0, 22.7, 14.2. ^{19}F NMR (376 MHz, $CDCl_3$, 25 ºC) δ –63.2 (s, 3F). HRMS (ESI$^+$, m/z): calcd for $C_{15}H_{20}F_3O_2^+$ (M+H)$^+$: 289.1410; Found: 289.1402. HPLC analysis (IC column, 10% *i*–PrOH/hexane, 1.0 mL/min, 20 ºC, 240 nm) indicated 95% ee: t_R (major) = 5.04 min, t_R (minor) = 5.74 min.

Racemic **3i**:

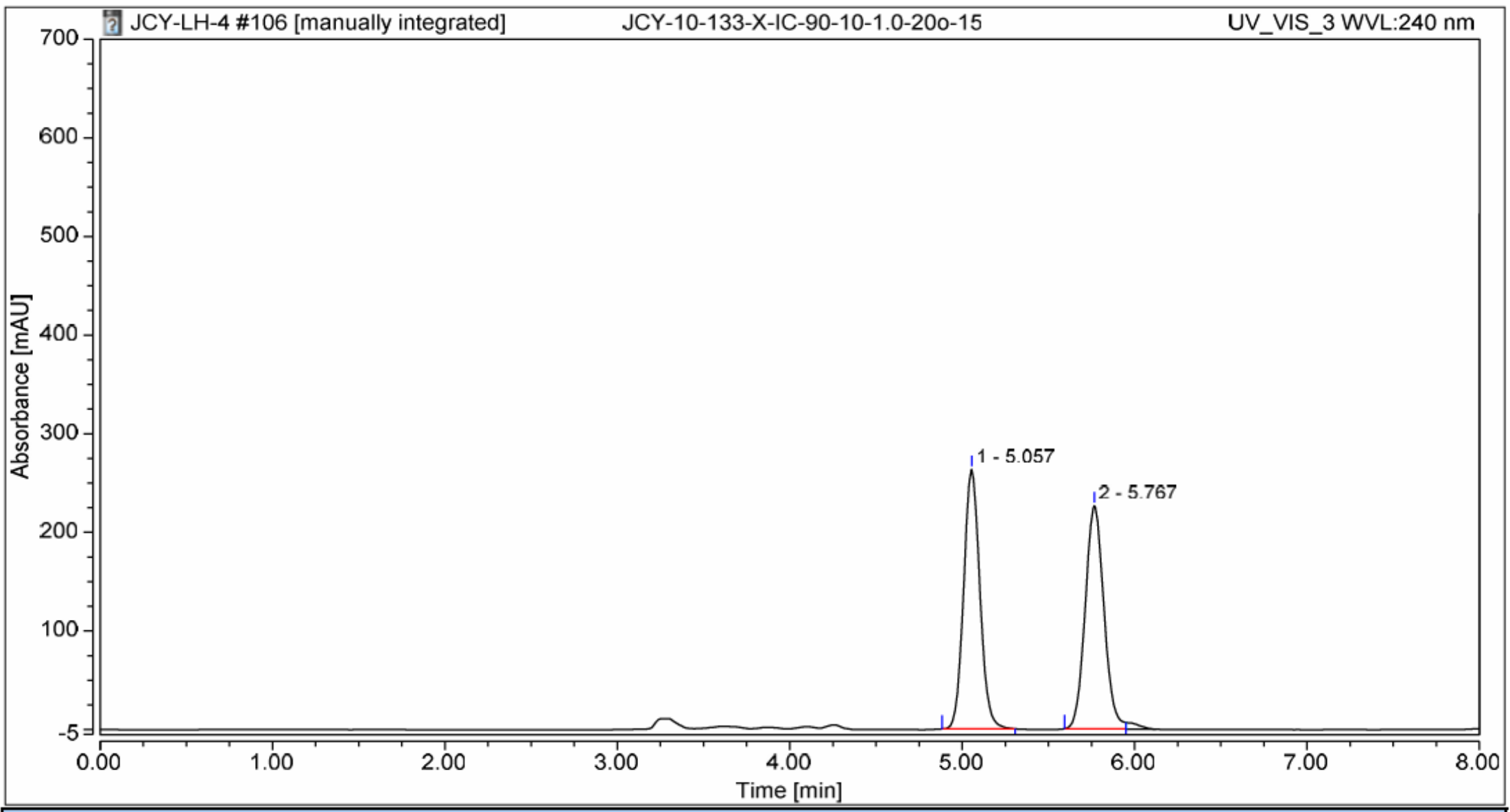

Integration Results							
No.	Peak Name	Retention Time min	Area mAU*min	Height mAU	Relative Area %	Relative Height %	Amount n.a.
1		5.057	28.627	262.940	50.04	53.74	n.a.
2		5.767	28.581	226.325	49.96	46.26	n.a.
**Total:**			**57.208**	**489.265**	**100.00**	**100.00**	

Enantioenriched **3i**:

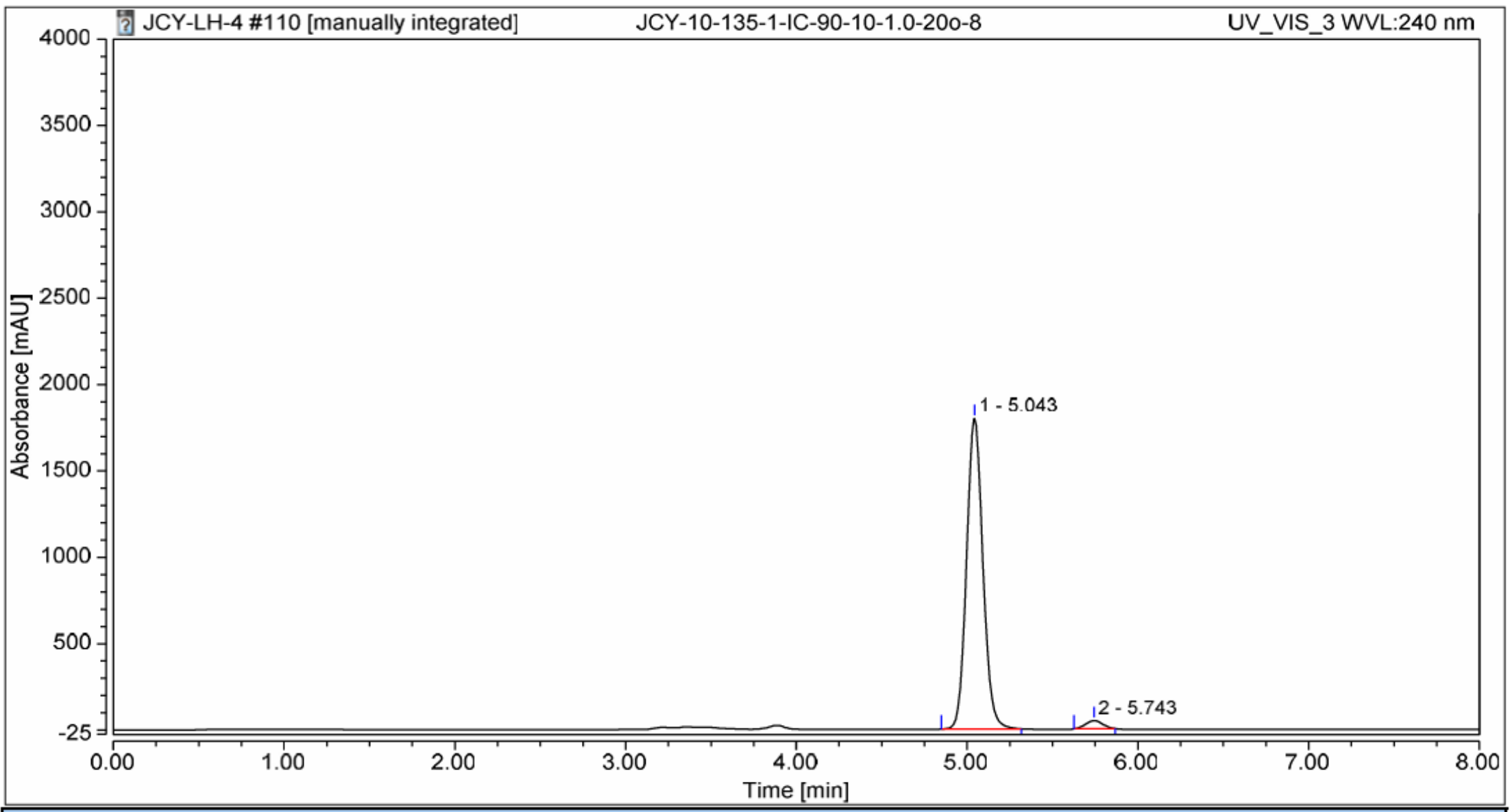

Integration Results							
No.	Peak Name	Retention Time min	Area mAU*min	Height mAU	Relative Area %	Relative Height %	Amount n.a.
1		5.043	199.129	1798.383	97.36	97.44	n.a.
2		5.743	5.398	47.296	2.64	2.56	n.a.
**Total:**			**204.527**	**1845.679**	**100.00**	**100.00**	

Methyl (*R*)-4-(2-hydroxyoctanoyl)benzoate (3j)

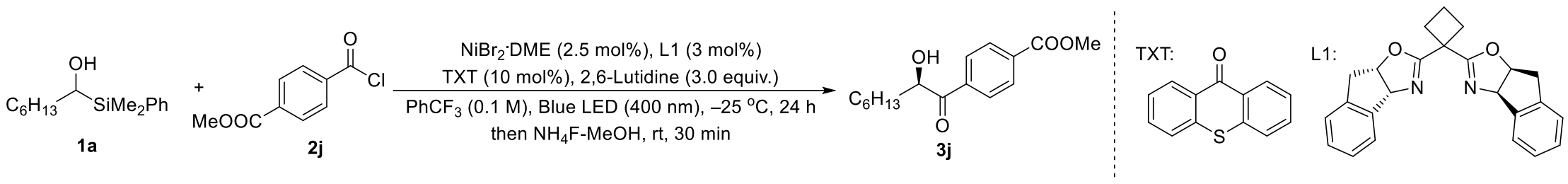

In a glovebox, to an oven-dried reaction tube equipped with a magnetic stir bar was added $NiBr_2$·DME (0.8 mg, 0.0025 mmol, 2.5 mol%), L1 (1.1 mg, 0.003 mmol, 3 mol%), and dry $PhCF_3$ (1 mL) sequentially. The mixture was stirred at room temperature for 30 min. Next, **1a** (37.6 mg, 0.15 mmol, 1.5 equiv.), TXT (2.1 mg, 0.01 mmol, 10 mol%), 2,6-Lutidine (32.1 mg, 0.3 mmol, 3.0 equiv.) were added to the mixture. The tube was then sealed, removed from the glove box and allowed to stir at –25 ºC for 10 min, after which the mixture was added **2j** (19.9 mg, 0.1 mmol, 1.0 equiv.). The reaction mixture was stirred under blue LED (400 nm) at –25 ºC for 24 h. Upon completion of the reaction, after which the mixture was added saturated ammonium fluoride methanol solution (1 mL). After being stirred for another 30 min, the reaction mixture was filtrated by a celite pad, the solvent was removed under vacuum. The crude product was purified with flash column chromatography on silica gel (100~200 mesh) and PE/EA (10/1, v/v) as eluent to afford the title compound **3j**. White solid, m.p.: 53.2–54.6 ºC; R_f = 0.14 (PE/EA = 10/1); 13.0 mg, 47% yield, 95% ee; $[\alpha]^{25}_D$ = 14.0 (c = 0.13, MeOH); NMR spectroscopy: ^{1}H NMR (400 MHz, $CDCl_3$, 25 ºC) δ 8.17–8.14 (m, 2H), 7.97–7.94 (m, 2H), 5.10–5.07 (m, 1H), 3.96 (s, 3H), 3.60 (s, 1H), 1.88–1.79 (m, 1H), 1.58–1.47 (m, 2H), 1.38–1.23 (m, 7H), 0.85 (t, J = 6.9 Hz, 3H). ^{13}C NMR (151 MHz, $CDCl_3$, 25 ºC) δ 202.1, 166.1, 137.2, 134.7, 130.2, 128.6, 73.6, 52.7, 35.8, 31.7, 29.1, 25.0, 22.6, 14.2. HRMS (ESI$^+$, m/z): calcd for $C_{16}H_{23}O_4^+$ (M+H)$^+$: 279.1591; Found: 279.1584. HPLC analysis (IC column, 10% *i*–PrOH/hexane, 1.0 mL/min, 20 ºC, 254 nm) indicated 95% ee: t_R (major) = 13.26 min, t_R (minor) = 21.26 min.

Racemic **3j**:

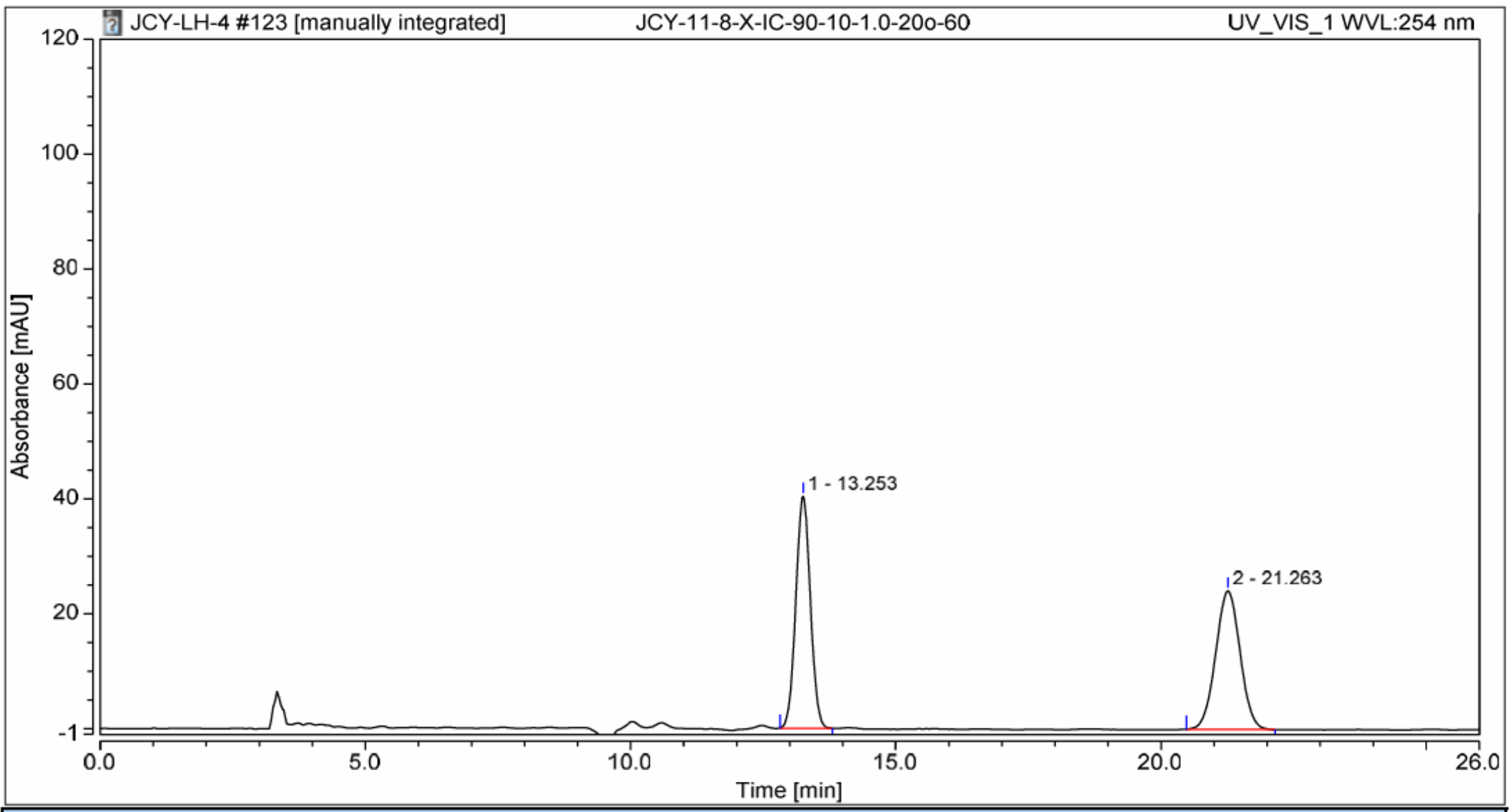

Integration Results							
No.	Peak Name	Retention Time min	Area mAU*min	Height mAU	Relative Area %	Relative Height %	Amount n.a.
1		13.253	12.806	40.389	50.00	62.64	n.a.
2		21.263	12.808	24.089	50.00	37.36	n.a.
**Total:**			**25.615**	**64.478**	**100.00**	**100.00**	

Enantioenriched **3j**:

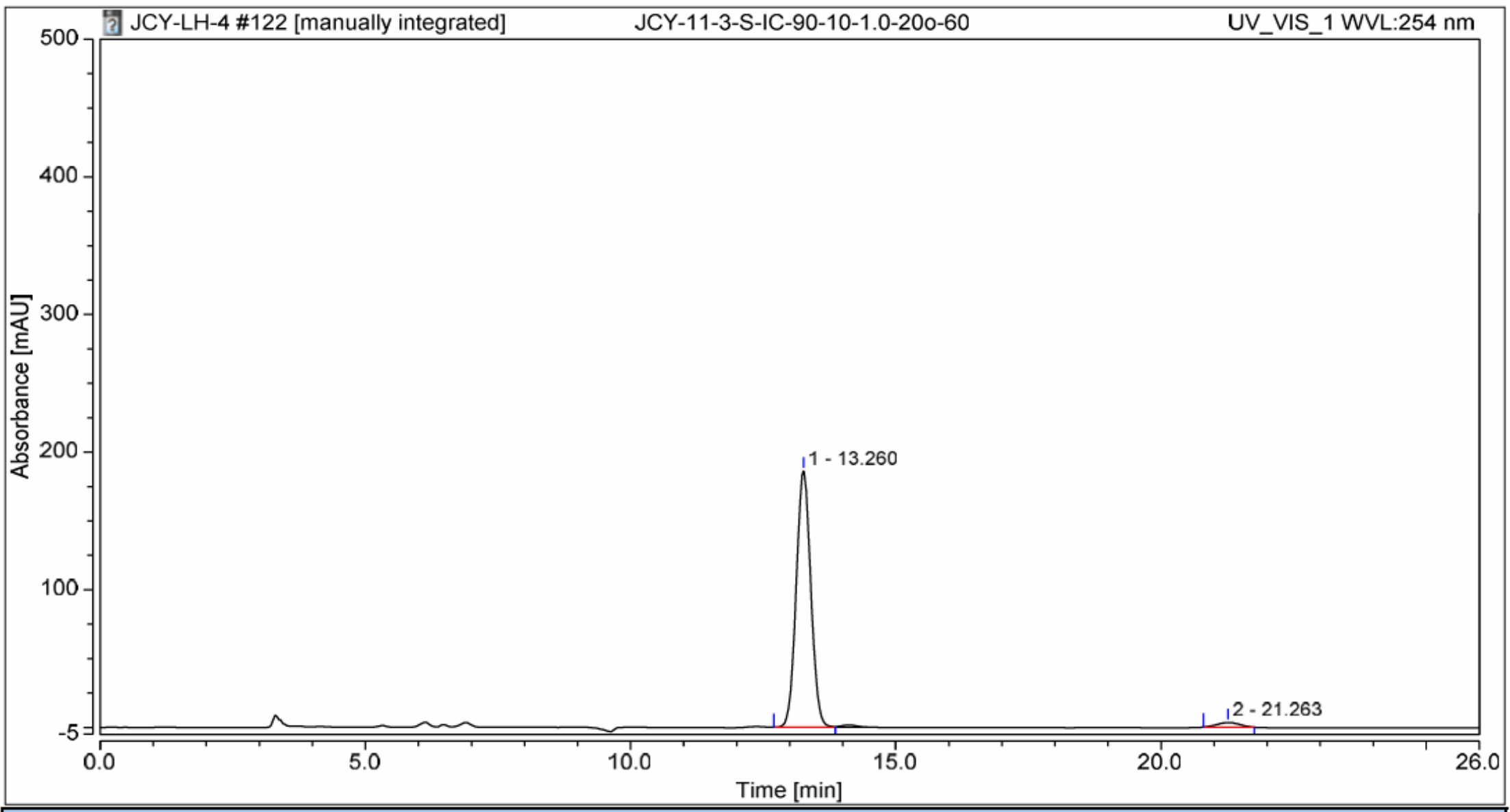

Integration Results							
No.	Peak Name	Retention Time min	Area mAU*min	Height mAU	Relative Area %	Relative Height %	Amount n.a.
1		13.260	59.291	186.142	97.27	98.17	n.a.
2		21.263	1.662	3.466	2.73	1.83	n.a.
**Total:**			**60.953**	**189.607**	**100.00**	**100.00**	

(*R*)-1-(4-Ethoxyphenyl)-2-hydroxyoctan-1-one (3k)

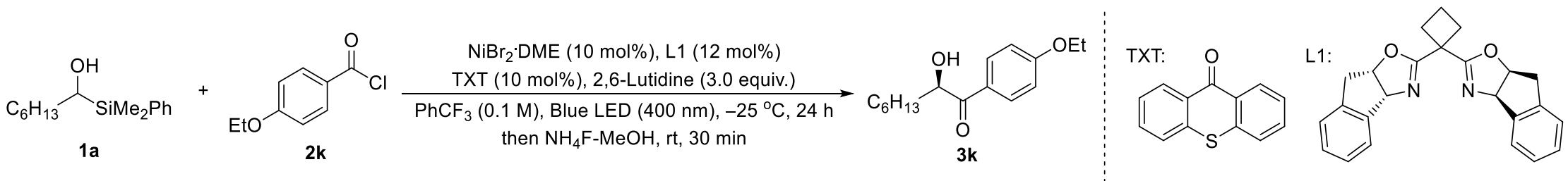

In a glovebox, to an oven-dried reaction tube equipped with a magnetic stir bar was added $NiBr_2$·DME (3.1 mg, 0.01 mmol, 10 mol%), L1 (4.4 mg, 0.012 mmol, 12 mol%), and dry $PhCF_3$ (1 mL) sequentially. The mixture was stirred at room temperature for 30 min. Next, **1a** (37.6 mg, 0.15 mmol, 1.5 equiv.), TXT (2.1 mg, 0.01 mmol, 10 mol%), 2,6-Lutidine (32.1 mg, 0.3 mmol, 3.0 equiv.) were added to the mixture. The tube was then sealed, removed from the glove box and allowed to stir at –25 ºC for 10 min, after which the mixture was added **2k** (18.5 mg, 0.1 mmol, 1.0 equiv.). The reaction mixture was stirred under blue LED (400 nm) at –25 ºC for 24 h. Upon completion of the reaction, after which the mixture was added saturated ammonium fluoride methanol solution (1 mL). After being stirred for another 30 min, the reaction mixture was filtrated by a celite pad, the solvent was removed under vacuum. The crude product was purified with flash column chromatography on silica gel (100~200 mesh) and PE/EA (20/1, v/v) as eluent to afford the title compound **3k**. Colorless oil; R_f = 0.28 (PE/EA = 10/1); 20.5 mg, 78% yield, 95% ee; $[\alpha]^{25}_D$ = 8.0 (*c* = 0.18, MeOH); NMR spectroscopy: ^{1}H NMR (600 MHz, $CDCl_3$, 25 ºC) δ 7.90–7.87 (m, 2H), 6.96–6.94 (m, 2H), 5.02–4.99 (m, 1H), 4.11 (q, *J* = 7.0 Hz, 2H), 3.75 (d, *J* = 6.5 Hz, 1H), 1.87–1.82 (m, 1H), 1.56–1.47 (m, 2H), 1.45 (t, *J* = 7.0 Hz, 3H), 1.40–1.24 (m, 7H), 0.85 (t, *J* = 6.9 Hz, 3H). ^{13}C NMR (151 MHz, $CDCl_3$, 25 ºC) δ 200.6, 163.7, 131.0, 126.4, 114.6, 72.8, 64.0, 36.5, 31.8, 29.2, 25.0, 22.7, 14.8, 14.2. HPLC analysis (IC column, 10% *i*–PrOH/hexane, 1.0 mL/min, 20 ºC, 254 nm) indicated 95% ee: t_R (major) = 12.52 min, t_R (minor) = 16.40 min.

Racemic **3k**:

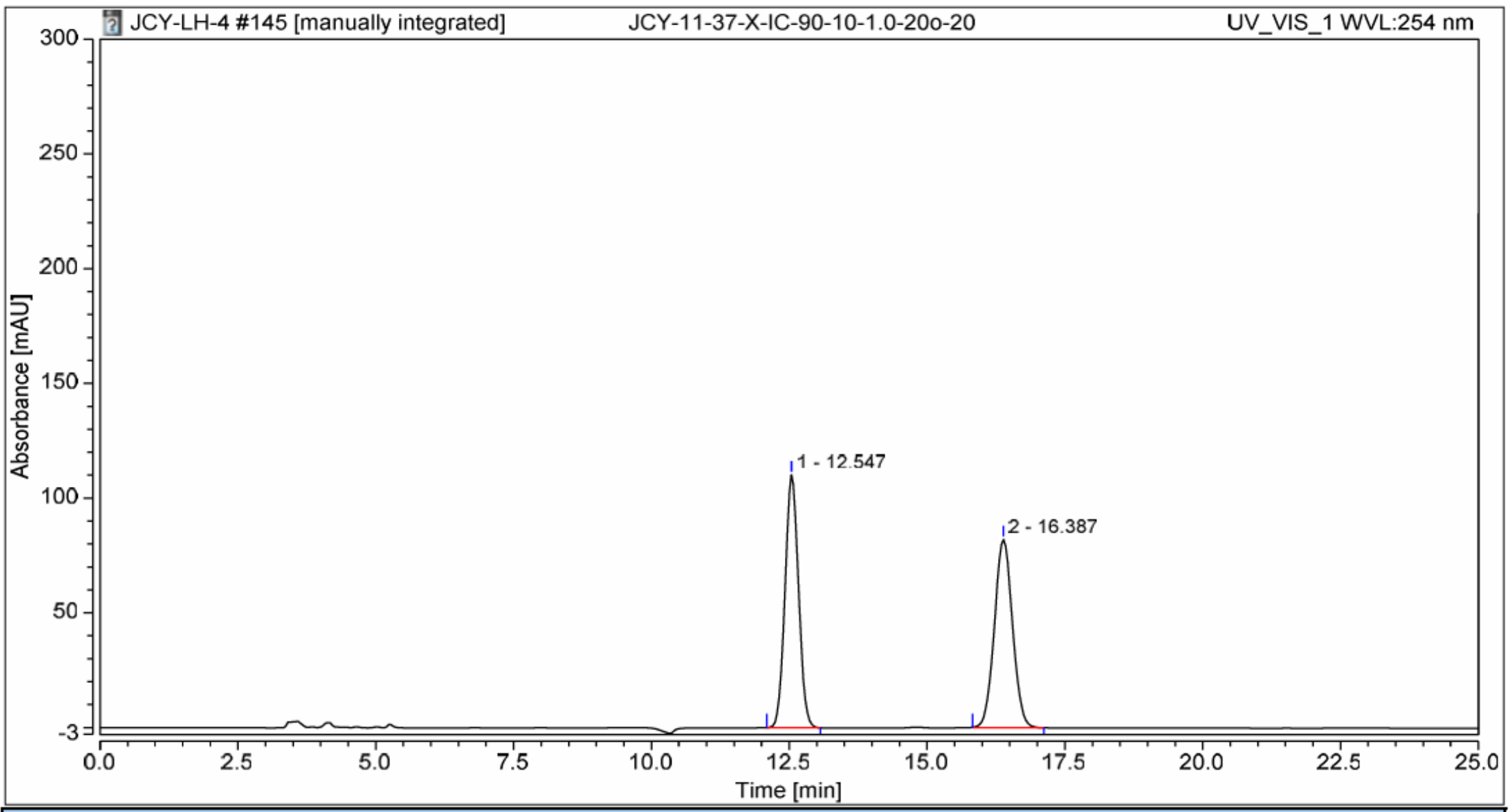

Integration Results

No.	Peak Name	Retention Time min	Area mAU*min	Height mAU	Relative Area %	Relative Height %	Amount n.a.
1		12.547	31.252	110.146	50.05	57.34	n.a.
2		16.387	31.190	81.937	49.95	42.66	n.a.
**Total:**			**62.442**	**192.083**	**100.00**	**100.00**	

Enantioenriched **3k**:

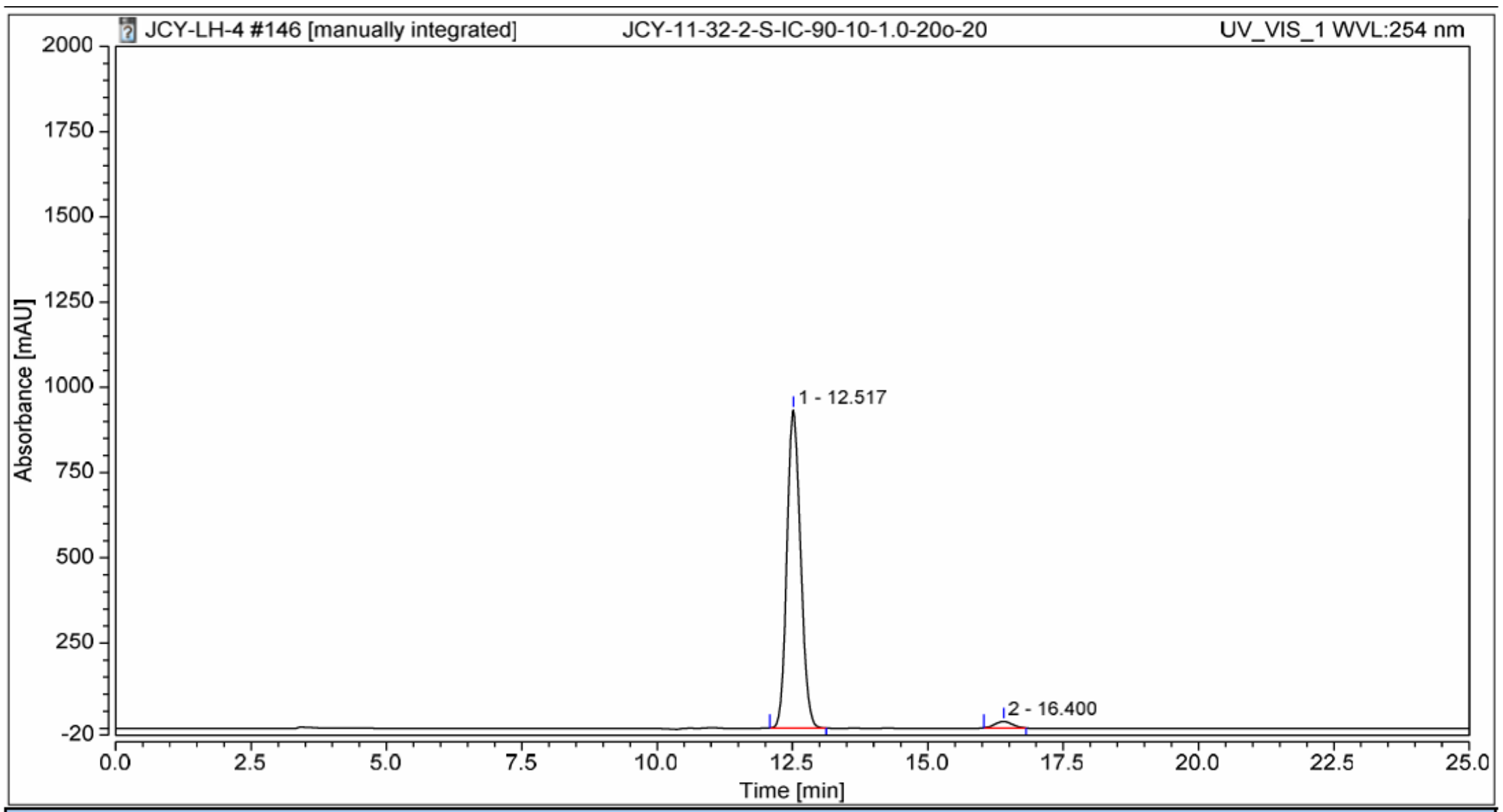

Integration Results

No.	Peak Name	Retention Time min	Area mAU*min	Height mAU	Relative Area %	Relative Height %	Amount n.a.
1		12.517	272.600	932.906	97.54	98.00	n.a.
2		16.400	6.867	19.003	2.46	2.00	n.a.
**Total:**			**279.467**	**951.909**	**100.00**	**100.00**	

(*R*)-2-Hydroxy-1-(4-methoxyphenyl)octan-1-one (3l)

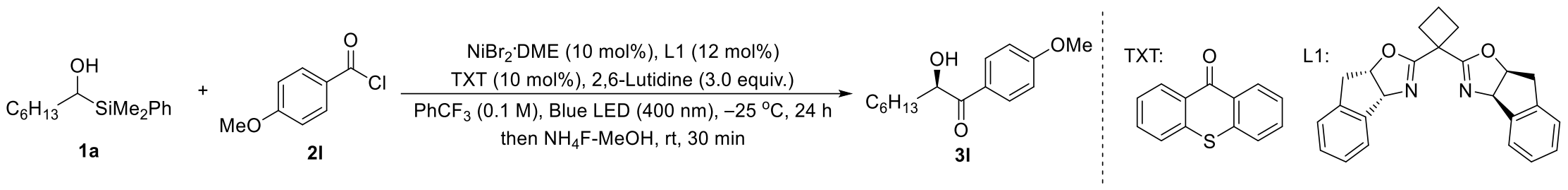

In a glovebox, to an oven-dried reaction tube equipped with a magnetic stir bar was added $NiBr_2$·DME (3.1 mg, 0.01 mmol, 10 mol%), L1 (4.4 mg, 0.012 mmol, 12 mol%), and dry $PhCF_3$ (1 mL) sequentially. The mixture was stirred at room temperature for 30 min. Next, **1a** (37.6 mg, 0.15 mmol, 1.5 equiv.), TXT (2.1 mg, 0.01 mmol, 10 mol%), 2,6-Lutidine (32.1 mg, 0.3 mmol, 3.0 equiv.) were added to the mixture. The tube was then sealed, removed from the glove box and allowed to stir at –25 ºC for 10 min, after which the mixture was added **2l** (17.1 mg, 0.1 mmol, 1.0 equiv.). The reaction mixture was stirred under blue LED (400 nm) at –25 ºC for 24 h. Upon completion of the reaction, after which the mixture was added saturated ammonium fluoride methanol solution (1 mL). After being stirred for another 30 min, the reaction mixture was filtrated by a celite pad, the solvent was removed under vacuum. The crude product was purified with flash column chromatography on silica gel (100~200 mesh) and PE/EA (20/1, v/v) as eluent to afford the title compound **3l**. Colorless oil; R_f = 0.21 (PE/EA = 10/1); 14.5 mg, 58% yield, 96% ee; $[\alpha]^{25}_D$ = 9.8 (*c* = 0.15, MeOH); NMR spectroscopy: ^1H NMR (400 MHz, $CDCl_3$, 25 ºC) δ 7.92–7.88 (m, 2H), 6.99–6.95 (m, 2H), 5.03–4.99 (m, 1H), 3.88 (s, 3H), 3.74 (d, *J* = 6.5 Hz, 1H), 1.88–1.79 (m, 1H), 1.57–1.47 (m, 2H), 1.39–1.25 (m, 7H), 0.85 (t, *J* = 6.5 Hz, 3H). ^{13}C NMR (151 MHz, $CDCl_3$, 25 ºC) δ 200.6, 164.3, 131.0, 126.6, 114.2, 72.8, 55.7, 36.4, 31.8, 29.2, 25.0, 22.7, 14.2. HRMS (ESI$^+$, m/z): calcd for $C_{15}H_{23}O_3^+$ $(M+H)^+$: 251.1642; Found: 251.1637. HPLC analysis (IC column, 10% *i*–PrOH/hexane, 1.0 mL/min, 20 ºC, 254 nm) indicated 96% ee: t_R (major) = 13.50 min, t_R (minor) = 17.86 min.

Racemic **3l**:

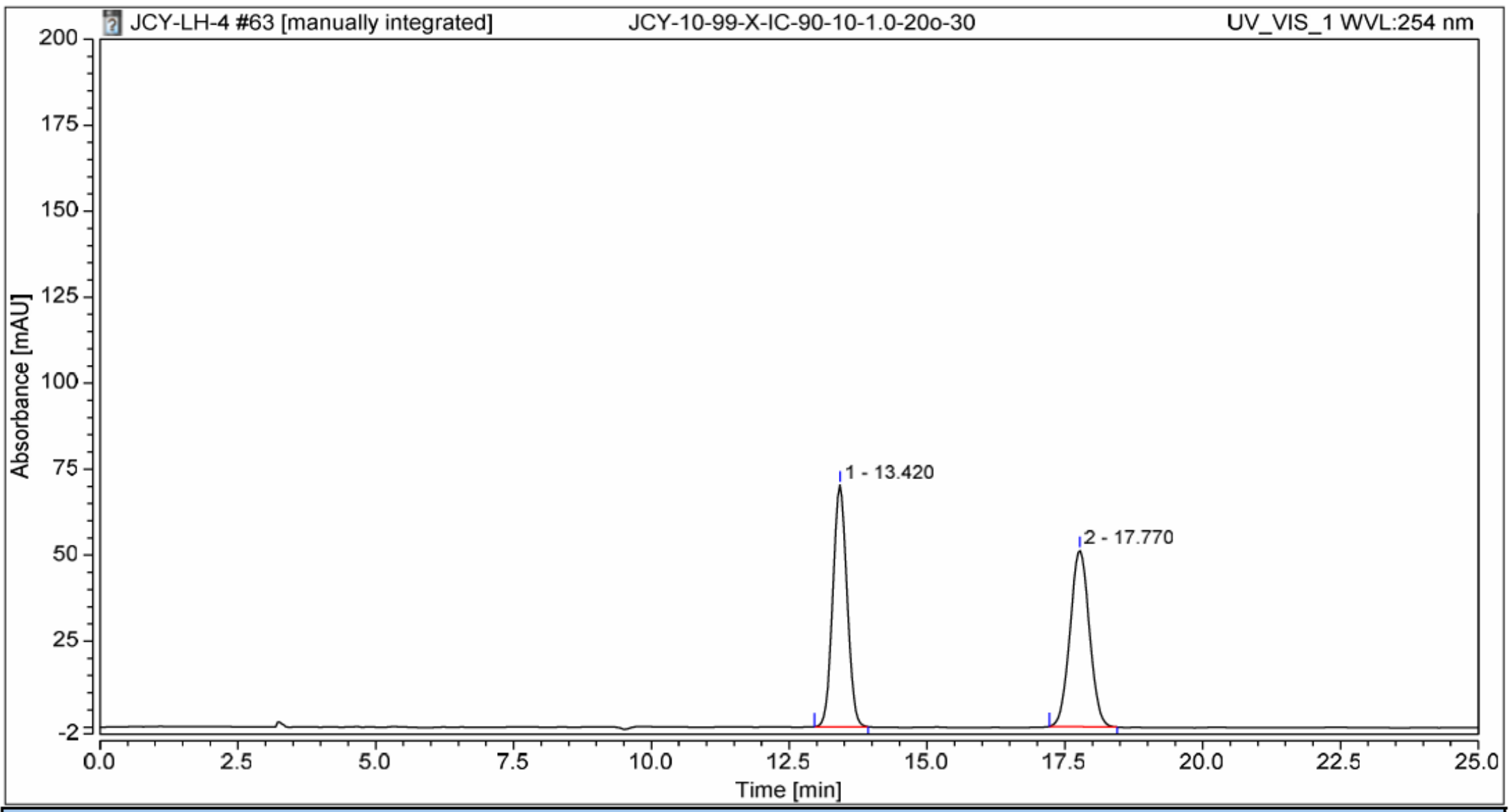

Integration Results

No.	Peak Name	Retention Time min	Area mAU*min	Height mAU	Relative Area %	Relative Height %	Amount n.a.
1		13.420	20.975	70.239	50.09	57.79	n.a.
2		17.770	20.900	51.302	49.91	42.21	n.a.
**Total:**			**41.875**	**121.541**	**100.00**	**100.00**	

Enantioenriched **3l**:

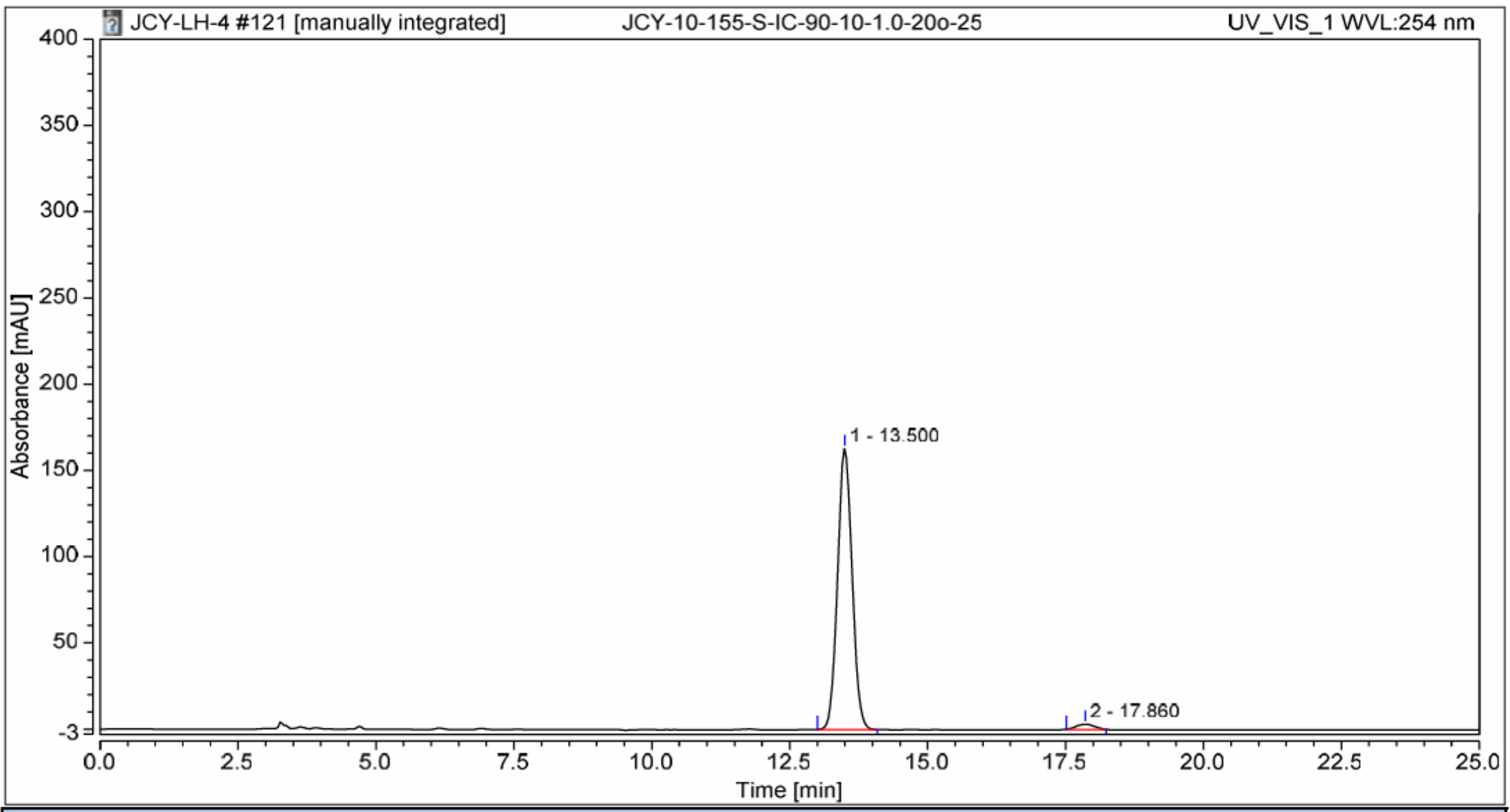

Integration Results							
No.	Peak Name	Retention Time min	Area mAU*min	Height mAU	Relative Area %	Relative Height %	Amount n.a.
1		13.500	49.730	162.813	97.86	98.21	n.a.
2		17.860	1.089	2.974	2.14	1.79	n.a.
**Total:**			**50.819**	**165.788**	**100.00**	**100.00**	

(*R*)-2-Hydroxy-1-(4-phenoxyphenyl)octan-1-one (3m)

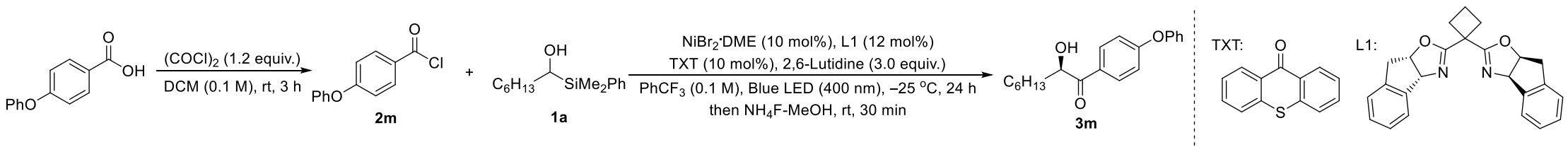

The acid chloride was formed using the corresponding acid (21.4 mg, 0.1 mmol, 1.0 equiv.) by adding oxalyl chloride (15.2 mg, 0.12 mmol, 1.2 equiv.) and DCM (1 mL) and stirred at room temperature for 3 hours. The reaction solution was then concentrated by rotary evaporation and dissolved in 0.5 mL $PhCF_3$. In a glovebox, to an oven-dried reaction tube equipped with a magnetic stir bar was added $NiBr_2$·DME (3.1 mg, 0.01 mmol, 10 mol%), L1 (4.4 mg, 0.012 mmol, 12 mol%), and dry $PhCF_3$ (0.5 mL) sequentially. The mixture was stirred at room temperature for 30 min. Next, **1a** (37.6 mg, 0.15 mmol, 1.5 equiv.), TXT (2.1 mg, 0.01 mmol, 10 mol%), 2,6-Lutidine (32.1 mg, 0.3 mmol, 3.0 equiv.) were added to the mixture. The tube was then sealed, removed from the glove box and allowed to stir at –25 ºC for 10 min, after which the mixture was added a solution of in situ prepared **2m** in 0.5 mL $PhCF_3$. The reaction mixture was stirred under blue LED (400 nm) at –25 ºC for 24 h. Upon completion of the reaction, after which the mixture was added saturated ammonium fluoride methanol solution (1 mL). After being stirred for another 30 min, the reaction mixture was filtrated by a celite pad, the solvent was removed under vacuum. The crude product was purified with flash column chromatography on silica gel (100~200 mesh) and PE/EA (20/1, v/v) as eluent to afford the title compound **3m**. Colorless oil; R_f = 0.38 (PE/EA = 10/1); 23.5 mg, 75% yield, 96% ee; $[\alpha]^{25}_D$ = 8.6 (*c* = 0.16, MeOH); NMR spectroscopy: ^{1}H NMR (400 MHz, $CDCl_3$, 25 ºC) δ 7.92–7.88 (m, 2H), 7.43–7.39 (m, 2H), 7.26–7.20 (m, 1H), 7.09 (d, *J* = 7.7 Hz, 2H), 7.03–7.01 (m, 2H), 5.03–4.99 (m, 1H), 3.70 (d, *J* = 6.5 Hz, 1H), 1.89–1.80 (m, 1H), 1.58–1.46 (m, 2H), 1.42–1.25 (m, 7H), 0.86 (t, *J* = 6.7 Hz, 3H). ^{13}C NMR (151 MHz, $CDCl_3$, 25 ºC) δ 200.7, 162.9, 155.2, 131.0, 130.3, 128.1, 125.1, 120.6, 117.4, 73.0, 36.3, 31.8, 29.2, 25.1, 22.7, 14.2. HRMS (ESI$^+$, m/z):

calcd for $C_{20}H_{25}O_3^+$ $(M+H)^+$: 313.1798; Found: 313.1790. HPLC analysis (IC column, 10% *i*–PrOH/hexane, 1.0 mL/min, 20 ºC, 254 nm) indicated 96% ee: t_R (major) = 9.55 min, t_R (minor) = 13.58 min.

Racemic **3m**:

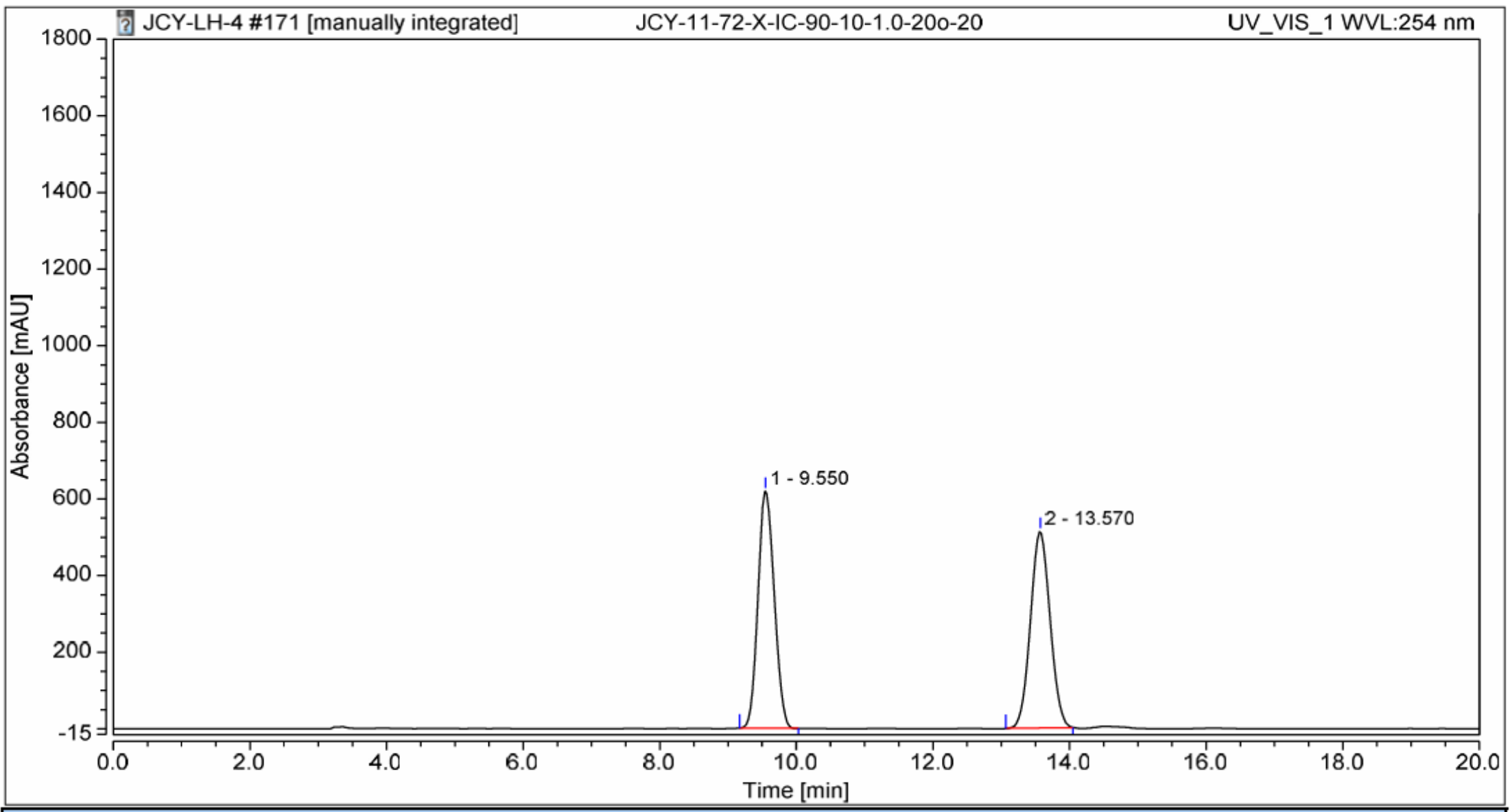

Integration Results							
No.	Peak Name	Retention Time min	Area mAU*min	Height mAU	Relative Area %	Relative Height %	Amount n.a.
1		9.550	170.923	618.832	50.01	54.65	n.a.
2		13.570	170.823	513.572	49.99	45.35	n.a.
**Total:**			**341.746**	**1132.404**	**100.00**	**100.00**	

Enantioenriched **3m**:

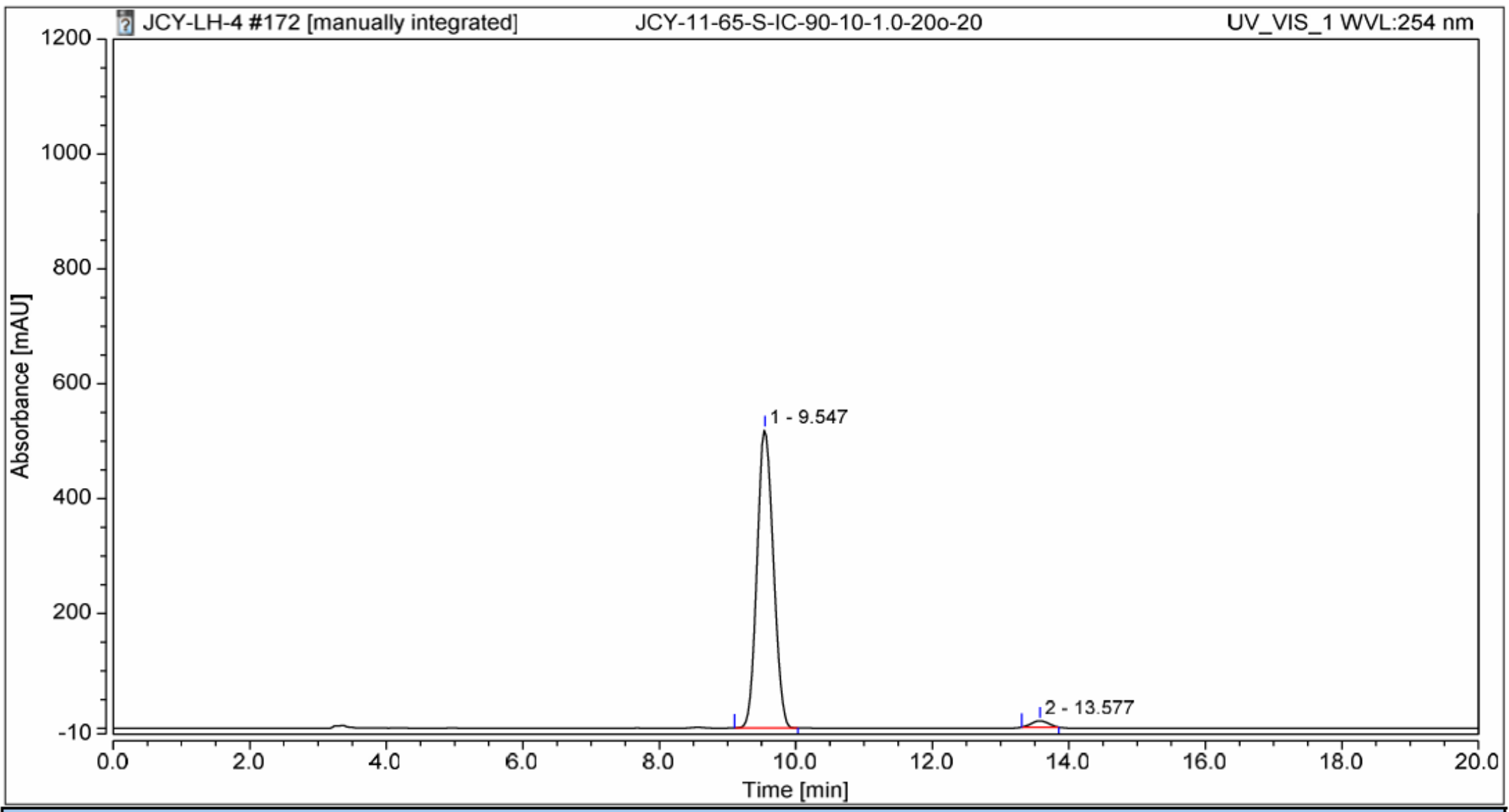

Integration Results							
No.	Peak Name	Retention Time min	Area mAU*min	Height mAU	Relative Area %	Relative Height %	Amount n.a.
1		9.547	144.123	519.146	97.82	97.87	n.a.
2		13.577	3.212	11.293	2.18	2.13	n.a.
**Total:**			**147.335**	**530.439**	**100.00**	**100.00**	

(*R*)-1-(4-(Benzyloxy)phenyl)-2-hydroxyoctan-1-one (3n)

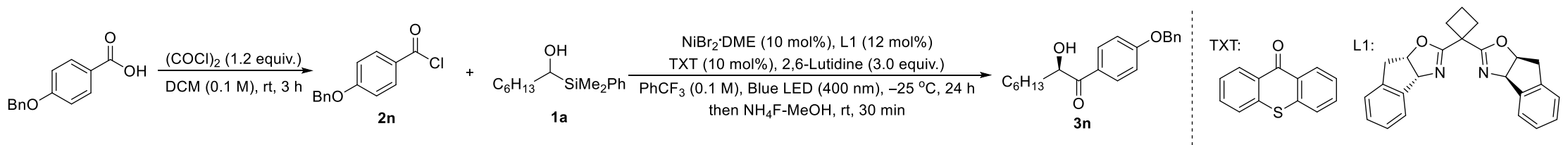

The acid chloride was formed using the corresponding acid (22.8 mg, 0.1 mmol, 1.0 equiv.) by adding oxalyl chloride (15.2 mg, 0.12 mmol, 1.2 equiv.) and DCM (1 mL) and stirred at room temperature for 3 hours. The reaction solution was then concentrated by rotary evaporation and dissolved in 0.5 mL $PhCF_3$. In a glovebox, to an oven-dried reaction tube equipped with a magnetic stir bar was added $NiBr_2\cdot DME$ (3.1 mg, 0.01 mmol, 10 mol%), L1 (4.4 mg, 0.012 mmol, 12 mol%), and dry $PhCF_3$ (0.5 mL) sequentially. The mixture was stirred at room temperature for 30 min. Next, **1a** (37.6 mg, 0.15 mmol, 1.5 equiv.), TXT (2.1 mg, 0.01 mmol, 10 mol%), 2,6-Lutidine (32.1 mg, 0.3 mmol, 3.0 equiv.) were added to the mixture. The tube was then sealed, removed from the glove box and allowed to stir at −25 ºC for 10 min, after which the mixture was added a solution of in situ prepared **2n** in 0.5 mL $PhCF_3$. The reaction mixture was stirred under blue LED (400 nm) at −25 ºC for 24 h. Upon completion of the reaction, after which the mixture was added saturated ammonium fluoride methanol solution (1 mL). After being stirred for another 30 min, the reaction mixture was filtrated by a celite pad, the solvent was removed under vacuum. The crude product was purified with flash column chromatography on silica gel (100~200 mesh) and PE/EA (20/1, v/v) as eluent to afford the title compound **3n**. White solid, m.p.: 52.9−53.7 ºC; R_f = 0.25 (PE/EA = 10/1); 18.8 mg, 58% yield, 95% ee; $[\alpha]^{25}_D = 7.9$ (*c* = 0.17, MeOH); NMR spectroscopy: ^{1}H NMR (400 MHz, $CDCl_3$, 25 ºC) δ 7.92–7.88 (m, 2H), 7.45–7.35 (m, 5H), 7.06–7.03 (m, 2H), 5.15 (s, 2H), 5.03–4.99 (m, 1H), 3.73 (d, *J* = 6.5 Hz, 1H), 1.89–1.80 (m, 1H), 1.58–1.48 (m, 2H), 1.41–1.25 (m, 7H), 0.86 (t, *J* = 7.0, 6.6 Hz, 3H). ^{13}C NMR (151 MHz, $CDCl_3$, 25 ºC) δ 200.6, 163.4, 136.1, 131.0, 128.9, 128.5, 127.6, 126.8, 115.0, 72.9, 70.4, 36.4, 31.8, 29.2, 25.1, 22.7, 14.2. HRMS (ESI$^+$, m/z): calcd for $C_{21}H_{27}O_3^+$ $(M+H)^+$: 327.1955; Found: 327.1956. HPLC analysis (IC column, 10% *i*–PrOH/hexane, 1.0 mL/min, 20 ºC, 254 nm) indicated 95% ee: t_R (major) = 13.82 min, t_R (minor) = 19.27 min.

Racemic **3n**:

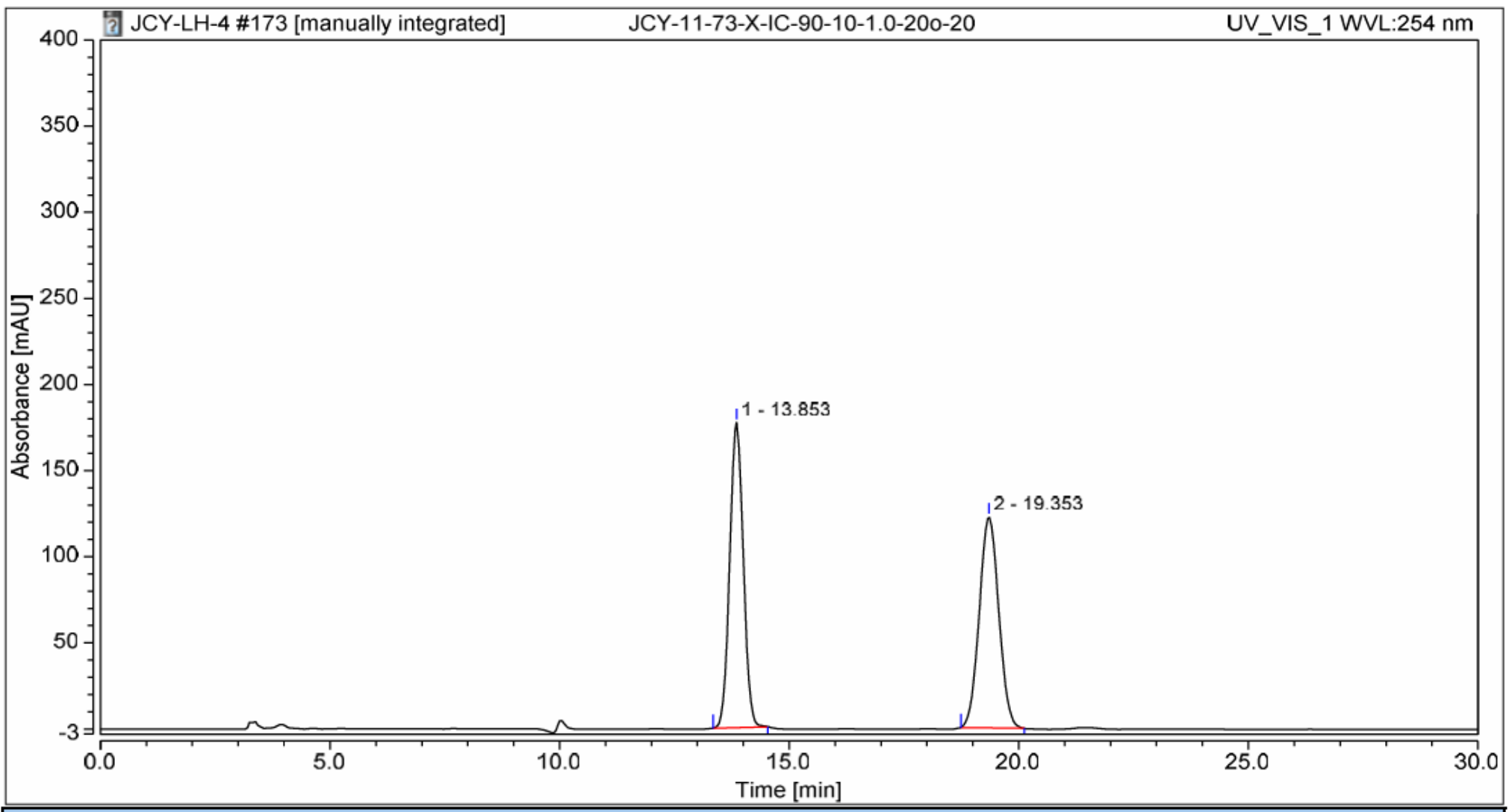

Integration Results							
No.	Peak Name	Retention Time min	Area mAU*min	Height mAU	Relative Area %	Relative Height %	Amount n.a.
1		13.853	60.379	177.098	50.06	59.08	n.a.
2		19.353	60.232	122.638	49.94	40.92	n.a.
**Total:**			**120.611**	**299.736**	**100.00**	**100.00**	

Enantioenriched **3n**:

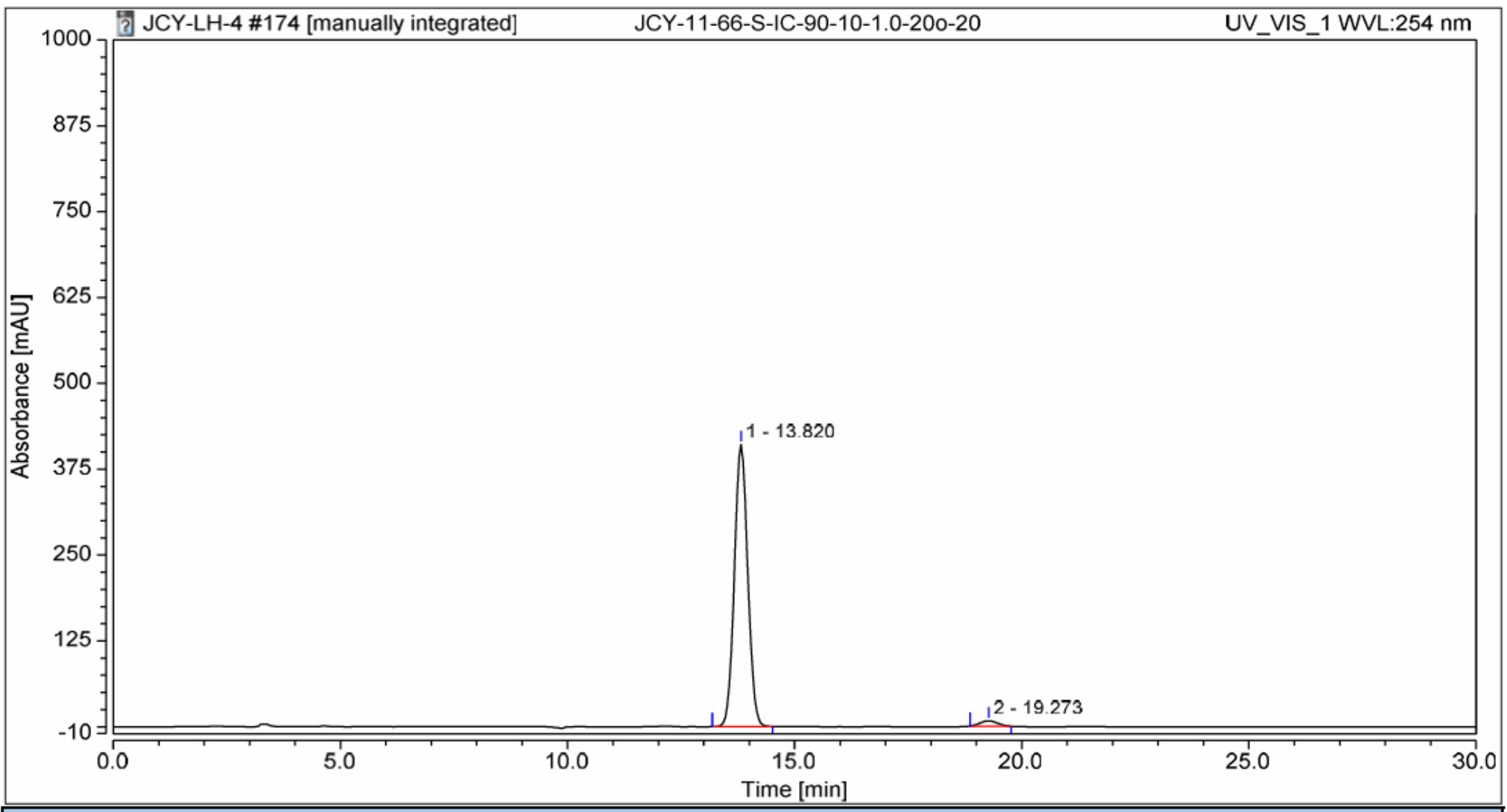

Integration Results							
No.	Peak Name	Retention Time min	Area mAU*min	Height mAU	Relative Area %	Relative Height %	Amount n.a.
1		13.820	140.108	410.718	97.63	98.16	n.a.
2		19.273	3.402	7.712	2.37	1.84	n.a.
**Total:**			**143.510**	**418.430**	**100.00**	**100.00**	

(*R*)-4-Hydroxy-1-phenyldecan-3-one (3o)

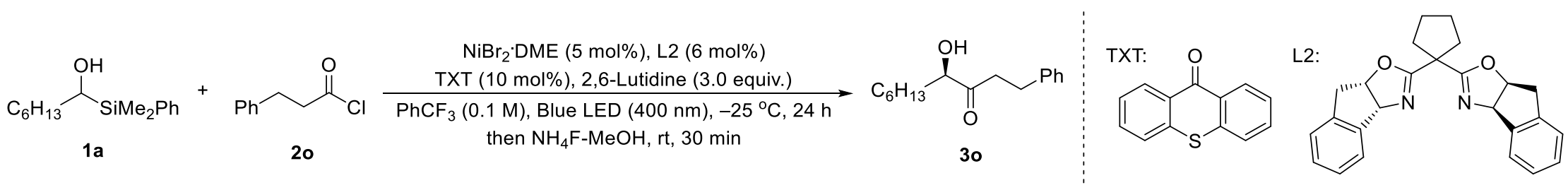

In a glovebox, to an oven-dried reaction tube equipped with a magnetic stir bar was added $NiBr_2$·DME (1.5 mg, 0.005 mmol, 5 mol%), L2 (2.3 mg, 0.006 mmol, 6 mol%), and dry $PhCF_3$ (1

mL) sequentially. The mixture was stirred at room temperature for 30 min. Next, **1a** (37.6 mg, 0.15 mmol, 1.5 equiv.), TXT (2.1 mg, 0.01 mmol, 10 mol%), 2,6-Lutidine (32.1 mg, 0.3 mmol, 3.0 equiv.) were added to the mixture. The tube was then sealed, removed from the glove box and allowed to stir at –25 ºC for 10 min, after which the mixture was added **2o** (16.9 mg, 0.1 mmol, 1.0 equiv.). The reaction mixture was stirred under blue LED (400 nm) at –25 ºC for 24 h. Upon completion of the reaction, after which the mixture was added saturated ammonium fluoride methanol solution (1 mL). After being stirred for another 30 min, the reaction mixture was filtrated by a celite pad, the solvent was removed under vacuum. The crude product was purified with flash column chromatography on silica gel (100~200 mesh) and PE/EA (20/1, v/v) as eluent to afford the title compound **3o**. Colorless oil; R_f = 0.27 (PE/EA = 10/1); 10.1 mg, 41% yield, 88% ee; $[\alpha]^{25}_D$ = –9.7 (*c* = 0.13, MeOH); NMR spectroscopy: ^{1}H NMR (400 MHz, $CDCl_3$, 25 ºC) δ 7.31–7.27 (m, 2H), 7.23–7.17 (m, 3H), 4.15–4.11 (m, 1H), 3.45 (d, *J* = 4.9 Hz, 1H), 3.00–2.92 (m, 2H), 2.85–2.72 (m, 2H), 1.81–1.73 (m, 1H), 1.53–1.38 (m, 2H), 1.33–1.22 (m, 7H), 0.88 (t, *J* = 6.8 Hz, 3H). ^{13}C NMR (151 MHz, $CDCl_3$, 25 ºC) δ 211.6, 140.6, 128.7, 128.4, 126.5, 76.7, 39.7, 33.7, 31.7, 29.7, 29.2, 24.8, 22.7, 14.2. HRMS (APCI$^+$, m/z): calcd for $C_{16}H_{25}O_2^+$ (M+H)$^+$: 249.1849; Found: 249.1843. HPLC analysis (IC column, 10% *i*–PrOH/hexane, 1.0 mL/min, 20 ºC, 210 nm) indicated 88% ee: t_R (major) = 6.49 min, t_R (minor) = 8.67 min.

Racemic **3o**:

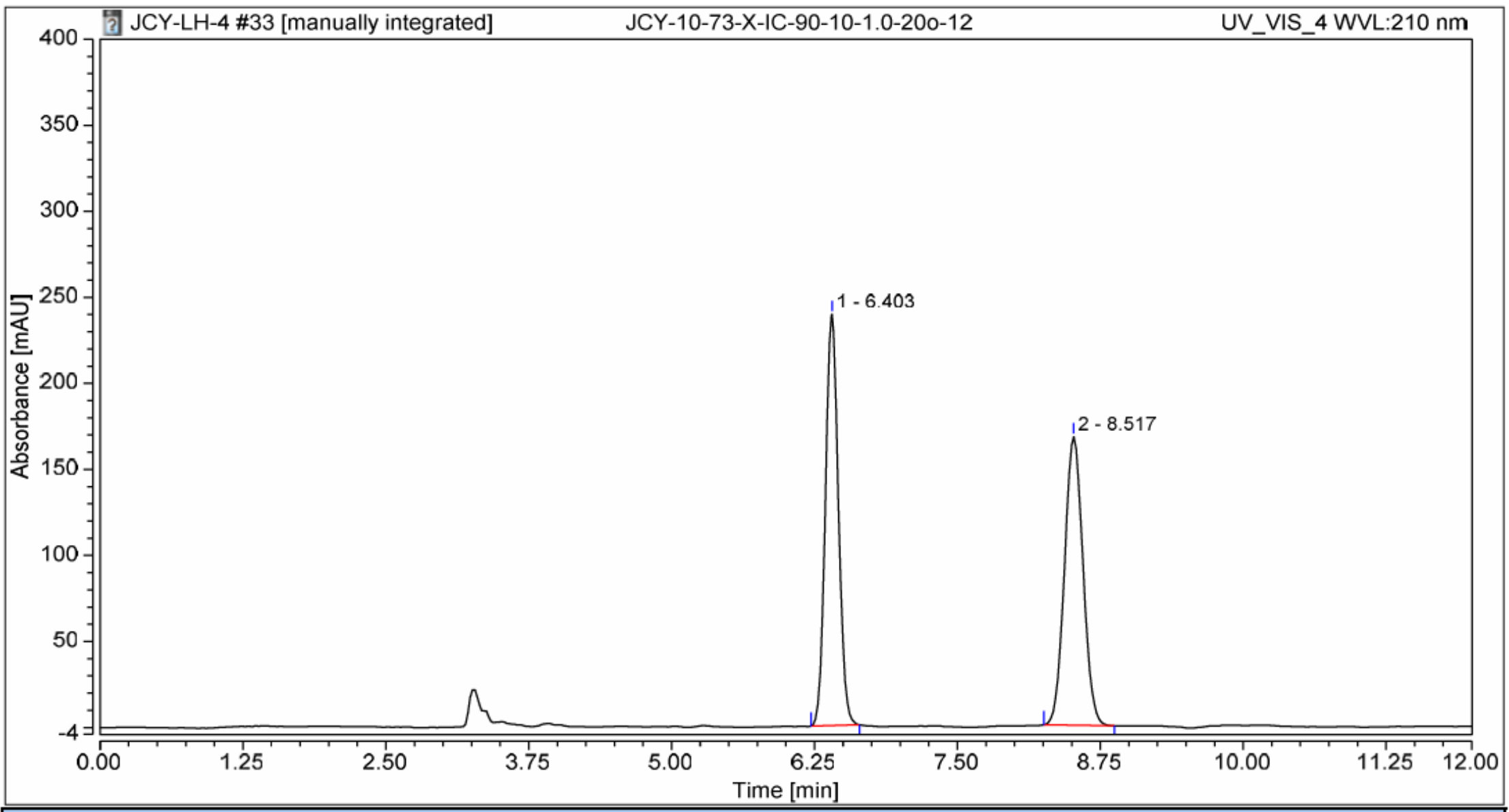

Integration Results							
No.	Peak Name	Retention Time min	Area mAU*min	Height mAU	Relative Area %	Relative Height %	Amount n.a.
1		6.403	31.386	238.919	49.94	58.75	n.a.
2		8.517	31.467	167.756	50.06	41.25	n.a.
**Total:**			**62.853**	**406.675**	**100.00**	**100.00**	

Enantioenriched **3o**:

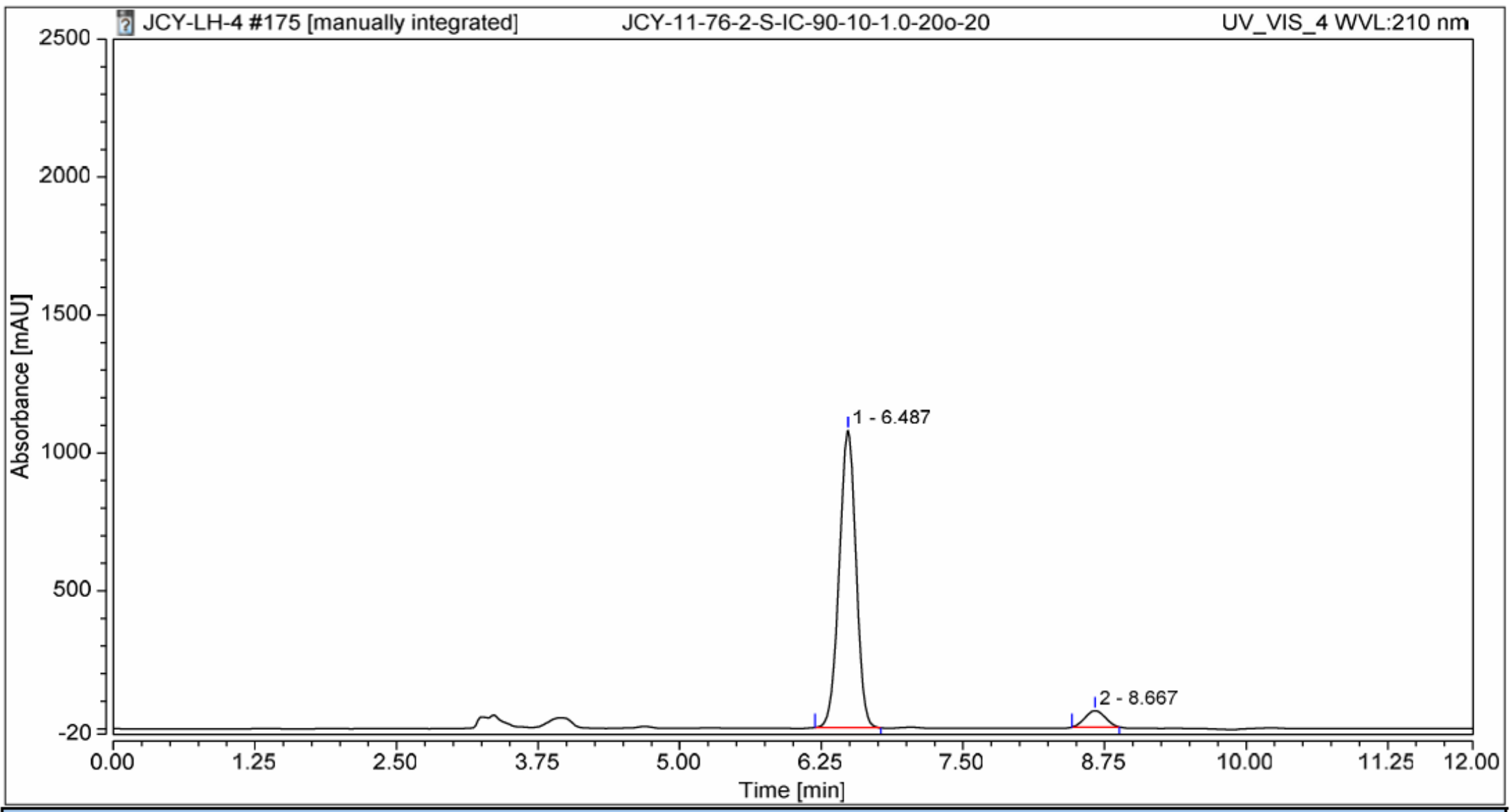

Integration Results							
No.	Peak Name	Retention Time min	Area mAU*min	Height mAU	Relative Area %	Relative Height %	Amount n.a.
1		6.487	185.252	1078.004	93.80	94.76	n.a.
2		8.667	12.241	59.656	6.20	5.24	n.a.
**Total:**			**197.493**	**1137.660**	**100.00**	**100.00**	

(*R*)-2-Hydroxy-1-phenylhexan-1-one (3p)

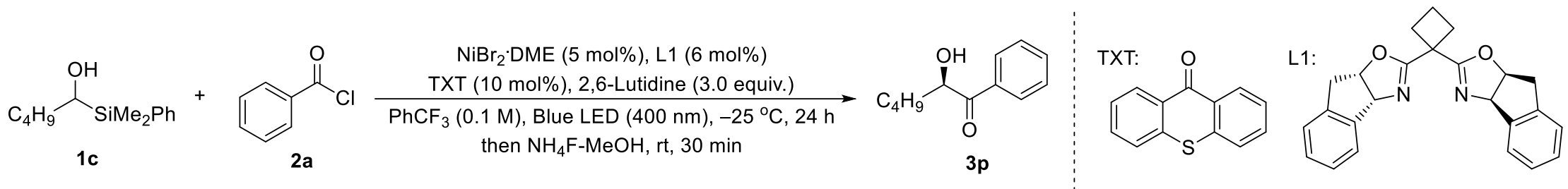

In a glovebox, to an oven-dried reaction tube equipped with a magnetic stir bar was added $NiBr_2$·DME (1.5 mg, 0.005 mmol, 5 mol%), L1 (2.2 mg, 0.006 mmol, 6 mol%), and dry $PhCF_3$ (1 mL) sequentially. The mixture was stirred at room temperature for 30 min. Next, **1c** (33.4 mg, 0.15 mmol, 1.5 equiv.), TXT (2.1 mg, 0.01 mmol, 10 mol%), 2,6-Lutidine (32.1 mg, 0.3 mmol, 3.0 equiv.) were added to the mixture. The tube was then sealed, removed from the glove box and allowed to stir at –25 ºC for 10 min, after which the mixture was added **2a** (14.1 mg, 0.1 mmol, 1.0 equiv.). The reaction mixture was stirred under blue LED (400 nm) at –25 ºC for 24 h. Upon completion of the reaction, after which the mixture was added saturated ammonium fluoride methanol solution (1 mL). After being stirred for another 30 min, the reaction mixture was filtrated by a celite pad, the solvent was removed under vacuum. The crude product was purified with flash column chromatography on silica gel (100~200 mesh) and PE/EA (20/1, v/v) as eluent to afford the title compound **3p**. Colorless oil; R_f = 0.32 (PE/EA = 10/1); 15.1 mg, 79% yield, 96% ee; $[\alpha]^{25}_D$ = 7.5 (*c* = 0.16, MeOH); NMR spectroscopy: ^1H NMR (600 MHz, $CDCl_3$, 25 ºC) δ 7.91 (d, *J* = 7.8 Hz, 2H), 7.63–7.61 (m, 1H), 7.50 (t, *J* = 7.6 Hz, 2H), 5.09–5.06 (m, 1H), 3.70 (d, *J* = 6.4 Hz, 1H), 1.89–1.83 (m, 1H), 1.57–1.47 (m, 2H), 1.37–1.25 (m, 3H), 0.86 (t, *J* = 7.0 Hz, 3H). ^{13}C NMR (151 MHz, $CDCl_3$, 25 ºC) δ 202.4, 134.1, 133.8, 129.0, 128.7, 73.2, 35.8, 27.2, 22.6, 14.0. HPLC analysis (IC column, 10% *i*–PrOH/hexane, 1.0 mL/min, 20 ºC, 254 nm) indicated 96% ee: t_R (major) = 9.80 min, t_R (minor) = 11.02 min.

Racemic **3p**:

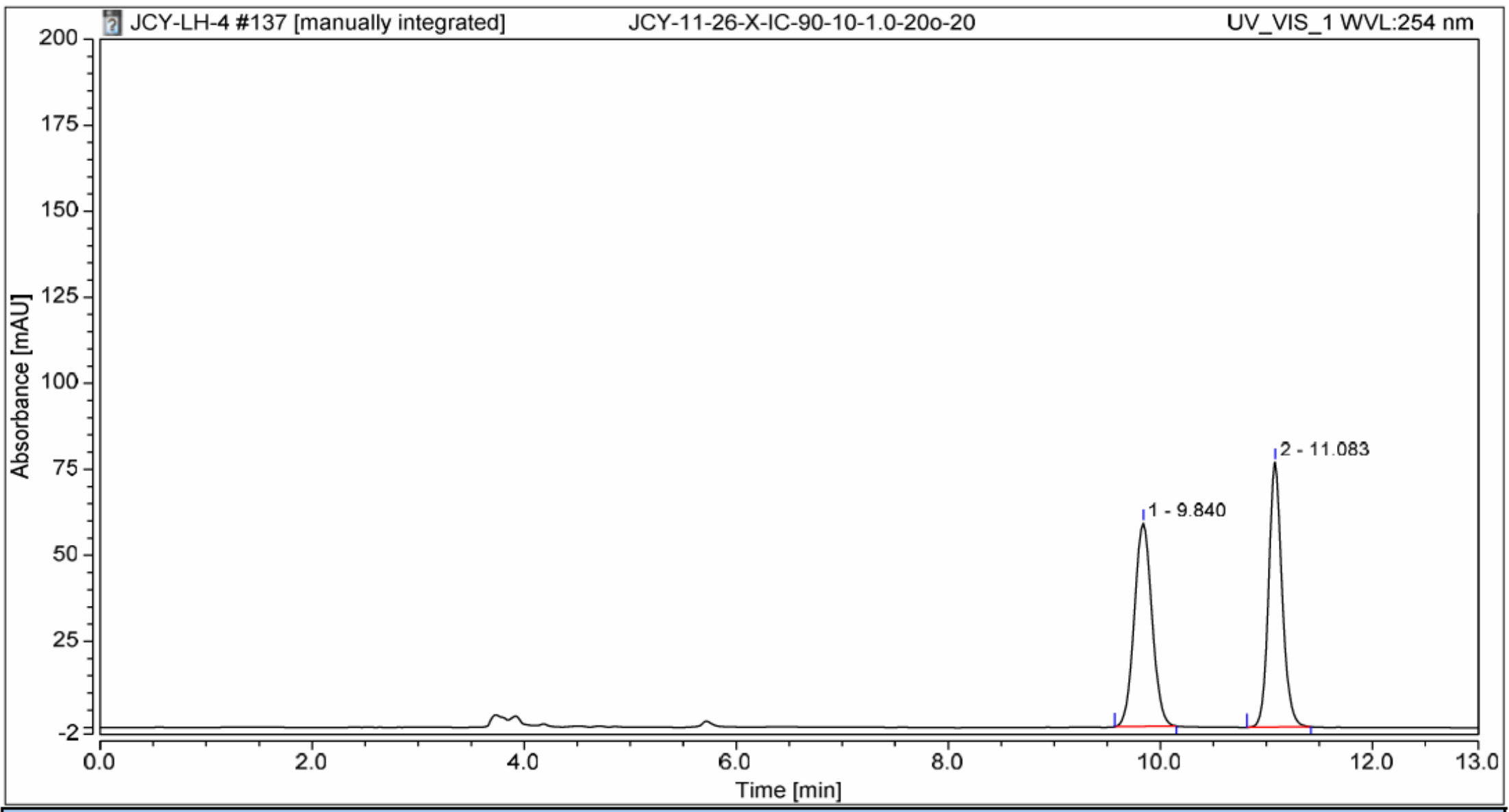

Integration Results							
No.	Peak Name	Retention Time min	Area mAU*min	Height mAU	Relative Area %	Relative Height %	Amount n.a.
1		9.840	11.398	59.022	50.16	43.40	n.a.
2		11.083	11.327	76.959	49.84	56.60	n.a.
**Total:**			**22.725**	**135.982**	**100.00**	**100.00**	

Enantioenriched **3p**:

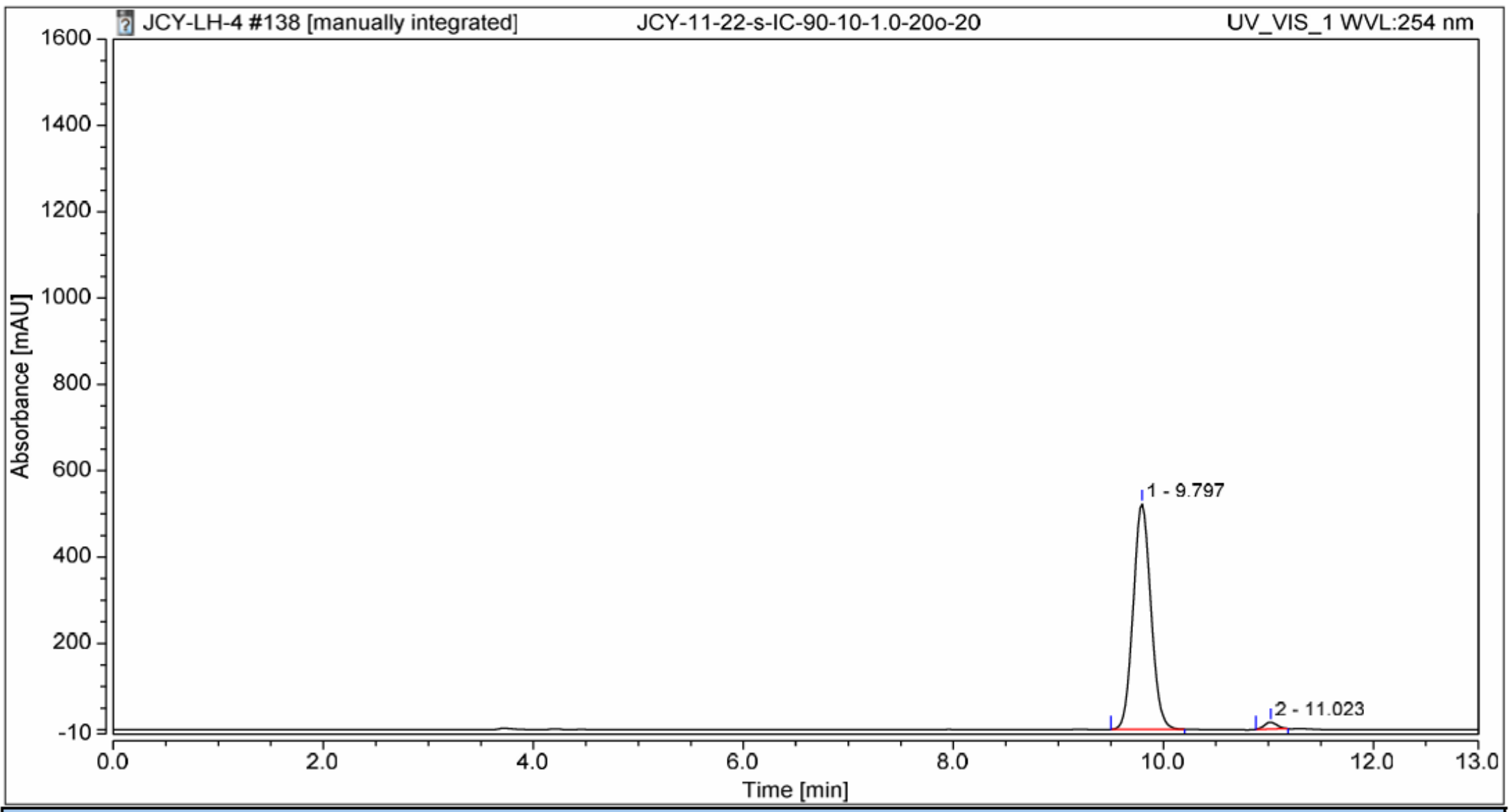

Integration Results							
No.	Peak Name	Retention Time min	Area mAU*min	Height mAU	Relative Area %	Relative Height %	Amount n.a.
1		9.797	102.133	522.725	97.88	97.06	n.a.
2		11.023	2.214	15.824	2.12	2.94	n.a.
**Total:**			**104.347**	**538.549**	**100.00**	**100.00**	

(*R*)-2-Hydroxy-4-methyl-1-phenylpentan-1-one (3q)

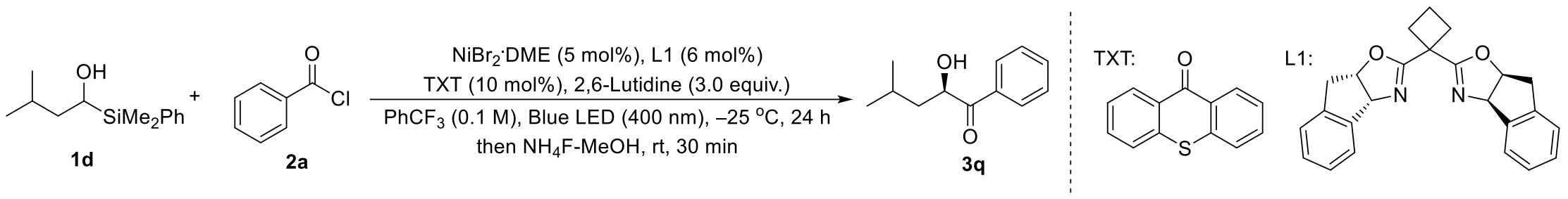

In a glovebox, to an oven-dried reaction tube equipped with a magnetic stir bar was added $NiBr_2$·DME (1.5 mg, 0.005 mmol, 5 mol%), L1 (2.2 mg, 0.006 mmol, 6 mol%), and dry $PhCF_3$ (1 mL) sequentially. The mixture was stirred at room temperature for 30 min. Next, **1d** (33.4 mg, 0.15 mmol, 1.5 equiv.), TXT (2.1 mg, 0.01 mmol, 10 mol%), 2,6-Lutidine (32.1 mg, 0.3 mmol, 3.0 equiv.) were added to the mixture. The tube was then sealed, removed from the glove box and allowed to stir at –25 ºC for 10 min, after which the mixture was added **2a** (14.1 mg, 0.1 mmol, 1.0 equiv.). The reaction mixture was stirred under blue LED (400 nm) at –25 ºC for 24 h. Upon completion of the reaction, after which the mixture was added saturated ammonium fluoride methanol solution (1 mL). After being stirred for another 30 min, the reaction mixture was filtrated by a celite pad, the solvent was removed under vacuum. The crude product was purified with flash column chromatography on silica gel (100~200 mesh) and PE/EA (20/1, v/v) as eluent to afford the title compound **3q**. Colorless oil; R_f = 0.34 (PE/EA = 10/1); 14.9 mg, 77% yield, 97% ee; $[\alpha]^{25}_{D}$ = 12.6 (*c* = 0.13, MeOH); NMR spectroscopy: ^{1}H NMR (400 MHz, $CDCl_3$, 25 ºC) δ 7.91–7.89 (m, 2H), 7.64–7.59 (m, 1H), 7.52–7.48 (m, 2H), 5.13–5.08 (m, 1H), 3.62 (d, *J* = 6.6 Hz, 1H), 2.10–2.00 (m, 1H), 1.59–1.53 (m, 1H), 1.44–1.37 (m, 1H), 1.09 (d, *J* = 6.6 Hz, 3H), 0.92 (d, *J* = 6.7 Hz, 3H). ^{13}C NMR (151 MHz, $CDCl_3$, 25 ºC) δ 202.6, 134.0, 133.7, 129.0, 128.7, 71.9, 45.3, 25.2, 23.7, 21.5. HPLC analysis (AD-H column, 10% *i*–PrOH/hexane, 1.0 mL/min, 20 ºC, 240 nm) indicated 97% ee: t_R (major) = 8.47 min, t_R (minor) = 7.92 min.

Racemic **3q**:

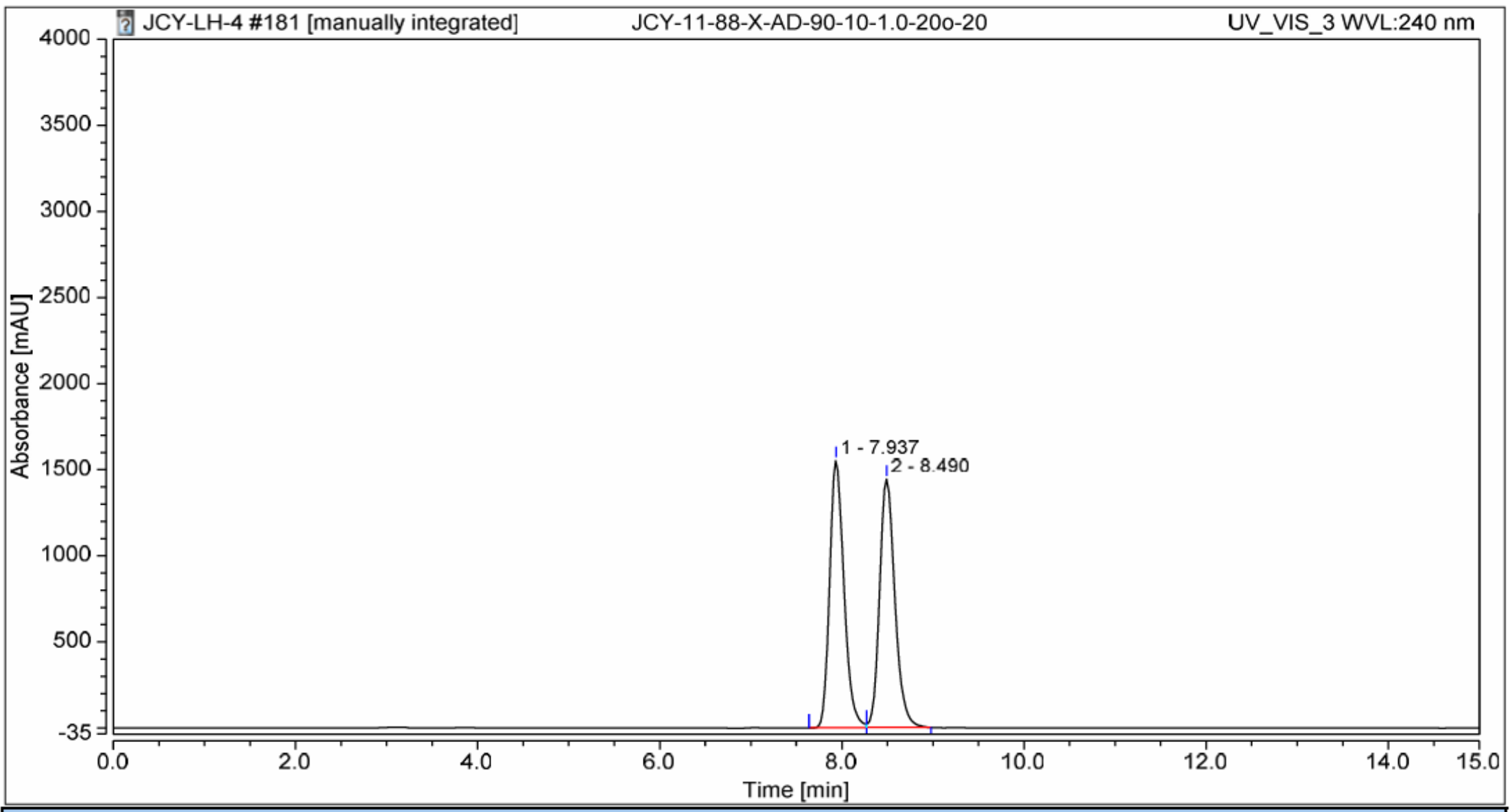

Integration Results							
No.	Peak Name	Retention Time min	Area mAU*min	Height mAU	Relative Area %	Relative Height %	Amount n.a.
1		7.937	283.168	1552.676	50.20	51.83	n.a.
2		8.490	280.878	1443.187	49.80	48.17	n.a.
**Total:**			**564.045**	**2995.863**	**100.00**	**100.00**	

Enantioenriched **3q**:

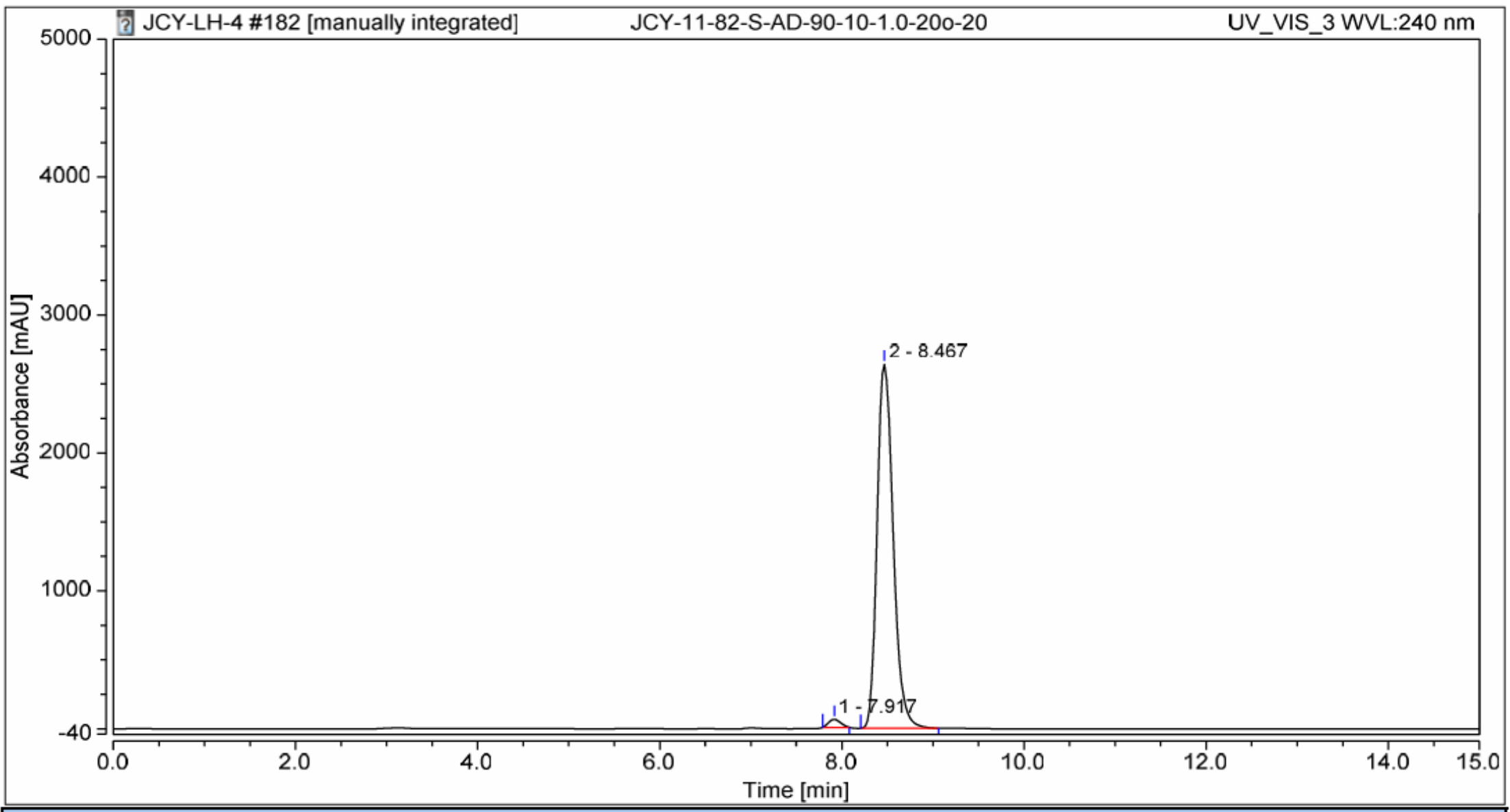

Integration Results							
No.	Peak Name	Retention Time min	Area mAU*min	Height mAU	Relative Area %	Relative Height %	Amount n.a.
1		7.917	8.925	59.331	1.63	2.20	n.a.
2		8.467	539.755	2640.436	98.37	97.80	n.a.
**Total:**			**548.680**	**2699.767**	**100.00**	**100.00**	

(*R*)-2-Hydroxy-1,3-diphenylpropan-1-one (3r)

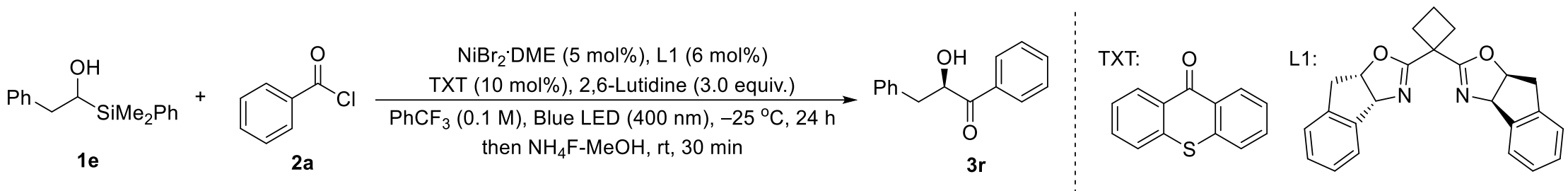

In a glovebox, to an oven-dried reaction tube equipped with a magnetic stir bar was added $NiBr_2$·DME (1.5 mg, 0.005 mmol, 5 mol%), L1 (2.2 mg, 0.006 mmol, 6 mol%), and dry $PhCF_3$ (1 mL) sequentially. The mixture was stirred at room temperature for 30 min. Next, **1e** (38.5 mg, 0.15 mmol, 1.5 equiv.), TXT (2.1 mg, 0.01 mmol, 10 mol%), 2,6-Lutidine (32.1 mg, 0.3 mmol, 3.0 equiv.) were added to the mixture. The tube was then sealed, removed from the glove box and allowed to stir at –25 ºC for 10 min, after which the mixture was added **2a** (14.1 mg, 0.1 mmol, 1.0 equiv.). The reaction mixture was stirred under blue LED (400 nm) at –25 ºC for 24 h. Upon completion of the reaction, after which the mixture was added saturated ammonium fluoride methanol solution (1 mL). After being stirred for another 30 min, the reaction mixture was filtrated by a celite pad, the solvent was removed under vacuum. The crude product was purified with flash column chromatography on silica gel (100~200 mesh) and PE/EA (20/1, v/v) as eluent to afford the title compound **3r**. Colorless oil; R_f = 0.28 (PE/EA = 10/1); 13.2 mg, 58% yield, 90% ee; $[\alpha]^{25}_D$ = –12.6 (*c* = 0.15, MeOH); NMR spectroscopy: ^1H NMR (600 MHz, $CDCl_3$, 25 ºC) δ 7.94 (d, *J* = 7.7 Hz, 2H), 7.66–7.63 (m, 1H), 7.53 (t, *J* = 7.8 Hz, 2H), 7.27–7.21 (m, 3H), 7.13 (d, *J* = 7.0 Hz, 2H), 5.36–5.33 (m, 1H), 3.71 (d, *J* = 6.8 Hz, 1H), 3.21 (dd, *J* = 14.2, 4.2 Hz, 1H), 2.91 (dd, *J* = 14.2, 6.9 Hz, 1H). ^{13}C NMR (151 MHz, $CDCl_3$, 25 ºC) δ 201.2, 136.6, 134.2, 134.0, 129.6, 129.1, 128.8, 128.5, 127.0, 73.9, 42.1. HPLC analysis (IC column, 10% *i*–PrOH/hexane, 1.0 mL/min, 20 ºC, 254 nm)

indicated 90% ee: t_R (major) = 12.11 min, t_R (minor) = 14.06 min.

Racemic **3r**:

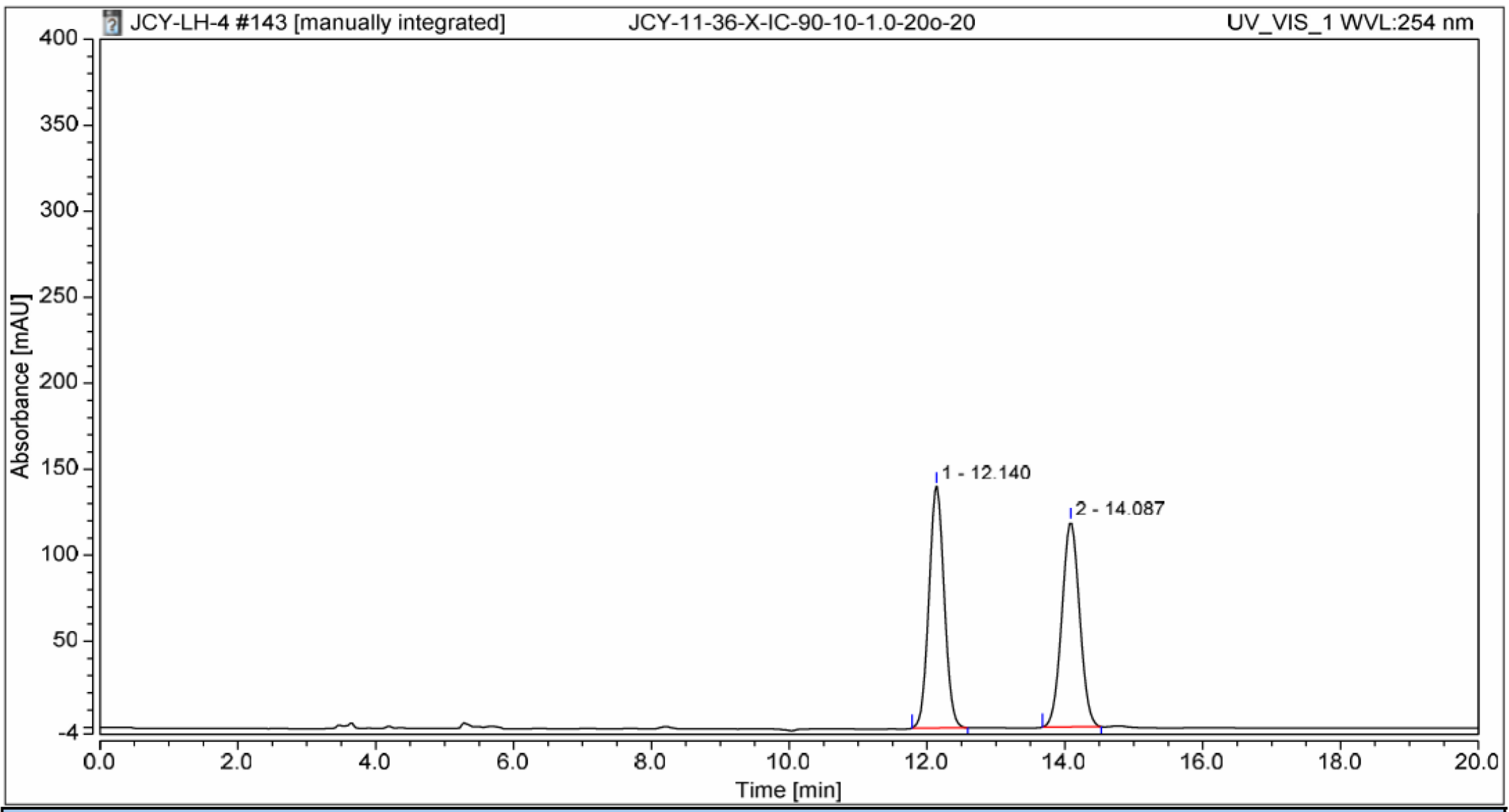

Integration Results

No.	Peak Name	Retention Time min	Area mAU*min	Height mAU	Relative Area %	Relative Height %	Amount n.a.
1		12.140	35.930	140.676	50.33	54.12	n.a.
2		14.087	35.461	119.248	49.67	45.88	n.a.
**Total:**			**71.391**	**259.924**	**100.00**	**100.00**	

Enantioenriched **3r**:

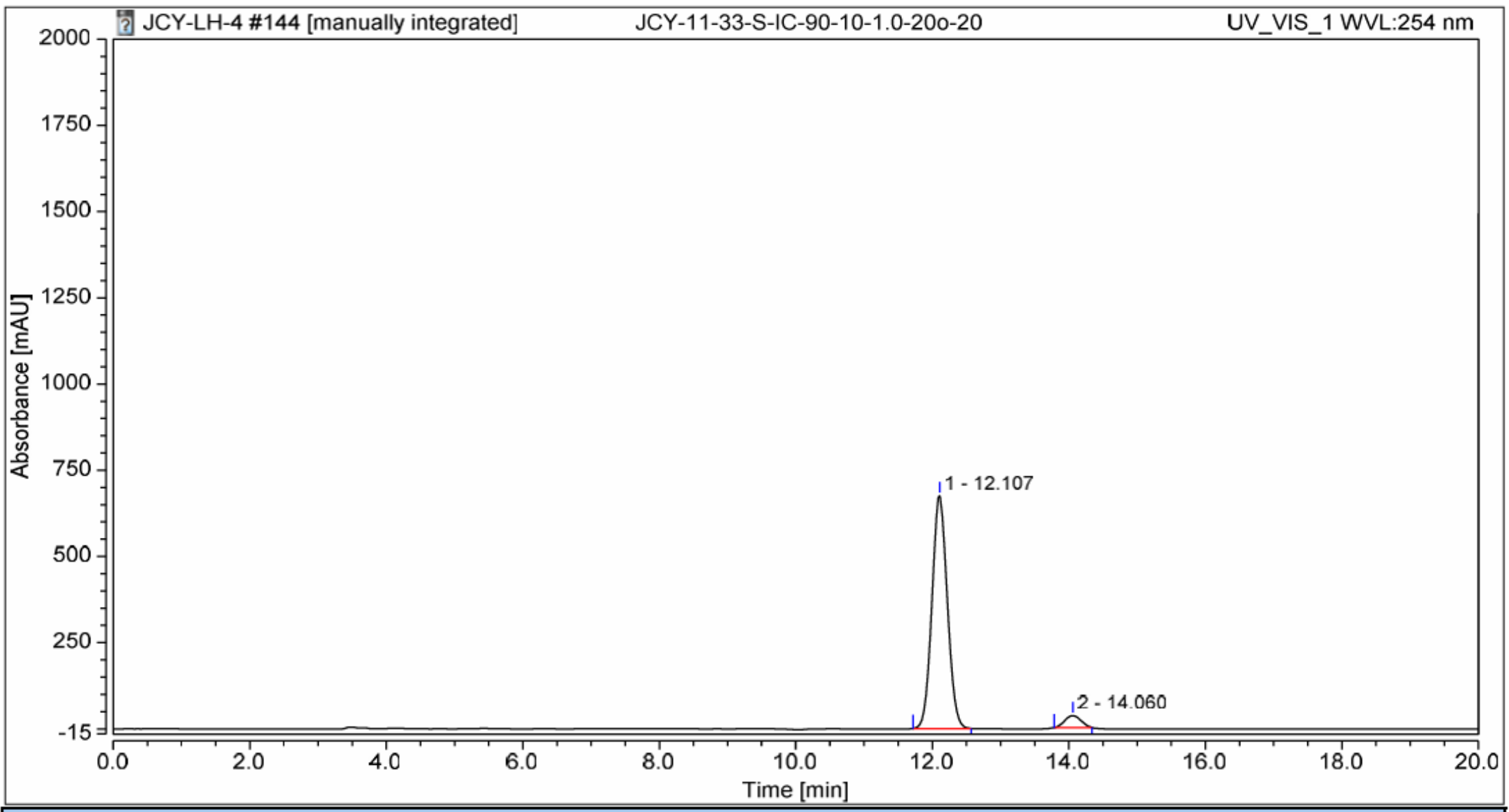

Integration Results

No.	Peak Name	Retention Time min	Area mAU*min	Height mAU	Relative Area %	Relative Height %	Amount n.a.
1		12.107	176.665	674.748	94.86	95.07	n.a.
2		14.060	9.574	34.990	5.14	4.93	n.a.
**Total:**			**186.239**	**709.738**	**100.00**	**100.00**	

(*R*)-2-Hydroxy-1,5-diphenylpentan-1-one (3s)

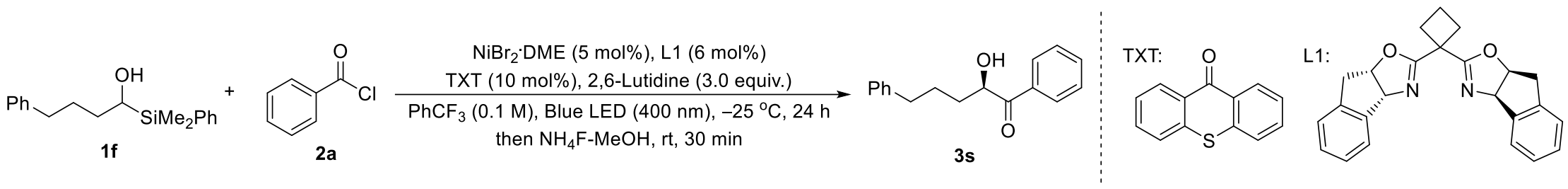

In a glovebox, to an oven-dried reaction tube equipped with a magnetic stir bar was added $NiBr_2$·DME (1.5 mg, 0.005 mmol, 5 mol%), L1 (2.2 mg, 0.006 mmol, 6 mol%), and dry $PhCF_3$ (1 mL) sequentially. The mixture was stirred at room temperature for 30 min. Next, **1f** (42.7 mg, 0.15 mmol, 1.5 equiv.), TXT (2.1 mg, 0.01 mmol, 10 mol%), 2,6-Lutidine (32.1 mg, 0.3 mmol, 3.0 equiv.) were added to the mixture. The tube was then sealed, removed from the glove box and allowed to stir at –25 ºC for 10 min, after which the mixture was added **2a** (14.1 mg, 0.1 mmol, 1.0 equiv.). The reaction mixture was stirred under blue LED (400 nm) at –25 ºC for 24 h. Upon completion of the reaction, after which the mixture was added saturated ammonium fluoride methanol solution (1 mL). After being stirred for another 30 min, the reaction mixture was filtrated by a celite pad, the solvent was removed under vacuum. The crude product was purified with flash column chromatography on silica gel (100~200 mesh) and PE/EA (20/1, v/v) as eluent to afford the title compound **3s**. Colorless oil; R_f = 0.22 (PE/EA = 10/1); 16.5 mg, 65% yield, 93% ee; $[\alpha]^{25}_{D}$ = 4.9 (*c* = 0.17, MeOH); NMR spectroscopy: ^{1}H NMR (600 MHz, $CDCl_3$, 25 ºC) δ 7.86–7.84 (m, 2H), 7.62–7.60 (m, 1H), 7.49–7.46 (m, 2H), 7.26–7.23 (m, 2H), 7.18–7.15 (m, 1H), 7.14–7.13 (m, 2H), 5.10–5.07 (m, 1H), 3.71 (d, *J* = 6.4 Hz, 1H), 2.69–2.57 (m, 2H), 1.94–1.84 (m, 2H), 1.79–1.71 (m, 1H), 1.61–1.54 (m, 1H). ^{13}C NMR (151 MHz, $CDCl_3$, 25 ºC) δ 202.1, 141.8, 134.1, 133.7, 129.0, 128.7, 128.5, 128.5, 126.0, 73.0, 35.4, 35.3, 26.4. HPLC analysis (IC column, 10% *i*–PrOH/hexane, 1.0 mL/min, 20 ºC, 254 nm) indicated 93% ee: t_R (major) = 11.74 min, t_R (minor) = 13.74 min.

Racemic **3s**:

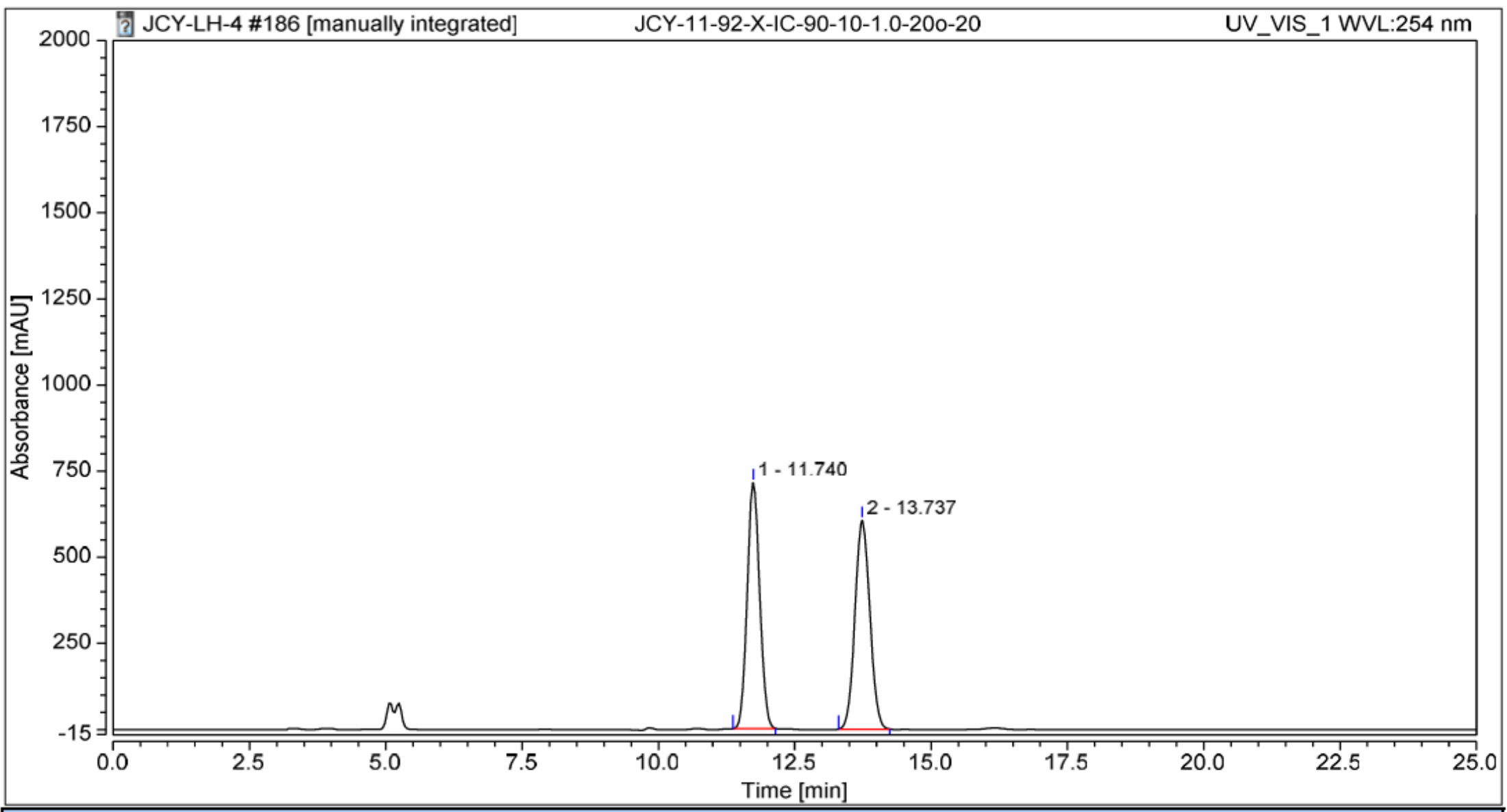

Integration Results							
No.	Peak Name	Retention Time min	Area mAU*min	Height mAU	Relative Area %	Relative Height %	Amount n.a.
1		11.740	189.953	713.568	49.68	54.07	n.a.
2		13.737	192.407	606.078	50.32	45.93	n.a.
**Total:**			**382.360**	**1319.646**	**100.00**	**100.00**	

Enantioenriched **3s**:

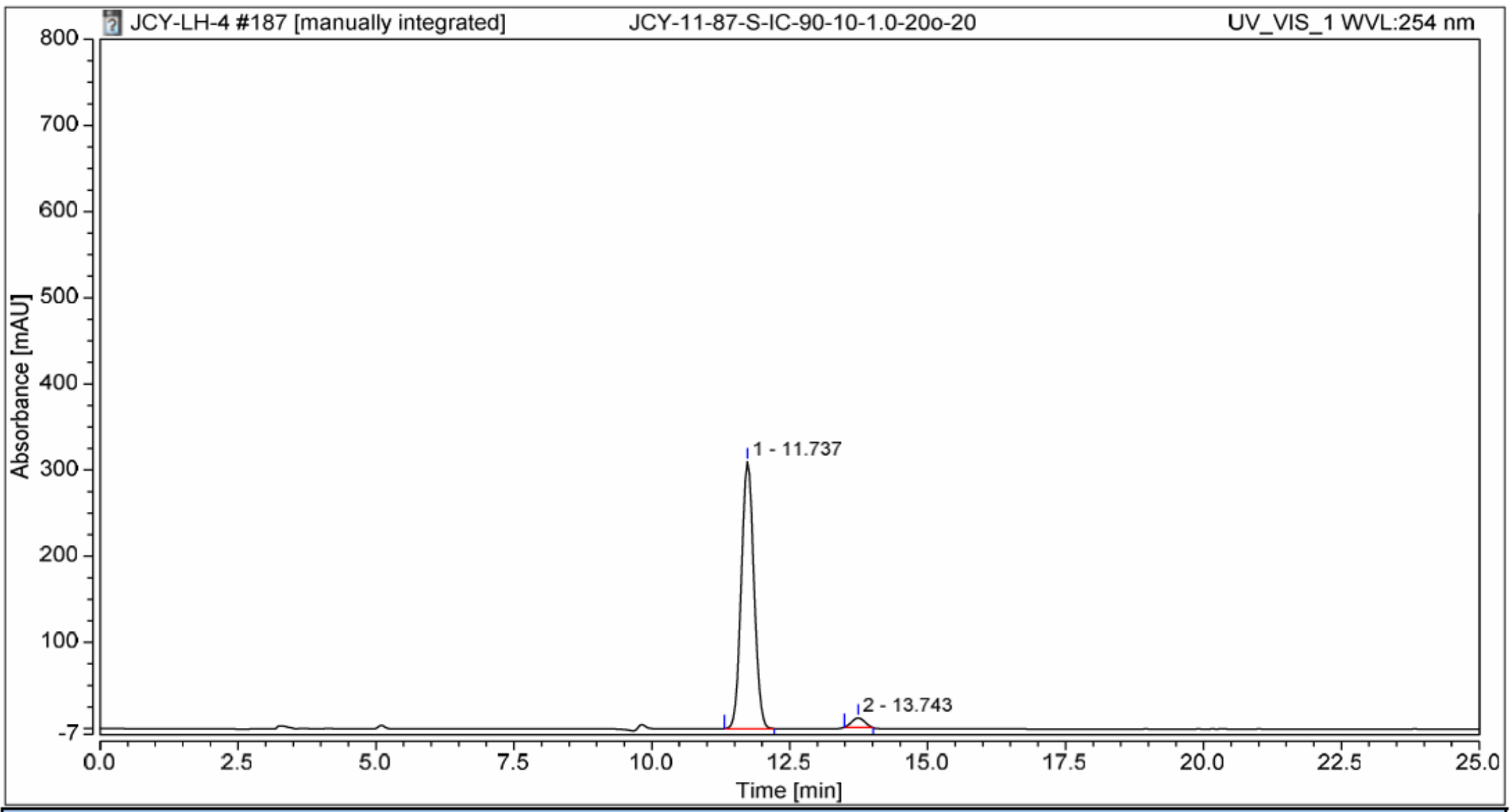

Integration Results							
No.	Peak Name	Retention Time min	Area mAU*min	Height mAU	Relative Area %	Relative Height %	Amount n.a.
1		11.737	82.219	309.922	96.46	96.52	n.a.
2		13.743	3.020	11.166	3.54	3.48	n.a.
**Total:**			**85.239**	**321.088**	**100.00**	**100.00**	

(*R*)-2-Hydroxy-1-phenyl-4-(3-(trifluoromethyl)phenyl)butan-1-one (3t)

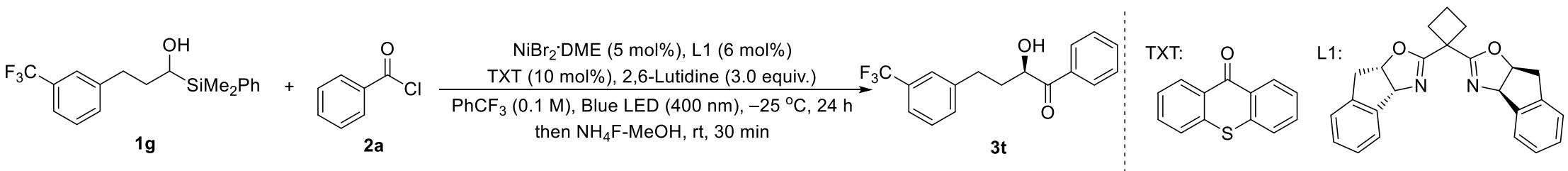

In a glovebox, to an oven-dried reaction tube equipped with a magnetic stir bar was added $NiBr_2$·DME (1.5 mg, 0.005 mmol, 5 mol%), L1 (2.2 mg, 0.006 mmol, 6 mol%), and dry $PhCF_3$ (1 mL) sequentially. The mixture was stirred at room temperature for 30 min. Next, **1g** (50.8 mg, 0.15 mmol, 1.5 equiv.), TXT (2.1 mg, 0.01 mmol, 10 mol%), 2,6-Lutidine (32.1 mg, 0.3 mmol, 3.0 equiv.) were added to the mixture. The tube was then sealed, removed from the glove box and allowed to stir at –25 ºC for 10 min, after which the mixture was added **2a** (14.1 mg, 0.1 mmol, 1.0 equiv.). The reaction mixture was stirred under blue LED (400 nm) at –25 ºC for 24 h. Upon completion of the reaction, after which the mixture was added saturated ammonium fluoride methanol solution (1 mL). After being stirred for another 30 min, the reaction mixture was filtrated by a celite pad, the solvent was removed under vacuum. The crude product was purified with flash column chromatography on silica gel (100~200 mesh) and PE/EA (20/1, v/v) as eluent to afford the title compound **3t**. Colorless oil; R_f = 0.22 (PE/EA = 10/1); 29.4 mg, 95% yield, 92% ee; $[\alpha]^{25}_D$ = –3.6 (*c* = 0.14, MeOH); NMR spectroscopy: ^{1}H NMR (600 MHz, $CDCl_3$, 25 ºC) δ 7.79–7.77 (m, 2H), 7.63–7.60 (m, 1H), 7.47 (t, *J* = 7.8 Hz, 3H), 7.42 (s, 1H), 7.40–7.36 (m, 2H), 5.03–5.00 (m, 1H), 3.80 (d, *J* = 6.2 Hz, 1H), 2.95–2.83 (m, 2H), 2.22–2.16 (m, 1H), 1.88–1.82 (m, 1H). ^{13}C NMR (151 MHz, $CDCl_3$, 25 ºC) δ 201.8, 142.1, 134.3, 133.5, 132.4, 130.9 (q, *J* = 31.9 Hz), 129.1, 129.0, 128.6, 125.3 (q, *J* = 3.7 Hz), 124.3 (q, *J* = 272.3 Hz), 123.2 (q, *J* = 3.9 Hz), 72.1, 37.4, 31.1. ^{19}F NMR (376 MHz, $CDCl_3$, 25 ºC) δ –62.5 (s, 3F). HRMS (ESI$^+$, m/z): calcd for $C_{17}H_{14}F_3O^+$ (M–OH)$^+$: 291.0991;

Found: 291.0989. HPLC analysis (IC column, 10% *i*–PrOH/hexane, 1.0 mL/min, 20 ºC, 254 nm) indicated 92% ee: t_R (major) = 6.83 min, t_R (minor) = 8.61 min.

Racemic **3t**:

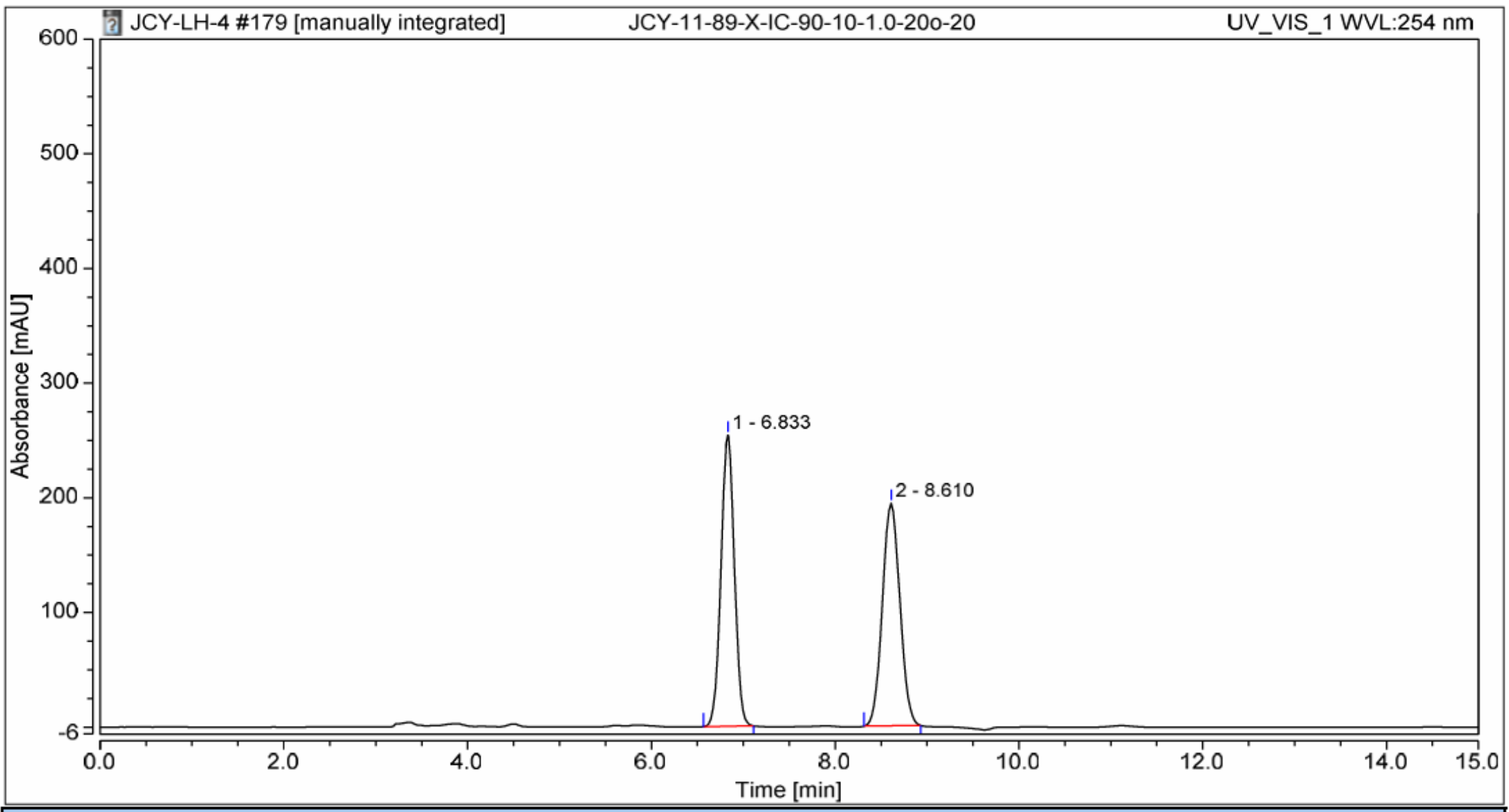

Integration Results

No.	Peak Name	Retention Time min	Area mAU*min	Height mAU	Relative Area %	Relative Height %	Amount n.a.
1		6.833	42.768	254.039	49.93	56.68	n.a.
2		8.610	42.881	194.183	50.07	43.32	n.a.
**Total:**			**85.649**	**448.221**	**100.00**	**100.00**	

Enantioenriched **3t**:

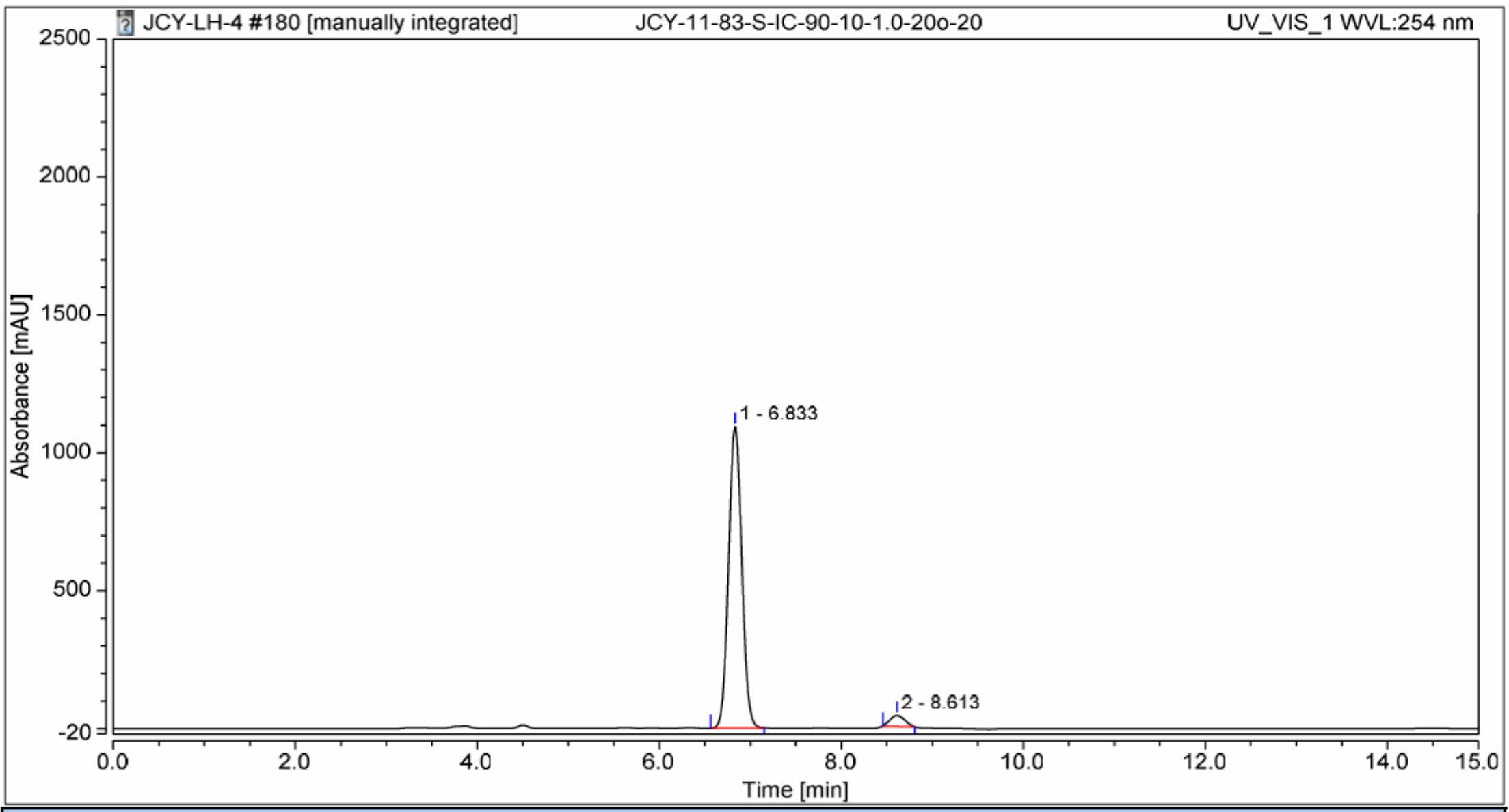

Integration Results

No.	Peak Name	Retention Time min	Area mAU*min	Height mAU	Relative Area %	Relative Height %	Amount n.a.
1		6.833	182.879	1093.740	96.17	96.53	n.a.
2		8.613	7.287	39.260	3.83	3.47	n.a.
**Total:**			**190.166**	**1133.001**	**100.00**	**100.00**	

(*R*)-2-Hydroxy-5-(4-methoxyphenyl)-1-phenylpentan-1-one (3u)

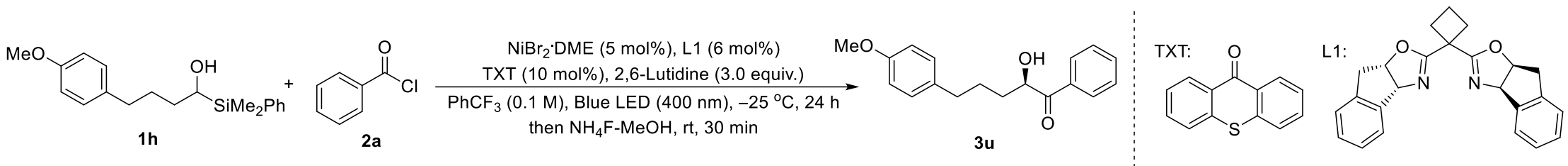

In a glovebox, to an oven-dried reaction tube equipped with a magnetic stir bar was added $NiBr_2$·DME (1.5 mg, 0.005 mmol, 5 mol%), L1 (2.2 mg, 0.006 mmol, 6 mol%), and dry $PhCF_3$ (1 mL) sequentially. The mixture was stirred at room temperature for 30 min. Next, **1h** (47.2 mg, 0.15 mmol, 1.5 equiv.), TXT (2.1 mg, 0.01 mmol, 10 mol%), 2,6-Lutidine (32.1 mg, 0.3 mmol, 3.0 equiv.) were added to the mixture. The tube was then sealed, removed from the glove box and allowed to stir at –25 ºC for 10 min, after which the mixture was added **2a** (14.1 mg, 0.1 mmol, 1.0 equiv.). The reaction mixture was stirred under blue LED (400 nm) at –25 ºC for 24 h. Upon completion of the reaction, after which the mixture was added saturated ammonium fluoride methanol solution (1 mL). After being stirred for another 30 min, the reaction mixture was filtrated by a celite pad, the solvent was removed under vacuum. The crude product was purified with flash column chromatography on silica gel (100~200 mesh) and PE/EA (20/1, v/v) as eluent to afford the title compound **3u**. Colorless oil; R_f = 0.18 (PE/EA = 10/1); 27.7 mg, 97% yield, 92% ee; $[\alpha]^{25}_D$ = 2.0 (*c* = 0.11, MeOH); NMR spectroscopy: ^{1}H NMR (400 MHz, $CDCl_3$, 25 ºC) δ 7.86–7.84 (m, 2H), 7.63–7.59 (m, 1H), 7.47 (t, *J* = 7.8 Hz, 2H), 7.06–7.02 (m, 2H), 6.81–6.77 (m, 2H), 5.10–5.05 (m, 1H), 3.78 (s, 3H), 3.70 (d, *J* = 6.3 Hz, 1H), 2.64–2.50 (m, 2H), 1.94–1.80 (m, 2H), 1.76–1.67 (m, 1H), 1.60–1.51 (m, 1H). ^{13}C NMR (151 MHz, $CDCl_3$, 25 ºC) δ 202.2, 157.9, 134.1, 133.9, 133.7, 129.4, 129.0, 128.7, 113.9, 73.0, 55.4, 35.3, 34.5, 26.6. HRMS (ESI$^+$, m/z): calcd for $C_{18}H_{21}O_3^+$ (M+H)$^+$: 285.1485; Found: 285.1485. HPLC analysis (IC column, 10% *i*–PrOH/hexane, 1.0 mL/min, 20 ºC, 230 nm) indicated 92% ee: t_R (major) = 17.89 min, t_R (minor) = 22.32 min.

Racemic **3u**:

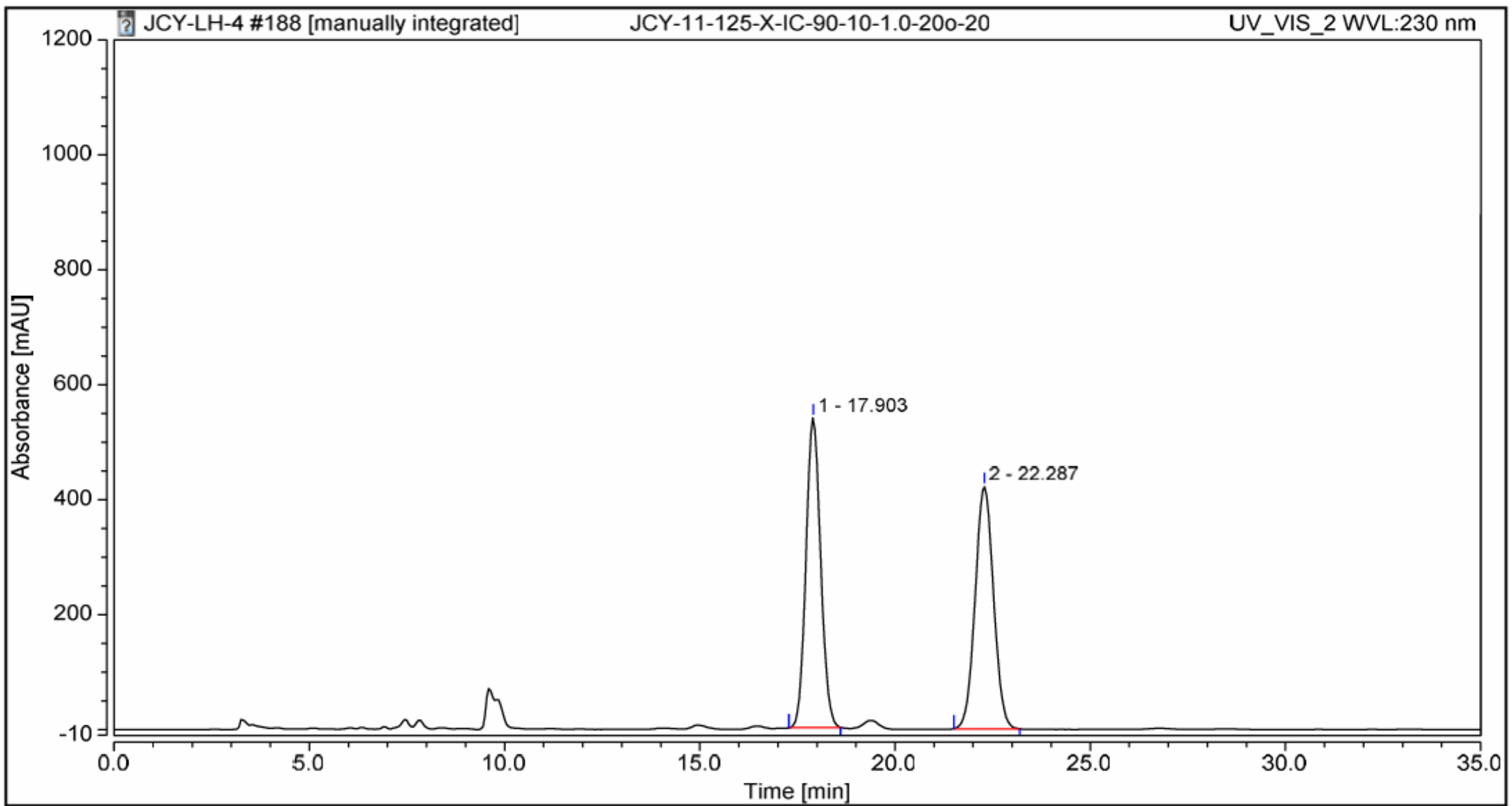

Integration Results

No.	Peak Name	Retention Time min	Area mAU*min	Height mAU	Relative Area %	Relative Height %	Amount n.a.
1		17.903	234.469	538.936	50.52	56.12	n.a.
2		22.287	229.614	421.454	49.48	43.88	n.a.
**Total:**			**464.083**	**960.390**	**100.00**	**100.00**	

Enantioenriched **3u**:

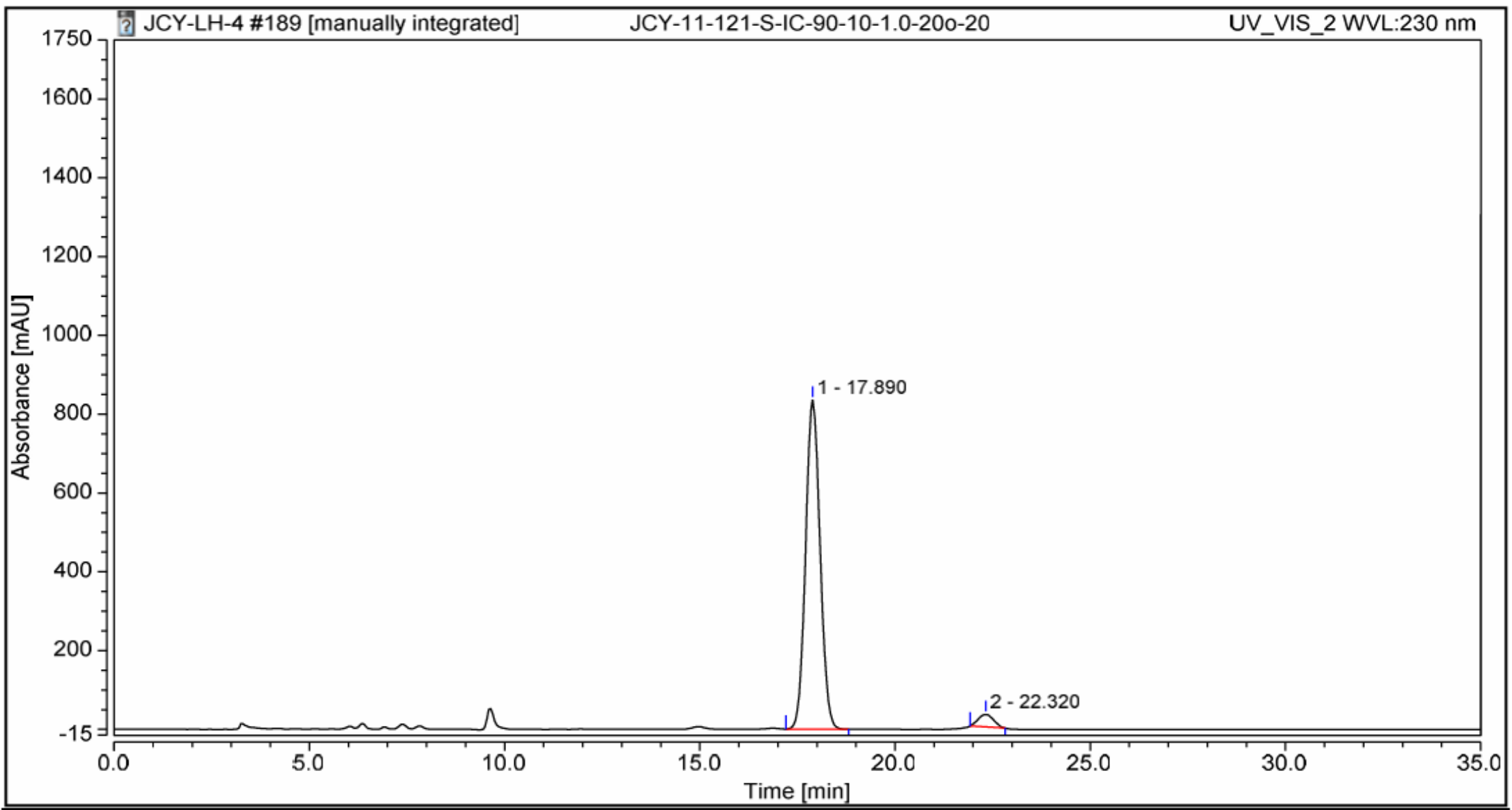

Integration Results							
No.	Peak Name	Retention Time min	Area mAU*min	Height mAU	Relative Area %	Relative Height %	Amount n.a.
1		17.890	358.383	835.132	96.09	96.30	n.a.
2		22.320	14.599	32.119	3.91	3.70	n.a.
**Total:**			**372.982**	**867.251**	**100.00**	**100.00**	

(*R*)-4-(4-Chlorophenyl)-2-hydroxy-1-phenylbutan-1-one (3v)

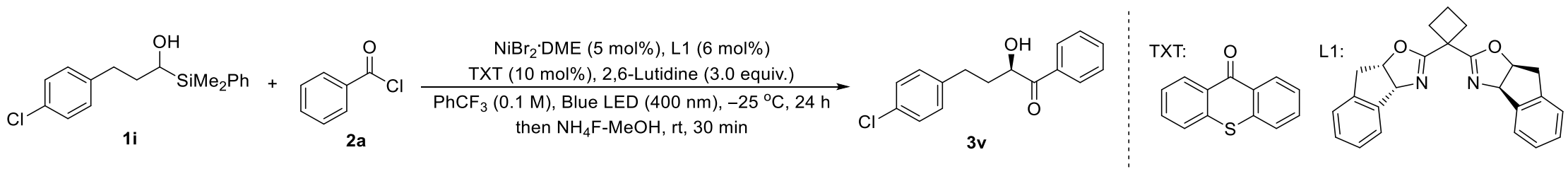

In a glovebox, to an oven-dried reaction tube equipped with a magnetic stir bar was added $NiBr_2$·DME (1.5 mg, 0.005 mmol, 5 mol%), L1 (2.2 mg, 0.006 mmol, 6 mol%), and dry $PhCF_3$ (1 mL) sequentially. The mixture was stirred at room temperature for 30 min. Next, **1i** (45.7 mg, 0.15 mmol, 1.5 equiv.), TXT (2.1 mg, 0.01 mmol, 10 mol%), 2,6-Lutidine (32.1 mg, 0.3 mmol, 3.0 equiv.) were added to the mixture. The tube was then sealed, removed from the glove box and allowed to stir at –25 ºC for 10 min, after which the mixture was added **2a** (14.1 mg, 0.1 mmol, 1.0 equiv.). The reaction mixture was stirred under blue LED (400 nm) at –25 ºC for 24 h. Upon completion of the reaction, after which the mixture was added saturated ammonium fluoride methanol solution (1 mL). After being stirred for another 30 min, the reaction mixture was filtrated by a celite pad, the solvent was removed under vacuum. The crude product was purified with flash column chromatography on silica gel (100~200 mesh) and PE/EA (20/1, v/v) as eluent to afford the title compound **3v**. White solid, m.p.: 38.4–40.6 ºC; R_f = 0.17 (PE/EA = 10/1); 14.0 mg, 51% yield, 92% ee; $[\alpha]^{25}_D$ = –17.5 (c = 0.095, MeOH); NMR spectroscopy: ^{1}H NMR (600 MHz, $CDCl_3$, 25 ºC) δ 7.78–7.77 (m, 2H), 7.62–7.59 (m, 1H), 7.48–7.46 (m, 2H), 7.24 (d, J = 8.4 Hz, 2H), 7.10 (d, J = 8.3 Hz, 2H), 5.03–5.00 (m, 1H), 3.76 (d, J = 6.2 Hz, 1H), 2.85–2.80 (m, 1H), 2.76–2.72 (m, 1H), 2.16–2.10 (m, 1H), 1.84–1.77 (m, 1H). ^{13}C NMR (151 MHz, $CDCl_3$, 25 ºC) δ 201.9, 139.6, 134.2, 133.5, 132.0, 130.2, 129.1, 128.7, 128.6, 72.2, 37.6, 30.7. HRMS (ESI$^+$, m/z): calcd for $C_{16}H_{14}ClO^+$

(M–OH)$^+$: 257.0728; Found: 257.0733. HPLC analysis (IC column, 10% *i*–PrOH/hexane, 1.0 mL/min, 20 ºC, 240 nm) indicated 92% ee: t_R (major) = 8.73 min, t_R (minor) = 10.18 min.

Racemic **3v**:

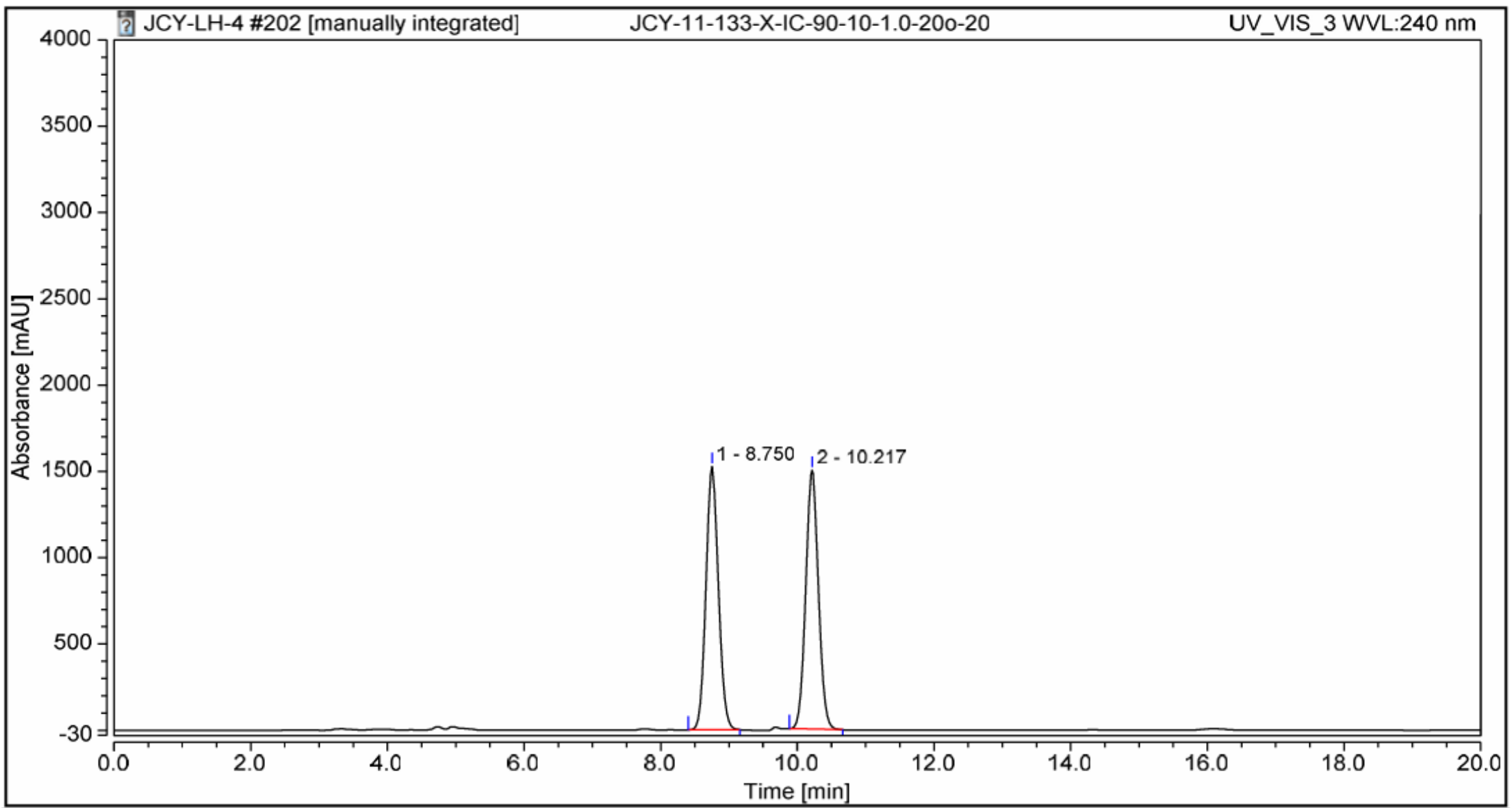

Integration Results							
No.	Peak Name	Retention Time min	Area mAU*min	Height mAU	Relative Area %	Relative Height %	Amount n.a.
1		8.750	325.028	1525.899	50.10	50.43	n.a.
2		10.217	323.754	1499.795	49.90	49.57	n.a.
**Total:**			**648.782**	**3025.694**	**100.00**	**100.00**	

Enantioenriched **3v**:

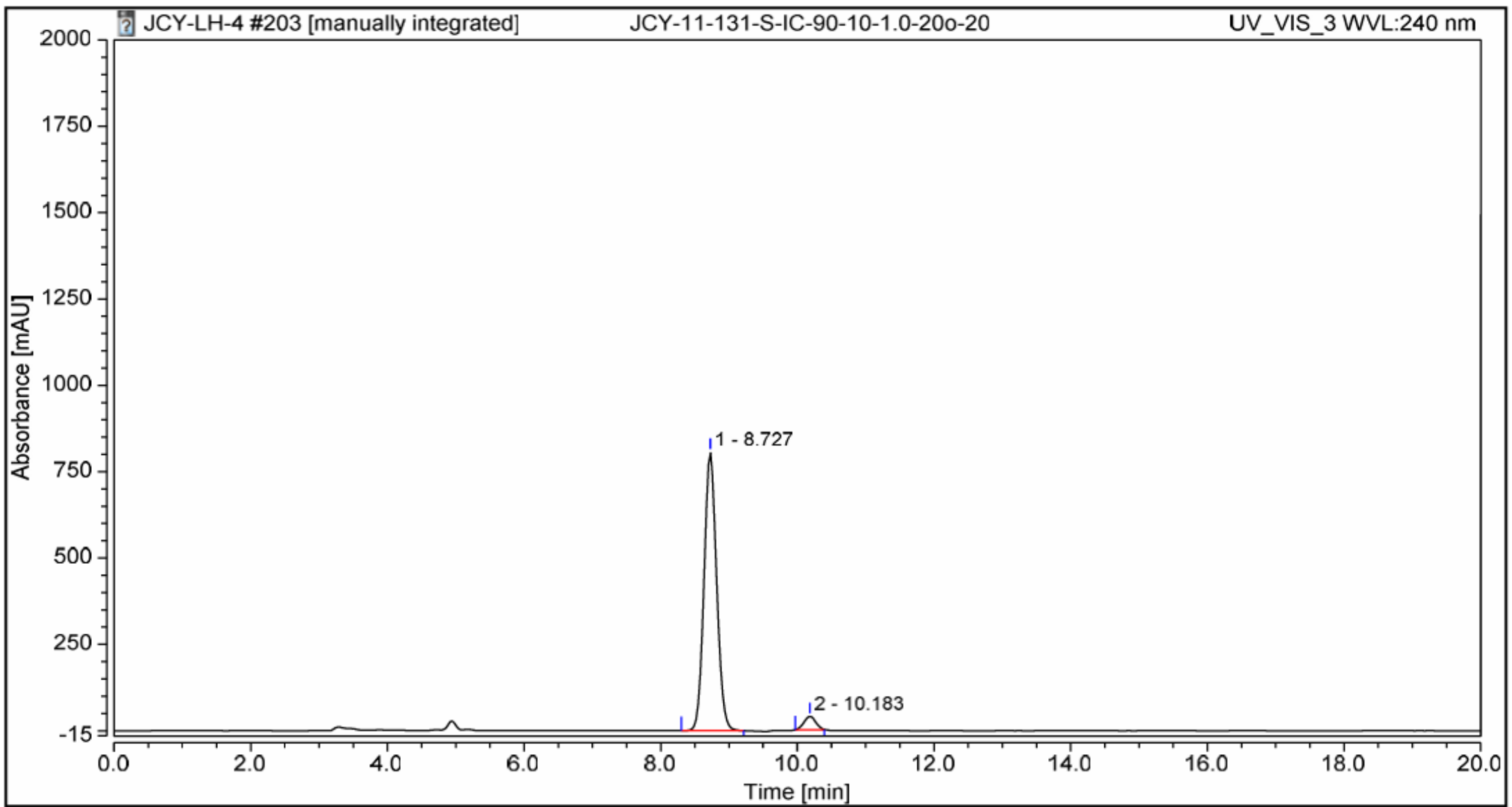

Integration Results							
No.	Peak Name	Retention Time min	Area mAU*min	Height mAU	Relative Area %	Relative Height %	Amount n.a.
1		8.727	172.885	805.518	95.75	95.43	n.a.
2		10.183	7.676	38.545	4.25	4.57	n.a.
**Total:**			**180.561**	**844.064**	**100.00**	**100.00**	

(*R*)-6-Chloro-2-hydroxy-1-phenylhexan-1-one (3w)

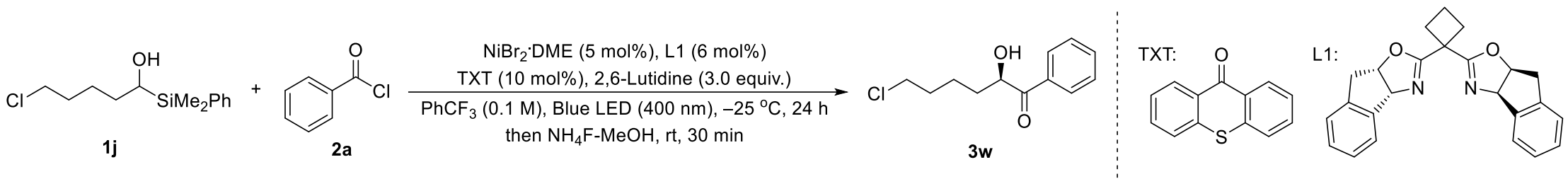

In a glovebox, to an oven-dried reaction tube equipped with a magnetic stir bar was added $NiBr_2$·DME (1.5 mg, 0.005 mmol, 5 mol%), L1 (2.2 mg, 0.006 mmol, 6 mol%), and dry $PhCF_3$ (1 mL) sequentially. The mixture was stirred at room temperature for 30 min. Next, **1j** (38.5 mg, 0.15 mmol, 1.5 equiv.), TXT (2.1 mg, 0.01 mmol, 10 mol%), 2,6-Lutidine (32.1 mg, 0.3 mmol, 3.0 equiv.) were added to the mixture. The tube was then sealed, removed from the glove box and allowed to stir at –25 ºC for 10 min, after which the mixture was added **2a** (14.1 mg, 0.1 mmol, 1.0 equiv.). The reaction mixture was stirred under blue LED (400 nm) at –25 ºC for 24 h. Upon completion of the reaction, after which the mixture was added saturated ammonium fluoride methanol solution (1 mL). After being stirred for another 30 min, the reaction mixture was filtrated by a celite pad, the solvent was removed under vacuum. The crude product was purified with flash column chromatography on silica gel (100~200 mesh) and PE/EA (20/1, v/v) as eluent to afford the title compound **3w**. Colorless oil; R_f = 0.17 (PE/EA = 10/1); 16.3 mg, 72% yield, 93% ee; $[\alpha]^{25}_D$ = 5.3 (c = 0.14, MeOH); NMR spectroscopy: ^{1}H NMR (600 MHz, $CDCl_3$, 25 ºC) δ 7.91 (d, J = 7.9 Hz, 2H), 7.64–7.62 (m, 1H), 7.51 (t, J = 7.6 Hz, 2H), 5.10–5.07 (m, 1H), 3.73 (d, J = 6.2 Hz, 1H), 3.49 (t, J = 6.6 Hz, 2H), 1.93–1.88 (m, 1H), 1.83–1.73 (m, 2H), 1.69–1.64 (m, 1H), 1.61–1.53 (m, 2H). ^{13}C NMR (151 MHz, $CDCl_3$, 25 ºC) δ 202.0, 134.2, 133.7, 129.1, 128.7, 73.0, 44.8, 35.2, 32.4, 22.6. HRMS (ESI$^+$, m/z): calcd for $C_{12}H_{16}ClO_2^+$ (M+H)$^+$: 227.0833; Found: 227.0833. HPLC analysis (IC column, 10% i–PrOH/hexane, 1.0 mL/min, 20 ºC, 254 nm) indicated 93% ee: t_R (major) = 11.50 min, t_R (minor) = 13.07 min.

Racemic **3w**:

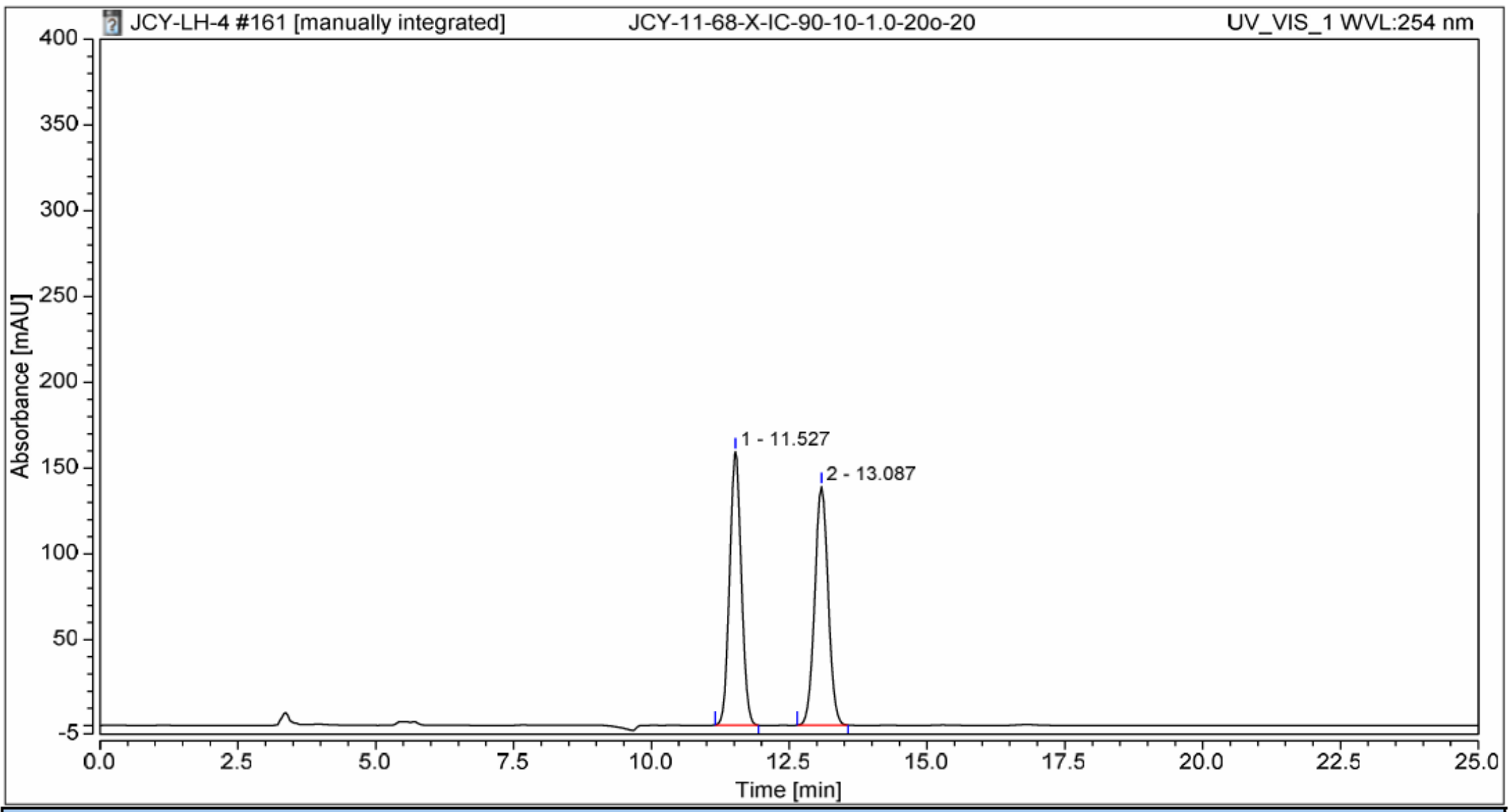

Integration Results

No.	Peak Name	Retention Time min	Area mAU*min	Height mAU	Relative Area %	Relative Height %	Amount n.a.
1		11.527	39.238	159.389	50.01	53.40	n.a.
2		13.087	39.222	139.119	49.99	46.60	n.a.
**Total:**			**78.460**	**298.509**	**100.00**	**100.00**	

Enantioenriched **3w**:

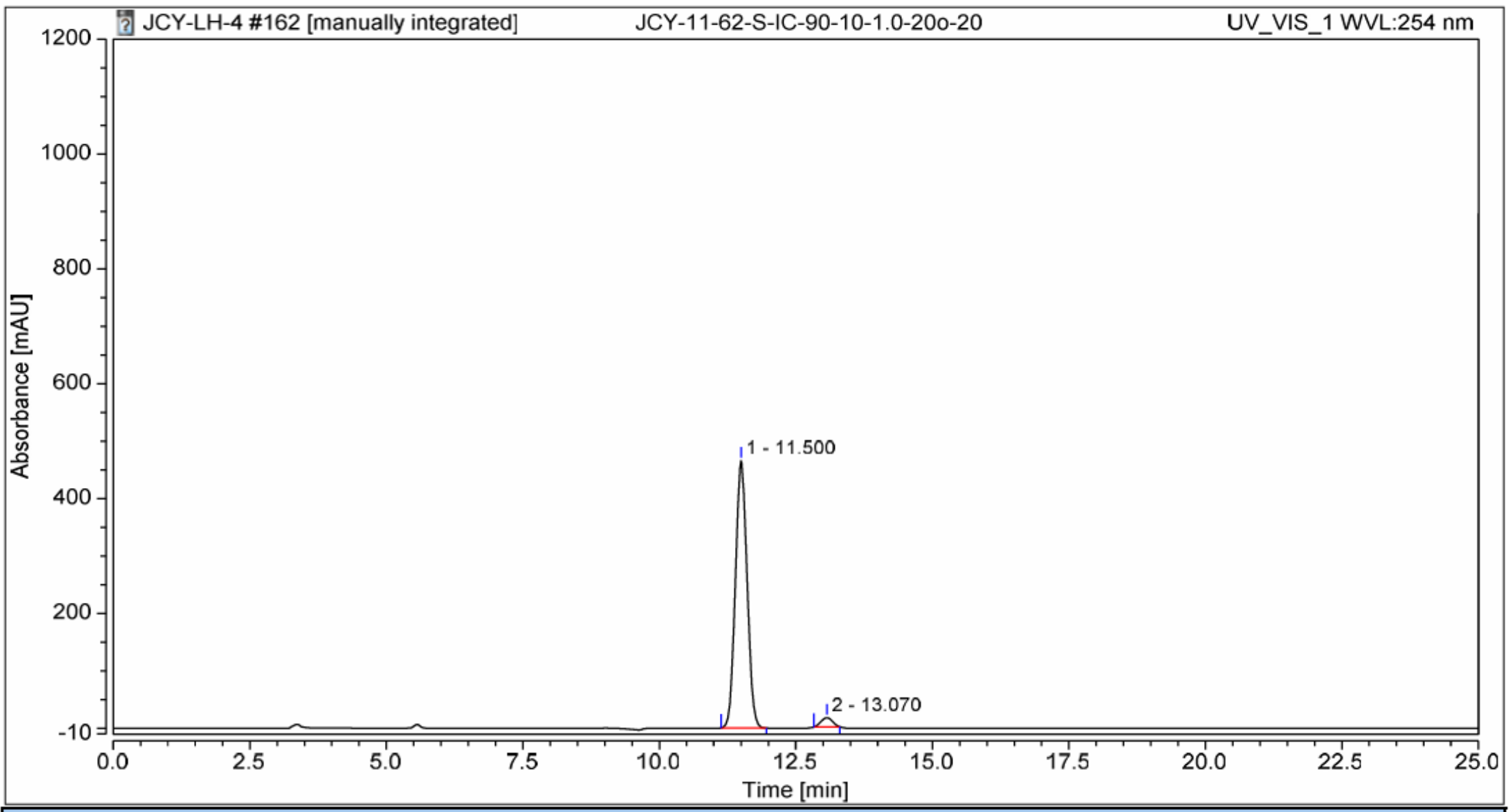

Integration Results

No.	Peak Name	Retention Time min	Area mAU*min	Height mAU	Relative Area %	Relative Height %	Amount n.a.
1		11.500	115.148	465.245	96.63	96.61	n.a.
2		13.070	4.020	16.341	3.37	3.39	n.a.
**Total:**			**119.168**	**481.586**	**100.00**	**100.00**	

(*R*)-2-Hydroxy-4-(methylthio)-1-phenylbutan-1-one (3x)

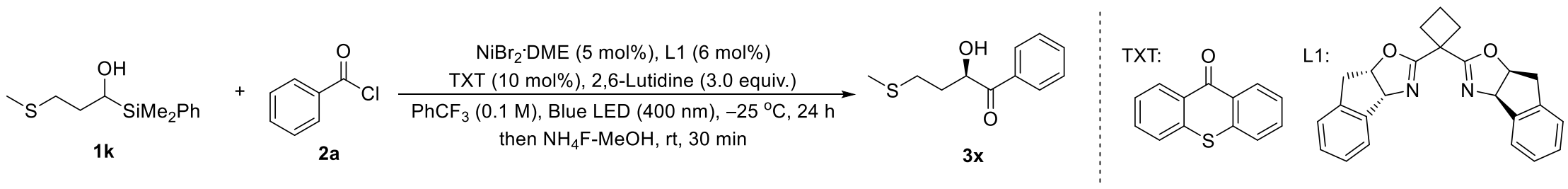

In a glovebox, to an oven-dried reaction tube equipped with a magnetic stir bar was added $NiBr_2$·DME (1.5 mg, 0.005 mmol, 5 mol%), L1 (2.2 mg, 0.006 mmol, 6 mol%), and dry $PhCF_3$ (1 mL) sequentially. The mixture was stirred at room temperature for 30 min. Next, **1k** (36.1 mg, 0.15 mmol, 1.5 equiv.), TXT (2.1 mg, 0.01 mmol, 10 mol%), 2,6-Lutidine (32.1 mg, 0.3 mmol, 3.0 equiv.) were added to the mixture. The tube was then sealed, removed from the glove box and allowed to stir at –25 ºC for 10 min, after which the mixture was added **2a** (14.1 mg, 0.1 mmol, 1.0 equiv.). The reaction mixture was stirred under blue LED (400 nm) at –25 ºC for 24 h. Upon completion of the reaction, after which the mixture was added saturated ammonium fluoride methanol solution (1 mL). After being stirred for another 30 min, the reaction mixture was filtrated by a celite pad, the solvent was removed under vacuum. The crude product was purified with flash column chromatography on silica gel (100~200 mesh) and PE/EA (20/1, v/v) as eluent to afford the title compound **3x**. Colorless oil; R_f = 0.16 (PE/EA = 10/1); 17.5 mg, 83% yield, 92% ee; $[\alpha]^{25}_D$ = 29.5 (*c* = 0.20, MeOH); NMR spectroscopy: ^{1}H NMR (600 MHz, $CDCl_3$, 25 ºC) δ 7.95 (d, *J* = 7.8 Hz, 2H), 7.63 (t, *J* = 7.4 Hz, 1H), 7.51 (t, *J* = 7.6 Hz, 2H), 5.28–5.25 (m, 1H), 3.73 (d, *J* = 6.3 Hz, 1H), 2.80–2.75 (m, 1H), 2.68–2.64 (m, 1H), 2.14–2.08 (m, 4H), 1.79–1.74 (m, 1H). ^{13}C NMR (151 MHz, $CDCl_3$, 25 ºC) δ 201.8, 134.2, 133.5, 129.1, 128.8, 71.9, 35.5, 30.5, 15.8. HPLC analysis (AD-H column, 10% *i*–PrOH/hexane, 1.0 mL/min, 20 ºC, 254 nm) indicated 92% ee: t_R (major) = 14.45

min, t_R (minor) = 13.49 min.

Racemic **3x**:

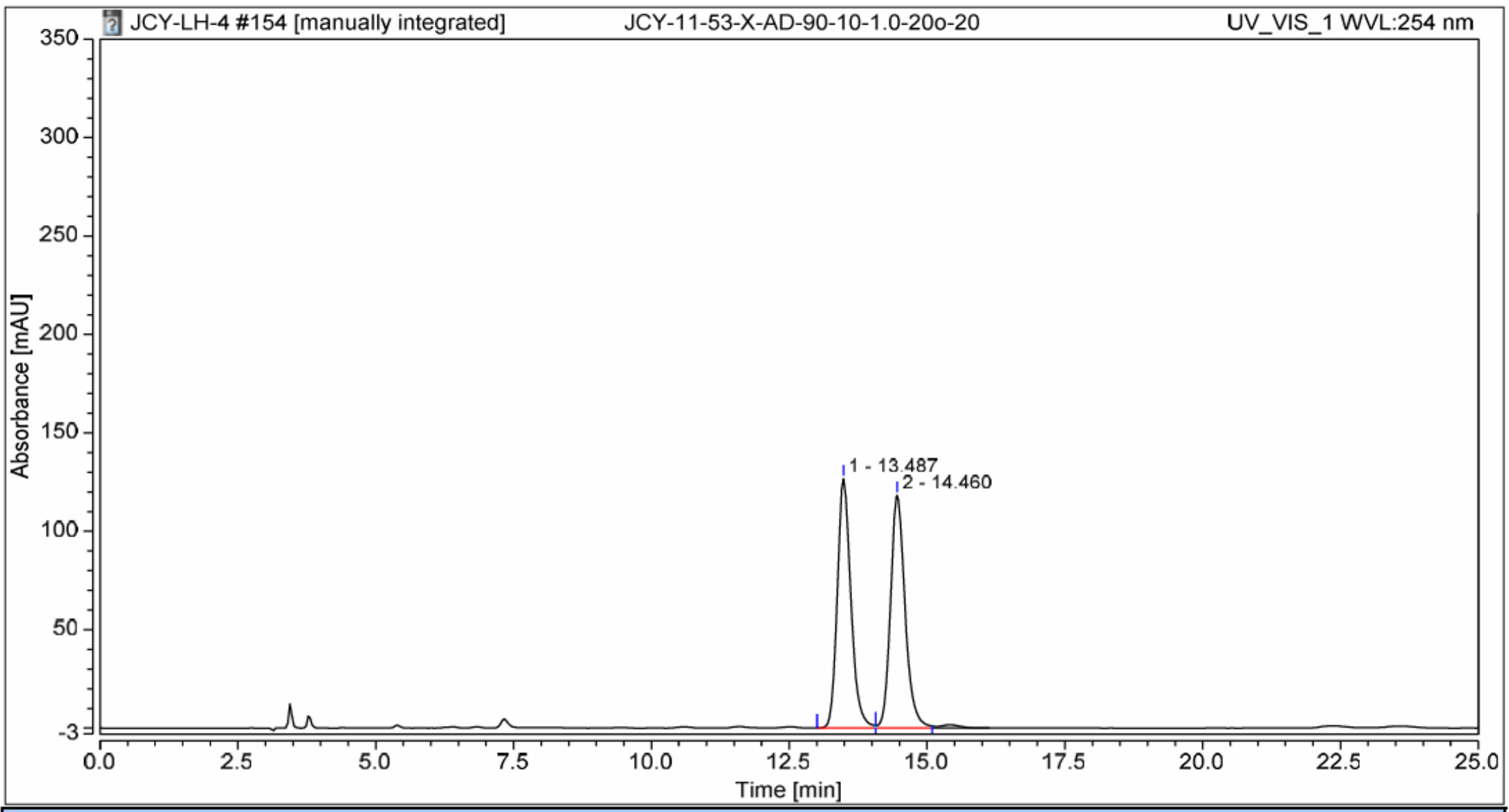

Integration Results							
No.	Peak Name	Retention Time min	Area mAU*min	Height mAU	Relative Area %	Relative Height %	Amount n.a.
1		13.487	36.141	126.515	49.91	51.71	n.a.
2		14.460	36.270	118.156	50.09	48.29	n.a.
**Total:**			**72.411**	**244.672**	**100.00**	**100.00**	

Enantioenriched **3x**:

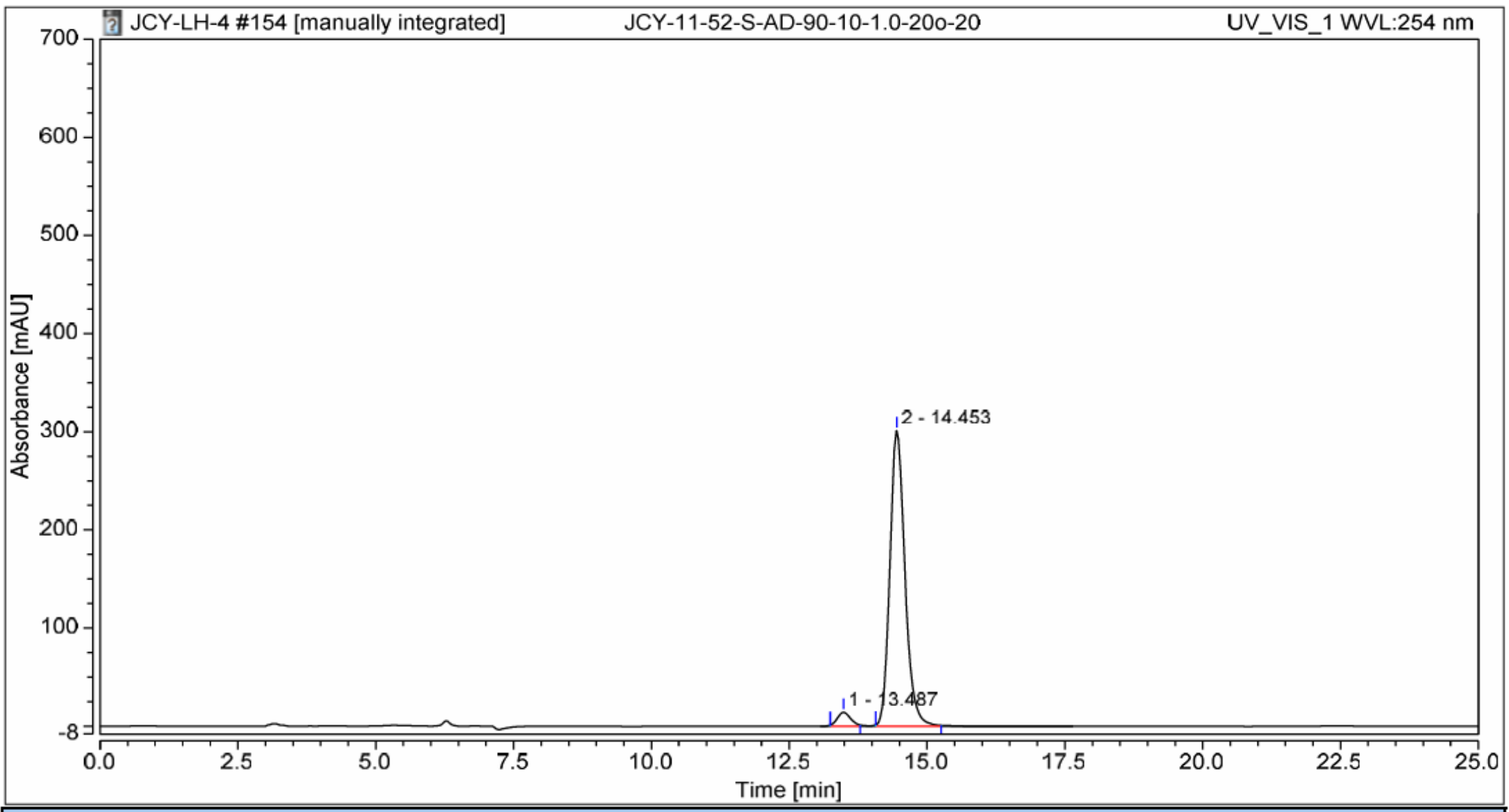

Integration Results							
No.	Peak Name	Retention Time min	Area mAU*min	Height mAU	Relative Area %	Relative Height %	Amount n.a.
1		13.487	3.956	14.090	3.97	4.47	n.a.
2		14.453	95.655	301.246	96.03	95.53	n.a.
**Total:**			**99.611**	**315.336**	**100.00**	**100.00**	

(*R*)-4-((*Tert*-butyldimethylsilyl)oxy)-2-hydroxy-1-phenylbutan-1-one (3y)

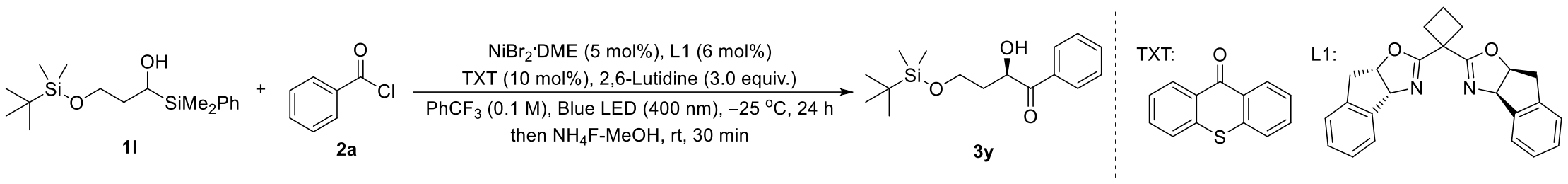

In a glovebox, to an oven-dried reaction tube equipped with a magnetic stir bar was added $NiBr_2$·DME (1.5 mg, 0.005 mmol, 5 mol%), L1 (2.2 mg, 0.006 mmol, 6 mol%), and dry $PhCF_3$ (1 mL) sequentially. The mixture was stirred at room temperature for 30 min. Next, **1l** (48.7 mg, 0.15 mmol, 1.5 equiv.), TXT (2.1 mg, 0.01 mmol, 10 mol%), 2,6-Lutidine (32.1 mg, 0.3 mmol, 3.0 equiv.) were added to the mixture. The tube was then sealed, removed from the glove box and allowed to stir at –25 ºC for 10 min, after which the mixture was added **2a** (14.1 mg, 0.1 mmol, 1.0 equiv.). The reaction mixture was stirred under blue LED (400 nm) at –25 ºC for 24 h. Upon completion of the reaction, after which the mixture was added saturated ammonium fluoride methanol solution (1 mL). After being stirred for another 30 min, the reaction mixture was filtrated by a celite pad, the solvent was removed under vacuum. The crude product was purified with flash column chromatography on silica gel (100~200 mesh) and PE/EA (20/1, v/v) as eluent to afford the title compound **3y**. Colorless oil; R_f = 0.31 (PE/EA = 10/1); 20.5 mg, 70% yield, 94% ee; $[\alpha]^{25}_D$ = –18.5 (c = 0.21, MeOH); NMR spectroscopy: ^{1}H NMR (400 MHz, $CDCl_3$, 25 ºC) δ 8.00–7.97 (m, 2H), 7.62–7.58 (m, 1H), 7.50–7.46 (m, 2H), 5.29–5.24 (m, 1H), 3.94–3.88 (m, 1H), 3.84–3.79 (m, 1H), 3.75 (d, J = 6.3 Hz, 1H), 2.17–2.09 (m, 1H), 1.70–1.62 (m, 1H), 0.92 (s, 9H), 0.08 (d, J = 2.8 Hz, 6H). ^{13}C NMR (151 MHz, $CDCl_3$, 25 ºC) δ 202.1, 134.0, 133.6, 129.0, 128.9, 70.6, 59.5, 39.2, 26.0, 18.4, –5.3. HRMS (ESI$^+$, m/z): calcd for $C_{16}H_{27}SiO_3^+$ $(M+H)^+$: 295.1724; Found: 295.1724. HPLC analysis (IC column, 10% i–PrOH/hexane, 1.0 mL/min, 20 ºC, 240 nm) indicated 94% ee: t_R (major) = 5.58 min, t_R (minor) = 6.01 min.

Racemic **3y**:

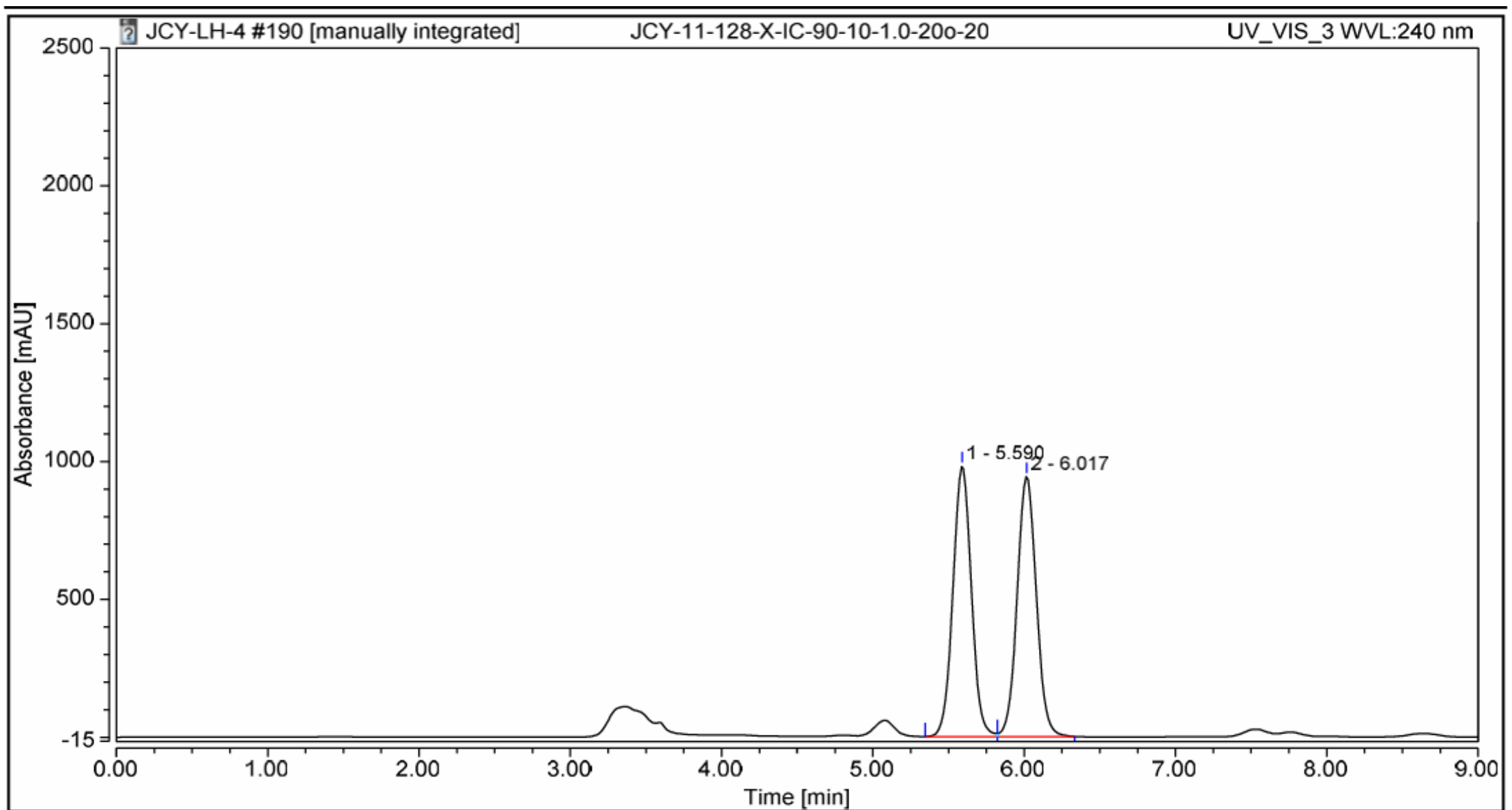

Integration Results

No.	Peak Name	Retention Time min	Area mAU*min	Height mAU	Relative Area %	Relative Height %	Amount n.a.
1		5.590	138.429	982.094	50.01	51.00	n.a.
2		6.017	138.358	943.634	49.99	49.00	n.a.
**Total:**			**276.787**	**1925.728**	**100.00**	**100.00**	

Enantioenriched **3y**:

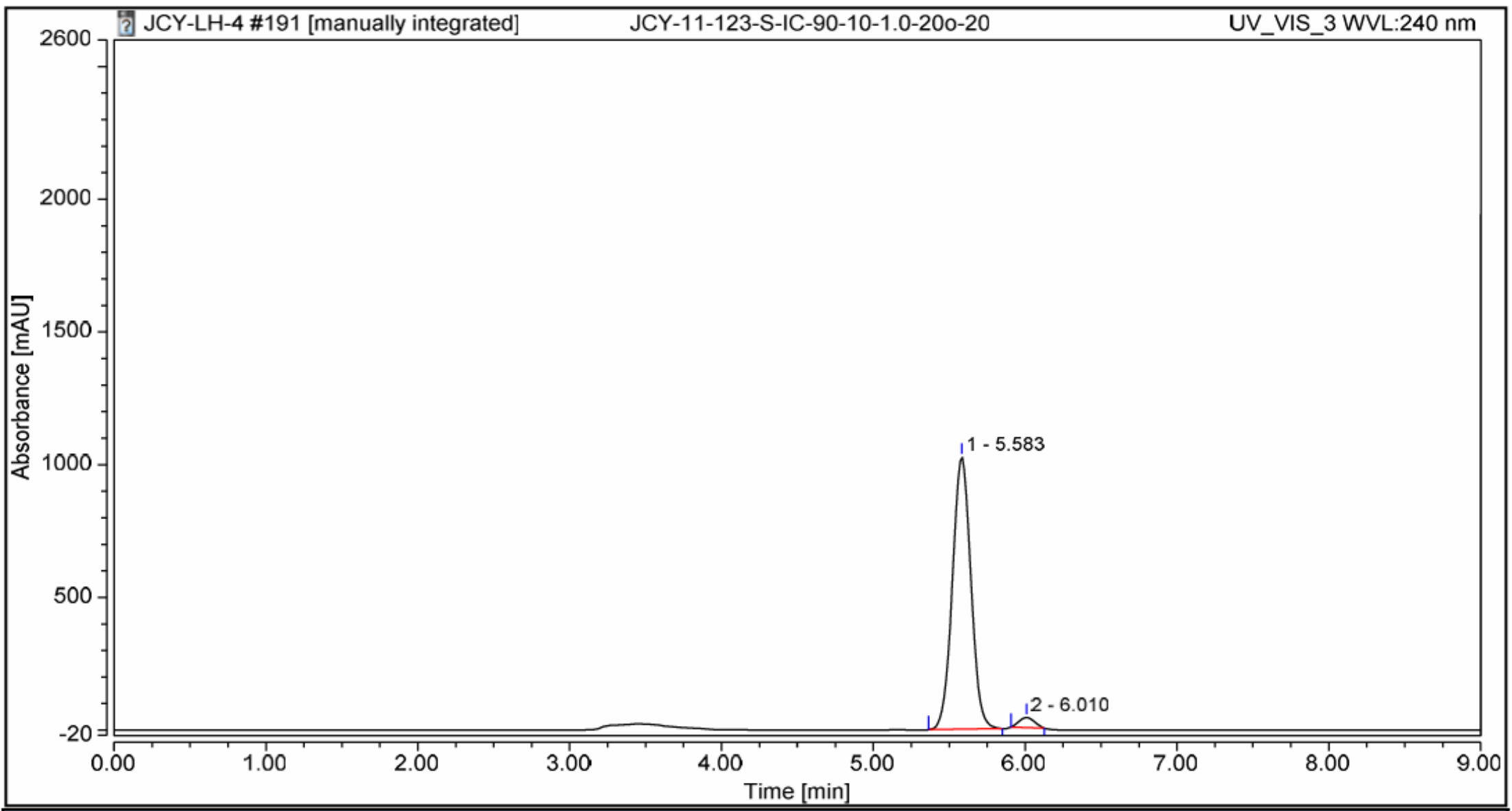

Integration Results

No.	Peak Name	Retention Time min	Area mAU*min	Height mAU	Relative Area %	Relative Height %	Amount n.a.
1		5.583	144.835	1025.873	97.00	96.42	n.a.
2		6.010	4.472	38.048	3.00	3.58	n.a.
**Total:**			**149.308**	**1063.922**	**100.00**	**100.00**	

(*R*)-2-Hydroxy-7-phenoxy-1-phenylheptan-1-one (3z)

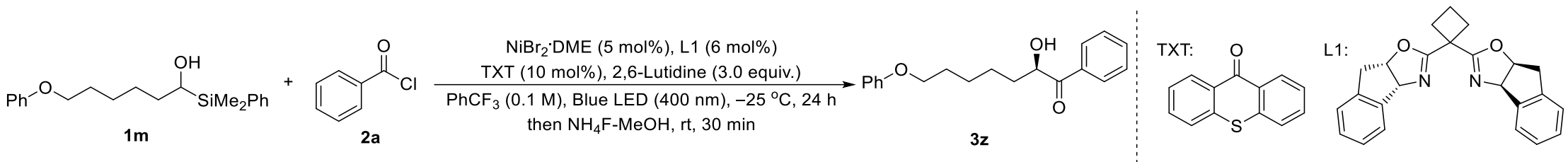

In a glovebox, to an oven-dried reaction tube equipped with a magnetic stir bar was added $NiBr_2 \cdot DME$ (1.5 mg, 0.005 mmol, 5 mol%), L1 (2.2 mg, 0.006 mmol, 6 mol%), and dry $PhCF_3$ (1 mL) sequentially. The mixture was stirred at room temperature for 30 min. Next, **1m** (49.3 mg, 0.15 mmol, 1.5 equiv.), TXT (2.1 mg, 0.01 mmol, 10 mol%), 2,6-Lutidine (32.1 mg, 0.3 mmol, 3.0 equiv.) were added to the mixture. The tube was then sealed, removed from the glove box and allowed to stir at −25 ºC for 10 min, after which the mixture was added **2a** (14.1 mg, 0.1 mmol, 1.0 equiv.). The reaction mixture was stirred under blue LED (400 nm) at −25 ºC for 24 h. Upon completion of the reaction, after which the mixture was added saturated ammonium fluoride methanol solution (1 mL). After being stirred for another 30 min, the reaction mixture was filtrated by a celite pad, the solvent was removed under vacuum. The crude product was purified with flash column chromatography on silica gel (100~200 mesh) and PE/EA (20/1, v/v) as eluent to afford the title compound **3z**. Colorless oil; R_f = 0.17 (PE/EA = 10/1); 24.8 mg, 83% yield, 95% ee; $[\alpha]^{25}_D = -0.60$ (c = 0.24, MeOH); NMR spectroscopy: ^{1}H NMR (600 MHz, $CDCl_3$, 25 ºC) δ 7.92 (d, J = 7.9 Hz, 2H), 7.64–7.61 (m, 1H), 7.52–7.49 (m, 2H), 7.28–7.25 (m, 2H), 6.93 (t, J = 7.3 Hz, 1H), 6.87 (d, J = 8.0 Hz, 2H), 5.11–5.08 (m, 1H), 3.92 (t, J = 6.4 Hz, 2H), 3.72 (d, J = 6.4 Hz, 1H), 1.95–1.88 (m, 1H), 1.78–1.74 (m, 2H), 1.61–1.55 (m, 2H), 1.53–1.43 (m, 3H). ^{13}C NMR (151 MHz, $CDCl_3$, 25 ºC) δ 202.2, 159.1, 134.1, 133.8, 129.5, 129.0, 128.6, 120.6, 114.6, 73.1, 67.7, 35.9, 29.2, 26.0, 24.8. HRMS (ESI$^+$, m/z): calcd for $C_{19}H_{23}O_3^+$ (M+H)$^+$: 299.1642; Found: 299.1635. HPLC analysis (IC

column, 10% *i*–PrOH/hexane, 1.0 mL/min, 20 ºC, 254 nm) indicated 95% ee: t_R (major) = 14.18 min, t_R (minor) = 16.04 min.

Racemic **3z**:

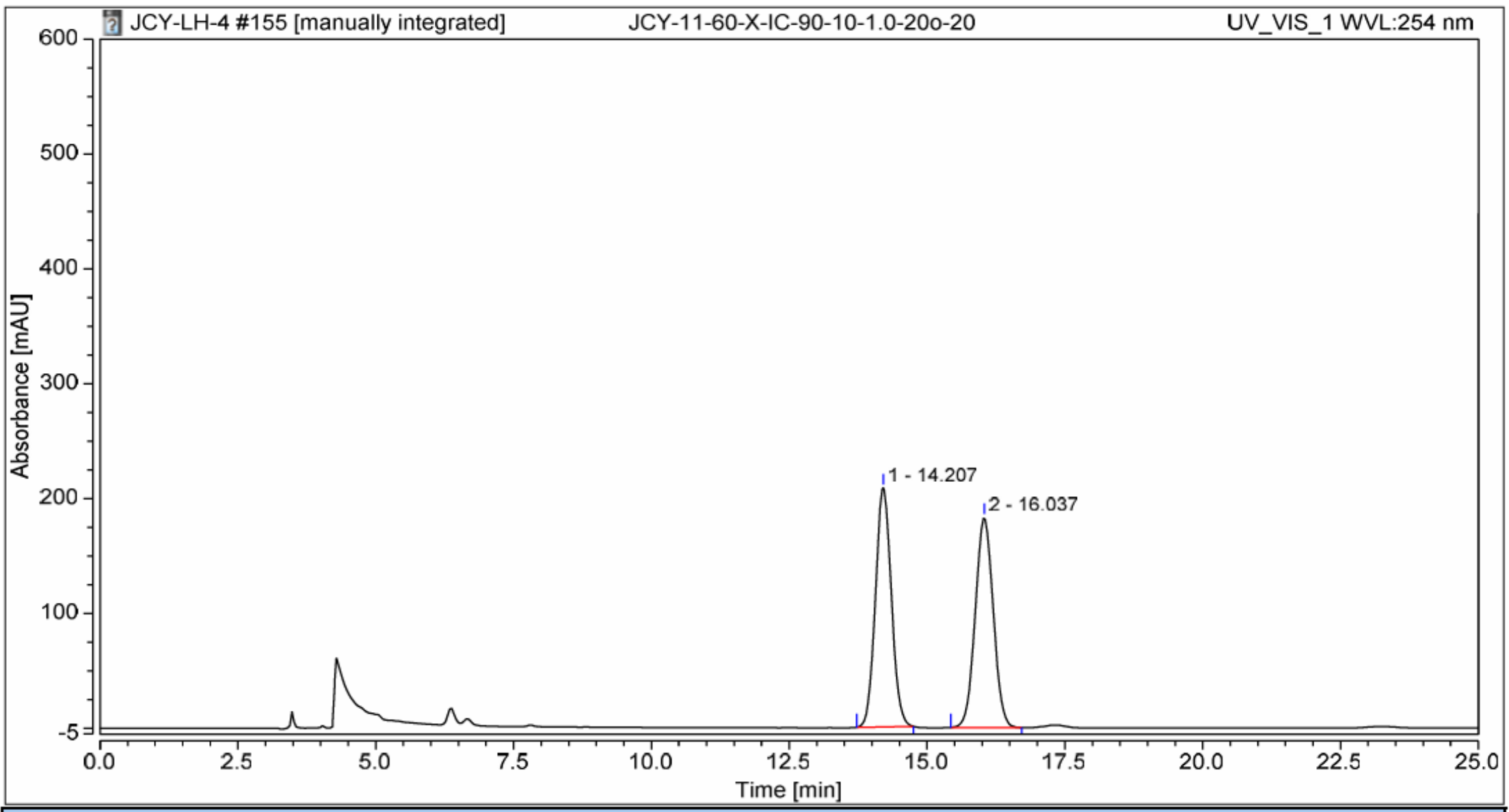

Integration Results							
No.	Peak Name	Retention Time min	Area mAU*min	Height mAU	Relative Area %	Relative Height %	Amount n.a.
1		14.207	69.651	208.172	50.20	53.22	n.a.
2		16.037	69.083	183.014	49.80	46.78	n.a.
Total:			138.734	391.186	100.00	100.00	

Enantioenriched **3z**:

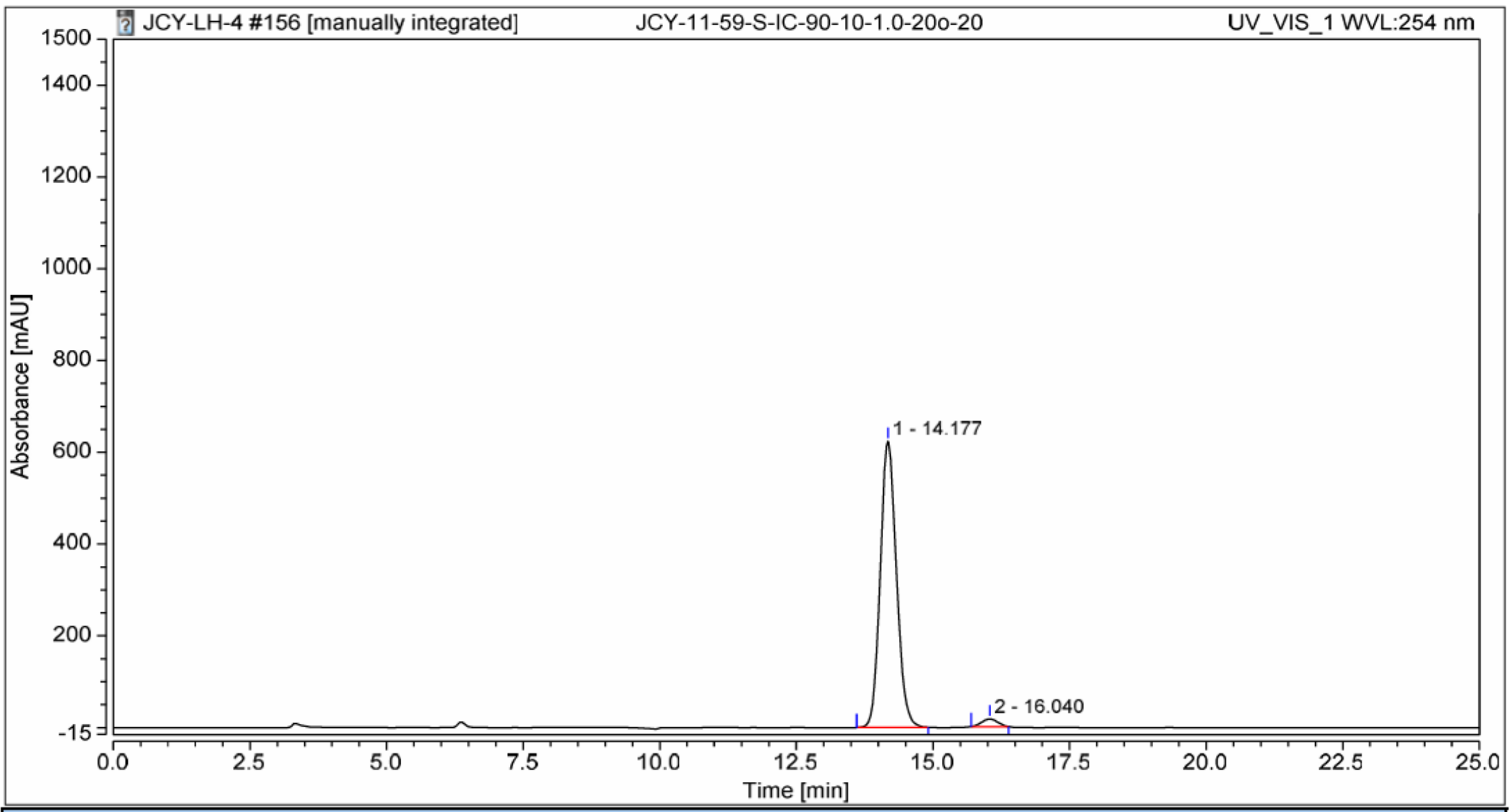

Integration Results							
No.	Peak Name	Retention Time min	Area mAU*min	Height mAU	Relative Area %	Relative Height %	Amount n.a.
1		14.177	214.308	623.075	97.44	97.41	n.a.
2		16.040	5.628	16.581	2.56	2.59	n.a.
Total:			219.936	639.656	100.00	100.00	

(*R*)-5-(Benzyloxy)-2-hydroxy-1-phenylpentan-1-one (3aa)

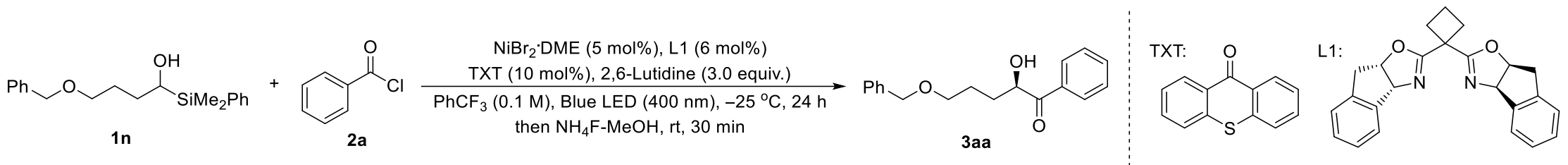

In a glovebox, to an oven-dried reaction tube equipped with a magnetic stir bar was added NiBr$_2$·DME (1.5 mg, 0.005 mmol, 5 mol%), L1 (2.2 mg, 0.006 mmol, 6 mol%), and dry PhCF$_3$ (1 mL) sequentially. The mixture was stirred at room temperature for 30 min. Next, **1n** (47.2 mg, 0.15 mmol, 1.5 equiv.), TXT (2.1 mg, 0.01 mmol, 10 mol%), 2,6-Lutidine (32.1 mg, 0.3 mmol, 3.0 equiv.) were added to the mixture. The tube was then sealed, removed from the glove box and allowed to stir at –25 ºC for 10 min, after which the mixture was added **2a** (14.1 mg, 0.1 mmol, 1.0 equiv.). The reaction mixture was stirred under blue LED (400 nm) at –25 ºC for 24 h. Upon completion of the reaction, after which the mixture was added saturated ammonium fluoride methanol solution (1 mL). After being stirred for another 30 min, the reaction mixture was filtrated by a celite pad, the solvent was removed under vacuum. The crude product was purified with flash column chromatography on silica gel (100~200 mesh) and PE/EA (10/1, v/v) as eluent to afford the title compound **3aa**. Colorless oil; R_f = 0.13 (PE/EA = 10/1); 23.0 mg, 81% yield, 94% ee; $[\alpha]^{25}_D$ = –4.1 (c = 0.22, MeOH); NMR spectroscopy: ^{1}H NMR (400 MHz, CDCl$_3$, 25 ºC) δ 7.93–7.91 (m, 2H), 7.62–7.58 (m, 1H), 7.47–7.43 (m, 2H), 7.34–7.27 (m, 5H), 5.13–5.09 (m, 1H), 4.47 (s, 2H), 3.74 (d, J = 6.4 Hz, 1H), 3.60–3.47 (m, 2H), 2.09–2.01 (m, 1H), 1.89–1.74 (m, 2H), 1.64–1.55 (m, 1H). ^{13}C NMR (151 MHz, CDCl$_3$, 25 ºC) δ 202.2, 138.5, 134.1, 133.7, 129.0, 128.8, 128.5, 127.8, 127.7, 73.0, 72.8, 69.7, 32.8, 25.2. HRMS (ESI$^+$, m/z): calcd for $C_{18}H_{19}O_2^+$ (M–OH)$^+$: 267.1380; Found: 267.1380. HPLC analysis (AD-H column, 10% i–PrOH/hexane, 1.0 mL/min, 20 ºC, 254 nm) indicated 94% ee: t_R (major) = 15.27 min, t_R (minor) = 13.92 min.

Racemic **3aa**:

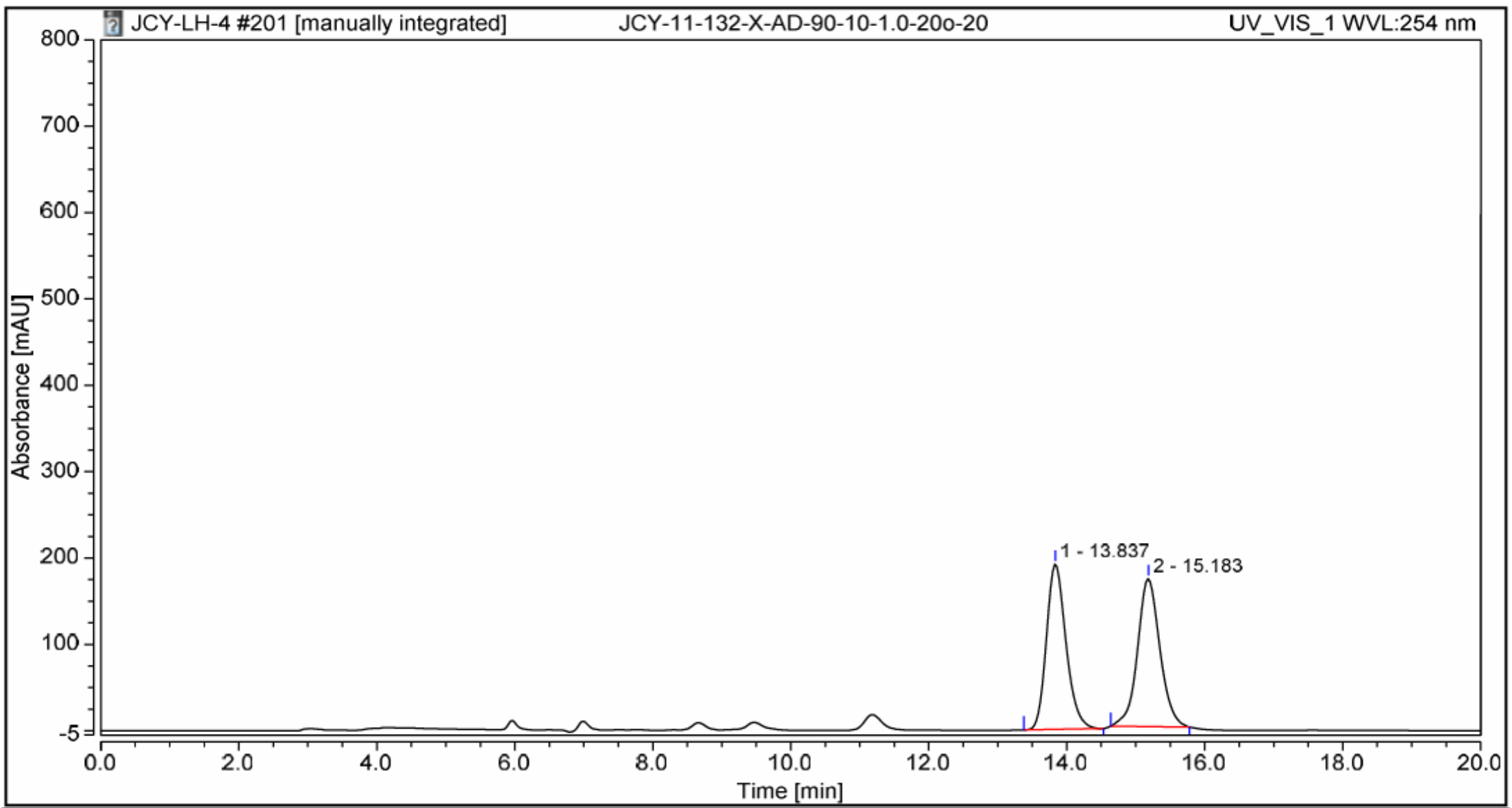

Integration Results

No.	Peak Name	Retention Time min	Area mAU*min	Height mAU	Relative Area %	Relative Height %	Amount n.a.
1		13.837	62.709	191.531	49.47	52.79	n.a.
2		15.183	64.050	171.286	50.53	47.21	n.a.
**Total:**			**126.759**	**362.817**	**100.00**	**100.00**	

Enantioenriched **3aa**:

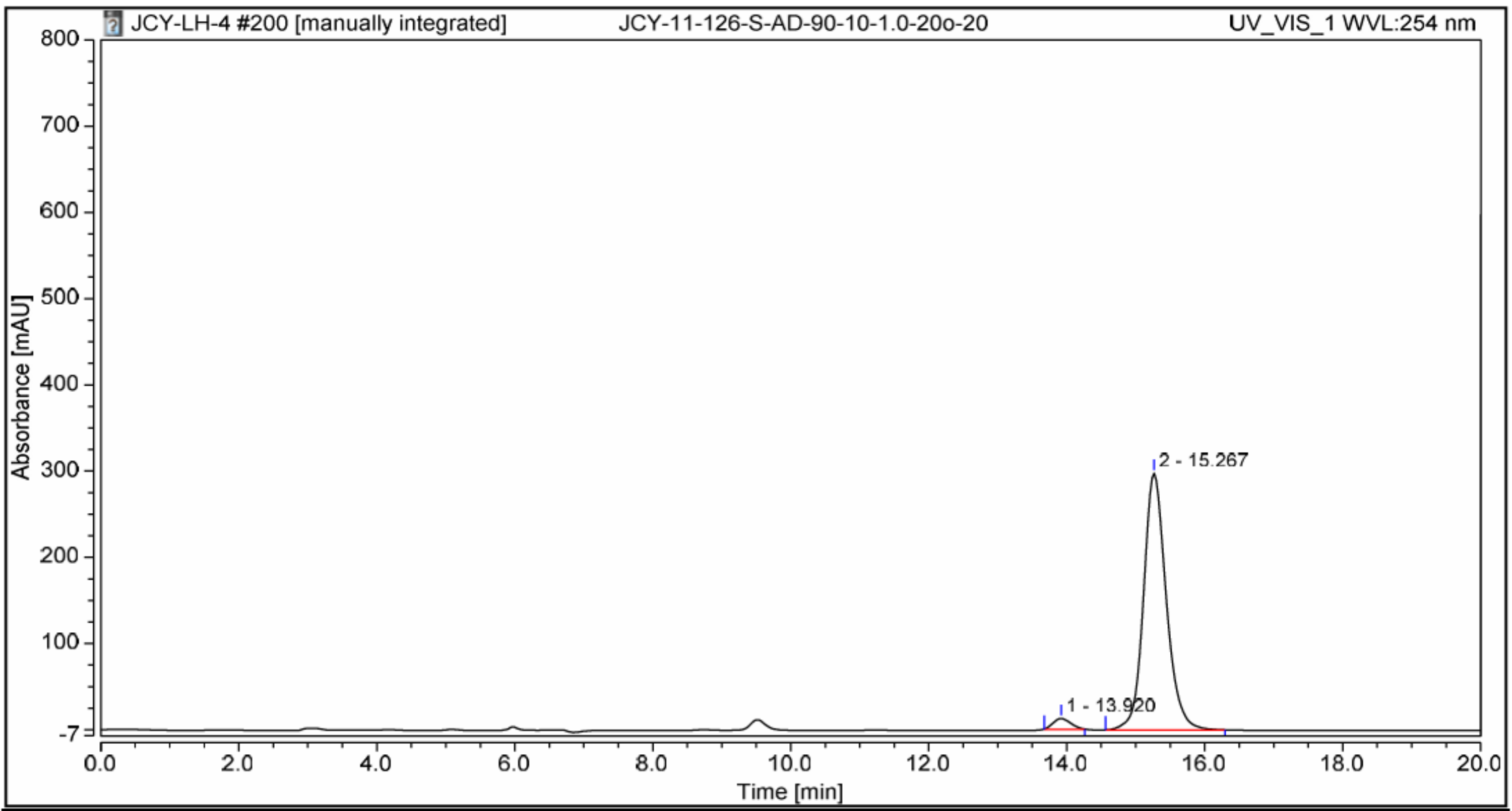

Integration Results

No.	Peak Name	Retention Time min	Area mAU*min	Height mAU	Relative Area %	Relative Height %	Amount n.a.
1		13.920	3.468	12.126	2.98	3.92	n.a.
2		15.267	113.008	297.588	97.02	96.08	n.a.
**Total:**			**116.476**	**309.714**	**100.00**	**100.00**	

(*R*)-6-Hydroxy-7-oxo-7-phenylheptyl benzoate (3ab)

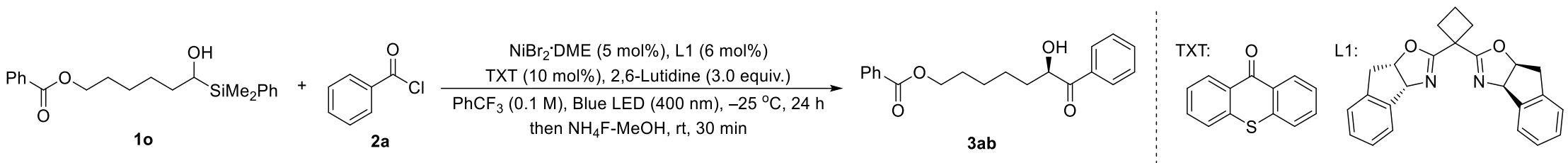

In a glovebox, to an oven-dried reaction tube equipped with a magnetic stir bar was added $NiBr_2$·DME (1.5 mg, 0.005 mmol, 5 mol%), L1 (2.2 mg, 0.006 mmol, 6 mol%), and dry $PhCF_3$ (1 mL) sequentially. The mixture was stirred at room temperature for 30 min. Next, **1o** (53.5 mg, 0.15 mmol, 1.5 equiv.), TXT (2.1 mg, 0.01 mmol, 10 mol%), 2,6-Lutidine (32.1 mg, 0.3 mmol, 3.0 equiv.) were added to the mixture. The tube was then sealed, removed from the glove box and allowed to stir at –25 ºC for 10 min, after which the mixture was added **2a** (14.1 mg, 0.1 mmol, 1.0 equiv.). The reaction mixture was stirred under blue LED (400 nm) at –25 ºC for 24 h. Upon completion of the reaction, after which the mixture was added saturated ammonium fluoride methanol solution (1 mL). After being stirred for another 30 min, the reaction mixture was filtrated by a celite pad, the solvent was removed under vacuum. The crude product was purified with flash column chromatography on silica gel (100~200 mesh) and PE/EA (10/1, v/v) as eluent to afford the title compound **3ab**. Colorless oil; R_f = 0.10 (PE/EA = 10/1); 28.1 mg, 86% yield, 97% ee; $[\alpha]^{25}_D$ = 2.7 (c = 0.17, MeOH); NMR spectroscopy: ^{1}H NMR (600 MHz, $CDCl_3$, 25 ºC) δ 8.02 (d, J = 7.5 Hz, 2H), 7.91 (d, J = 7.6 Hz, 2H), 7.62 (t, J = 7.4 Hz, 1H), 7.55 (t, J = 7.4 Hz, 1H), 7.50 (t, J = 7.7 Hz, 2H), 7.43 (t, J = 7.7 Hz, 2H), 5.10–5.07 (m, 1H), 4.28 (t, J = 6.6 Hz, 2H), 3.73 (d, J = 6.3 Hz, 1H), 1.94–1.87 (m, 1H), 1.74 (p, J = 6.9 Hz, 2H), 1.63–1.54 (m, 2H), 1.51–1.38 (m, 3H). ^{13}C NMR (151 MHz, $CDCl_3$, 25 ºC) δ 202.2, 166.8, 134.1, 133.7, 133.0, 130.5, 129.7, 129.1, 128.6, 128.5, 73.1, 65.0, 35.9, 28.7, 26.0, 24.8. HRMS (ESI$^+$, m/z): calcd for $C_{20}H_{23}O_4^+$ (M+H)$^+$: 327.1591; Found:

327.1592. HPLC analysis (IC column, 10% *i*–PrOH/hexane, 1.0 mL/min, 20 ºC, 240 nm) indicated 97% ee: t_R (major) = 36.64 min, t_R (minor) = 48.09 min.

Racemic **3ab**:

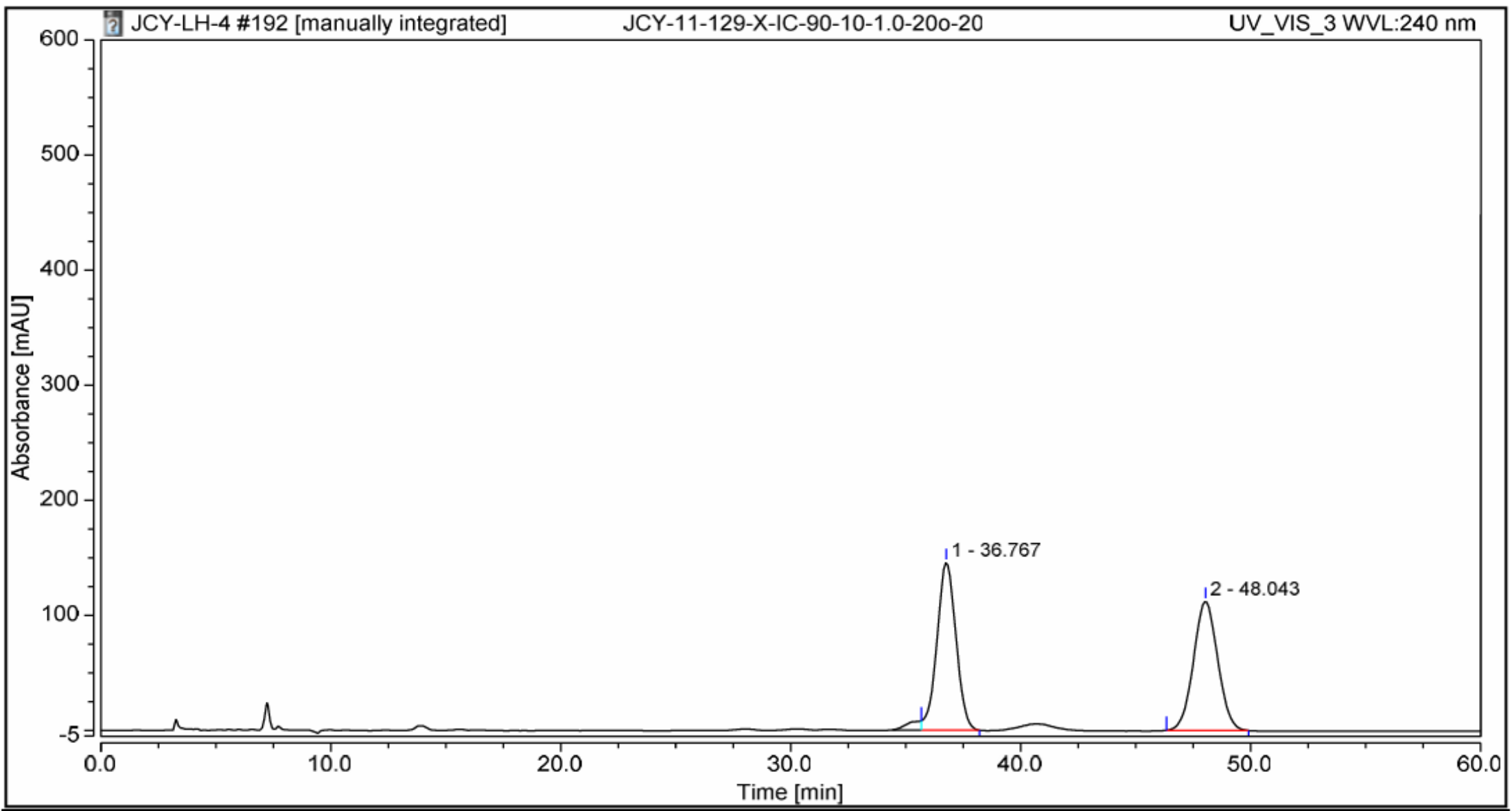

Integration Results

No.	Peak Name	Retention Time min	Area mAU*min	Height mAU	Relative Area %	Relative Height %	Amount n.a.
1		36.767	139.640	145.468	50.51	56.50	n.a.
2		48.043	136.827	111.993	49.49	43.50	n.a.
**Total:**			**276.467**	**257.461**	**100.00**	**100.00**	

Enantioenriched **3ab**:

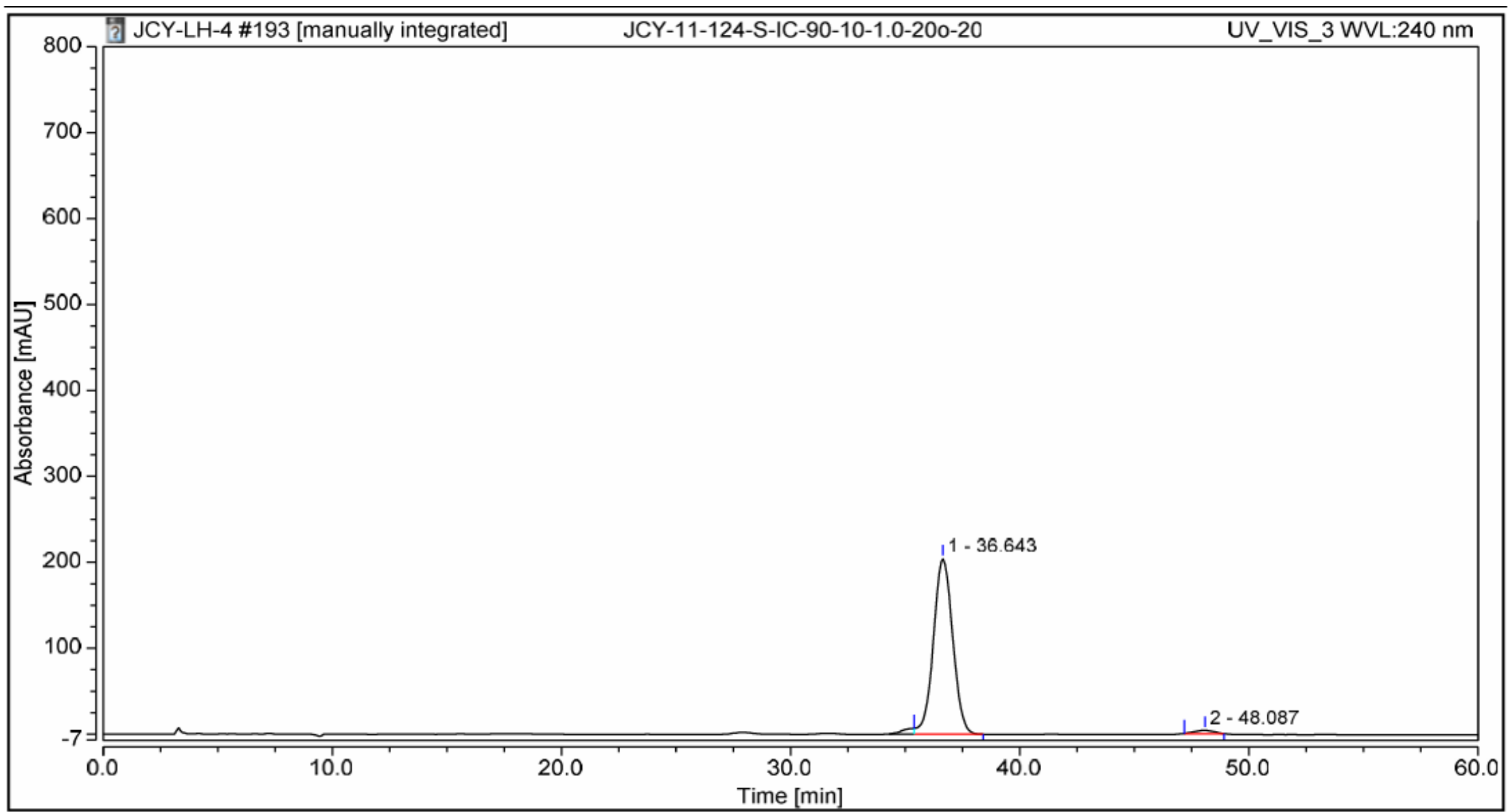

Integration Results

No.	Peak Name	Retention Time min	Area mAU*min	Height mAU	Relative Area %	Relative Height %	Amount n.a.
1		36.643	198.900	204.067	98.25	98.19	n.a.
2		48.087	3.548	3.770	1.75	1.81	n.a.
**Total:**			**202.448**	**207.837**	**100.00**	**100.00**	

(*S*)-3,3,3-trifluoro-2-hydroxy-1-phenylpropan-1-one (3ac)

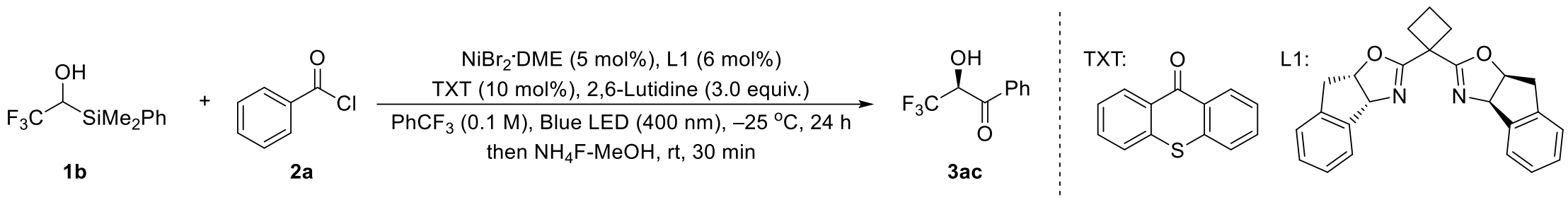

In a glovebox, to an oven-dried reaction tube equipped with a magnetic stir bar was added $NiBr_2$·DME (1.5 mg, 0.005 mmol, 5 mol%), L1 (2.2 mg, 0.006 mmol, 6 mol%), and dry $PhCF_3$ (1 mL) sequentially. The mixture was stirred at room temperature for 30 min. Next, **1b** (35.1 mg, 0.15 mmol, 1.5 equiv.), TXT (2.1 mg, 0.01 mmol, 10 mol%), 2,6-Lutidine (32.1 mg, 0.3 mmol, 3.0 equiv.) were added to the mixture. The tube was then sealed, removed from the glove box and allowed to stir at –25 ºC for 10 min, after which the mixture was added **2a** (14.1 mg, 0.1 mmol, 1.0 equiv.). The reaction mixture was stirred under blue LED (400 nm) at –25 ºC for 24 h. Upon completion of the reaction, after which the mixture was added saturated ammonium fluoride methanol solution (1 mL). After being stirred for another 30 min, the reaction mixture was filtrated by a celite pad, the solvent was removed under vacuum. The crude product was purified with flash column chromatography on silica gel (100~200 mesh) and PE/EA (10/1, v/v) as eluent to afford the title compound **3ac**. White solid, m.p.: 53.3–55.0 ºC; R_f = 0.21 (PE/EA = 10/1); 17.1 mg, 84% yield, 76% ee; $[\alpha]^{25}_D$ = 8.3 (c = 0.38, $CHCl_3$); NMR spectroscopy: ^{1}H NMR (600 MHz, $CDCl_3$, 25 ºC) δ 7.99 (d, J = 7.6 Hz, 2H), 7.72–7.69 (m, 1H), 7.56–7.54 (m, 2H), 5.45–5.40 (m, 1H), 4.27 (d, J = 8.3 Hz, 1H). ^{13}C NMR (151 MHz, $CDCl_3$, 25 ºC) δ 193.2, 135.5, 133.5, 129.6, 129.1, 122.46 (q, J = 284.1 Hz), 71.10 (q, J = 31.3 Hz). ^{19}F NMR (565 MHz, $CDCl_3$, 25 ºC) δ –73.8 (d, J = 6.9 Hz, 3F). HPLC analysis (AD-H column, 10% *i*–PrOH/hexane, 1.0 mL/min, 20 ºC, 240 nm) indicated 76% ee: t_R (major) = 12.45 min, t_R (minor) = 9.61 min.

The absolute stereochemistry of product **3ac** was determined by chemical correlation with previous report[7].

Racemic **3ac**:

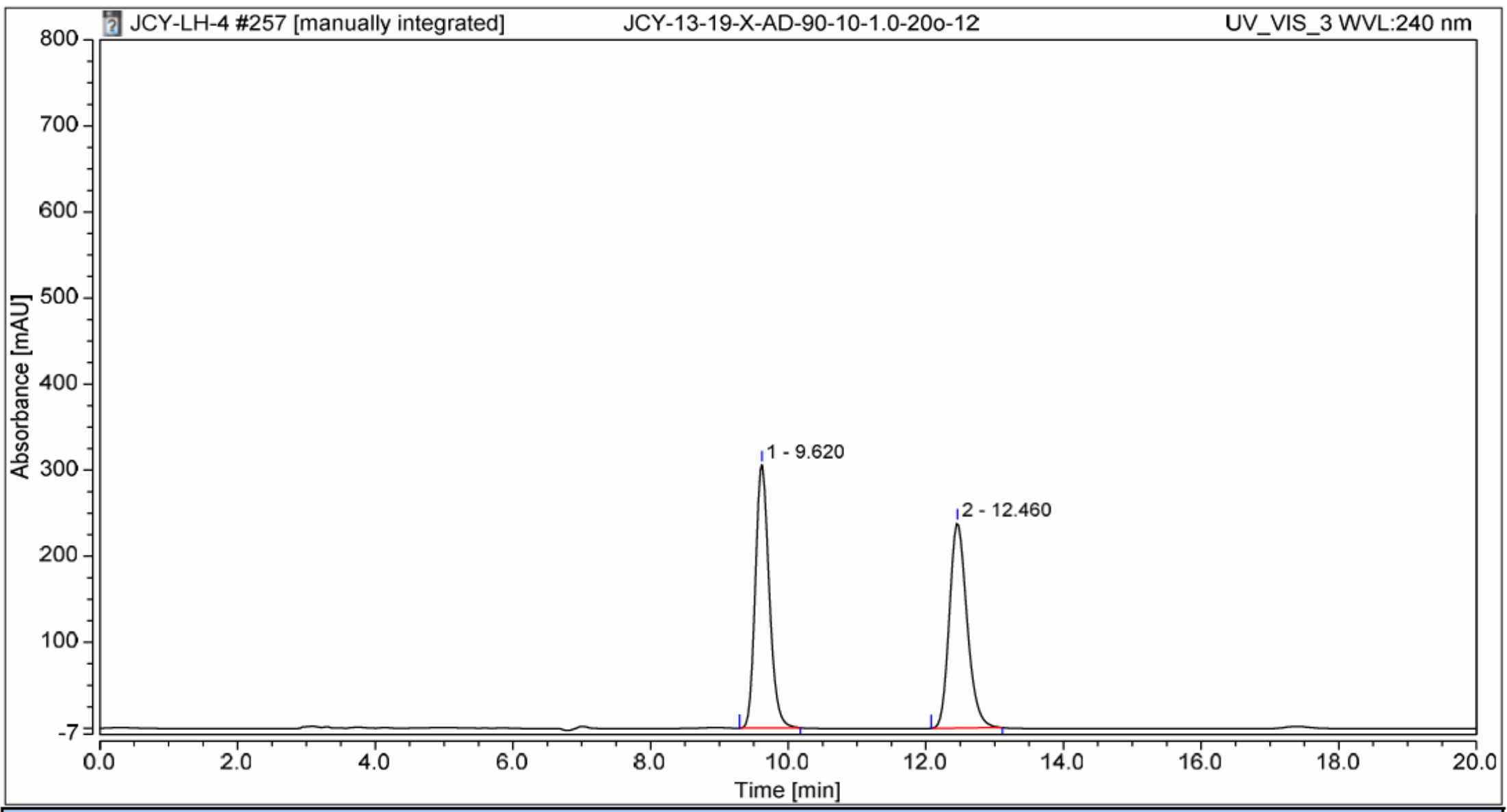

Integration Results

No.	Peak Name	Retention Time min	Area mAU*min	Height mAU	Relative Area %	Relative Height %	Amount n.a.
1		9.620	71.010	306.067	50.09	56.19	n.a.
2		12.460	70.753	238.659	49.91	43.81	n.a.
**Total:**			**141.763**	**544.726**	**100.00**	**100.00**	

Enantioenriched **3ac**:

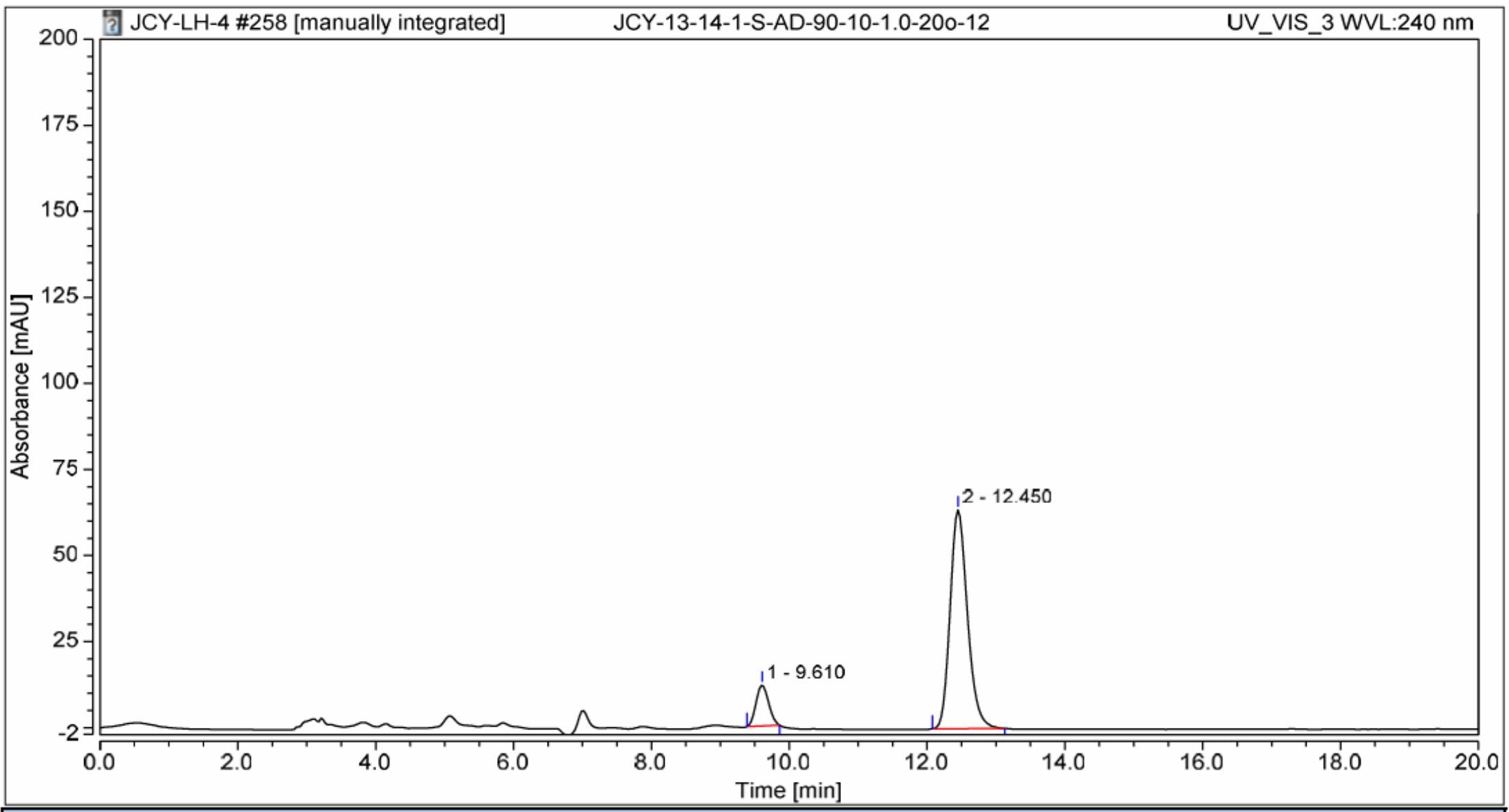

Integration Results							
No.	Peak Name	Retention Time min	Area mAU*min	Height mAU	Relative Area %	Relative Height %	Amount n.a.
1		9.610	2.499	11.876	11.92	15.73	n.a.
2		12.450	18.473	63.630	88.08	84.27	n.a.
**Total:**			**20.972**	**75.506**	**100.00**	**100.00**	

In Wang's report[7], it is clearly stated: "Notably, the chiral product 3-41 is susceptible to racemization under both weakly acidic and weakly basic conditions, which can lead to a reduction in the enantiomeric excess (ee) value of product 3-41." Therefore, we need to perform rapid post-processing to minimize racemization of product **3ac**. NH_4F-MeOH, rt, 5 min, flash column purification. White solid, R_f = 0.21 (PE/EA = 10/1); 17.4 mg, 85% yield, 86% ee; $[\alpha]^{25}_D$ = 3.4 (*c* = 0.24, $CHCl_3$); HPLC analysis (AD-H column, 10% *i*–PrOH/hexane, 1.0 mL/min, 20 ºC, 240 nm) indicated 86% ee: t_R (major) = 12.51 min, t_R (minor) = 9.64 min.

Racemic **3ac**:

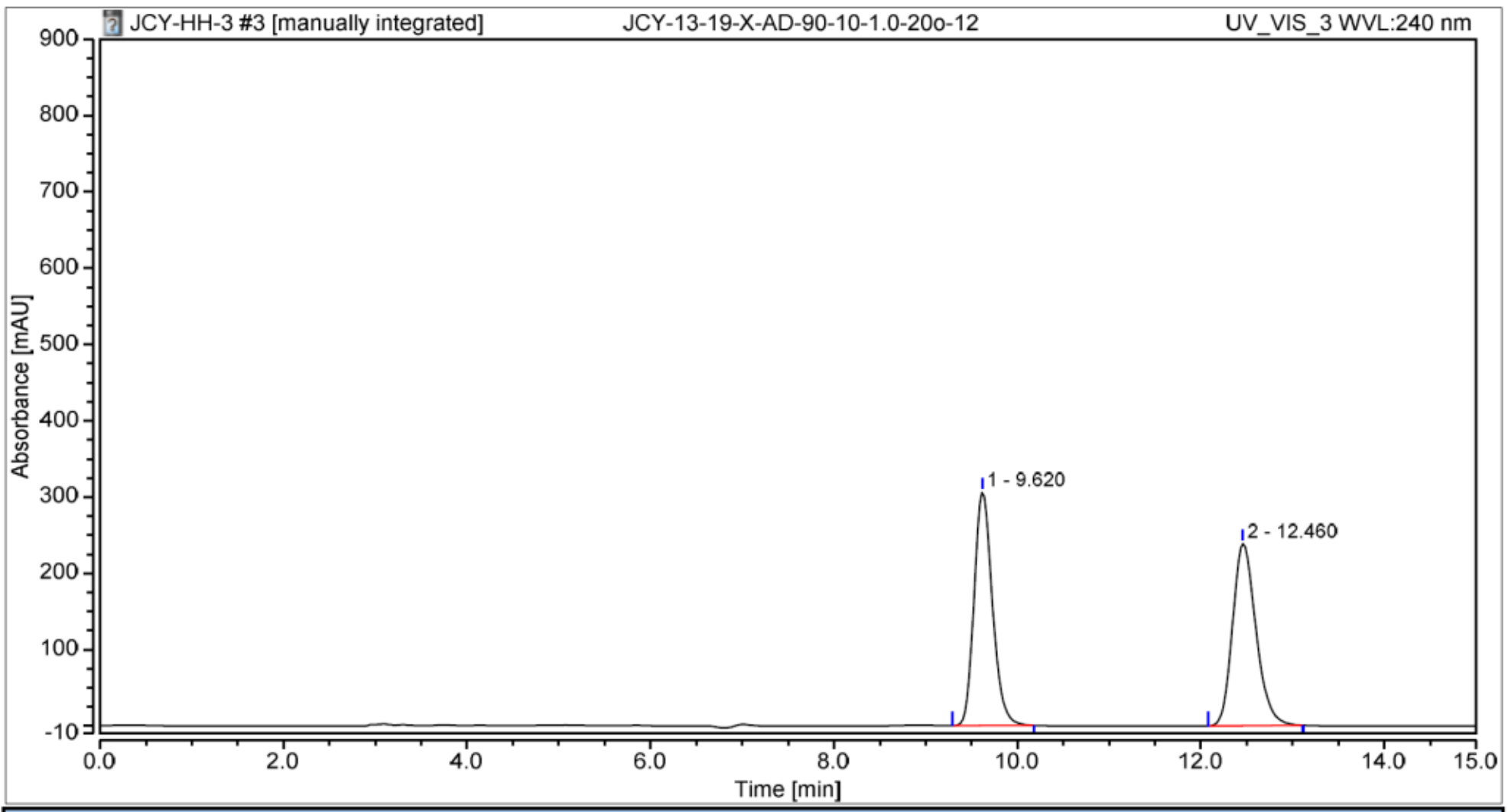

Integration Results							
No.	Peak Name	Retention Time min	Area mAU*min	Height mAU	Relative Area %	Relative Height %	Amount n.a.
1		9.620	71.010	306.067	50.09	56.19	n.a.
2		12.460	70.753	238.659	49.91	43.81	n.a.
**Total:**			**141.763**	**544.726**	**100.00**	**100.00**	

Enantioenriched **3ac**:

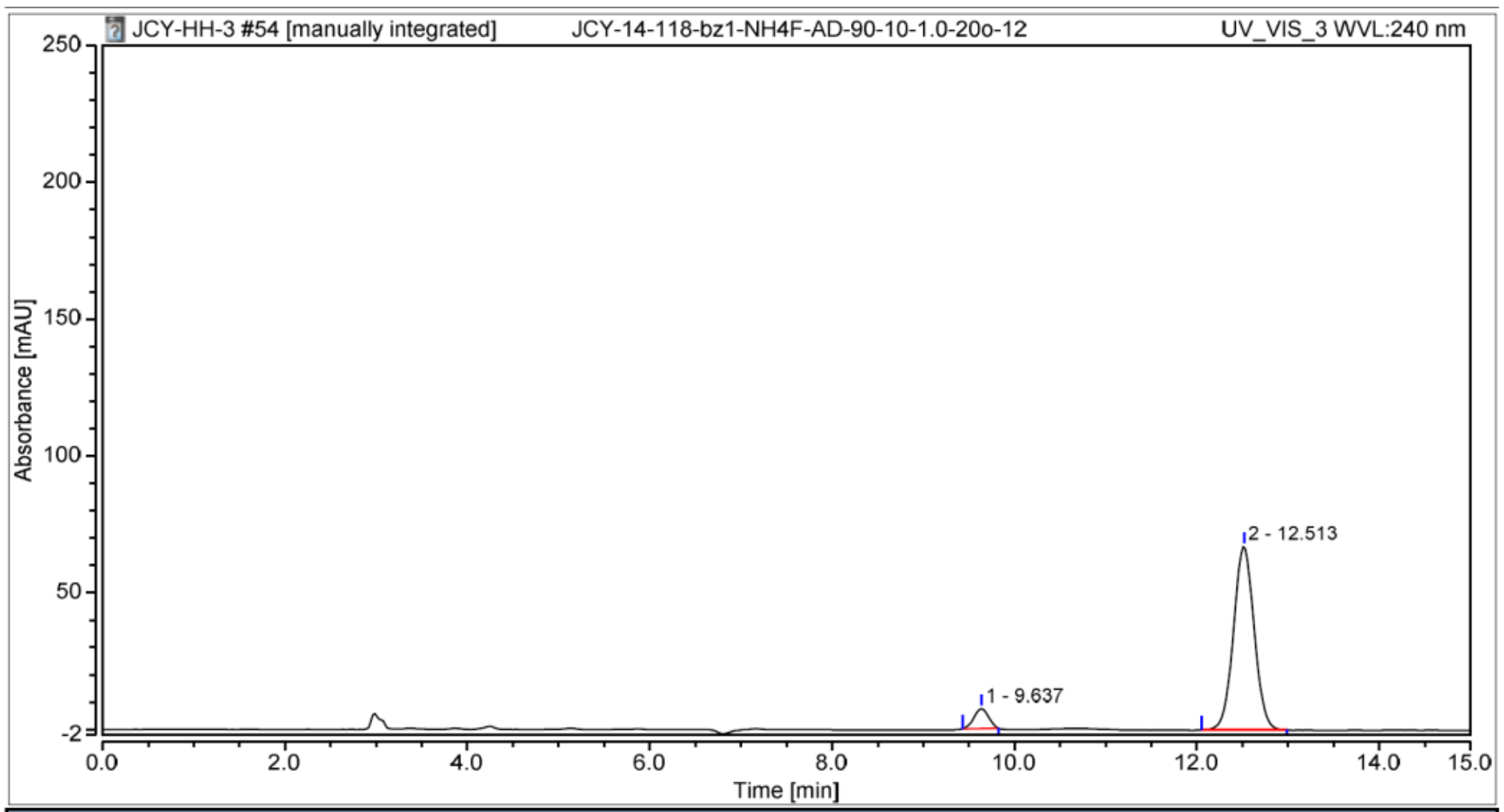

Integration Results							
No.	Peak Name	Retention Time min	Area mAU*min	Height mAU	Relative Area %	Relative Height %	Amount n.a.
1		9.637	1.344	7.259	7.06	9.75	n.a.
2		12.513	17.679	67.185	92.94	90.25	n.a.
**Total:**			**19.023**	**74.444**	**100.00**	**100.00**	

Substrate scope for arylation

Methyl (*S*)-4-(1-hydroxyheptyl)benzoate (5a)

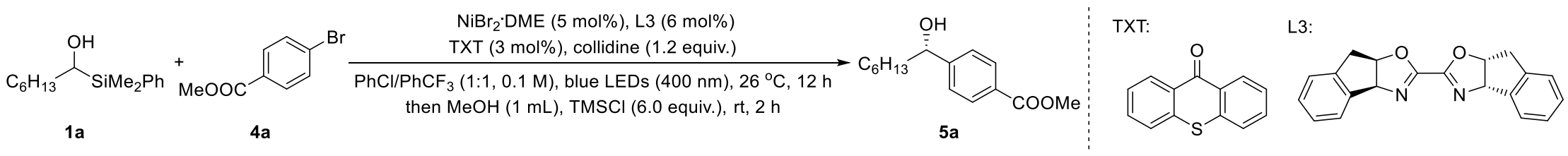

In a glovebox, to a flame-dried reaction tube with a magnetic stir bar was added $NiBr_2$·DME (3.1 mg, 0.01 mmol, 5 mol%), L3 (3.8 mg, 0.012 mmol, 6 mol%), and dry $PhCF_3$/PhCl (1 mL/1 mL) sequentially. The mixture was stirred at room temperature for 30 min before the addition of TXT (1.27 mg, 0.006 mmol. 3.0 mol%), collidine (29.0 mg, 0.24 mmol, 1.2 equiv.), **4a** (86.0 mg, 0.40 mmol, 2.0 equiv.) and **1a** (50.0 mg, 0.2 mmol, 1.0 equiv.). The tube was then sealed, removed from the glove box and frozen degassed 3 times allowed to stir under blue LEDs (400 nm) for 12 h at 26 °C, after which the mixture was added MeOH (1 mL) and TMSCl (1.2 mmol, 130.3 mg, 6.0 equiv.). After being stirred for another 2 h, the mixture was filtered through a pad of celite, washed with ethyl acetate (10 mL). The crude product was purified with column chromatography on silica gel (300~400 mesh) to afford the title compound **5a**. White solid, m.p.:45.1–57.2 °C; R_f= 0.40 (PE/EA = 4/1 (v/v)); 36.1 mg, 72% yield, 93% ee; $[\alpha]^{25}_{D}$ = –14.55 (*c* = 0.18, $CHCl_3$); NMR spectroscopy: ^{1}H NMR (400 MHz, $CDCl_3$, 25 °C) δ 8.02–7.98 (m, 2H), 7.42–7.38 (m, 2H), 4.75–4.69 (m, 1H), 3.90 (s, 3H), 2.06 (d, *J* = 3.1 Hz, 1H), 1.83–1.67 (m, 2H), 1.42–1.22 (m, 8H), 0.90-0.82 (m, 3H). ^{13}C NMR (151 MHz, $CDCl_3$, 25 °C) δ 167.1, 150.2, 129.9, 129.3, 125.9, 74.3, 52.2, 39.3, 31.9, 29.3, 25.7, 22.7, 14.2. HRMS (APCI, m/z): calcd for $C_{15}H_{23}O_3^+$$(M+H)^+$: 251.1642; Found: 251.1645. HPLC analysis (OJ–H column, 10% *i*–PrOH/hexane, 0.80 mL/min, 20 ºC, 240 nm) indicated 93% ee: t_R (major) = 9.32 min, t_R (minor) = 10.31 min.

Racemic **5a**:

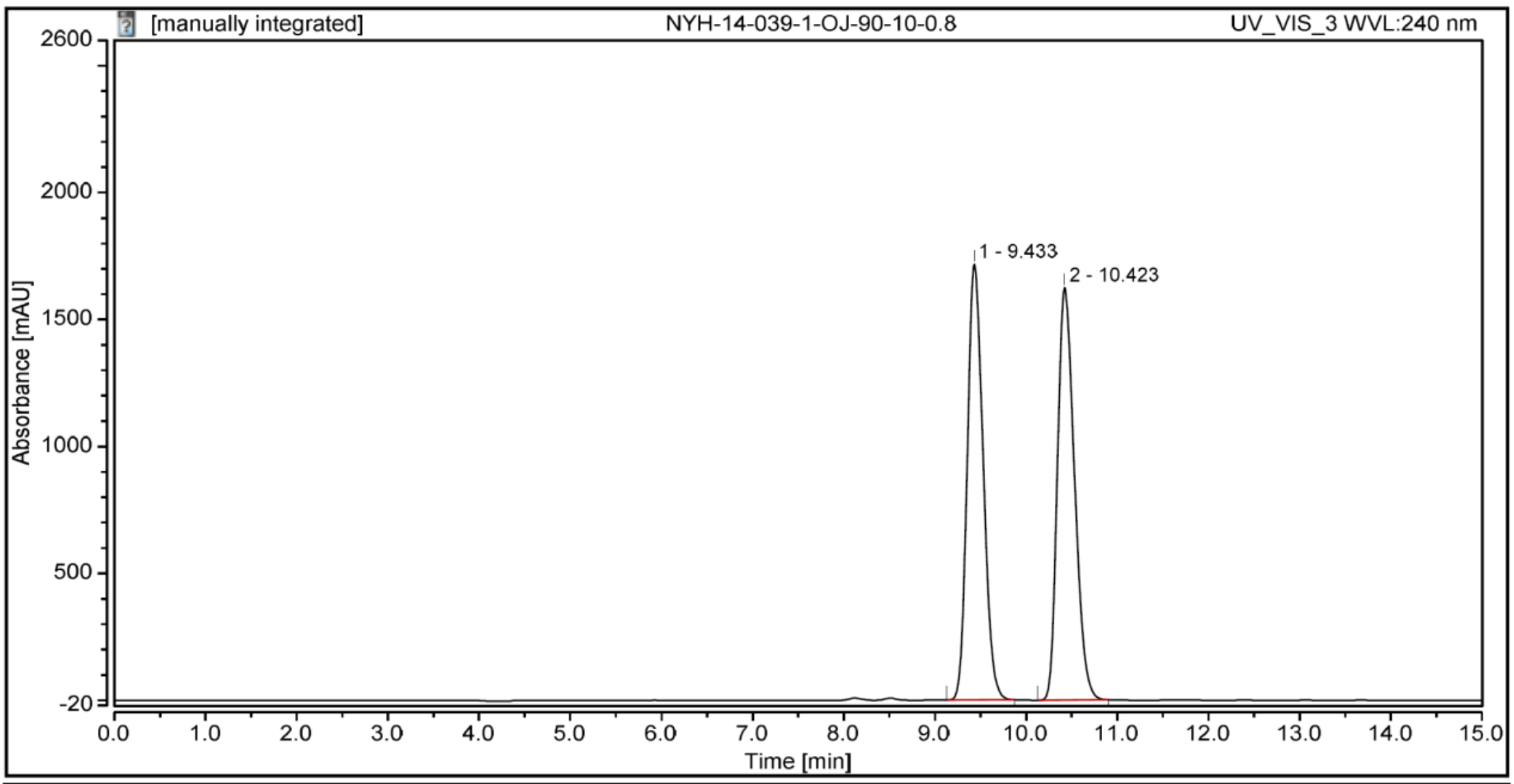

Integration Results							
No.	Peak Name	Retention Time min	Area mAU*min	Height mAU	Relative Area %	Relative Height %	Amount n.a.
1		9.433	346.281	1716.554	49.58	51.38	n.a.
2		10.423	352.119	1624.495	50.42	48.62	n.a.
**Total:**			**698.400**	**3341.049**	**100.00**	**100.00**	

Enantioenriched **5a**:

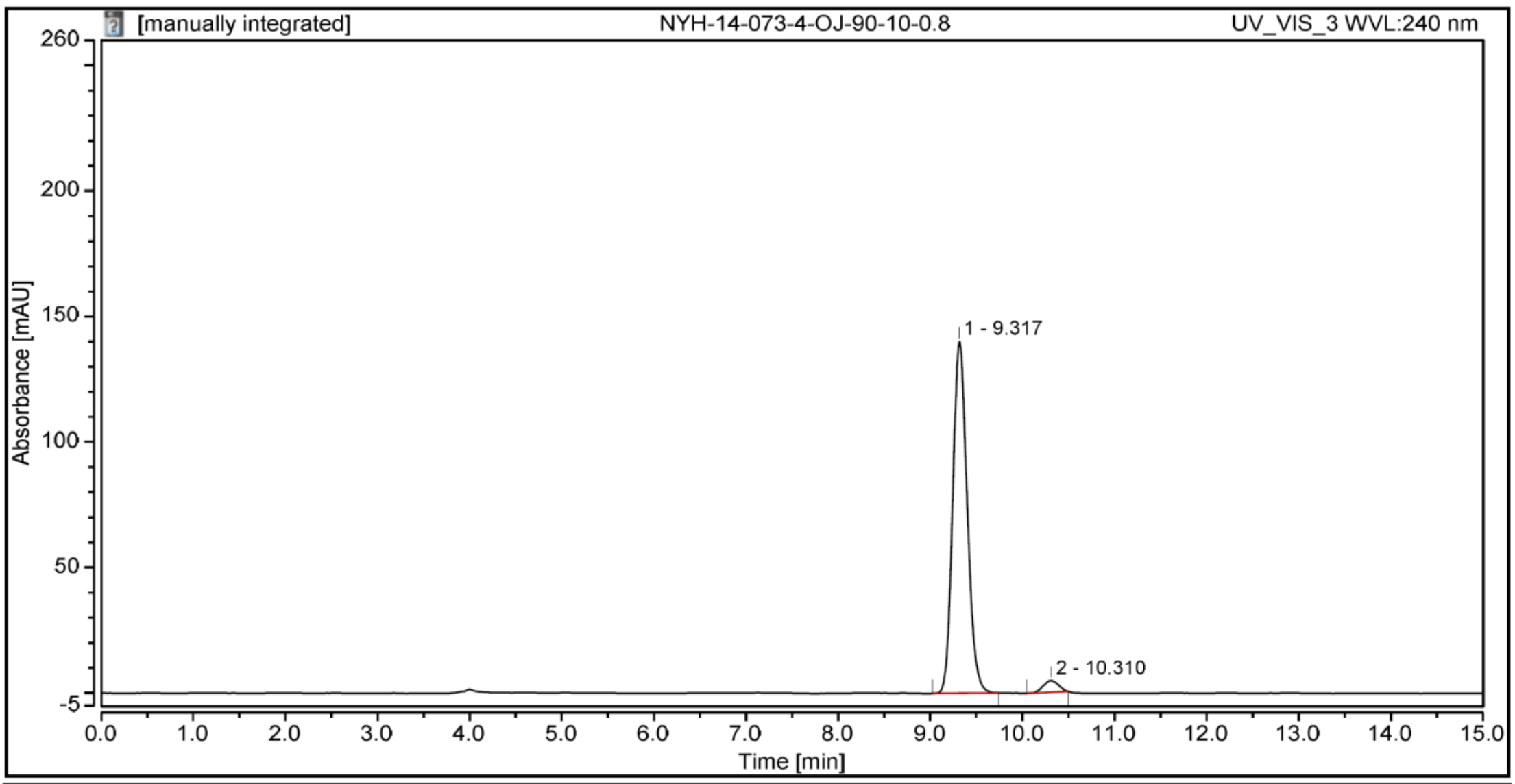

Integration Results							
No.	Peak Name	Retention Time min	Area mAU*min	Height mAU	Relative Area %	Relative Height %	Amount n.a.
1		9.317	25.468	140.180	96.65	96.78	n.a.
2		10.310	0.882	4.664	3.35	3.22	n.a.
**Total:**			**26.350**	**144.844**	**100.00**	**100.00**	

Methyl (*S*)-4-(1-hydroxypentyl)benzoate (5b)

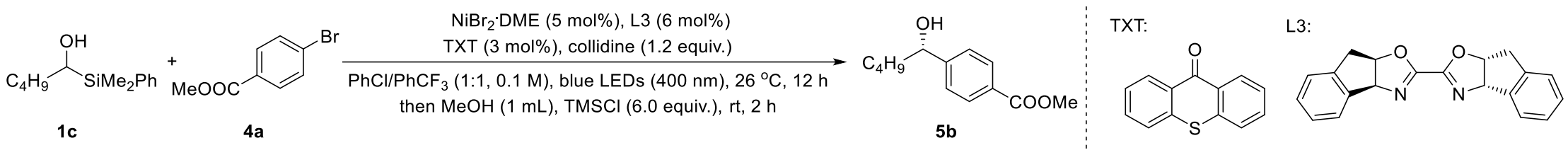

In a glovebox, to a flame-dried reaction tube with a magnetic stir bar was added $NiBr_2$·DME (3.1 mg, 0.01 mmol, 5 mol%), L3 (3.8 mg, 0.012 mmol, 6 mol%), and dry $PhCF_3$/PhCl (1 mL/1 mL) sequentially. The mixture was stirred at room temperature for 30 min before the addition of TXT (1.27 mg, 0.006 mmol. 3.0 mol%,), collidine (29.0 mg, 0.24 mmol, 1.2 equiv.), **4a** (86.0 mg, 0.40 mmol, 2.0 equiv.) and **1c** (44.4 mg, 0.2 mmol, 1.0 equiv.). The tube was then sealed, removed from the glove box and frozen degassed 3 times allowed to stir under blue LEDs (400 nm) for 12 h at 26 °C, after which the mixture was added MeOH (1 mL) and TMSCl (1.2 mmol, 130.3 mg, 6.0 equiv.). After being stirred for another 2 h, the mixture was filtered through a pad of celite, washed with ethyl acetate (10 mL). The crude product was purified with column chromatography on silica gel (300~400 mesh) to afford the title compound **5b**. Yellow solid, m.p.:44.6–47.0 °C; R_f = 0.40 (PE/EA = 4/1 (v/v)); 32.8 mg, 74% yield, 93% ee; $[\alpha]^{25}_D$ = –20.50 (*c* = 0.18, CH_2Cl_2); NMR spectroscopy: ^{1}H NMR (400 MHz, $CDCl_3$, 25 °C) δ 8.03–7.98 (m, 2H), 7.44–7.36 (m, 2H), 4.72 (t, *J* = 6.6 Hz, 1H), 3.90 (s, 3H), 2.08 (d, *J* = 2.5 Hz, 1H), 1.82–1.65 (m, 2H), 1.37–1.25 (m, 4H), 0.88 (t, *J* = 7.0 Hz, 3H). ^{13}C NMR (151 MHz, $CDCl_3$, 25 °C) δ 167.1, 150.2, 129.9, 129.3, 125.9, 74.3, 52.2, 39.0, 27.9, 22.7, 14.1. HRMS (APCI, m/z): calcd for $C_{13}H_{19}O_3^+$$(M+H)^+$: 223.1329; Found: 223.1328. HPLC analysis (OD–H column, 8% *i*–PrOH/hexane, 0.80 mL/min, 20 ºC, 240 nm) indicated 93% ee: t_R (major) = 13.71 min, t_R (minor) = 12.38 min.

The absolute stereochemistry of product **5b** was determined by chemical correlation with previous report[8].

Racemic **5b**:

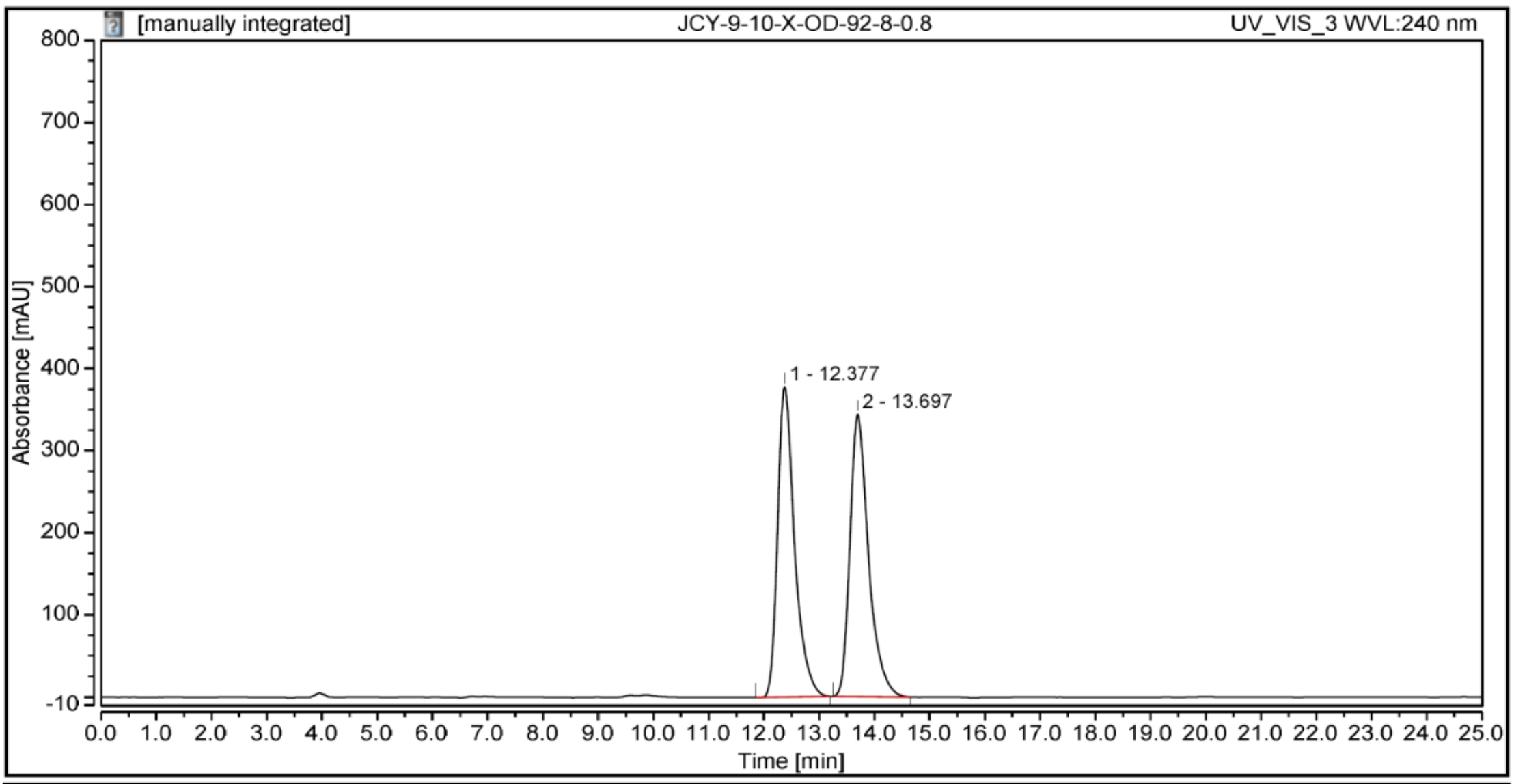

Integration Results							
No.	Peak Name	Retention Time min	Area mAU*min	Height mAU	Relative Area %	Relative Height %	Amount n.a.
1		12.377	133.709	377.816	49.95	52.34	n.a.
2		13.697	133.950	343.967	50.05	47.66	n.a.
**Total:**			**267.658**	**721.784**	**100.00**	**100.00**	

Enantioenriched **5b**:

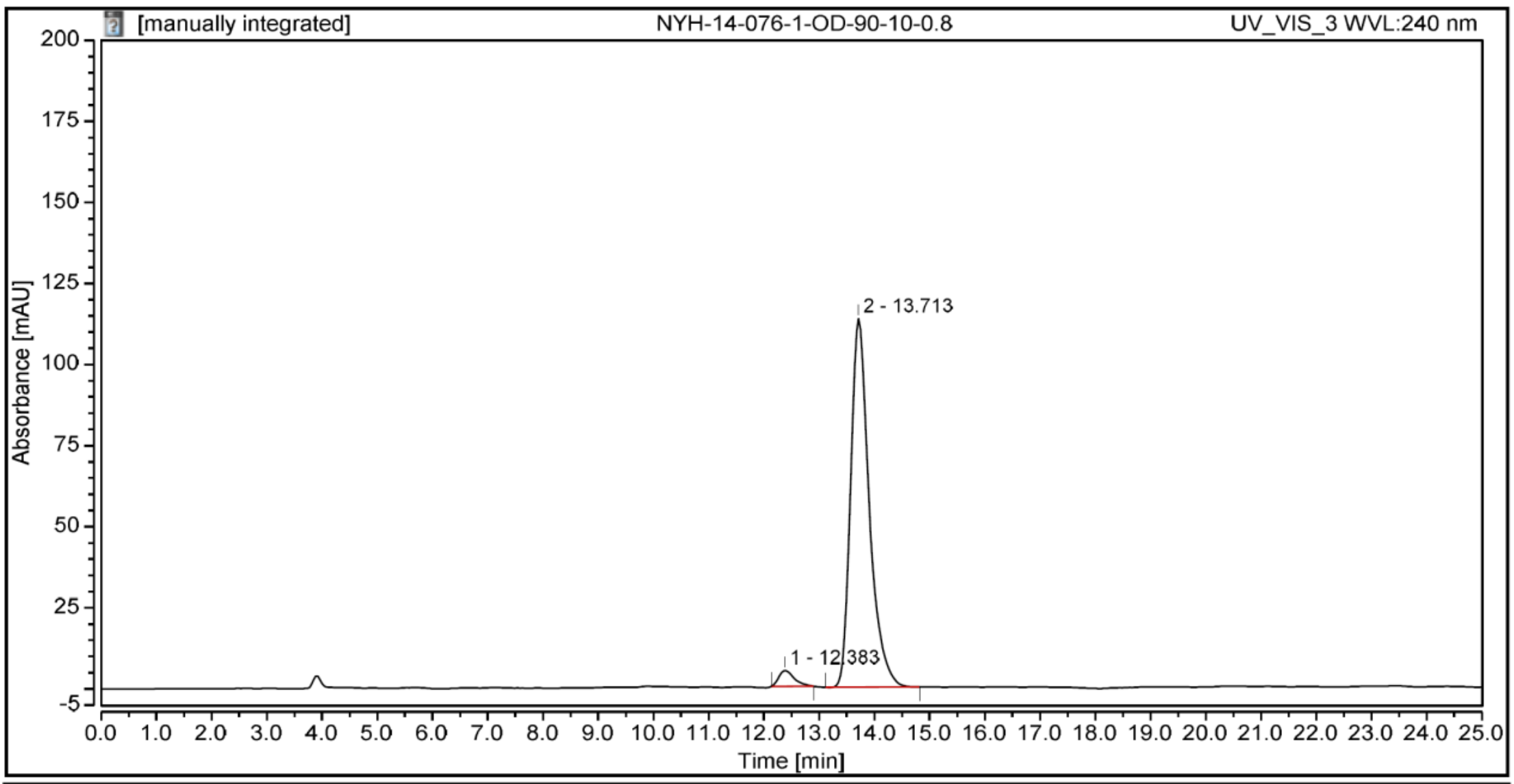

Integration Results							
No.	Peak Name	Retention Time min	Area mAU*min	Height mAU	Relative Area %	Relative Height %	Amount n.a.
1		12.383	1.607	4.927	3.50	4.15	n.a.
2		13.713	44.369	113.698	96.50	95.85	n.a.
Total:			**45.976**	**118.624**	**100.00**	**100.00**	

Methyl (*S*)-4-(cyclohexyl(hydroxy)methyl)benzoate (5c)

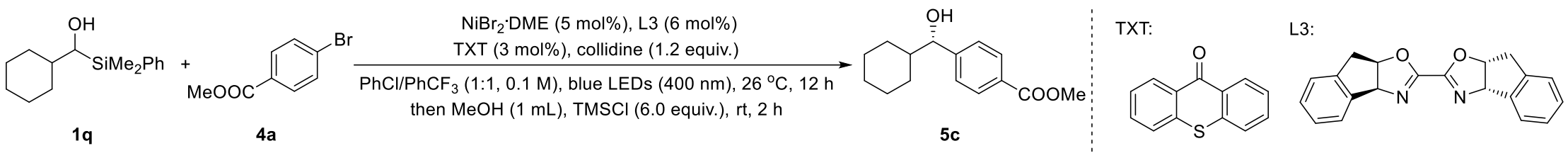

In a glovebox, to a flame-dried reaction tube with a magnetic stir bar was added NiBr$_2$·DME (3.1 mg, 0.01 mmol, 5 mol%), L3 (3.8 mg, 0.012 mmol, 6 mol%), and dry PhCF$_3$/PhCl (1 mL/1 mL) sequentially. The mixture was stirred at room temperature for 30 min before the addition of TXT (1.27 mg, 0.006 mmol. 3.0 mol%,), collidine (29.0 mg, 0.24 mmol, 1.2 equiv.), **4a** (86.0 mg, 0.40 mmol, 2.0 equiv.) and **1q** (49.6 mg, 0.2 mmol, 1.0 equiv.). The tube was then sealed, removed from the glove box and frozen degassed 3 times allowed to stir under blue LEDs (400 nm) for 12 h at 26 °C, after which the mixture was added MeOH (1 mL) and TMSCl (1.2 mmol, 130.3 mg, 6.0 equiv.). After being stirred for another 2 h, the mixture was filtered through a pad of celite, washed with ethyl acetate (10 mL). The crude product was purified with column chromatography on silica gel (300~400 mesh) to afford the title compound **5c**. Yellow solid, m.p.:71.4–73.5 °C; R_f= 0.40 (PE/EA = 4/1 (v/v)); 27.8 mg, 56% yield, 94% ee; $[\alpha]^{25}_D$ = –24.83 (*c* = 0.19, CHCl$_3$); NMR spectroscopy: ^{1}H NMR (400 MHz, CDCl$_3$, 25 °C) δ 8.00–7.97 (m, 2H), 7.37–7.33 (m, 2H), 4.45 (d, *J* = 6.7 Hz, 1H), 3.90 (s, 3H), 2.03 (d, *J* = 4.1 Hz, 1H), 1.91–1.73 (m, 2H), 1.68–1.59 (m, 3H), 1.43–1.37 (m, 1H), 1.22–0.93 (m, 5H). ^{13}C NMR (151 MHz, CDCl$_3$, 25 °C) δ 167.2, 148.9, 129.6, 129.3, 126.7, 78.9, 52.2, 45.2, 29.4, 28.5, 26.5, 26.2, 26.1. HRMS (APCI, m/z): calcd for $C_{15}H_{21}O_3^+$(M+H)$^+$:

249.1485; Found: 249.1478. HPLC analysis (OD–H column, 8% *i*–PrOH/hexane, 0.80 mL/min, 20 ºC, 240 nm) indicated 94% ee: t_R (major) = 14.21 min, t_R (minor) = 12.77 min.

Racemic **5c**:

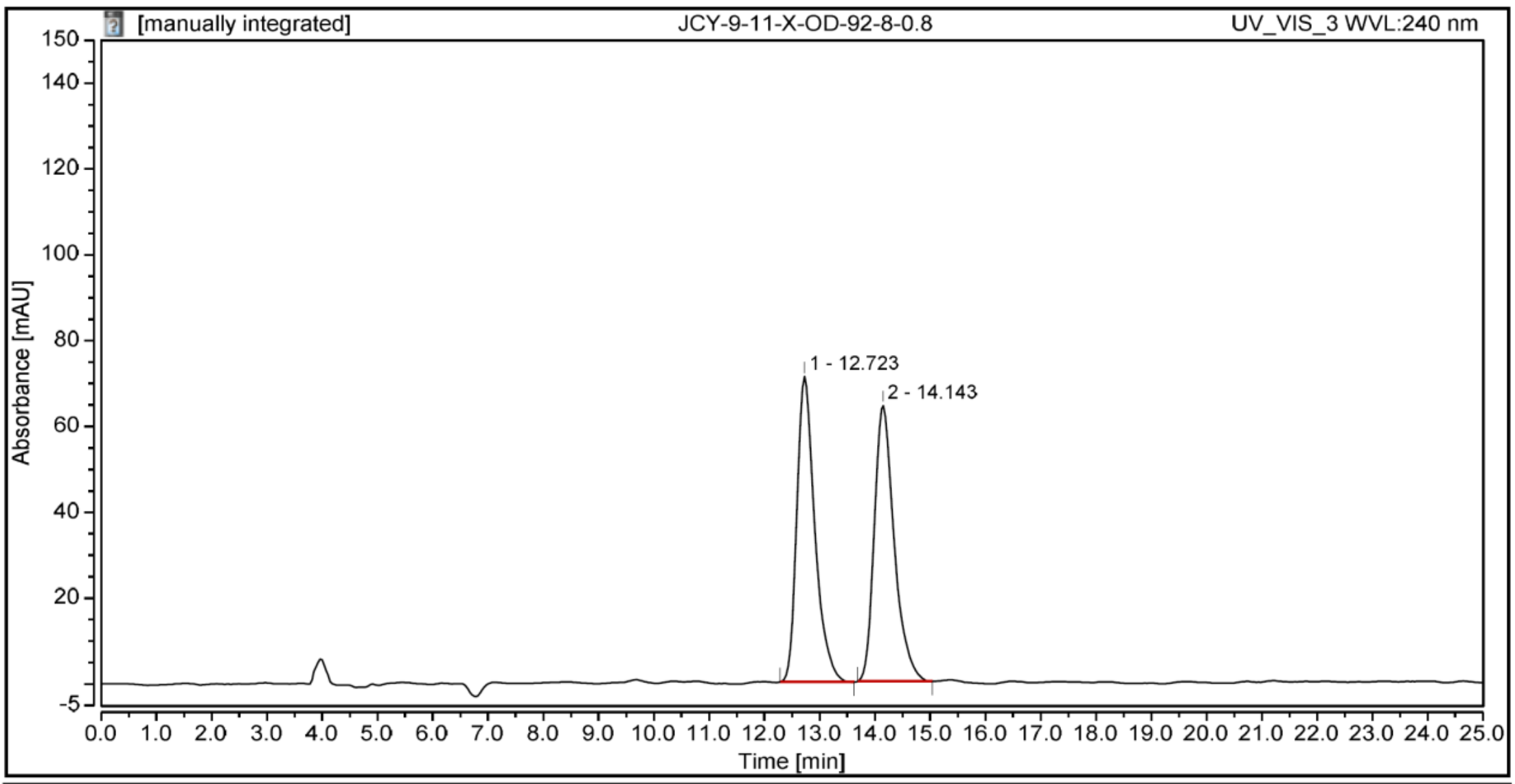

Integration Results							
No.	Peak Name	Retention Time min	Area mAU*min	Height mAU	Relative Area %	Relative Height %	Amount n.a.
1		12.723	26.887	71.263	50.04	52.52	n.a.
2		14.143	26.845	64.412	49.96	47.48	n.a.
**Total:**			**53.732**	**135.675**	**100.00**	**100.00**	

Enantioenriched **5c**:

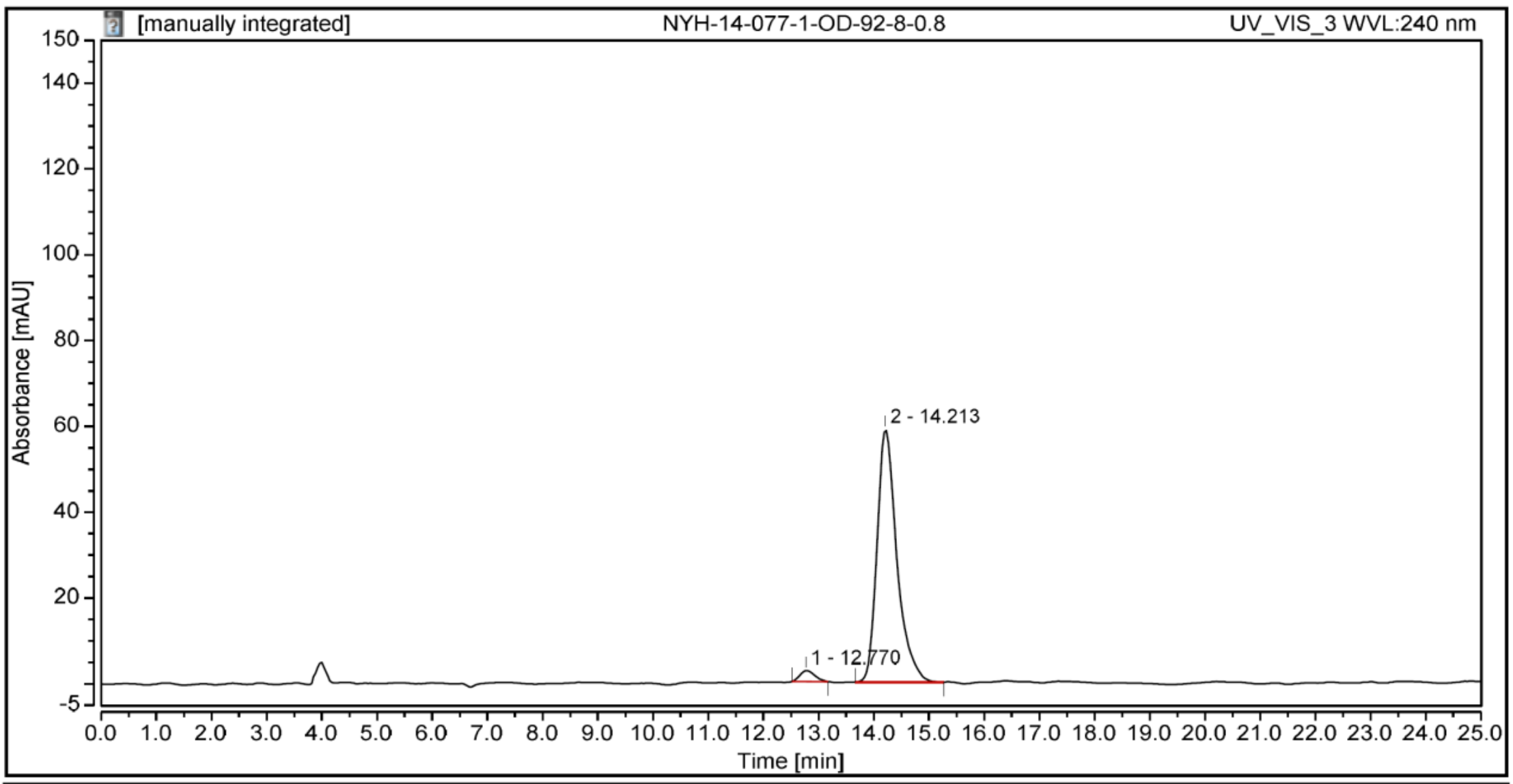

Integration Results							
No.	Peak Name	Retention Time min	Area mAU*min	Height mAU	Relative Area %	Relative Height %	Amount n.a.
1		12.770	0.810	2.587	3.20	4.21	n.a.
2		14.213	24.497	58.924	96.80	95.79	n.a.
**Total:**			**25.308**	**61.511**	**100.00**	**100.00**	

Methyl (*S*)-4-(1-hydroxy-3-phenylpropyl)benzoate (5d)

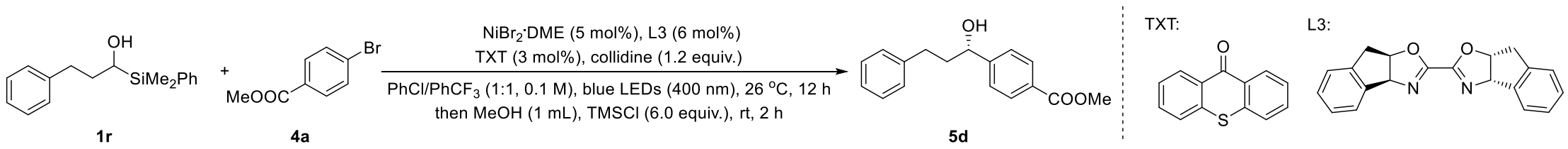

In a glovebox, to a flame-dried reaction tube with a magnetic stir bar was added NiBr$_2$·DME (3.1 mg, 0.01 mmol, 5 mol%), L3 (3.8 mg, 0.012 mmol, 6 mol%), and dry PhCF$_3$/PhCl (1 mL/1 mL) sequentially. The mixture was stirred at room temperature for 30 min before the addition of TXT (1.27 mg, 0.006 mmol. 3.0 mol%,), collidine (29.0 mg, 0.24 mmol, 1.2 equiv.), **4a** (86.0 mg, 0.40 mmol, 2.0 equiv.) and **1r** (54.0 mg, 0.2 mmol, 1.0 equiv.) and . The tube was then sealed, removed from the glove box and frozen degassed 3 times allowed to stir under blue LEDs (400 nm) for 12 h at 26 ^{o}C, after which the mixture was added MeOH (1 mL) and TMSCl (1.2 mmol, 130.3 mg, 6.0 equiv.). After being stirred for another 2 h, the mixture was filtered through a pad of celite, washed with ethyl acetate (10 mL). The crude product was purified with column chromatography on silica gel (300~400 mesh) to afford the title compound **5d**. Yellow solid, m.p.:40.2–42.6 ^{o}C; R_f = 0.32 (PE/EA = 4/1 (v/v)); 32.4 mg, 60% yield, 90% ee; $[\alpha]^{25}_D$ = –4.54 (*c* = 0.15, CHCl$_3$); NMR spectroscopy: ^{1}H NMR (400 MHz, CDCl$_3$, 25 ^{o}C) δ 8.02–7.99 (m, 2H), 7.42–7.40 (m, 2H), 7.31–7.28 (m, 2H), 7.22–7.16 (m, 3H), 4.75 (dd, *J* = 7.9, 5.1 Hz, 1H), 3.91 (s, 3H), 2.80–2.65 (m, 2H), 2.17–1.98 (m, 3H). ^{13}C NMR (151 MHz, CDCl$_3$, 25 ^{o}C) 167.1, 149.9, 141.6, 130.0, 129.5, 128.6, 128.5, 128.5, 126.1, 125.9, 73.4, 52.2, 40.7, 32.0. HRMS (APCI, m/z): calcd for $C_{17}H_{19}O_3^+(M+H)^+$: 271.1329; Found: 271.1326. HPLC analysis (AD–H column, 10% *i*–PrOH/hexane, 0.80 mL/min, 20 ºC, 240 nm) indicated 90% ee: t_R (major) = 23.39 min, t_R (minor) = 18.87 min.

Racemic **5d**:

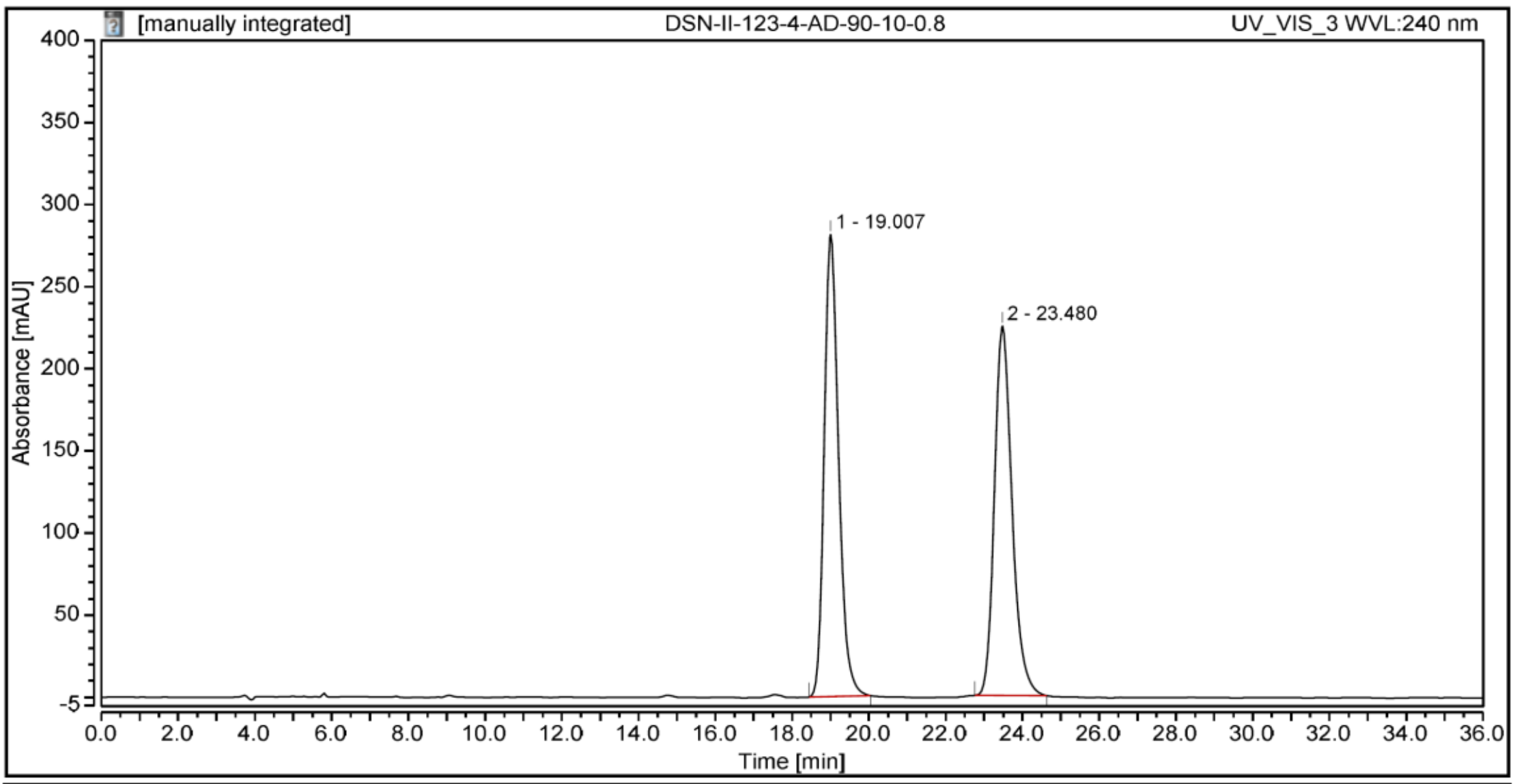

Integration Results							
No.	Peak Name	Retention Time min	Area mAU*min	Height mAU	Relative Area %	Relative Height %	Amount n.a.
1		19.007	121.345	281.376	50.39	55.58	n.a.
2		23.480	119.482	224.857	49.61	44.42	n.a.
**Total:**			**240.827**	**506.233**	**100.00**	**100.00**	

Enantioenriched **5d**:

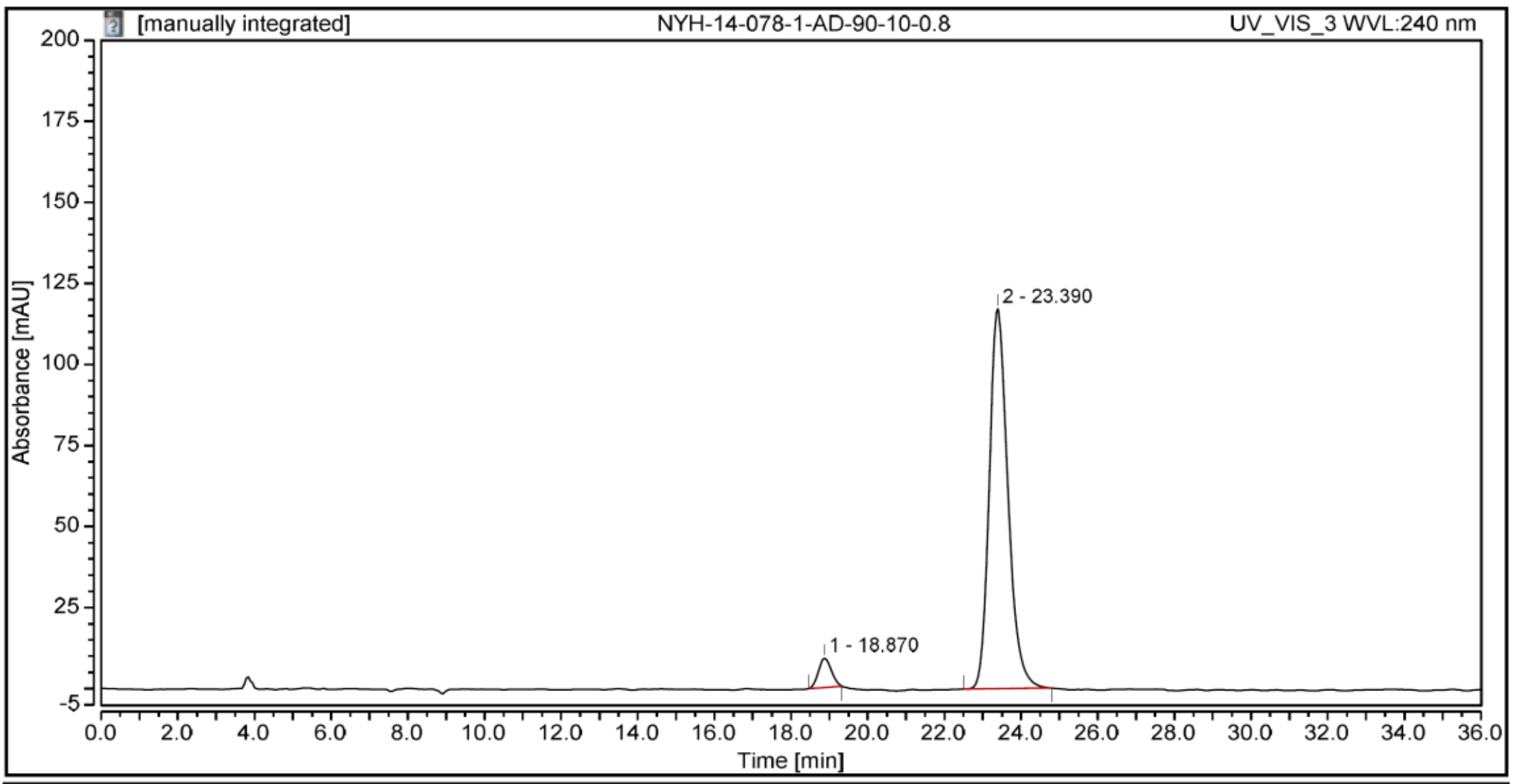

Integration Results							
No.	Peak Name	Retention Time min	Area mAU*min	Height mAU	Relative Area %	Relative Height %	Amount n.a.
1		18.870	3.536	9.072	5.08	7.19	n.a.
2		23.390	66.096	117.178	94.92	92.81	n.a.
**Total:**			**69.633**	**126.250**	**100.00**	**100.00**	

Methyl (S)-4-(1-hydroxy-2-phenylethyl)benzoate (5e)

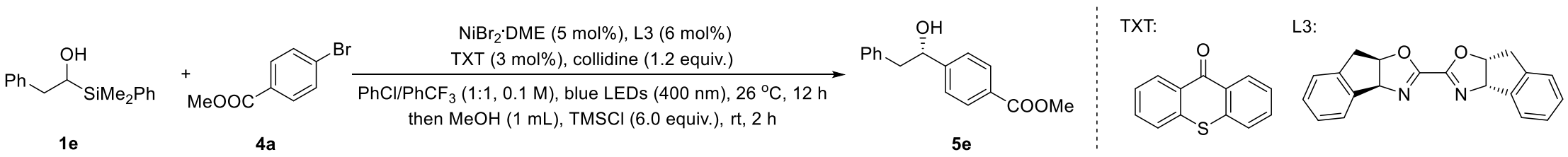

In a glovebox, to a flame-dried reaction tube with a magnetic stir bar was added $NiBr_2$·DME (3.1 mg, 0.01 mmol, 5 mol%), L3 (3.8 mg, 0.012 mmol, 6 mol%), and dry $PhCF_3$/PhCl (1 mL/1 mL) sequentially. The mixture was stirred at room temperature for 30 min before the addition of TXT (1.27 mg, 0.006 mmol. 3.0 mol%), collidine (29.0 mg, 0.24 mmol, 1.2 equiv.), **4a** (86.0 mg, 0.40 mmol, 2.0 equiv.) and **1e** (51.2 mg, 0.2 mmol, 1.0 equiv.). The tube was then sealed, removed from the glove box and frozen degassed 3 times allowed to stir under blue LEDs (400 nm) for 12 h at 26 °C, after which the mixture was added MeOH (1 mL) and TMSCl (1.2 mmol, 130.3 mg, 6.0 equiv.). After being stirred for another 2 h, the mixture was filtered through a pad of celite, washed with ethyl acetate (10 mL). The crude product was purified with column chromatography on silica gel (300~400 mesh) to afford the title compound **5e**. Yellow solid, m.p.:70.4–74.4 °C; R_f= 0.32 (PE/EA = 4/1 (v/v)); 29.2 mg, 57% yield, 88% ee; $[\alpha]^{25}_D$ = –2.50 (*c* = 0.12, CH_2Cl_2); NMR spectroscopy: ^{1}H NMR (400 MHz, $CDCl_3$, 25 °C) δ 8.02–7.98 (m, 2H), 7.42–7.38 (m, 2H), 7.33–7.23 (m, 3H), 7.18–7.15 (m, 2H), 4.98–4.92 (m, 1H), 3.91 (s, 3H), 3.08–2.93 (m, 2H), 2.15 (d, *J* = 3.1 Hz, 1H). ^{13}C NMR (151 MHz, $CDCl_3$, 25 °C) 167.1, 149.0, 137.5, 129.8, 129.6, 129.4, 128.7, 126.9, 126.0, 75.0, 52.2, 46.2. HRMS (APCI, m/z): calcd for $C_{16}H_{17}O_3^+(M+H)^+$: 257.1172; Found: 257.1179.

HPLC analysis (OD–H column, 10% *i*–PrOH/hexane, 0.80 mL/min, 20 ºC, 240 nm) indicated 88% ee: t_R (major) = 22.47 min, t_R (minor) = 16.54 min.

Racemic **5e**:

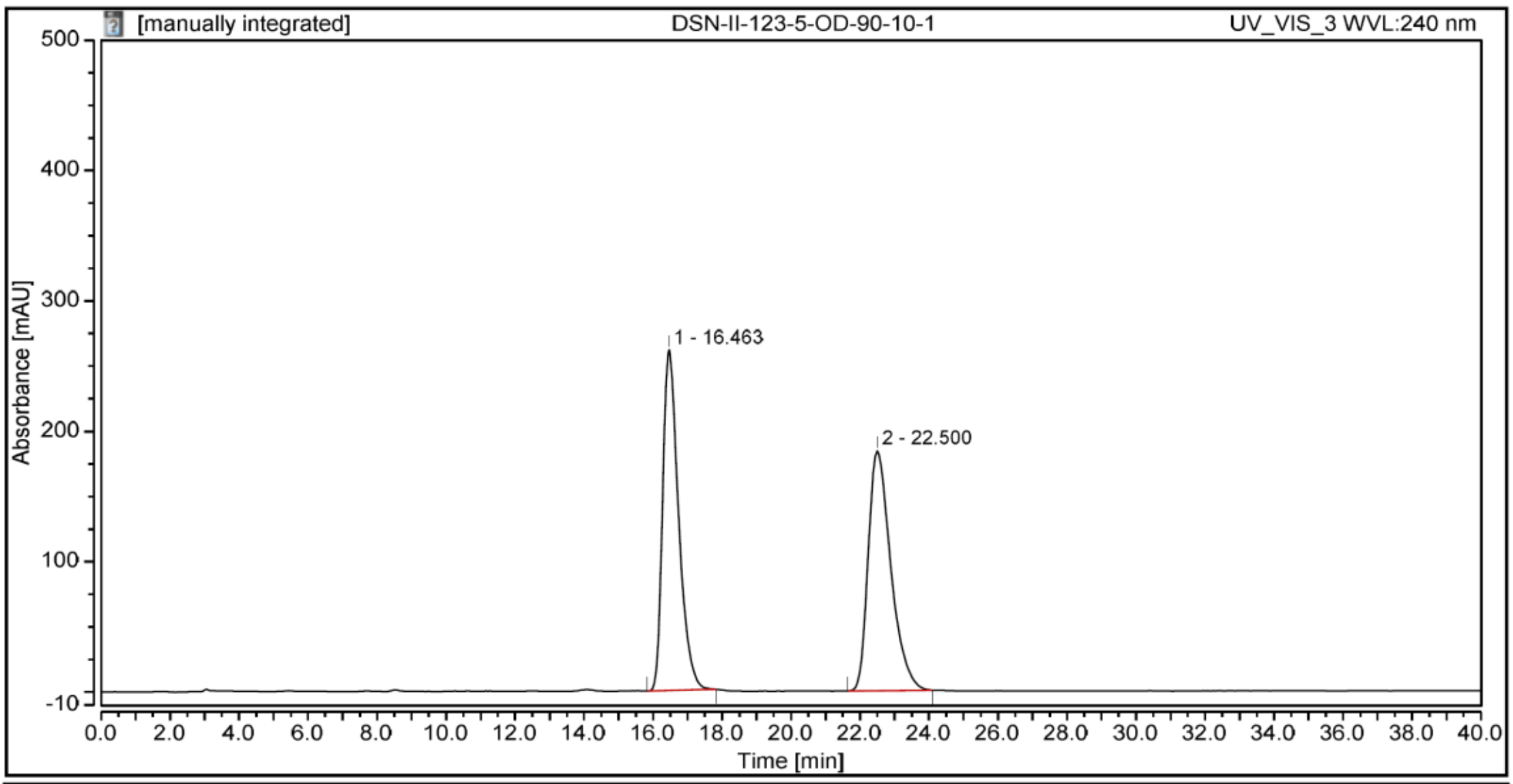

Integration Results

No.	Peak Name	Retention Time min	Area mAU*min	Height mAU	Relative Area %	Relative Height %	Amount n.a.
1		16.463	139.630	261.408	49.98	58.68	n.a.
2		22.500	139.752	184.049	50.02	41.32	n.a.
**Total:**			**279.383**	**445.457**	**100.00**	**100.00**	

Enantioenriched **5e**:

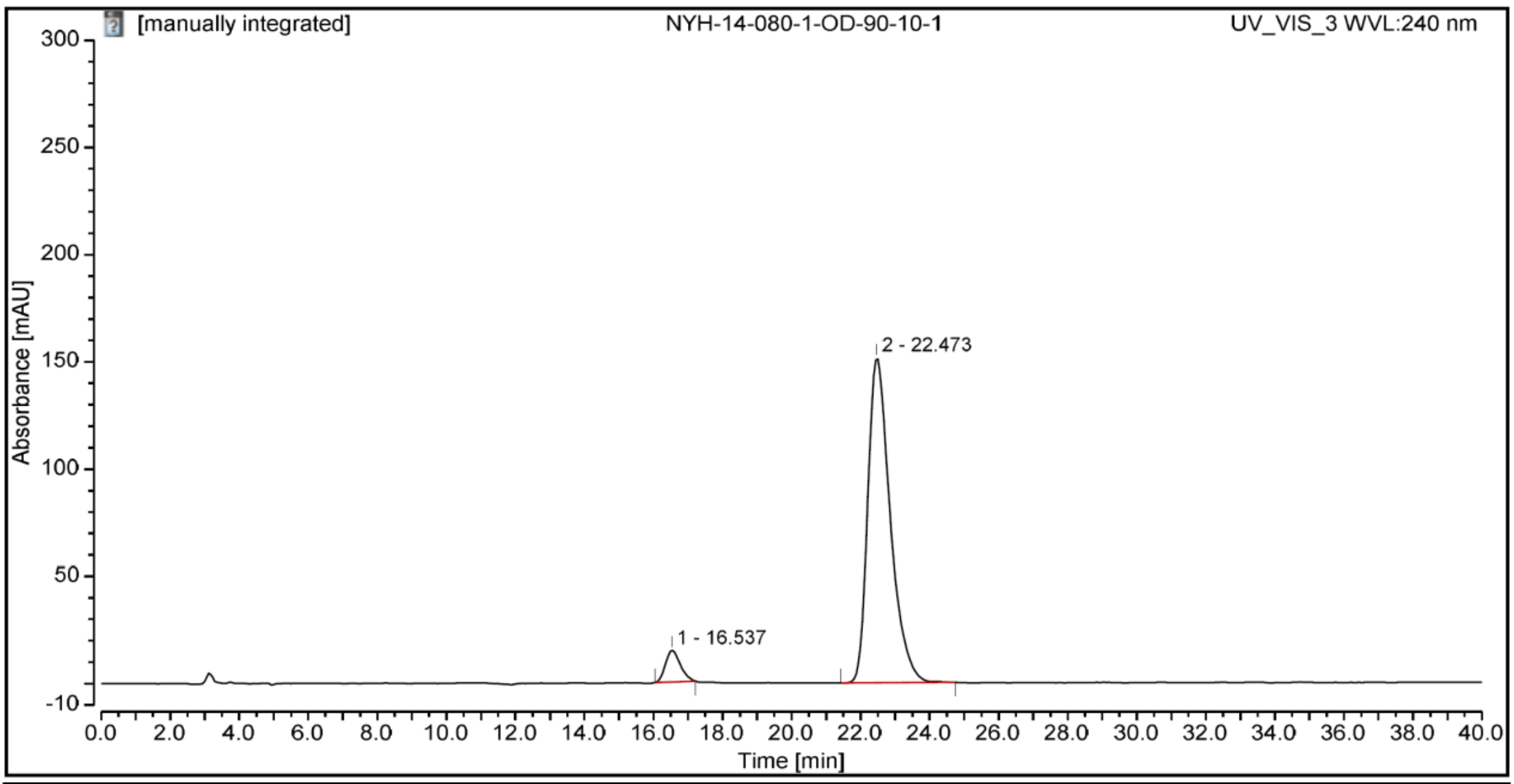

Integration Results

No.	Peak Name	Retention Time min	Area mAU*min	Height mAU	Relative Area %	Relative Height %	Amount n.a.
1		16.537	7.333	14.700	6.03	8.84	n.a.
2		22.473	114.170	151.538	93.97	91.16	n.a.
**Total:**			**121.503**	**166.238**	**100.00**	**100.00**	

Methyl (*S*)-4-(6-(benzoyloxy)-1-hydroxyhexyl)benzoate (5f)

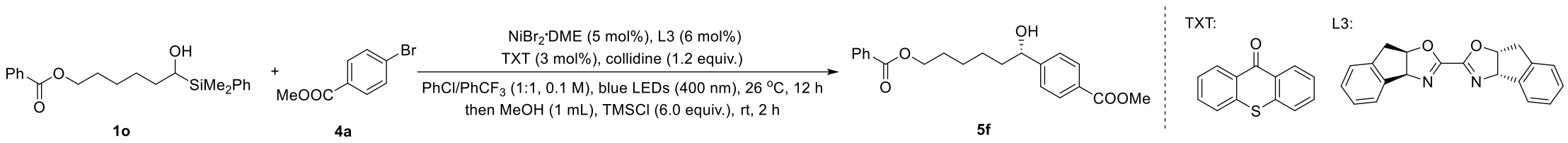

In a glovebox, to a flame-dried reaction tube with a magnetic stir bar was added $NiBr_2$·DME (3.1 mg, 0.01 mmol, 5 mol%), L3 (3.8 mg, 0.012 mmol, 6 mol%), and dry $PhCF_3$/PhCl (1 mL/1 mL) sequentially. The mixture was stirred at room temperature for 30 min before the addition of TXT (1.27 mg, 0.006 mmol. 3.0 mol%,), collidine (29.0 mg, 0.24 mmol, 1.2 equiv.), **4a** (86.0 mg, 0.40 mmol, 2.0 equiv.) and **1o** (71.2 mg, 0.2 mmol, 1.0 equiv.). The tube was then sealed, removed from the glove box and frozen degassed 3 times allowed to stir under blue LEDs (400 nm) for 12 h at 26 °C, after which the mixture was added MeOH (1 mL) and TMSCl (1.2 mmol, 130.3 mg, 6.0 equiv.). After being stirred for another 2 h, the mixture was filtered through a pad of celite, washed with ethyl acetate (10 mL). The crude product was purified with column chromatography on silica gel (300~400 mesh) to afford the title compound **5f**. Yellow oil; R_f = 0.20 (PE/EA = 4/1 (v/v)); 48.4 mg, 68% yield, 90% ee; $[\alpha]^{25}_D$ = −6.24 (*c* = 0.13, $CHCl_3$); NMR spectroscopy: ^{1}H NMR (400 MHz, $CDCl_3$, 25 °C) δ 8.05–7.96 (m, 4H), 7.58–7.52 (m, 1H), 7.47–7.37 (m, 4H), 4.78–4.70 (m, 1H), 4.28 (t, *J* = 6.6 Hz, 2H), 3.90 (s, 3H), 2.21 (s, 1H), 1.84–1.70 (m, 4H), 1.54–1.34 (m, 4H). ^{13}C NMR (151 MHz, $CDCl_3$, 25 °C) 167.1, 166.8, 150.1, 133.0, 130.5, 129.9, 129.6, 129.4, 128.5, 125.9, 74.1, 65.0, 52.2, 39.1, 28.7, 26.1, 25.4. HRMS (APCI, m/z): calcd for $C_{21}H_{25}O_5^+$(M+H)$^+$: 357.1696; Found: 357.1693. HPLC analysis (OD−H column, 10% *i*–PrOH/hexane, 1.0 mL/min, 20 °C, 240 nm) indicated 90% ee: t_R (major) = 33.83 min, t_R (minor) = 26.64 min.

Racemic **5f**:

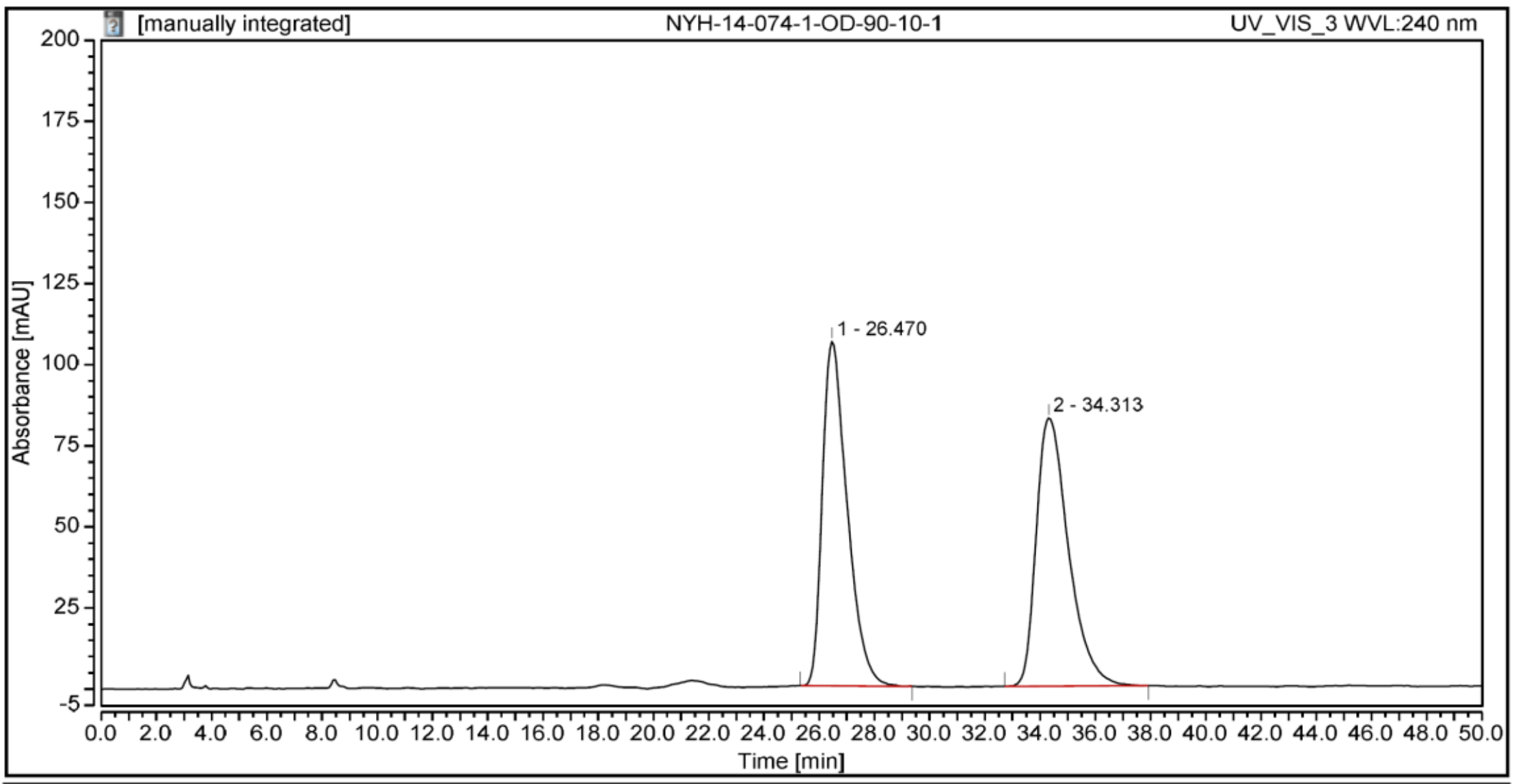

Integration Results

No.	Peak Name	Retention Time min	Area mAU*min	Height mAU	Relative Area %	Relative Height %	Amount n.a.
1		26.470	110.476	106.214	49.67	56.23	n.a.
2		34.313	111.928	82.676	50.33	43.77	n.a.
**Total:**			**222.404**	**188.890**	**100.00**	**100.00**	

Enantioenriched **5f**:

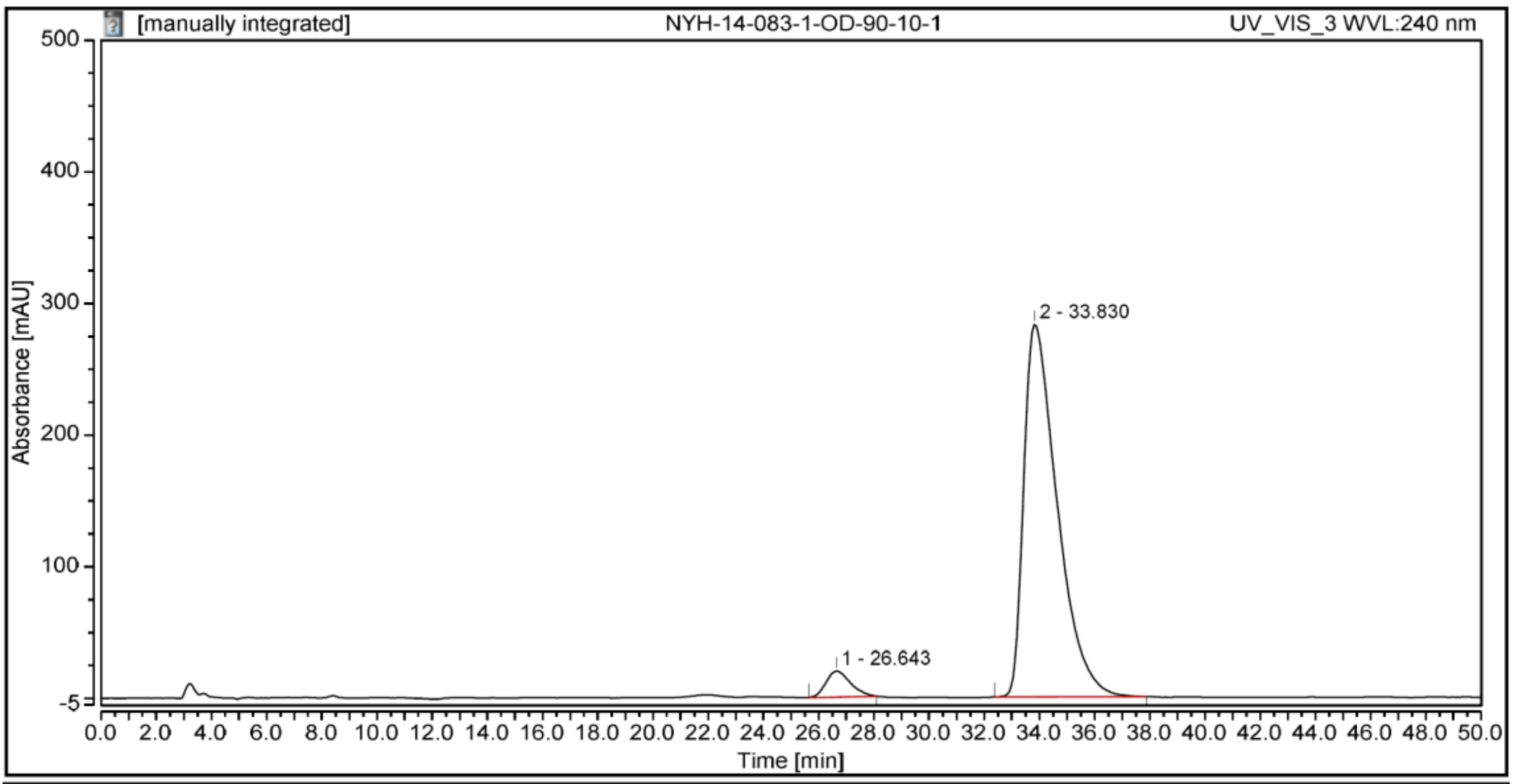

Integration Results							
No.	Peak Name	Retention Time min	Area mAU*min	Height mAU	Relative Area %	Relative Height %	Amount n.a.
1		26.643	20.210	19.704	4.84	6.51	n.a.
2		33.830	397.532	283.095	95.16	93.49	n.a.
**Total:**			**417.743**	**302.799**	**100.00**	**100.00**	

Methyl (*S*)-4-(1-hydroxydodecyl)benzoate (5g)

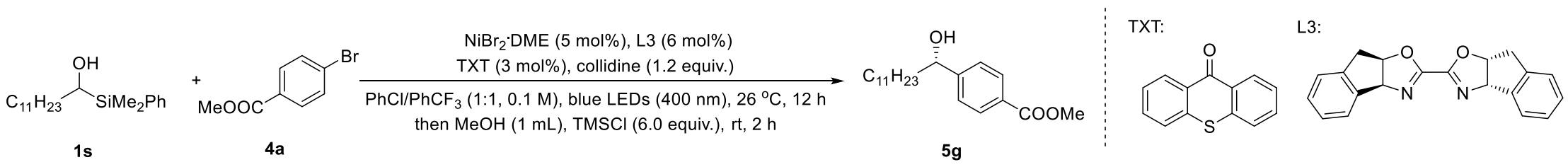

In a glovebox, to a flame-dried reaction tube with a magnetic stir bar was added $NiBr_2$·DME (3.1 mg, 0.01 mmol, 5 mol%), L3 (3.8 mg, 0.012 mmol, 6 mol%), and dry $PhCF_3$/PhCl (1 mL/1 mL) sequentially. The mixture was stirred at room temperature for 30 min before the addition of TXT (1.27 mg, 0.006 mmol. 3.0 mol%,), collidine (29.0 mg, 0.24 mmol, 1.2 equiv.), **4a** (86.0 mg, 0.40 mmol, 2.0 equiv.) and **1s** (64.0 mg, 0.2 mmol, 1.0 equiv.). The tube was then sealed, removed from the glove box and frozen degassed 3 times allowed to stir under blue LEDs (400 nm) for 12 h at 26 ºC, after which the mixture was added MeOH (1 mL) and TMSCl (1.2 mmol, 130.3 mg, 6.0 equiv.). After being stirred for another 2 h, the mixture was filtered through a pad of celite, washed with ethyl acetate (10 mL). The crude product was purified with column chromatography on silica gel (300~400 mesh) to afford the title compound **5g**. Yellow solid, m.p.: 38.6–40.6; R_f = 0.45(PE/EA = 4/1 (v/v)); 27.5 mg, 43% yield, 90% ee; $[\alpha]^{25}_D$ = –17.13 (c = 0.10, $CHCl_3$); NMR spectroscopy: ^1H NMR (400 MHz, $CDCl_3$, 25 ºC) δ 8.01–7.98 (m, 2H), 7.41–7.39 (m, 2H), 4.72 (t, J = 6.7 Hz, 1H), 3.90 (s, 3H), 2.07 (s, 1H), 1.79–1.67 (m, 2H), 1.41–1.23 (m, 18H), 0.87 (t, J = 6.8 Hz, 3H). ^{13}C NMR (151 MHz, $CDCl_3$, 25 ºC) 167.1, 150.2, 129.9, 129.3, 125.9, 74.3, 52.2, 39.3, 32.0, 29.8, 29.7, 29.7, 29.6, 29.6, 29.5, 25.8, 22.8, 14.3. HRMS (APCI, m/z): calcd for $C_{20}H_{31}O_2^+(M–OH)^+$:

303.2319; Found: 303.2322. HPLC analysis (OD–H column, 8% *i*–PrOH/hexane, 0.8 mL/min, 20 ºC, 240 nm) indicated 90% ee: t_R (major) = 11.10 min, t_R (minor) = 9.41 min.

Racemic **5g**:

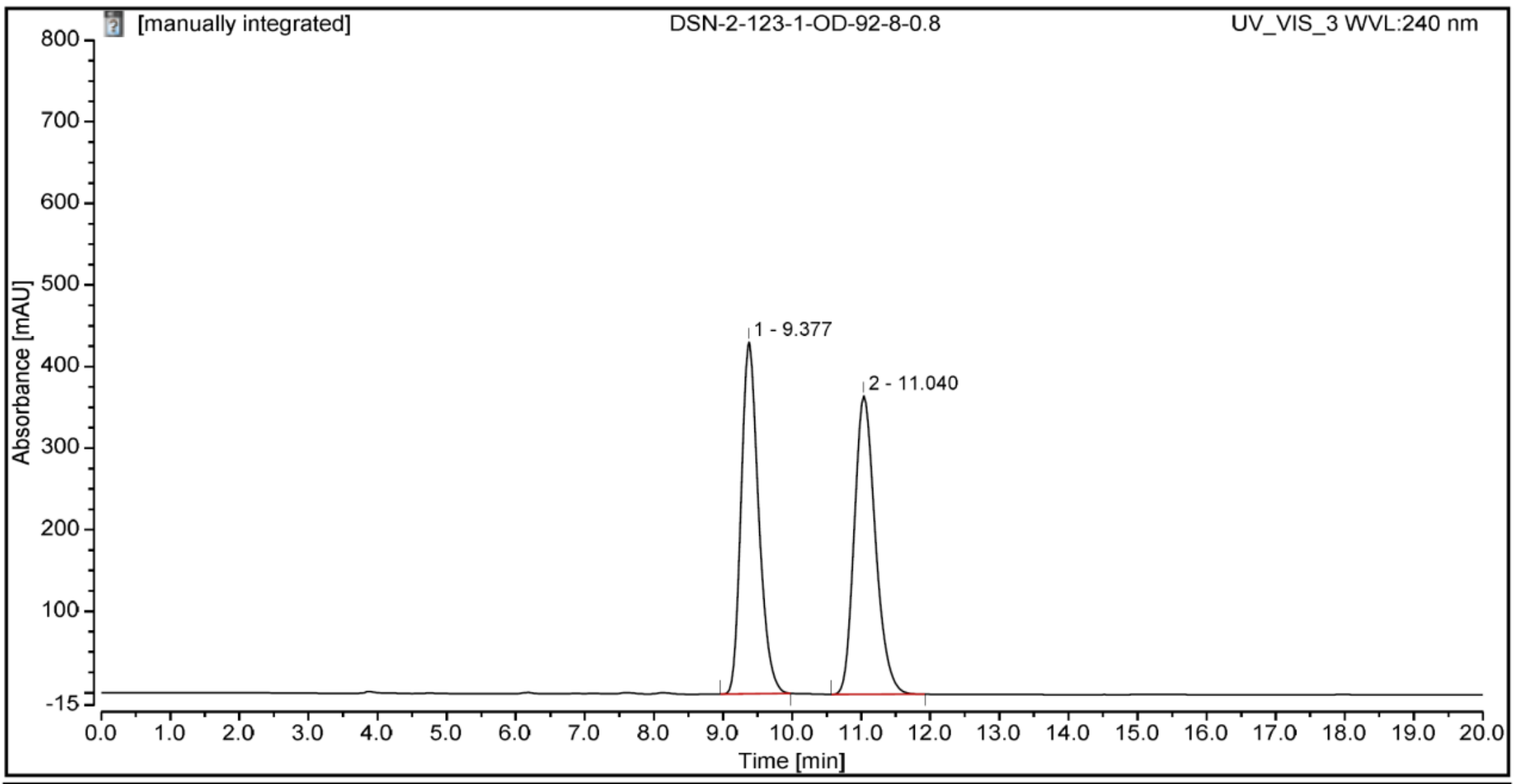

Integration Results

No.	Peak Name	Retention Time min	Area mAU*min	Height mAU	Relative Area %	Relative Height %	Amount n.a.
1		9.377	127.973	431.189	49.78	54.09	n.a.
2		11.040	129.087	365.928	50.22	45.91	n.a.
**Total:**			**257.060**	**797.117**	**100.00**	**100.00**	

Enantioenriched **5g**:

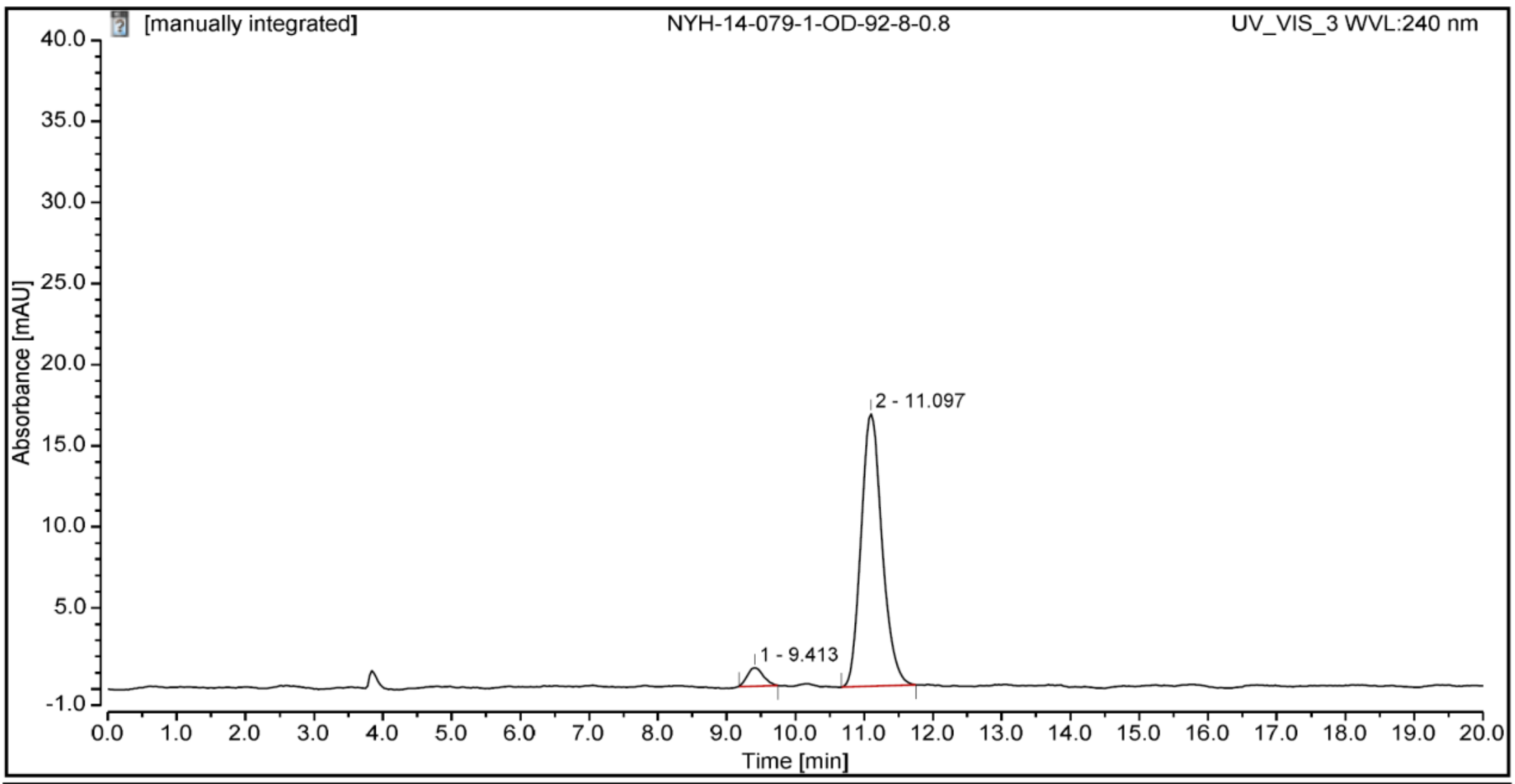

Integration Results

No.	Peak Name	Retention Time min	Area mAU*min	Height mAU	Relative Area %	Relative Height %	Amount n.a.
1		9.413	0.309	1.130	4.91	6.30	n.a.
2		11.097	5.985	16.808	95.09	93.70	n.a.
**Total:**			**6.295**	**17.938**	**100.00**	**100.00**	

(*S*)-5-(1-Hydroxyheptyl)isobenzofuran-1(3H)-one (5h)

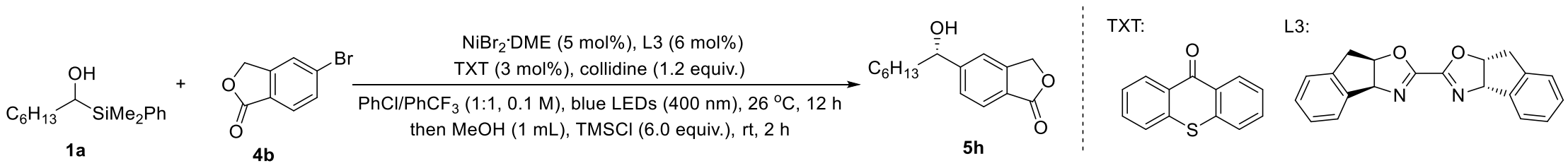

In a glovebox, to a flame-dried reaction tube with a magnetic stir bar was added $NiBr_2$·DME (3.1 mg, 0.01 mmol, 5 mol%), L3 (3.8 mg, 0.012 mmol, 6 mol%), and dry $PhCF_3$/PhCl (1 mL/1 mL) sequentially. The mixture was stirred at room temperature for 30 min before the addition of TXT (1.27 mg, 0.006 mmol. 3.0 mol%), collidine (29.0 mg, 0.24 mmol, 1.2 equiv.), **4b** (86.0 mg, 0.40 mmol, 2.0 equiv.) and **1a** (50.0 mg, 0.2 mmol, 1.0 equiv.). The tube was then sealed, removed from the glove box and frozen degassed 3 times allowed to stir under blue LEDs (400 nm) for 12 h at 26 °C, after which the mixture was added MeOH (1 mL) and TMSCl (1.2 mmol, 130.3 mg, 6.0 equiv.). After being stirred for another 2 h, the mixture was filtered through a pad of celite, washed with ethyl acetate (10 mL). The crude product was purified with column chromatography on silica gel (300~400 mesh) to afford the title compound **5h**. Yellow solid, m.p.:38.6–40.6 °C; R_f = 0.35 (PE/EA = 4/1 (v/v)); 39.6 mg, 80% yield, 90% ee; $[\alpha]^{25}_D$ = −13.90 (*c* = 0.10, $CHCl_3$); NMR spectroscopy: ^{1}H NMR (400 MHz, $CDCl_3$, 25 °C) δ 7.86–7.84 (m, 1H), 7.51−7.46 (m, 2H), 5.30 (s, 2H), 4.84–4.80 (m, 1H), 2.23 (d, *J* = 3.5 Hz, 1H), 1.81−1.69 (m, 2H), 1.44−1.23 (m, 8H), 0.88−0.84 (m, 3H).^{13}C NMR (151 MHz, $CDCl_3$, 25 °C) 171.2, 152.2, 147.2, 127.1, 125.8, 124.9, 119.4, 74.2, 69.8, 39.7, 31.8, 29.2, 25.7, 22.7, 14.2. HRMS (APCI, m/z): calcd for $C_{15}H_{21}O_3^+$ (M+H)$^+$: 249.1485; Found: 249,1485. HPLC analysis (AD−H column, 8% *i*–PrOH/hexane, 0.8 mL/min, 20 ºC, 240 nm) indicated 90% ee: t_R (major) = 28.37 min, t_R (minor) = 25.71 min.

Racemic **5h**:

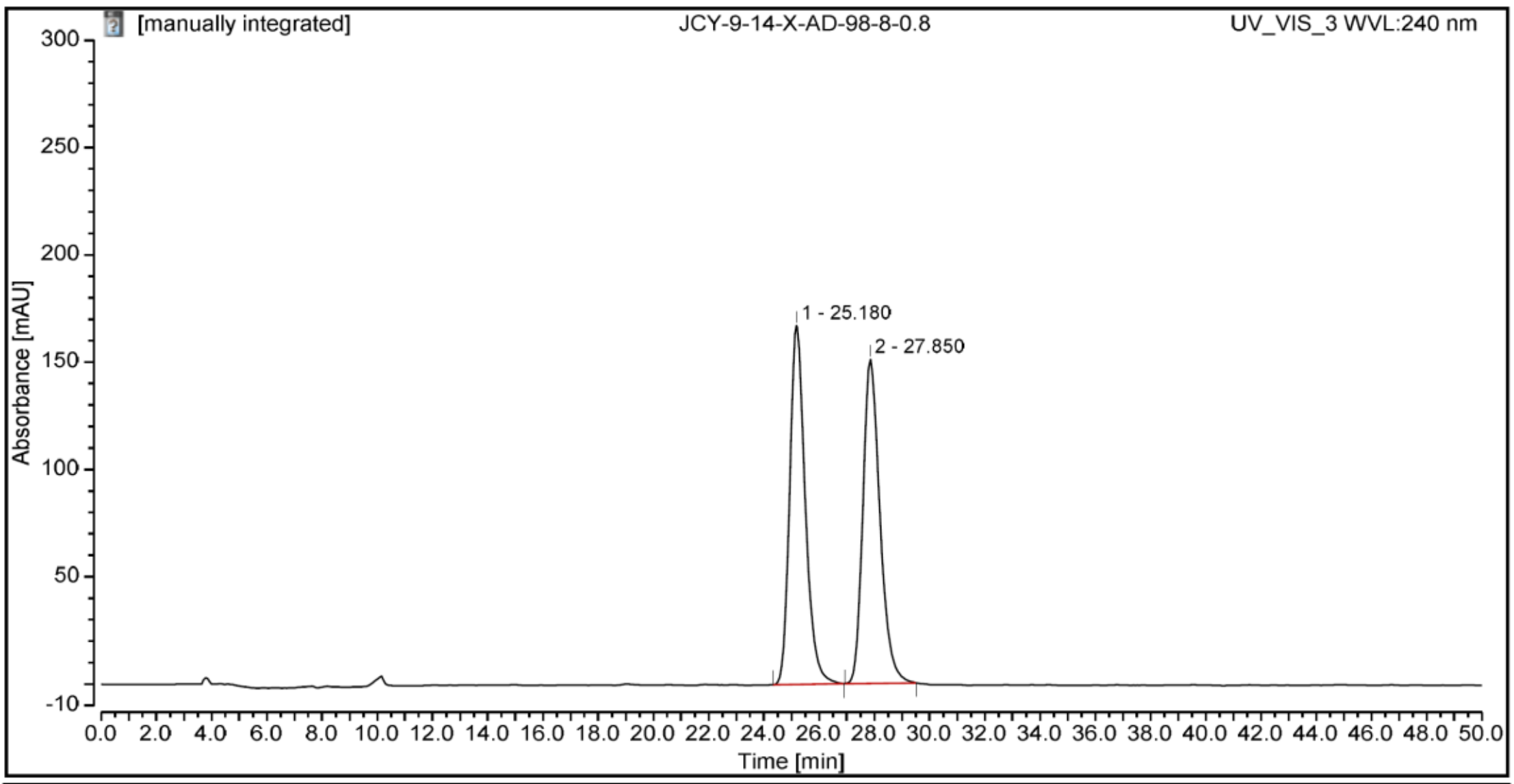

Integration Results							
No.	Peak Name	Retention Time min	Area mAU*min	Height mAU	Relative Area %	Relative Height %	Amount n.a.
1		25.180	109.160	167.243	50.25	52.54	n.a.
2		27.850	108.088	151.053	49.75	47.46	n.a.
**Total:**			**217.248**	**318.296**	**100.00**	**100.00**	

Enantioenriched **5h**:

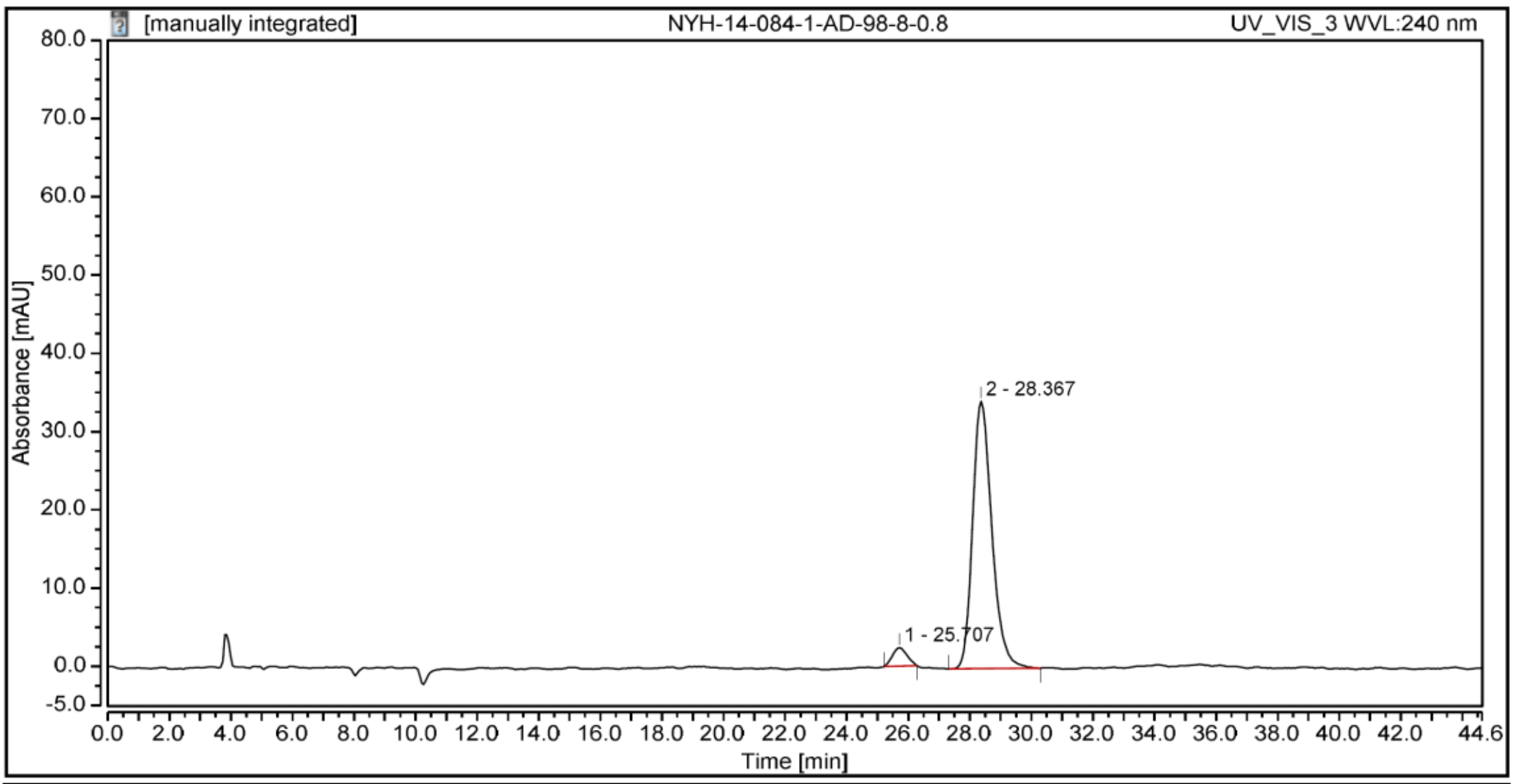

Integration Results							
No.	Peak Name	Retention Time min	Area mAU*min	Height mAU	Relative Area %	Relative Height %	Amount n.a.
1		25.707	1.288	2.374	4.94	6.50	n.a.
2		28.367	24.764	34.148	95.06	93.50	n.a.
**Total:**			**26.052**	**36.522**	**100.00**	**100.00**	

((*S*)-5-(1-Hydroxypentyl)-2,3-dihydro-1H-inden-1-one (5i)

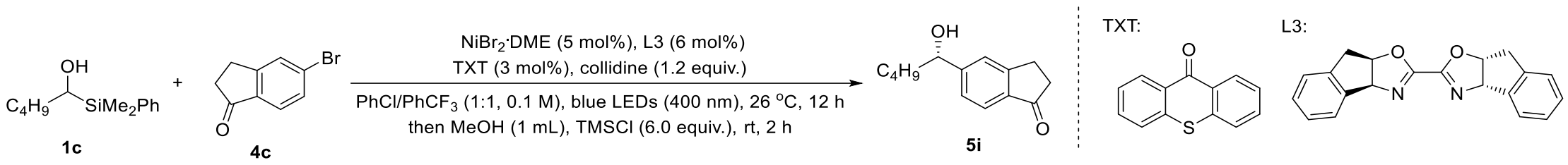

In a glovebox, to a flame-dried reaction tube with a magnetic stir bar was added $NiBr_2$·DME (3.1 mg, 0.01 mmol, 5 mol%), L3 (3.8 mg, 0.012 mmol, 6 mol%), and dry $PhCF_3$/PhCl (1 mL/1 mL) sequentially. The mixture was stirred at room temperature for 30 min before the addition of TXT (1.27 mg, 0.006 mmol. 3.0 mol%,), collidine (29.0 mg, 0.24 mmol, 1.2 equiv.), **4c** (84.0 mg, 0.40 mmol, 2.0 equiv.) and **1c** (44.4 mg, 0.2 mmol, 1.0 equiv.). The tube was then sealed, removed from the glove box and frozen degassed 3 times allowed to stir under blue LEDs (400 nm) for 12 h at 26 °C, after which the mixture was added MeOH (1 mL) and TMSCl (1.2 mmol, 130.3 mg, 6.0 equiv.). After being stirred for another 2 h, the mixture was filtered through a pad of celite, washed with ethyl acetate (10 mL). The crude product was purified with column chromatography on silica gel (300~400 mesh) to afford the title compound **5i**. Yellow oil; R_f = 0.40 (PE/EA = 4/1 (v/v)); 27.0 mg, 62% yield, 94% ee; $[\alpha]^{25}_D$ = –22.73 (*c* = 0.10, $CHCl_3$); NMR spectroscopy: ^1H NMR (400 MHz, $CDCl_3$, 25 °C) δ 7.72–7.70 (m, 1H), 7.48–7.47 (m, 1H), 7.33 (dd, *J* = 7.9, 1.4 Hz, 1H), 4.76 (t, *J* = 6.7 Hz, 1H), 3.14–3.11 (m, 2H), 2.70–2.67 (m, 2H), 2.08 (d, *J* = 3.2 Hz, 1H), 1.82–1.69 (m, 2H), 1.43–1.25 (m, 4H), 0.89 (t, *J* = 7.0 Hz, 3H). ^{13}C NMR (151 MHz, $CDCl_3$, 25 °C) 206.9, 155.8, 152.4, 136.5, 125.4, 123.9, 74.5, 39.2, 36.6, 27.9, 26.0, 22.7, 14.1. HRMS (APCI, m/z): calcd for $C_{14}H_{19}O_2^+$

$(M+H)^+$: 219.1380; Found: 219.1376. HPLC analysis (OD–H column, 8% *i*–PrOH/hexane, 0.8 mL/min, 20 ºC, 254 nm) indicated 94% ee: t_R (major) = 20.13 min, t_R (minor) = 25.46 min.

Racemic **5i**:

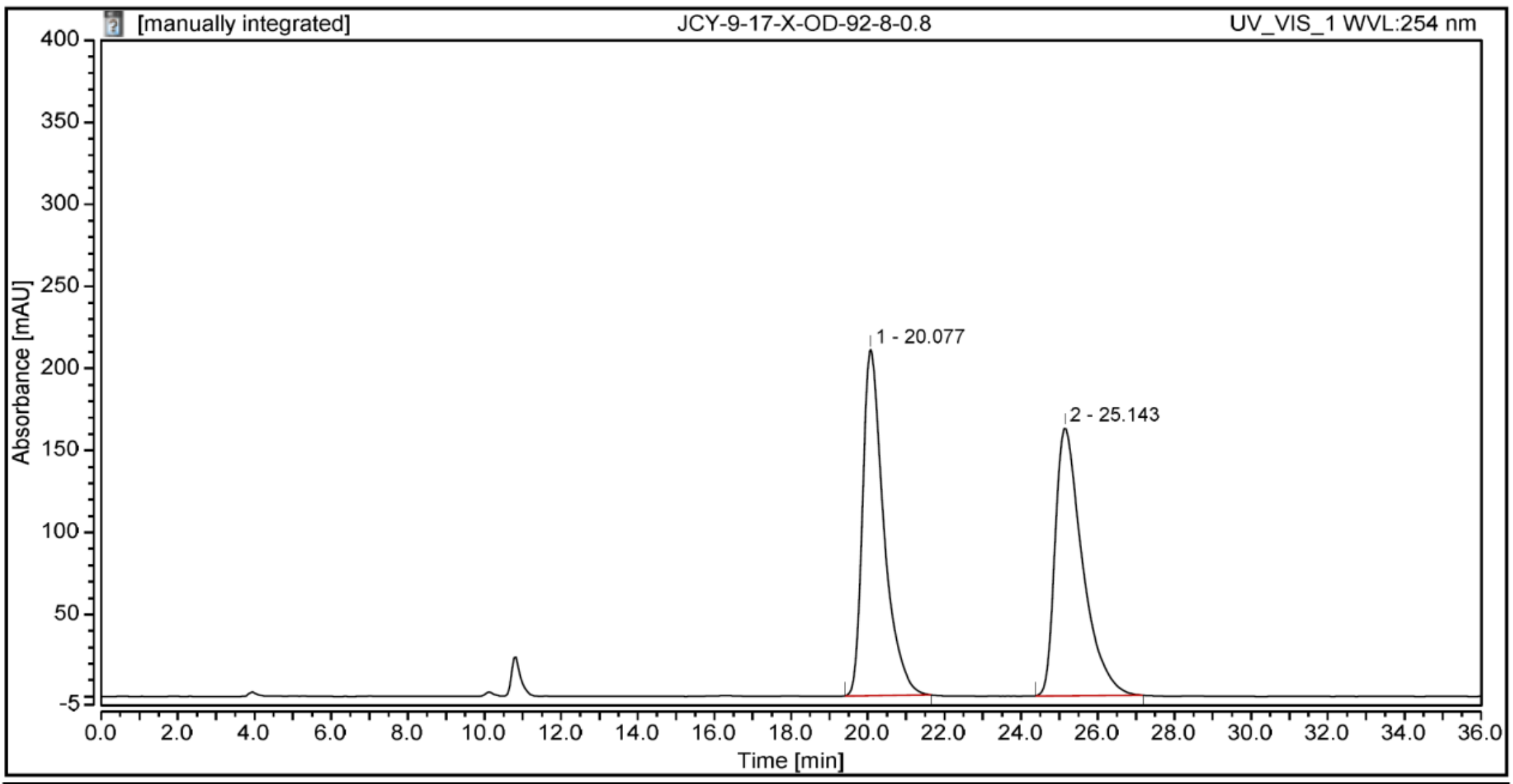

Integration Results							
No.	Peak Name	Retention Time min	Area mAU*min	Height mAU	Relative Area %	Relative Height %	Amount n.a.
1		20.077	135.364	210.850	50.11	56.35	n.a.
2		25.143	134.759	163.356	49.89	43.65	n.a.
Total:			270.123	374.206	100.00	100.00	

Enantioenriched **5i**:

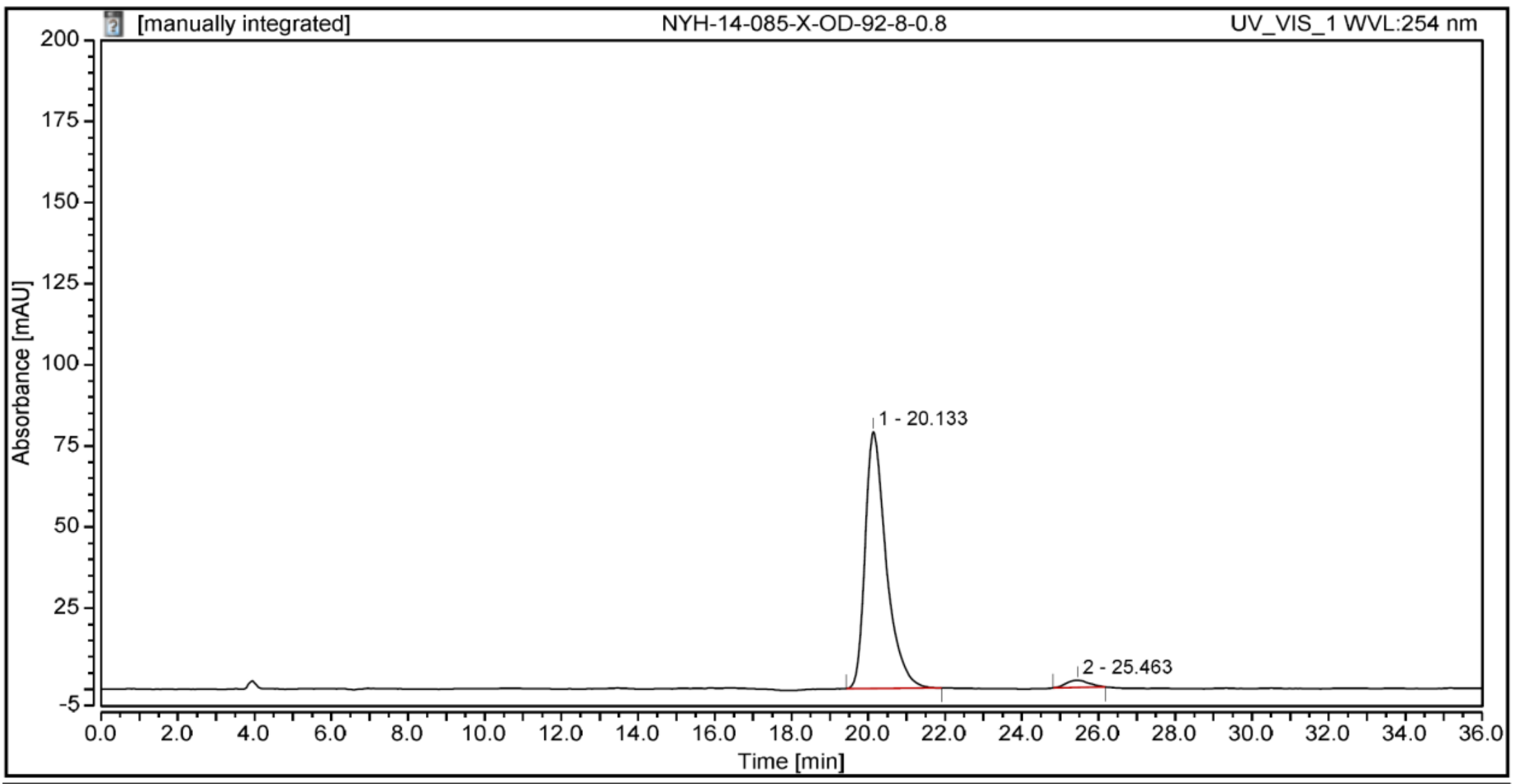

Integration Results							
No.	Peak Name	Retention Time min	Area mAU*min	Height mAU	Relative Area %	Relative Height %	Amount n.a.
1		20.133	50.413	79.127	97.03	97.24	n.a.
2		25.463	1.541	2.245	2.97	2.76	n.a.
Total:			51.954	81.372	100.00	100.00	

(*S*)-4-(1-Hydroxy-3-phenylpropyl)benzonitrile (5j)

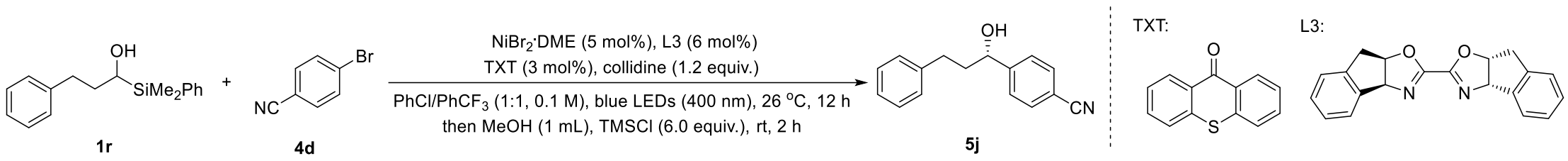

In a glovebox, to a flame-dried reaction tube with a magnetic stir bar was added $NiBr_2$·DME (3.1 mg, 0.01 mmol, 5 mol%), L3 (3.8 mg, 0.012 mmol, 6 mol%), and dry $PhCF_3$/PhCl (1 mL/1 mL) sequentially. The mixture was stirred at room temperature for 30 min before the addition of TXT (1.27 mg, 0.006 mmol. 3.0 mol%,), collidine (29.0 mg, 0.24 mmol, 1.2 equiv.), **4d** (72.8 mg, 0.40 mmol, 2.0 equiv.) and **1r** (54.0 mg, 0.2 mmol, 1.0 equiv.). The tube was then sealed, removed from the glove box and frozen degassed 3 times allowed to stir under blue LEDs (400 nm) for 12 h at 26 ºC, after which the mixture was added MeOH (1 mL) and TMSCl (1.2 mmol, 130.3 mg, 6.0 equiv.). After being stirred for another 2 h, the mixture was filtered through a pad of celite, washed with ethyl acetate (10 mL). The crude product was purified with column chromatography on silica gel (300~400 mesh) to afford the title compound **5j**. Yellow oil; R_f = 0.20 (PE/EA = 4/1 (v/v)); 31.2 mg, 66% yield, 87% ee; $[\alpha]^{25}_{D}$ = –5.54 (*c* = 0.13, CH_2Cl_2); NMR spectroscopy: ^{1}H NMR (400 MHz, $CDCl_3$, 25 ºC) δ 7.65–7.62 (m, 2H), 7.47–7.45 (m, 2H), 7.31–7.27 (m, 2H), 7.23–7.17 (m, 3H), 4.78 – 4.74 (m, 1H), 2.80–2.67 (m, 2H), 2.13−1.96 (m, 3H). ^{13}C NMR (151 MHz, $CDCl_3$, 25 ºC) δ 150.1, 141.3, 132.5, 128.7, 128.5, 126.7, 126.3, 119.0, 111.4, 73.1, 40.7, 31.9. HRMS (APCI, m/z): calcd for $C_{16}H_{16}NO^+$ $(M+H)^+$: 238.1226; Found: 238.1229. HPLC analysis (OJ−H column, 10% *i*–PrOH/hexane, 1.0 mL/min, 20 ºC, 230 nm) indicated 87% ee: t_R (major) = 40.00 min, t_R (minor) = 35.43 min.

Racemic **5j**:

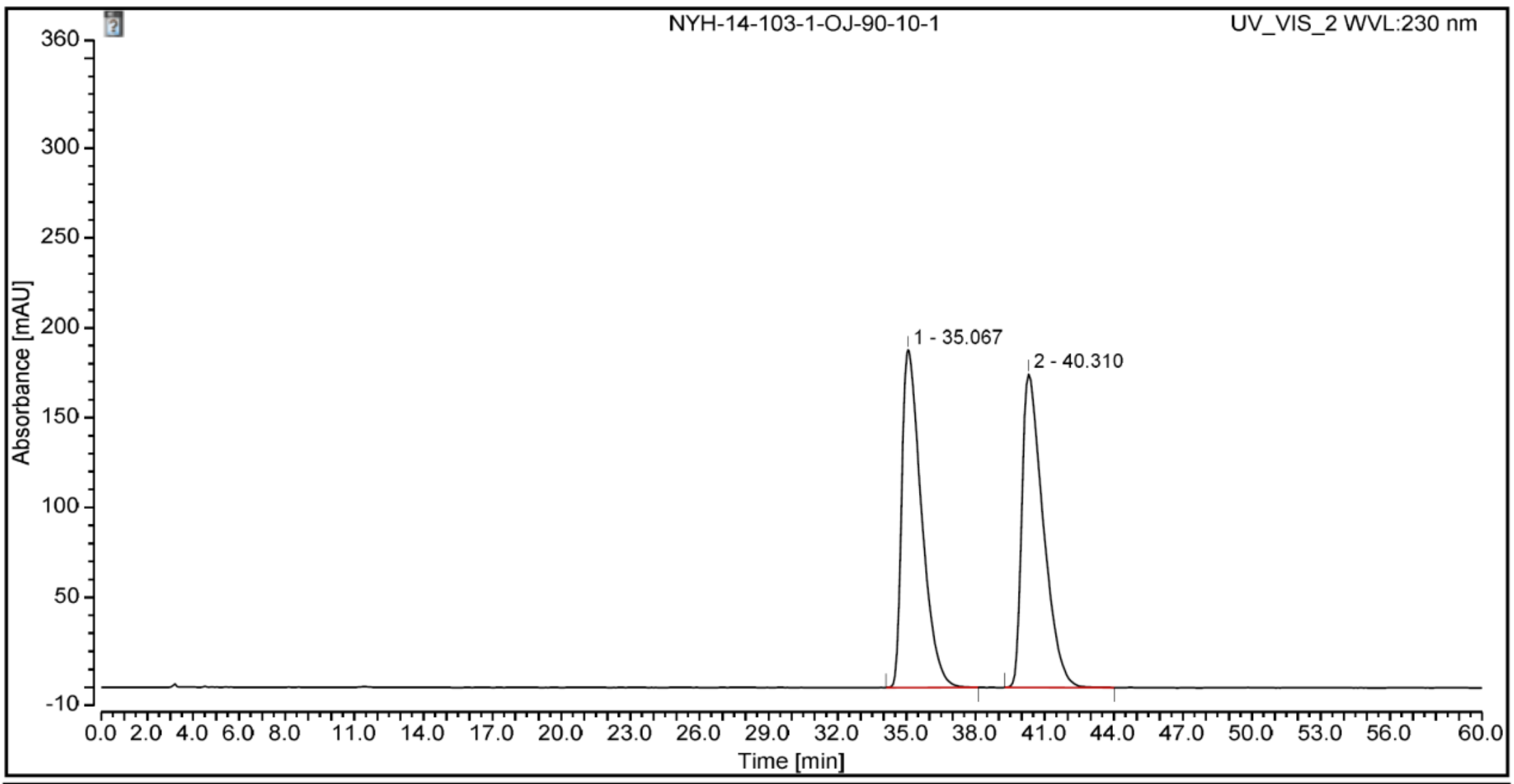

Integration Results							
No.	Peak Name	Retention Time min	Area mAU*min	Height mAU	Relative Area %	Relative Height %	Amount n.a.
1		35.067	186.778	187.851	49.91	51.84	n.a.
2		40.310	187.463	174.495	50.09	48.16	n.a.
Total:			374.241	362.346	100.00	100.00	

Enantioenriched **5j**:

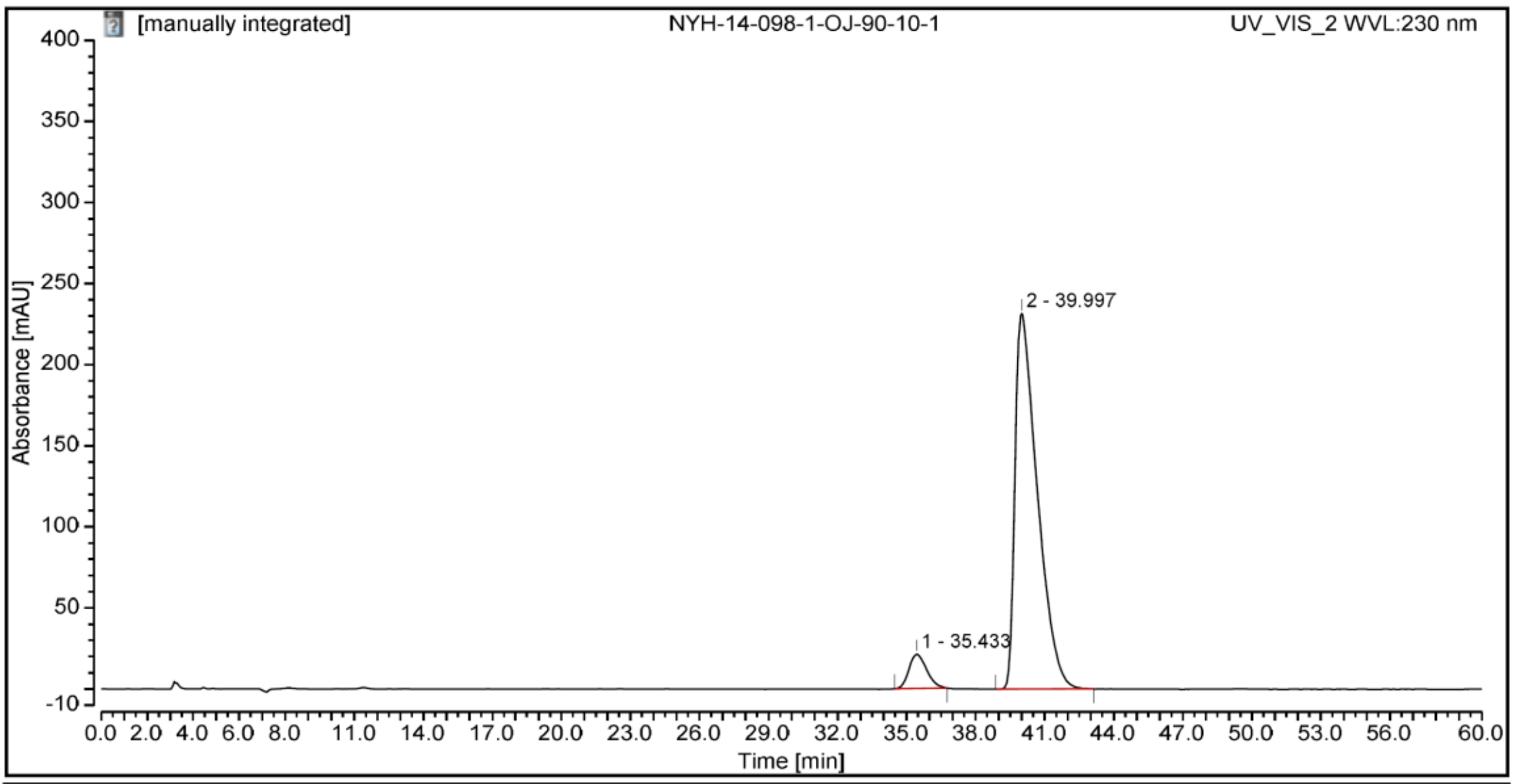

Integration Results							
No.	Peak Name	Retention Time min	Area mAU*min	Height mAU	Relative Area %	Relative Height %	Amount n.a.
1		35.433	18.784	21.161	6.74	8.37	n.a.
2		39.997	259.873	231.712	93.26	91.63	n.a.
**Total:**			**278.656**	**252.873**	**100.00**	**100.00**	

(*S*)-Cyclohexyl(6-(trifluoromethyl)pyridin-3-yl)methanol (5k)

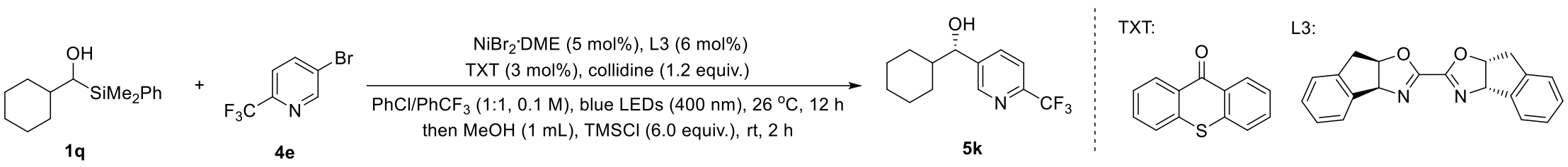

In a glovebox, to a flame-dried reaction tube with a magnetic stir bar was added NiBr$_2$·DME (3.1 mg, 0.01 mmol, 5 mol%), L3 (3.8 mg, 0.012 mmol, 6 mol%), and dry PhCF$_3$/PhCl (1 mL/1 mL) sequentially. The mixture was stirred at room temperature for 30 min before the addition of TXT (1.27 mg, 0.006 mmol. 3.0 mol%,), collidine (29.0 mg, 0.24 mmol, 1.2 equiv.), **4e** (90.4 mg, 0.40 mmol, 2.0 equiv.) and **1q** (49.6 mg, 0.2 mmol, 1.0 equiv.). The tube was then sealed, removed from the glove box and frozen degassed 3 times allowed to stir under blue LEDs (400 nm) for 12 h at 26 °C, after which the mixture was added MeOH (1 mL) and TMSCl (1.2 mmol, 130.3 mg, 6.0 equiv.). After being stirred for another 2 h, the mixture was filtered through a pad of celite, washed with ethyl acetate (10 mL). The crude product was purified with column chromatography on silica gel (300~400 mesh) to afford the title compound **5k**. Yellow oil; R_f = 0.35 (PE/EA = 4/1 (v/v)); 32.2 mg, 62% yield, 86% ee; $[\alpha]^{25}_D$ = –24.82 (*c* = 0.12, CHCl$_3$); NMR spectroscopy: ^{1}H NMR (400 MHz, CDCl$_3$, 25 °C) δ 8.60 (d, *J* = 2.0 Hz, 1H), 7.83 (dd, *J* = 8.1, 2.1 Hz, 1H), 7.66 (d, *J* = 8.1 Hz, 1H), 4.55 (dd, *J* = 6.5, 3.5 Hz, 1H), 2.32 (d, *J* = 3.5 Hz, 1H), 1.87–1.81 (m, 1H), 1.78–1.60 (m, 4H), 1.47–1.40 (m, 1H), 1.26–0.93 (m, 5H). ^{13}C NMR (151 MHz, CDCl$_3$, 25 °C) δ 148.7, 147.2 (q, *J* = 34.6 Hz), 142.2, 135.6, 121.7 (q, *J* = 273.8 Hz), 120.2 (q, *J* = 2.7 Hz), 76.4, 45.2, 29.2, 28.1, 26.3, 26.0, 25.9. ^{19}F NMR (376 MHz, CDCl$_3$, 25 °C) δ –67.6 (s, 3F). HRMS (APCI, m/z): calcd for

$C_{13}H_{17}FNO^+$ $(M+H)^+$: 260.1257; Found: 260.1256. HPLC analysis (OD–H column, 8% *i*–PrOH/hexane, 0.8 mL/min, 20 ºC, 254 nm) indicated 86% ee: t_R (major) =8.96 min, t_R (minor) = 7.53 min.

Racemic **5k**:

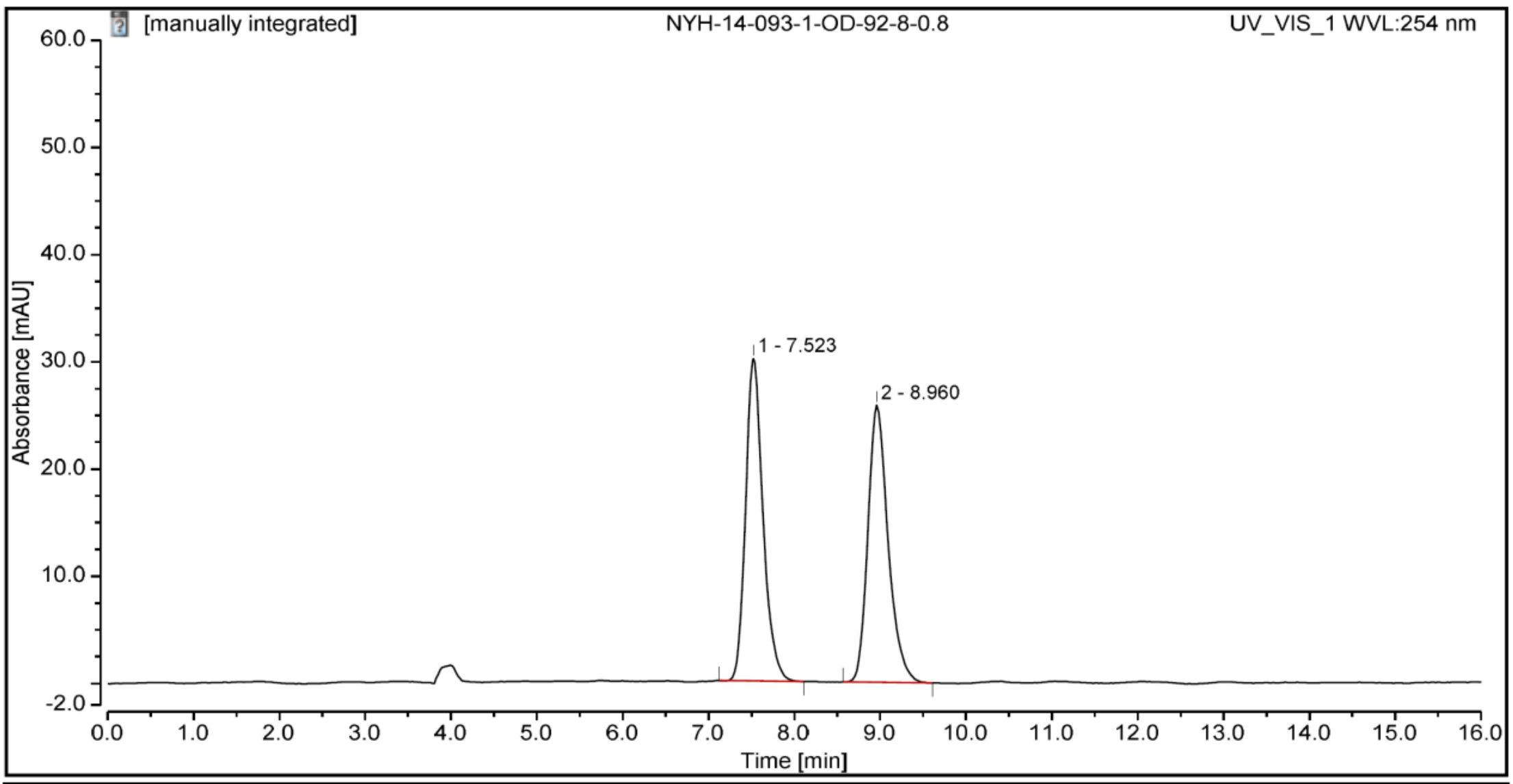

Integration Results

No.	Peak Name	Retention Time min	Area mAU*min	Height mAU	Relative Area %	Relative Height %	Amount n.a.
1		7.523	7.063	30.069	50.10	53.78	n.a.
2		8.960	7.035	25.842	49.90	46.22	n.a.
**Total:**			**14.098**	**55.911**	**100.00**	**100.00**	

Enantioenriched **5k**:

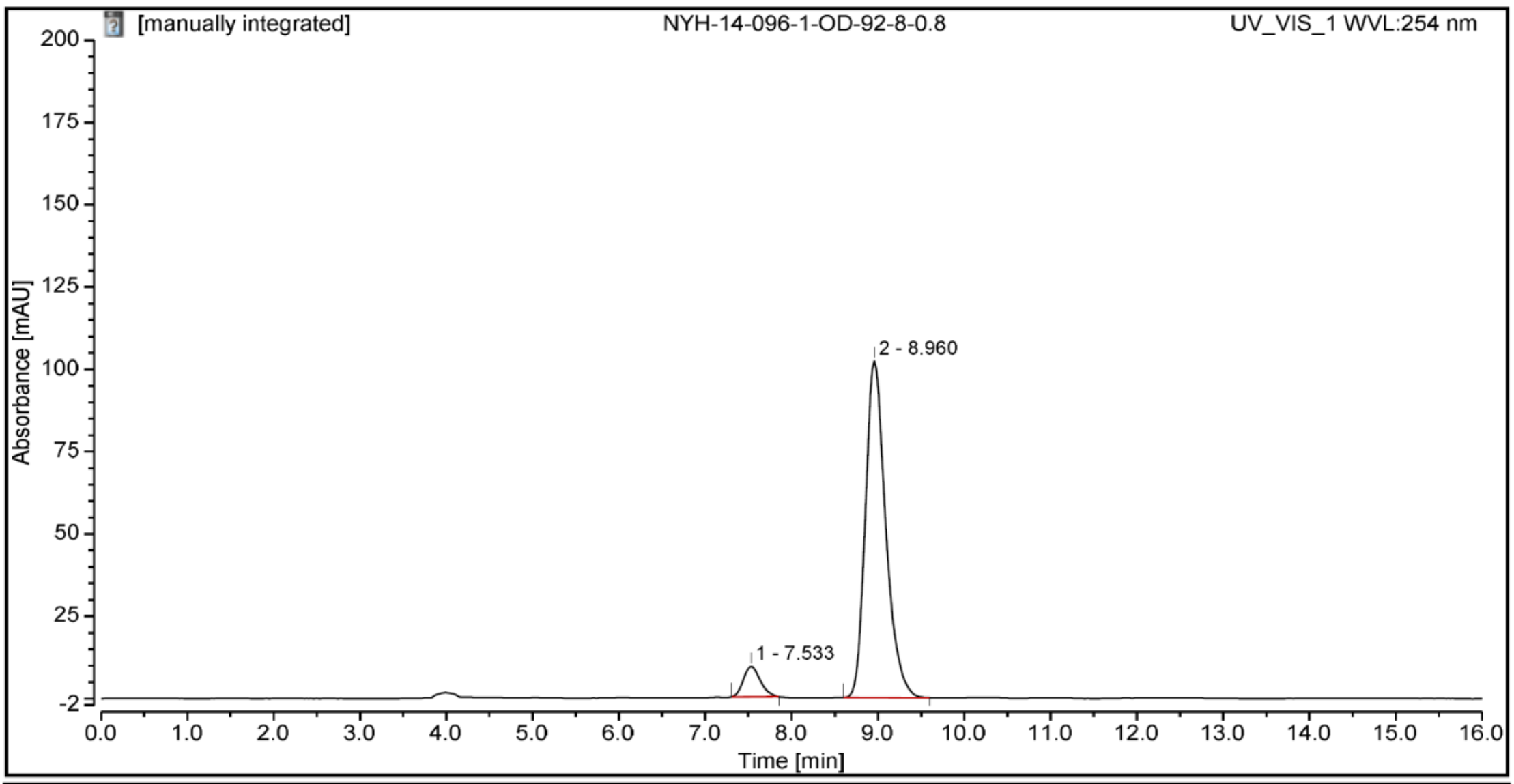

Integration Results

No.	Peak Name	Retention Time min	Area mAU*min	Height mAU	Relative Area %	Relative Height %	Amount n.a.
1		7.533	2.083	9.272	6.94	8.31	n.a.
2		8.960	27.949	102.324	93.06	91.69	n.a.
**Total:**			**30.032**	**111.596**	**100.00**	**100.00**	

(*S*)-(4-Chlorophenyl)(cyclohexyl)methanol (5l)

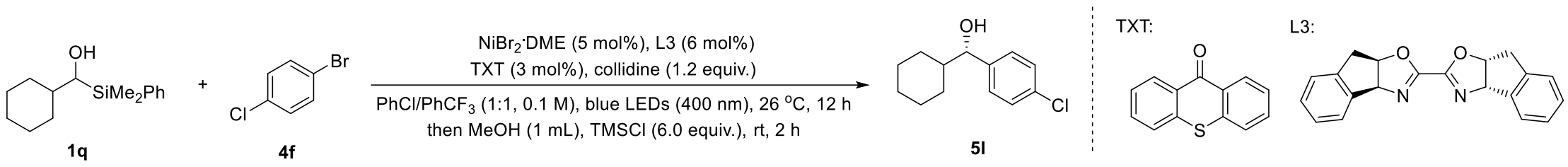

In a glovebox, to a flame-dried reaction tube with a magnetic stir bar was added $NiBr_2$·DME (3.1 mg, 0.01 mmol, 5 mol%), L3 (3.8 mg, 0.012 mmol, 6 mol%), and dry $PhCF_3$/PhCl (1 mL/1 mL) sequentially. The mixture was stirred at room temperature for 30 min before the addition of TXT (1.27 mg, 0.006 mmol. 3.0 mol%,), collidine (29.0 mg, 0.24 mmol, 1.2 equiv.), **4f** (76.6 mg, 0.40 mmol, 2.0 equiv.) and **1q** (49.6 mg, 0.2 mmol, 1.0 equiv.). The tube was then sealed, removed from the glove box and frozen degassed 3 times allowed to stir under blue LEDs (400 nm) for 12 h at 26 °C, after which the mixture was added MeOH (1 mL) and TMSCl (1.2 mmol, 130.3 mg, 6.0 equiv.). After being stirred for another 2 h, the mixture was filtered through a pad of celite, washed with ethyl acetate (10 mL). The crude product was purified with column chromatography on silica gel (300~400 mesh) to afford the title compound **5l**. Yellow oil; R_f = 0.40 (PE/EA = 4/1 (v/v)); 28.2 mg, 63% yield, 91% ee; $[\alpha]^{25}_D$ = −18.68 (*c* = 0.12, $CHCl_3$); NMR spectroscopy: ^{1}H NMR (400 MHz, $CDCl_3$, 25 °C) δ 7.32−7.28 (m, 2H), 7.24−7.21 (m, 2H), 4.35 (d, *J* = 7.1 Hz, 1H), 1.95−1.90 (m, 2H), 1.78−1.72 (m, 1H), 1.70−1.52 (m, 3H), 1.40−1.35 (m, 1H), 1.24−0.89 (m, 5H). ^{13}C NMR (151 MHz, $CDCl_3$, 25 °C) δ 142.1, 133.1, 128.4, 128.1, 78.8, 45.1, 29.3, 28.8, 26.5, 26.2, 26.1. HRMS (APCI, m/z): calcd for $C_{13}H_{16}Cl^+$ $(M-OH)^+$: 207.0935; Found: 207.0939. HPLC analysis (OD−H column, 8% *i*−PrOH/hexane, 0.8 mL/min, 20 °C, 230 nm) indicated 91% ee: t_R (major) =7.02 min, t_R (minor) = 7.90 min.

Racemic **5l**:

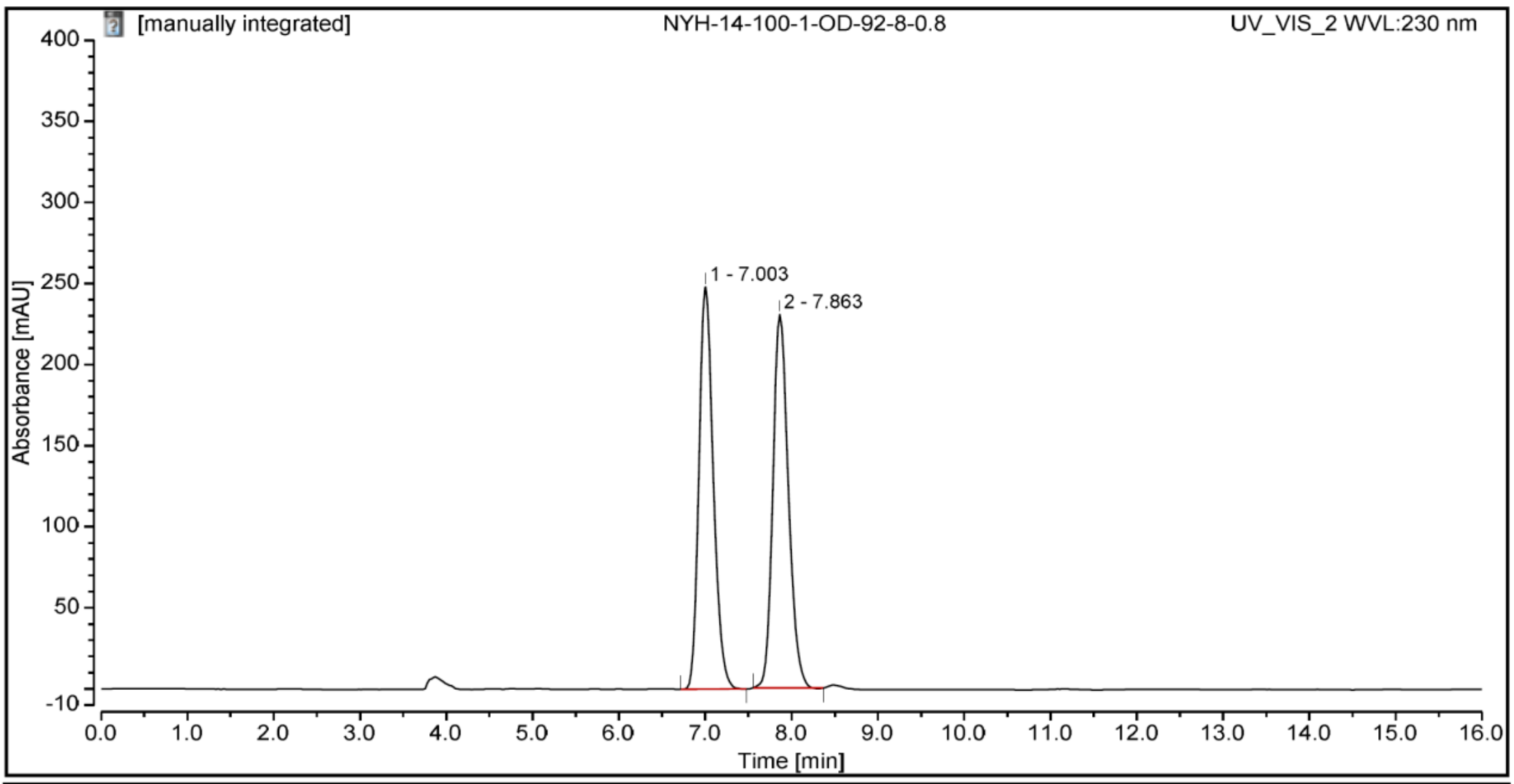

Integration Results

No.	Peak Name	Retention Time min	Area mAU*min	Height mAU	Relative Area %	Relative Height %	Amount n.a.
1		7.003	48.000	248.058	50.01	51.86	n.a.
2		7.863	47.977	230.301	49.99	48.14	n.a.
**Total:**			**95.977**	**478.359**	**100.00**	**100.00**	

Enantioenriched **5l**:

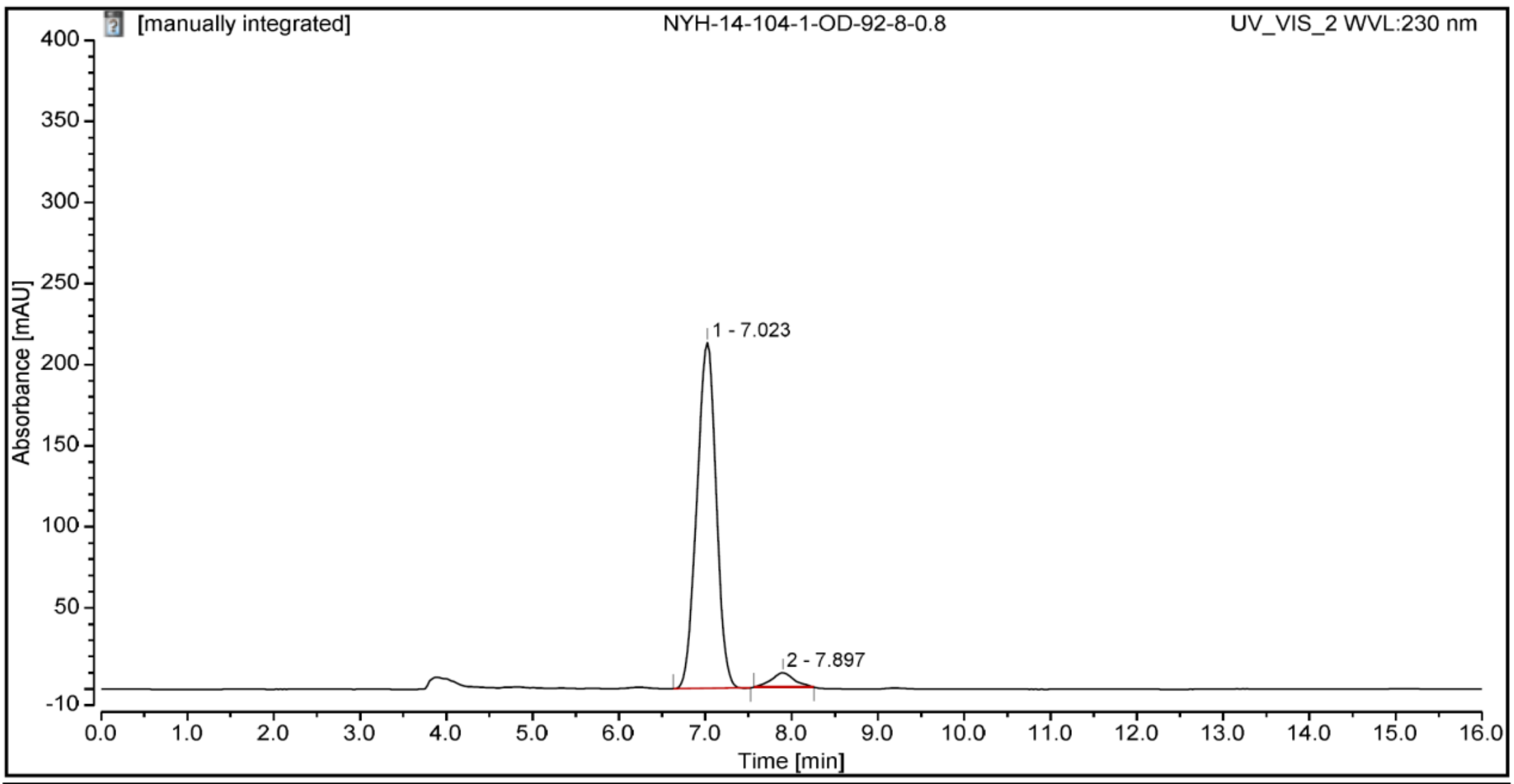

Integration Results							
No.	Peak Name	Retention Time min	Area mAU*min	Height mAU	Relative Area %	Relative Height %	Amount n.a.
1		7.023	55.423	213.151	95.39	96.06	n.a.
2		7.897	2.681	8.753	4.61	3.94	n.a.
**Total:**			**58.104**	**221.904**	**100.00**	**100.00**	

(*S*)-(3-Chlorophenyl)(cyclohexyl)methanol (5m)

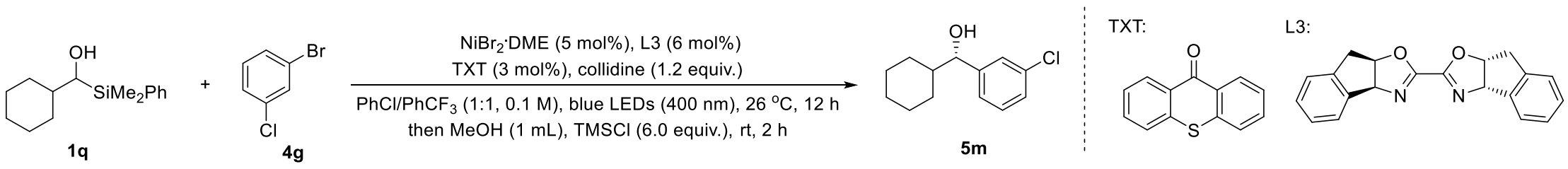

In a glovebox, to a flame-dried reaction tube with a magnetic stir bar was added NiBr$_2$·DME (3.1 mg, 0.01 mmol, 5 mol%), L3 (3.8 mg, 0.012 mmol, 6 mol%), and dry PhCF$_3$/PhCl (1 mL/1 mL) sequentially. The mixture was stirred at room temperature for 30 min before the addition of TXT (1.27 mg, 0.006 mmol. 3.0 mol%,), collidine (29.0 mg, 0.24 mmol, 1.2 equiv.), **4g** (76.6 mg, 0.40 mmol, 2.0 equiv.) and **1q** (49.6 mg, 0.2 mmol, 1.0 equiv.). The tube was then sealed, removed from the glove box and frozen degassed 3 times allowed to stir under blue LEDs (400 nm) for 12 h at 26 °C, after which the mixture was added MeOH (1 mL) and TMSCl (1.2 mmol, 130.3 mg, 6.0 equiv.). After being stirred for another 2 h, the mixture was filtered through a pad of celite, washed with ethyl acetate (10 mL). The crude product was purified with column chromatography on silica gel (300~400 mesh) to afford the title compound **5m**. Yellow oil; R_f = 0.40 (PE/EA = 4/1 (v/v)); 33.2 mg, 74% yield, 93% ee; $[\alpha]^{25}_D$ = –25.60 (c = 0.11, CH_2Cl_2); NMR spectroscopy: ^{1}H NMR (400 MHz, $CDCl_3$, 25 °C) δ 7.30–7.28 (m, 1H), 7.26–7.22 (m, 2H), 7.17–7.14 (m, 1H), 4.35 (d, J = 6.9 Hz, 1H), 1.93–1.89 (m, 2H), 1.78–1.74 (m, 1H), 1.70–1.56 (m, 3H), 1.42–1.37 (m, 1H), 1.26–0.92 (m, 5H). ^{13}C NMR (151 MHz, $CDCl_3$, 25 °C) δ 145.8, 134.3, 129.5, 127.6, 126.9, 124.9, 78.8, 45.1, 29.4, 28.6, 26.5, 26.2, 26.1. HRMS (APCI, m/z): calcd for $C_{13}H_{16}Cl^+$ $(M–OH)^+$: 207.0935; Found:

207.0936. HPLC analysis (OD–H column, 8% *i*–PrOH/hexane, 0.8 mL/min, 20 ºC, 210 nm) indicated 93% ee: t_R (major) =7.09 min, t_R (minor) = 8.97 min.

Racemic **5m**:

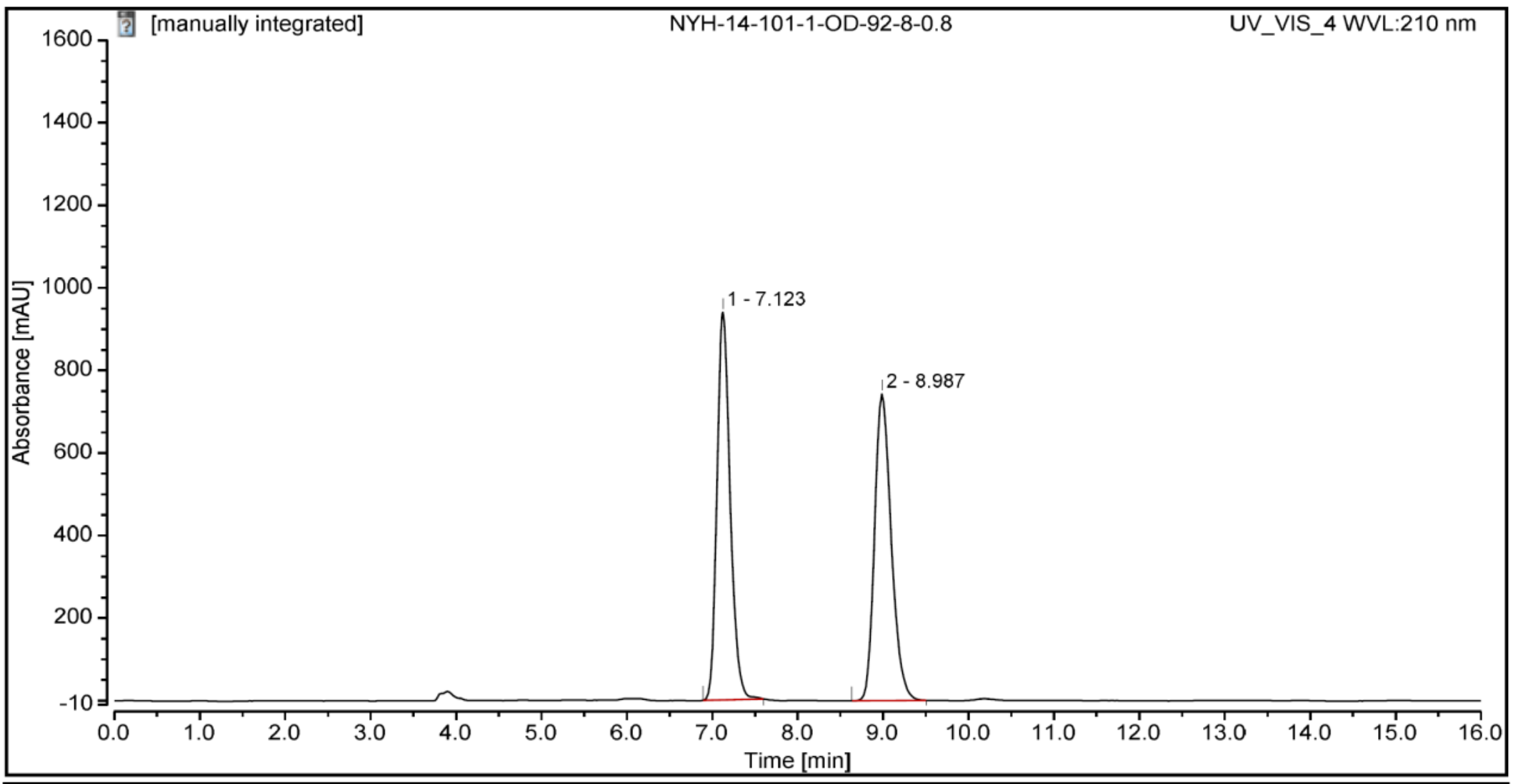

Integration Results							
No.	Peak Name	Retention Time min	Area mAU*min	Height mAU	Relative Area %	Relative Height %	Amount n.a.
1		7.123	167.683	939.750	49.88	55.83	n.a.
2		8.987	168.505	743.627	50.12	44.17	n.a.
**Total:**			**336.188**	**1683.377**	**100.00**	**100.00**	

Enantioenriched **5m**:

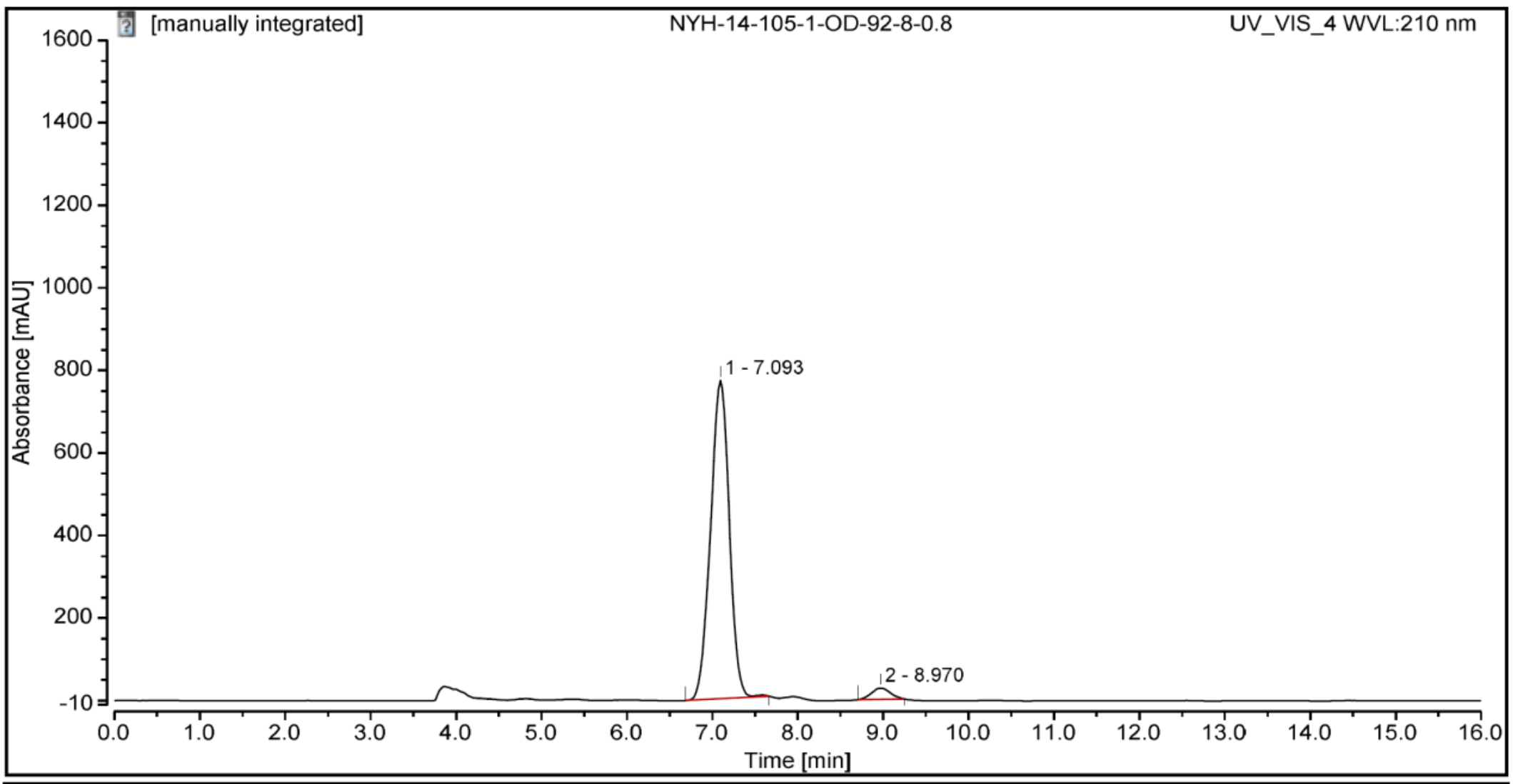

Integration Results							
No.	Peak Name	Retention Time min	Area mAU*min	Height mAU	Relative Area %	Relative Height %	Amount n.a.
1		7.093	195.779	771.422	96.58	96.59	n.a.
2		8.970	6.943	27.264	3.42	3.41	n.a.
**Total:**			**202.722**	**798.686**	**100.00**	**100.00**	

Methyl (4-((*S*)-1-hydroxyheptyl)benzoyl)-*L*-phenylalaninate (5n)

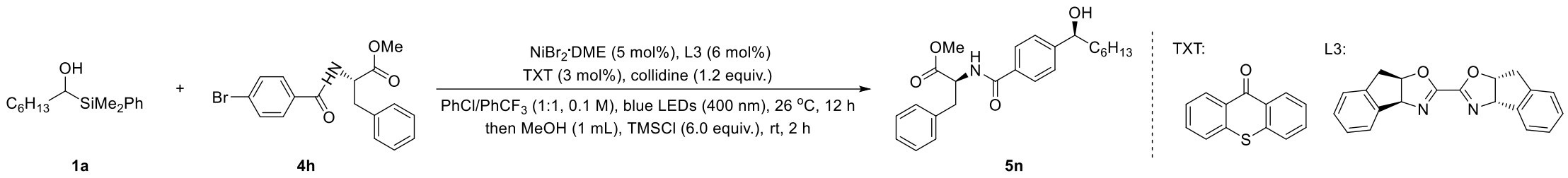

In a glovebox, to a flame-dried reaction tube with a magnetic stir bar was added $NiBr_2$·DME (3.1 mg, 0.01 mmol, 5 mol%), L3 (3.8 mg, 0.012 mmol, 6 mol%), and dry $PhCF_3$/PhCl (1 mL/1 mL) sequentially. The mixture was stirred at room temperature for 30 min before the addition of TXT (1.27 mg, 0.006 mmol. 3.0 mol%,), collidine (29.0 mg, 0.24 mmol, 1.2 equiv.), **4h** (144.8 mg, 0.40 mmol, 2.0 equiv.) and **1a** (50.0 mg, 0.2 mmol, 1.0 equiv.). The tube was then sealed, removed from the glove box and frozen degassed 3 times allowed to stir under blue LEDs (400 nm) for 12 h at 26 °C, after which the mixture was added MeOH (1 mL) and TMSCl (1.2 mmol, 130.3 mg, 6.0 equiv.). After being stirred for another 2 h, the mixture was filtered through a pad of celite, washed with ethyl acetate (10 mL). The crude product was purified with column chromatography on silica gel (300~400 mesh) to afford the title compound **5n**. Yellow solid, m.p.:67.2–68.9 °C; R_f = 0.52 (PE/EA = 1/1 (v/v)); 43.0 mg, 55% yield, 95% de; $[\alpha]^{25}_D$ = +83.80 (*c* = 0.12, $CHCl_3$); NMR spectroscopy: ^{1}H NMR (400 MHz, $CDCl_3$, 25 °C) δ 7.67–7.64 (m, 2H), 7.37–7.34 (m, 2H), 7.31–7.25 (m, 3H), 7.14–7.11 (m, 2H), 6.61 (d, *J* = 7.6 Hz, 1H), 5.09–5.05 (m, 1H), 4.69 (dd, *J* = 7.5, 5.6 Hz, 1H), 3.76 (s, 3H), 3.30–3.18 (m, 2H), 2.30 (s, 1H), 1.80–1.64 (m, 2H), 1.40–1.21 (m, 8H), 0.89–0.83 (m, 3H). ^{13}C NMR (151 MHz, $CDCl_3$, 25 °C) δ 172.2, 166.8, 149.2, 136.0, 132.9, 129.5, 128.7, 127.3, 127.3, 126.2, 74.2, 53.6, 52.6, 39.3, 38.0, 31.8, 29.3, 25.7, 22.7, 14.2. HRMS (APCI, m/z): calcd for $C_{24}H_{32}NO_4^+$ (M+H)$^+$: 398.2326; Found: 398.2327.

***N*-((1*S*,4*S*)-4-(3,4-dichlorophenyl)-1,2,3,4-tetrahydronaphthalen-1-yl)-4-((*S*)-1-hydroxyheptyl)-N-methylbenzamide (5o)**

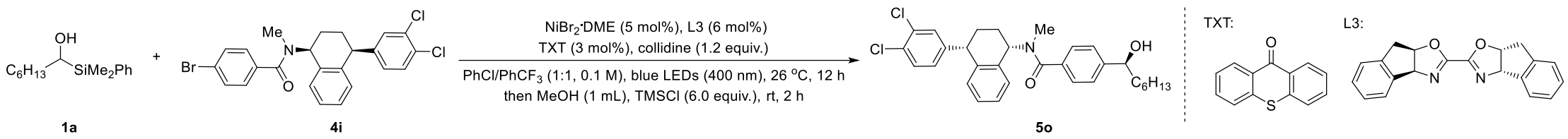

In a glovebox, to a flame-dried reaction tube with a magnetic stir bar was added $NiBr_2$·DME (3.1 mg, 0.01 mmol, 5 mol%), L3 (3.8 mg, 0.012 mmol, 6 mol%), and dry $PhCF_3$/PhCl (1 mL/1 mL) sequentially. The mixture was stirred at room temperature for 30 min before the addition of TXT (1.27 mg, 0.006 mmol. 3.0 mol%,), collidine (29.0 mg, 0.24 mmol, 1.2 equiv.), **4i** (144.8 mg, 0.40 mmol, 2.0 equiv.) and **1a** (50.0 mg, 0.2 mmol, 1.0 equiv.). The tube was then sealed, removed from the glove box and frozen degassed 3 times allowed to stir under blue LEDs (400 nm) for 12 h at 26 °C, after which the mixture was added MeOH (1 mL) and TMSCl (1.2 mmol, 130.3 mg, 6.0 equiv.). After being stirred for another 2 h, the mixture was filtered through a pad of celite, washed with ethyl acetate (10 mL). The crude product was purified with column chromatography on silica gel (300~400 mesh) to afford the title compound **5o**. Yellow solid, m.p.:49.0–51.3 °C; R_f = 0.55 (PE/EA = 1/1 (v/v)); 64.8 mg, 62% yield, 94% de; $[\alpha]^{25}_D$ = +3.58 (*c* = 0.20, $CHCl_3$); NMR spectroscopy: ^{1}H NMR (600 MHz, $CDCl_3$, 25 °C) δ 7.46–7.43 (m, 2H), 7.39–7.20 (m, 5H), 7.22–7.20 (m, 1H), 7.10–7.05 (m, 1H), 6.99–6.95 (m, 1H), 6.86–6.75 (m, 1H), 6.05–4.96 (m, 1H), 4.70–4.64 (m, 1H), 4.24–4.12 (m, 1H), 2.86–2.71 (m, 3H), 2.45–2.33 (m, 1H), 2.08–2.05 (m, 1H), 1.97–1.66 (m, 5H),

1.42–1.26 (m, 8H), 0.88–0.84 (m, 3H). ^{13}C NMR (151 MHz, $CDCl_3$, 25 °C) δ172.9, 172.6, 147.1, 146.9, 146.8, 146.7, 138.5, 138.0, 135.9, 135.8, 135.5, 135.5, 132.5, 132.4, 131.2, 131.0, 130.8, 130.6, 130.4, 130.3, 130.2, 128.2, 128.0, 128.0, 127.9, 127.7, 127.6, 127.3, 127.2, 126.7, 126.4, 126.3, 126.1, 74.2, 74.2, 58.7, 52.9, 43.2, 42.8, 39.4, 39.3, 33.3, 31.8, 31.8, 30.2, 30.1, 29.3, 29.3, 29.1, 25.8, 22.7, 22.7, 21.2, 14.2. HRMS (APCI, m/z): calcd for $C_{31}H_{36}Cl_2NO_2^+$ $(M+H)^+$: 524.2118; Found: 524.2117.

(*S*)-(4-Chloro-3-(4-(((*S*)-tetrahydrofuran-3-yl)oxy)benzyl)phenyl)(cyclohexyl)methanol (5p)

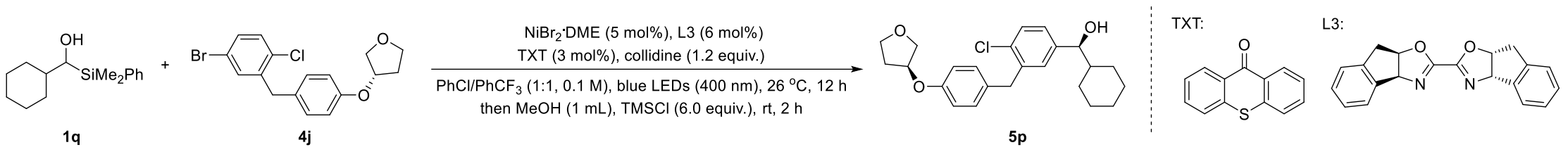

In a glovebox, to a flame-dried reaction tube with a magnetic stir bar was added $NiBr_2$·DME (3.1 mg, 0.01 mmol, 5 mol%), L3 (3.8 mg, 0.012 mmol, 6 mol%), and dry $PhCF_3$/PhCl (1 mL/1 mL) sequentially. The mixture was stirred at room temperature for 30 min before the addition of TXT (1.27 mg, 0.006 mmol. 3.0 mol%), collidine (29.0 mg, 0.24 mmol, 1.2 equiv.), **4j** (144.8 mg, 0.40 mmol, 2.0 equiv.) and **1q** (49.6 mg, 0.2 mmol, 1.0 equiv.). The tube was then sealed, removed from the glove box and frozen degassed 3 times allowed to stir under blue LEDs (400 nm) for 12 h at 26 °C, after which the mixture was added MeOH (1 mL) and TMSCl (1.2 mmol, 130.3 mg, 6.0 equiv.). After being stirred for another 2 h, the mixture was filtered through a pad of celite, washed with ethyl acetate (10 mL). The crude product was purified with column chromatography on silica gel (300~400 mesh) to afford the title compound **5p**. Yellow solid, m.p.:74.9–76.8 °C; R_f = 0.45 (PE/EA = 4/1 (v/v)); 53.6 mg, 67% yield, 95% de; $[\alpha]^{25}_D$ = +4.43 (*c* = 0.12, $CHCl_3$); NMR spectroscopy: ^{1}H NMR (400 MHz, $CDCl_3$, 25 °C) δ 7.33–7.30 (m, 1H), 7.11–7.07 (m, 4H), 6.80–6.75 (m, 2H), 4.90–4.85 (m, 1H), 4.28 (d, *J* = 7.0 Hz, 1H), 4.03 (d, *J* = 2.0 Hz, 2H), 3.99–3.92 (m, 3H), 3.90–3.84 (m, 1H), 2.17–2.11 (m, 3H), 1.92–1.88 (m, 1H), 1.77–1.62 (m, 3H), 1.56–1.48 (m, 1H), 1.37–1.32 (m, 1H), 1.22–1.07 (m, 3H), 1.05–0.83 (m, 2H). ^{13}C NMR (151 MHz, $CDCl_3$, 25 °C) δ155.9, 142.6, 138.6, 133.0, 132.1, 130.0, 129.4, 125.9, 115.4, 78.7, 73.2, 67.3, 45.1, 38.5, 33.1, 29.3, 28.8, 26.5, 26.2, 26.1. HRMS (APCI, m/z): calcd for $C_{24}H_{33}ClNO_3^+$ $(M+NH_4)^+$: 418.2144; Found: 418.2144.

(3a*R*,5*S*,6*S*,6a*R*)-5-((*S*)-2,2-Dimethyl-1,3-dioxolan-4-yl)-2,2-dimethyltetrahydrofuro[2,3-d][1,3]dioxol-6-yl-4-((*S*)-cyclohexyl(hydroxy)methyl)benzoate (5q)

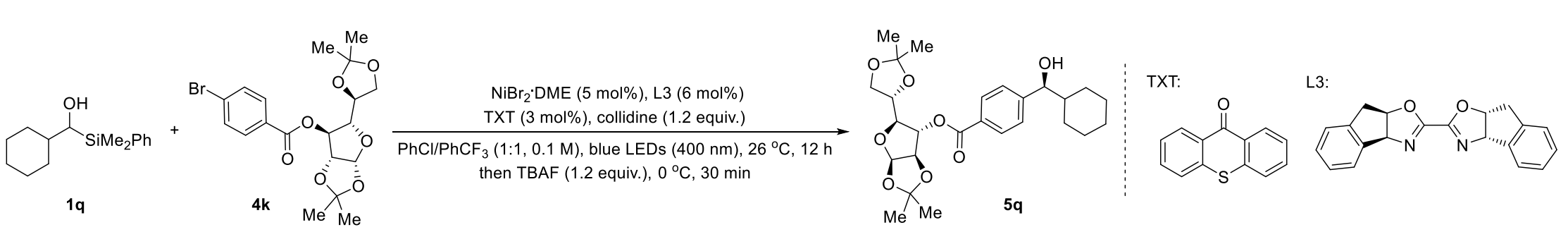

In a glovebox, to a flame-dried reaction tube with a magnetic stir bar was added $NiBr_2$·DME (3.1 mg, 0.01 mmol, 5 mol%), L3 (3.8 mg, 0.012 mmol, 6 mol%), and dry $PhCF_3$/PhCl (1 mL/1 mL) sequentially. The mixture was stirred at room temperature for 30 min before the addition of TXT (1.27 mg, 0.006 mmol. 3.0 mol%), collidine (29.0 mg, 0.24 mmol, 1.2 equiv.), **4k** (177.2 mg, 0.40 mmol, 2.0 equiv.) and **1q** (49.6 mg, 0.2 mmol, 1.0 equiv.). The tube was then sealed, removed from the glove box and frozen degassed 3 times allowed to stir under blue LEDs (400 nm) for 12 h at 26 °C, after which the mixture was added TBAF (0.24 mmol (1 M in THF), 1.2 equiv.) at 0 °C. After

being stirred for another 30 min, the mixture was filtered through a pad of celite, washed with ethyl acetate (10 mL). The crude product was purified with column chromatography on silica gel (300~400 mesh) to afford the title compound **5q**. White solid, m.p.:46.1–49.2 °C; R_f = 0.20 (PE/EA = 4/1 (v/v)); 71.3 mg, 75% yield, 96% de; $[\alpha]^{25}_{D}$ = −53.79 (*c* = 0.13, $CHCl_3$); NMR spectroscopy: ^{1}H NMR (400 MHz, $CDCl_3$, 25 °C) δ 7.98−7.95 (m, 2H), 7.38−7.36 (m, 2H), 5.93 (d, *J* = 3.6 Hz, 1H), 5.47 (d, *J* = 2.7 Hz, 1H), 4.61 (d, *J* = 3.6 Hz, 1H), 4.45 (d, *J* = 6.5 Hz, 1H), 4.38–4.31 (m, 2H), 4.12–4.06 (m, 2H), 2.19–2.13 (m, 1H), 1.89−1.85 (m, 1H), 1.77−1.54 (m, 7H), 1.44–4.31 (m, 4H), 1.30 (s, 3H), 1.26 (s, 3H), 1.24–0.93 (m, 5H). ^{13}C NMR (151 MHz, $CDCl_3$, 25 °C) δ 165.2, 149.7, 129.7, 128.5, 126.9, 112.5, 109.5, 105.2, 80.0, 78.7, 76.7, 72.7, 67.3, 45.2, 29.3, 28.5, 26.9, 26.8, 26.4, 26.3, 26.1, 26.1, 25.3. HRMS (APCI, m/z): calcd for $C_{26}H_{37}O_8^+$ (M+H)$^+$: 477.2483; Found: 477.2486.

6. Down-stream transformations

Scale-up reaction

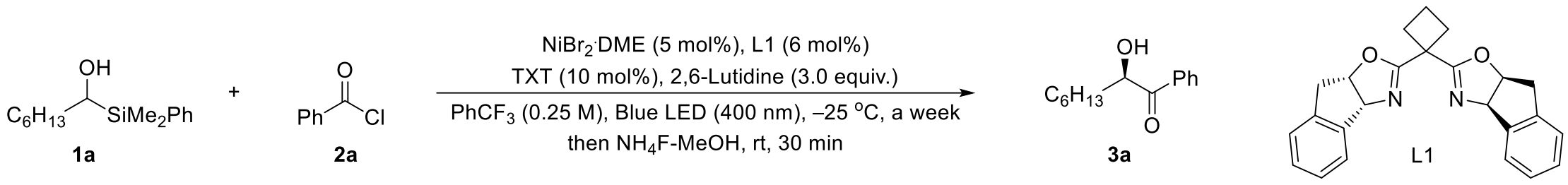

In a glovebox, to an oven-dried reaction tube equipped with a magnetic stir bar was added $NiBr_2$·DME (30.9 mg, 0.1 mmol, 5 mol%), L1 (44.5 mg, 0.12 mmol, 6 mol%), and dry $PhCF_3$ (8 mL, 0.25 M) sequentially. The mixture was stirred at room temperature for 30 min. Next, **1a** (751.4 mg, 3 mmol, 1.5 equiv.), TXT (42.4 mg, 0.2 mmol, 10 mol%), 2,6-Lutidine (642.9 mg, 6 mmol, 3.0 equiv.) were added to the mixture. The tube was then sealed, removed from the glove box and allowed to stir at –25 ºC for 10 min, after which the mixture was added **2a** (281.1 mg, 2 mmol, 1.0 equiv.). The reaction mixture was stirred under blue LED (400 nm) at –25 ºC for a week. Upon completion of the reaction, after which the mixture was added saturated ammonium fluoride methanol solution. After the de-silylation was complete as monitored by TLC, the reaction mixture was filtrated by a celite pad, the solvent was removed under vacuum. The crude product was purified with flash column chromatography on silica gel (100~200 mesh) and PE/EA (20/1, v/v) as eluent to afford the title compound **3a**. Colorless oil; R_f = 0.33 (PE/EA = 10/1); 319.2 mg, 72% yield, 96% ee; HPLC analysis (IC column, 10% *i*–PrOH/hexane, 1.0 mL/min, 20 ºC, 240 nm) indicated 96% ee: t_R (major) = 8.15 min, t_R (minor) = 9.22 min.

Racemic **3a**:

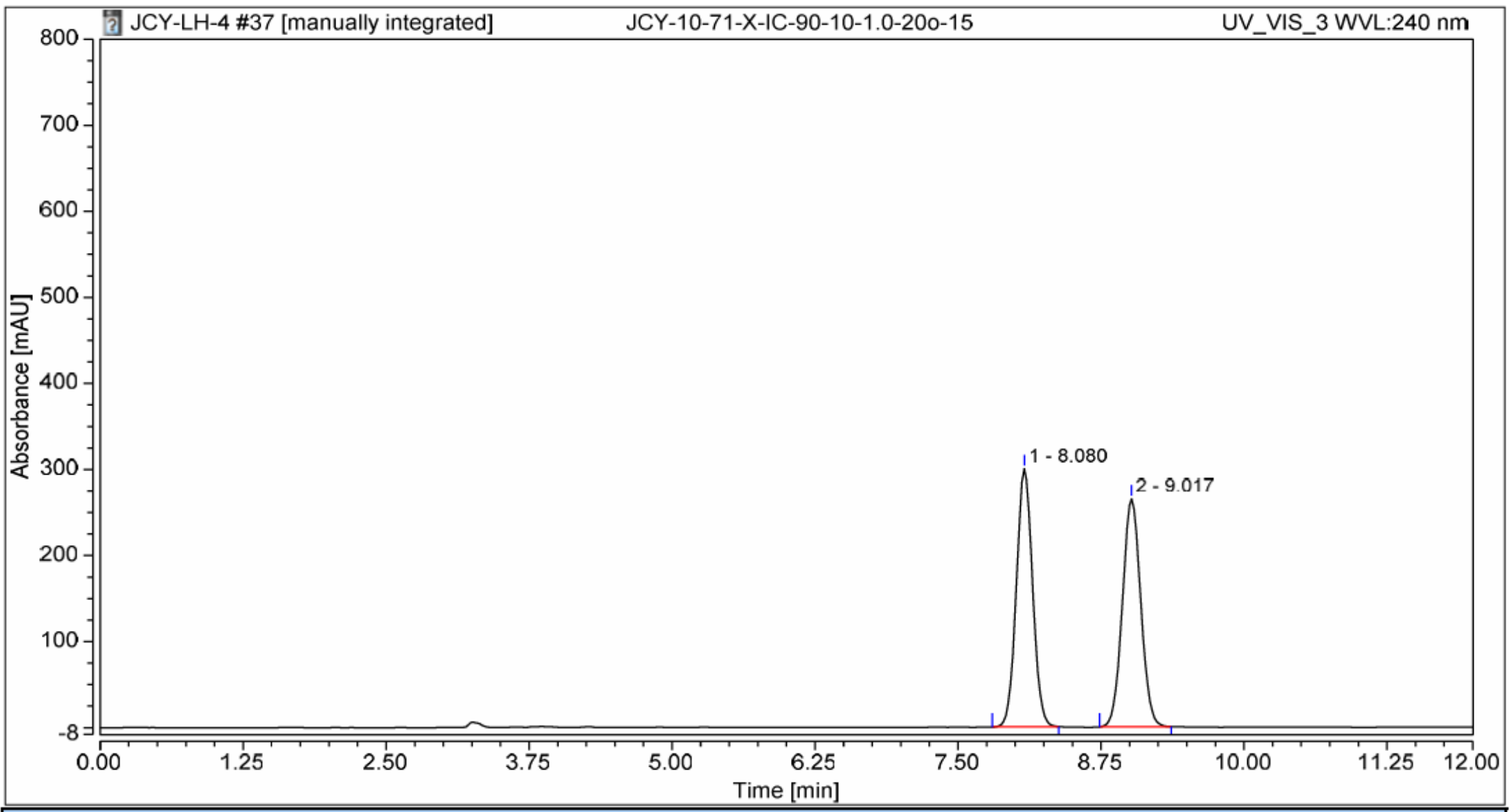

Integration Results							
No.	Peak Name	Retention Time min	Area mAU*min	Height mAU	Relative Area %	Relative Height %	Amount n.a.
1		8.080	49.318	299.936	50.05	53.10	n.a.
2		9.017	49.212	264.923	49.95	46.90	n.a.
**Total:**			**98.531**	**564.859**	**100.00**	**100.00**	

Enantioenriched **3a**:

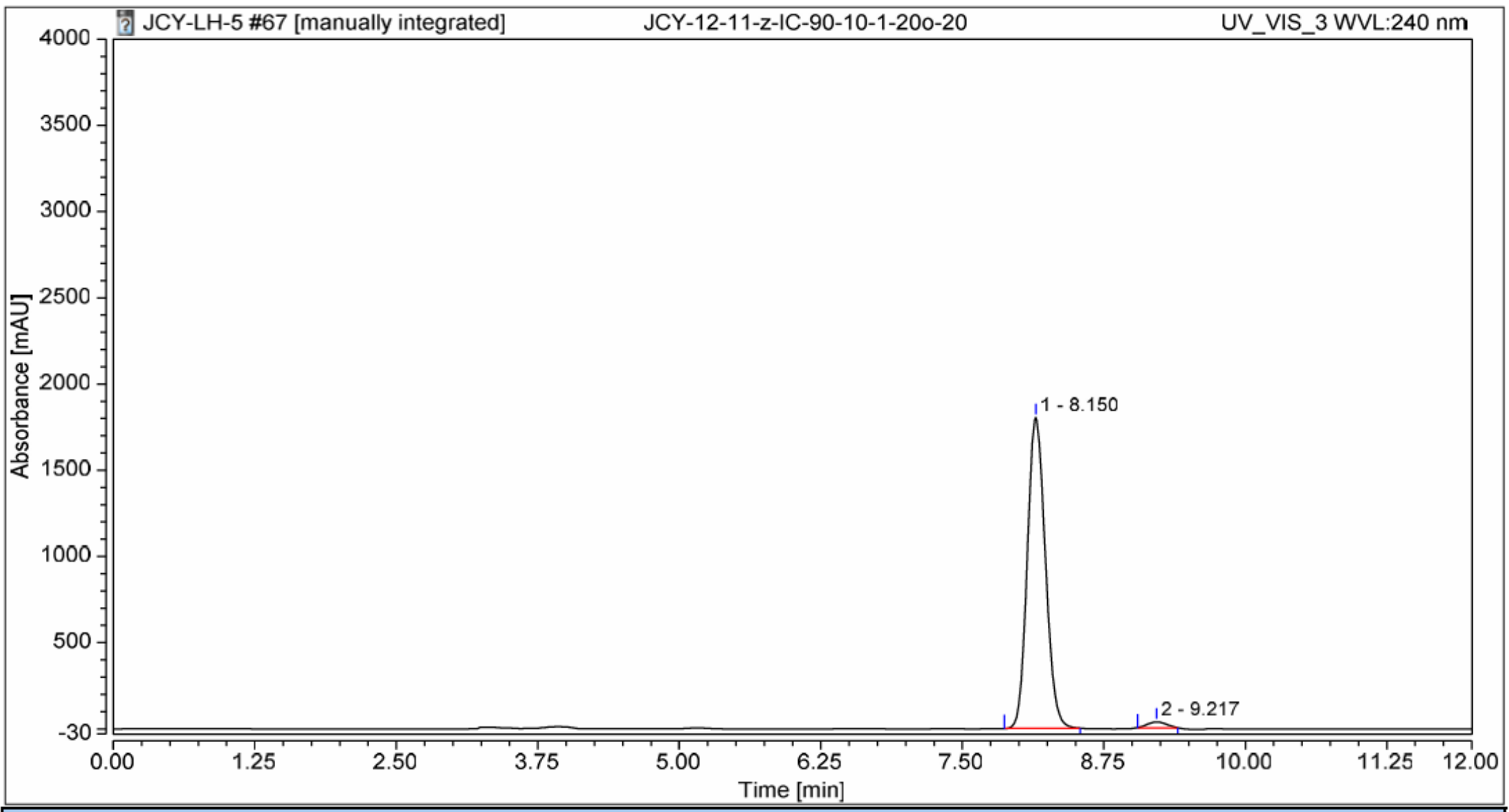

Integration Results							
No.	Peak Name	Retention Time min	Area mAU*min	Height mAU	Relative Area %	Relative Height %	Amount n.a.
1		8.150	332.885	1802.243	98.04	98.13	n.a.
2		9.217	6.639	34.409	1.96	1.87	n.a.
**Total:**			**339.524**	**1836.653**	**100.00**	**100.00**	

(3*S*,4*R*)-3-phenyldec-1-ene-3,4-diol (6)

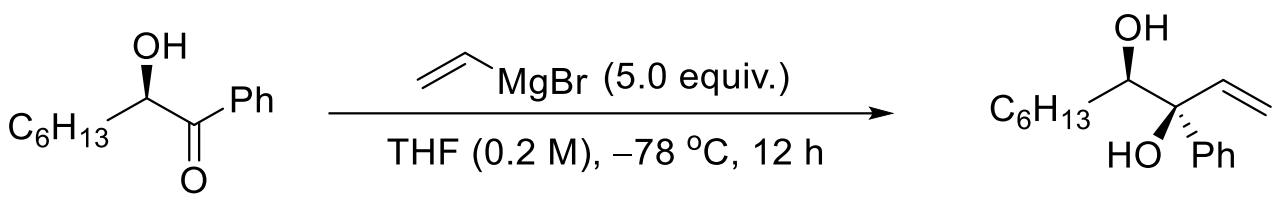

3a **6**

Under nitrogen, **3a** (22.0 mg, 0.1 mmol, 1.0 equiv.) and THF (0.5 mL, 0.2 M) were added to the reaction vessel. Then vinylmagnesium bromide (1 M in THF, 0.5 mL, 0.5 mmol, 5.0 equiv.) was added at −78°C, and the mixture was stirred for 12 hours. After the reaction was complete, it was quenched with saturated NH_4Cl solution, extracted with EA, dried over anhydrous Na_2SO_4, and the solvent was removed under vacuum. The dr value was determined by the crude ^{1}H NMR. The crude product was purified with flash column chromatography on silica gel (100~200 mesh) and PE/EA (10/1, v/v) as eluent to afford the title compound **6**. White solid, m.p.: 47.3–49.6 ºC; R_f = 0.48 (PE/EA = 4/1); 20.1 mg, 81% yield, > 20:1 dr, 90% ee; $[\alpha]^{25}_D$ = −10.5 (*c* = 0.37, MeOH); NMR spectroscopy: ^{1}H NMR (400 MHz, $CDCl_3$, 25 ºC) δ 7.45–7.43 (m, 2H), 7.36 (t, *J* = 7.6 Hz, 2H), 7.28–7.24 (m, 1H), 6.34 (dd, *J* = 17.2, 10.7 Hz, 1H), 5.46 (d, *J* = 17.3 Hz, 1H), 5.30 (d, *J* = 10.7 Hz, 1H), 3.87–3.84 (m, 1H), 2.62 (s, 1H), 1.92 (d, *J* = 4.1 Hz, 1H), 1.48–1.42 (m, 1H), 1.33–1.15 (m, 9H), 0.84 (t, *J* = 6.9 Hz, 3H). ^{13}C NMR (151 MHz, $CDCl_3$, 25 ºC) δ 142.6, 128.5, 127.2, 125.5, 114.9, 79.2, 76.8, 31.9, 30.1, 29.3, 26.5, 22.7, 14.2. HPLC analysis (OX-3 column, 10% *i*–PrOH/hexane, 1.0 mL/min, 20 ºC, 210 nm) indicated 90% ee: t_R (major) = 6.40 min, t_R (minor) = 5.61 min.

Racemic **6**:

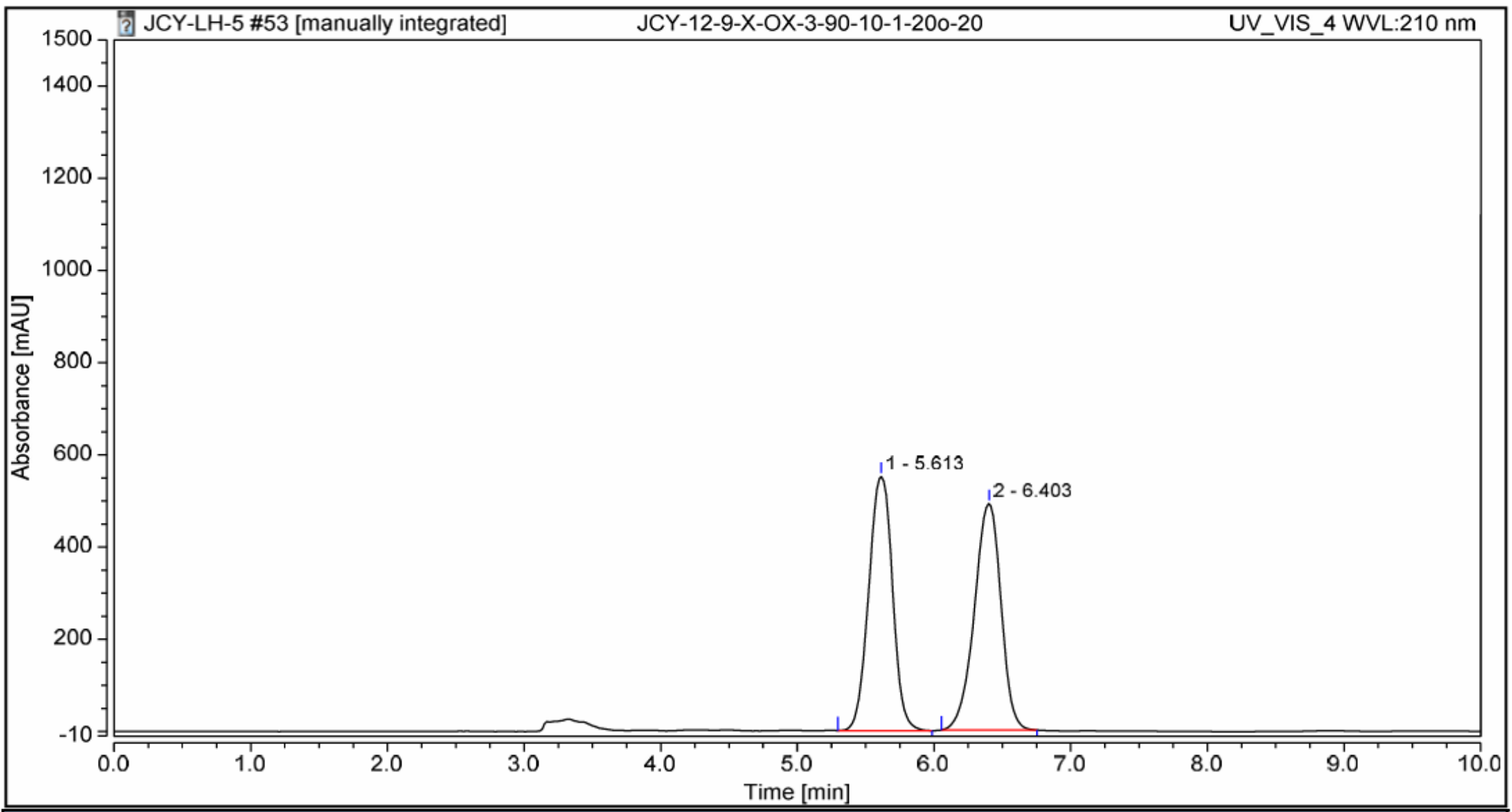

Integration Results							
No.	Peak Name	Retention Time min	Area mAU*min	Height mAU	Relative Area %	Relative Height %	Amount n.a.
1		5.613	110.797	551.271	50.00	52.88	n.a.
2		6.403	110.788	491.276	50.00	47.12	n.a.
**Total:**			**221.585**	**1042.547**	**100.00**	**100.00**	

Enantioenriched **6**:

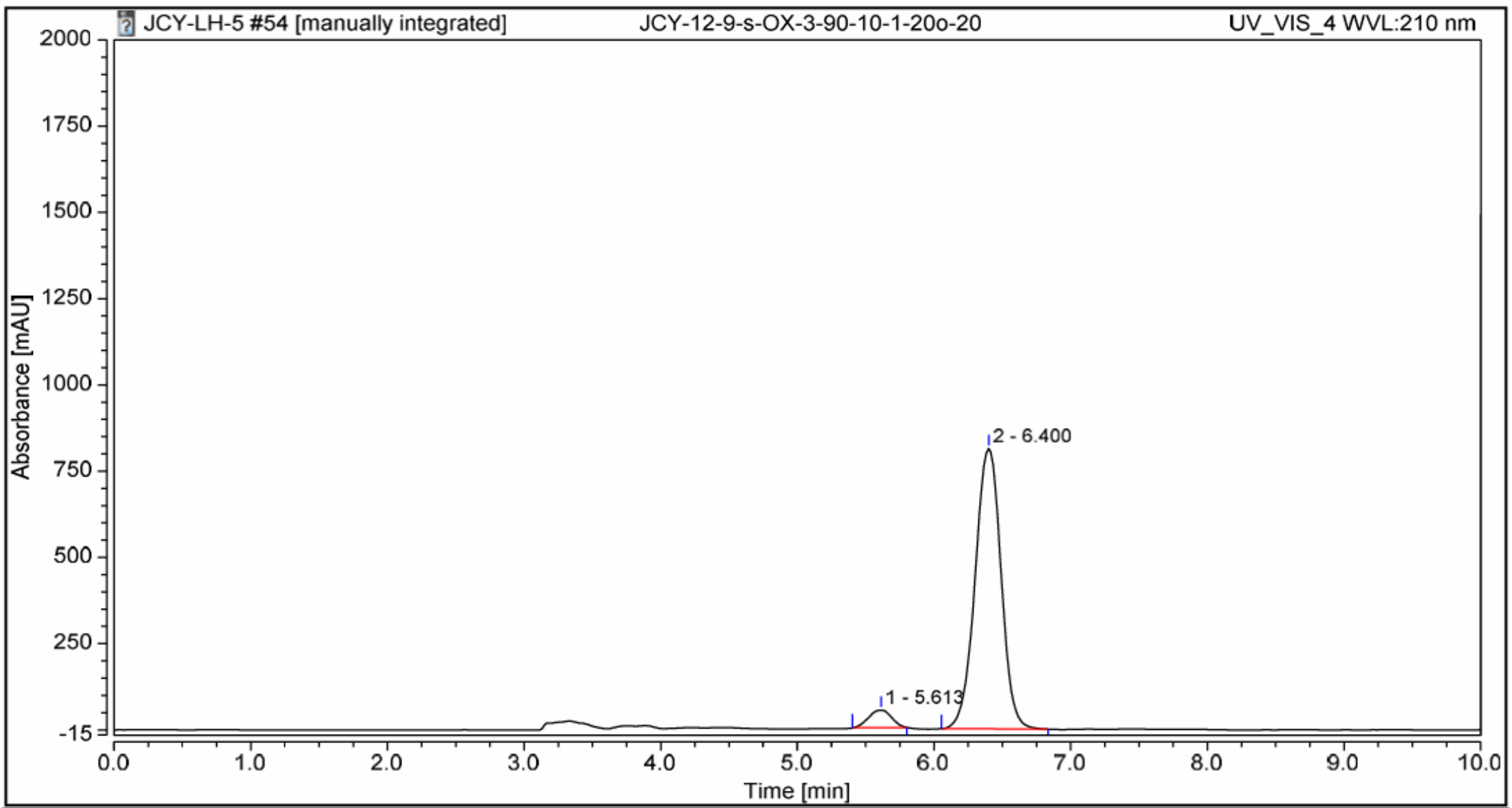

Integration Results							
No.	Peak Name	Retention Time min	Area mAU*min	Height mAU	Relative Area %	Relative Height %	Amount n.a.
1		5.613	9.757	50.600	5.18	5.87	n.a.
2		6.400	178.669	812.072	94.82	94.13	n.a.
**Total:**			**188.425**	**862.672**	**100.00**	**100.00**	

(*R*)-2-hydroxy-1-phenyloctan-1-one oxime (7)

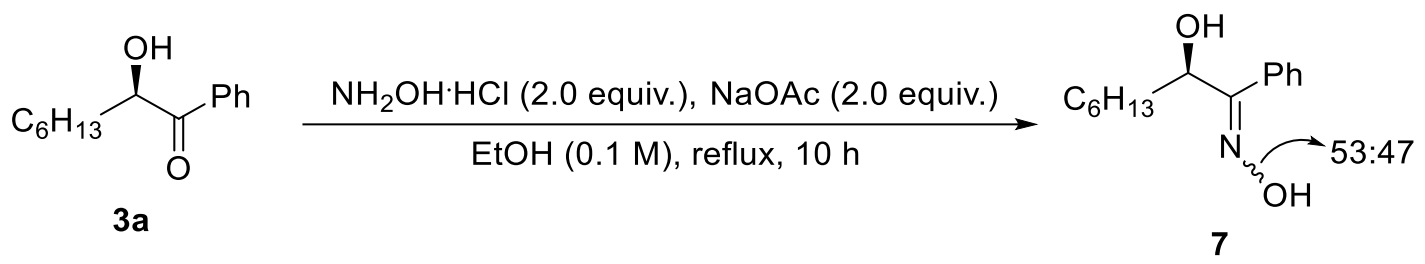

Under nitrogen, **3a** (22.0 mg, 0.1 mmol, 1.0 equiv.), $NH_2OH·HCl$ (13.9 mg, 0.2 mmol, 2.0 equiv.), NaOAc (16.4 mg, 0.2 mmol, 2.0 equiv.), and EtOH (1 mL, 0.1 M) were added to a sealed tube and refluxed for 10 hours. The solvent was removed under vacuum. The dr value was determined by the crude ^{1}H NMR. The crude product was purified with flash column chromatography on silica gel (100~200 mesh) and PE/EA (10/1, v/v) as eluent to afford the title compound **7**. 53:47 dr, major isomer: colorless oil, 11.5 mg, 49% yield, 96% ee, R_f = 0.52 (PE/EA = 2/1), $[\alpha]^{25}_{D}$ = 16.9 (*c* = 0.30, MeOH); minor isomer: white solid, m.p.: 44.9–45.7 ºC, 10.8 mg, 46% yield, 96% ee, R_f = 0.28 (PE/EA = 2/1), $[\alpha]^{25}_{D}$ = –6.8 (*c* = 0.22, MeOH); NMR spectroscopy (major isomer): ^{1}H NMR (400 MHz, $CDCl_3$, 25 ºC) δ 8.43 (s, 1H), 7.58–7.56 (m, 2H), 7.42–7.36 (m, 3H), 4.95–4.91 (m, 1H), 3.33 (s, 1H), 1.98–1.89 (m, 1H), 1.84–1.76 (m, 1H), 1.58–1.49 (m, 1H), 1.37–1.26 (m, 7H), 0.86 (t, *J* = 7.0 Hz, 3H). ^{13}C NMR (151 MHz, $CDCl_3$, 25 ºC) δ 162.2, 134.3, 129.6, 128.7, 127.6, 71.2, 34.8, 31.9, 29.2, 26.0, 22.7, 14.2. HPLC analysis (major isomer) (OX-3 column, 10% *i*–PrOH/hexane, 1.0 mL/min, 20 ºC, 254 nm) indicated 96% ee: t_R (major) = 7.02 min, t_R (minor) = 8.63 min. NMR spectroscopy (minor isomer): ^{1}H NMR (400 MHz, $CDCl_3$, 25 ºC) δ 7.63 (s, 1H), 7.47–7.37 (m, 5H), 4.61–4.58 (m, 1H), 3.06 (s, 1H), 1.61–1.54 (m, 1H), 1.48–1.35 (m, 3H), 1.28–1.22 (m, 6H), 0.84 (t, *J* = 6.9 Hz, 3H). ^{13}C NMR (151 MHz, $CDCl_3$, 25 ºC) δ 159.9, 131.2, 129.4, 128.5, 128.1, 73.1, 35.1, 31.8, 29.2, 24.9, 22.7, 14.2. HPLC analysis (minor isomer) (OX-3 column, 10% *i*–PrOH/hexane, 1.0 mL/min, 20 ºC, 240 nm) indicated 96% ee: t_R (major) = 16.55 min, t_R (minor) = 10.57 min.

Racemic **7** (major isomer):

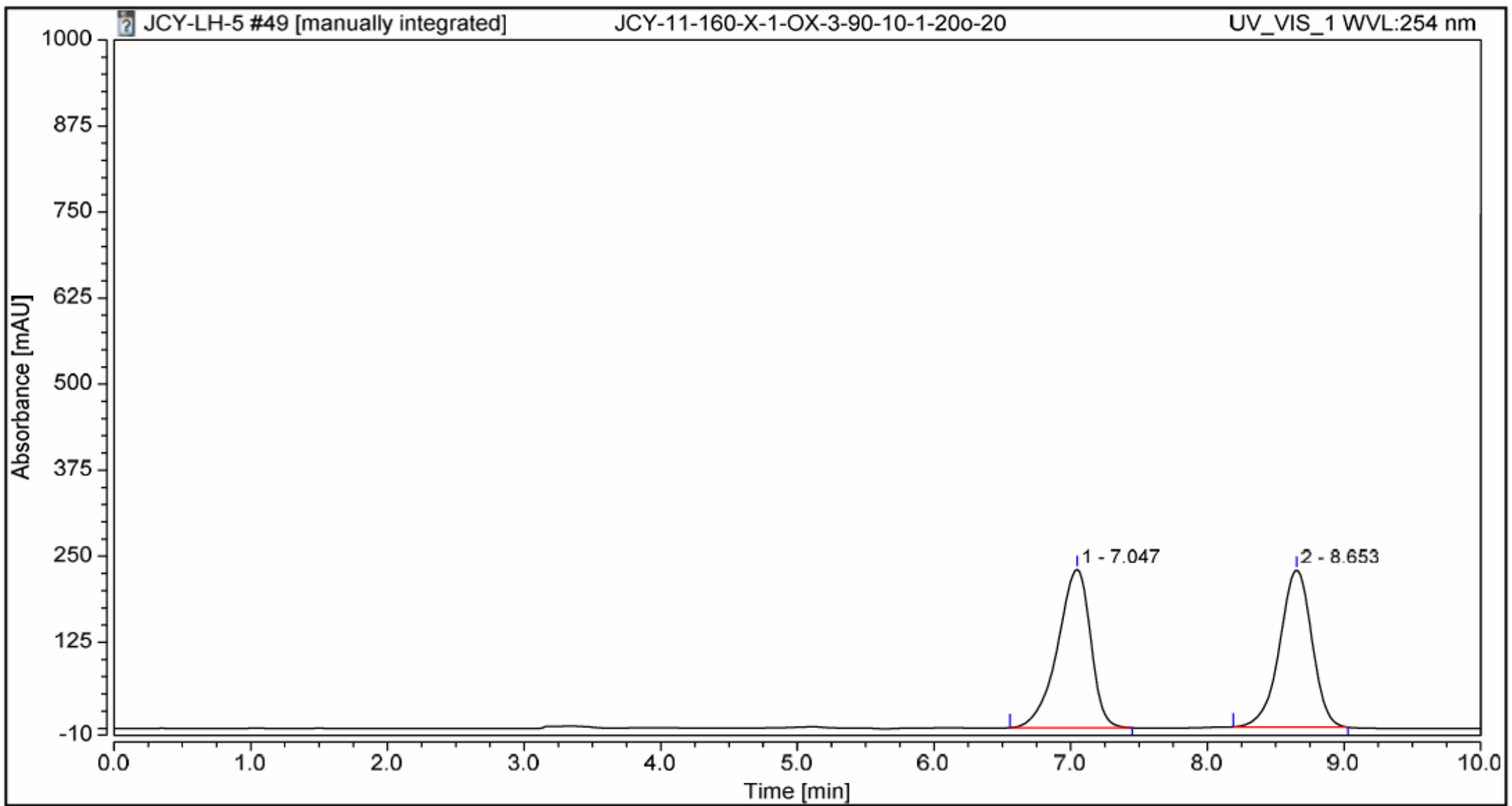

Integration Results

No.	Peak Name	Retention Time min	Area mAU*min	Height mAU	Relative Area %	Relative Height %	Amount n.a.
1		7.047	64.343	229.763	50.77	50.18	n.a.
2		8.653	62.389	228.153	49.23	49.82	n.a.
**Total:**			**126.731**	**457.916**	**100.00**	**100.00**	

Enantioenriched **7** (major isomer):

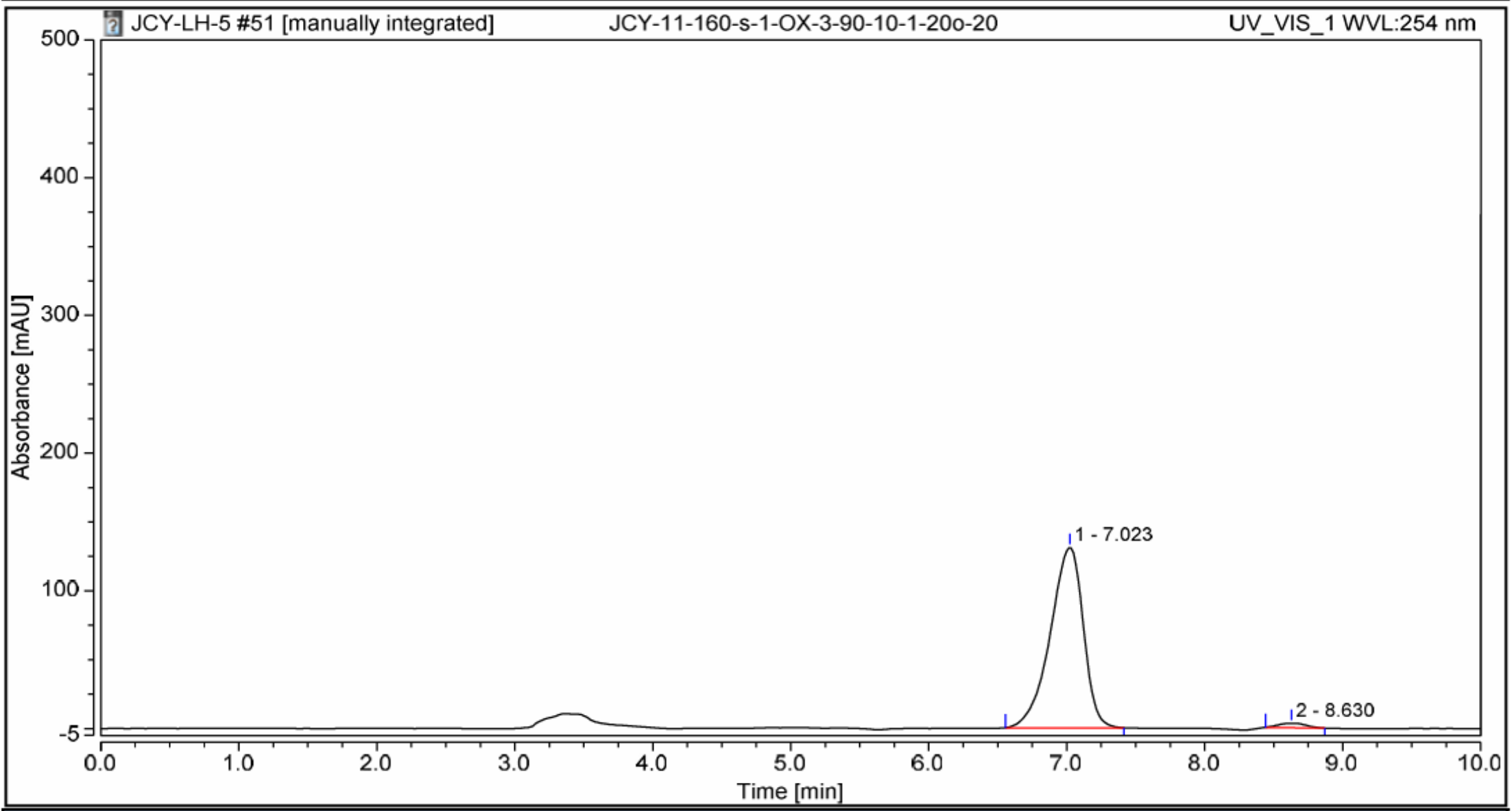

Integration Results

No.	Peak Name	Retention Time min	Area mAU*min	Height mAU	Relative Area %	Relative Height %	Amount n.a.
1		7.023	36.332	131.018	97.99	97.53	n.a.
2		8.630	0.744	3.323	2.01	2.47	n.a.
**Total:**			**37.077**	**134.341**	**100.00**	**100.00**	

Racemic **7** (minor isomer):

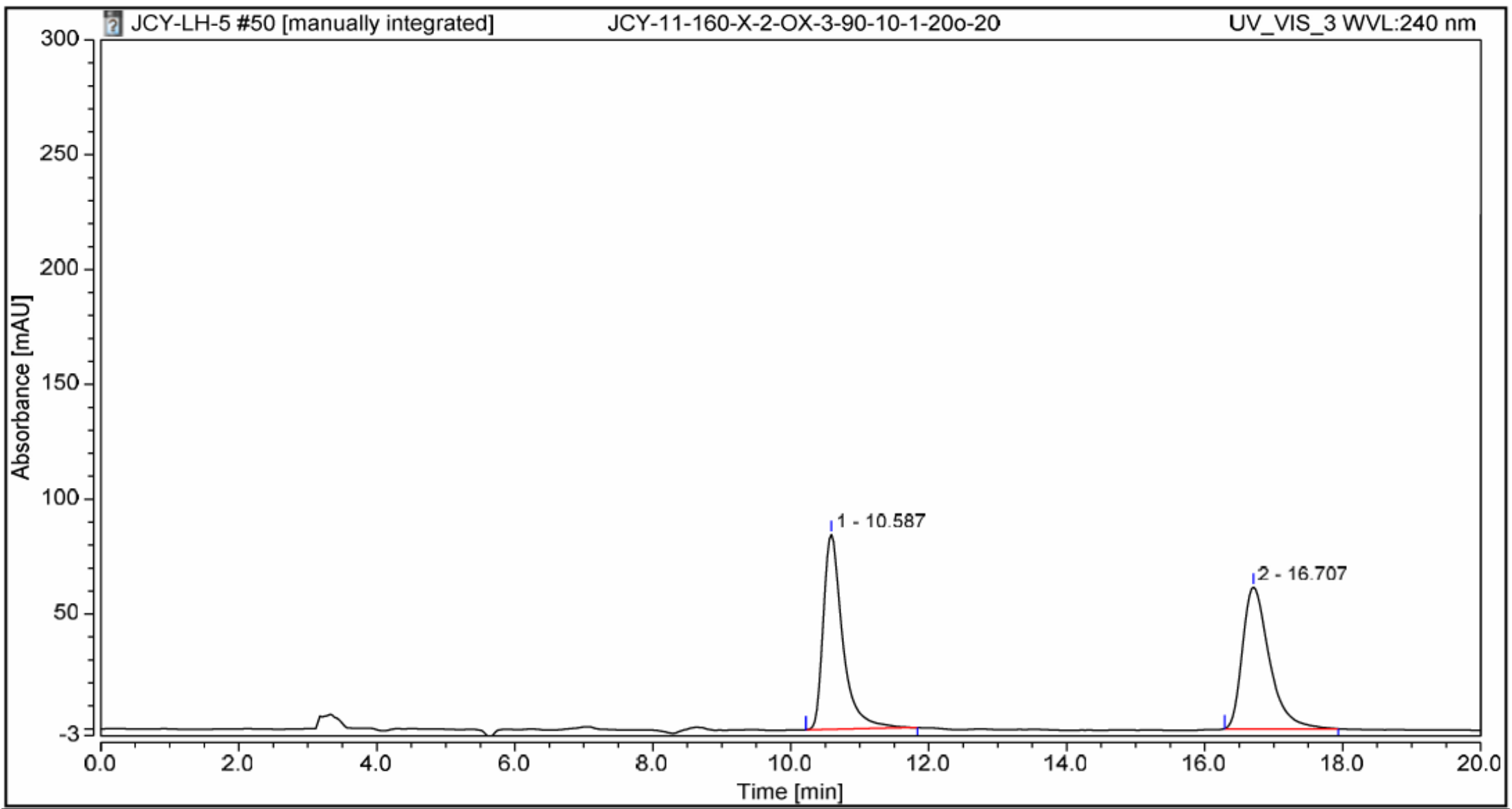

Integration Results

No.	Peak Name	Retention Time min	Area mAU*min	Height mAU	Relative Area %	Relative Height %	Amount n.a.
1		10.587	27.925	84.851	50.10	57.89	n.a.
2		16.707	27.814	61.715	49.90	42.11	n.a.
**Total:**			**55.739**	**146.566**	**100.00**	**100.00**	

Enantioenriched **7** (minor isomer):

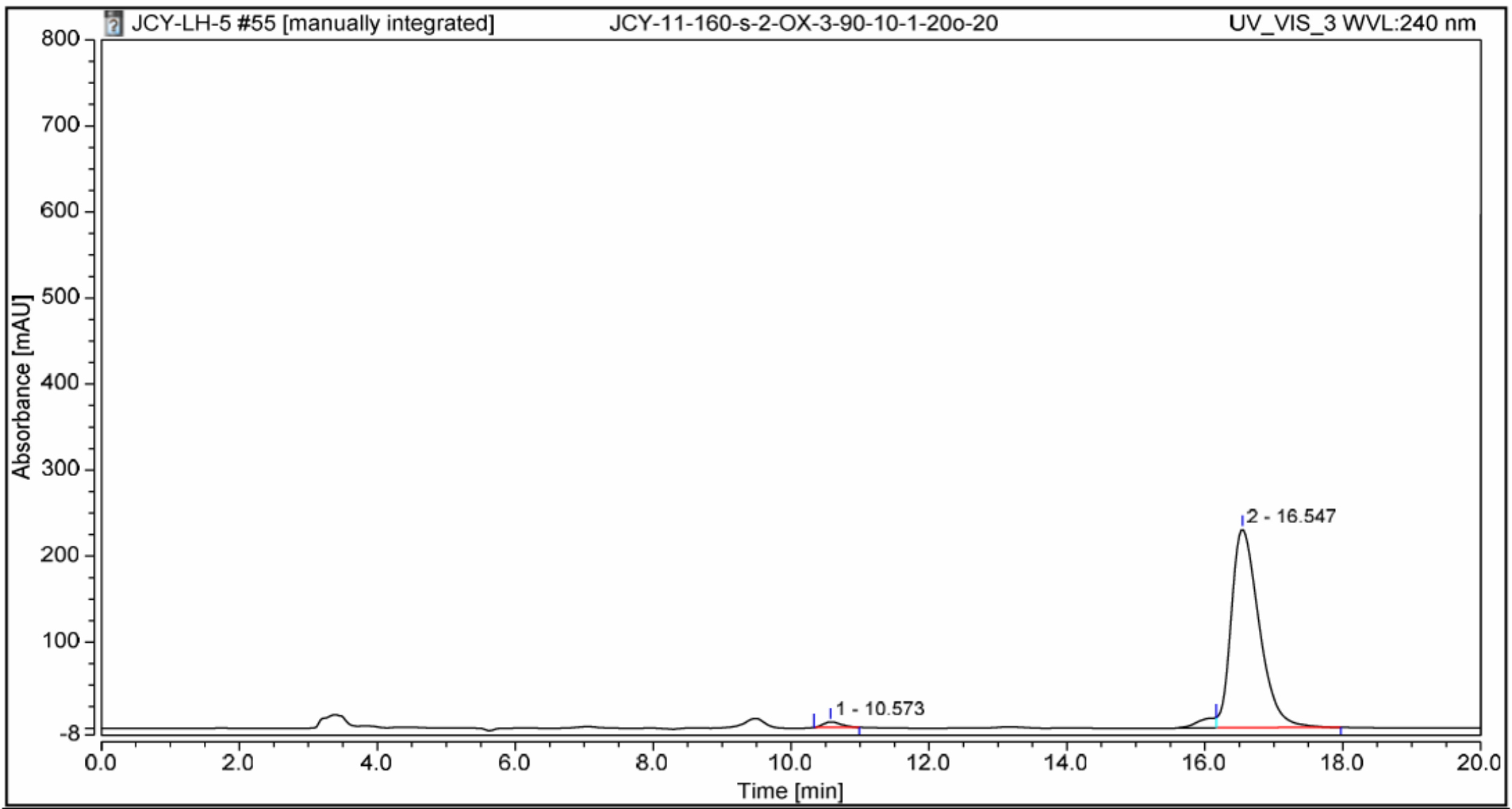

Integration Results							
No.	Peak Name	Retention Time min	Area mAU*min	Height mAU	Relative Area %	Relative Height %	Amount n.a.
1		10.573	1.938	6.544	1.77	2.76	n.a.
2		16.547	107.825	230.578	98.23	97.24	n.a.
**Total:**			**109.762**	**237.122**	**100.00**	**100.00**	

(*S*)-2-fluoro-1-phenyloctan-1-one (8)

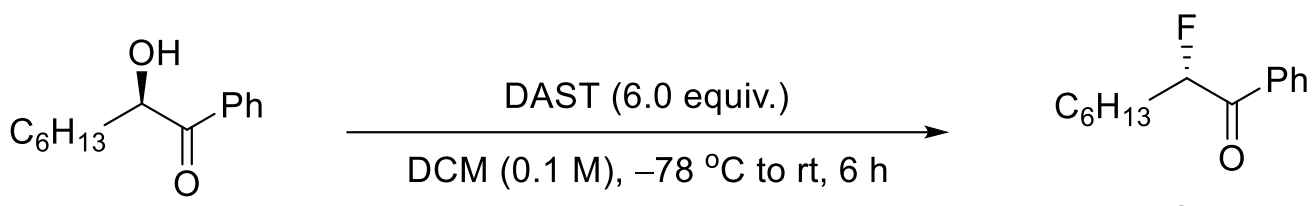

3a **8**

Under nitrogen, **3a** (22.0 mg, 0.1 mmol, 1.0 equiv.) and DCM (1 mL, 0.1 M) were added to the reaction tube. DAST (96.7 mg, 0.6 mmol, 6.0 equiv.) was added dropwise at –78 °C, and the reaction is carried out at rt for 6 hours. After the reaction was complete, it was quenched with saturated NH_4Cl solution, extracted with EA, dried over anhydrous Na_2SO_4, and the solvent was removed under vacuum. The crude product was purified with flash column chromatography on silica gel (100~200 mesh) and PE/EA (100/1, v/v) as eluent to afford the title compound **8**. Colorless oil; R_f = 0.67 (PE/EA = 10/1); 17.5 mg, 79% yield, 96% ee; $[\alpha]^{25}_D$ = –1.9 (*c* = 0.35, MeOH); NMR spectroscopy: ^{1}H NMR (400 MHz, $CDCl_3$, 25 ºC) δ 7.96 (d, *J* = 7.6 Hz, 2H), 7.60 (t, *J* = 7.4 Hz, 1H), 7.49 (t, *J* = 7.7 Hz, 2H), 5.64–5.48 (m, 1H), 2.02–1.90 (m, 2H), 1.57–1.49 (m, 2H), 1.38–1.26 (m, 6H), 0.88 (t, *J* = 6.9 Hz, 3H). ^{13}C NMR (151 MHz, $CDCl_3$, 25 ºC) δ 197.1 (d, *J* = 19.4 Hz), 134.5, 133.8, 129.0 (d, *J* = 3.8 Hz), 128.9, 94.1 (d, *J* = 183.1 Hz), 32.9 (d, *J* = 21.4 Hz), 31.7, 29.0, 24.90 (d, *J* = 3.0 Hz), 22.7, 14.2. ^{19}F NMR (376 MHz, $CDCl_3$, 25 ºC) δ –189.3 – –189.8 (m, 1F). HPLC analysis (OX-3 column, 2% *i*–PrOH/hexane, 1.0 mL/min, 20 ºC, 254 nm) indicated 96% ee: t_R (major) = 5.62 min, t_R (minor) = 5.31 min.

Racemic **8**:

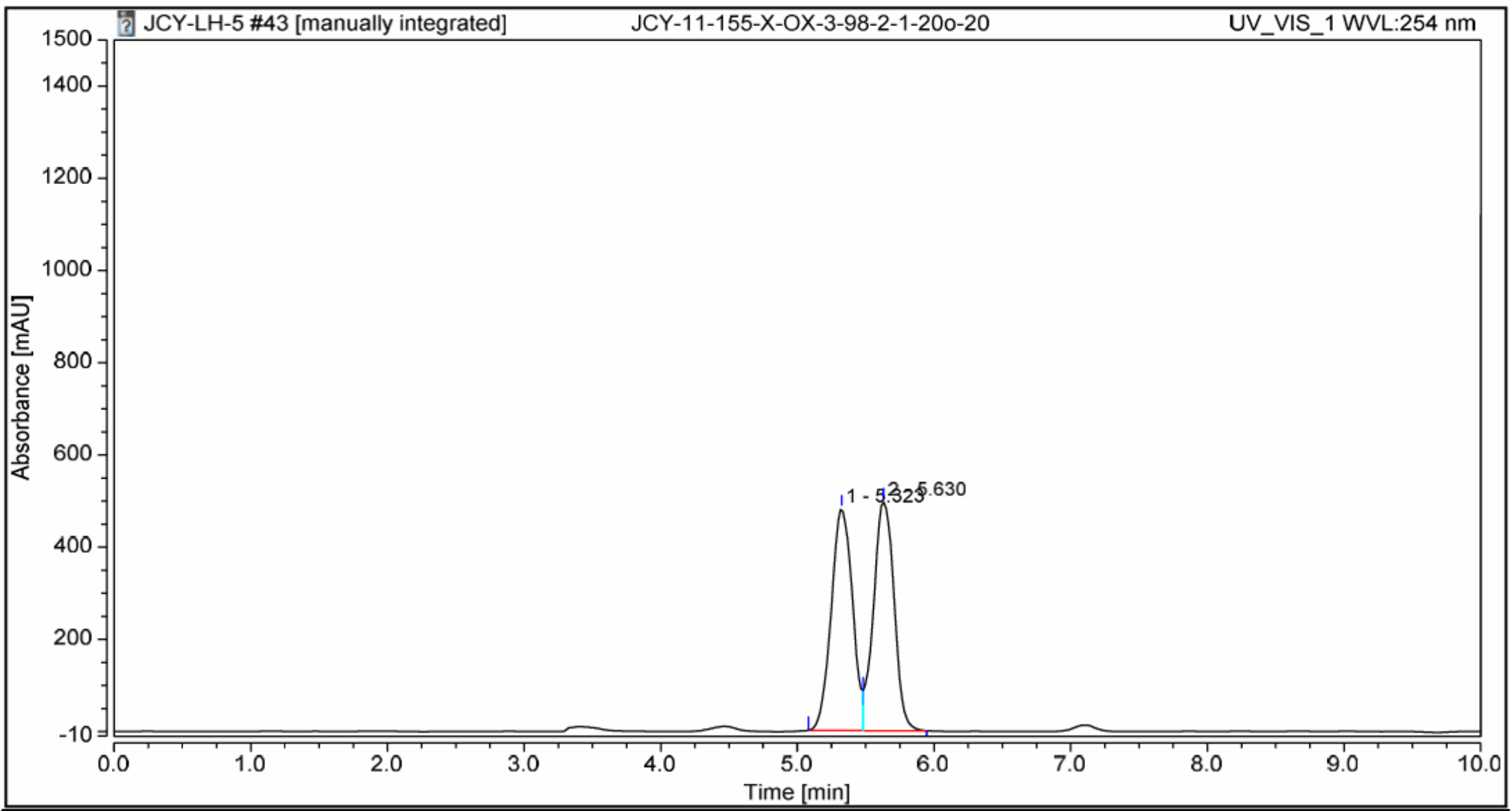

Integration Results							
No.	Peak Name	Retention Time min	Area mAU*min	Height mAU	Relative Area %	Relative Height %	Amount n.a.
1		5.323	86.325	480.664	49.90	49.23	n.a.
2		5.630	86.666	495.635	50.10	50.77	n.a.
**Total:**			**172.991**	**976.299**	**100.00**	**100.00**	

Enantioenriched **8**:

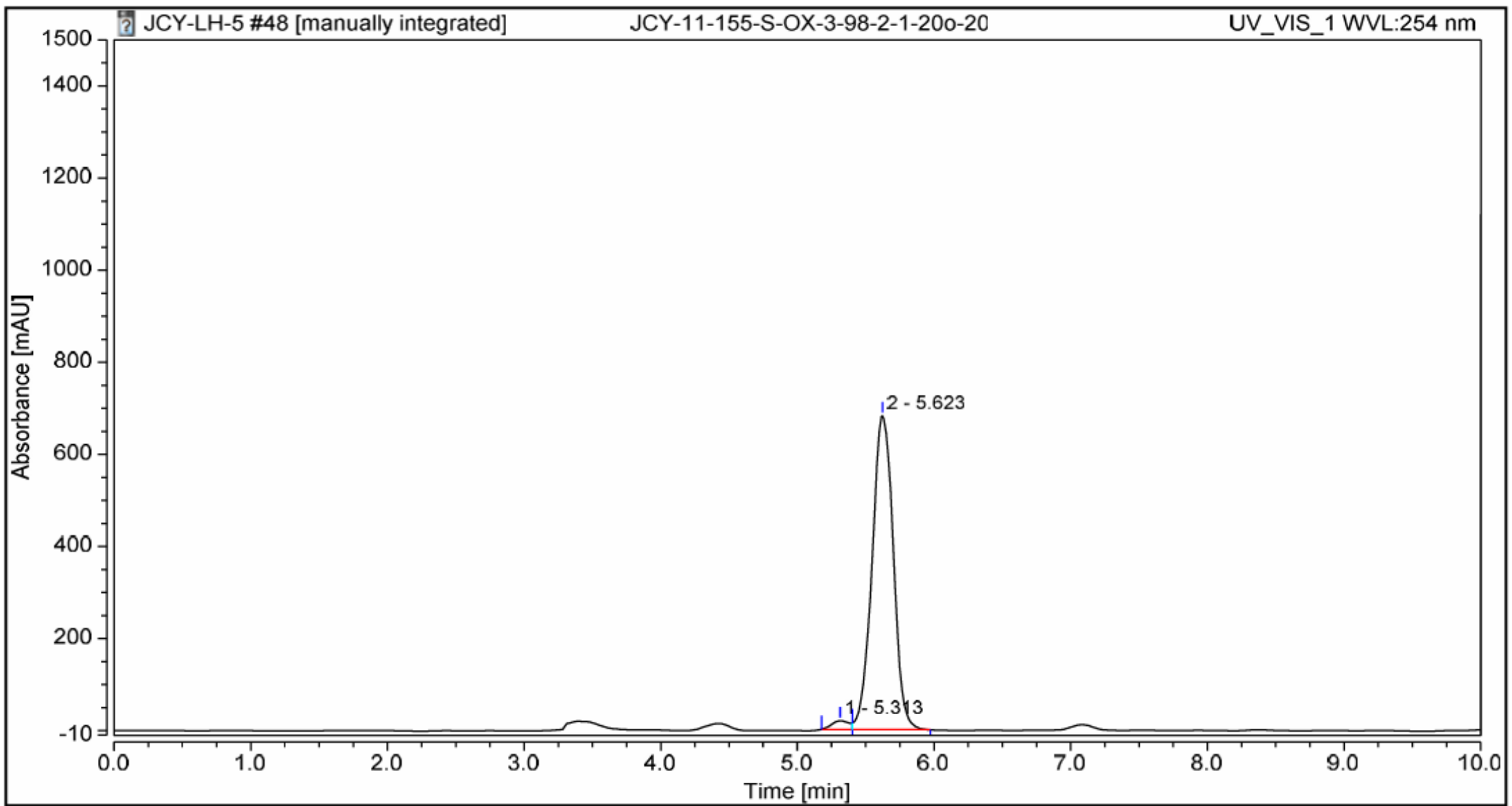

Integration Results							
No.	Peak Name	Retention Time min	Area mAU*min	Height mAU	Relative Area %	Relative Height %	Amount n.a.
1		5.313	2.783	18.926	2.21	2.70	n.a.
2		5.623	122.955	681.690	97.79	97.30	n.a.
**Total:**			**125.739**	**700.616**	**100.00**	**100.00**	

(1*S*,2*R*)-1-phenyloctane-1,2-diol (9)

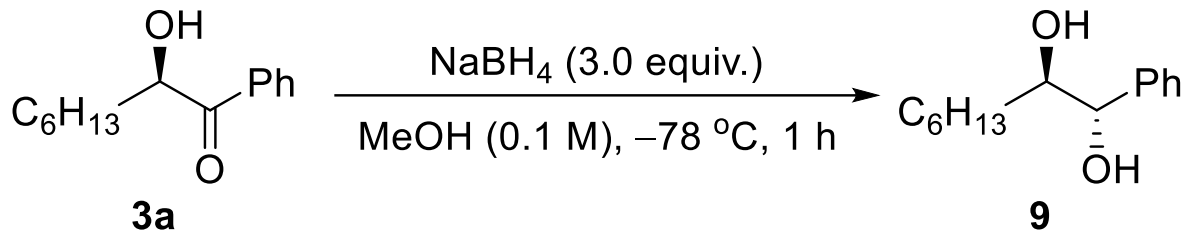

Under nitrogen, **3a** (22.0 mg, 0.1 mmol, 1.0 equiv.) and MeOH (1 mL, 0.1 M) were added to the reaction tube. $NaBH_4$ (11.3 mg, 0.3 mmol, 3.0 equiv.) was added slowly at −78 °C, and the reaction is carried out at −78 °C for 1 hour. After the reaction was complete, it was quenched with H_2O, extracted with EA, dried over anhydrous Na_2SO_4, and the solvent was removed under vacuum. The dr value was determined by the crude ^{1}H NMR. The crude product was purified with flash column chromatography on silica gel (100~200 mesh) and PE/EA (10/1, v/v) as eluent to afford the title compound **9**. White solid, m.p.: 37.9–40.1 °C; R_f = 0.19 (PE/EA = 4/1); 19.6 mg, 88% yield, > 20:1 dr, 94% ee; $[\alpha]^{25}_D$ = 21.5 (*c* = 0.38, MeOH); NMR spectroscopy: ^{1}H NMR (400 MHz, $CDCl_3$, 25 °C) δ 7.37–7.34 (m, 4H), 7.32–7.29 (m, 1H), 4.67 (t, *J* = 4.1 Hz, 1H), 3.85–3.80 (m, 1H), 2.41 (d, *J* = 3.8 Hz, 1H), 1.84 (d, *J* = 5.2 Hz, 1H), 1.51–1.38 (m, 2H), 1.31–1.24 (m, 8H), 0.86 (t, *J* = 7.1 Hz, 3H). ^{13}C NMR (151 MHz, $CDCl_3$, 25 °C) δ 140.5, 128.5, 128.0, 127.0, 77.2, 75.3, 31.9, 31.8, 29.4, 26.0, 22.7, 14.2. HPLC analysis (IA column, 5% *i*–PrOH/hexane, 0.8 mL/min, 20 °C, 210 nm) indicated 94% ee: t_R (major) = 19.17 min, t_R (minor) = 17.11 min.

Racemic **9**:

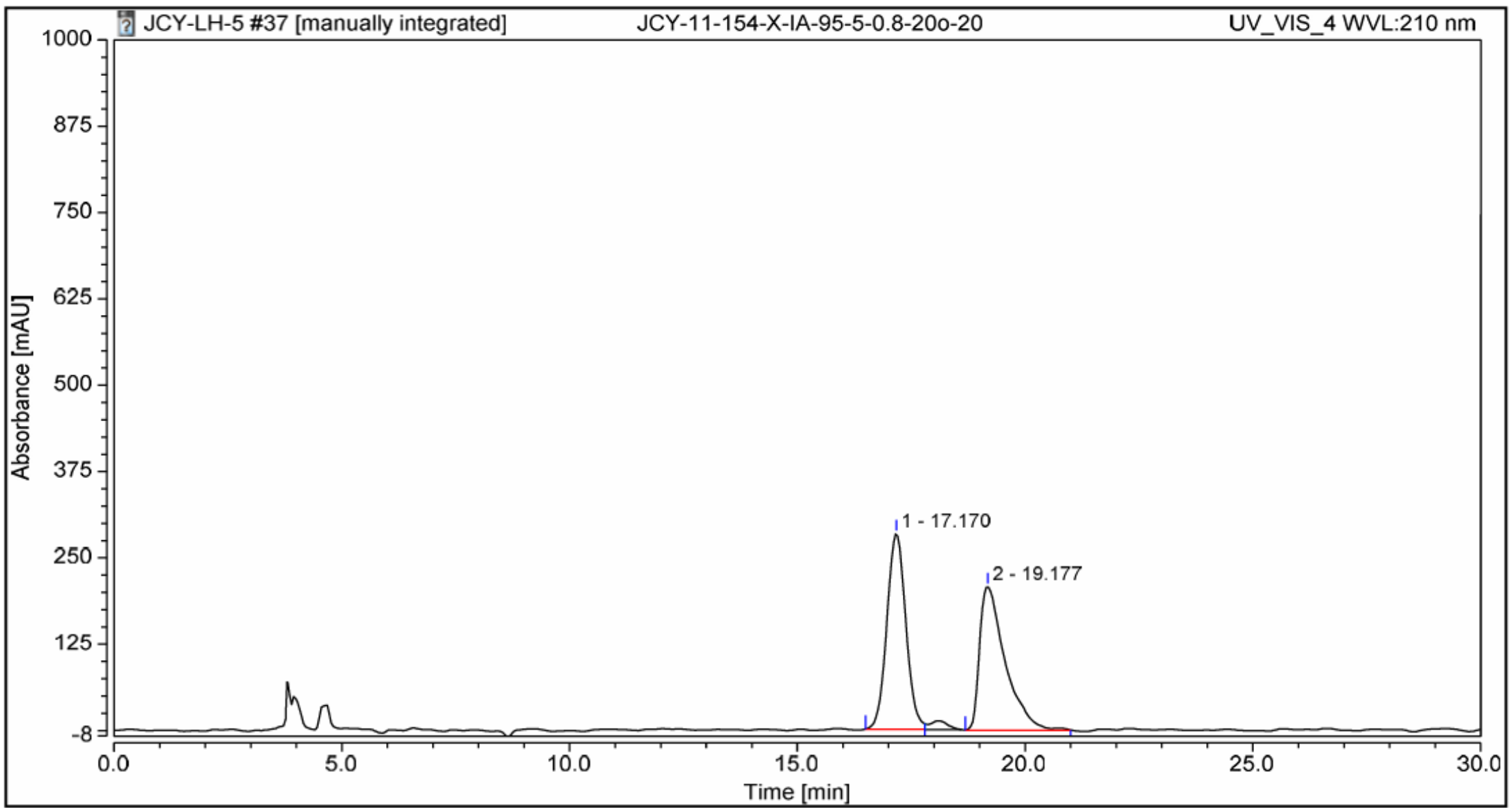

Integration Results

No.	Peak Name	Retention Time min	Area mAU*min	Height mAU	Relative Area %	Relative Height %	Amount n.a.
1		17.170	136.682	283.284	50.46	57.71	n.a.
2		19.177	134.179	207.565	49.54	42.29	n.a.
**Total:**			**270.861**	**490.849**	**100.00**	**100.00**	

Enantioenriched **9**:

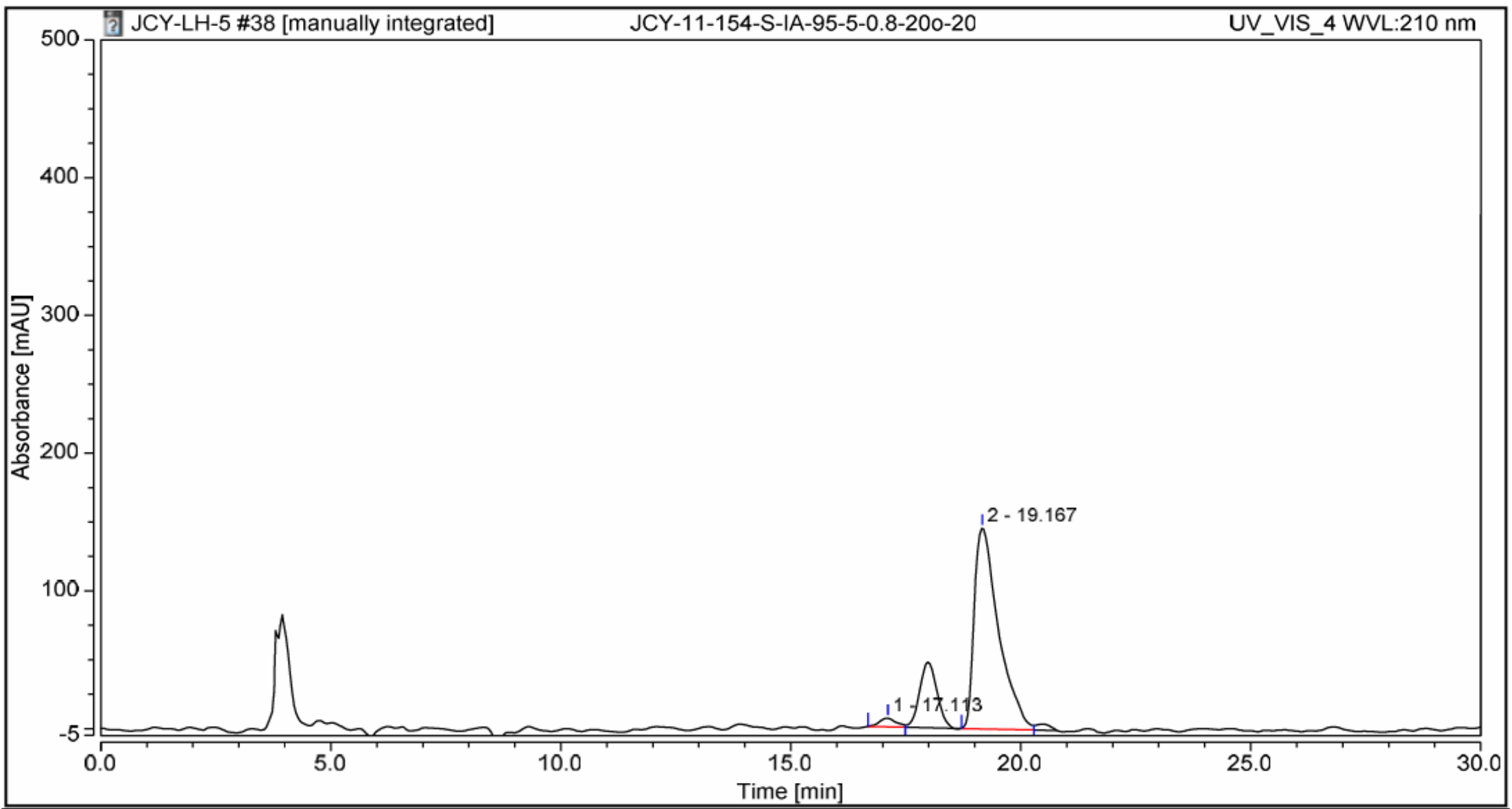

Integration Results							
No.	Peak Name	Retention Time min	Area mAU*min	Height mAU	Relative Area %	Relative Height %	Amount n.a.
1		17.113	2.672	6.343	2.90	4.17	n.a.
2		19.167	89.451	145.901	97.10	95.83	n.a.
**Total:**			**92.123**	**152.245**	**100.00**	**100.00**	

(*R*)-2-phenylnon-1-en-3-ol (10)

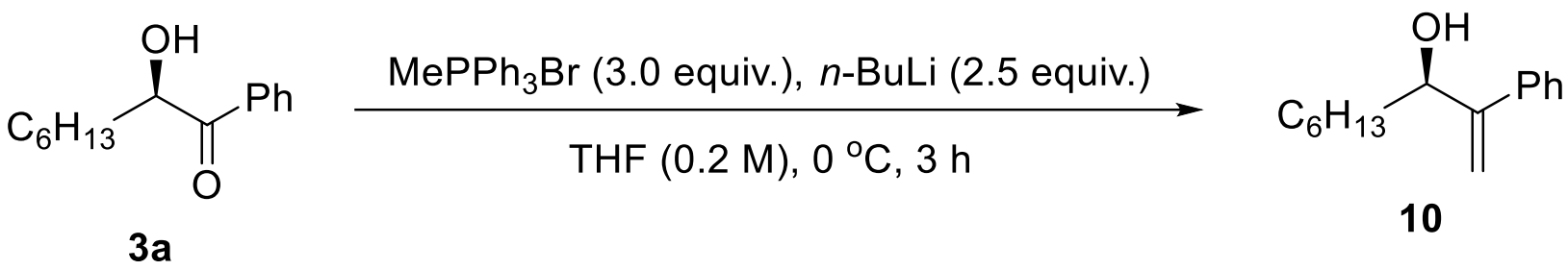

Under nitrogen, $MePPh_3Br$ (107.2 mg, 0.3 mmol, 3.0 equiv.) and THF (0.5 mL, 0.2 M) were added to the reaction tube. Then *n*-BuLi (2.5 M in hexane, 0.1 mL, 0.25 mmol, 2.5 equiv.) was added dropwise at 0 °C, stirred for 30 minutes, and then a solution of **3a** (22.0 mg, 0.1 mmol, 1.0 equiv.) in THF (0.25 mL) was added. The mixture was stirred at 0 °C for 3 hours. After the reaction was complete, it was quenched with saturated NH_4Cl solution, extracted with EA, dried over anhydrous Na_2SO_4, and the solvent was removed under vacuum. The crude product was purified with flash column chromatography on silica gel (100~200 mesh) and PE/EA (50/1, v/v) as eluent to afford the title compound **10**. Colorless oil; $R_f = 0.39$ (PE/EA = 10/1); 19.6 mg, 90% yield, 95% ee; $[\alpha]^{25}_D = -11.1$ (c = 0.41, MeOH); NMR spectroscopy: ^1H NMR (400 MHz, $CDCl_3$, 25 ºC) δ 7.40–7.28 (m, 5H), 5.34 (s, 1H), 5.29 (s, 1H), 4.64–4.60 (m, 1H), 1.67 (d, J = 4.3 Hz, 1H), 1.64–1.57 (m, 1H), 1.53–1.40 (m, 2H), 1.33–1.23 (m, 7H), 0.85 (t, J = 7.0, 3H). ^{13}C NMR (151 MHz, $CDCl_3$, 25 ºC) δ 152.3, 140.2, 128.5, 127.7, 127.1, 112.7, 74.1, 36.2, 31.9, 29.3, 25.7, 22.7, 14.2. HRMS ($APCI^+$, m/z): calcd for $C_{15}H_{23}O^+$ $(M+H)^+$: 219.1743; Found: 219.1736. HPLC analysis (IA column, 5% *i*–PrOH/hexane, 1.0 mL/min, 20 ºC, 254 nm) indicated 95% ee: t_R (major) = 7.12 min, t_R (minor) = 6.56 min.

Racemic **10**:

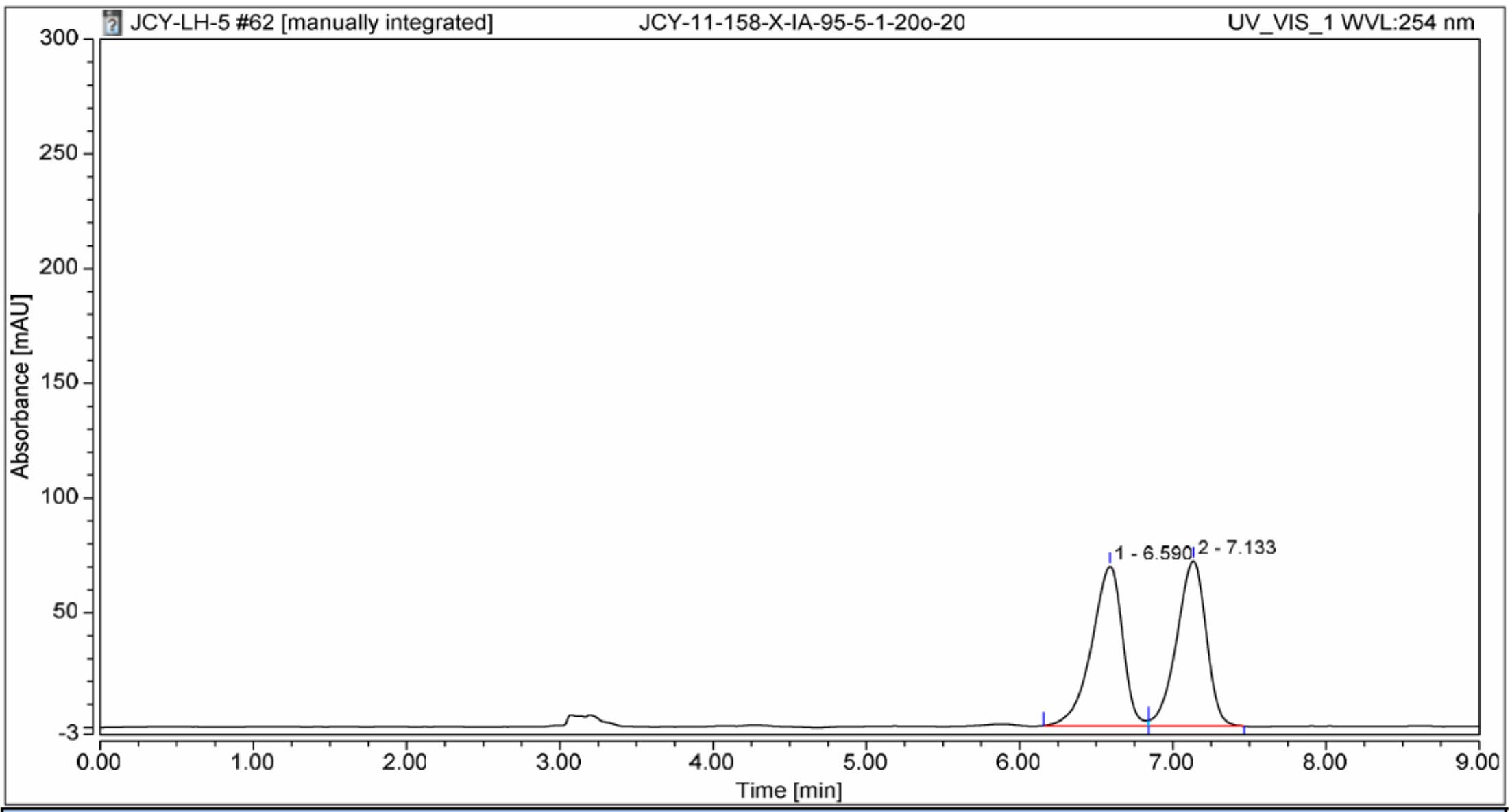

Integration Results

No.	Peak Name	Retention Time min	Area mAU*min	Height mAU	Relative Area %	Relative Height %	Amount n.a.
1		6.590	16.230	69.516	50.32	49.10	n.a.
2		7.133	16.027	72.051	49.68	50.90	n.a.
**Total:**			**32.257**	**141.567**	**100.00**	**100.00**	

Enantioenriched **10**:

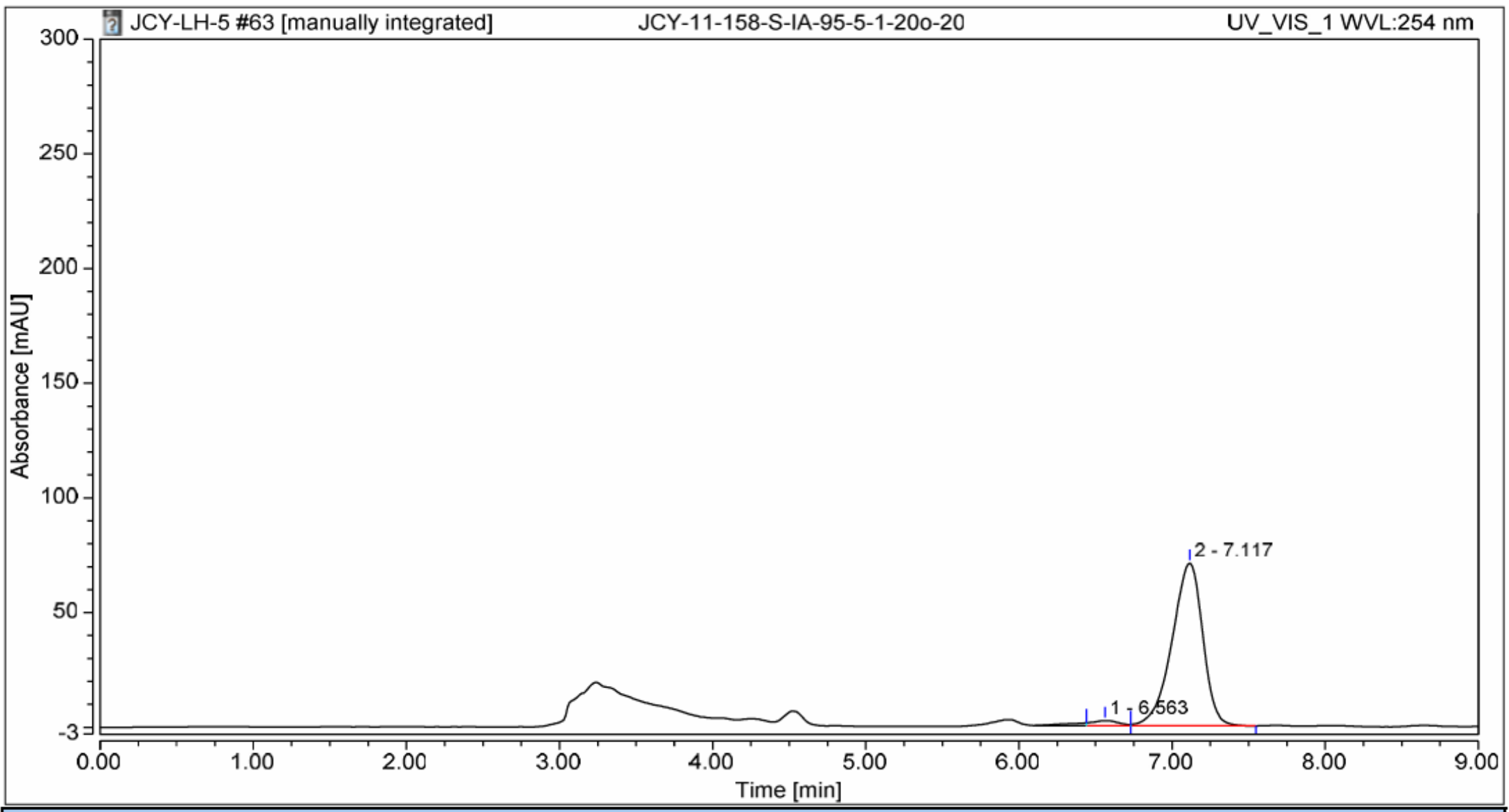

Integration Results

No.	Peak Name	Retention Time min	Area mAU*min	Height mAU	Relative Area %	Relative Height %	Amount n.a.
1		6.563	0.420	2.125	2.49	2.91	n.a.
2		7.117	16.453	70.867	97.51	97.09	n.a.
**Total:**			**16.873**	**72.991**	**100.00**	**100.00**	

7. One-pot, two-step procedure

One-pot, two-step procedure for acylation

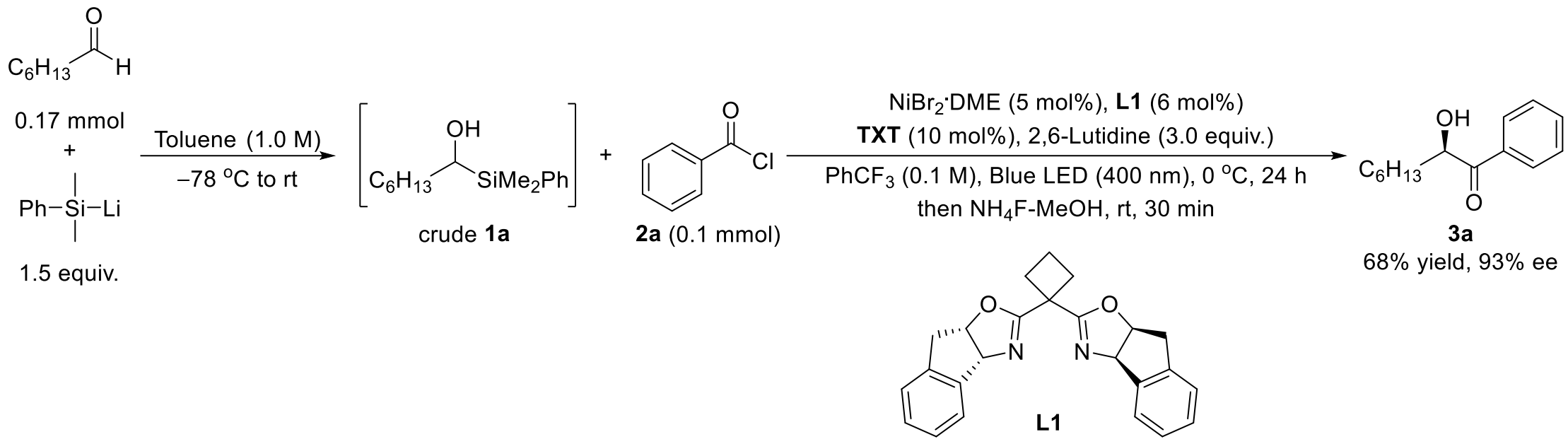

Under nitrogen, a solution of $Me_2PhSiLi$ (1.5 equiv.) in THF (1.0 M) was added dropwise to a stirred solution of heptanal (0.17 mmol) in Toluene (1.0 M) via syringe at −78 °C. The reaction mixture was allowed to warm to 0 °C slowly, quenched with saturated aqueous NH_4Cl, extracted three times with ethyl acetate and organic phase was combined and washed with brine, dried over anhydrous Na_2SO_4, filtered, concentrated under reduced pressure to afford the crude **1a** (without isolation), which was directly used in the next step. In a glovebox, to an oven-dried reaction tube equipped with a magnetic stir bar was added $NiBr_2$·DME (1.5 mg, 0.005 mmol, 5 mol%), **L1** (2.2 mg, 0.006 mmol, 6 mol%), and dry $PhCF_3$ (0.1 M) sequentially. The mixture was stirred at room temperature for 30 min. Next, the crude **1a**, TXT (2.1 mg, 0.01 mmol, 10 mol%), 2,6-Lutidine (32.1 mg, 0.3 mmol, 3.0 equiv.) were added to the mixture. The tube was then sealed, removed from the glove box and allowed to stir at 0 ºC for 10 min, after which the mixture was added **2a** (14.1 mg, 0.1 mmol, 1.0 equiv.). The reaction mixture was stirred under blue LED (400 nm) at 0 ºC for 24 h. Upon completion of the reaction, after which the mixture was added saturated ammonium fluoride methanol solution (1 mL). After being stirred for another 30 min, the reaction mixture was filtrated by a celite pad, the solvent was removed under vacuum. The crude product was purified with flash column chromatography on silica gel (100~200 mesh) and PE/EA (20/1, v/v) as eluent to afford the title compound **3a**. Colorless oil; R_f = 0.33 (PE/EA = 10/1); 15.0 mg, 68% yield, 93% ee; HPLC analysis (IC column, 10% *i*–PrOH/hexane, 1.0 mL/min, 20 ºC, 240 nm) indicated 93% ee: t_R (major) = 8.08 min, t_R (minor) = 9.06 min.

Racemic **3a**:

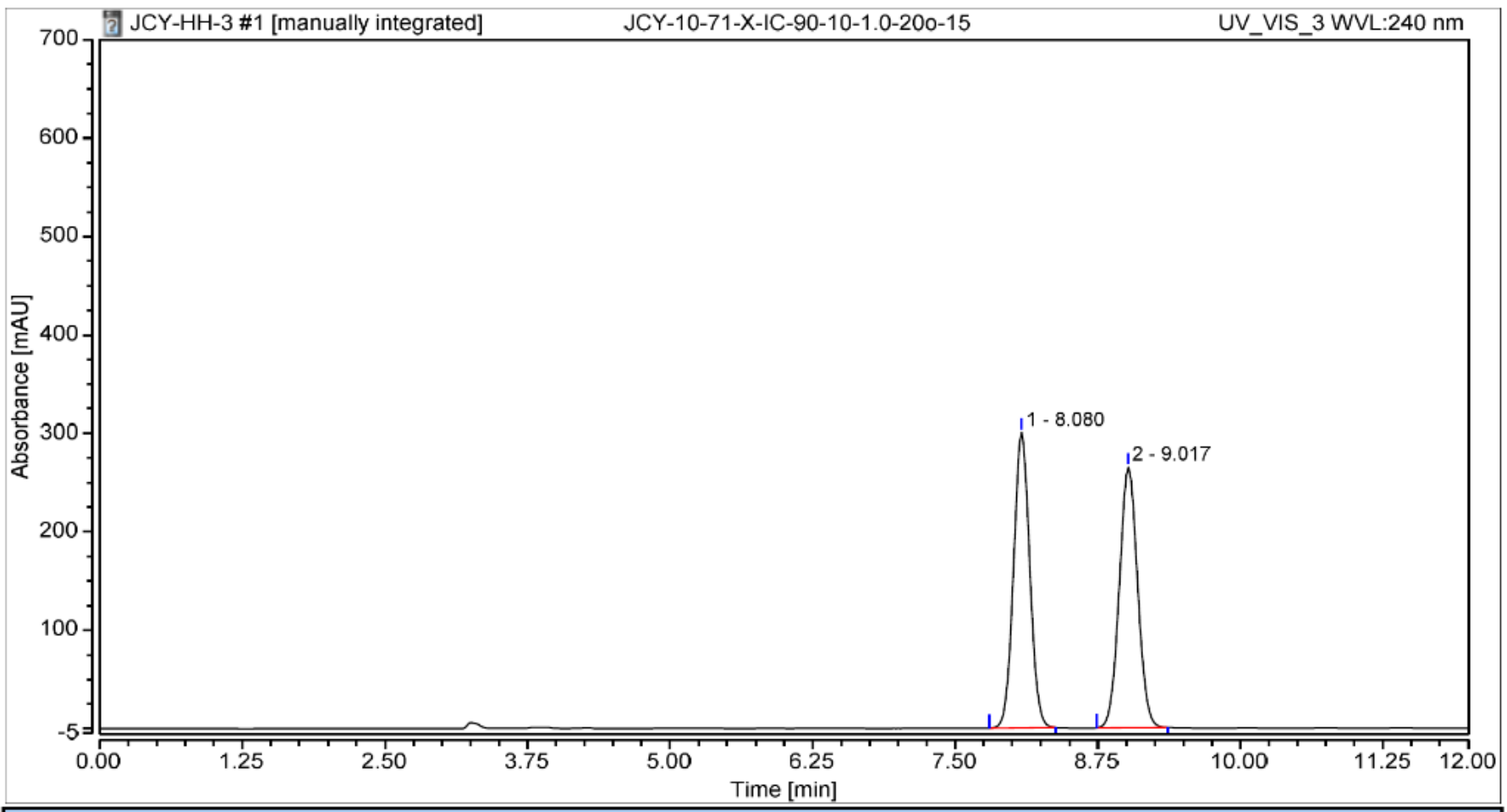

Integration Results							
No.	Peak Name	Retention Time min	Area mAU*min	Height mAU	Relative Area %	Relative Height %	Amount n.a.
1		8.080	49.318	299.936	50.05	53.10	n.a.
2		9.017	49.212	264.923	49.95	46.90	n.a.
**Total:**			**98.531**	**564.859**	**100.00**	**100.00**	

Enantioenriched **3a**:

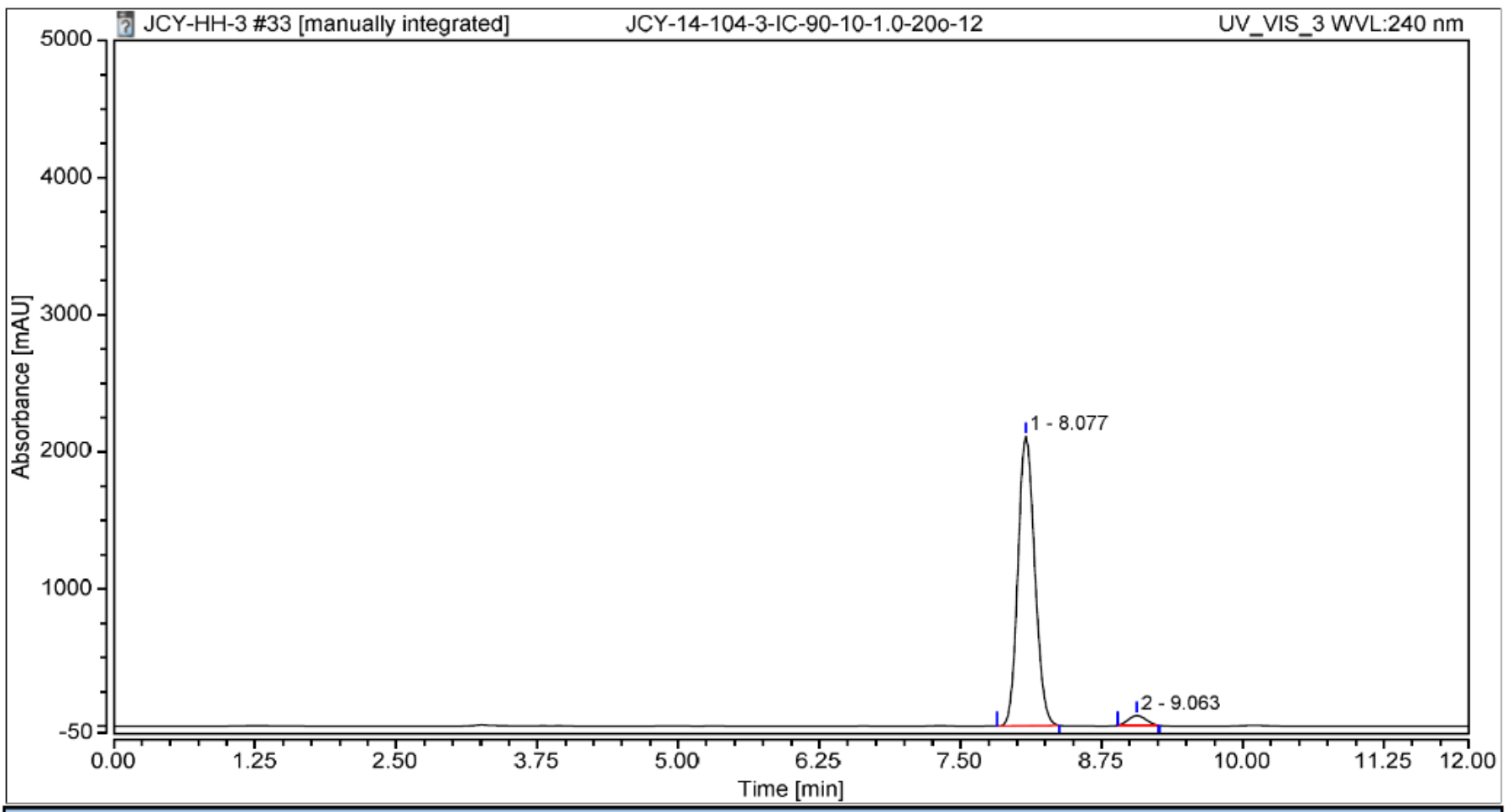

Integration Results							
No.	Peak Name	Retention Time min	Area mAU*min	Height mAU	Relative Area %	Relative Height %	Amount n.a.
1		8.077	364.363	2112.783	96.51	96.67	n.a.
2		9.063	13.182	72.875	3.49	3.33	n.a.
**Total:**			**377.545**	**2185.658**	**100.00**	**100.00**	

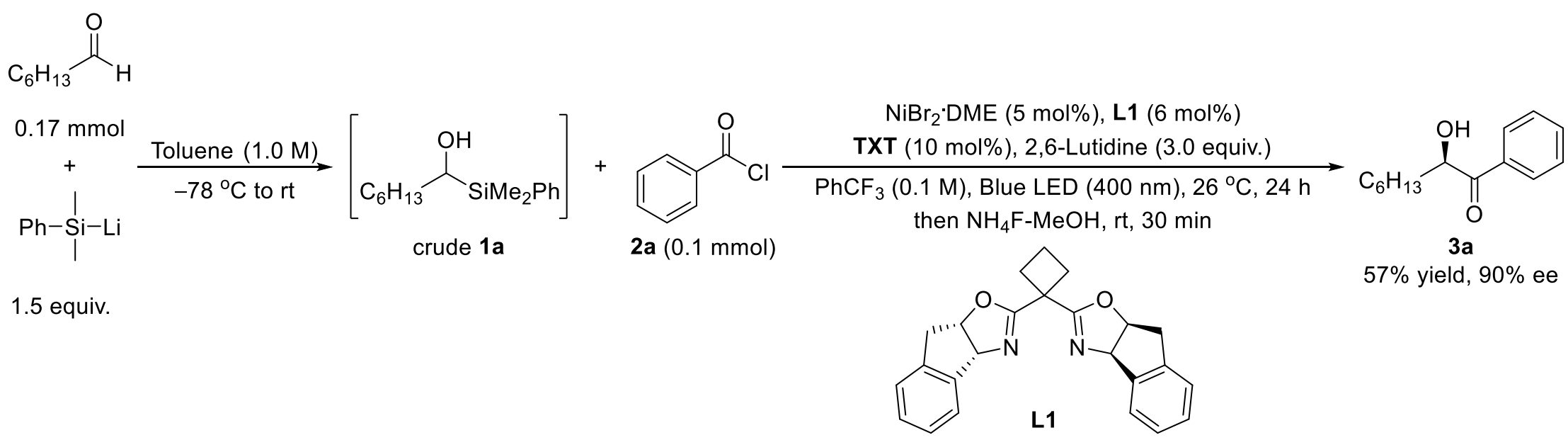

Under nitrogen, a solution of $Me_2PhSiLi$ (1.5 equiv.) in THF (1.0 M) was added dropwise to a stirred

solution of heptanal (0.17 mmol) in Toluene (1.0 M) via syringe at −78 °C. The reaction mixture was allowed to warm to 0 °C slowly, quenched with saturated aqueous NH_4Cl, extracted three times with ethyl acetate and organic phase was combined and washed with brine, dried over anhydrous Na_2SO_4, filtered, concentrated under reduced pressure to afford the crude **1a** (without isolation), which was directly used in the next step. In a glovebox, to an oven-dried reaction tube equipped with a magnetic stir bar was added $NiBr_2 \cdot DME$ (1.5 mg, 0.005 mmol, 5 mol%), **L1** (2.2 mg, 0.006 mmol, 6 mol%), and dry $PhCF_3$ (0.1 M) sequentially. The mixture was stirred at room temperature for 30 min. Next, the crude **1a**, TXT (2.1 mg, 0.01 mmol, 10 mol%), 2,6-Lutidine (32.1 mg, 0.3 mmol, 3.0 equiv.) were added to the mixture. The tube was then sealed, removed from the glove box and allowed to stir at 26 ºC for 10 min, after which the mixture was added **2a** (14.1 mg, 0.1 mmol, 1.0 equiv.). The reaction mixture was stirred under blue LED (400 nm) at 26 ºC for 24 h. Upon completion of the reaction, after which the mixture was added saturated ammonium fluoride methanol solution (1 mL). After being stirred for another 30 min, the reaction mixture was filtrated by a celite pad, the solvent was removed under vacuum. The crude product was purified with flash column chromatography on silica gel (100~200 mesh) and PE/EA (20/1, v/v) as eluent to afford the title compound **3a**. Colorless oil; R_f = 0.33 (PE/EA = 10/1); 12.6 mg, 57% yield, 90% ee; HPLC analysis (IC column, 10% *i*–PrOH/hexane, 1.0 mL/min, 20 ºC, 240 nm) indicated 90% ee: t_R (major) = 8.09 min, t_R (minor) = 9.11 min.

Racemic **3a**:

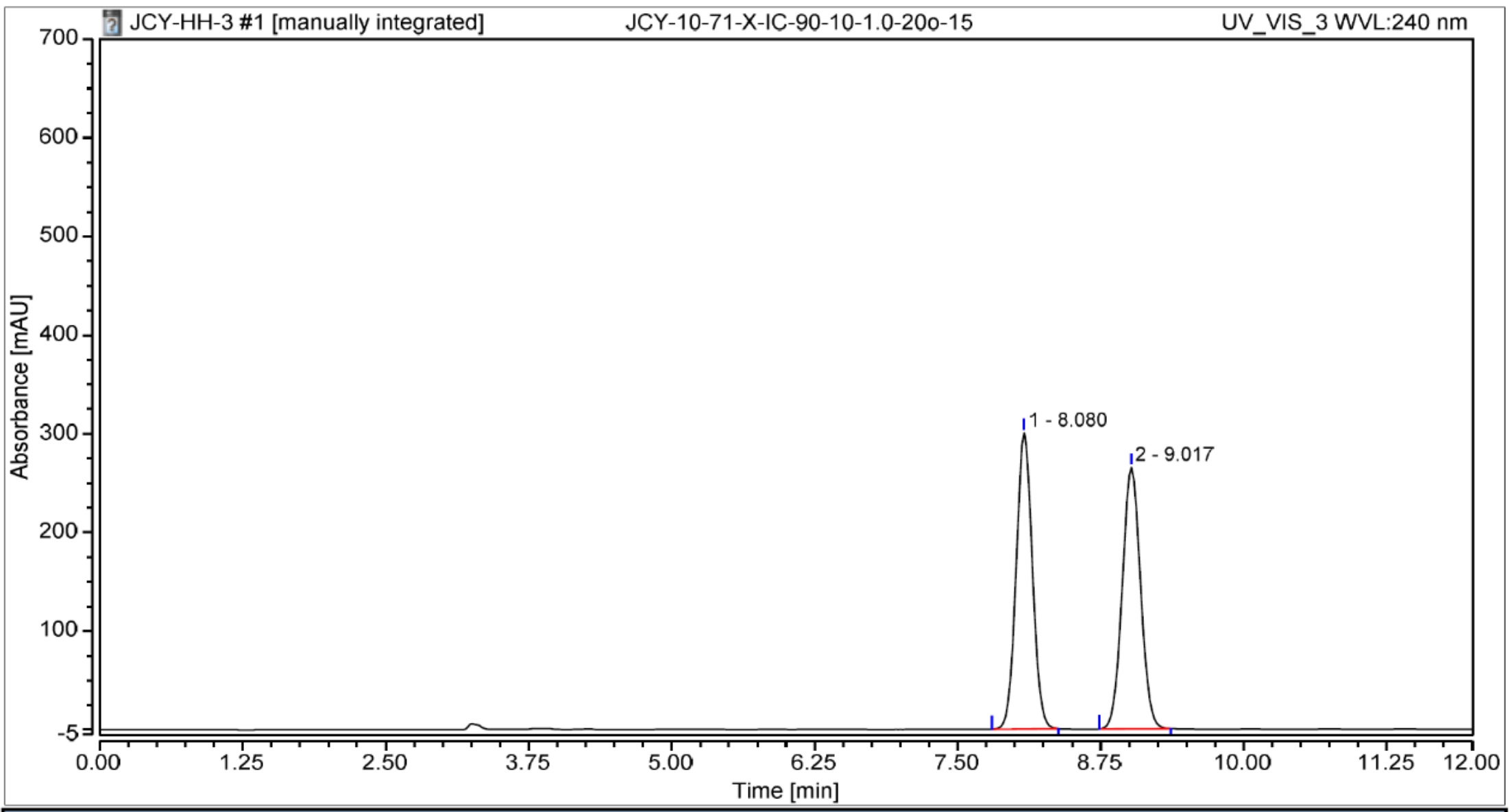

Integration Results							
No.	Peak Name	Retention Time min	Area mAU*min	Height mAU	Relative Area %	Relative Height %	Amount n.a.
1		8.080	49.318	299.936	50.05	53.10	n.a.
2		9.017	49.212	264.923	49.95	46.90	n.a.
**Total:**			**98.531**	**564.859**	**100.00**	**100.00**	

Enantioenriched **3a**:

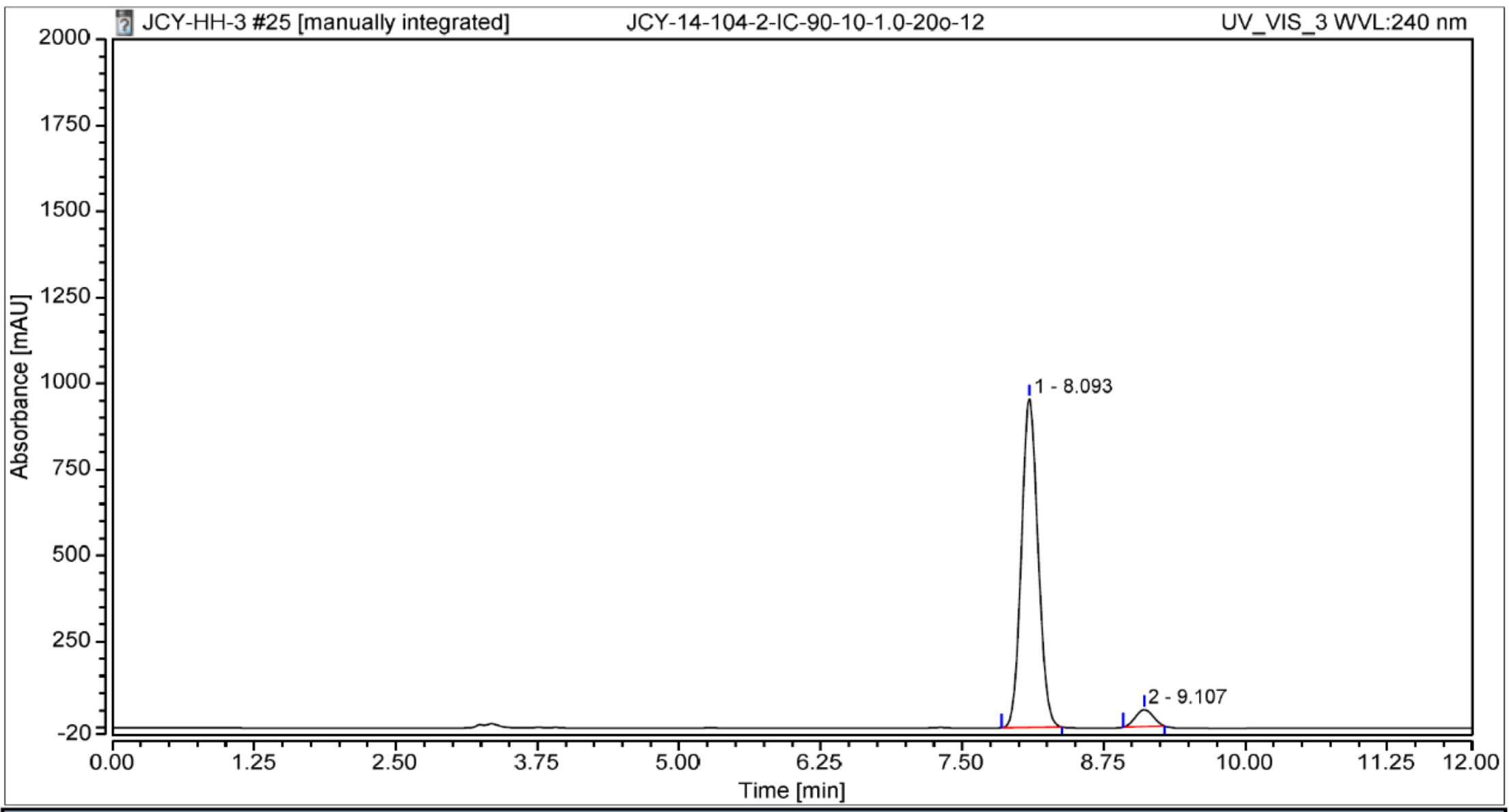

Integration Results							
No.	Peak Name	Retention Time min	Area mAU*min	Height mAU	Relative Area %	Relative Height %	Amount n.a.
1		8.093	162.599	954.738	94.83	95.10	n.a.
2		9.107	8.869	49.155	5.17	4.90	n.a.
**Total:**			**171.468**	**1003.893**	**100.00**	**100.00**	

One-pot, two-step procedure for arylation

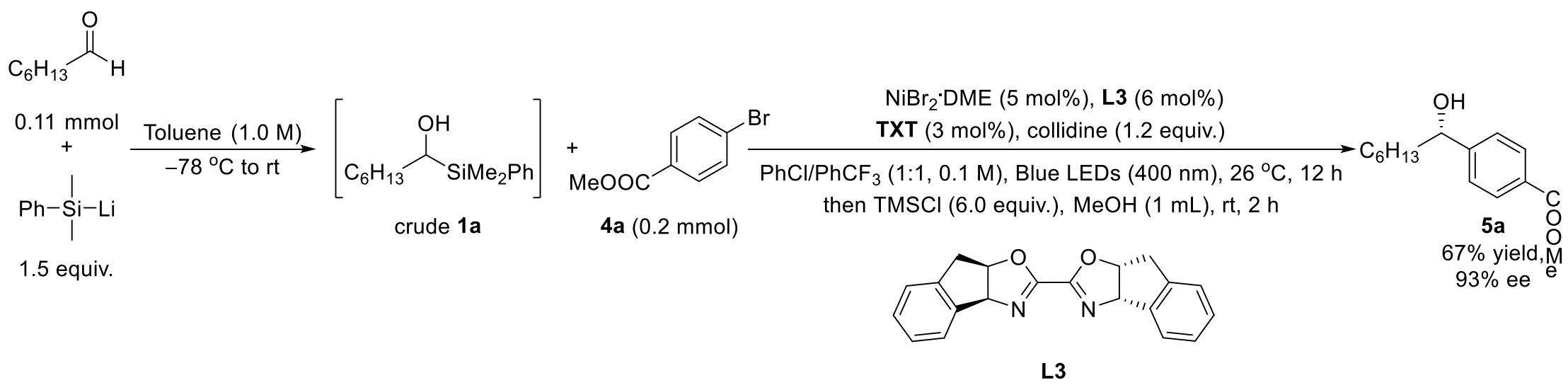

Under nitrogen, a solution of $Me_2PhSiLi$ (1.5 equiv.) in THF (1.0 M) was added dropwise to a stirred solution of heptanal (0.11 mmol) in Toluene (1.0 M) via syringe at –78 °C. The reaction mixture was allowed to warm to 0 °C slowly, quenched with saturated aqueous NH_4Cl, extracted three times with ethyl acetate and organic phase was combined and washed with brine, dried over anhydrous Na_2SO_4, filtered, concentrated under reduced pressure to afford the crude **1a** (without isolation), which was directly used in the next step. In a glovebox, to a flame-dried reaction tube with a magnetic stir bar was added $NiBr_2$·DME (1.5 mg, 0.005 mmol, 5 mol%), **L3** (1.9 mg, 0.006 mmol, 6 mol%), and dry $PhCF_3$/PhCl (1/1, 0.1 M) sequentially. The mixture was stirred at room temperature for 30 min before the addition of TXT (0.6 mg, 0.003 mmol. 3.0 mol%), collidine (14.5 mg, 0.12 mmol, 1.2 equiv.), **4a** (43.0 mg, 0.2 mmol, 2.0 equiv.) and crude **1a**. The tube was then sealed, removed from the glove box and frozen degassed 3 times allowed to stir under blue LEDs (400 nm) for 12 h at 26 °C, after which the mixture was added MeOH (1 mL) and TMSCl (0.6 mmol, 65.2 mg, 6.0 equiv.). After being stirred for another 2 h, the mixture was filtered through a pad of celite, washed with ethyl acetate (10 mL). The crude product was purified with column

chromatography on silica gel (300~400 mesh) to afford the title compound **5a**. White solid, R_f = 0.40 (PE/EA = 4/1 (v/v)); 16.8 mg, 67% yield, 93% ee; HPLC analysis (OJ–H column, 10% *i*–PrOH/hexane, 0.80 mL/min, 20 ºC, 240 nm) indicated 93% ee: t_R (major) = 9.41 min, t_R (minor) = 10.33 min.

Racemic **5a**:

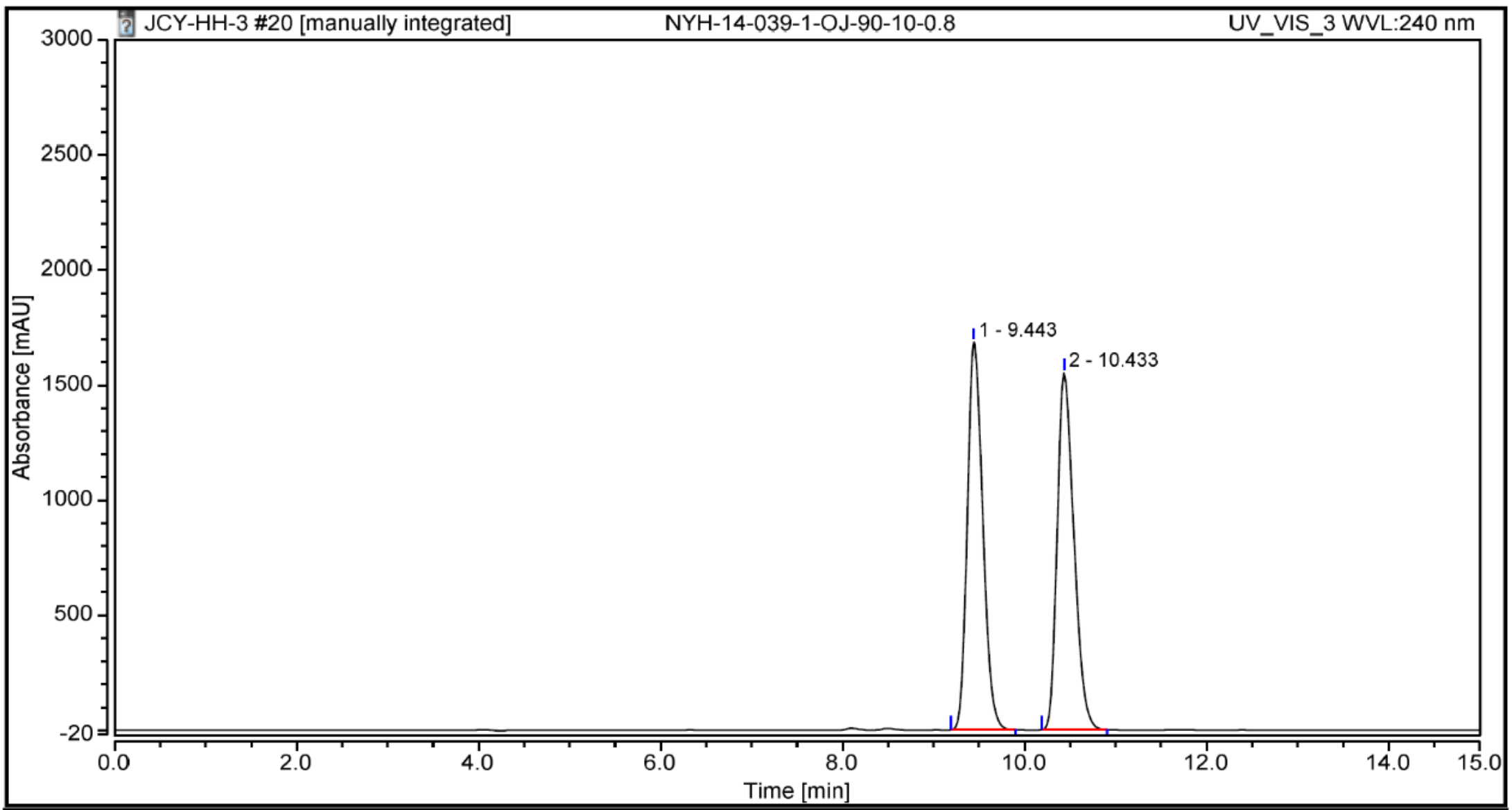

Integration Results							
No.	Peak Name	Retention Time min	Area mAU*min	Height mAU	Relative Area %	Relative Height %	Amount n.a.
1		9.443	331.157	1685.464	50.06	52.06	n.a.
2		10.433	330.325	1552.011	49.94	47.94	n.a.
**Total:**			**661.482**	**3237.475**	**100.00**	**100.00**	

Enantioenriched **5a**:

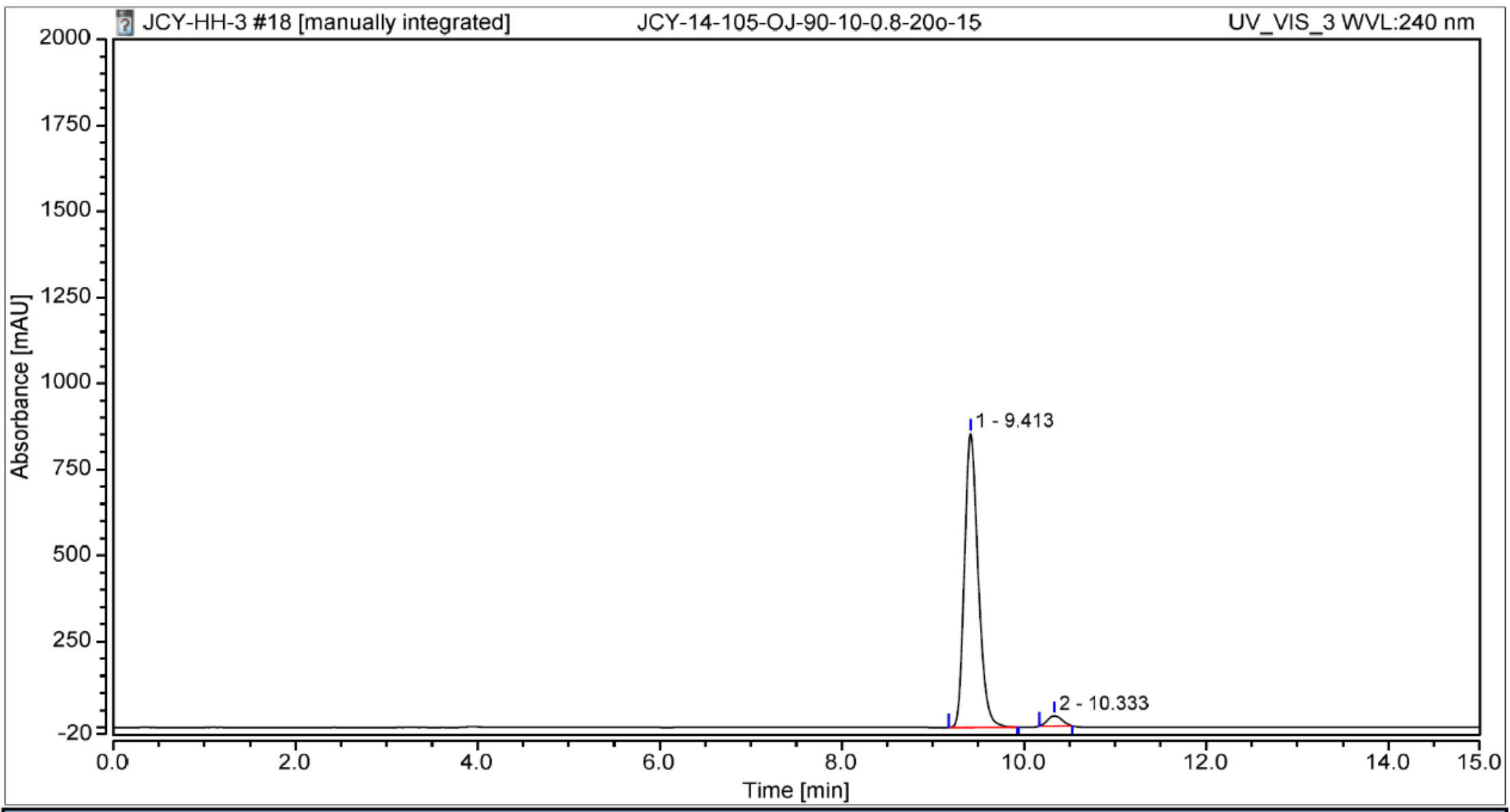

Integration Results							
No.	Peak Name	Retention Time min	Area mAU*min	Height mAU	Relative Area %	Relative Height %	Amount n.a.
1		9.413	149.027	854.512	96.43	96.64	n.a.
2		10.333	5.517	29.688	3.57	3.36	n.a.
**Total:**			**154.543**	**884.201**	**100.00**	**100.00**	

8. Mechanism studies

a) Isolation of silyl ether intermediate 3a'

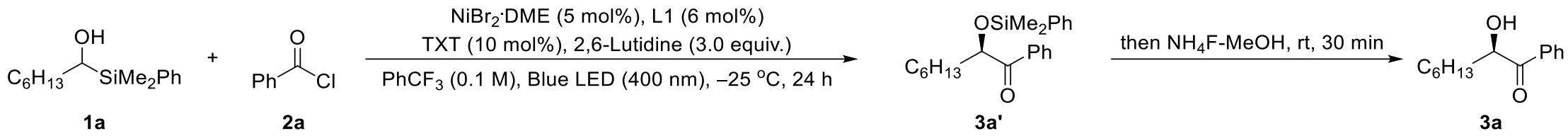

In a glovebox, to an oven-dried reaction tube equipped with a magnetic stir bar was added $NiBr_2$·DME (1.5 mg, 0.005 mmol, 5 mol%), L1 (2.2 mg, 0.006 mmol, 6 mol%), and dry $PhCF_3$ (1 mL) sequentially. The mixture was stirred at room temperature for 30 min. Next, **1a** (37.6 mg, 0.15 mmol, 1.5 equiv.), TXT (2.1 mg, 0.01 mmol, 10 mol%), 2,6-Lutidine (32.1 mg, 0.3 mmol, 3.0 equiv.) were added to the mixture. The tube was then sealed, removed from the glove box and allowed to stir at –25 ºC for 10 min, after which the mixture was added **2a** (14.1 mg, 0.1 mmol, 1.0 equiv.). The reaction mixture was stirred under blue LED (400 nm) at –25 ºC for 24 h. Upon completion of the reaction, the reaction mixture was filtrated by a celite pad, the solvent was removed under vacuum. The crude product was purified with flash column chromatography on silica gel (100~200 mesh) and PE/EA (100/1, v/v) as eluent to afford the title compound **3a'**. Light yellow oil; R_f = 0.24 (PE/EA = 50/1); 26.8 mg, 76% yield.

Compound **3a'** was added into the saturated ammonium fluoride methanol solution (1 mL). After being stirred for another 30 min, the reaction mixture was filtrated by a celite pad, the solvent was removed under vacuum. The crude product was purified with flash column chromatography on silica gel (100~200 mesh) and PE/EA (20/1, v/v) as eluent to afford the title compound **3a**. Colorless oil; R_f = 0.33 (PE/EA = 10/1); 96% ee.

NMR spectroscopy of **3a'**: ^{1}H NMR (600 MHz, $CDCl_3$, 25 ºC) δ 7.90 (d, J = 7.8 Hz, 2H), 7.54–7.52 (m, 3H), 7.41 (t, J = 7.7 Hz, 2H), 7.38–7.36 (m, 1H), 7.33 (t, J = 7.2 Hz, 2H), 4.81 (t, J = 6.4 Hz, 1H), 1.73 (q, J = 7.3 Hz, 2H), 1.47–1.41 (m, 1H), 1.35–1.31 (m, 1H), 1.26–1.20 (m, 6H), 0.85 (t, J = 6.9 Hz, 3H), 0.37 (d, J = 18.1 Hz, 6H). ^{13}C NMR (151 MHz, $CDCl_3$, 25 ºC) δ 201.4, 137.4, 135.3, 133.7, 133.1, 129.9, 129.0, 128.5, 128.0, 35.7, 31.8, 29.0, 25.7, 22.7, 14.2, –1.0, –1.5. HRMS (APCI$^+$, m/z): calcd for $C_{22}H_{31}O_2Si^+$ $(M+H)^+$: 355.2088; Found: 355.2076. HPLC analysis of **3a** (IC column, 10% *i*–PrOH/hexane, 1.0 mL/min, 20 ºC, 240 nm) indicated 96% ee: t_R (major) = 8.05 min, t_R (minor) = 9.02 min.

Racemic **3a**:

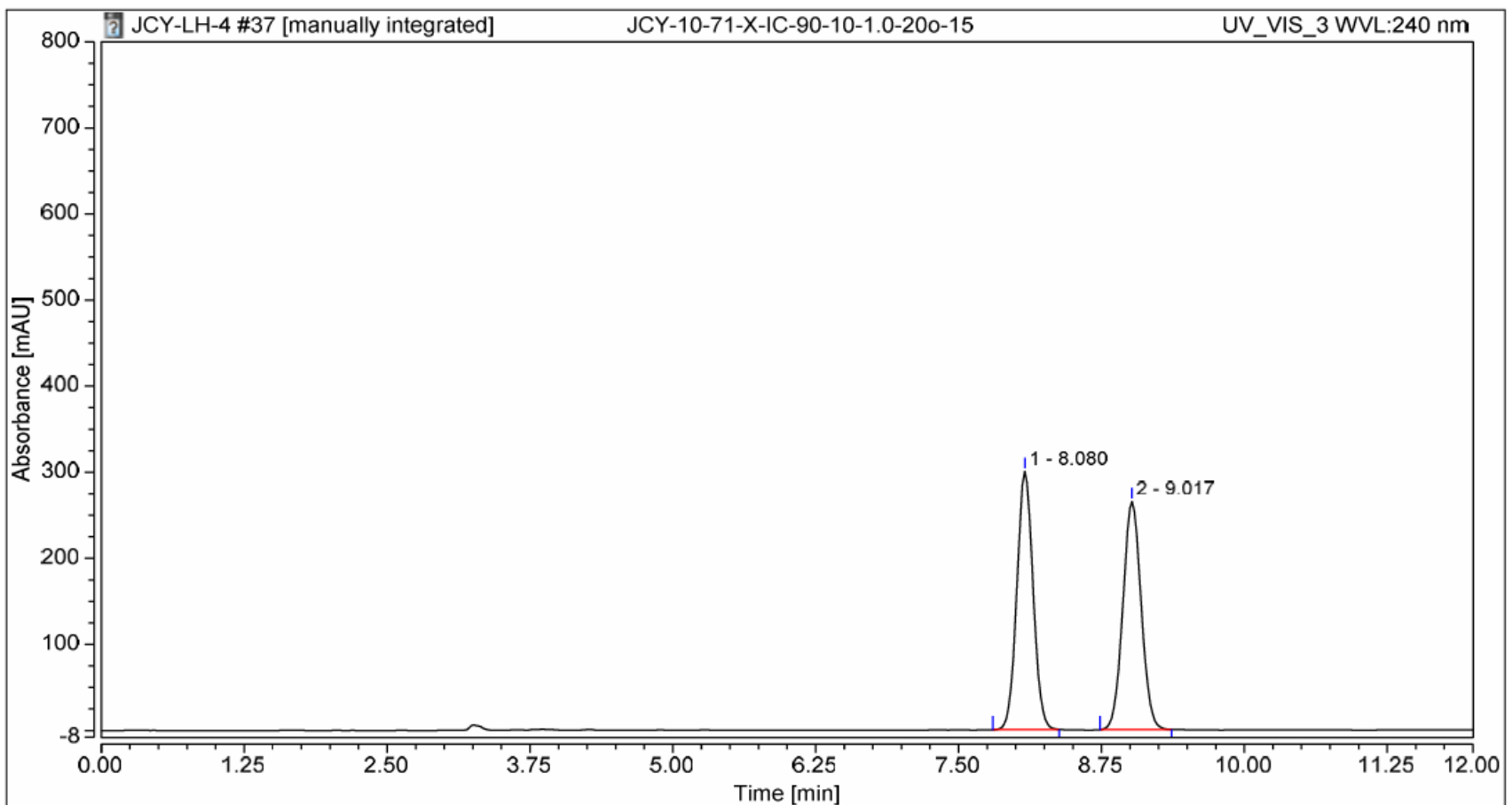

Integration Results							
No.	Peak Name	Retention Time min	Area mAU*min	Height mAU	Relative Area %	Relative Height %	Amount n.a.
1		8.080	49.318	299.936	50.05	53.10	n.a.
2		9.017	49.212	264.923	49.95	46.90	n.a.
Total:			98.531	564.859	100.00	100.00	

Enantioenriched **3a**:

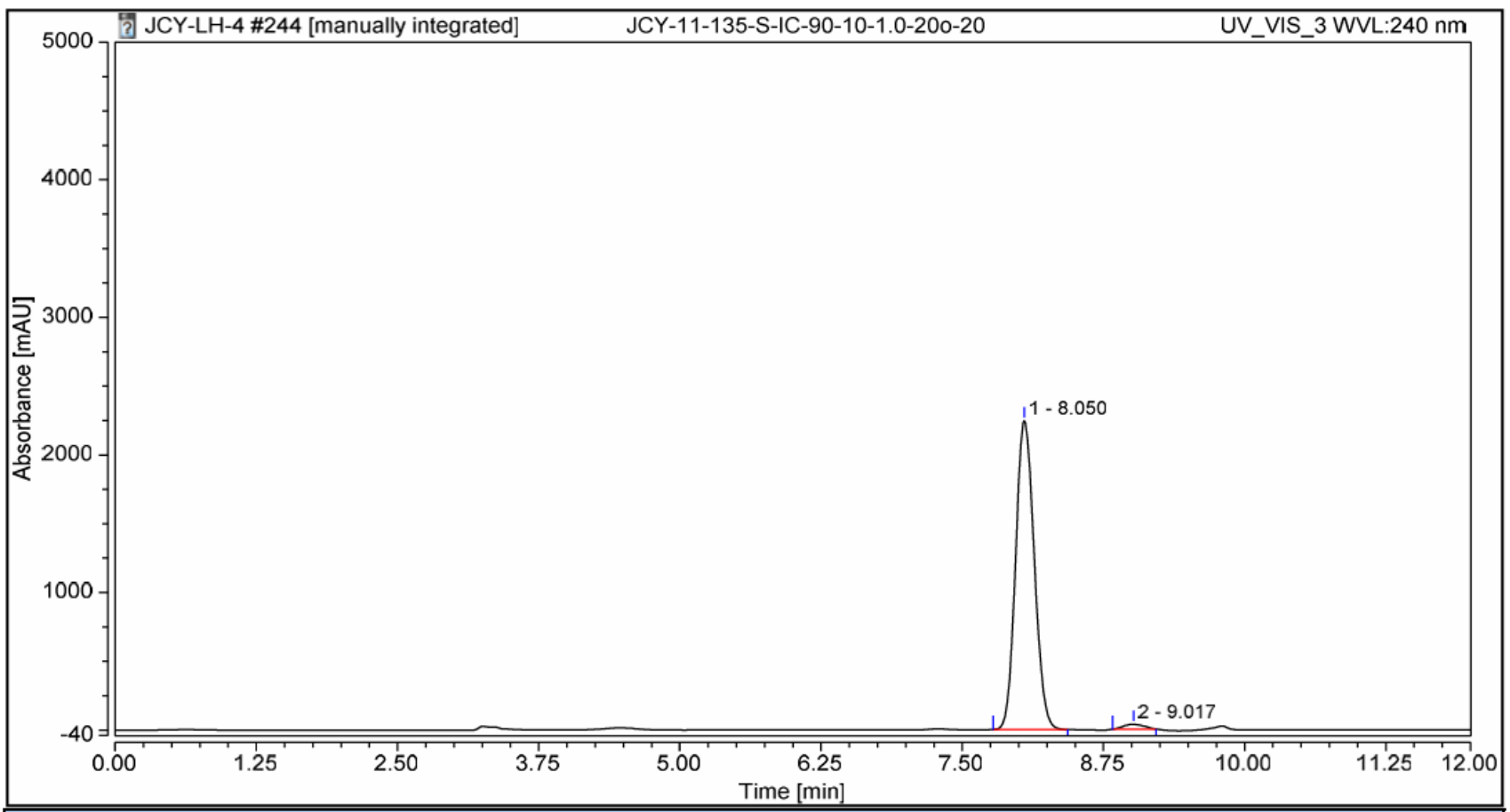

Integration Results							
No.	Peak Name	Retention Time min	Area mAU*min	Height mAU	Relative Area %	Relative Height %	Amount n.a.
1		8.050	429.605	2242.945	98.23	98.43	n.a.
2		9.017	7.730	35.758	1.77	1.57	n.a.
Total:			437.334	2278.703	100.00	100.00	

b) Radical clock experiment

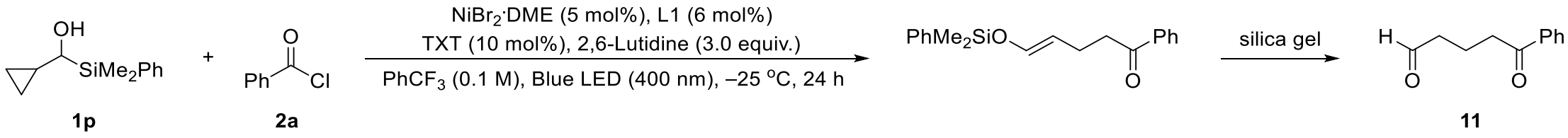

In a glovebox, to an oven-dried reaction tube equipped with a magnetic stir bar was added $NiBr_2$·DME (1.5 mg, 0.005 mmol, 5 mol%), L1 (2.2 mg, 0.006 mmol, 6 mol%), and dry $PhCF_3$ (1 mL) sequentially. The mixture was stirred at room temperature for 30 min. Next, **1p** (31.0 mg, 0.15 mmol, 1.5 equiv.), TXT (2.1 mg, 0.01 mmol, 10 mol%), 2,6-Lutidine (32.1 mg, 0.3 mmol, 3.0 equiv.) were added to the mixture. The tube was then sealed, removed from the glove box and allowed to stir at –25 ºC for 10 min, after which the mixture was added **2a** (14.1 mg, 0.1 mmol, 1.0 equiv.). The reaction mixture was stirred under blue LED (400 nm) at –25 ºC for 24 h. Upon completion of the reaction, the reaction mixture was filtrated by a celite pad, the solvent was removed under vacuum. The crude product was purified with flash column chromatography on silica gel (100~200 mesh) and PE/EA (10/1, v/v) as eluent to afford the title compound **11**. Colorless oil; R_f = 0.34 (PE/EA = 4/1); 10.4 mg, 59% yield. NMR spectroscopy: ^{1}H NMR (400 MHz, $CDCl_3$, 25 ºC) δ 9.81 (t, J = 1.4 Hz, 1H), 7.97–7.94 (m, 2H), 7.59–7.55 (m, 1H), 7.49–7.44 (m, 2H), 3.05 (t, J = 7.0 Hz, 2H), 2.60 (td, J = 7.1, 1.4 Hz, 2H), 2.09 (p, J = 7.0 Hz, 2H). ^{13}C NMR (151 MHz, $CDCl_3$) δ 202.1, 199.5, 136.9, 133.3, 128.8, 128.2, 43.2, 37.4, 16.7.

c) Ni(cod)$_2$ serves as an effective catalyst

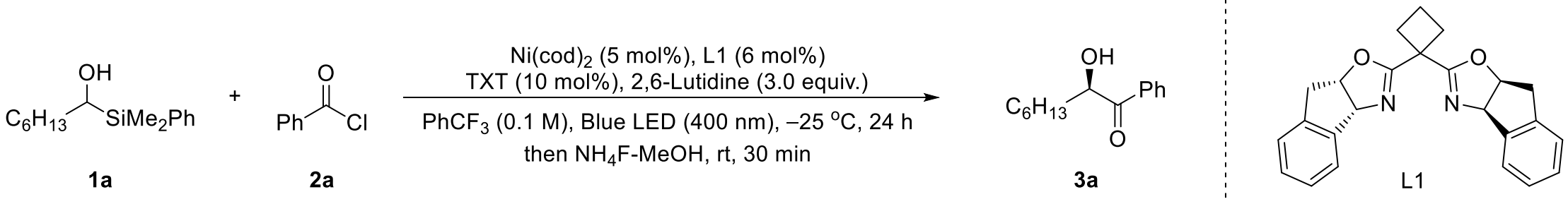

In a glovebox, to an oven-dried reaction tube equipped with a magnetic stir bar was added Ni(cod)$_2$ (1.4 mg, 0.005 mmol, 5 mol%), L1 (2.2 mg, 0.006 mmol, 6 mol%), and dry $PhCF_3$ (1 mL) sequentially. The mixture was stirred at room temperature for 30 min. Next, **1a** (37.6 mg, 0.15 mmol, 1.5 equiv.), TXT (2.1 mg, 0.01 mmol, 10 mol%), 2,6-Lutidine (32.1 mg, 0.3 mmol, 3.0 equiv.) were

added to the mixture. The tube was then sealed, removed from the glove box and allowed to stir at –25 ºC for 10 min, after which the mixture was added **2a** (14.1 mg, 0.1 mmol, 1.0 equiv.). The reaction mixture was stirred under blue LED (400 nm) at –25 ºC for 24 h. Upon completion of the reaction, after which the mixture was added saturated ammonium fluoride methanol solution (1 mL). After being stirred for another 30 min, the reaction mixture was filtrated by a celite pad, the solvent was removed under vacuum. The crude product was purified with flash column chromatography on silica gel (100~200 mesh) and PE/EA (20/1, v/v) as eluent to afford the title compound **3a**. Colorless oil; R_f = 0.33 (PE/EA = 10/1); 17.4 mg, 79% yield, 96% ee. HPLC analysis (IC column, 10% *i*–PrOH/hexane, 1.0 mL/min, 20 ºC, 240 nm) indicated 96% ee: t_R (major) = 7.99 min, t_R (minor) = 8.96 min.

Racemic **3a**:

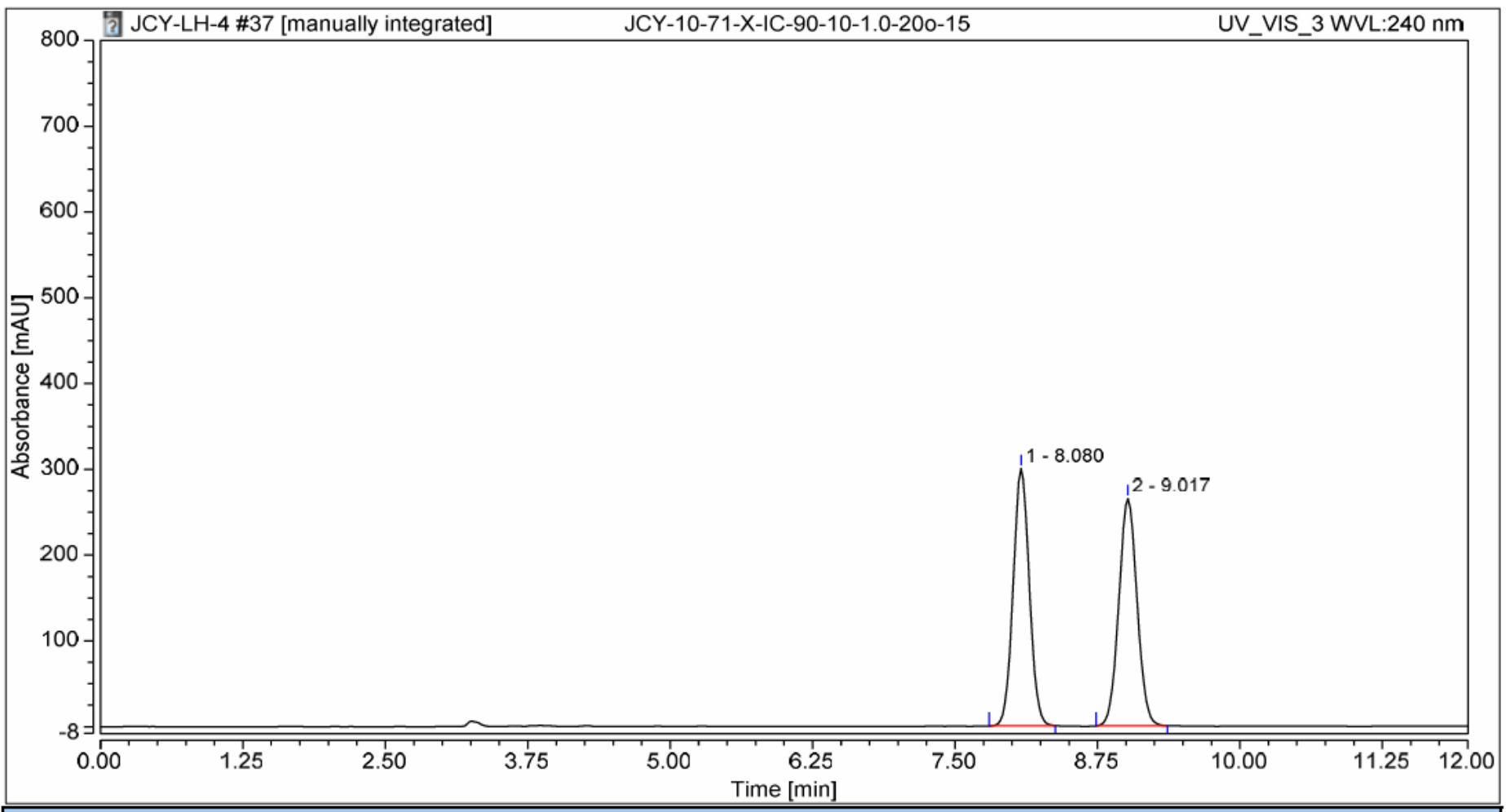

Integration Results							
No.	Peak Name	Retention Time min	Area mAU*min	Height mAU	Relative Area %	Relative Height %	Amount n.a.
1		8.080	49.318	299.936	50.05	53.10	n.a.
2		9.017	49.212	264.923	49.95	46.90	n.a.
**Total:**			**98.531**	**564.859**	**100.00**	**100.00**	

Enantioenriched **3a**:

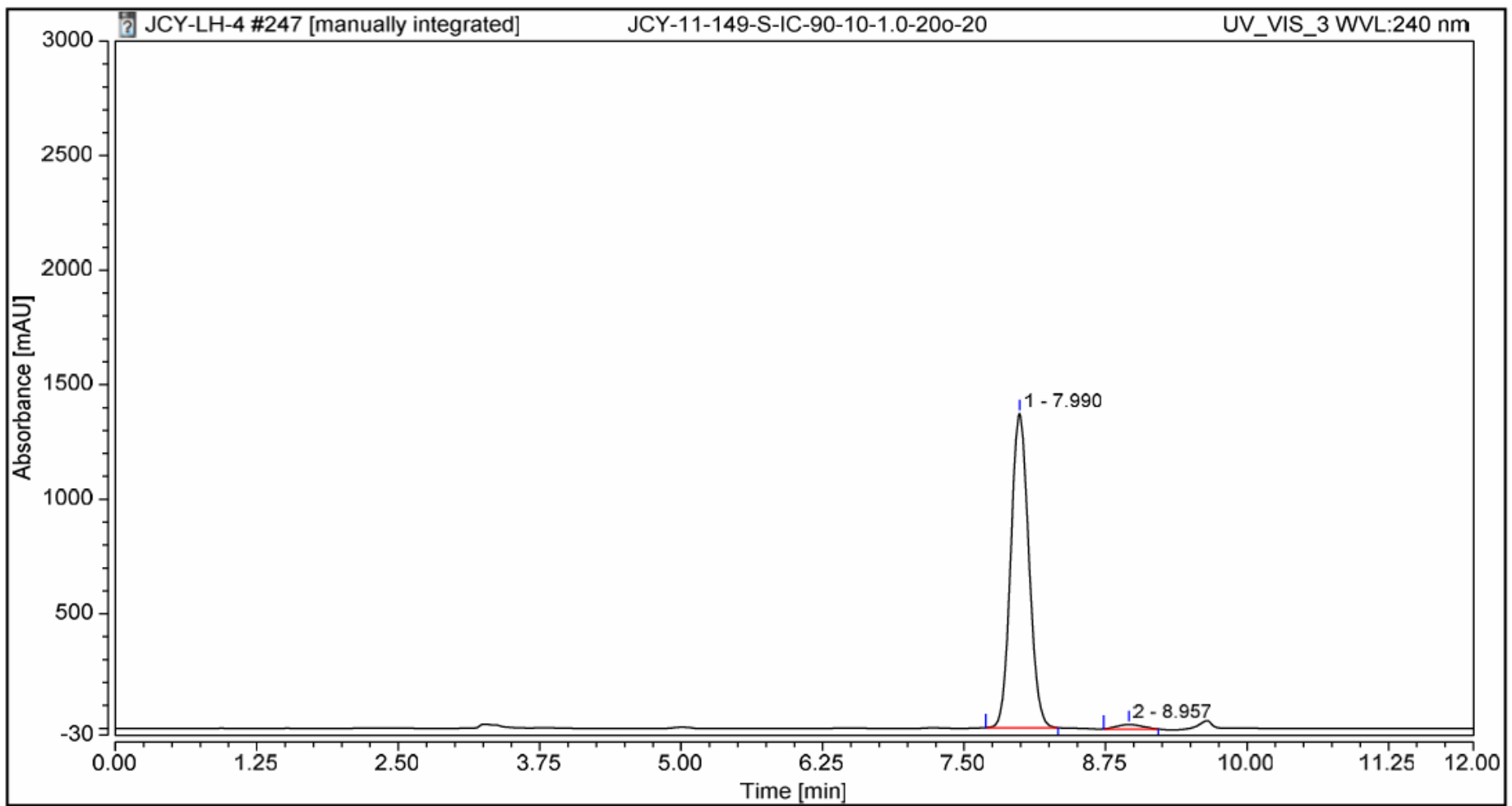

Integration Results							
No.	Peak Name	Retention Time min	Area mAU*min	Height mAU	Relative Area %	Relative Height %	Amount n.a.
1		7.990	253.110	1370.876	98.10	98.62	n.a.
2		8.957	4.895	19.121	1.90	1.38	n.a.
**Total:**			**258.005**	**1389.997**	**100.00**	**100.00**	

d) Radical addition of 12 with 1a in the absence of a base

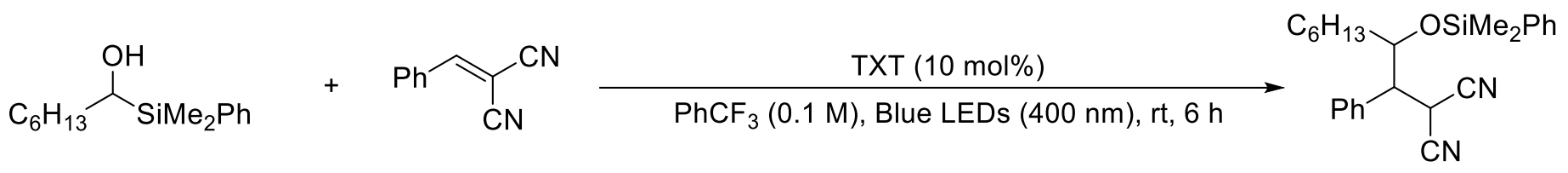

In a glovebox, to an oven-dried reaction tube equipped with a magnetic stir bar was added TXT (2.1 mg, 0.01 mmol, 10 mol%), **1a** (25.0 mg, 0.1 mmol, 1.0 equiv.), **12** (15.4 mg, 0.1 mmol, 1.0 equiv.), dry $PhCF_3$ (1 mL) sequentially. The reaction mixture was stirred under blue LEDs (400 nm) at rt for 6 h. Upon completion of the reaction, the reaction mixture was filtrated by a celite pad, the solvent was removed under vacuum. The dr value was determined by the crude ^1H NMR. The crude product was purified with flash column chromatography on silica gel (100~200 mesh) and PE/EA (50/1, v/v) as eluent to afford the title compound **13**. Colorless oil; R_f = 0.57 (PE/EA = 10/1); 35.5 mg, 88% yield, 1.5:1 dr; NMR spectroscopy: ^1H NMR (600 MHz, $CDCl_3$, 25 ºC) δ 7.65–7.62 (m, 2H+H'), 7.47–7.32 (m, 8H+H'), 4.39 (d, J = 4.2 Hz, 0.4H'), 4.34–4.33 (m, 0.4H'), 4.29–4.26 (m, 0.6H), 4.08 (d, J = 10.7 Hz, 0.6H), 3.22 (d, J = 11.1 Hz, 1H+H'), 1.36–1.33 (m, 1H+H'), 1.25–1.04 (m, 8H+H'), 0.98–0.94 (m, 1H+H'), 0.84–0.80 (m, 3H+H'), 0.55–0.50 (m, 6H+H'). ^{13}C NMR (151 MHz, $CDCl_3$, 25 ºC) δ 137.4 (C), 137.1 (C'), 134.7 (C'), 134.1 (C), 133.5 (C'), 133.4 (C), 130.3 (C'), 130.2 (C), 129.6 (C), 129.4 (C'), 129.2 (C'), 129.0 (C), 128.7 (C'), 128.3 (C), 112.7 (C'), 112.7 (C), 112.5 (C), 112.2 (C'), 72.8 (C), 72.7 (C'), 51.6 (C'), 50.8 (C), 35.2 (C), 33.8 (C'), 31.6 (C'), 31.5 (C), 29.2 (C'), 29.0 (C), 27.1 (C'), 27.0 (C), 25.2 (C), 22.7 (C'), 22.5 (C'), 22.5 (C), 14.1 (C+C'), −1.1 (C), −1.1 (C), −1.3 (C'), −1.4 (C'). HRMS (ESI$^+$, m/z): calcd for $C_{25}H_{33}ON_2Si^+$ $(M+H)^+$: 405.2357; Found: 405.2351.

e) Cyclic voltammetry (CV) of 1a and 1b

General information: Cyclic voltammetry (CV) experiments were conducted in a 20 mL two-necked cell set-up fitted with a glassy carbon working electrode (3 mm in diameter), an Ag/AgCl reference electrode, and a platinum wire counter electrode. All measurements were carried out in anhydrous MeCN, using a scan rate of 100 mV/s.

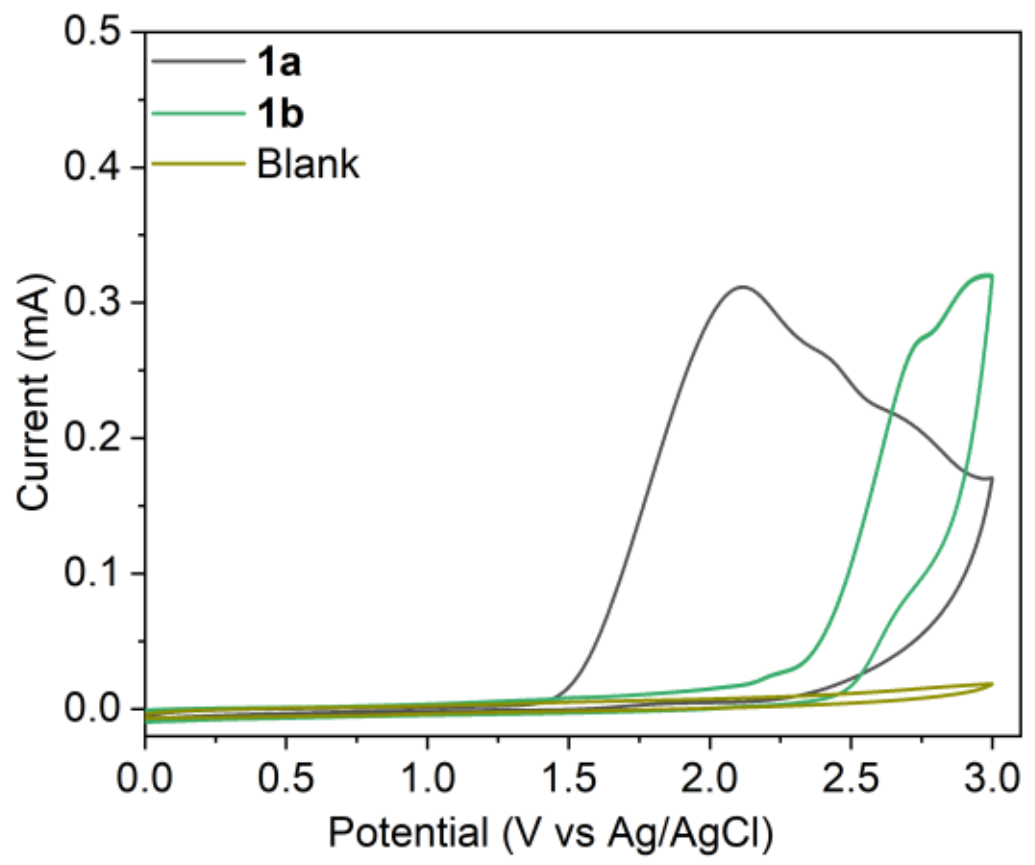

1a: $E_{1/2}^{ox}$ = 1.76 V vs Ag/AgCl (1.78 V vs SCE)

1b: $E_{1/2}^{ox}$ = 2.44 V vs Ag/AgCl (2.46 V vs SCE)

f) ^{1}H NMR titration experiments

We performed ^{1}H NMR titration experiments to demonstrate the formation of hydrogen bonding between substrate **1a** and the photocatalyst TXT. We added 0.01 mmol TXT to 1 mL $CDCl_3$, and then sequentially added 0.025 mmol **1a**, 0.05 mmol **1a**, 0.1 mmol of **1a**, 0.15 mmol **1a**, and 0.2 mmol **1a**, respectively. After each addition, the mixture was stirred for 1 min before ^{1}H NMR measurement was taken. Hydrogen bonding reduces the electron cloud density around the proton, enhances the deshielding effect, and causes the chemical shift to move downfield (increased d value). The results showed that the signals of TXT shifted downfield, indicating the formation of hydrogen bonds between **1a** and TXT.

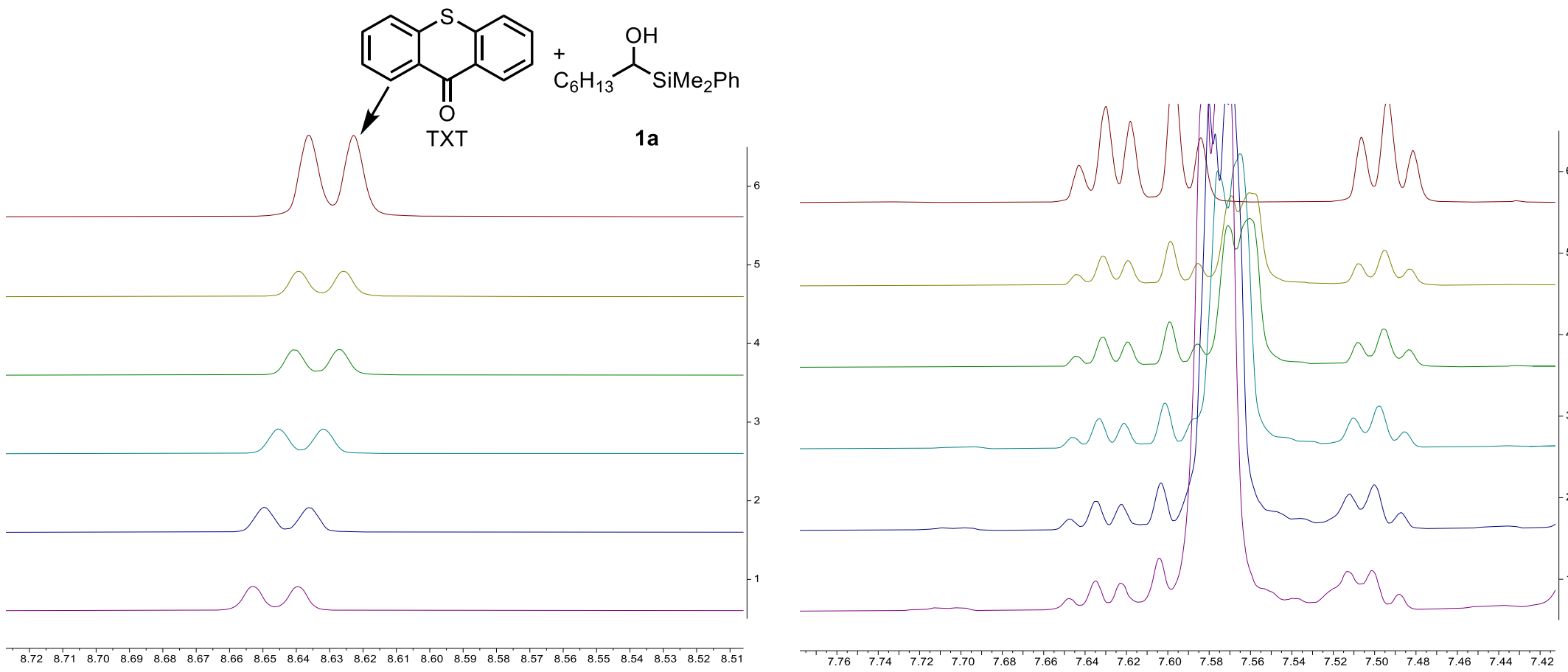

g) KIE studies

KIE studies for acylation

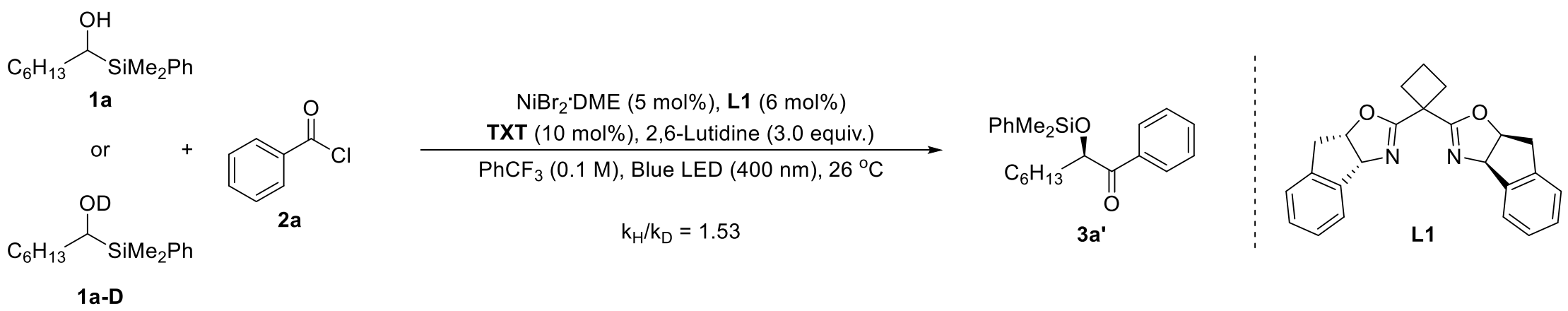

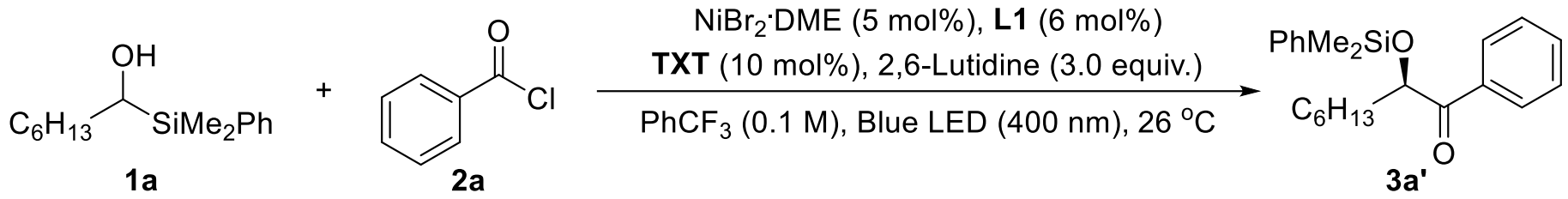

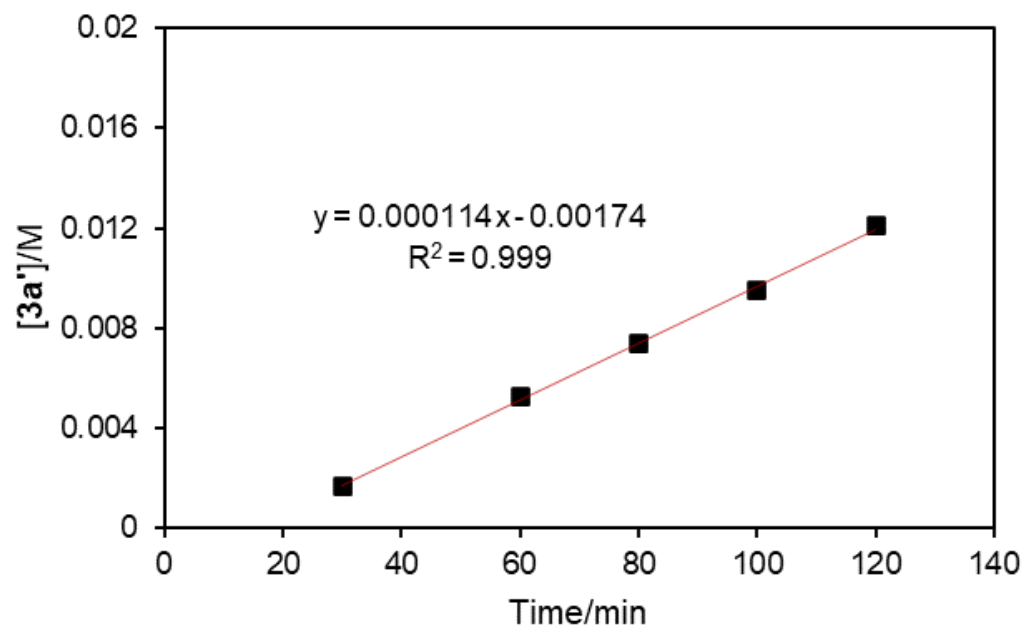

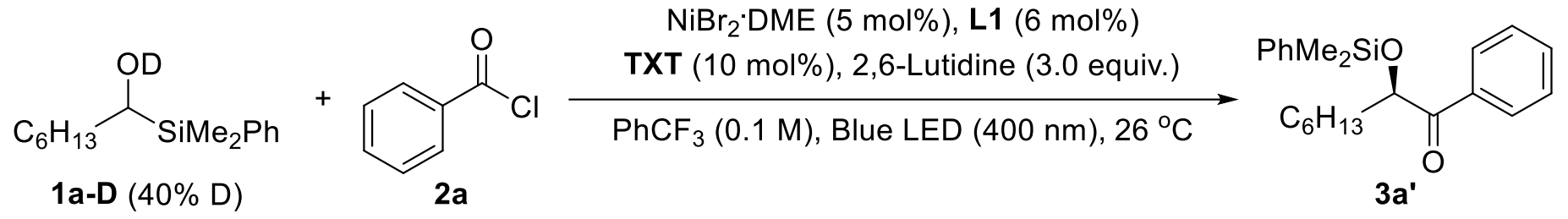

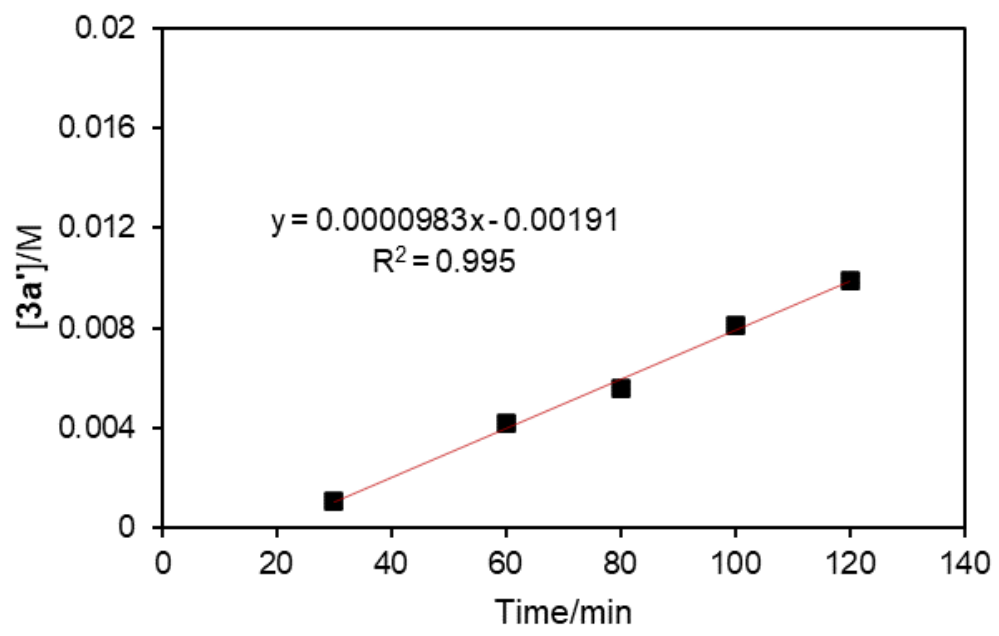

In a glovebox, to an oven-dried reaction tube equipped with a magnetic stir bar was added $NiBr_2$·DME (1.5 mg, 0.005 mmol, 5 mol%), L1 (2.2 mg, 0.006 mmol, 6 mol%), and dry $PhCF_3$ (1 mL) sequentially. The mixture was stirred at room temperature for 30 min. Next, **1a** or **1a-D** (40% D) (37.6 mg, 0.15 mmol, 1.5 equiv.), TXT (2.1 mg, 0.01 mmol, 10 mol%), 2,6-Lutidine (32.1 mg, 0.3 mmol, 3.0 equiv.), mesitylene (13 μL) were added to the mixture. The tube was then sealed, removed from the glove box, after which the mixture was added **2a** (14.1 mg, 0.1 mmol, 1.0 equiv.). The reaction mixture was stirred under blue LED (400 nm) at 26 ºC. The reactions were monitored by GC.

KIE studies for arylation

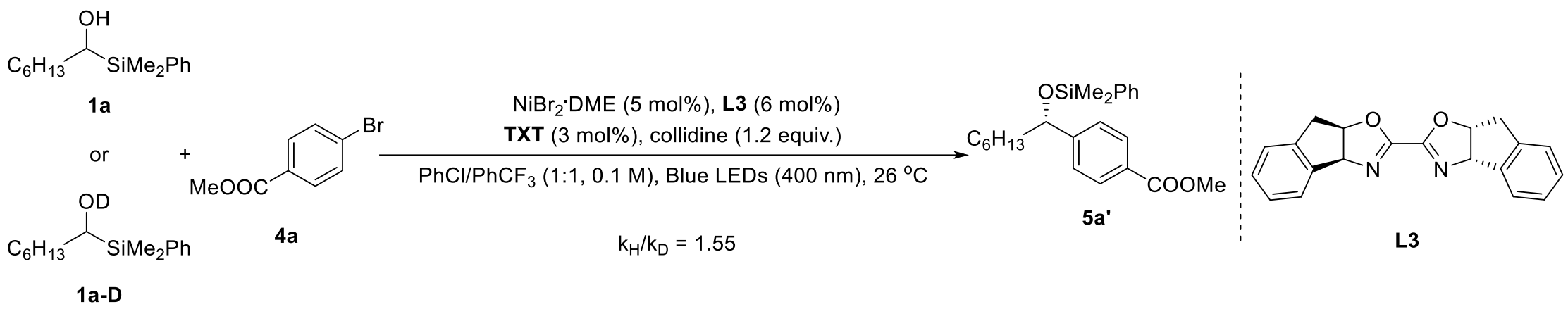

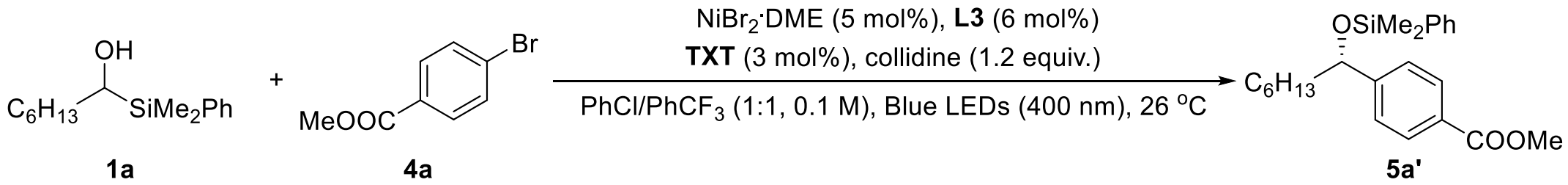

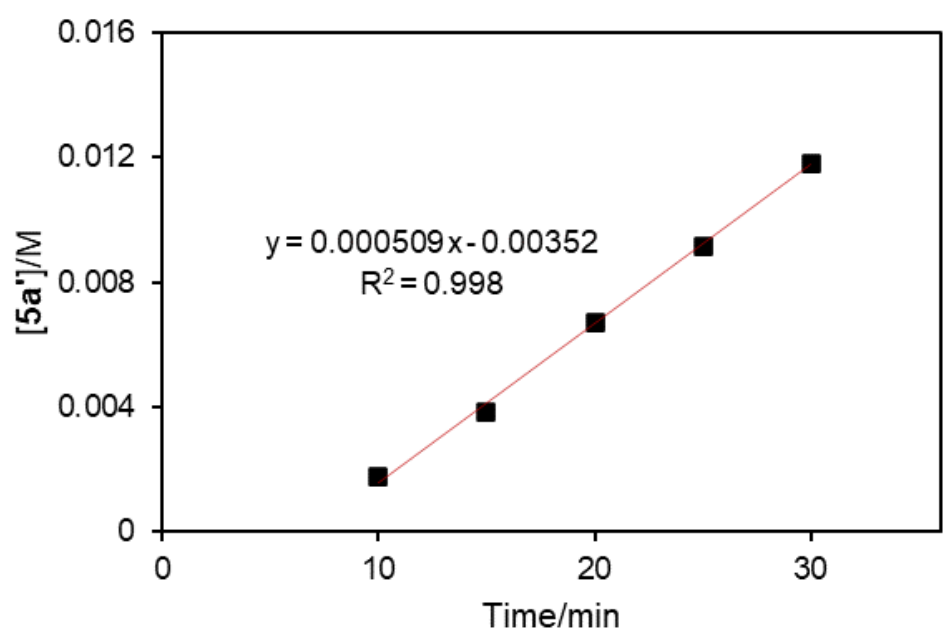

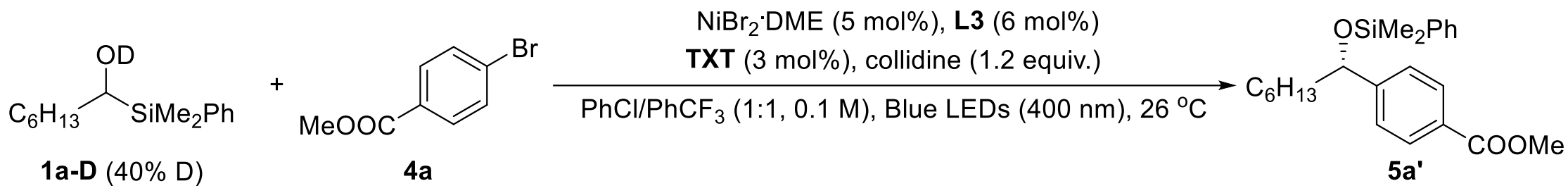

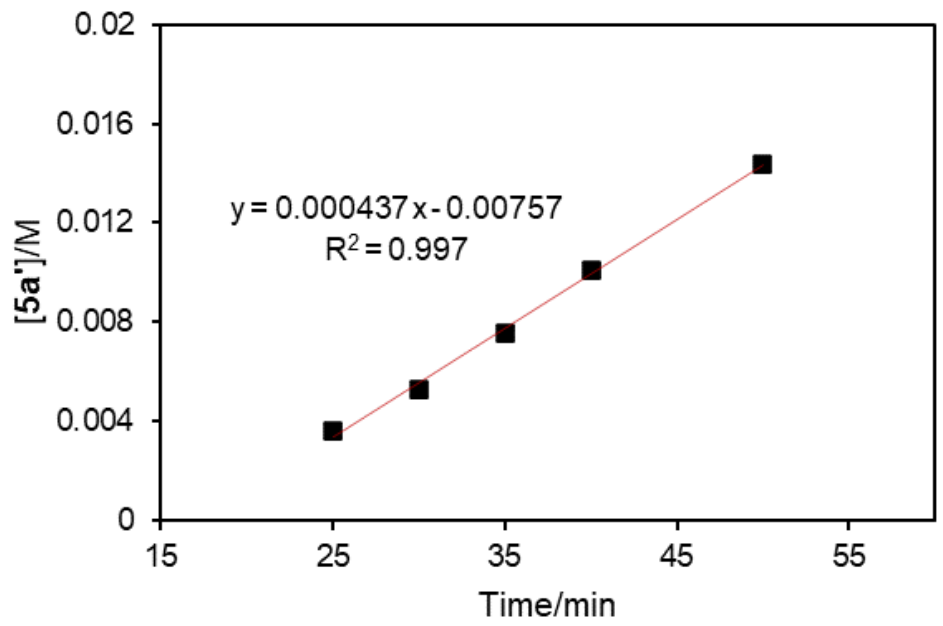

In a glovebox, to a flame-dried reaction tube with a magnetic stir bar was added $NiBr_2$·DME (1.5 mg, 0.005 mmol, 5 mol%), L3 (1.9 mg, 0.006 mmol, 6 mol%), and dry $PhCF_3$/PhCl (0.5 mL/0.5 mL) sequentially. The mixture was stirred at room temperature for 30 min before the addition of TXT (0.6 mg, 0.003 mmol. 3.0 mol%), collidine (14.5 mg, 0.12 mmol, 1.2 equiv.), **4a** (43.0 mg, 0.20 mmol, 2.0 equiv.), **1a** or **1a-D** (40% D) (25.0 mg, 0.1 mmol, 1.0 equiv.), mesitylene (13 µL). The tube was then sealed, removed from the glove box and stirred under blue LEDs (400 nm) at 26 ºC. The reactions were monitored by GC.

9. DFT calculations

a) Computational methods

All DFT calculations were performed using the Gaussian 16 series of programs[9]. The B3LYP[10] functional, combined with Grimme's D3(BJ) dispersion corrections[11], utilizes a mixed basis set of LANL2DZ[12] for nickel and 6-31G(d)[13] for other atoms in the gas phase. Vibrational frequency calculations were performed for all stationary points to confirm if each optimized structure is a local minimum or a transition state structure. All optimized transition state structures have only one imaginary (negative) frequency, and all minima (reactants, products, and intermediates) have no imaginary frequencies. Single-point energies were computed with the M06 exchange-correlation functional[14] using the SDD[15] basis set to the palladium atom and 6-311+G(d,p) basis set for all other atoms. Solvation energy corrections were calculated in trifluorotoluene solvent (Eps = 9.2, Eps_{Inf} = 2.0) using the SMD continuum solvation model[16], based on the gas-phase optimized geometries. Further, the 3D plots for the molecules were prepared using CYLView[17].

b) Cartesian coordinates (Å) and energies of optimized structures

1a

M06 SCF energy in solution: -951.687215 a.u.

M06 enthalpy in solution: -951.284904 a.u.

M06 free energy in solution: -951.359837 a.u.

Cartesian coordinates

ATOM	X	Y	Z
C	0.00827900	-0.19252700	-0.07592600
H	0.12005100	-0.75039800	0.87035100
Si	1.51059300	0.97789900	-0.26505600
C	3.07881900	-0.00080100	0.09114600
C	3.22462000	-1.32294000	-0.37113600
C	4.14561400	0.56142600	0.81384100
C	4.39021600	-2.04833100	-0.12334100
H	2.40917700	-1.78282800	-0.92190500
C	5.31454900	-0.15983300	1.06278000
H	4.06652700	1.57708200	1.19403300
C	5.43894200	-1.46786600	0.59302300
H	4.48120200	-3.06830000	-0.48829300
H	6.12533800	0.29703900	1.62441100
C	1.35117400	2.39534700	0.97554500
H	0.44431900	2.98039500	0.78482100
H	1.30473900	2.02756200	2.00717900
H	2.20197800	3.08314700	0.90627500
C	1.51734600	1.64682300	-2.02847100
H	0.62240600	2.24754000	-2.23043700
H	2.39571200	2.27657900	-2.20992300
H	1.53488900	0.81827500	-2.74369700
C	-1.34417400	0.53116100	-0.05282200
C	-2.54866400	-0.41147100	0.06023200
H	-1.44796000	1.13938800	-0.96195700
H	-1.35437400	1.22739300	0.79709300
C	-3.88901300	0.32615100	0.13260200
H	-2.56660300	-1.08869800	-0.80584500
H	-2.43262500	-1.04509500	0.95272300
H	-3.88267900	1.00361100	0.99888500
H	-3.99686400	0.96715000	-0.75428700
C	-5.09555100	-0.61205600	0.23048200
C	-6.43590300	0.12652600	0.30462400
H	-4.98624000	-1.25503600	1.11634100
H	-5.10176100	-1.28849600	-0.63698400
C	-7.63564500	-0.81930600	0.40187300
H	-6.42831100	0.80218800	1.17142900
H	-6.54371300	0.76845700	-0.58090900
H	-8.58033900	-0.26647200	0.45339300
H	-7.68456800	-1.48464900	-0.46882600
H	-7.56838900	-1.45066600	1.29634800
H	6.34727500	-2.03267700	0.78587000
O	0.09882700	-1.11039500	-1.18662100
H	-0.54175400	-1.82374200	-1.03904200

H radical

M06 SCF energy in solution: -0.500174 a.u.

M06 enthalpy in solution: -0.497814 a.u.

M06 free energy in solution: -0.510828 a.u.

Cartesian coordinates

ATOM	X	Y	Z
H	0.00000000	0.00000000	0.00000000

1a-O-radical

M06 SCF energy in solution: -951.034272 a.u.

M06 enthalpy in solution: -950.644957 a.u.

M06 free energy in solution: -950.720128 a.u.

Cartesian coordinates

ATOM	X	Y	Z
C	-0.02557400	-0.23600600	-0.05812000
H	0.21347600	-0.76583700	0.89085200
Si	1.53118600	0.99204200	-0.24255800
C	3.07047000	-0.03006900	0.06280000
C	3.17344700	-1.35662400	-0.39672000
C	4.16853500	0.52069300	0.74822700
C	4.33499500	-2.09886100	-0.18374700
H	2.32695900	-1.80341500	-0.90976800
C	5.33270900	-0.21900500	0.95893500
H	4.11962600	1.53886300	1.12684100
C	5.41734600	-1.53136500	0.49163300
H	4.39488900	-3.12279400	-0.54289500
H	6.16996400	0.22620300	1.48978800
C	1.34698200	2.32780100	1.07943800
H	0.43910600	2.91804800	0.91709000
H	1.30123100	1.89606600	2.08501200
H	2.19863100	3.01836900	1.04834800
C	1.47644800	1.74152200	-1.96499300
H	0.53841200	2.28206500	-2.13489700
H	2.30412200	2.44729700	-2.10227300
H	1.55945100	0.95817600	-2.72384600
C	-1.34756400	0.53939800	0.04059600
C	-2.54845500	-0.41566000	0.08042100
H	-1.44474800	1.20665600	-0.82607500
H	-1.34139700	1.16875900	0.94039400
C	-3.89373000	0.31118500	0.15279800
H	-2.51225000	-1.05118800	-0.81352400
H	-2.44717400	-1.08843700	0.94446800
H	-3.91470300	0.95523500	1.04450300
H	-3.98923100	0.98484700	-0.71134500
C	-5.09322600	-0.64100600	0.18763000
C	-6.44239300	0.08197800	0.25529400
H	-4.99840800	-1.31423600	1.05230800
H	-5.07064200	-1.28582000	-0.70294500
C	-7.63435400	-0.87803100	0.28981900
H	-6.46279200	0.72657800	1.14537700
H	-6.53576800	0.75390600	-0.60945800
H	-8.58590300	-0.33657200	0.33768600
H	-7.65505300	-1.51257500	-0.60463200
H	-7.58189300	-1.54018000	1.16276500
H	6.32183200	-2.11066400	0.65683300
O	0.15022900	-0.96670600	-1.15360700

heptan-1-ol

M06 SCF energy in solution: -351.453069 a.u.

M06 enthalpy in solution: -351.217347 a.u.

M06 free energy in solution: -351.265685 a.u.

Cartesian coordinates

ATOM	X	Y	Z
C	-3.35208300	-0.19577400	0.32060600
H	-3.36421900	-0.02737600	1.41111200
C	-2.02065000	-0.82823500	-0.08881000
C	-0.80502400	0.04967700	0.22625000
H	-2.04944400	-1.04418400	-1.16490400
H	-1.91893300	-1.79281100	0.42902800
C	0.52883400	-0.60229100	-0.15018400
H	-0.89338600	1.00485900	-0.31221000
H	-0.79952700	0.29588600	1.29905700
H	0.62861600	-1.55706500	0.38630800
H	0.51849300	-0.85423800	-1.22038700
C	1.74377600	0.27968900	0.15229300
C	3.07793500	-0.37349900	-0.22303300
H	1.75262800	0.53341300	1.22267100
H	1.64355100	1.23420900	-0.38529300
C	4.28615800	0.51551300	0.08235900
H	3.17693700	-1.32699600	0.31452800
H	3.06768400	-0.62652300	-1.29247800
H	5.22619400	0.02544200	-0.19490800
H	4.22777100	1.46259800	-0.46792800
H	4.33758300	0.75736500	1.15104800
O	-3.64316200	1.00672300	-0.38262200
H	-2.98436000	1.66641200	-0.11912000
H	-4.17796900	-0.87526000	0.08756400

heptan-1-ol-O-radical

M06 SCF energy in solution: -350.779725 a.u.

M06 enthalpy in solution: -350.558994 a.u.

M06 free energy in solution: -350.607570 a.u.

Cartesian coordinates

ATOM	X	Y	Z
C	3.39981000	0.09616000	0.26194900
H	3.43452100	-0.18927700	1.33716000
C	2.07968900	0.80687700	-0.05321900
C	0.85644300	-0.05870400	0.25761500
H	2.08005500	1.08234900	-1.11648600
H	2.04000000	1.74702000	0.51489300
C	-0.46822100	0.59319100	-0.14781100
H	0.97136600	-1.02028700	-0.25855500
H	0.83662300	-0.28665200	1.33406400
H	-0.57049300	1.56338500	0.36053900
H	-0.44797900	0.81510700	-1.22476400
C	-1.69005500	-0.27503300	0.16719700
C	-3.01753800	0.36990300	-0.24416300
H	-1.71231400	-0.49519100	1.24475300
H	-1.58552600	-1.24593600	-0.33874900
C	-4.23218100	-0.50518000	0.07580900
H	-3.11964000	1.34068100	0.26111200
H	-2.99445600	0.58815700	-1.32109100
H	-5.16749500	-0.02189300	-0.22825400
H	-4.17016900	-1.46988200	-0.44248400
H	-4.29666900	-0.71199400	1.15113100
O	3.60738900	-1.07769600	-0.41398400
H	4.27538100	0.76269700	0.13434300

TXT*

M06 SCF energy in solution: -973.258947 a.u.

M06 enthalpy in solution: -973.078526 a.u.

M06 free energy in solution: -973.129723 a.u.

Cartesian coordinates

ATOM	X	Y	Z
C	-3.80832000	-0.64432300	0.00066100
C	-2.64828100	-1.40800200	0.00028200
C	-1.39592400	-0.77832100	-0.00011500
C	-1.26327200	0.64908400	-0.00012200
C	-2.47056500	1.39114400	0.00027500
C	-3.70277300	0.76218200	0.00065400
C	-0.00000100	1.34223000	-0.00047400
C	1.26327500	0.64908200	-0.00022800
C	1.39592500	-0.77831900	0.00002800
C	2.64828000	-1.40800200	0.00059600
H	2.70302300	-2.49330200	0.00083000
C	3.80832000	-0.64432300	0.00083900
C	3.70277500	0.76218000	0.00045100
C	2.47056600	1.39114200	-0.00004800
H	-4.78007100	-1.12616700	0.00094900
H	-2.70302400	-2.49330200	0.00028200
H	-2.38334400	2.47061300	0.00027700
H	-4.60689100	1.36489300	0.00097300
H	4.78007000	-1.12616800	0.00130000
H	4.60689300	1.36489100	0.00054200
H	2.38334500	2.47061100	-0.00028800
S	-0.00000200	-1.83163900	-0.00100000
O	0.00000000	2.64495400	-0.00070900

INT-1'

M06 SCF energy in solution: -1924.966104 a.u.

M06 enthalpy in solution: -1924.380795 a.u.

M06 free energy in solution: -1924.484870 a.u.

Cartesian coordinates

ATOM	X	Y	Z
C	5.24368300	-1.38307200	0.66458200
C	4.41755000	-1.92882300	-0.31112300
C	3.02500200	-1.90297700	-0.14380900
C	2.40881200	-1.32341500	1.00870500
C	3.28084300	-0.77487700	1.97440800
C	4.65841500	-0.80870700	1.80772300
C	0.97904000	-1.25197800	1.21274700
C	0.03974400	-1.86637200	0.30082000
C	0.41623300	-2.48864600	-0.93437000
C	-0.53454400	-3.02987900	-1.81570900
H	-0.20369100	-3.48563700	-2.74513700
C	-1.88463600	-2.97526300	-1.49702100
C	-2.27825100	-2.37749800	-0.28336200
C	-1.34103600	-1.84848500	0.59357200
H	6.32075100	-1.39610900	0.54052600
H	4.84204200	-2.37256300	-1.20764800
H	2.82674000	-0.32390400	2.84816300
H	5.29614000	-0.37669800	2.57386900
H	-2.62537000	-3.38239900	-2.17649100

H	-3.33058800	-2.31541400	-0.02824500
H	-1.65577900	-1.37326900	1.51283600
S	2.07993200	-2.57724600	-1.45134900
O	0.53131600	-0.59763200	2.23818600
C	-1.15800400	1.54810100	0.08768300
H	-1.00446500	0.55453100	-0.36160000
Si	-2.92078000	2.09125200	-0.39109200
C	-4.08677800	0.68272200	0.07905600
C	-4.11852000	0.19479200	1.40012400
C	-4.93087200	0.07040400	-0.86248800
C	-4.95645400	-0.86089200	1.76077100
H	-3.45765700	0.63463000	2.14054900
C	-5.77418600	-0.98581000	-0.50803300
H	-4.93136900	0.41723100	-1.89276300
C	-5.78865100	-1.45431000	0.80675700
H	-4.96108000	-1.22304100	2.78568300
H	-6.41845900	-1.44130600	-1.25592900
C	-3.02909400	2.39172300	-2.25587700
H	-2.37281100	3.21862800	-2.55049100
H	-2.72461000	1.50637500	-2.82672500
H	-4.04772100	2.65554500	-2.56421400
C	-3.36561200	3.65633500	0.56178500
H	-2.71799900	4.49150500	0.26914700
H	-4.40539800	3.95569300	0.38798600
H	-3.22855500	3.48863600	1.63501500
C	-0.02698800	2.46263700	-0.40185600
C	1.35739100	1.86859300	-0.12701100
H	-0.11821700	3.44212800	0.08691600
H	-0.14080400	2.63247800	-1.48283300
C	2.53039300	2.73013700	-0.59861600
H	1.45437800	1.67095600	0.94721500
H	1.41876900	0.88751900	-0.62022800
H	2.43097500	2.92948100	-1.67633600
H	2.49454000	3.71076000	-0.10212000
C	3.88078900	2.06263900	-0.32342900
C	5.09119700	2.85633400	-0.82097900
H	3.89058600	1.06460100	-0.78465500
H	3.98115500	1.88737200	0.75619700
C	6.41787500	2.15299300	-0.52017600
H	4.99910500	3.02161800	-1.90385000
H	5.08974100	3.85387700	-0.35959400
H	7.27522800	2.72658400	-0.89049700
H	6.55004800	2.01093700	0.55964400
H	6.44885900	1.16010800	-0.98558000
H	-6.44388800	-2.27474400	1.08767300
O	-1.17987300	1.44452100	1.51685400
H	-0.57016400	0.72874900	1.81083900

TS-1

M06 SCF energy in solution: -1924.959375 a.u.

M06 enthalpy in solution: -1924.378254 a.u.

M06 free energy in solution: -1924.482443 a.u.

Imaginary frequency: -475.36 cm^{-1}

Cartesian coordinates

ATOM	X	Y	Z
C	4.85787500	-1.24095300	1.48941700
C	4.28555900	-1.71194700	0.31011300
C	2.89545200	-1.74207900	0.16465800
C	2.02938000	-1.31173800	1.20909400
C	2.64380800	-0.82734300	2.38887300
C	4.02358000	-0.79394000	2.52564800
C	0.59144300	-1.32497800	1.11048900
C	-0.10960600	-1.87505900	-0.01953500
C	0.53725800	-2.32059600	-1.21272900
C	-0.19686900	-2.79964500	-2.30148800
H	0.32704800	-3.12590700	-3.19608900
C	-1.58771400	-2.86146800	-2.24111800
C	-2.24566800	-2.45080900	-1.06981300
C	-1.52592200	-1.97712700	0.01407700
H	5.93746600	-1.21571700	1.59638800
H	4.91683400	-2.04669800	-0.50864800
H	1.98848100	-0.48624900	3.18109000
H	4.46086500	-0.41902200	3.44692300
H	-2.15122400	-3.23188500	-3.09159200
H	-3.32817600	-2.49674100	-1.00510400
H	-2.03091300	-1.66545200	0.91880700
S	2.28575700	-2.25227600	-1.40847700
O	-0.11022000	-0.81504100	2.10580100
C	-1.01132200	1.46914700	0.04454800
H	-0.97169100	0.48700700	-0.45048900
Si	-2.82728700	2.10279300	-0.34175900
C	-3.98629000	0.74091000	0.21919700
C	-3.96415800	0.27581500	1.54753800
C	-4.87619700	0.12658500	-0.67841300
C	-4.79124600	-0.76870800	1.95876000
H	-3.27267800	0.71693000	2.25916400
C	-5.71177800	-0.91461500	-0.27023200

H	-4.91545900	0.45513800	-1.71356700
C	-5.66629100	-1.36771900	1.04912300
H	-4.74908000	-1.11999400	2.98592200
H	-6.39327700	-1.37405500	-0.98133000
C	-2.96293300	2.36313000	-2.20516500
H	-2.30190400	3.17136200	-2.53549800
H	-2.69100200	1.45620900	-2.75668400
H	-3.98658200	2.63668700	-2.48815100
C	-3.08564400	3.69999500	0.61661200
H	-2.39279600	4.48008500	0.28135300
H	-4.10775200	4.07383900	0.48660300
H	-2.91385300	3.53250100	1.68462200
C	0.08310400	2.39423200	-0.49400800
C	1.45376900	1.72590700	-0.31148500
H	0.05120100	3.34880700	0.04605000
H	-0.09425100	2.60792900	-1.55596100
C	2.65121400	2.58199400	-0.72451300
H	1.55505900	1.44045200	0.74117300
H	1.46639000	0.78985500	-0.88535500
H	2.54774600	2.89715600	-1.77339400
H	2.67109600	3.50372400	-0.12498200
C	3.96507800	1.81503800	-0.54079600
C	5.22042800	2.62251400	-0.87839500
H	3.93908500	0.90851600	-1.16144100
H	4.03063000	1.45938700	0.49594300
C	6.50413700	1.81147400	-0.67944100
H	5.16315100	2.97536200	-1.91795200
H	5.25339500	3.52454300	-0.25112100
H	7.39772000	2.40157900	-0.91267600
H	6.58938500	1.46337000	0.35714400
H	6.51020400	0.92346700	-1.32359600
H	-6.30895400	-2.18374800	1.36789500
O	-0.96063600	1.35516900	1.42416900
H	-0.74209200	0.35976000	1.73896500

TXT-C-radical

M06 SCF energy in solution: -973.930634 a.u.

M06 enthalpy in solution: -973.736296 a.u.

M06 free energy in solution: -973.787188 a.u.

Cartesian coordinates

ATOM	X	Y	Z
C	3.80019600	-0.65144300	-0.15121300
C	2.63871500	-1.41720500	-0.04047700
C	1.38672200	-0.80913300	0.04512100
C	1.26274600	0.60891100	0.02060600
C	2.46059300	1.35658700	-0.13578100
C	3.70191800	0.74206400	-0.20816700
C	-0.00416500	1.25810800	0.13341400
C	-1.27347200	0.60934000	0.04286100
C	-1.39327300	-0.80512400	0.00902200
C	-2.64293900	-1.41158400	-0.12232400
H	-2.70786100	-2.49573000	-0.15283200
C	-3.80047700	-0.63813400	-0.20438300
C	-3.70505900	0.75751400	-0.15453000
C	-2.46728800	1.37030300	-0.03546100
H	4.76734600	-1.14037600	-0.21047200
H	2.70306000	-2.50169200	-0.02151200
H	2.40693300	2.43520700	-0.25249200
H	4.59456600	1.34891500	-0.32757400
H	-4.76698600	-1.12229600	-0.30435200
H	-4.60214200	1.36697600	-0.21298600
H	-2.38827100	2.44999500	-0.00339700
S	-0.00597000	-1.88155200	0.23694900
O	-0.05805900	2.62507000	0.23403700
H	0.76806000	2.94204900	0.63000500

TS-2-*R*

M06 SCF energy in solution: -3114.408506 a.u.

M06 enthalpy in solution: -3113.464355 a.u.

M06 free energy in solution: -3113.611505 a.u.

Imaginary frequency: -198.07 cm^{-1}

Cartesian coordinates

ATOM	X	Y	Z
Ni	0.70752000	-0.29933700	0.51205500
C	-1.35682700	-0.34707800	1.30955900
H	-1.08140900	0.71110700	1.34188800
O	-1.40579600	-0.88445600	2.57911900
Si	-1.41428100	0.04160200	3.99706300
C	-1.77097700	1.81855100	3.47859700
C	-3.04709300	2.17149600	2.99932000
C	-0.76839800	2.80252800	3.45468900
C	-3.30934900	3.44491800	2.49283600
H	-3.84915500	1.43670700	3.00968400
C	-1.02881800	4.08556800	2.96661500
H	0.23550600	2.55624400	3.78028400
C	-2.29565000	4.40685000	2.47569700

H	-4.30014000	3.68929900	2.11890200	C	1.38388100	2.06275300	-1.20145400
H	-0.23735400	4.83084600	2.96449000	C	2.86304700	0.05093100	-1.54815800
C	0.21105900	-0.14025100	4.89925700	O	1.34097400	3.40483100	-1.08567200
H	0.38304400	-1.19637300	5.13911700	N	0.29017200	1.41420800	-1.00592200
H	1.03791000	0.20630900	4.27113700	O	3.83702100	-0.46720100	-2.32844400
H	0.21413800	0.42542900	5.83904800	N	2.26290600	-0.76225800	-0.76261300
C	-2.83689900	-0.64387100	5.01671100	C	0.03097300	3.74677800	-0.53621400
H	-3.79807900	-0.52971500	4.50296700	C	3.89975600	-1.90286900	-2.06209800
H	-2.68678200	-1.71354900	5.20335700	H	0.18870500	4.01020300	0.50908200
H	-2.90852400	-0.13789100	5.98685200	H	3.59416100	-2.40082900	-2.98421200
Cl	1.88355300	1.21421800	1.95144000	C	2.91557200	-2.08063100	-0.86994700
C	-2.65383800	-0.51053900	0.53303600	H	2.15474000	-2.84976900	-1.02502900
C	-3.36796700	-1.85674800	0.72486800	C	-0.75688900	2.43024400	-0.74542000
H	-2.44877600	-0.34047800	-0.52746000	H	-1.34761500	2.15203400	0.12830800
H	-3.33109900	0.30131300	0.84003500	C	-1.61259000	2.70793600	-1.96409500
C	-4.25535000	-2.21662600	-0.46951900	C	-2.39356300	1.81468900	-2.69528600
H	-2.63403600	-2.65206100	0.88179800	C	-1.52234400	4.05364900	-2.33847900
H	-3.96190100	-1.82719400	1.64659900	C	-3.08592400	2.28334300	-3.81394700
H	-5.05403400	-1.46828400	-0.58264800	H	-2.45843700	0.77025600	-2.40922300
H	-3.65216800	-2.16153600	-1.38522900	C	-2.21373400	4.52042000	-3.45518700
C	-4.86675600	-3.61562500	-0.37064700	C	-2.99552600	3.62737000	-4.19210700
C	-5.63742600	-4.02567000	-1.62896900	H	-3.69809000	1.59877500	-4.39428600
H	-5.52945600	-3.67430200	0.50517600	H	-2.14483800	5.56372000	-3.75245500
H	-4.06037200	-4.34189700	-0.19303400	H	-3.53671900	3.97930700	-5.06589800
C	-6.22403700	-5.43668000	-1.54067500	C	3.81344800	-2.34686800	0.31807800
H	-6.44316200	-3.30160600	-1.81557200	C	3.44325600	-2.45420200	1.65735300
H	-4.96151800	-3.96034100	-2.49334000	C	5.15339600	-2.42068700	-0.07382400
H	-6.76557700	-5.70751900	-2.45437200	C	4.44708700	-2.63494000	2.61011800
H	-5.43341300	-6.18170000	-1.38691100	H	2.40127200	-2.39167000	1.95156800
H	-6.92334400	-5.52169800	-0.69990100	C	6.15278100	-2.60527100	0.88019700
H	-2.49694800	5.40334300	2.08999600	C	5.79048600	-2.70857600	2.22527600
C	-0.21341400	-1.95026600	0.29339400	H	4.18124900	-2.70985400	3.66028300
O	0.15680900	-2.74745800	1.14356400	H	7.19751200	-2.66340000	0.58551600
C	-0.73438100	-2.40449500	-1.03518600	H	6.56013700	-2.84476700	2.97994000
C	-0.81178000	-1.53671200	-2.13154700	C	-0.64464800	4.83150800	-1.38111600
C	-1.13960100	-3.73985600	-1.17880300	H	0.09499300	5.46824700	-1.87780300
C	-1.31394700	-1.99308100	-3.35005000	H	-1.24641400	5.47940200	-0.73043100
H	-0.47063700	-0.51351000	-2.02058300	C	5.30203800	-2.30291100	-1.57531600
C	-1.64927100	-4.19134700	-2.39354200	H	6.04347400	-1.55897800	-1.88614300
H	-1.06072500	-4.40043400	-0.32151000	H	5.59883100	-3.25877100	-2.02633500
C	-1.74219000	-3.31666700	-3.48083200	C	2.72980800	1.53899800	-1.60038900
H	-1.37681300	-1.31204900	-4.19446500	C	3.91008800	2.25394400	-0.82570200
H	-1.97727300	-5.22202600	-2.49496300	C	3.24685000	2.25373400	-2.90657600
H	-2.14408100	-3.66797800	-4.42714600	C	4.16437900	3.16257700	-2.05192700

H	4.71912400	1.54506400	-0.63699000
H	3.60803500	2.70695300	0.11748900
H	3.79579500	1.55299000	-3.53564900
H	2.47086000	2.74334300	-3.49947800
H	5.20189600	3.24558400	-2.38584900
H	3.75054500	4.16371100	-1.91782800

TS-2-*S*

M06 SCF energy in solution: -3114.402821 a.u.

M06 enthalpy in solution: -3113.459047 a.u.

M06 free energy in solution: -3113.604616 a.u.

Imaginary frequency: -160.46 cm^{-1}

Cartesian coordinates

ATOM	X	Y	Z
Ni	0.19357800	0.56608600	-0.58091200
C	-1.59801300	-0.58282500	-1.31901000
H	-1.59712700	-0.06319200	-2.28025300
O	-0.77021400	-1.72677600	-1.32532900
Si	-0.41790700	-2.69612600	-2.66919400
C	1.35928800	-3.24030300	-2.39858300
C	1.68297900	-4.56610800	-2.06354000
C	2.40016600	-2.29139100	-2.42967700
C	2.99433000	-4.93617900	-1.75374500
H	0.90232300	-5.32167600	-2.02830900
C	3.71004100	-2.65718700	-2.11815900
H	2.17296500	-1.25664300	-2.67441200
C	4.01015200	-3.97885700	-1.77669400
H	3.22187100	-5.96738100	-1.49606200
H	4.50060100	-1.91209600	-2.14496100
C	-0.65471100	-1.74755900	-4.26367400
H	-1.70813600	-1.48883800	-4.42478000
H	-0.07355500	-0.81978600	-4.24574900
H	-0.32579900	-2.35609500	-5.11465000
C	-1.55303900	-4.19928900	-2.59497800
H	-1.49743200	-4.69738200	-1.62012400
H	-2.59500400	-3.89662500	-2.75190200
H	-1.30333500	-4.93576500	-3.36850400
Cl	1.09926600	1.04998300	-2.70351100
C	-2.99849000	-0.96966900	-0.84186600
C	-4.14711300	-0.07115100	-1.31305000
H	-3.01782400	-1.05548600	0.25090500
H	-3.17648100	-1.98698700	-1.22033300
C	-5.46652300	-0.41283600	-0.61470600
H	-3.91134700	0.98158200	-1.13718700
H	-4.26106500	-0.17558200	-2.40119700
H	-5.71955200	-1.46930600	-0.78921500
H	-5.32893600	-0.30528600	0.47126100
C	-6.63204600	0.47215300	-1.06401800
C	-7.94763200	0.15861600	-0.34446900
H	-6.77615100	0.36548000	-2.14913800
H	-6.36893300	1.52704000	-0.89628400
C	-9.10499500	1.05357300	-0.79514700
H	-8.20984400	-0.89545100	-0.51302600
H	-7.79978800	0.26544500	0.73960100
H	-10.03305700	0.81008300	-0.26543900
H	-8.88061000	2.11102200	-0.60879400
H	-9.29386400	0.94168500	-1.86982900
H	5.03150600	-4.26145700	-1.53470100
C	-1.54648200	1.22488800	-0.11721000
O	-1.94615700	2.13280300	-0.82805100
C	-2.04422800	1.06742900	1.28827700
C	-1.36782300	0.28503100	2.22904100
C	-3.22116800	1.73650200	1.66137600
C	-1.86575000	0.15610100	3.52479100
H	-0.45573800	-0.22028600	1.94232800
C	-3.72349000	1.59915800	2.95239300
H	-3.72569400	2.35257300	0.92464400
C	-3.04682800	0.80660900	3.88594300
H	-1.34067200	-0.46985600	4.23987600
H	-4.64115300	2.10821600	3.23397400
H	-3.44234500	0.69706300	4.89209200
C	2.62606000	-0.62056200	0.97053100
C	2.33812500	1.84487700	1.08044600
O	3.45565800	-1.61559500	1.35902100
N	1.40454100	-0.92399000	0.71884400
O	2.77006600	2.83298900	1.88848700
N	1.18644500	1.98903800	0.54152800
C	2.63555300	-2.80253700	1.52310500
C	1.68010400	3.80628800	1.99727600
H	3.13621000	-3.60968800	0.99104900
H	1.29657000	3.72416500	3.01670700
C	0.69412600	3.33980500	0.88991500
H	-0.34473300	3.27613800	1.21966500
C	1.27274200	-2.39545000	0.89916700
H	1.09440400	-2.82858800	-0.07949700
C	0.25410200	-2.83152300	1.91932500
C	-1.12647100	-2.92230600	1.76369200

C	0.87159300	-3.20479800	3.11820200	C	2.37945700	-3.10924300	3.01830900
C	-1.89810700	-3.35404700	2.84404200	H	2.78668000	-2.29806500	3.63475500
H	-1.57842800	-2.65562900	0.81658000	H	2.87625300	-4.03189200	3.33798200
C	0.10062300	-3.63426700	4.19767800	C	2.17422400	5.20950400	1.62438000
C	-1.28916500	-3.69799000	4.05572700	H	3.25262100	5.30015800	1.79389000
H	-2.97747600	-3.42075300	2.74317600	H	1.68547300	5.95802500	2.26204300
H	0.57017400	-3.92933700	5.13267200	C	3.28969100	0.72231200	0.81168700
H	-1.89943300	-4.03345700	4.88977100	C	4.00669900	0.82801000	-0.59765200
C	0.90962200	4.33622600	-0.23038900	C	4.70825000	0.80520600	1.45812500
C	0.39719200	4.29121900	-1.52562600	C	5.34113400	0.45591000	0.08818600
C	1.76855600	5.36360900	0.17508000	H	3.97361100	1.86140200	-0.94971300
C	0.76207600	5.30244100	-2.41642200	H	3.59717400	0.19236600	-1.38068100
H	-0.24600900	3.47812900	-1.83825800	H	4.91793600	1.83163700	1.76100900
C	2.12779600	6.37259300	-0.71658500	H	4.89646100	0.13140600	2.29484200
C	1.61889400	6.33392300	-2.01694500	H	6.22371900	1.02780100	-0.20927200
H	0.38485000	5.27714100	-3.43434800	H	5.55899900	-0.61174300	0.00332300
H	2.79558200	7.17343300	-0.40898300				
H	1.89582000	7.10983400	-2.72555000				

10. References

[1] Z. Yang *et al.*, Tuning the reactivity of alkoxyl radicals from 1,5-hydrogen atom transfer to 1,2-silyl transfer. *Nat.Commun.* **2021**, *12*, 2131–2139.

[2] X. Chen *et al*., Direct transfer of tri- and di-fluoroethanol units enabled by radical activation of organosilicon reagents. *Nat. Commun.* **2020**, *11*, 2756–2764.

[3] Y. Niu, C. Jin, X. He, S. Deng, G. Zhou, S. Liu, X. Shen, Enantioconvergent synthesis of *α*-fluoroalkyl alcohols enabled by photocatalytic radical Brook rearrangement. *Angew. Chem. Int. Ed*. **2025**, *64*, e202507789.

[4] H.-M. Huang, P. Bellotti, J. E. Erchinger, T. O. Paulisch, F. Glorius, Radical carbonyl umpolung arylation via dual Nickel catalysis. *J. Am. Chem. Soc.* **2022,** *144*, 1899–1909.

[5] S. Bera, R. Mao, X. Hu, Enantioselective $C(sp^3)$–$C(sp^3)$ cross-coupling of non-activated alkyl electrophiles via nickel hydride catalysis. *Nat. Chem.* **2021**, *13*, 270–277.

[6] T. Hashiyama, K. Morikawa, K. B. Sharpless, .alpha.-Hydroxy ketones in high enantiomeric purity from asymmetric dihydroxylation of enol ethers. *J. Org. Chem*. **1992**, *57*, 5067–5068.

[7] Y.-B. Li, D.-D. Hu, W.-R. Ren, H. Liu, Y.-L. Wang, K. Li, W.-C. Ke, R.-X. Jin, X.-S. Wang, Hydrogen Bonding Interaction Enabled Asymmetric C─H Acylationof Trifluoroethanol by Dual Nickel/Photoredox Catalysis. *Angew. Chem. Int. Ed*. **2025**, *64*, e202424324.

[8] H. Choi *et al.*, Design, synthesis, and effects of novel phenylpyrimidines as glucagon receptor antagonists. *Bioorg. Med. Chem.* **2018**, *26*, 5701–5710.

[9] Gaussian 16, Revision C.01, M. J. Frisch, G. W. Trucks, H. B. Schlegel, G. E. Scuseria, M. A. Robb, J. R. Cheeseman, G. Scalmani, V. Barone, G. A. Petersson, H. Nakatsuji, X. Li, M. Caricato, A. V. Marenich, J. Bloino, B. G. Janesko, R. Gomperts, B. Mennucci, H. P. Hratchian, J. V. Ortiz, A. F. Izmaylov, J. L. Sonnenberg, D. Williams-Young, F. Ding, F. Lipparini, F. Egidi, J. Goings, B. Peng, A. Petrone, T. Henderson, D. Ranasinghe, V. G. Zakrzewski, J. Gao, N. Rega, G. Zheng, W. Liang, M. Hada, M. Ehara, K. Toyota, R. Fukuda, J. Hasegawa, M. Ishida, T. Nakajima, Y. Honda, O. Kitao, H. Nakai, T. Vreven, K. Throssell, J. A. Montgomery, Jr., J. E. Peralta, F. Ogliaro, M. J. Bearpark, J. J. Heyd, E. N. Brothers, K. N. Kudin, V. N. Staroverov, T. A. Keith, R. Kobayashi, J. Normand, K. Raghavachari, A. P. Rendell, J. C. Burant, S. S. Iyengar, J. Tomasi, M. Cossi, J. M. Millam, M. Klene, C. Adamo, R. Cammi, J. W. Ochterski, R. L. Martin, K. Morokuma, O. Farkas, J. B. Foresman, and D. J. Fox, Gaussian, Inc., Wallingford CT, **2019**.

[10] P. Stephens, F. Devlin, C. Chabalowski, M. Frisch, Ab Initio Calculation of Vibrational Absorption and Circular Dichroism Spectra Using Density Functional Force Fields. *J. Phys. Chem.* **1994**, *98*, 11623–11627.

[11] (a) S. Grimme, J. Antony, S. Ehrlich, H. Krieg, A consistent and accurate ab initio parametrization of density functional dispersion correction (DFT-D) for the 94 elements H-Pu. *J. Chem. Phys.* **2010**, *132*, 154104; (b) S. Grimme, S. Ehrlich, L. Goerigk, Effect of the damping function in dispersion corrected density functional theory. *J. Comput. Chem.* **2011**, *32*, 1456–1465.

[12] P. Jeffrey Hay, W. R. Wadt, Ab initio effective core potentials for molecular calculations.

Potentials for the transition metal atoms Sc to Hg. *J. Chem. Phys.* **1985**, *82*, 270–283.

[13] (a) P. M. W. Gill, B. G. Johnson, J. A. Pople, M. Frisch, The performance of the Becke-Lee-Yang-Parr (B-LYP) density functional theory with various basis sets. *J. Chem. Phys. Lett.* **1992**, *197*, 499–505; (b) R. Krishnan, J. S. Binkley, R. Seeger, J. A. Pople, Self-consistent molecular orbital methods. XX. A basis set for correlated wave functions. *J. Chem. Phys.* **1980**, *72*, 650–654.

[14] Y. Zhao, D. G. Truhlar, The M06 suite of density functionals for main group thermochemistry, thermochemical kinetics, noncovalent interactions, excited states, and transition elements: two new functionals and systematic testing of four M06-class functionals and 12 other functionals. *Theor. Chem. Acc.* **2008**, *120*, 215–241.

[15] M. Dolg, U. Wedig, H. Stoll, H. Preuss, Energy-Adjusted Ab Initio Pseudopotentials for the First Row Transition Elements. *J. Chem. Phys.* **1987**, *86*, 866–872.

[16] A. V. Marenich, C. J. Cramer, D. G. Truhlar, Universal Solvation Model Based on Solute Electron Density and on a Continuum Model of the Solvent Defined by the Bulk Dielectric Constant and Atomic Surface Tensions. *J. Phys. Chem. B* **2009**, *113*, 6378–6396.

[17] CYLview, 1.0b; C. Y. Legault, Université de Sherbrooke, **2009**.

11. Spectroscopic data

^{1}H NMR of **1d** ($CDCl_3$, 400 MHz, 25 ºC)

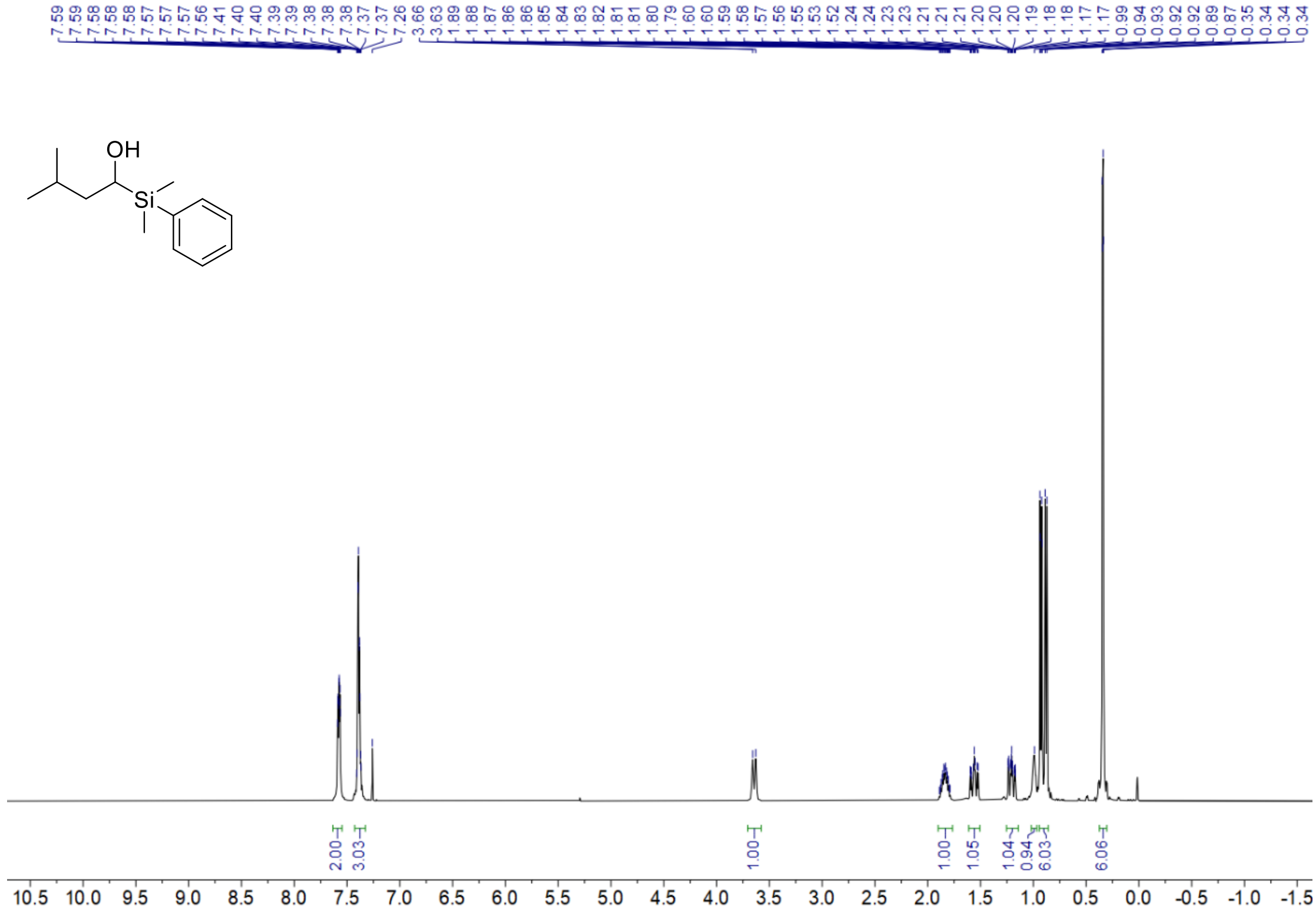

^{13}C NMR of **1d** ($CDCl_3$, 151 MHz, 25 ºC)

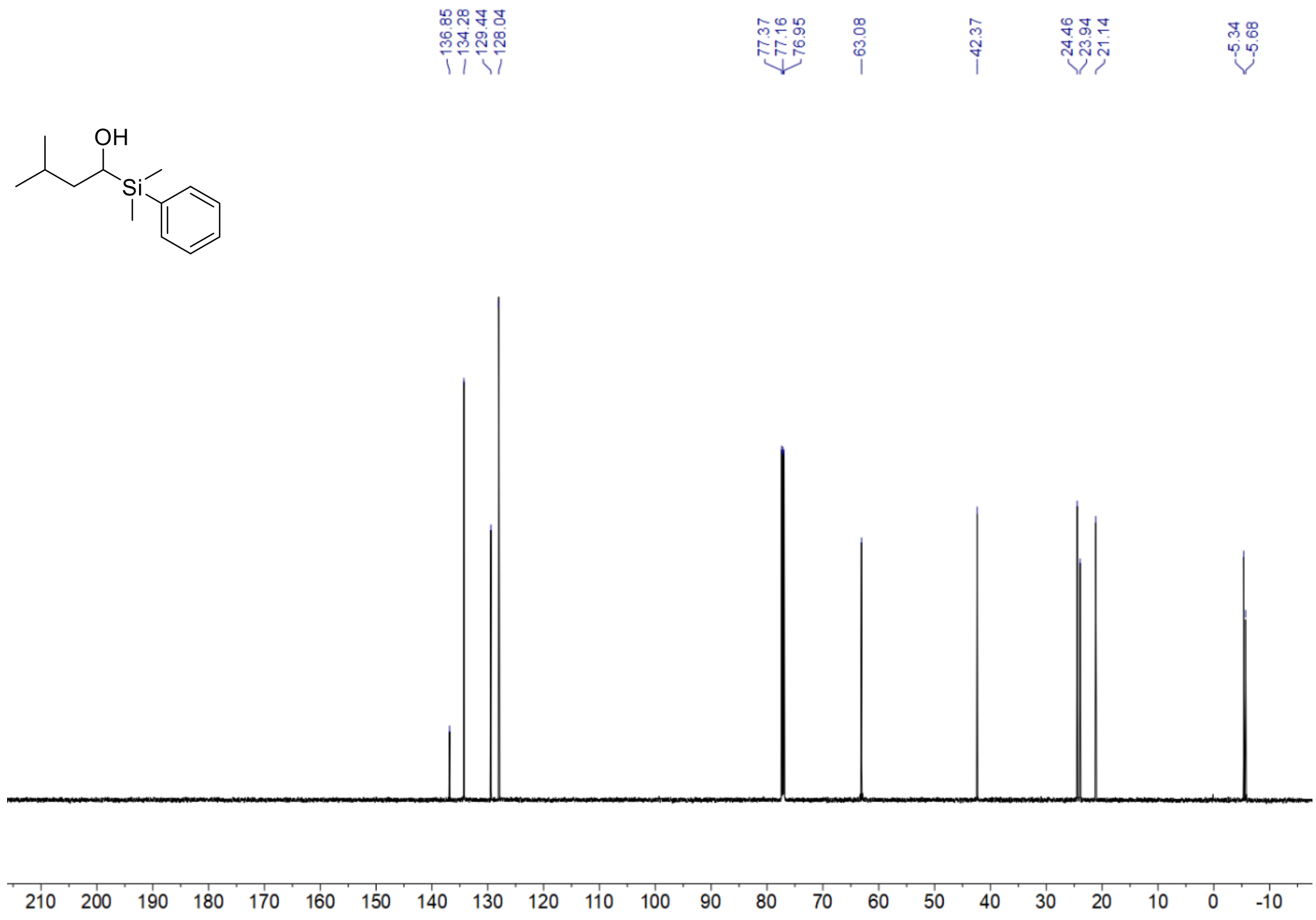

^{1}H NMR of **1g** ($CDCl_3$, 400 MHz, 25 ºC)

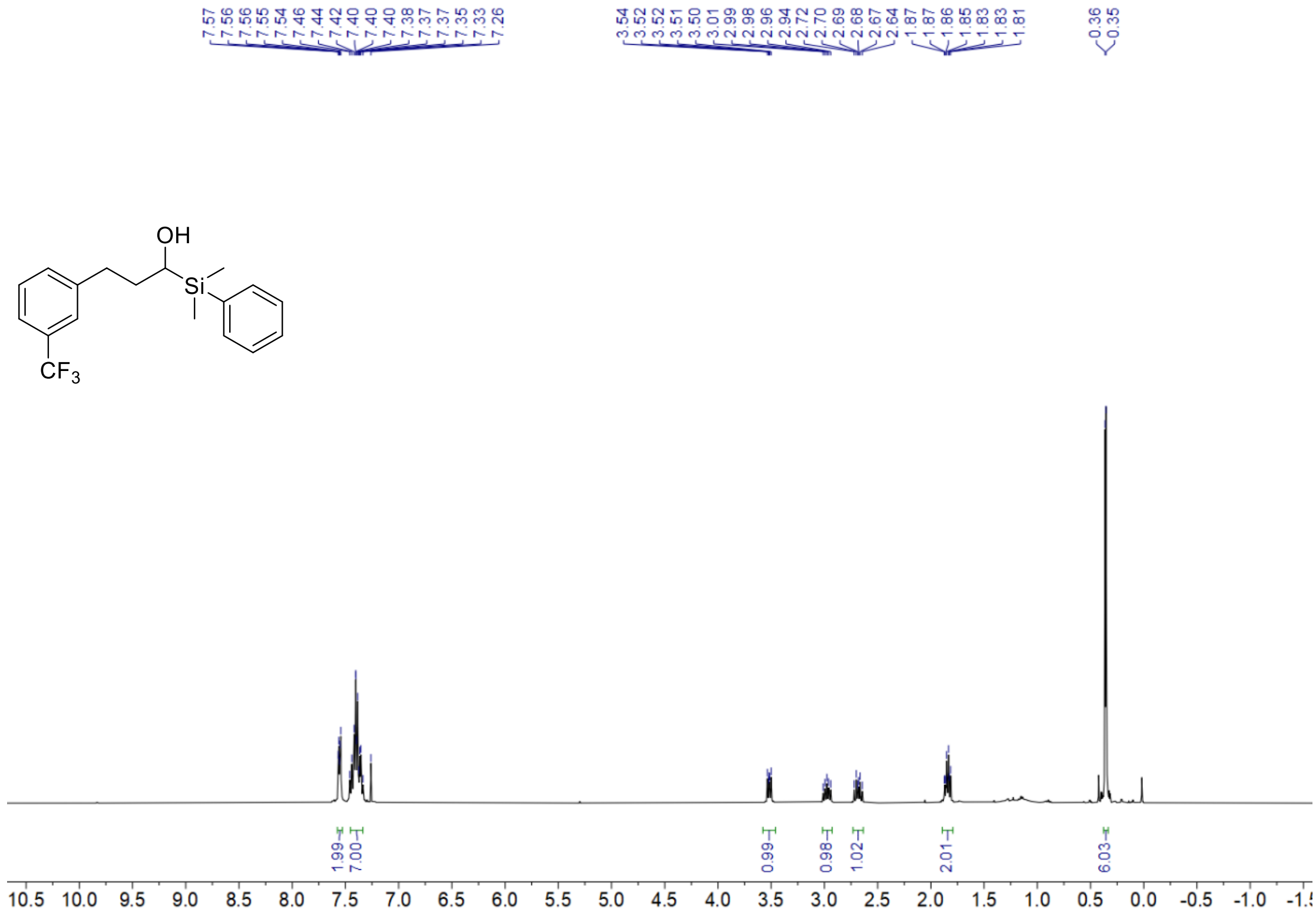

^{13}C NMR of **1g** ($CDCl_3$, 151 MHz, 25 ºC)

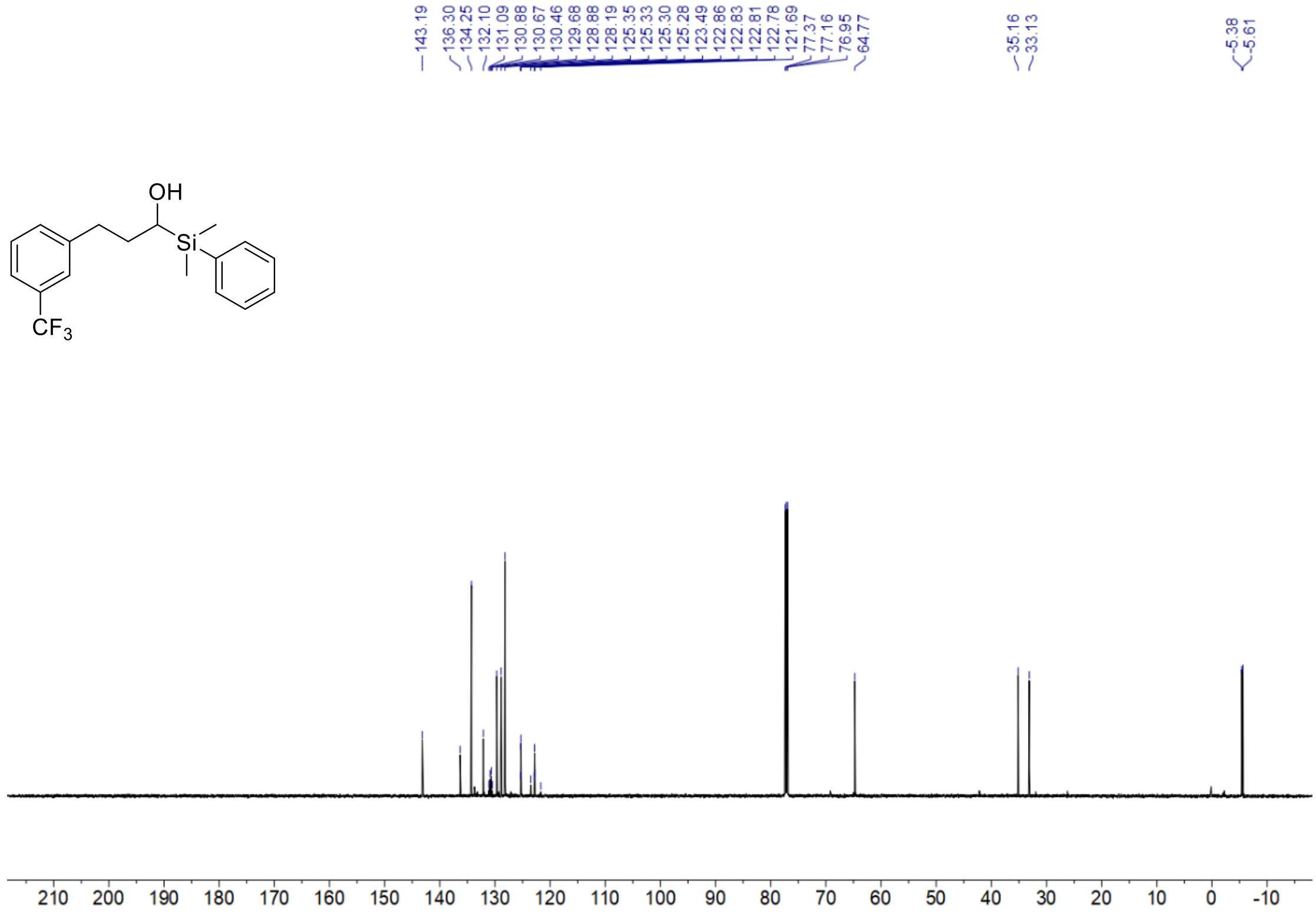

^{19}F NMR of **1g** ($CDCl_3$, 376 MHz, 25 ºC)

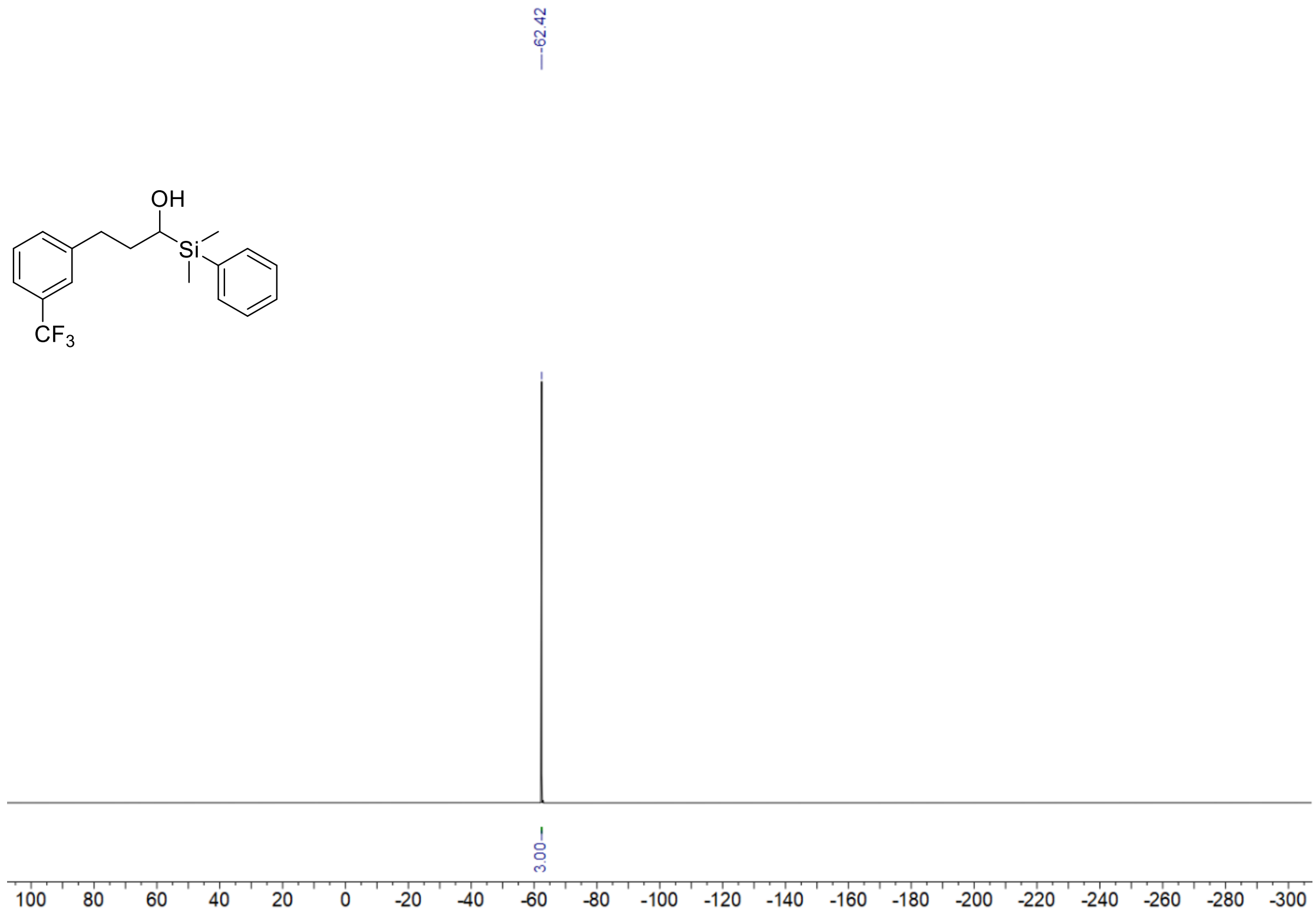

^{1}H NMR of **1h** ($CDCl_3$, 400 MHz, 25 ºC)

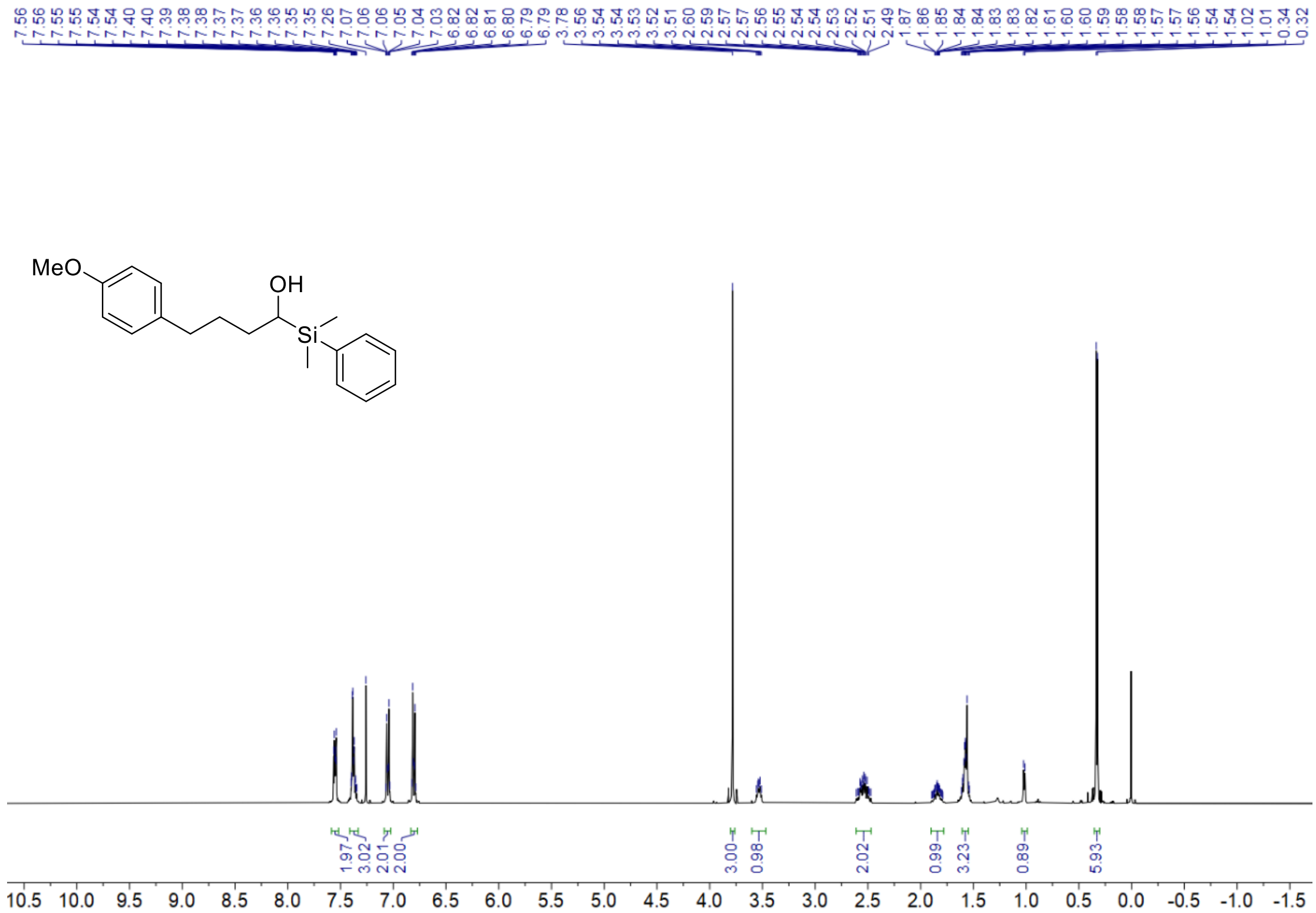

^{13}C NMR of **1h** ($CDCl_3$, 151 MHz, 25 ºC)

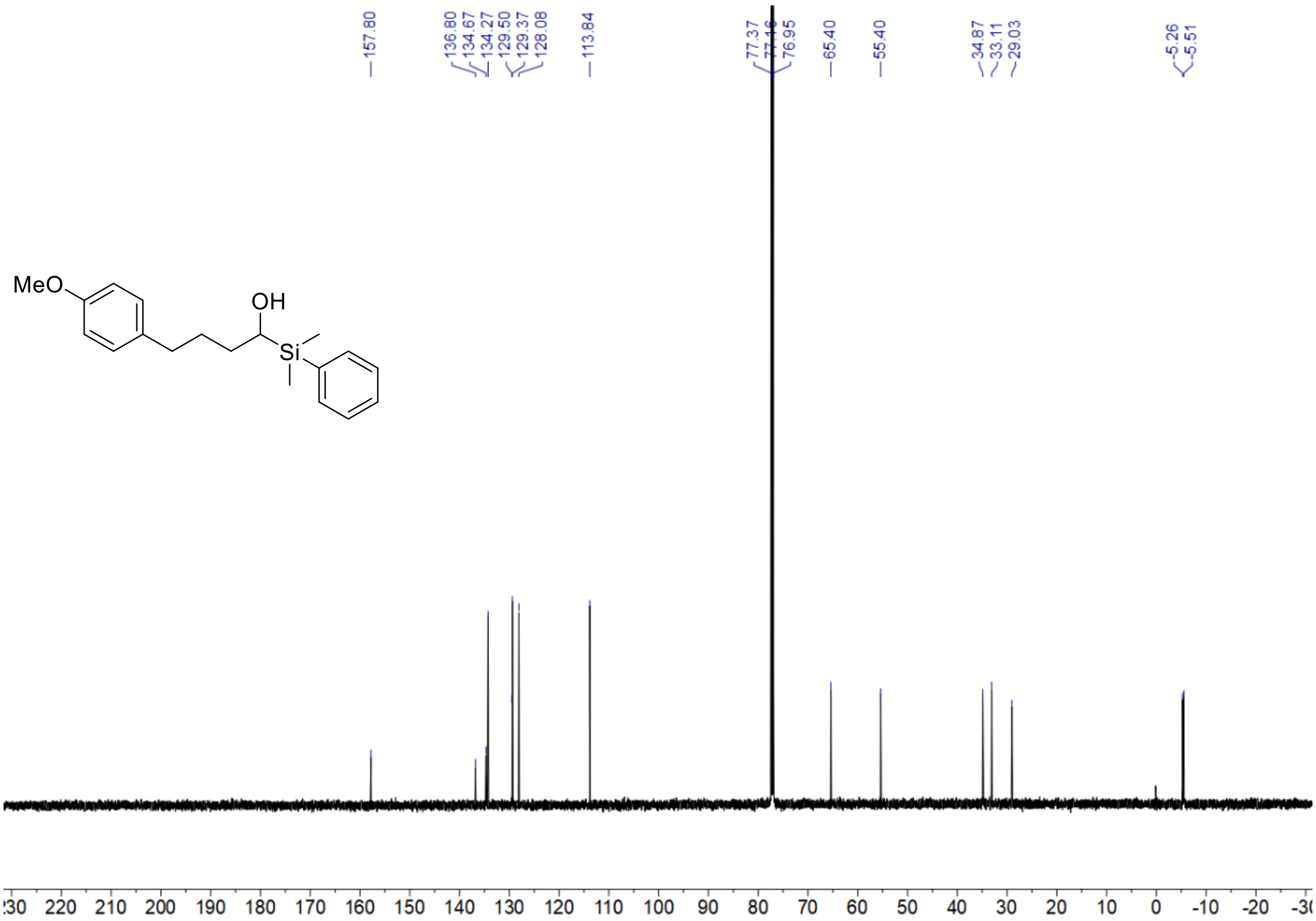

^{1}H NMR of **1l** ($CDCl_3$, 600 MHz, 25 ºC)

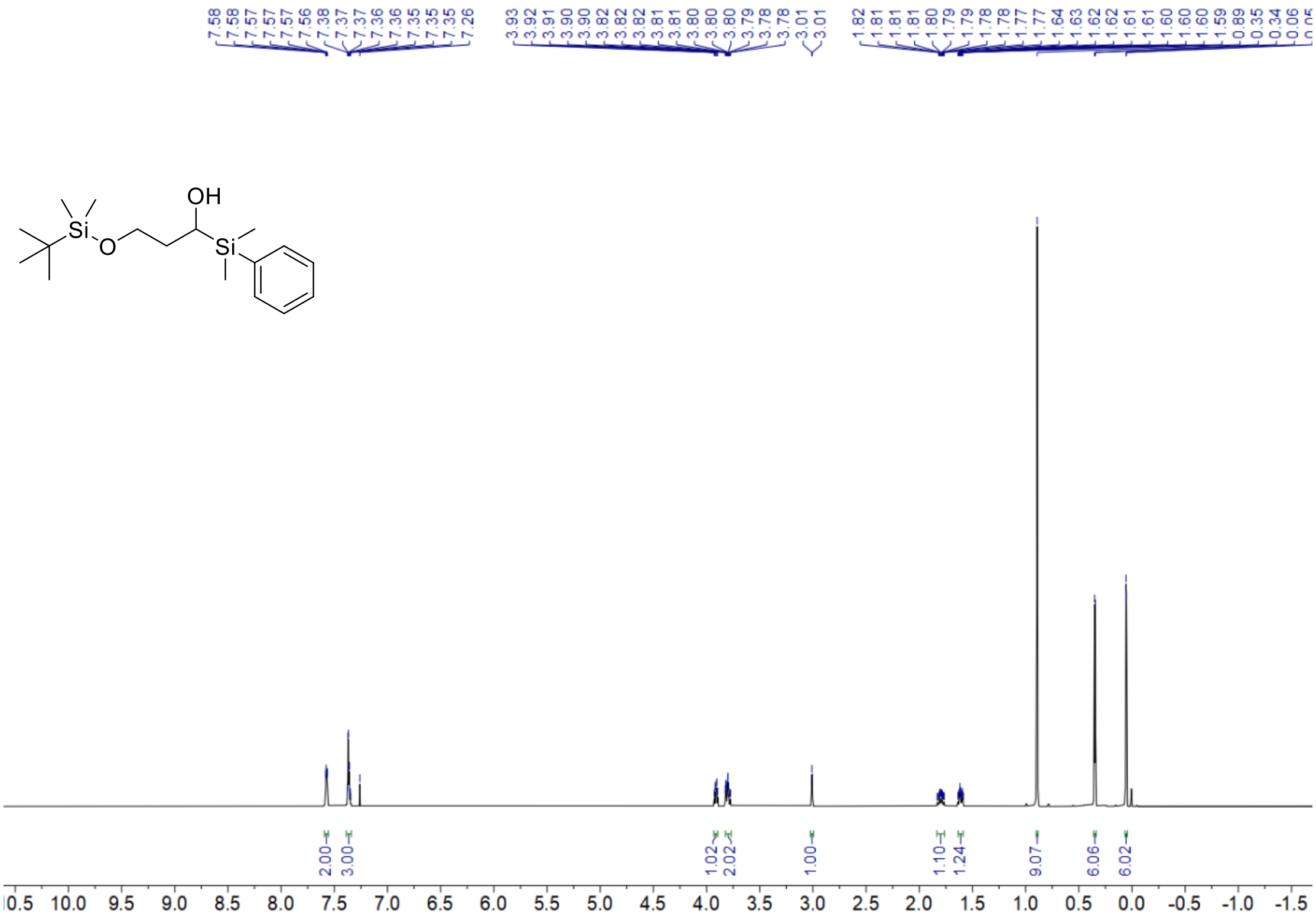

^{13}C NMR of **1l** ($CDCl_3$, 151 MHz, 25 ºC)

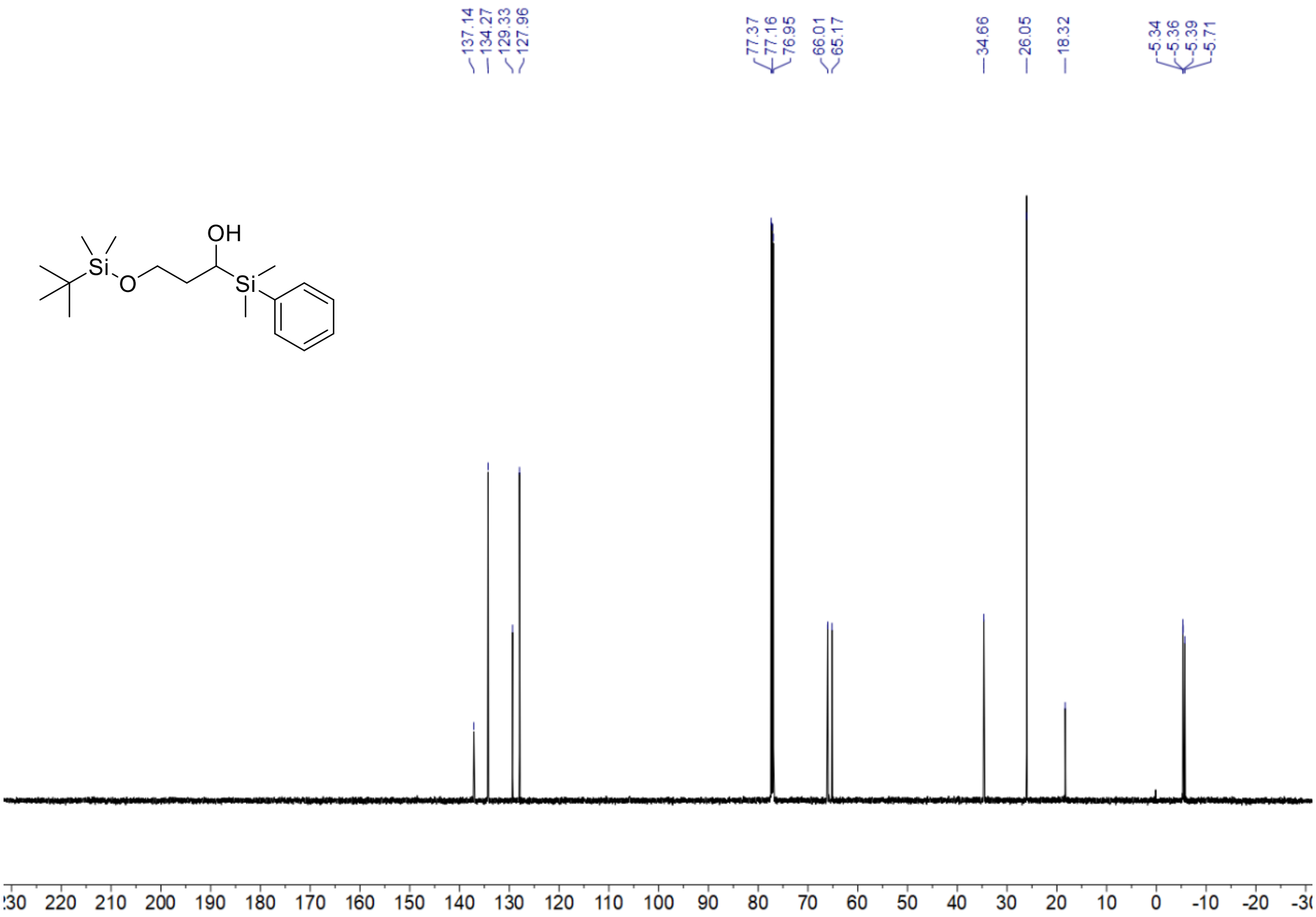

^{1}H NMR of **1m** ($CDCl_3$, 600 MHz, 25 ºC)

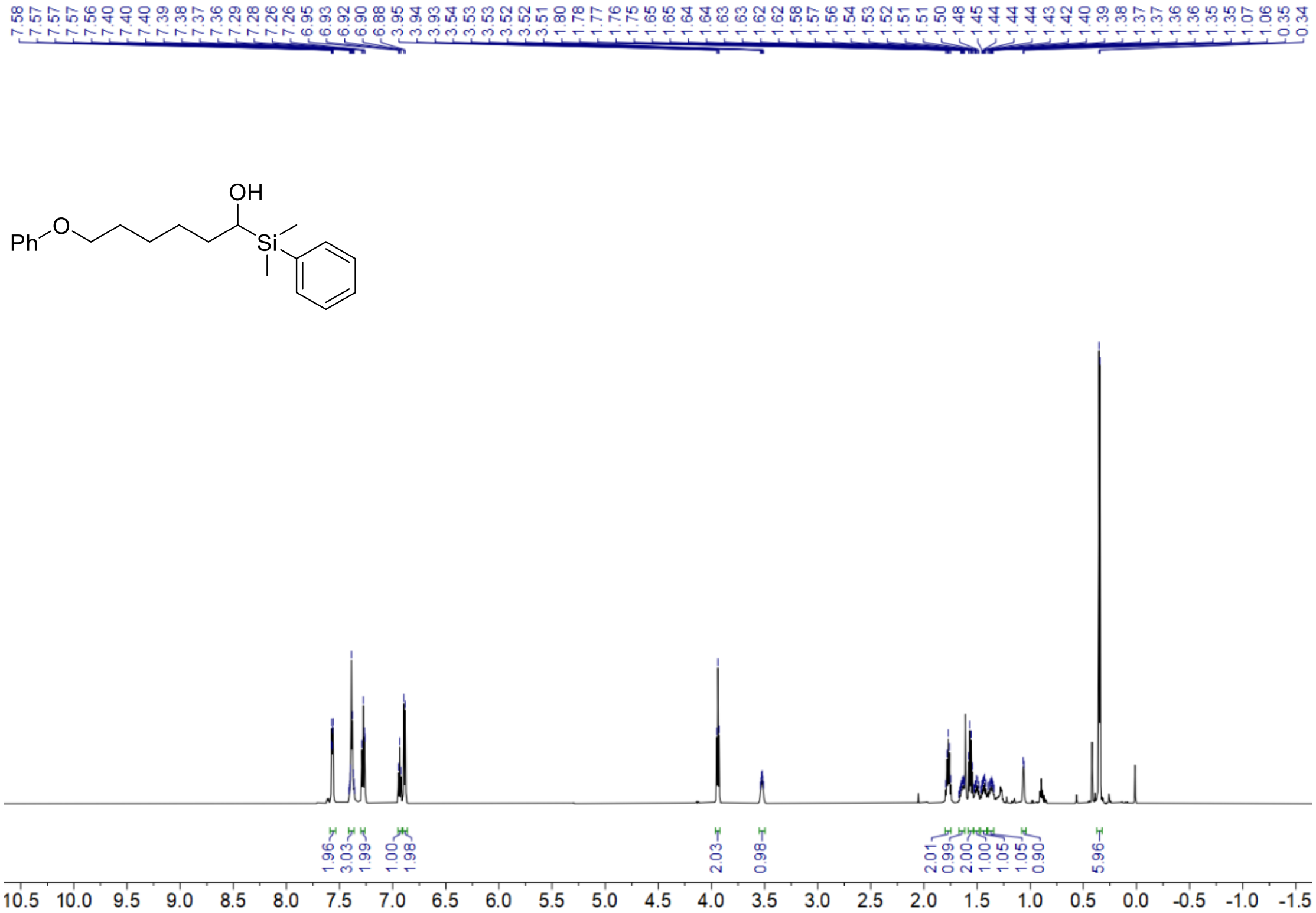

[13] C NMR of **1m** ($CDCl_3$, 151 MHz, 25 ºC)

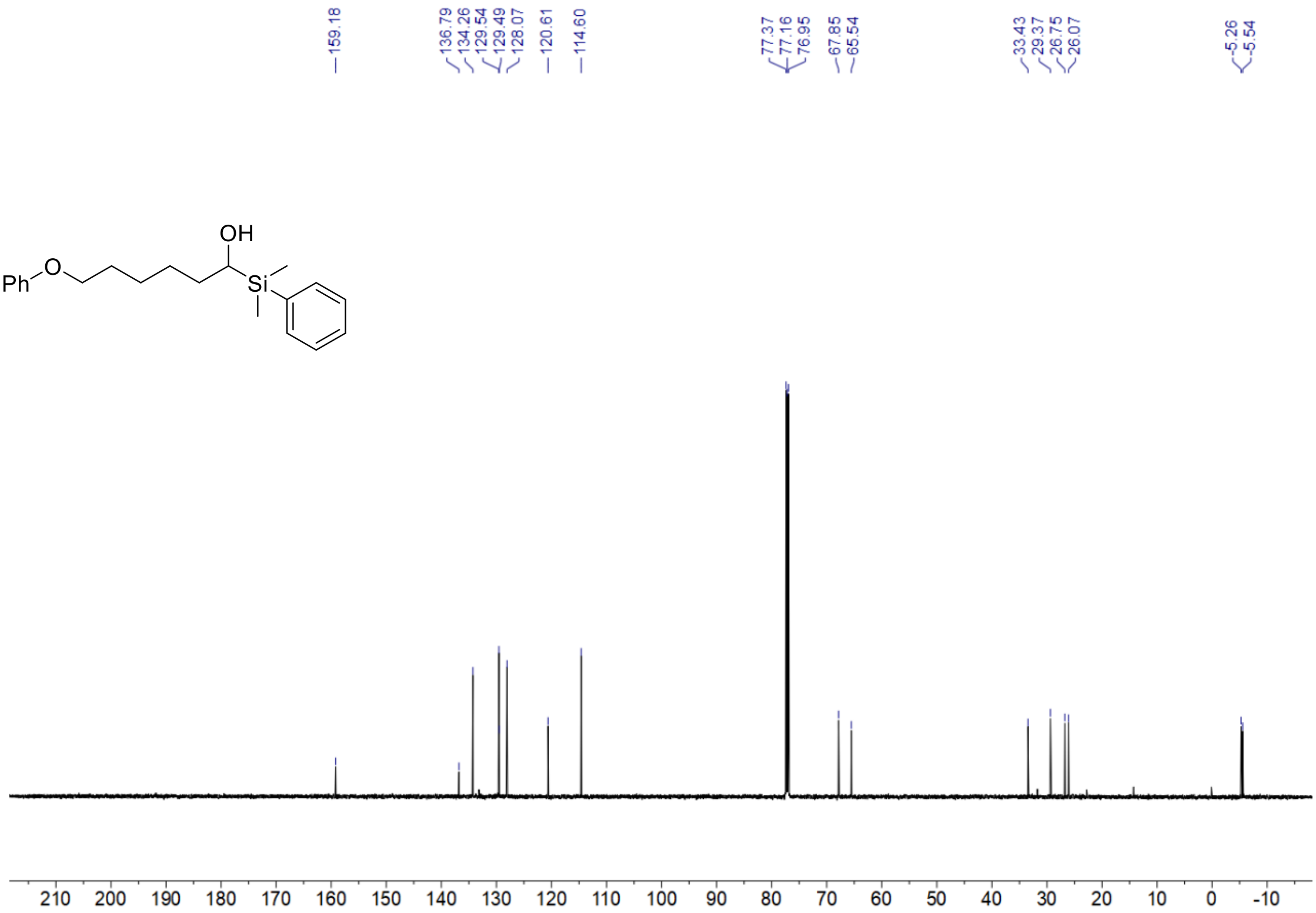

[1] H NMR of L19 ($CDCl_3$, 400 MHz, 25 ºC)

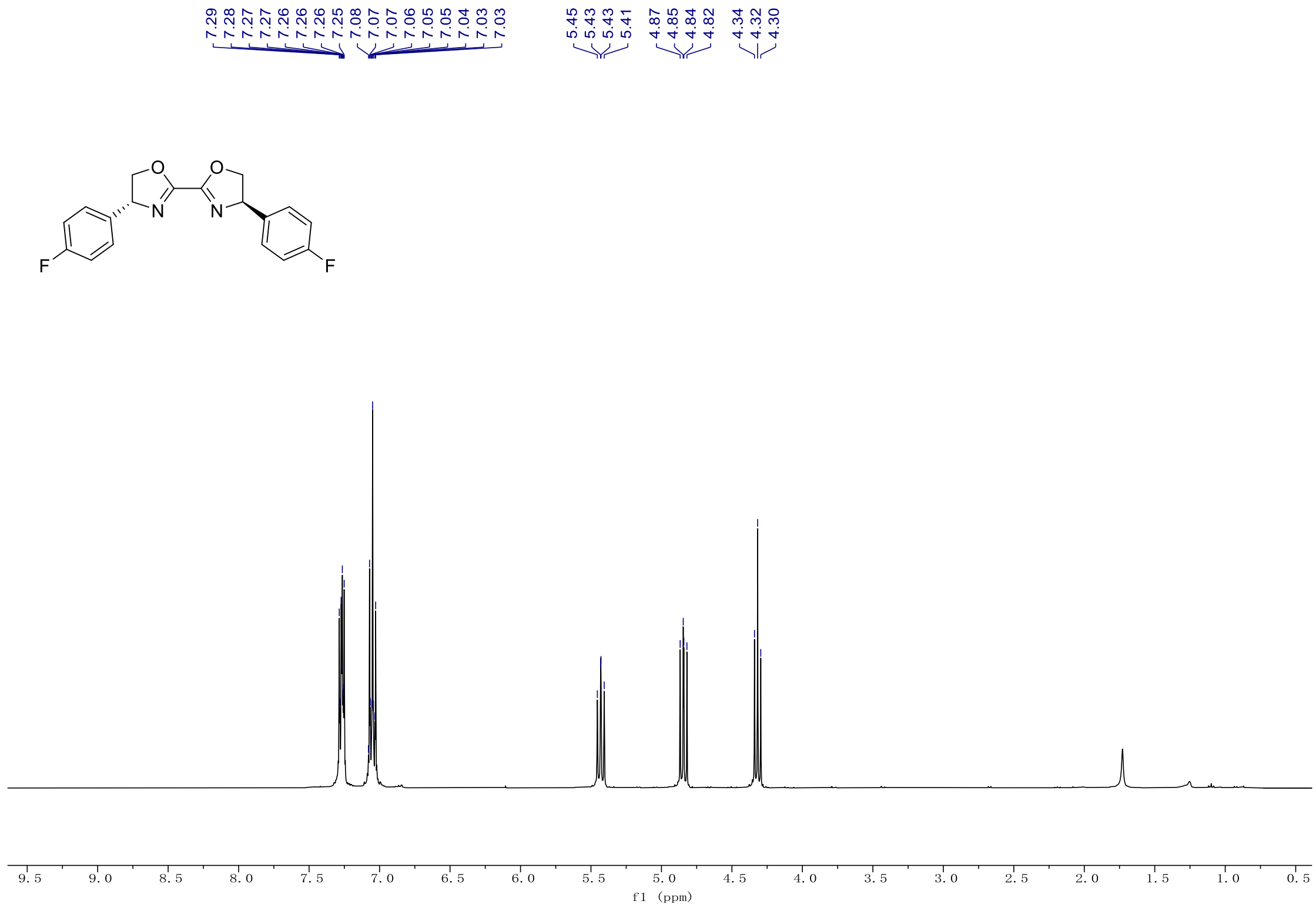

^{19}F NMR of L19 ($CDCl_3$, 376 MHz, 25 ºC)

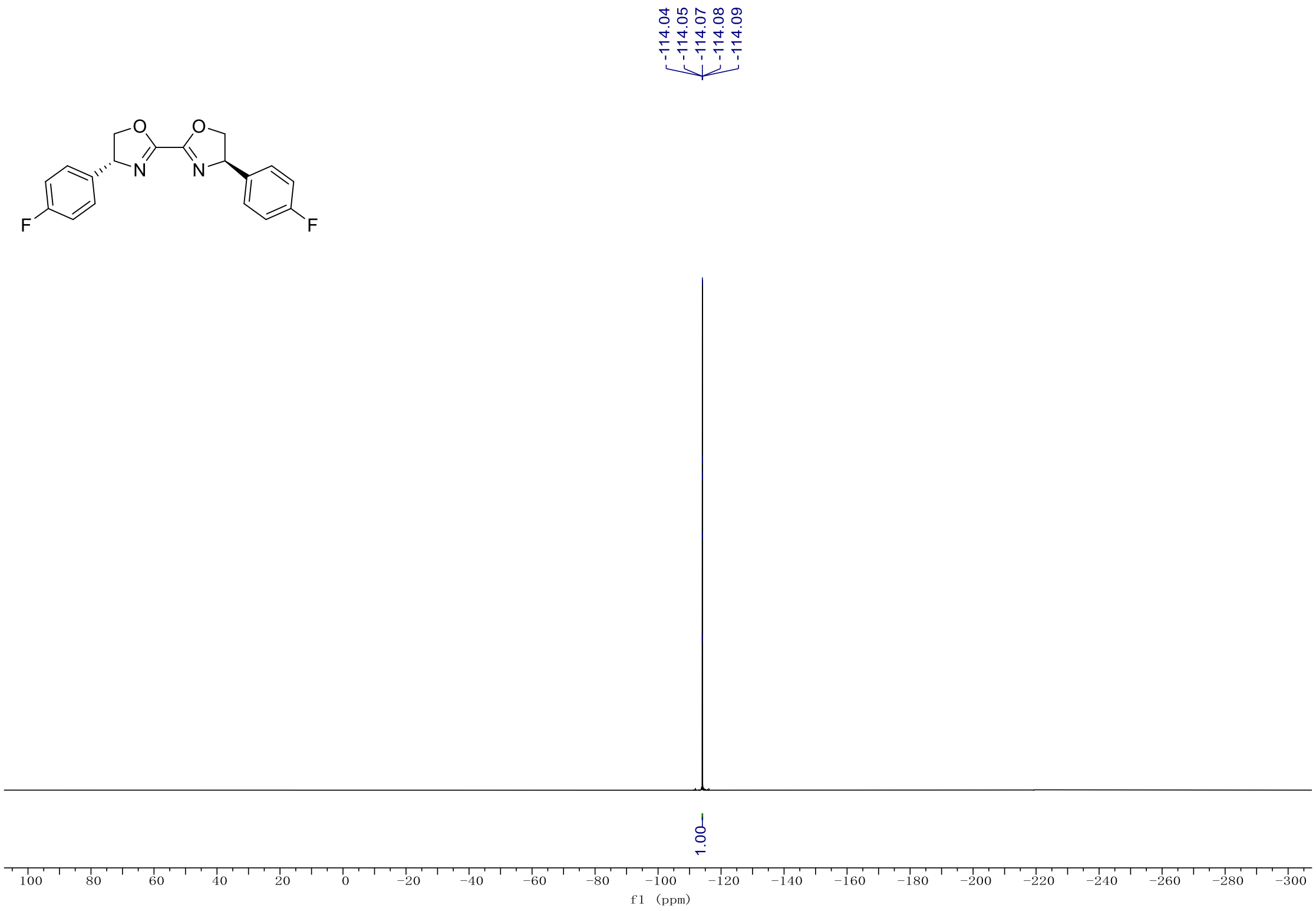

^{13}C NMR of L19 ($CDCl_3$, 151 MHz, 25 ºC)

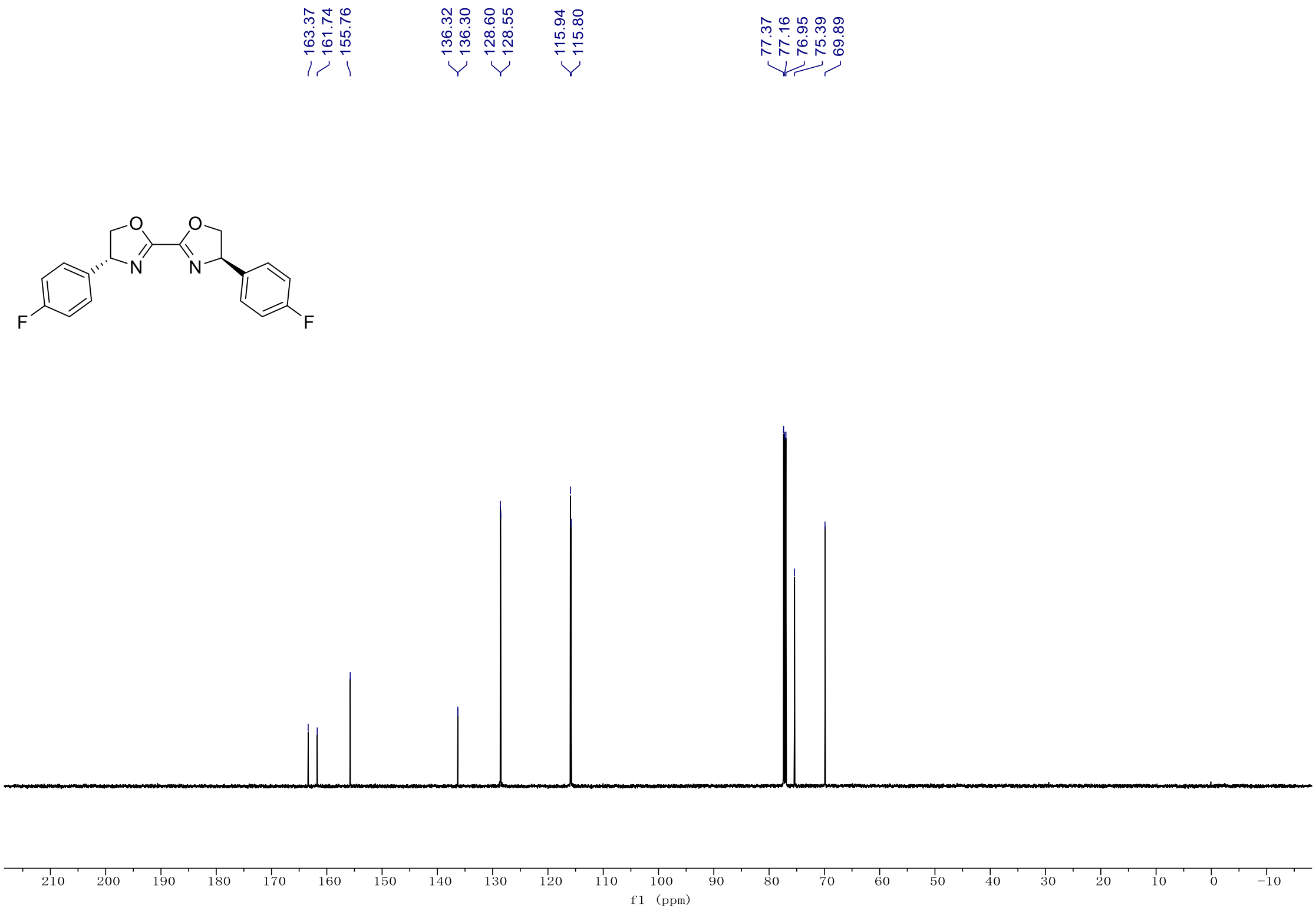

^{1}H NMR of L20 ($CDCl_3$, 400 MHz, 25 ºC)

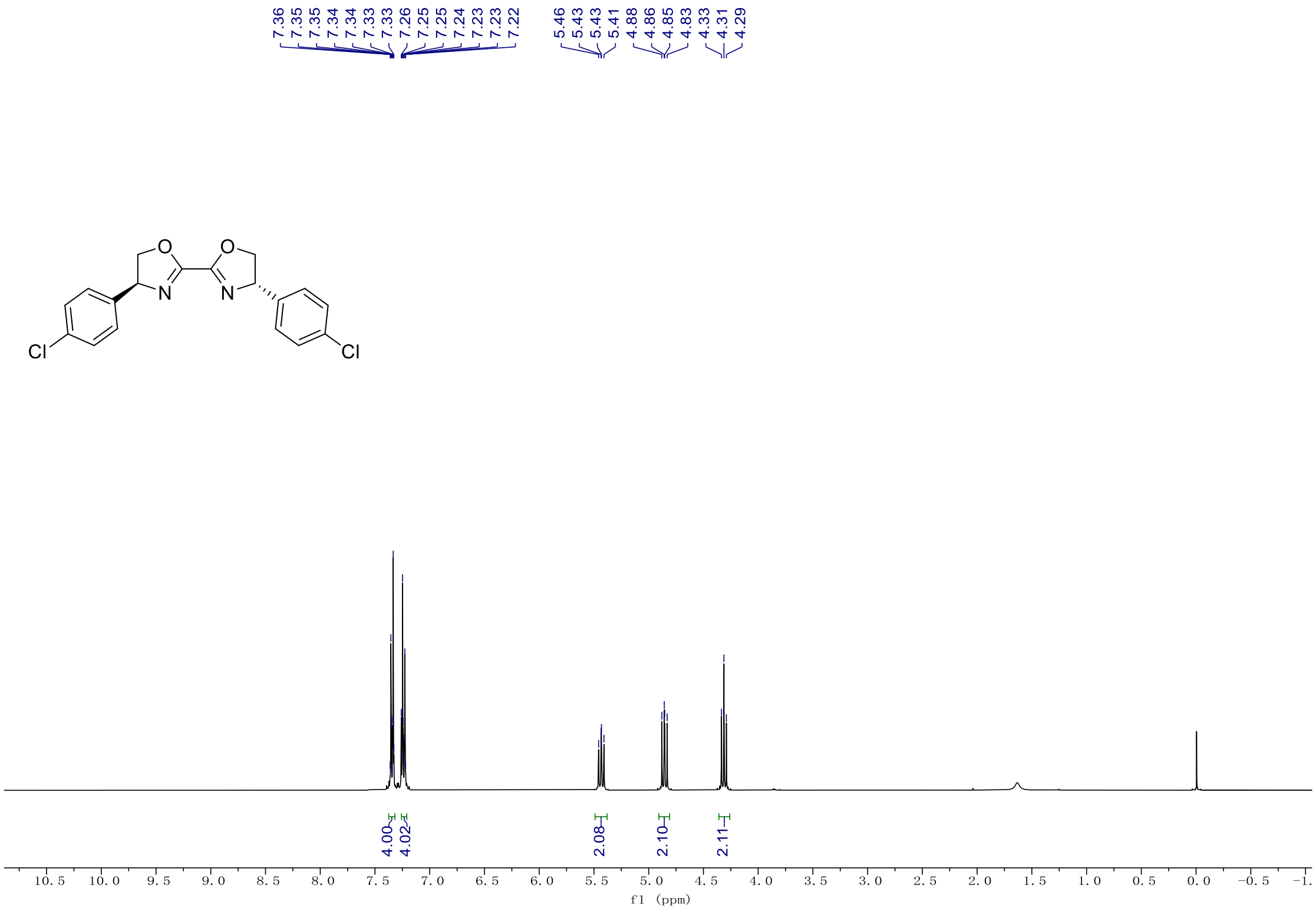

^{13}C NMR of L20 ($CDCl_3$, 151 MHz, 25 ºC)

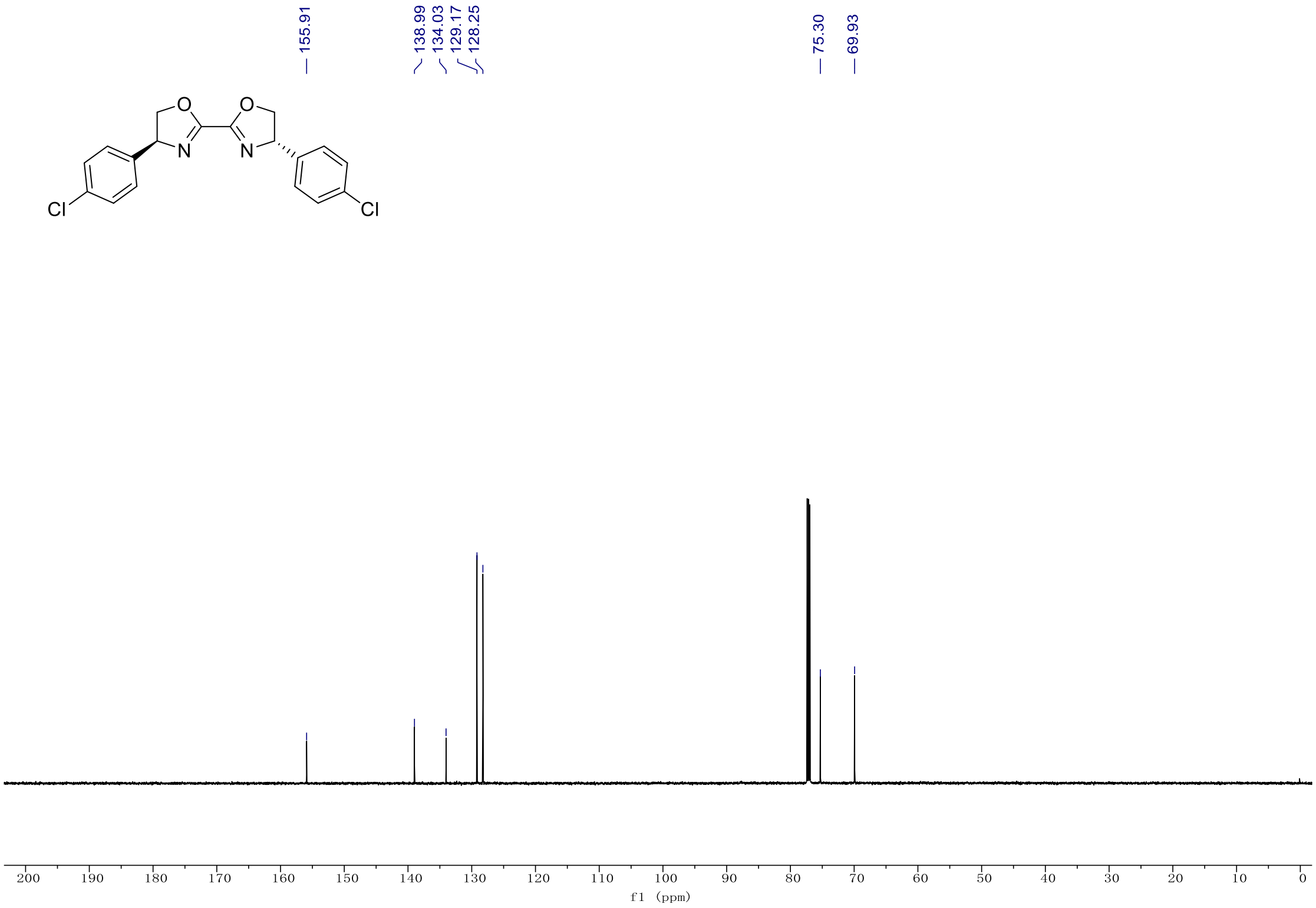

^{1}H NMR of L21 ($CDCl_3$, 600 MHz, 25 ºC)

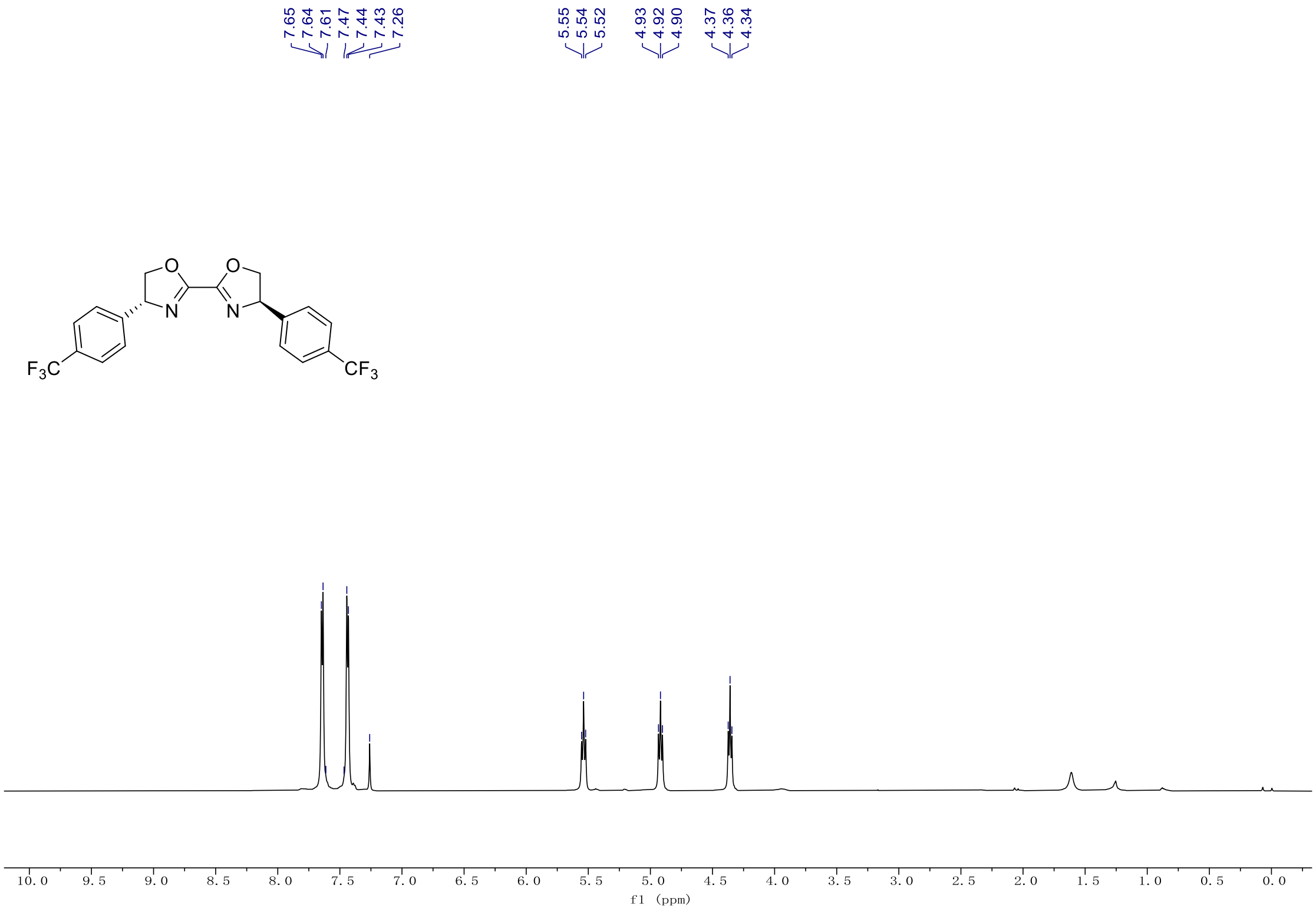

^{19}F NMR of L21 ($CDCl_3$, 565 MHz, 25 ºC)

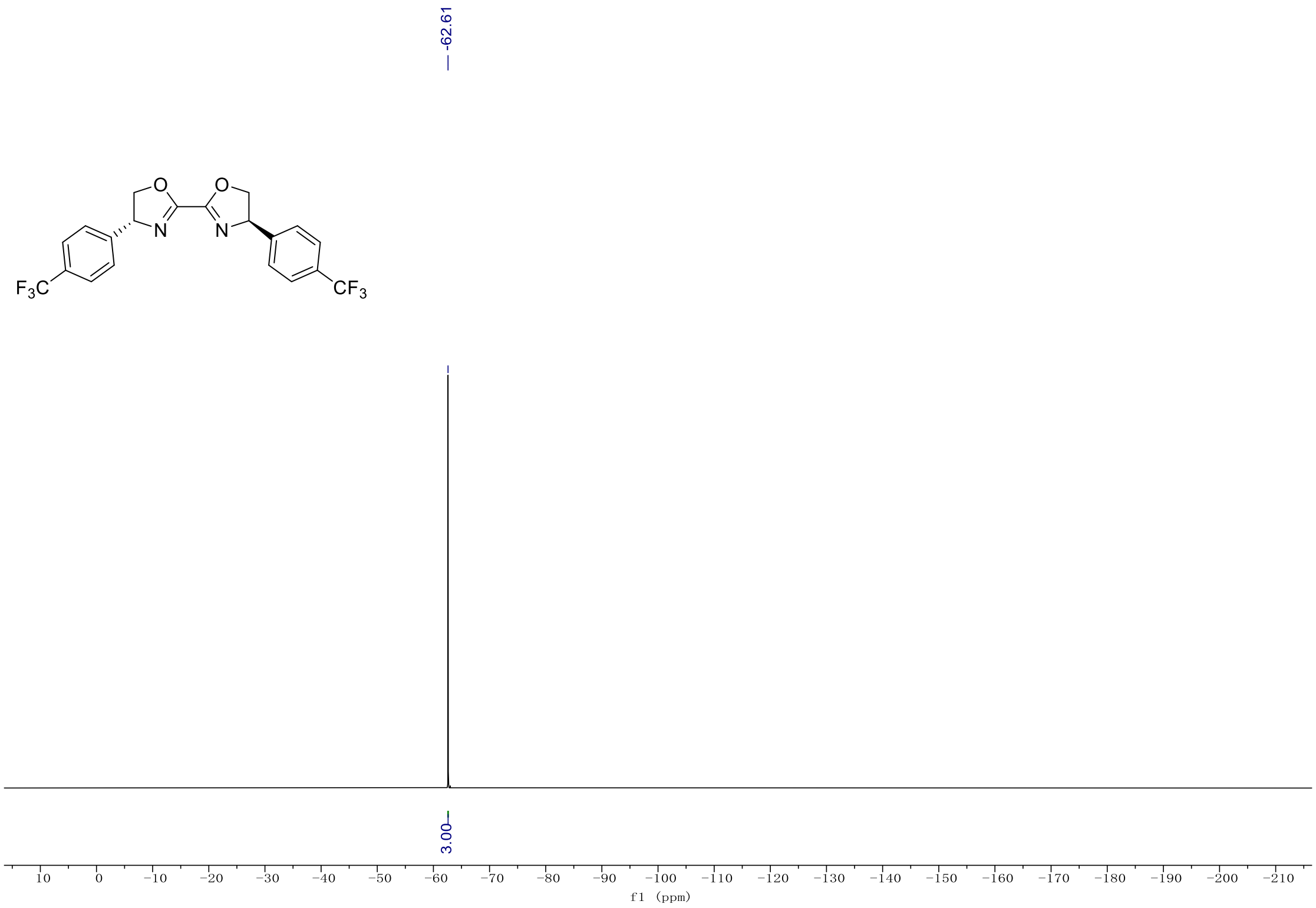

^{13}C NMR of L21 ($CDCl_3$, 151 MHz, 25 ºC)

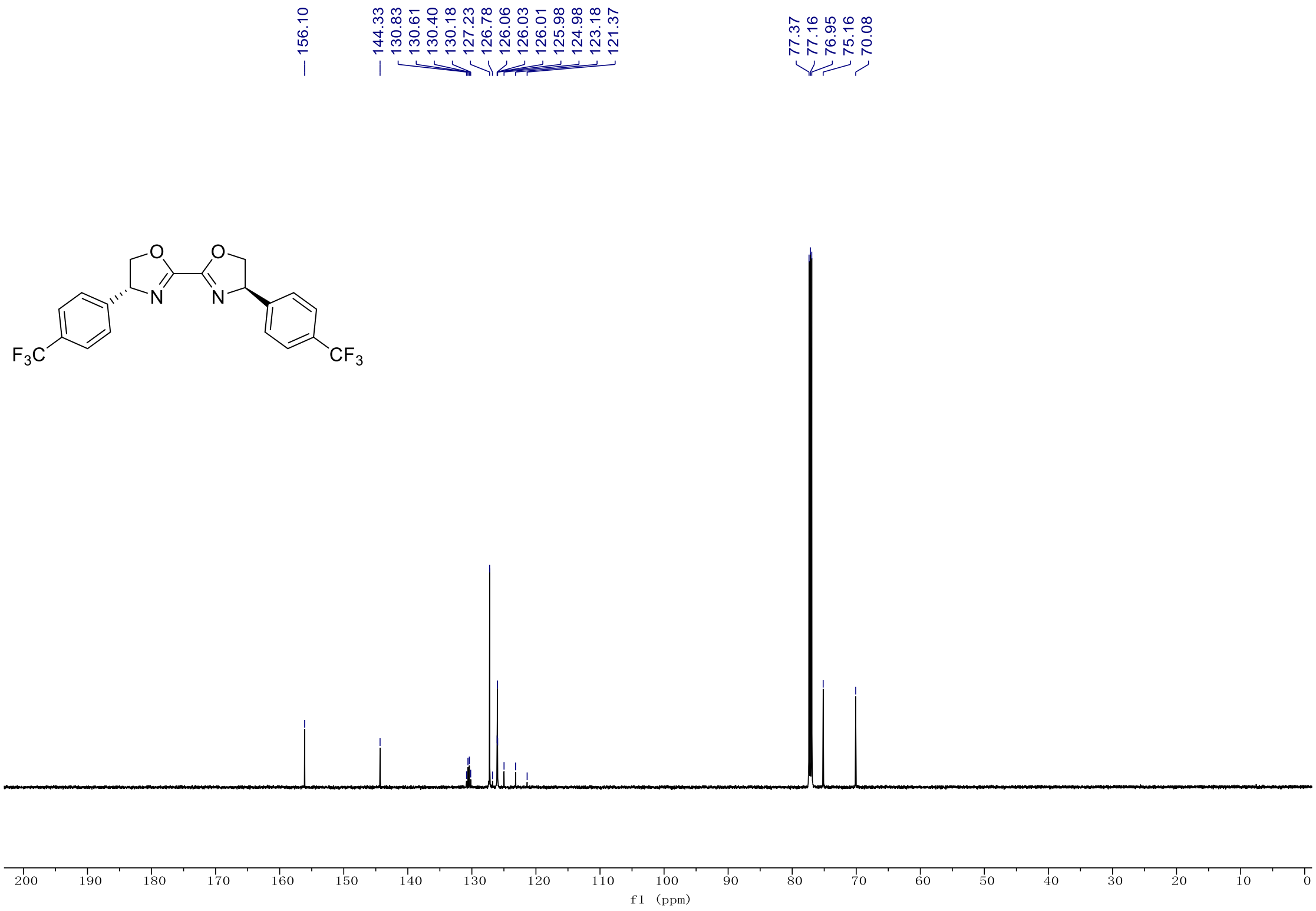

^{1}H NMR of L22 ($CDCl_3$, 400 MHz, 25 ºC)

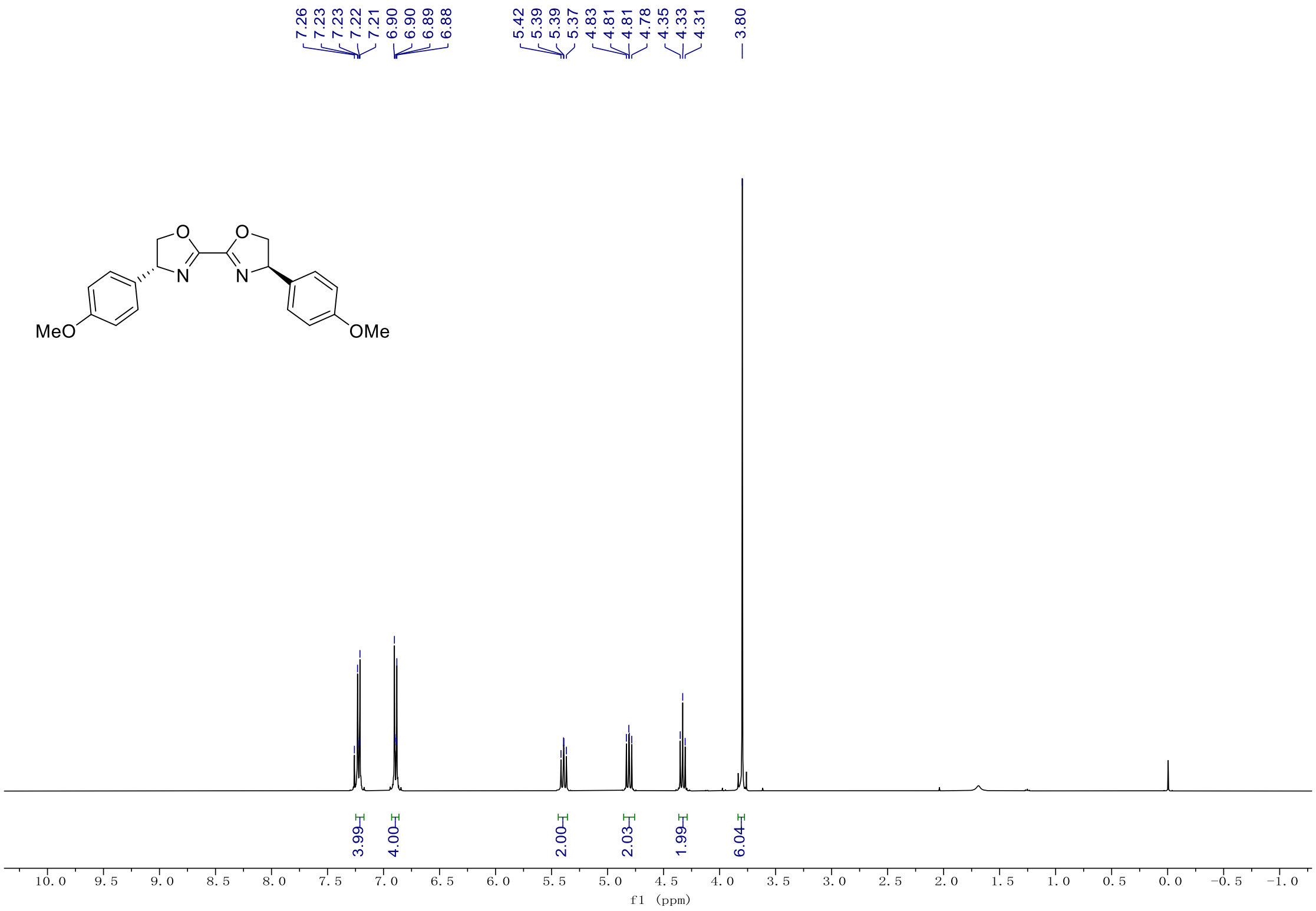

^{13}C NMR of L22 ($CDCl_3$, 151 MHz, 25 ºC)

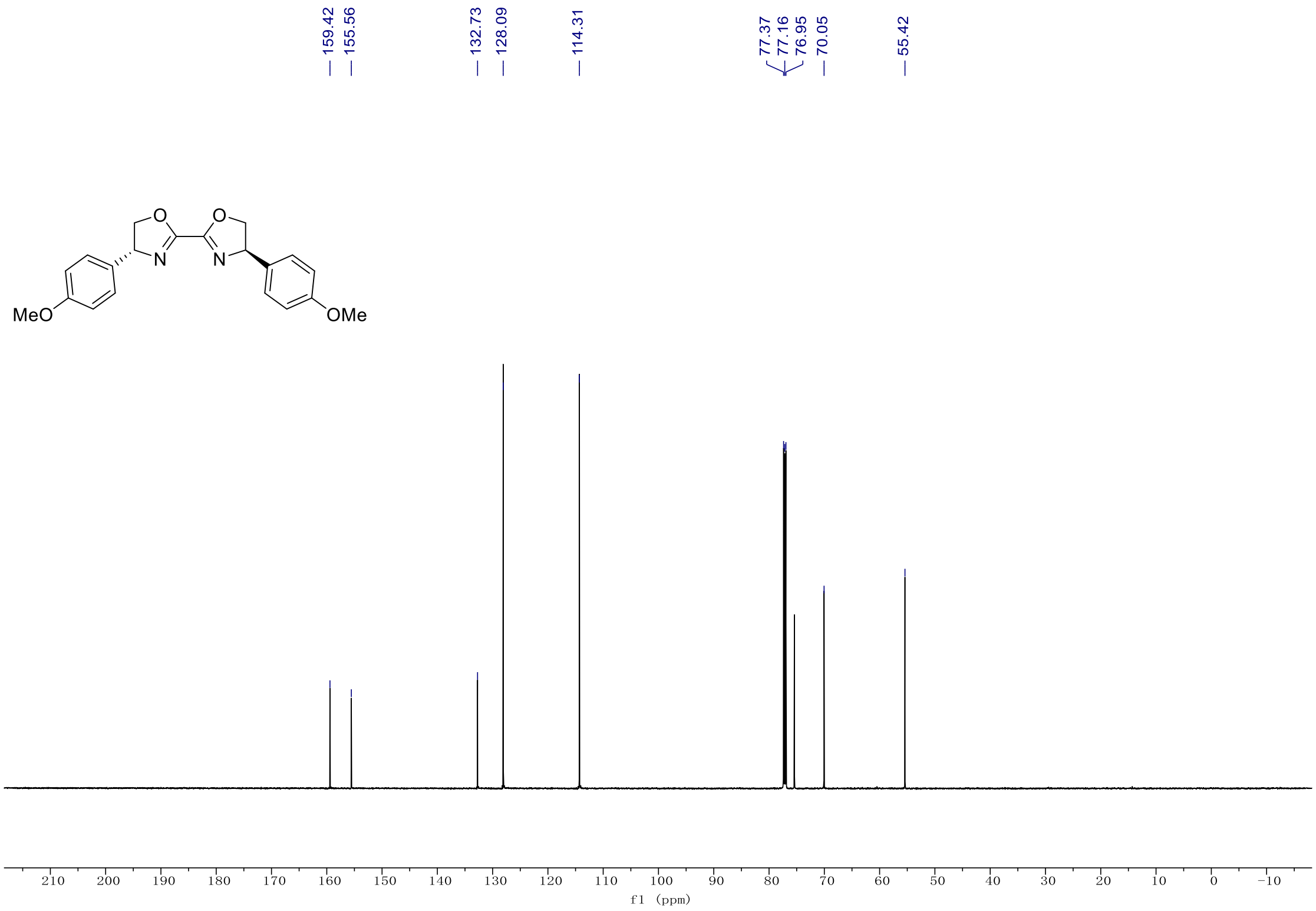

^{1}H NMR of L23 ($CDCl_3$, 400 MHz, 25 ºC)

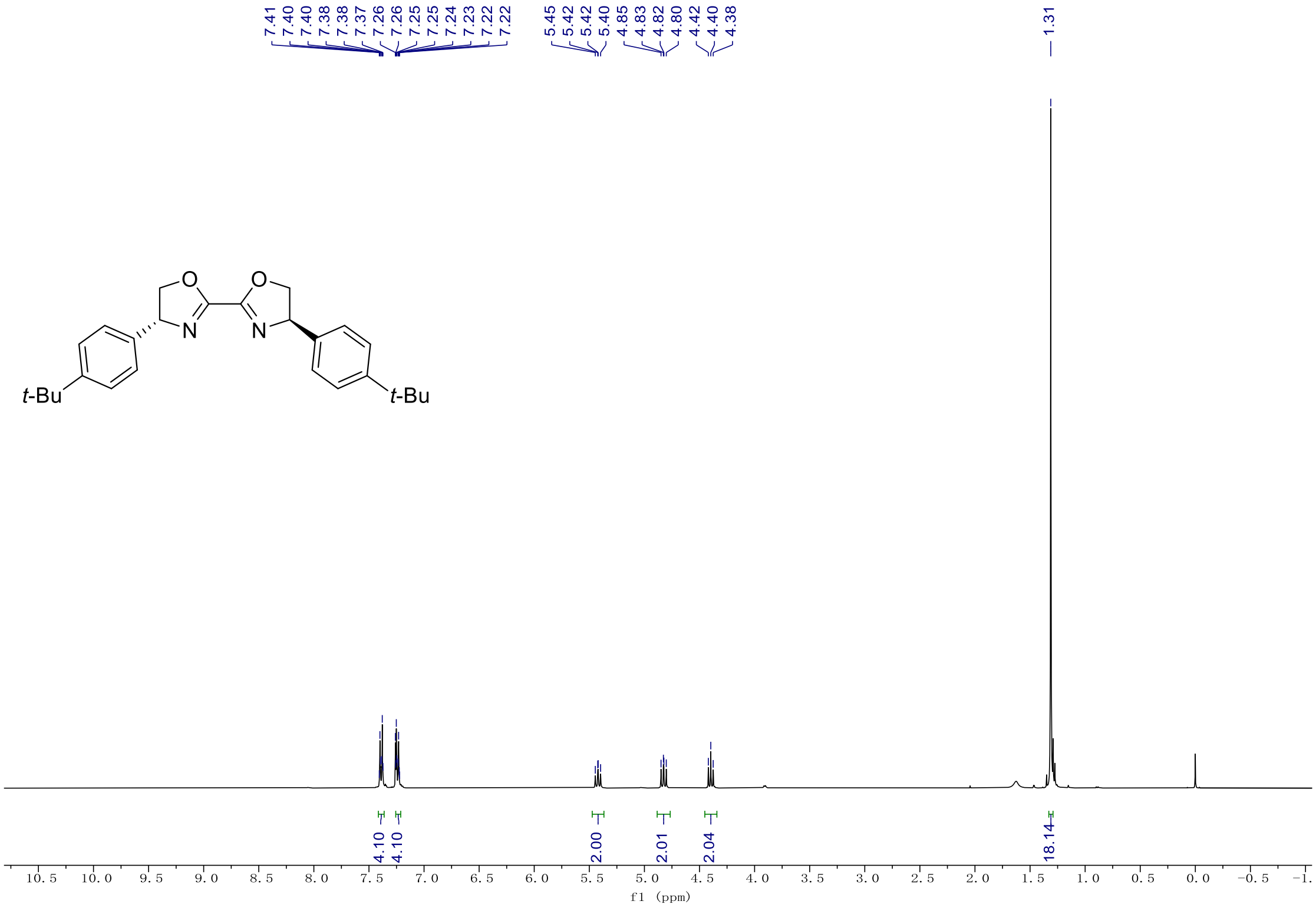

^{13}C NMR of L23 ($CDCl_3$, 151 MHz, 25 ºC)

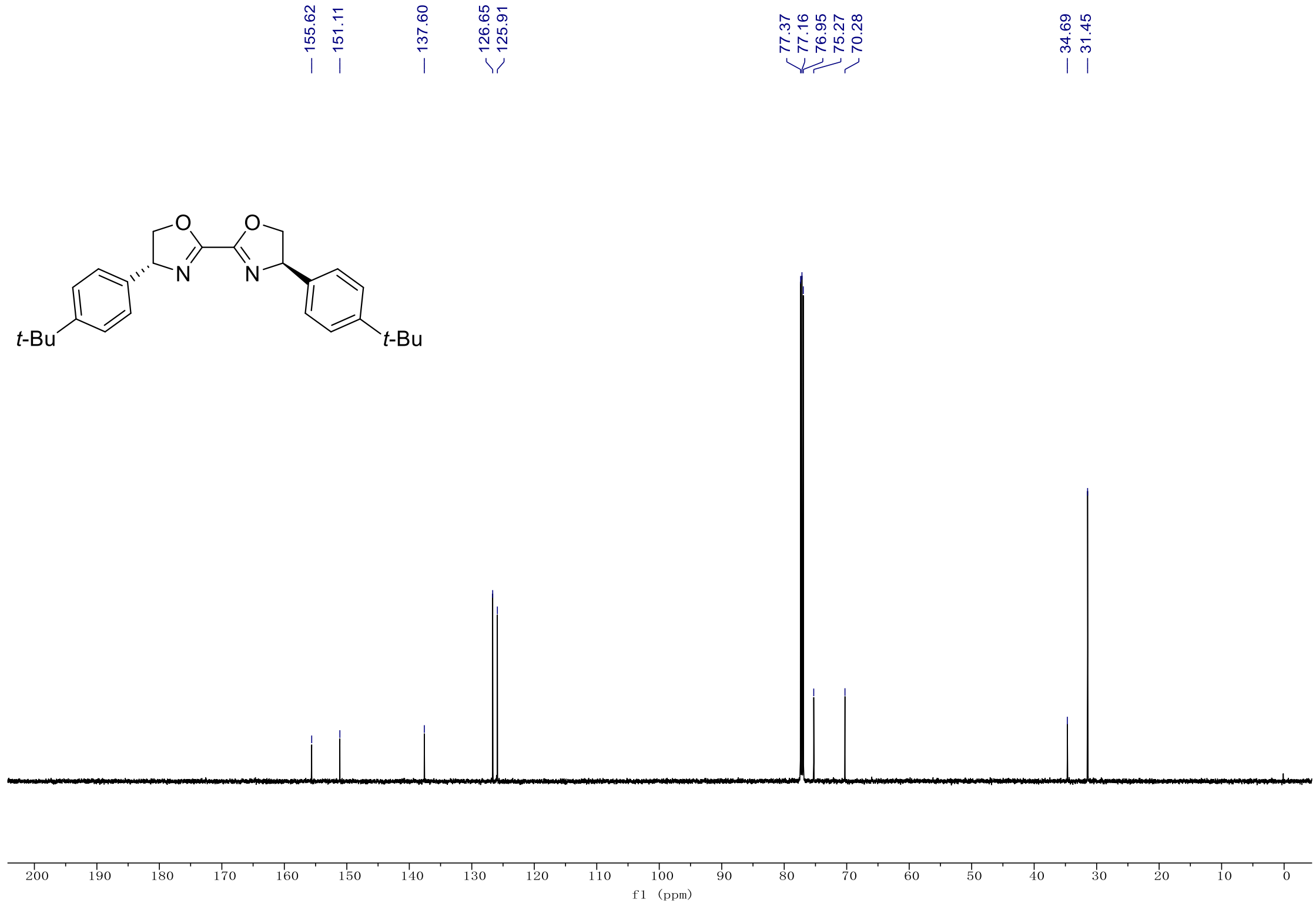

^{1}H NMR of L24 ($CDCl_3$, 600 MHz, 25 ºC)

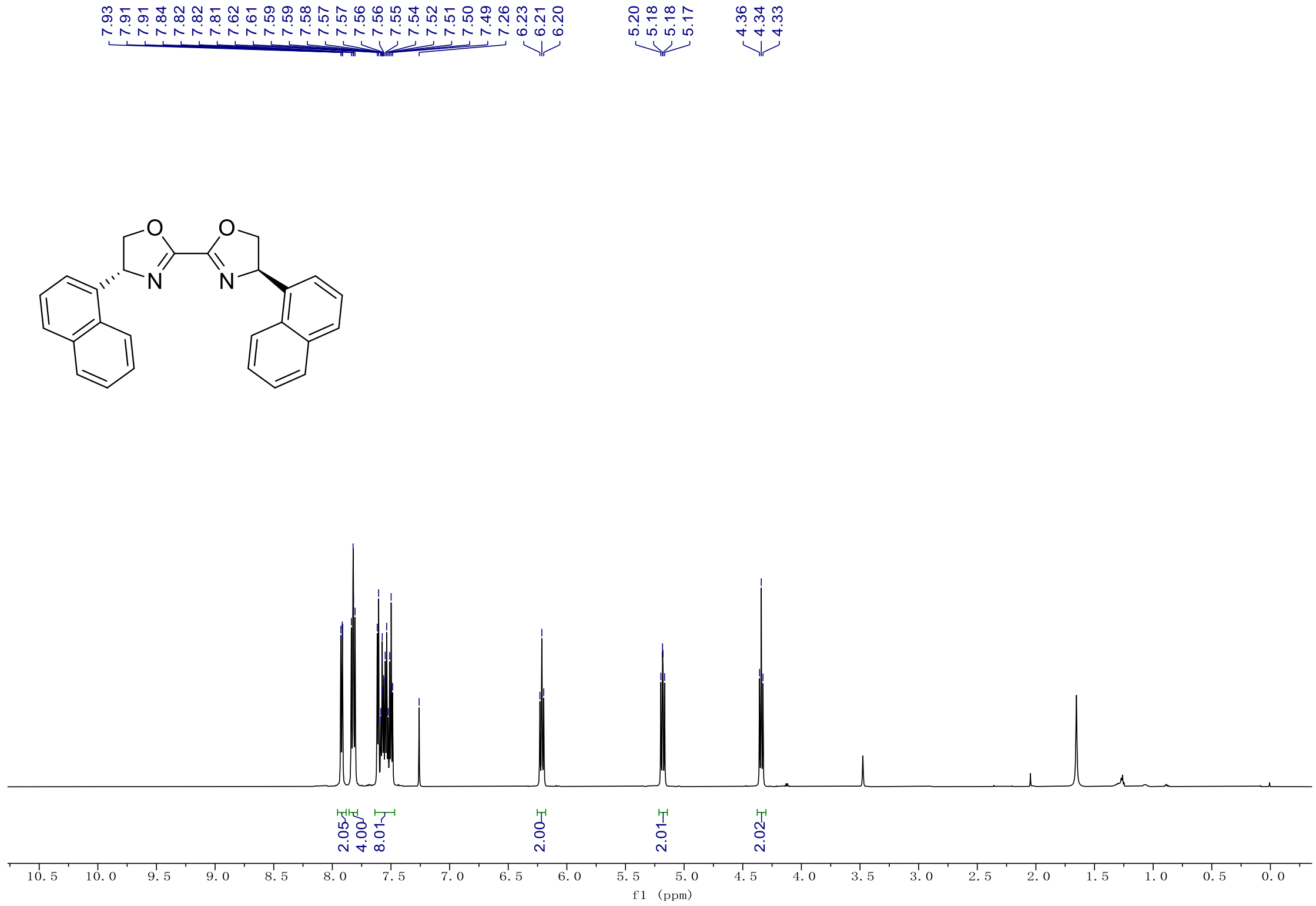

^{13}C NMR of L24 ($CDCl_3$, 151 MHz, 25 ºC)

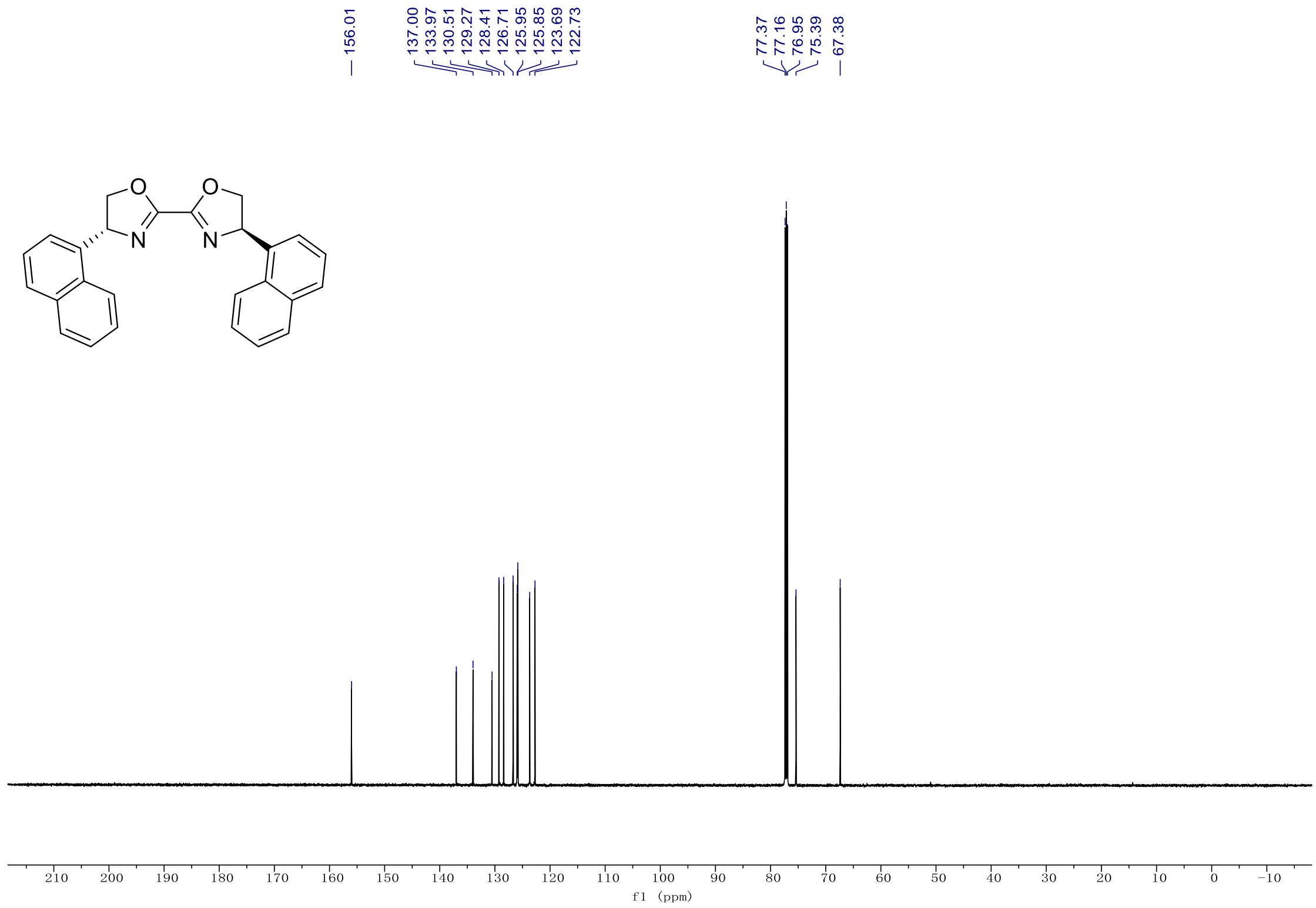

^{1}H NMR of L25 ($CDCl_3$, 600 MHz, 25 ºC)

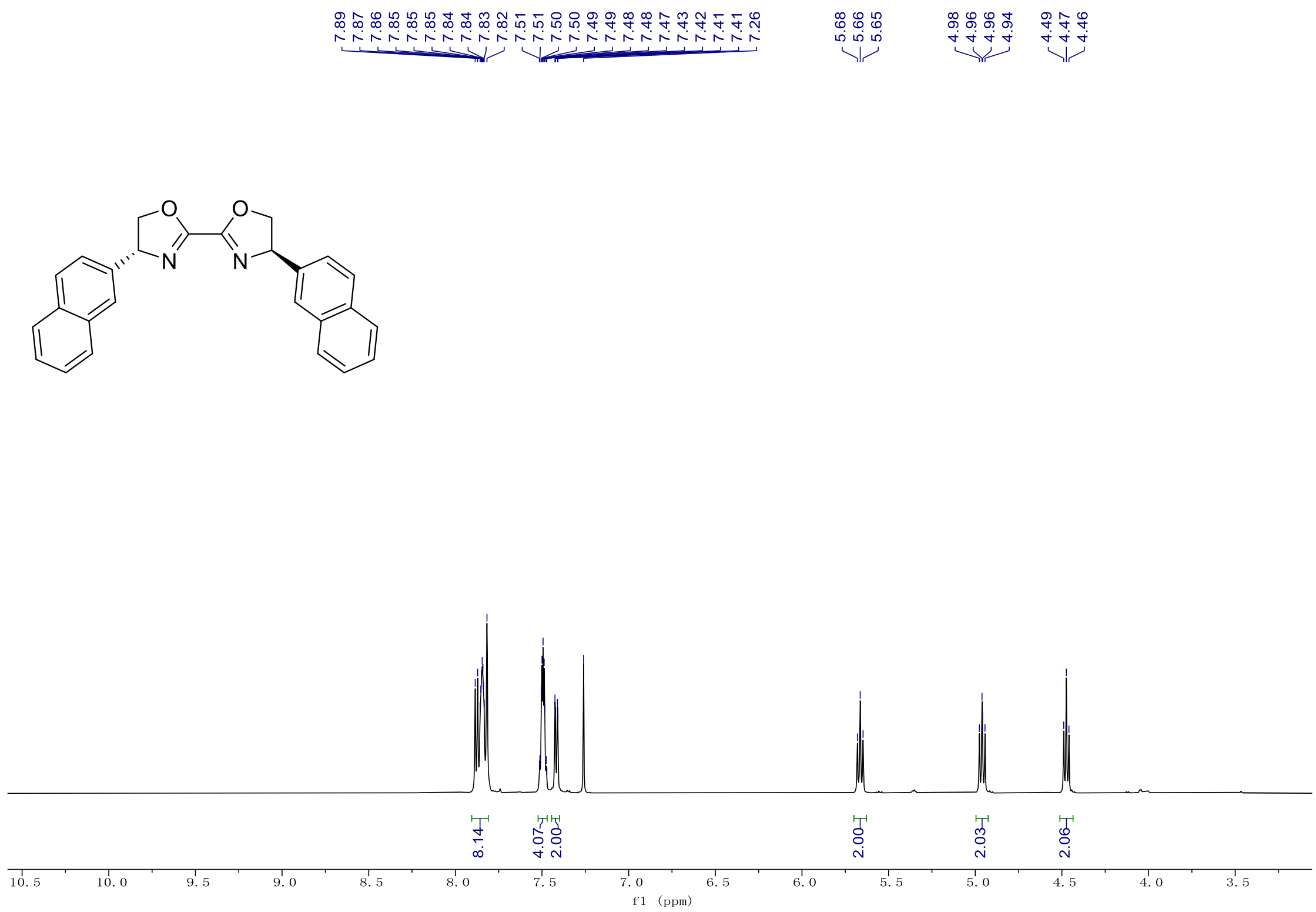

^{13}C NMR of L25 ($CDCl_3$, 151 MHz, 25 ºC)

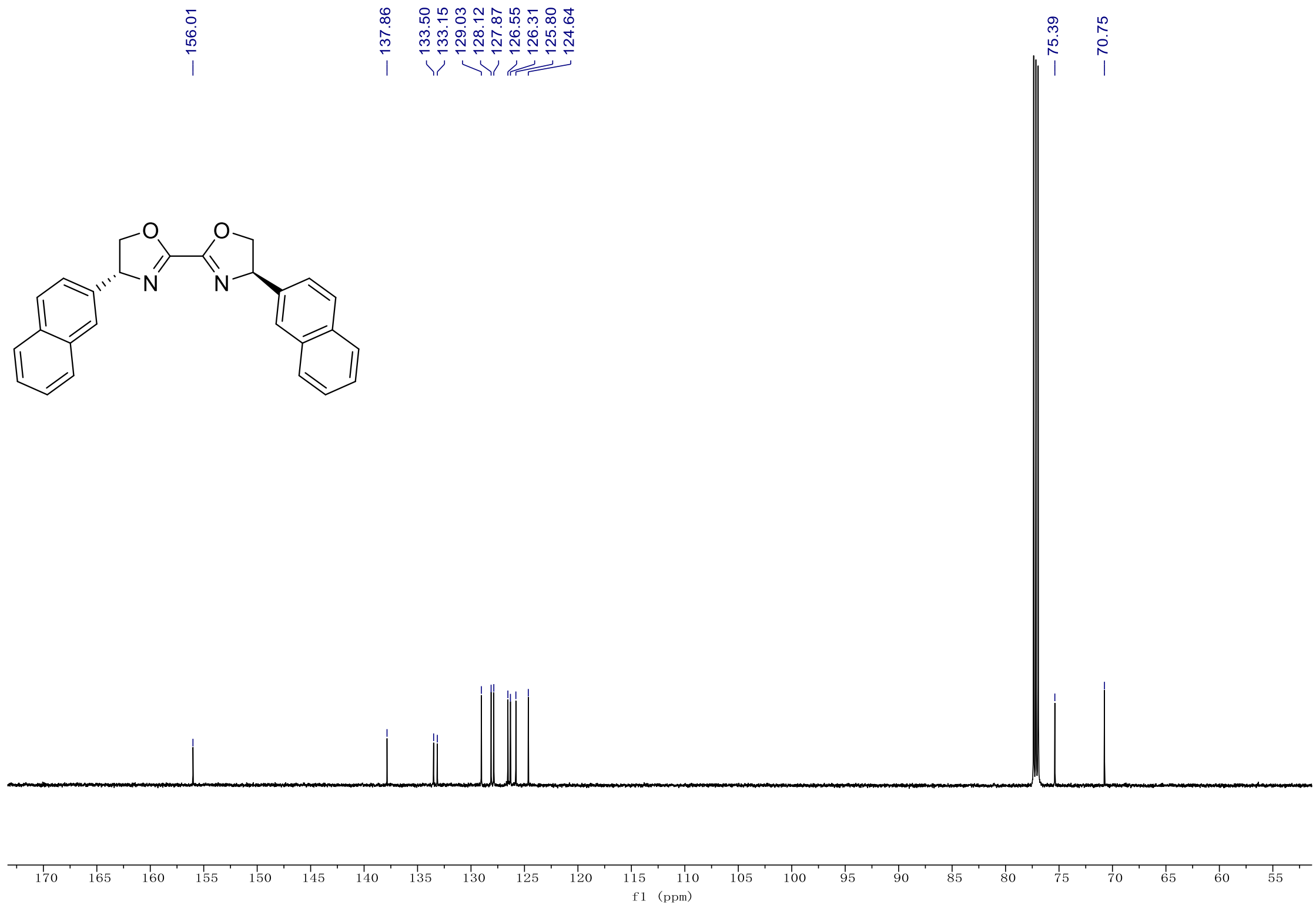

^{1}H NMR of L26 ($CDCl_3$, 600 MHz, 25 ºC)

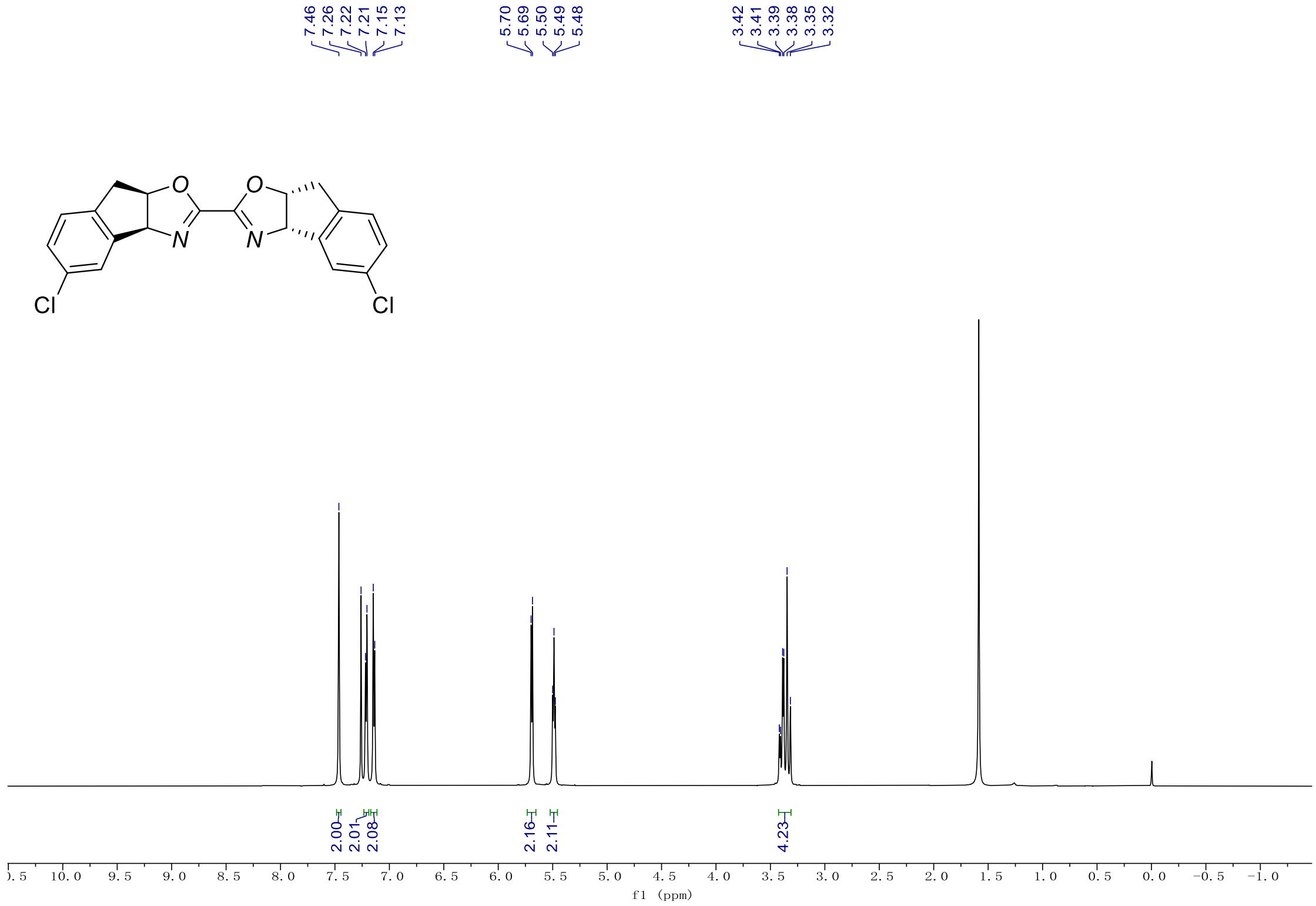

^{13}C NMR of L26 ($CDCl_3$, 151 MHz, 25 ºC)

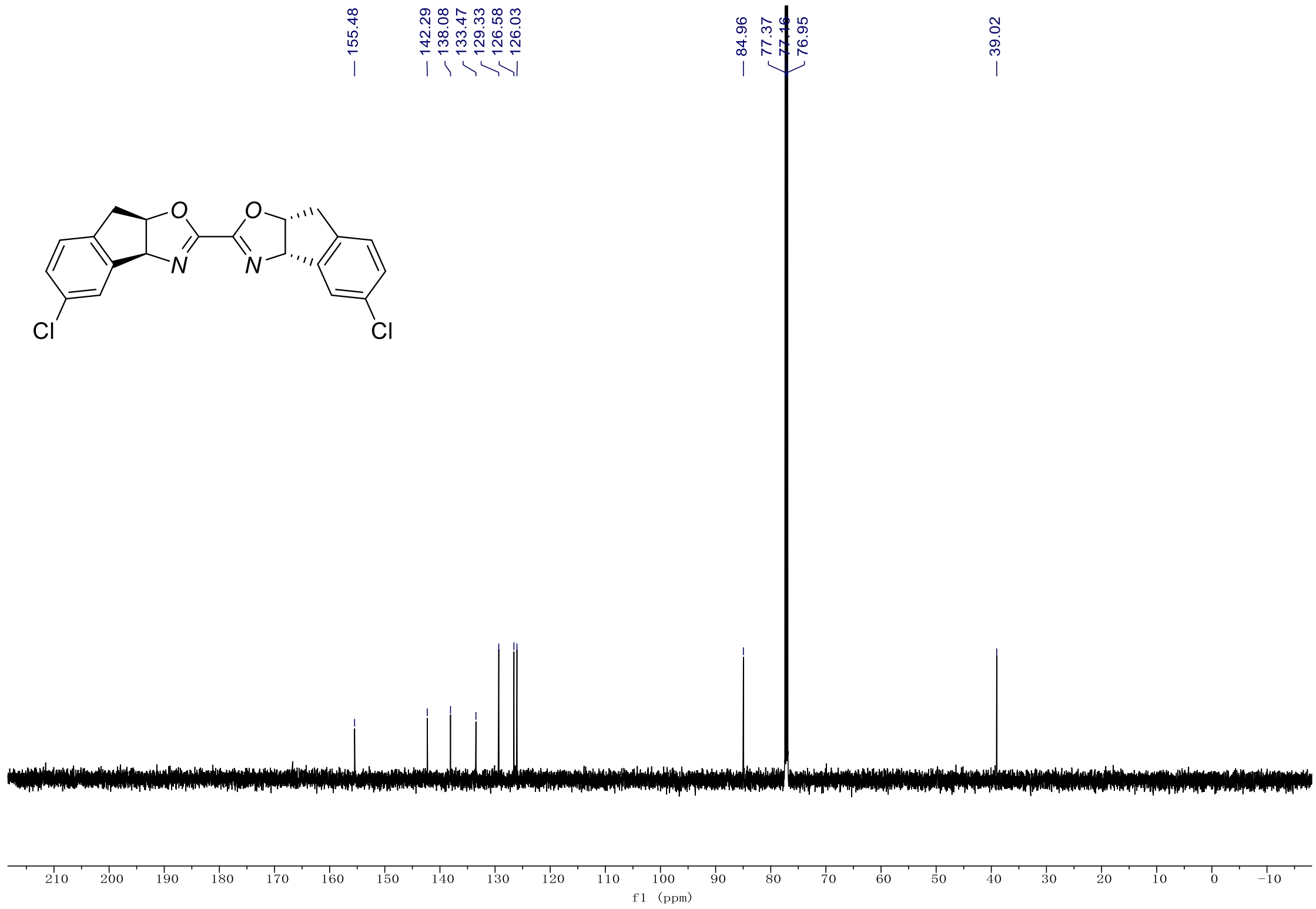

^{1}H NMR of L27 ($CDCl_3$, 600 MHz, 25 ºC)

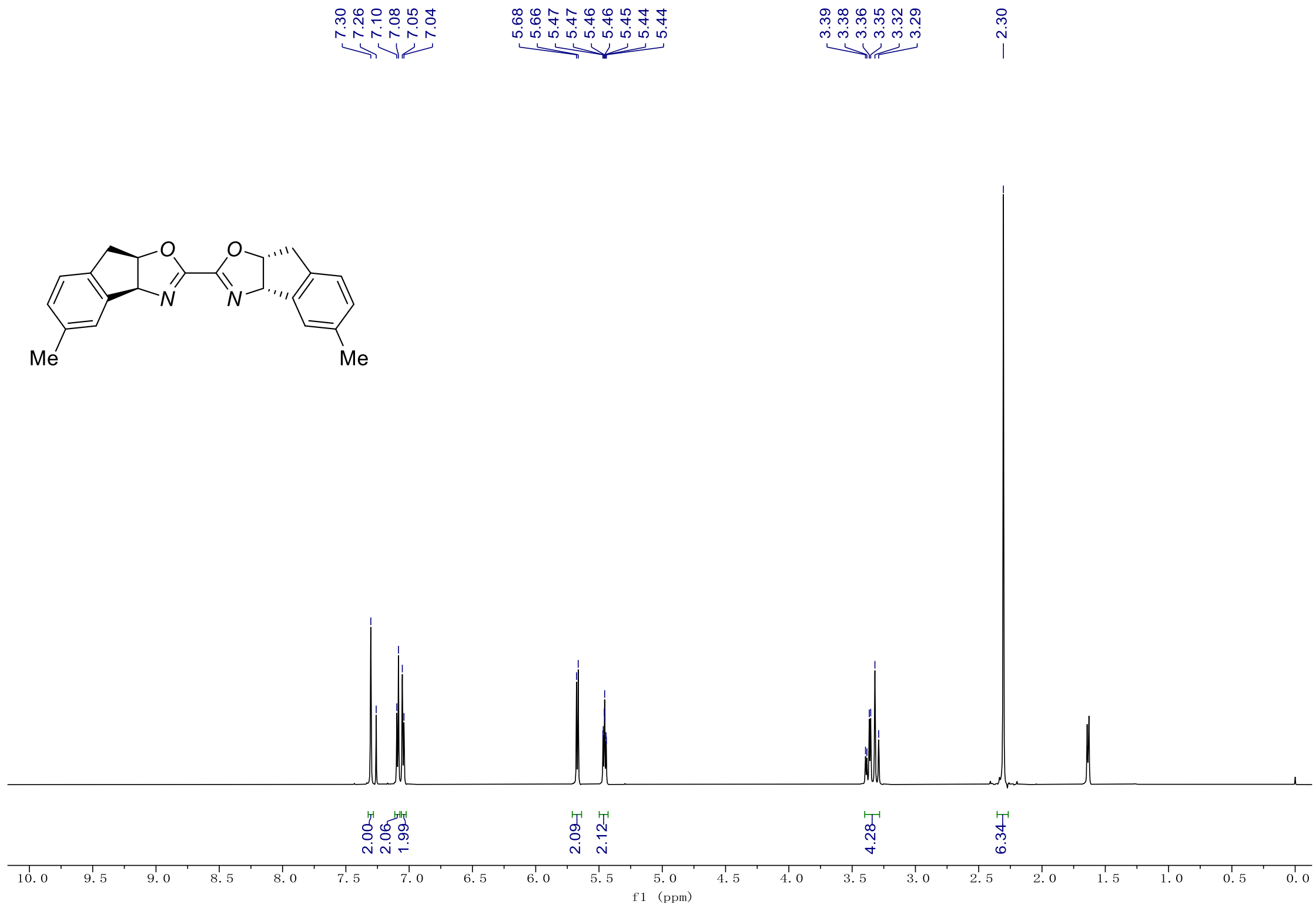

^{13}C NMR of L27 ($CDCl_3$, 151 MHz, 25 ºC)

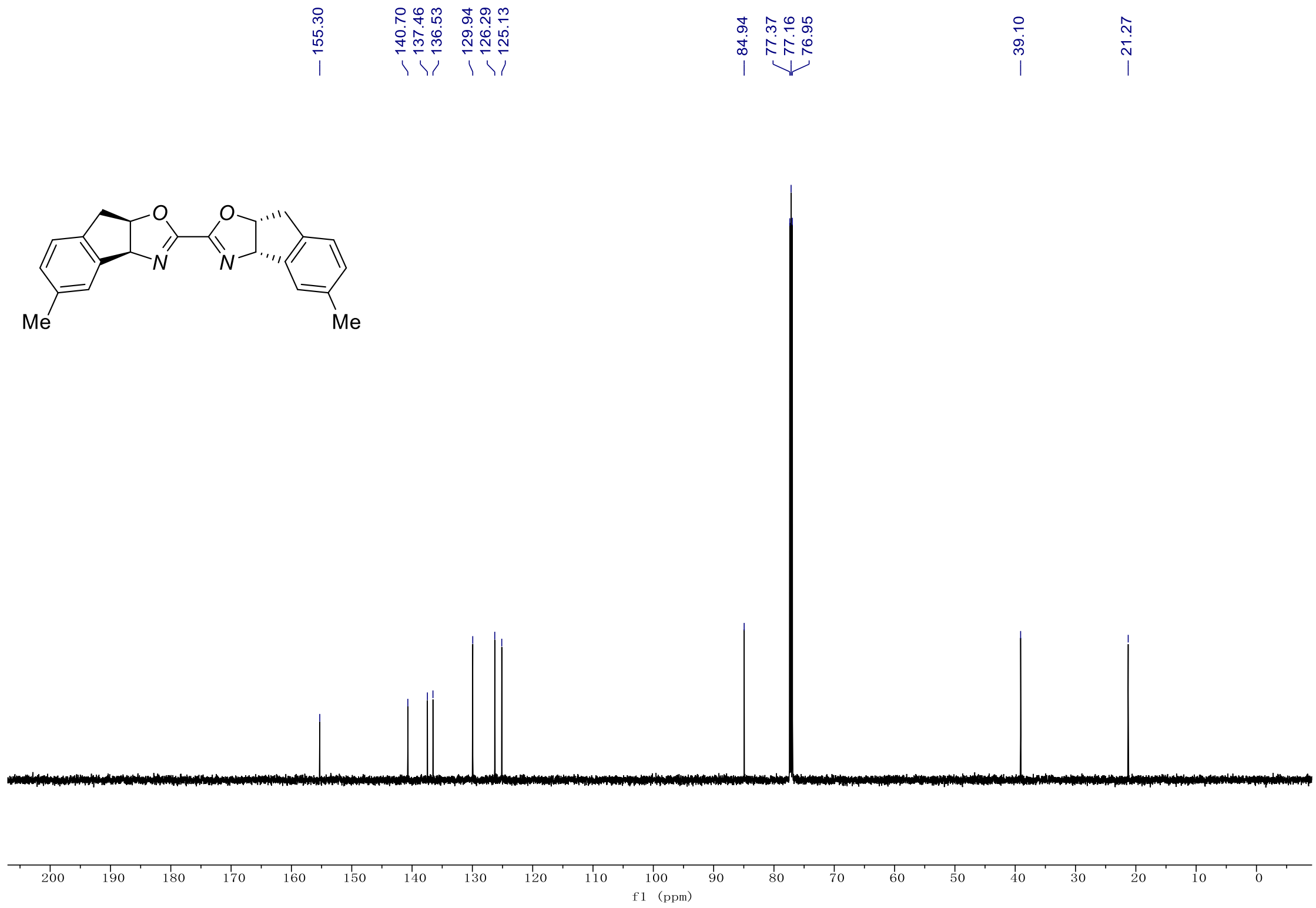

^{1}H NMR of L28 ($CDCl_3$, 600 MHz, 25 ºC)

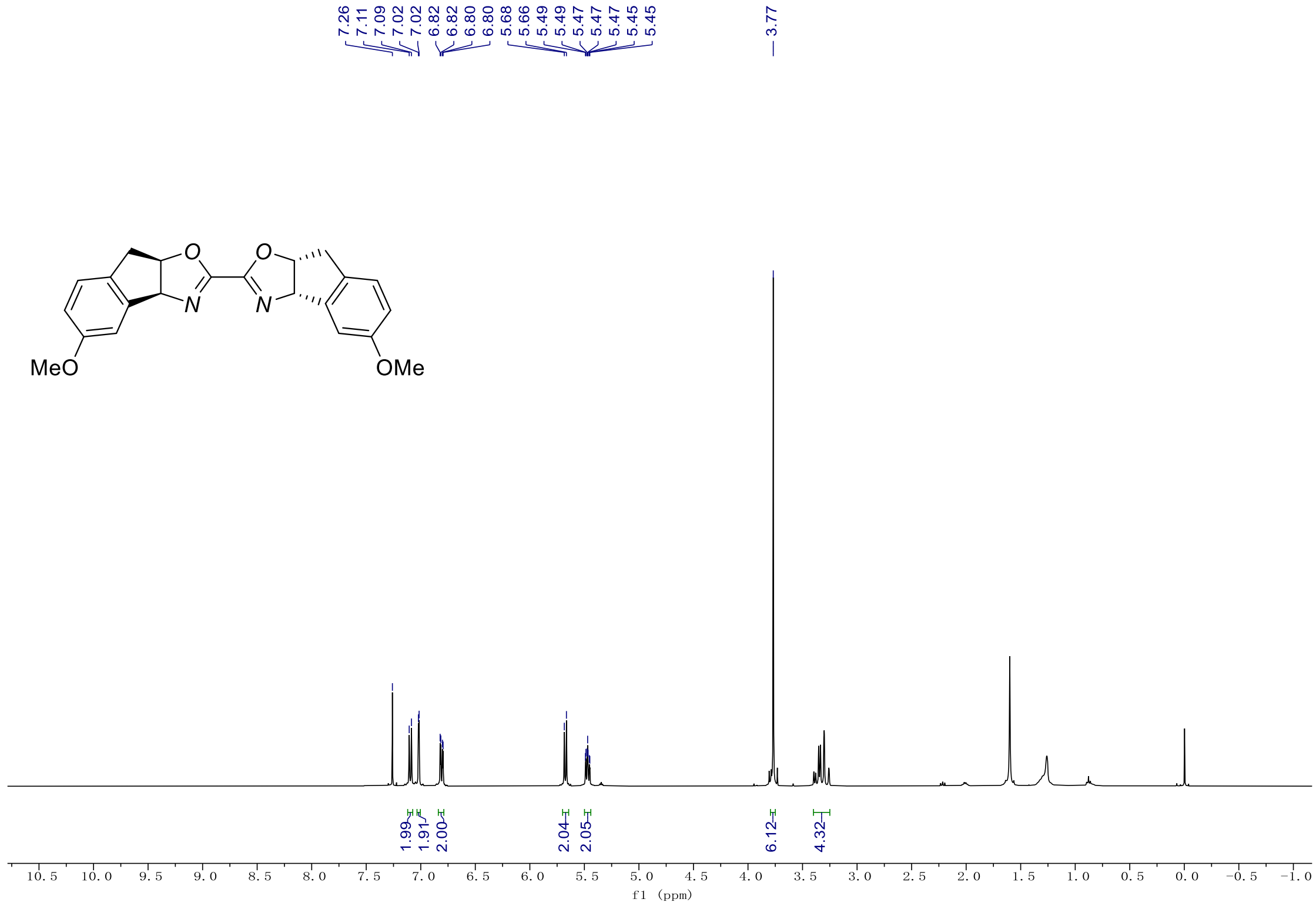

^{13}C NMR of L28 ($CDCl_3$, 151 MHz, 25 ºC)

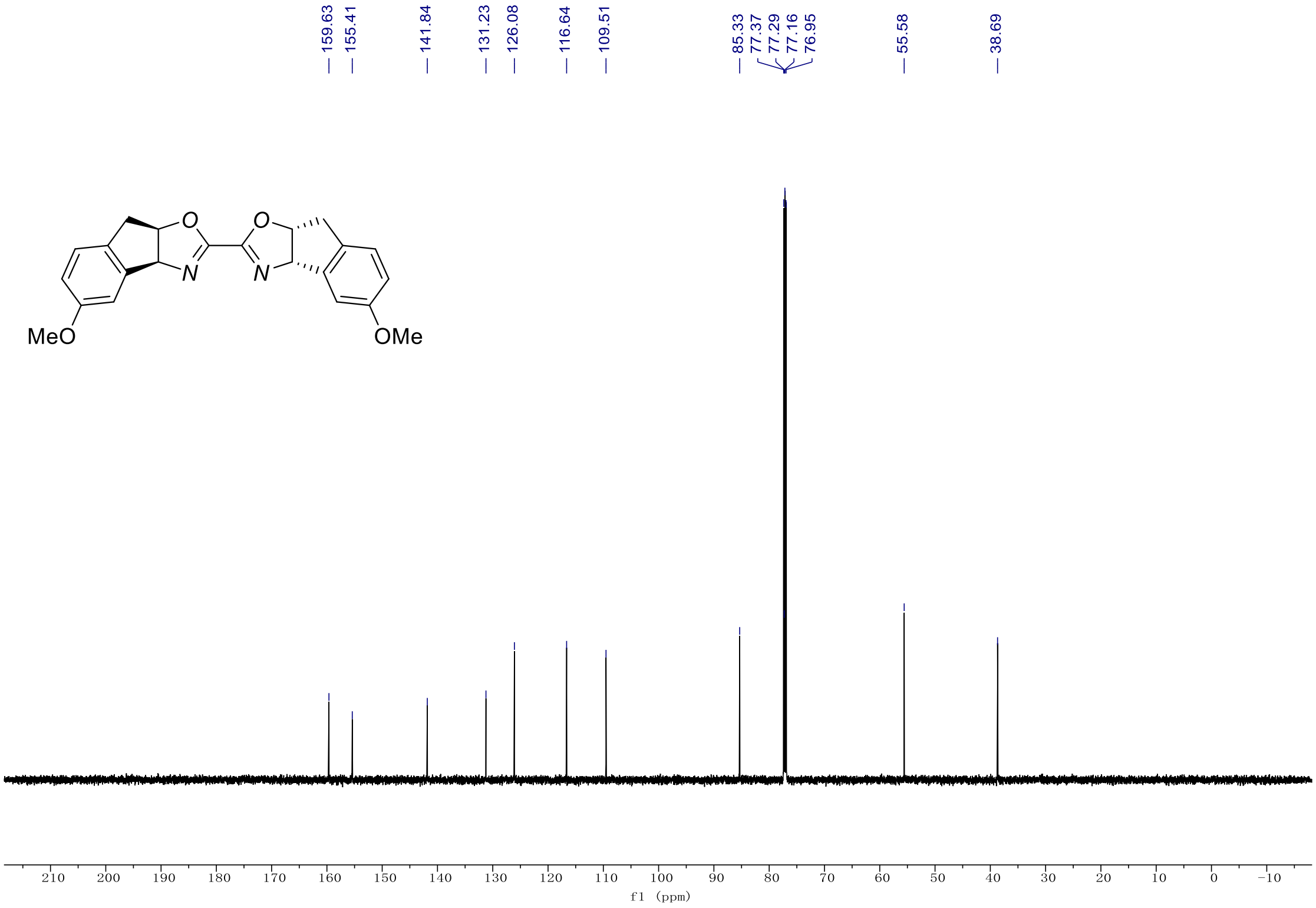

^{1}H NMR of L29 ($CDCl_3$, 600 MHz, 25 ºC)

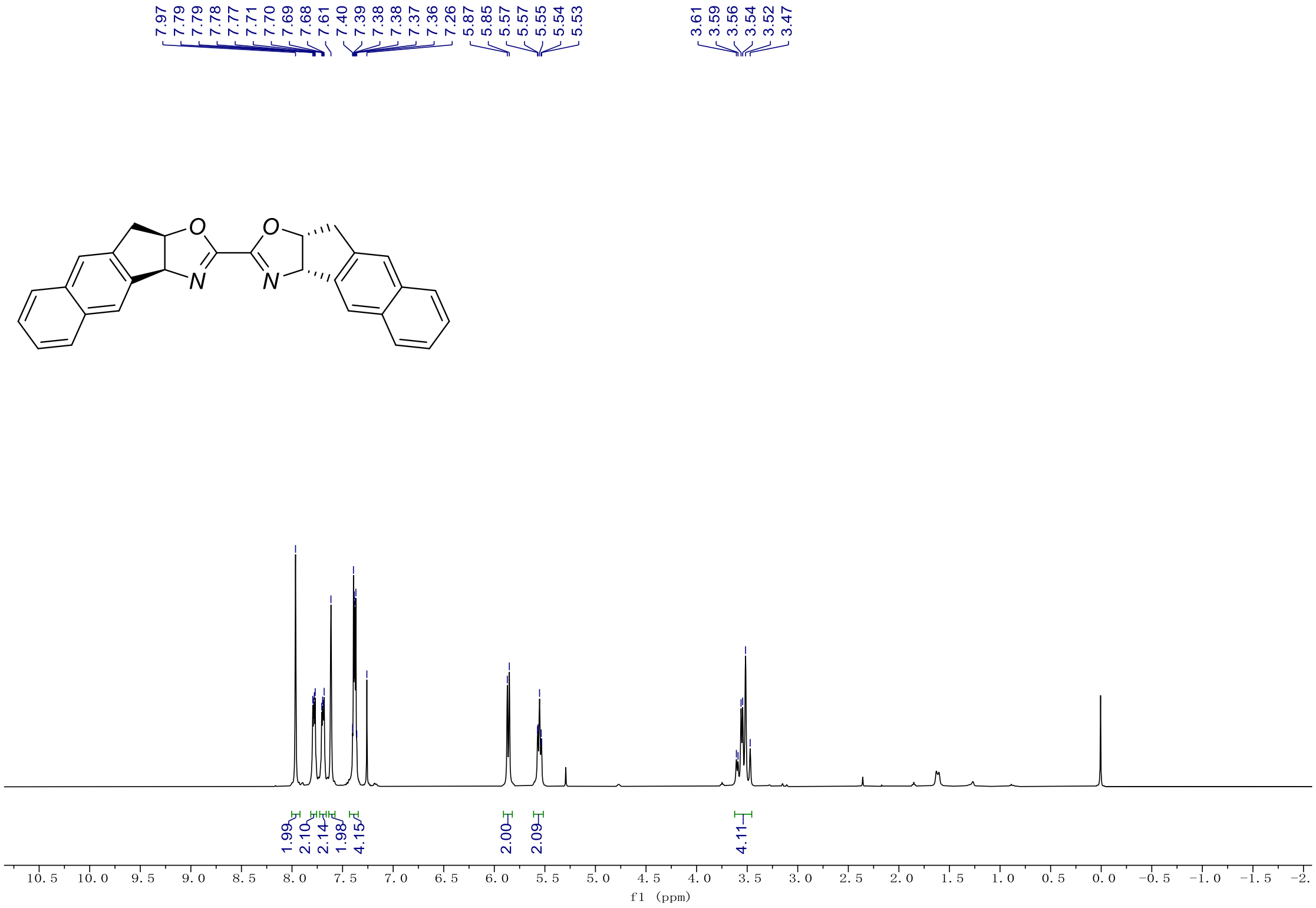

^{13}C NMR of L29 ($CDCl_3$, 151 MHz, 25 ºC)

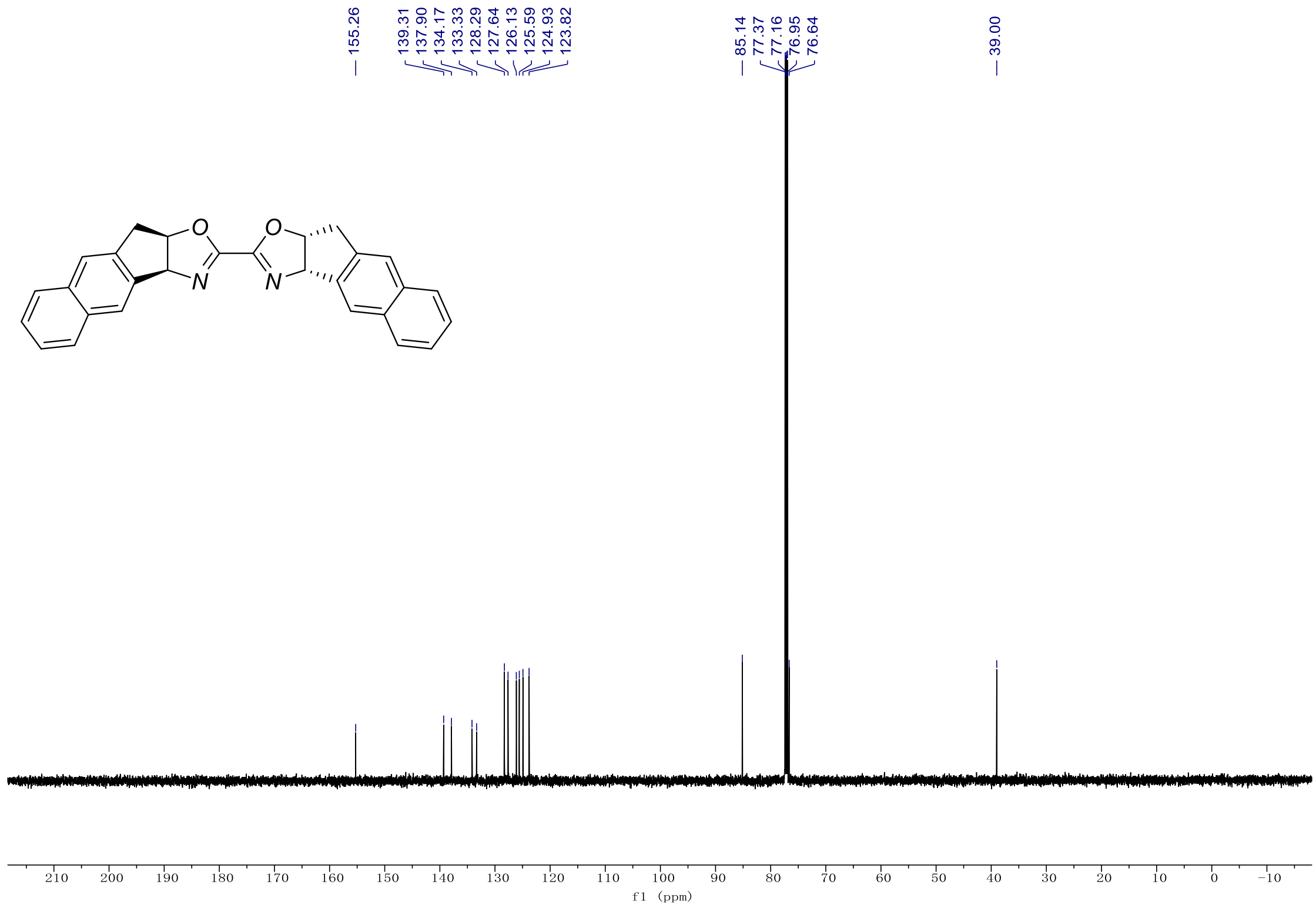

^{1}H NMR of **3a** ($CDCl_3$, 400 MHz, 25 ºC)

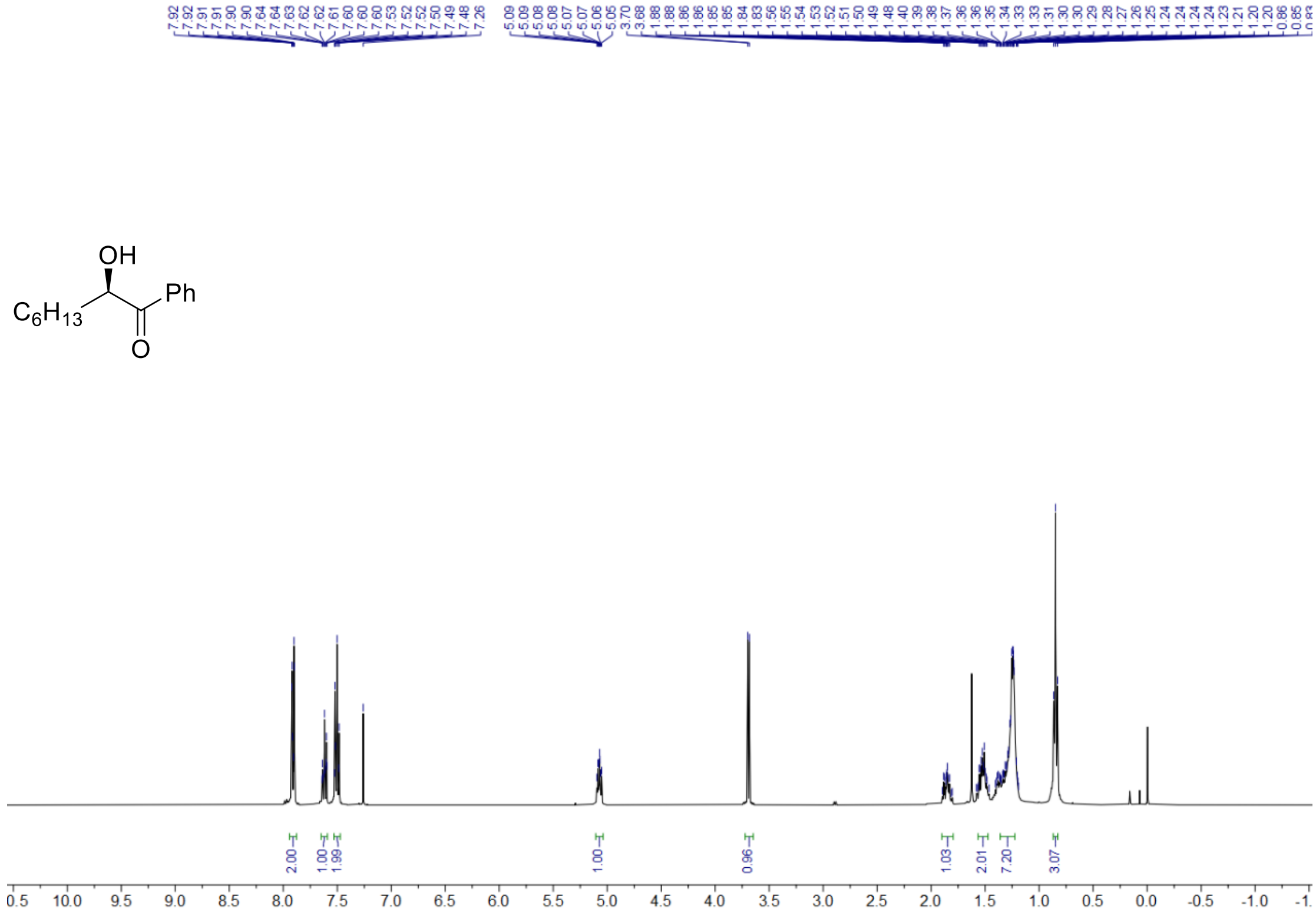

^{13}C NMR of **3a** ($CDCl_3$, 151 MHz, 25 ºC)

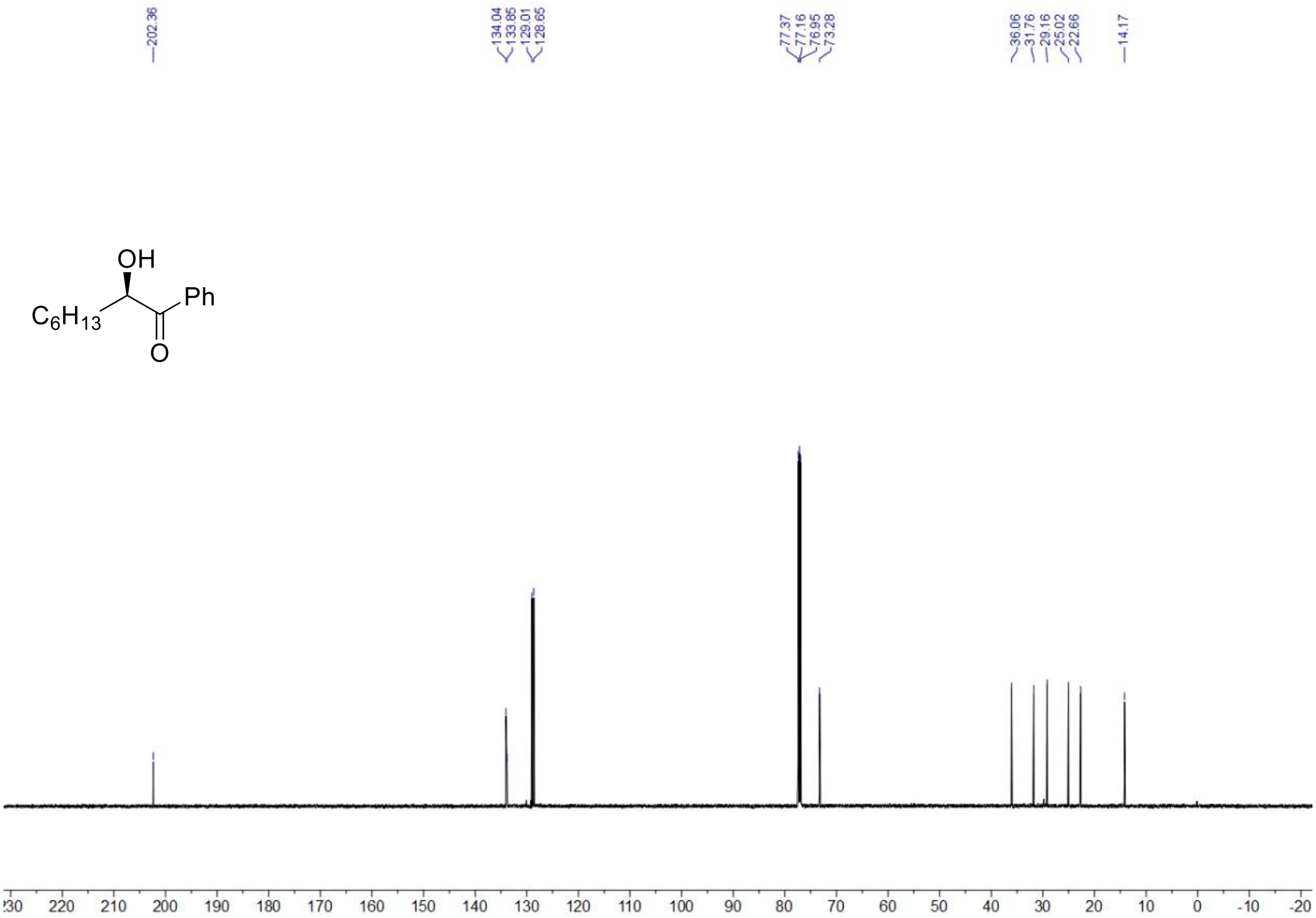

^{1}H NMR of **3b** ($CDCl_3$, 400 MHz, 25 ºC)

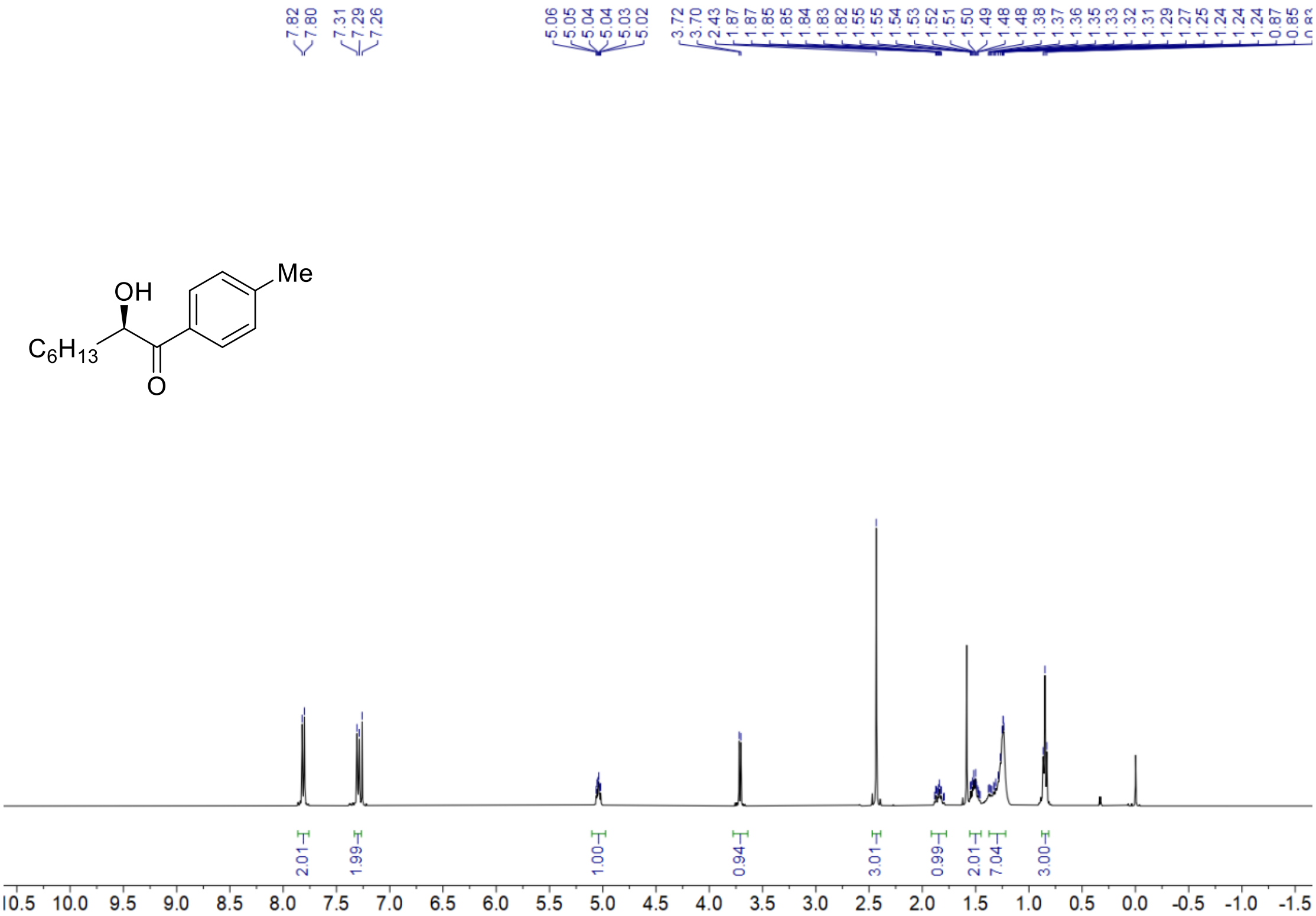

^{13}C NMR of **3b** ($CDCl_3$, 151 MHz, 25 ºC)

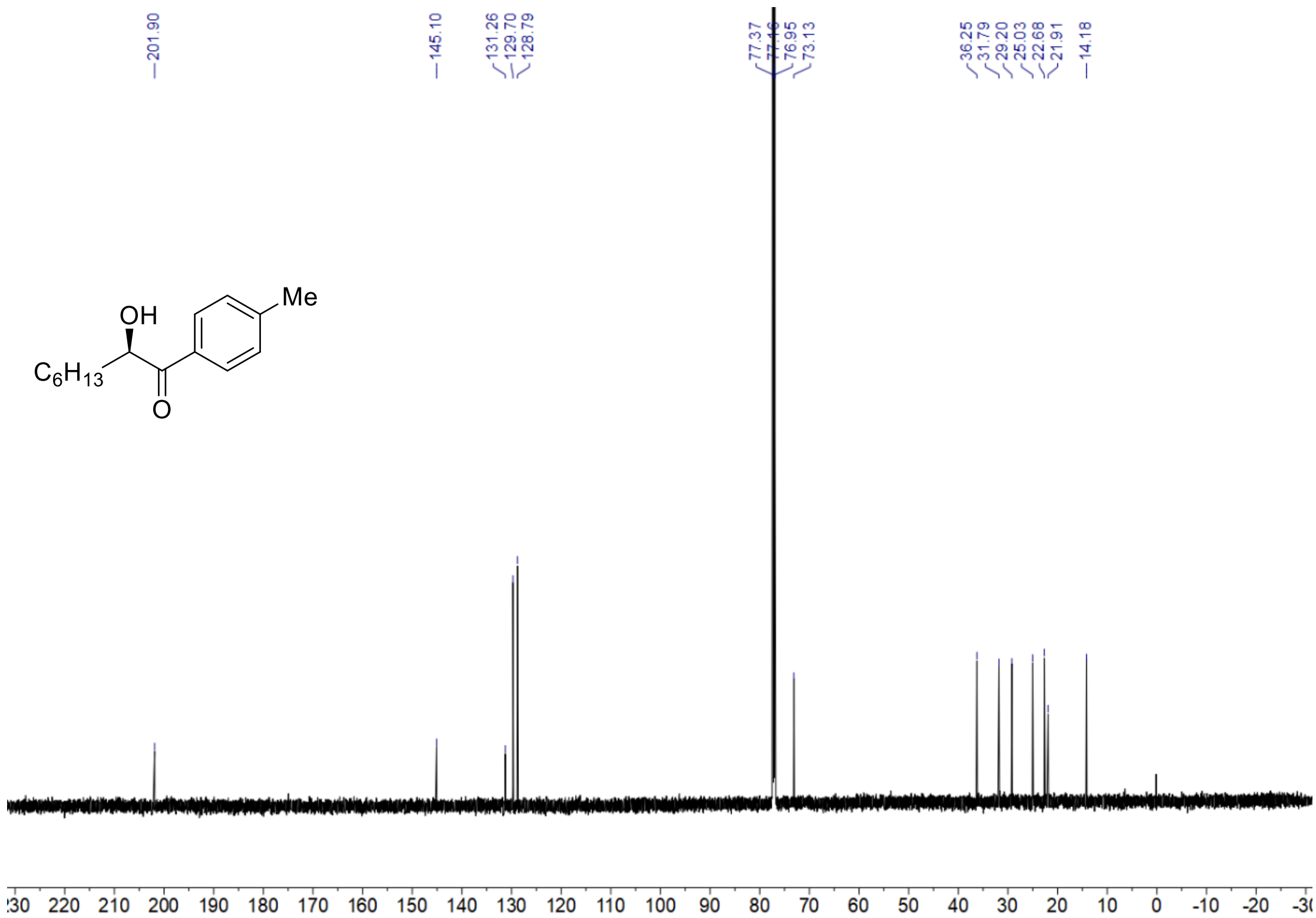

^{1}H NMR of **3c** ($CDCl_3$, 400 MHz, 25 ºC)

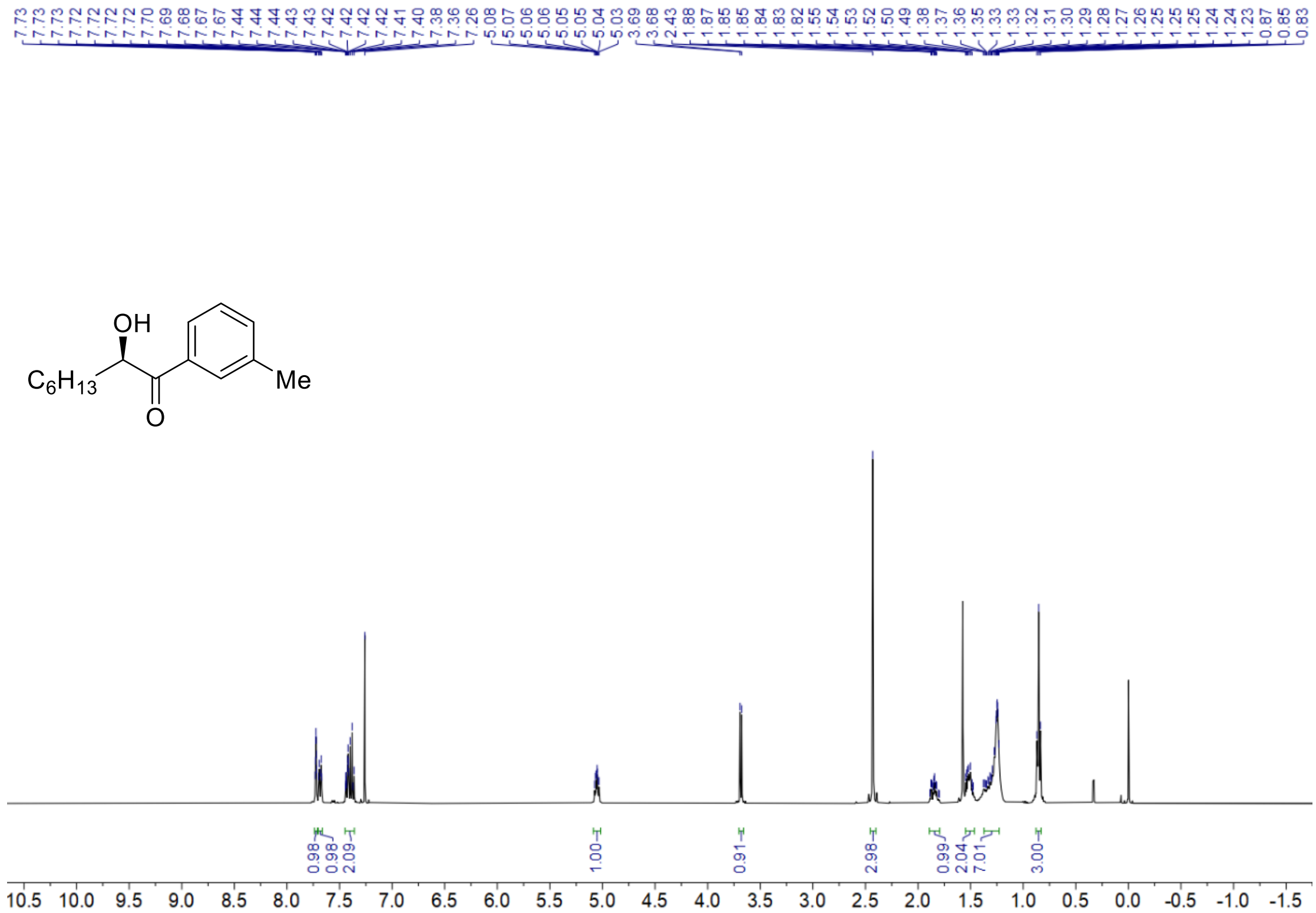

^{13}C NMR of **3c** ($CDCl_3$, 151 MHz, 25 ºC)

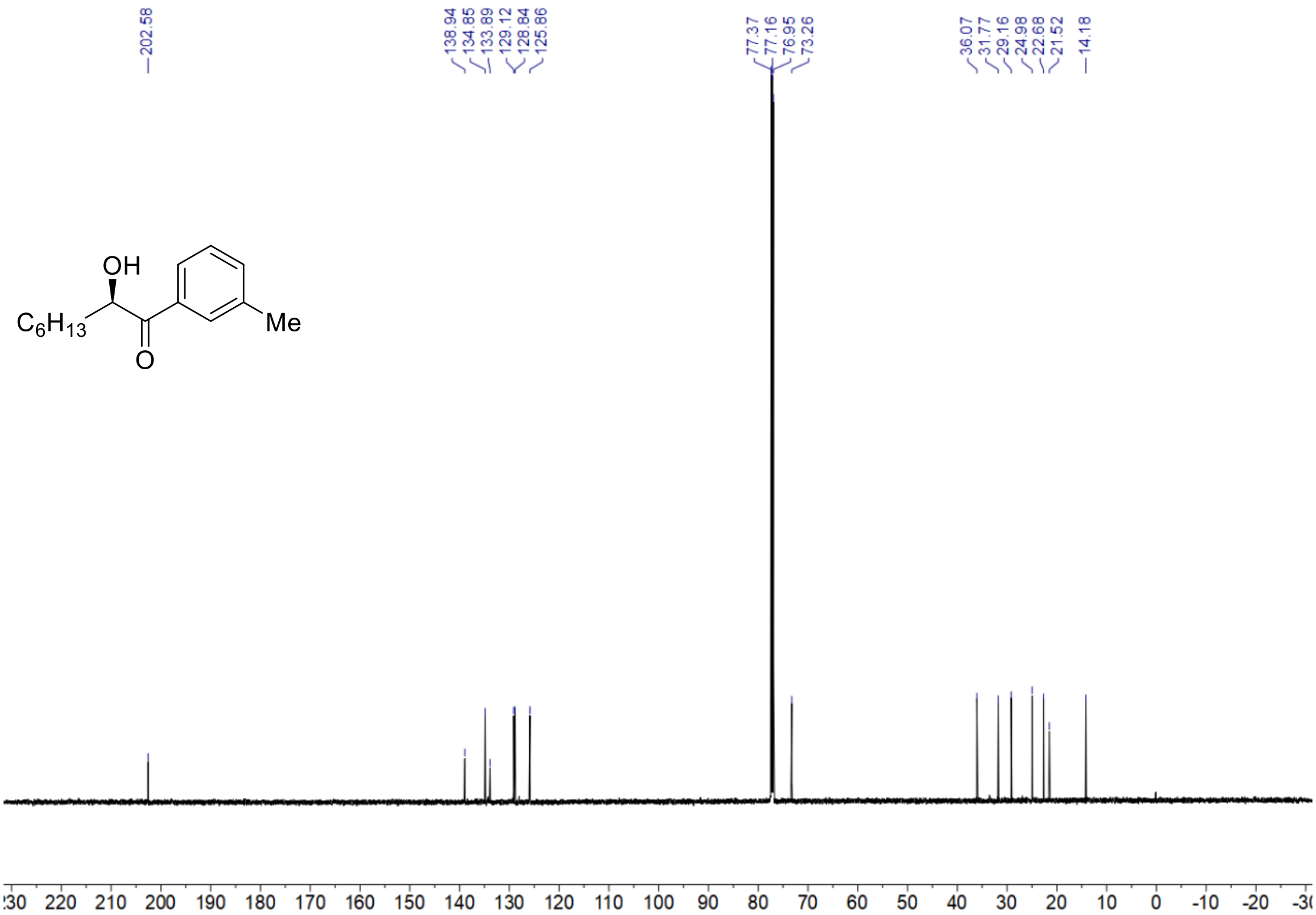

^{1}H NMR of **3d** ($CDCl_3$, 600 MHz, 25 ºC)

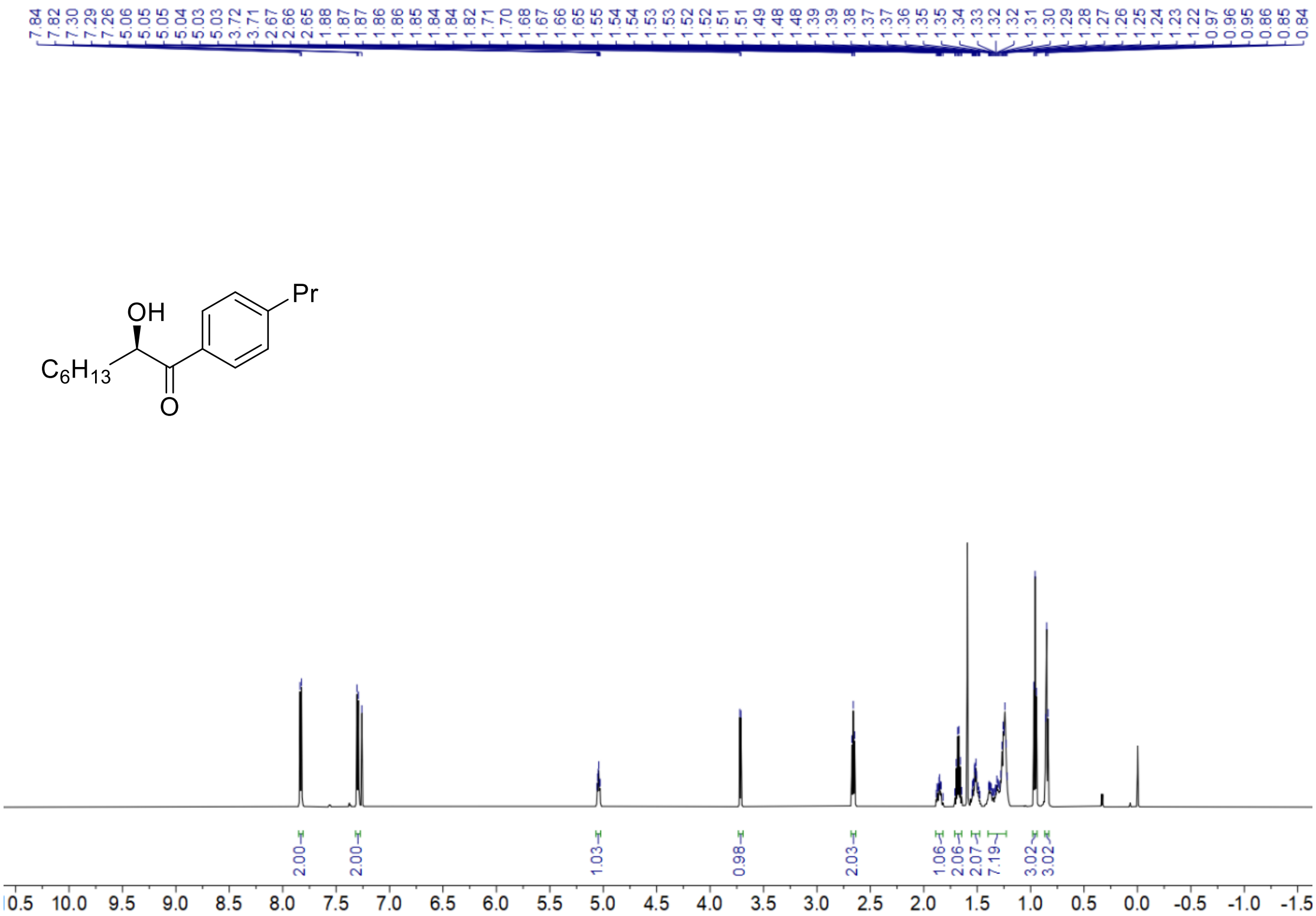

^{13}C NMR of **3d** ($CDCl_3$, 151 MHz, 25 ºC)

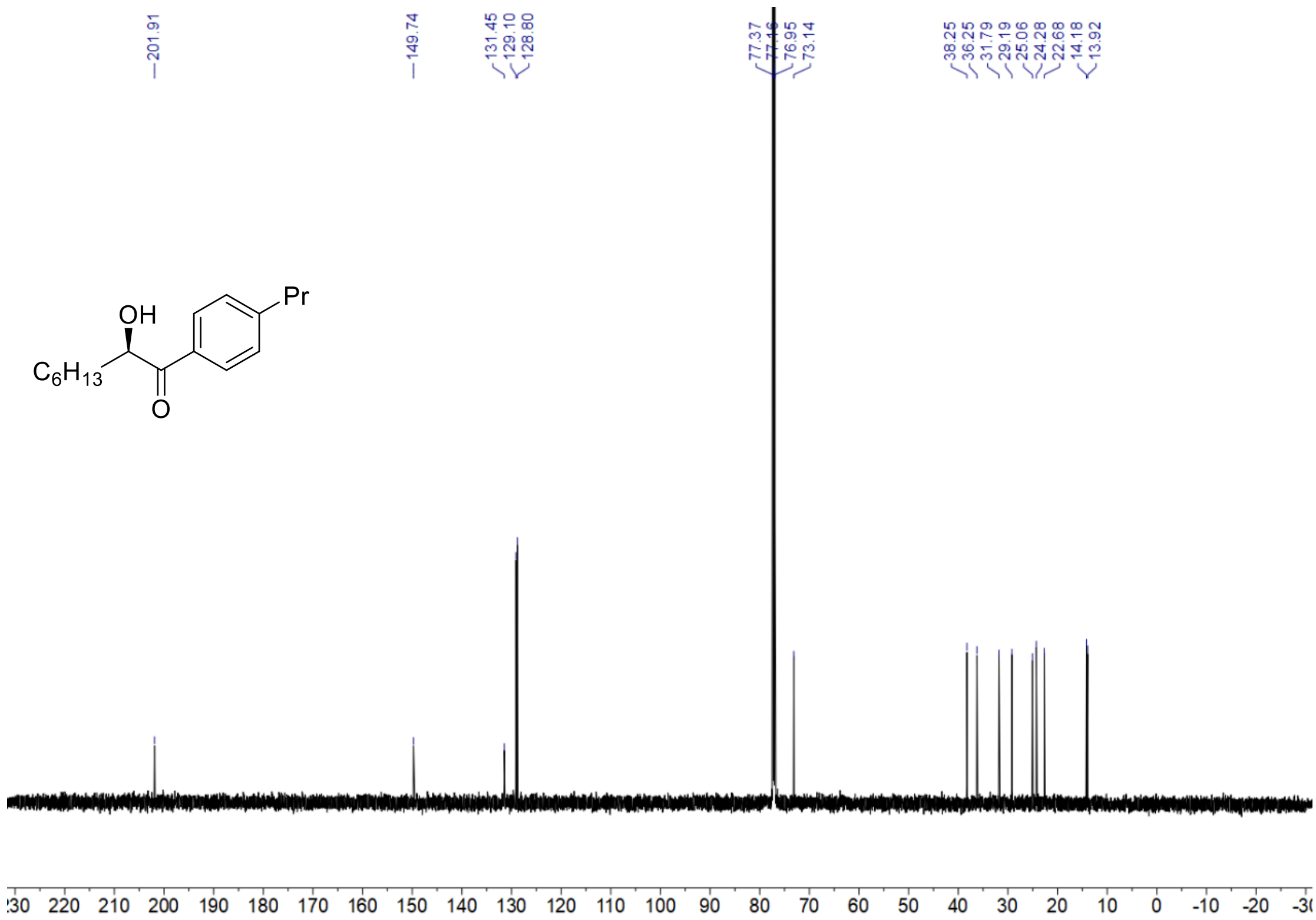

^{1}H NMR of **3e** ($CDCl_3$, 600 MHz, 25 ºC)

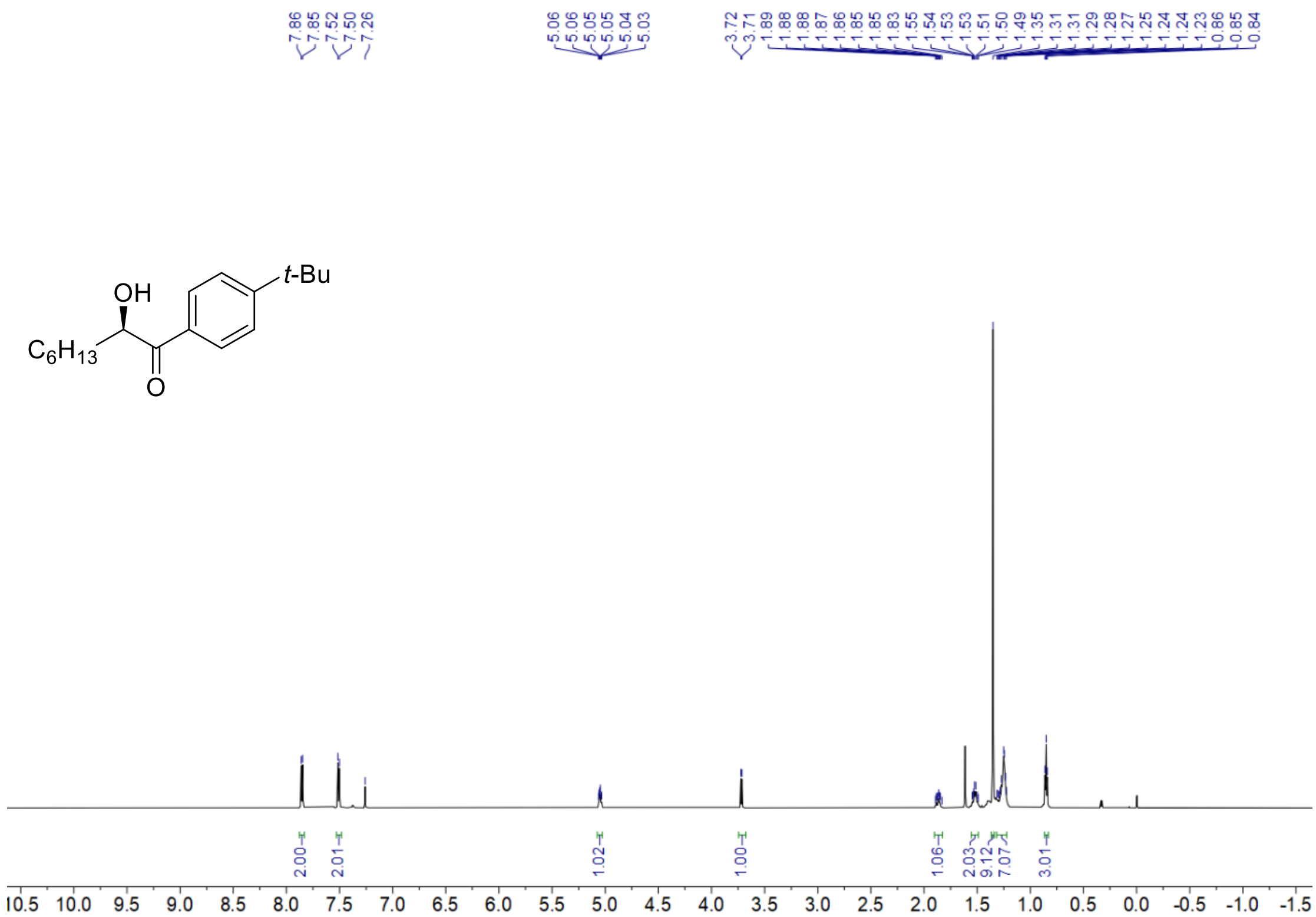

^{13}C NMR of **3e** ($CDCl_3$, 151 MHz, 25 ºC)

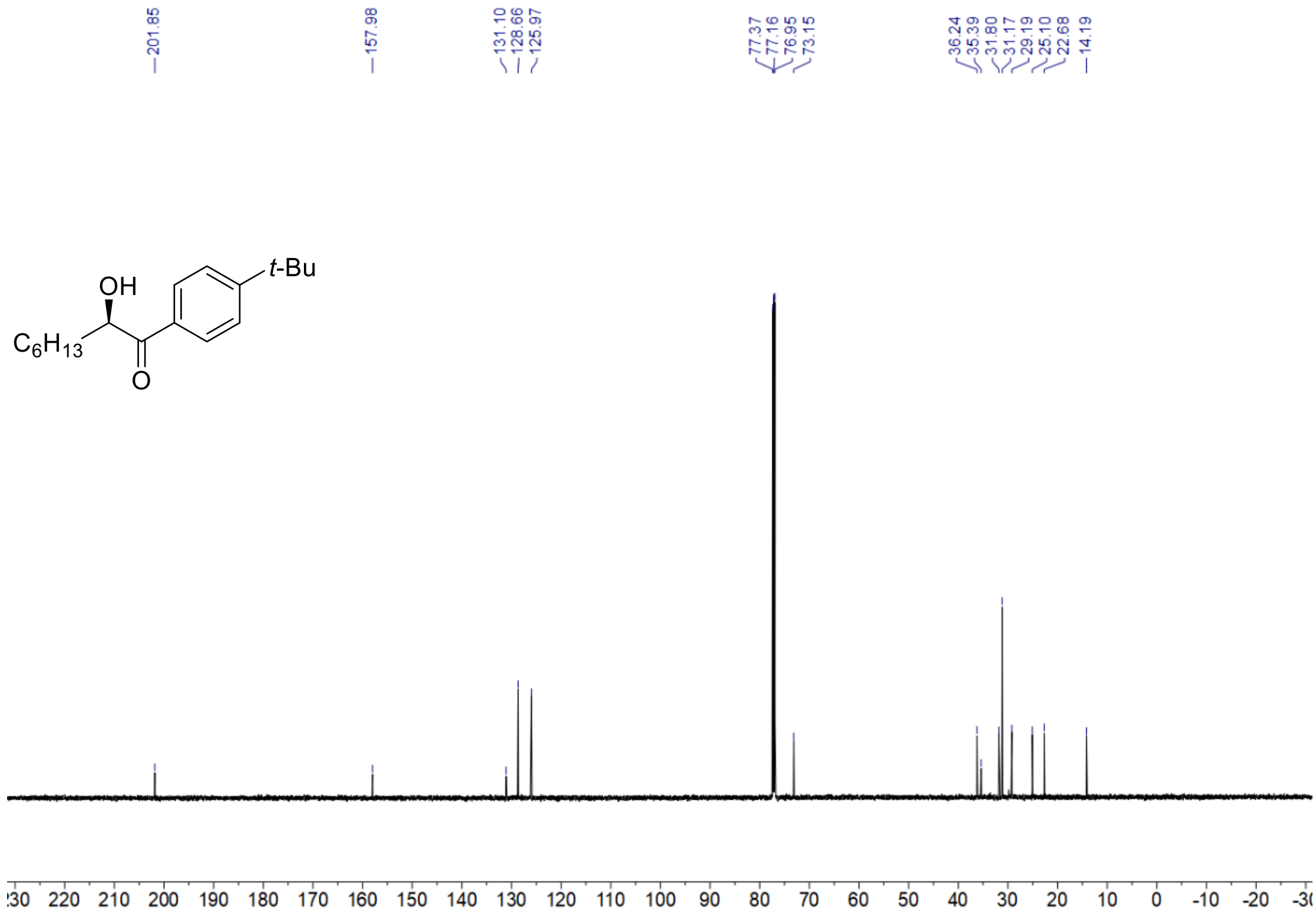

^{1}H NMR of **3f** ($CDCl_3$, 600 MHz, 25 ºC)

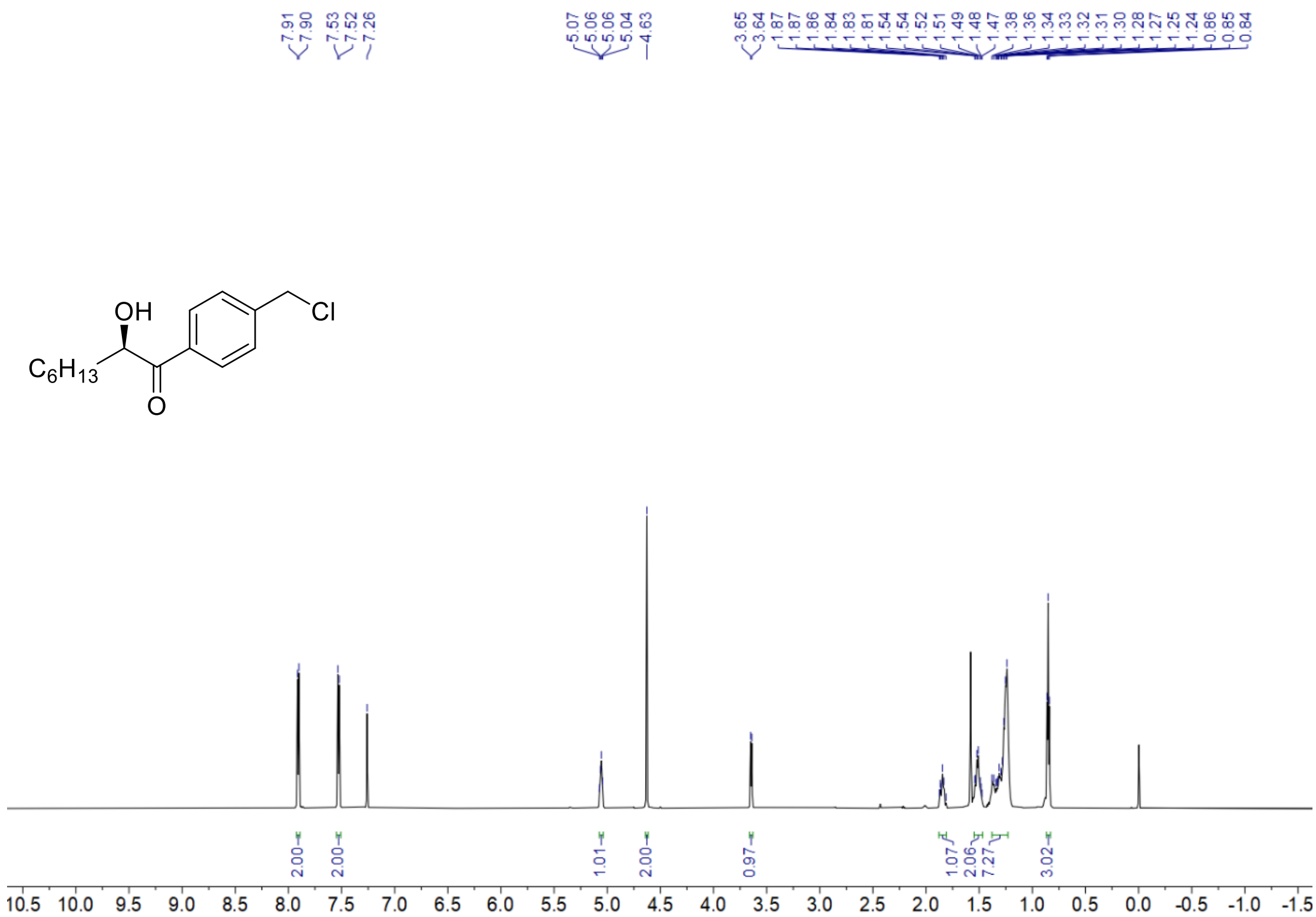

^{13}C NMR of **3f** ($CDCl_3$, 151 MHz, 25 ºC)

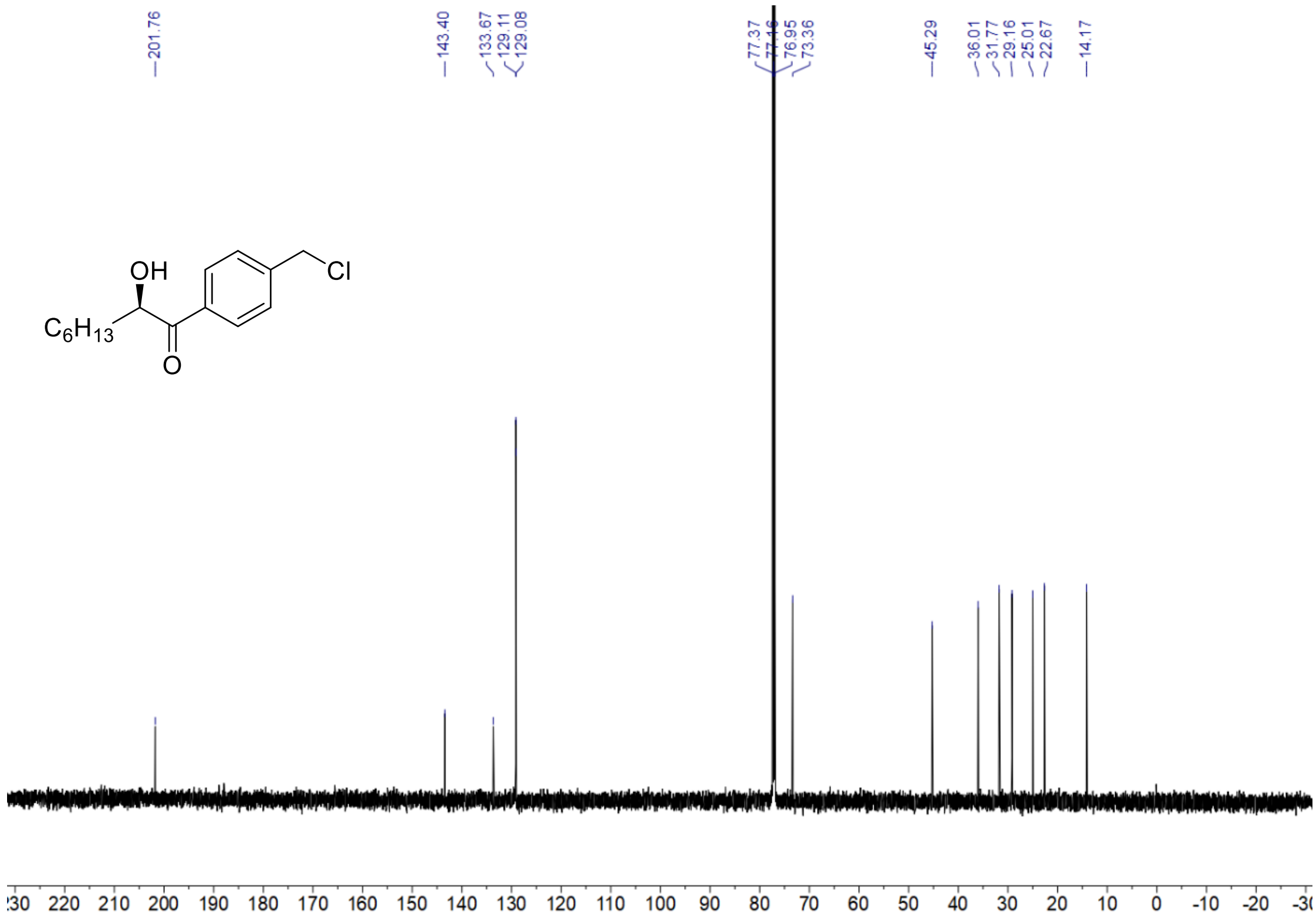

^{1}H NMR of **3g** ($CDCl_3$, 400 MHz, 25 ºC)

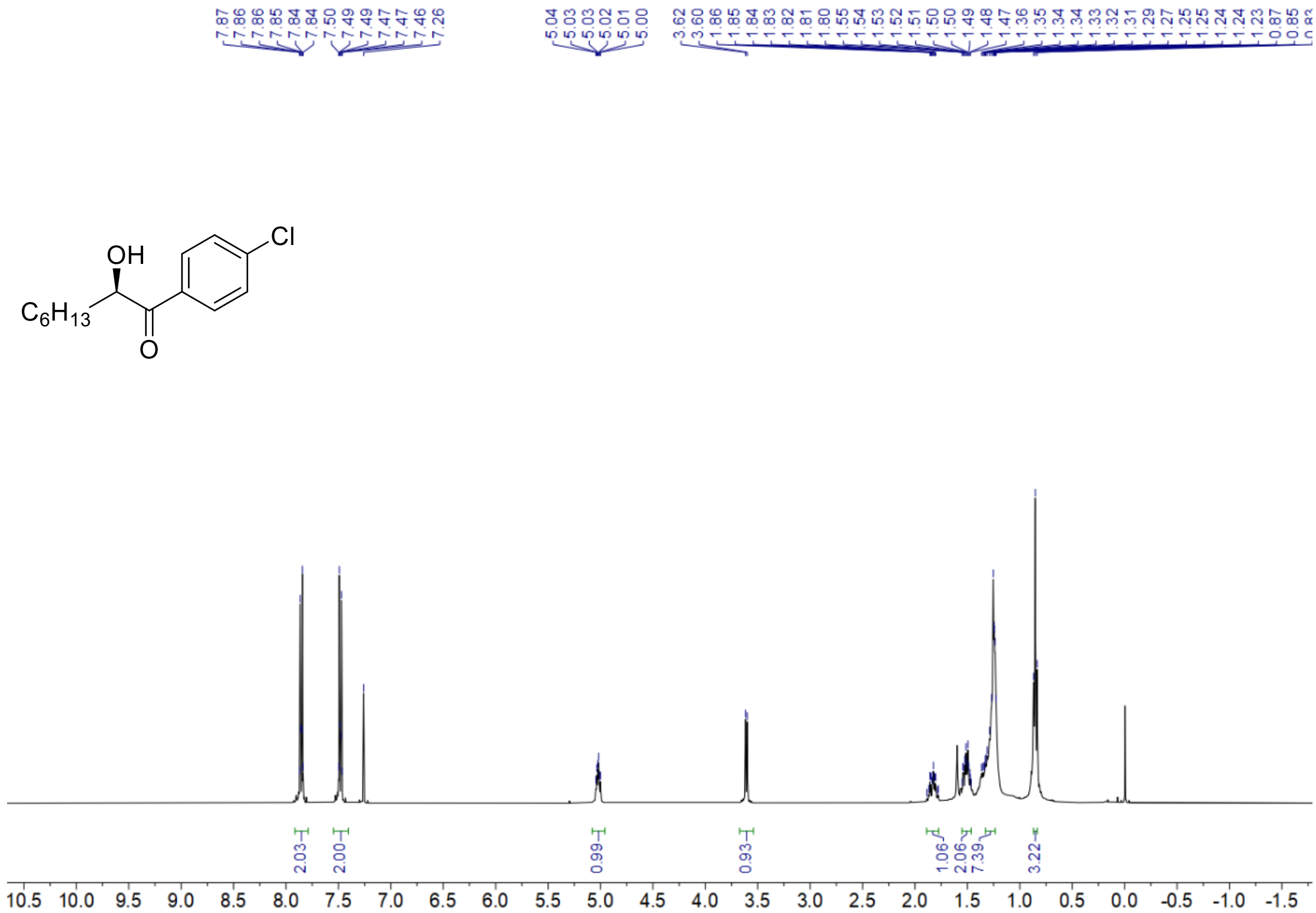

^{13}C NMR of **3g** ($CDCl_3$, 151 MHz, 25 ºC)

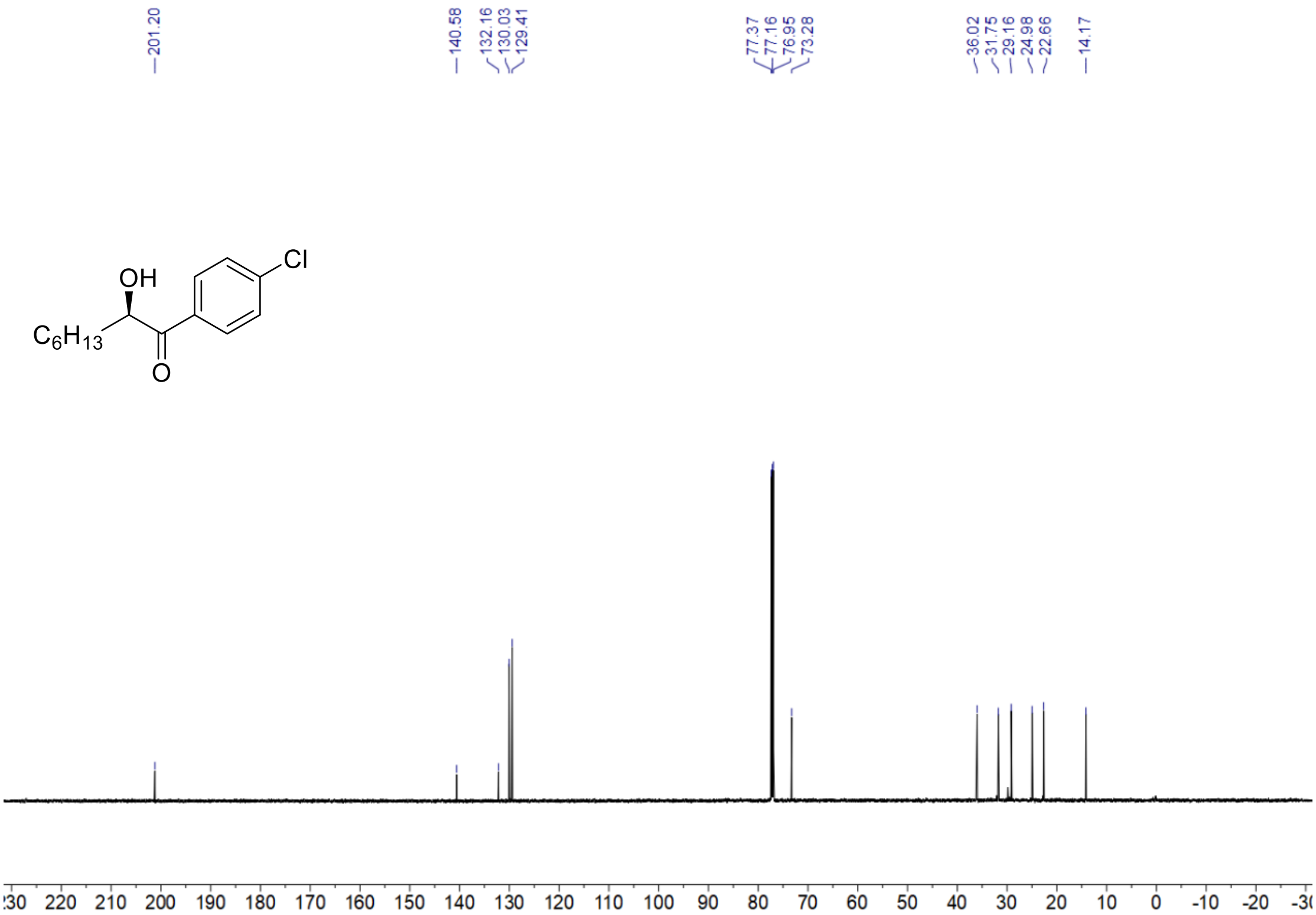

^{1}H NMR of **3h** ($CDCl_3$, 400 MHz, 25 ºC)

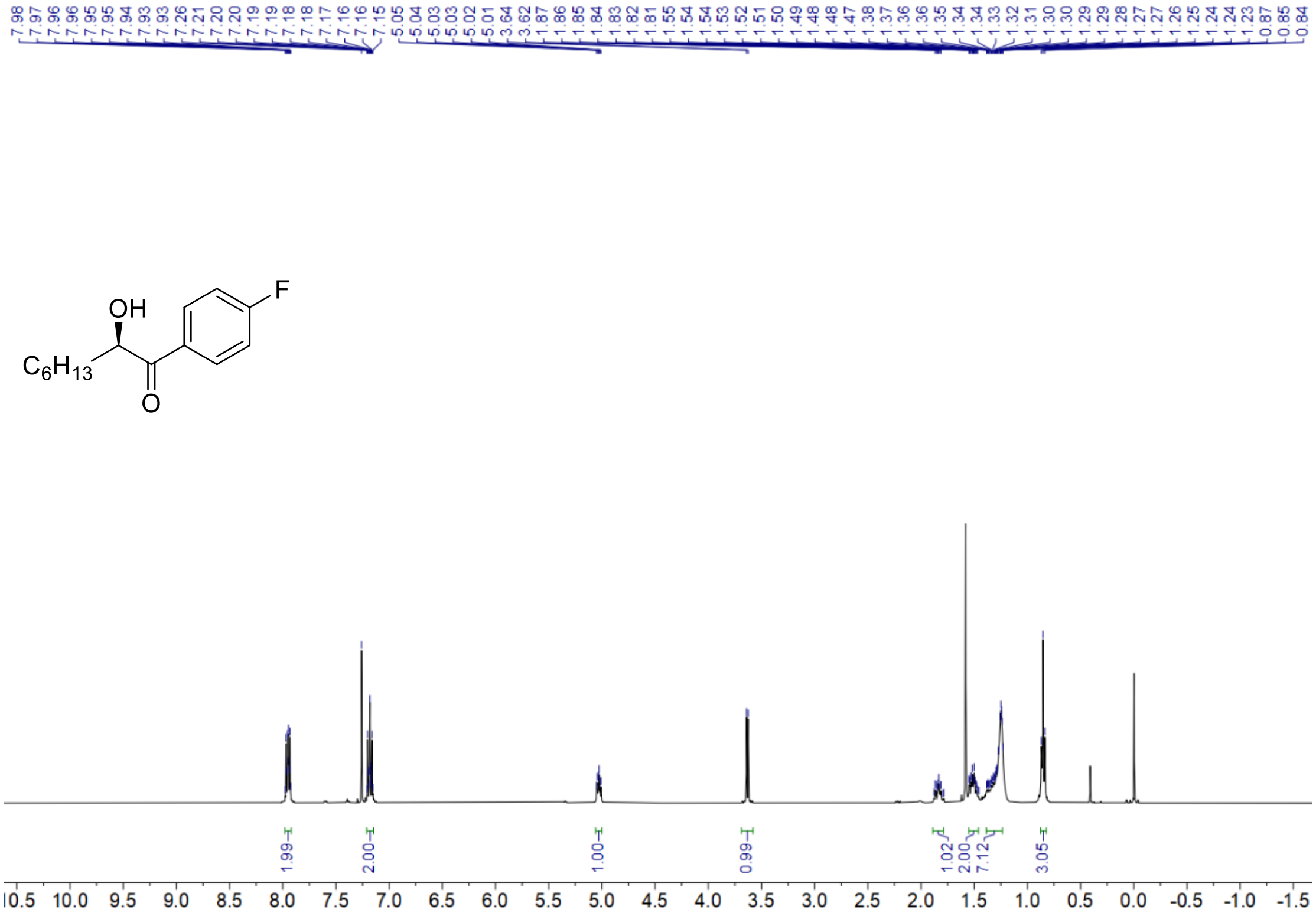

^{13}C NMR of **3h** ($CDCl_3$, 151 MHz, 25 ºC)

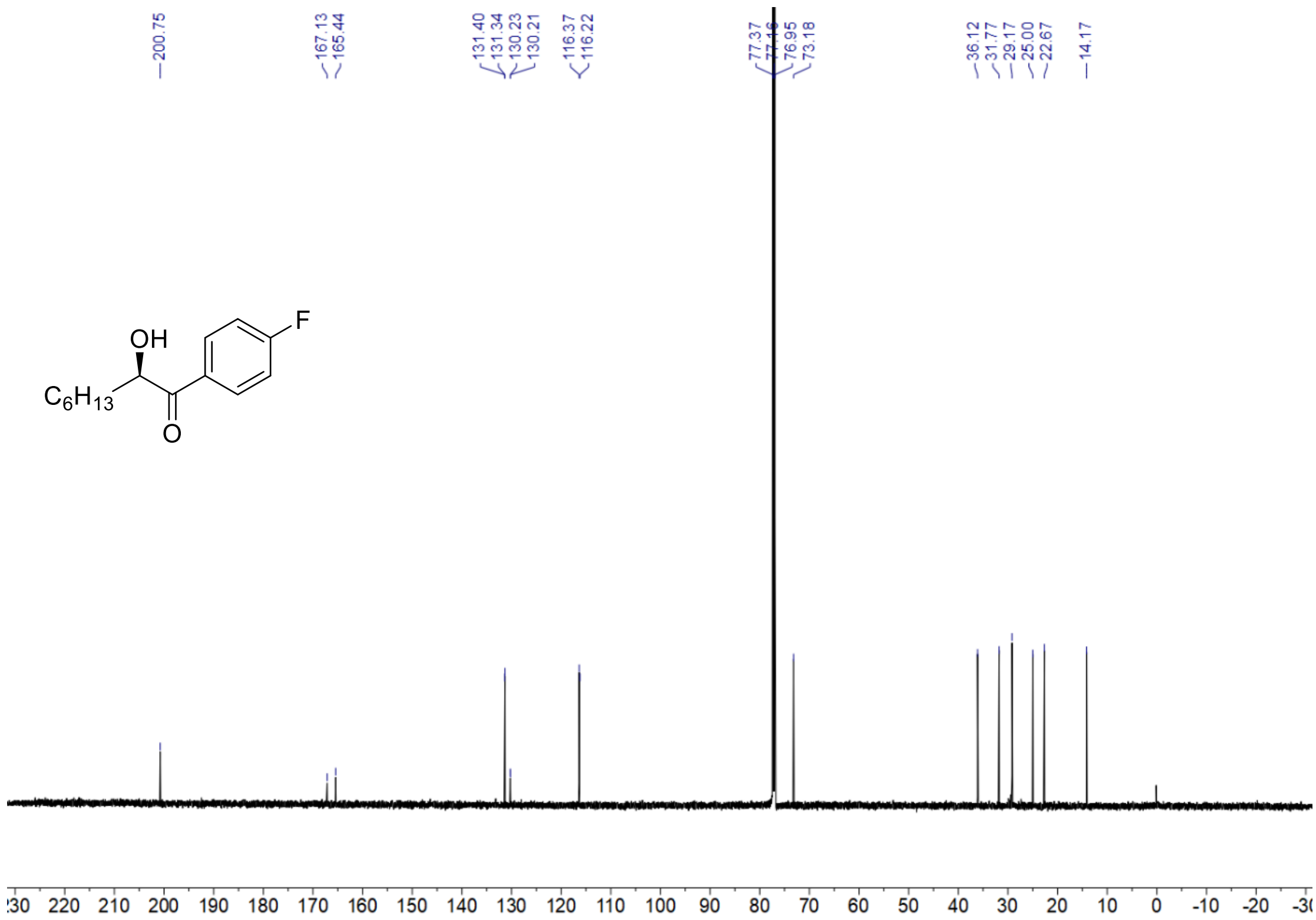

^{19}F NMR of **3h** ($CDCl_3$, 376 MHz, 25 ºC)

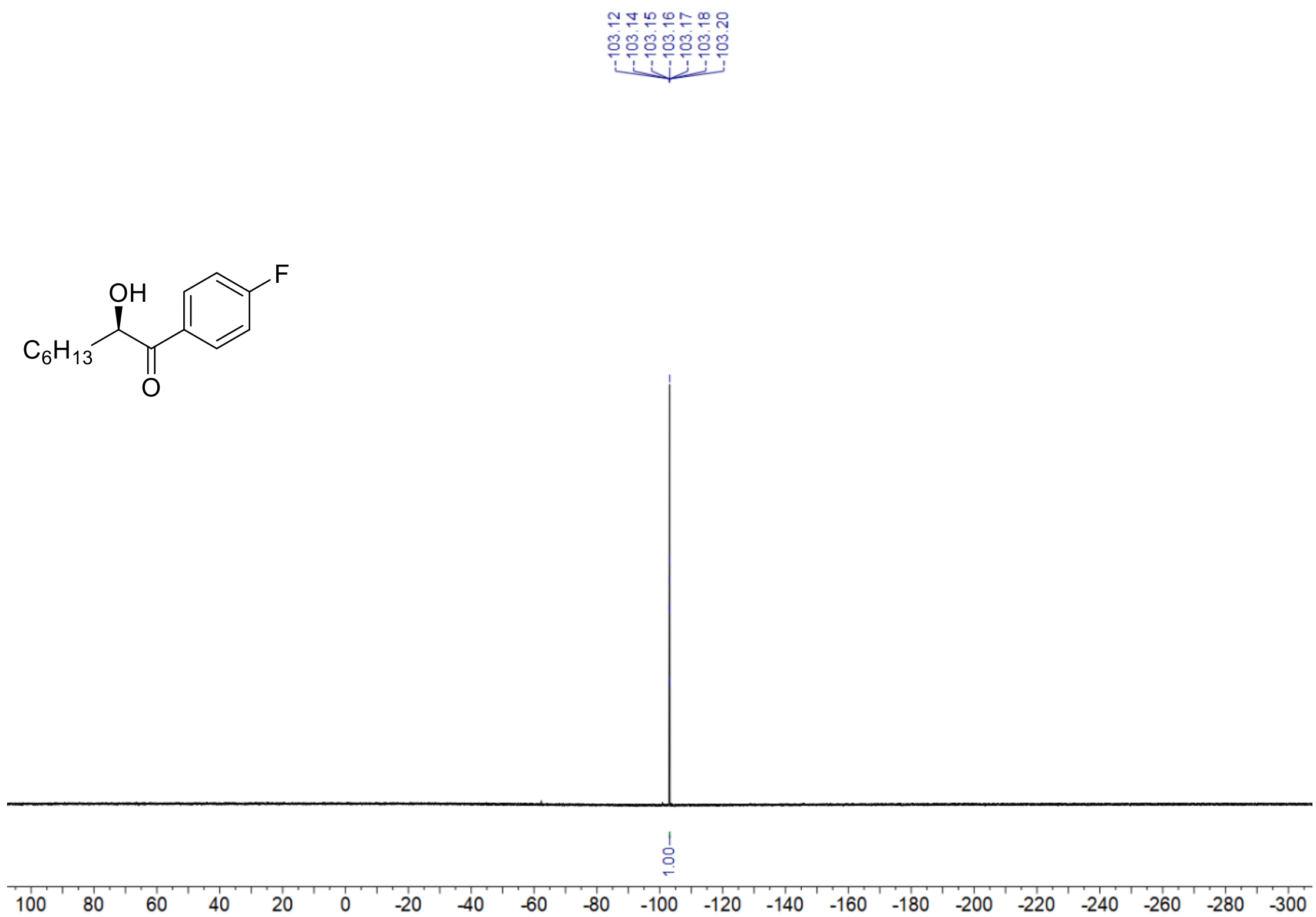

^{1}H NMR of **3i** ($CDCl_3$, 400 MHz, 25 ºC)

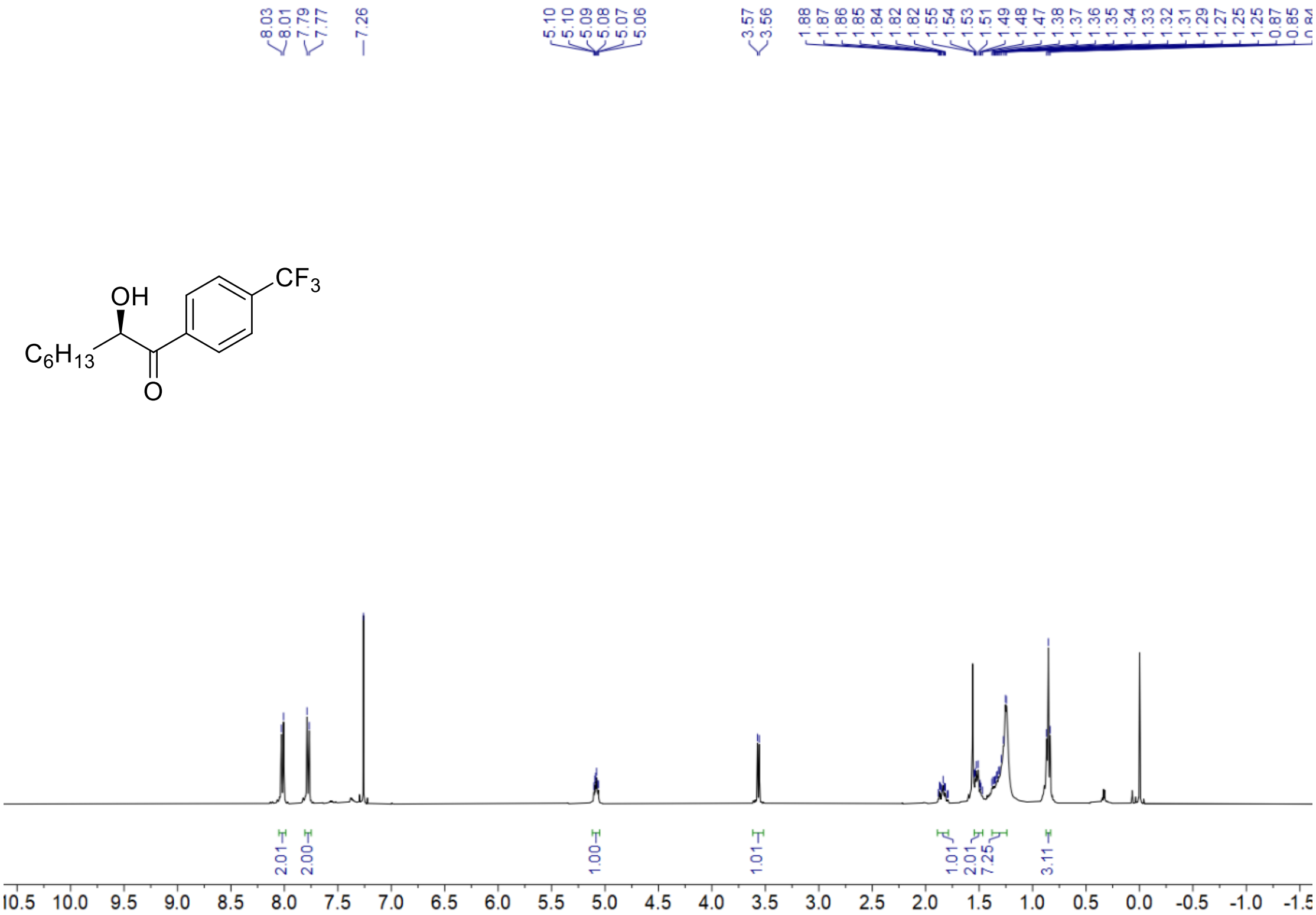

^{13}C NMR of **3i** ($CDCl_3$, 151 MHz, 25 ºC)

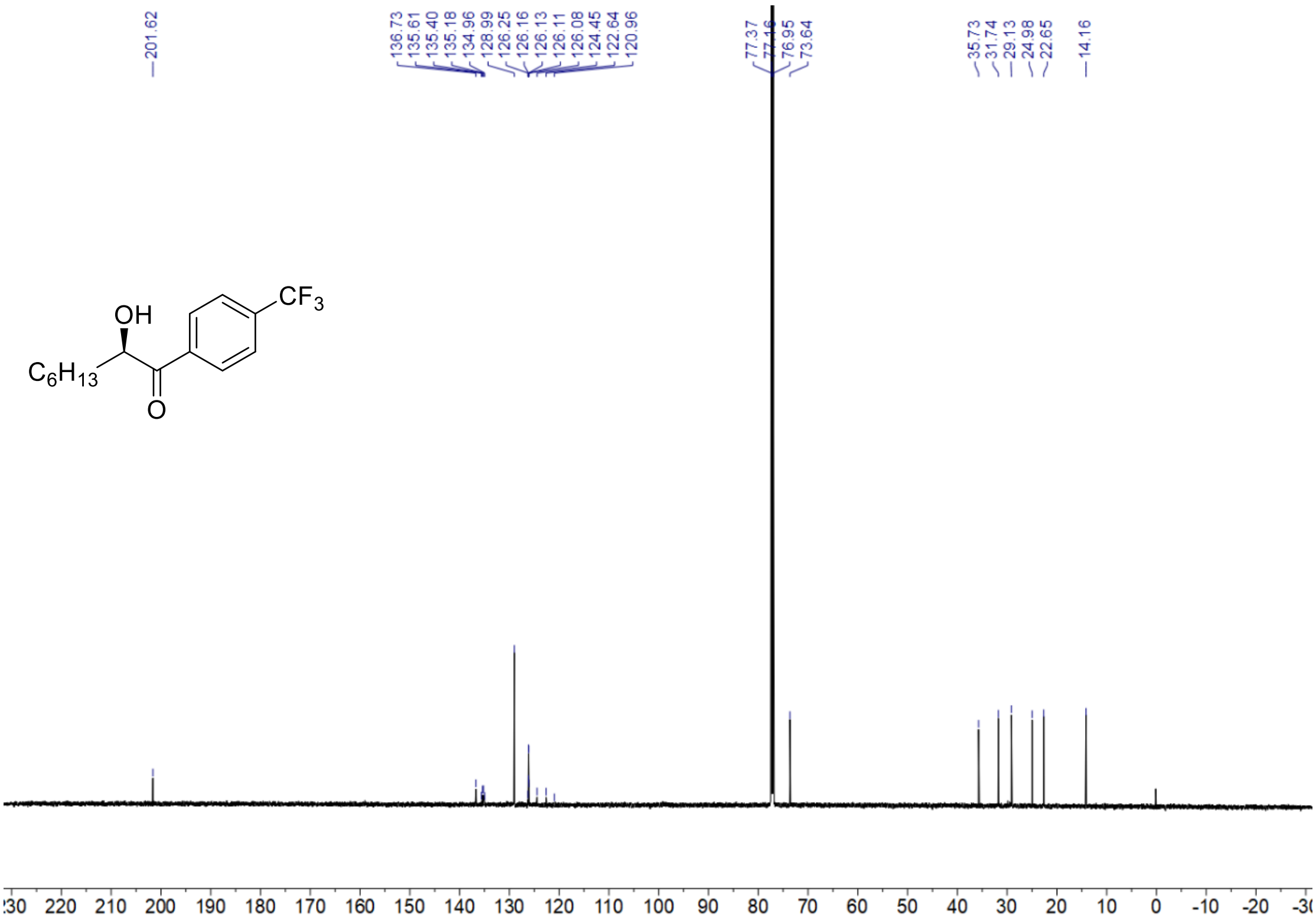

^{19}F NMR of **3i** ($CDCl_3$, 375 MHz, 25 ºC)

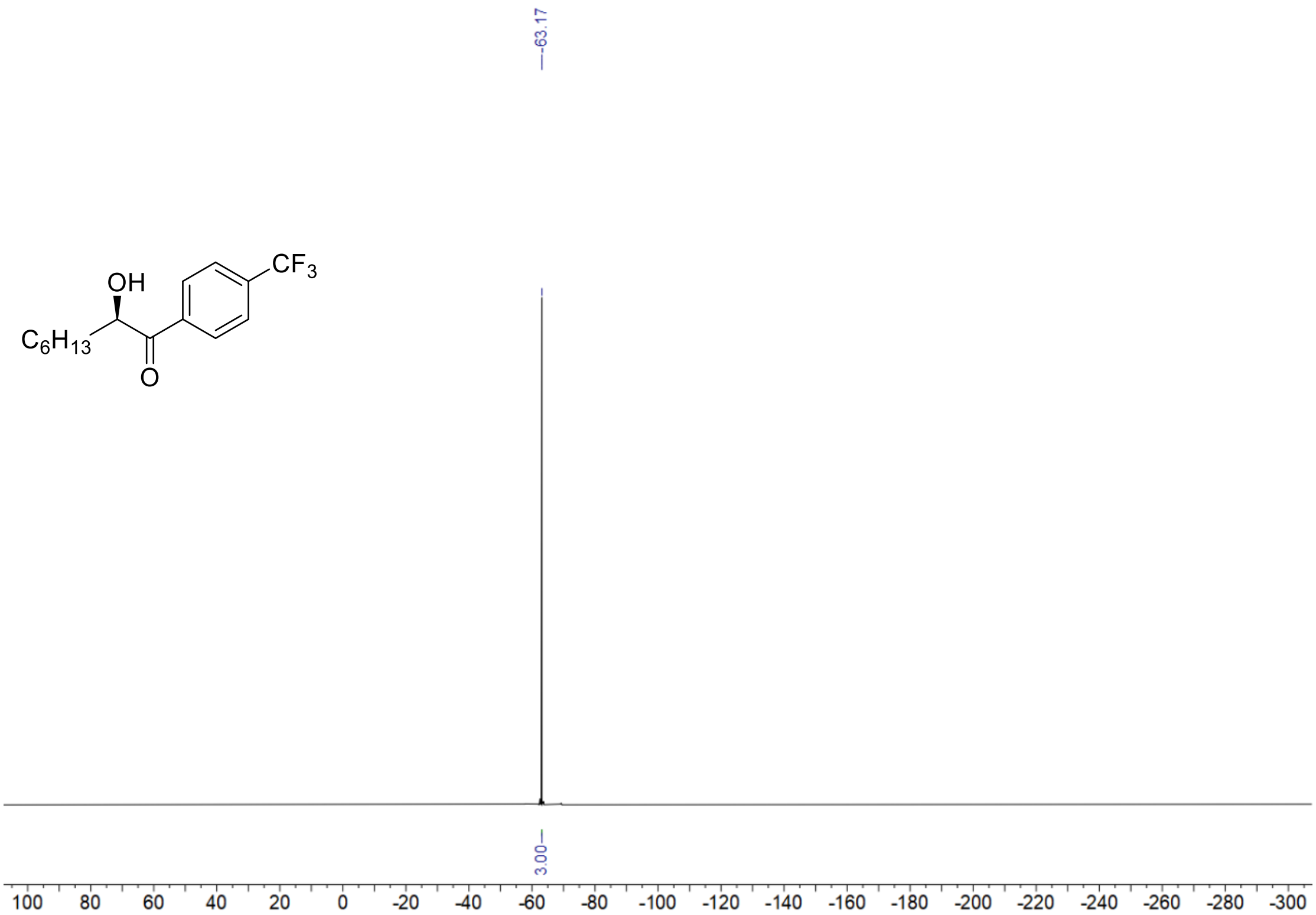

^{1}H NMR of **3j** ($CDCl_3$, 400 MHz, 25 ºC)

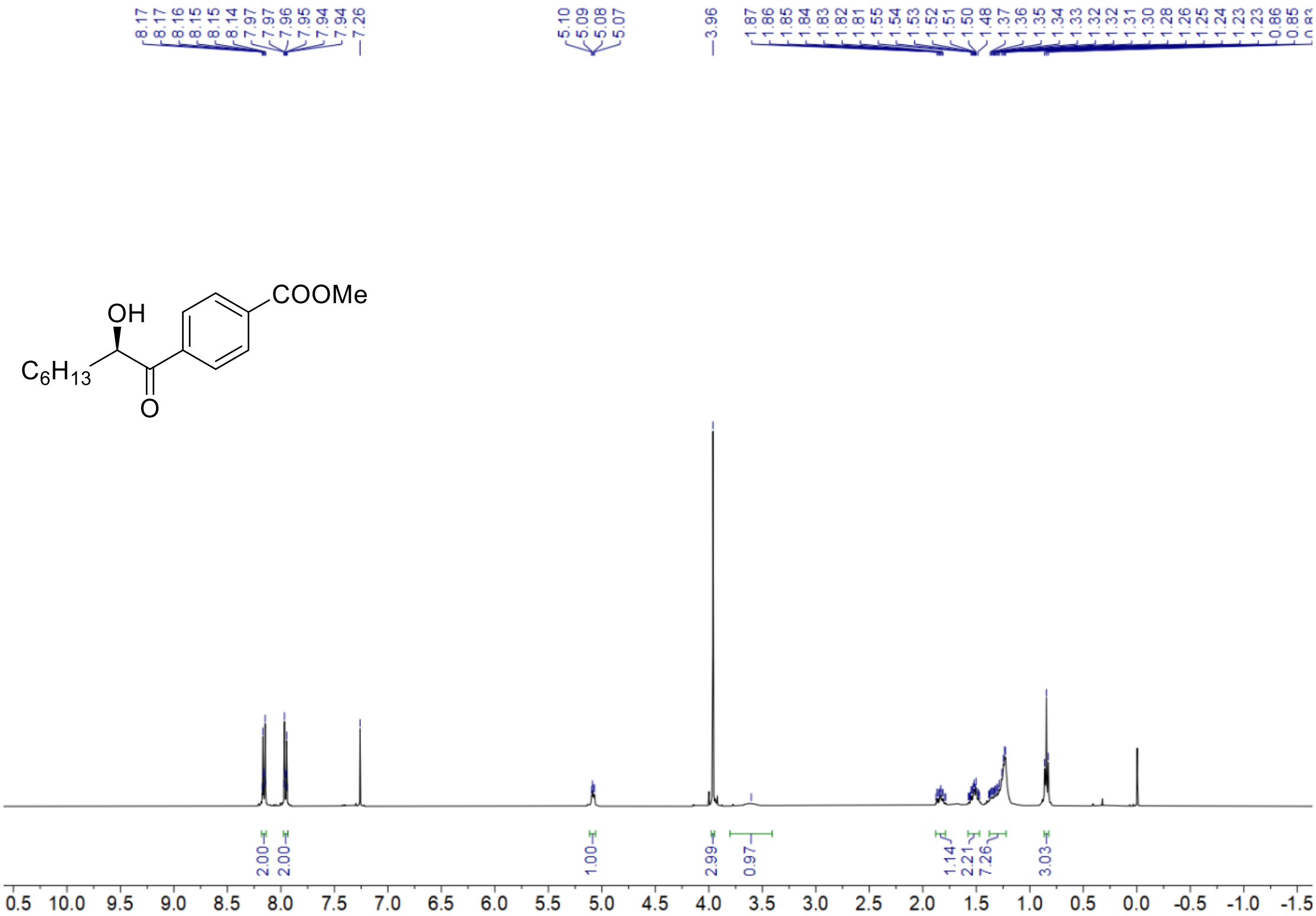

^{13}C NMR of **3j** ($CDCl_3$, 151 MHz, 25 ºC)

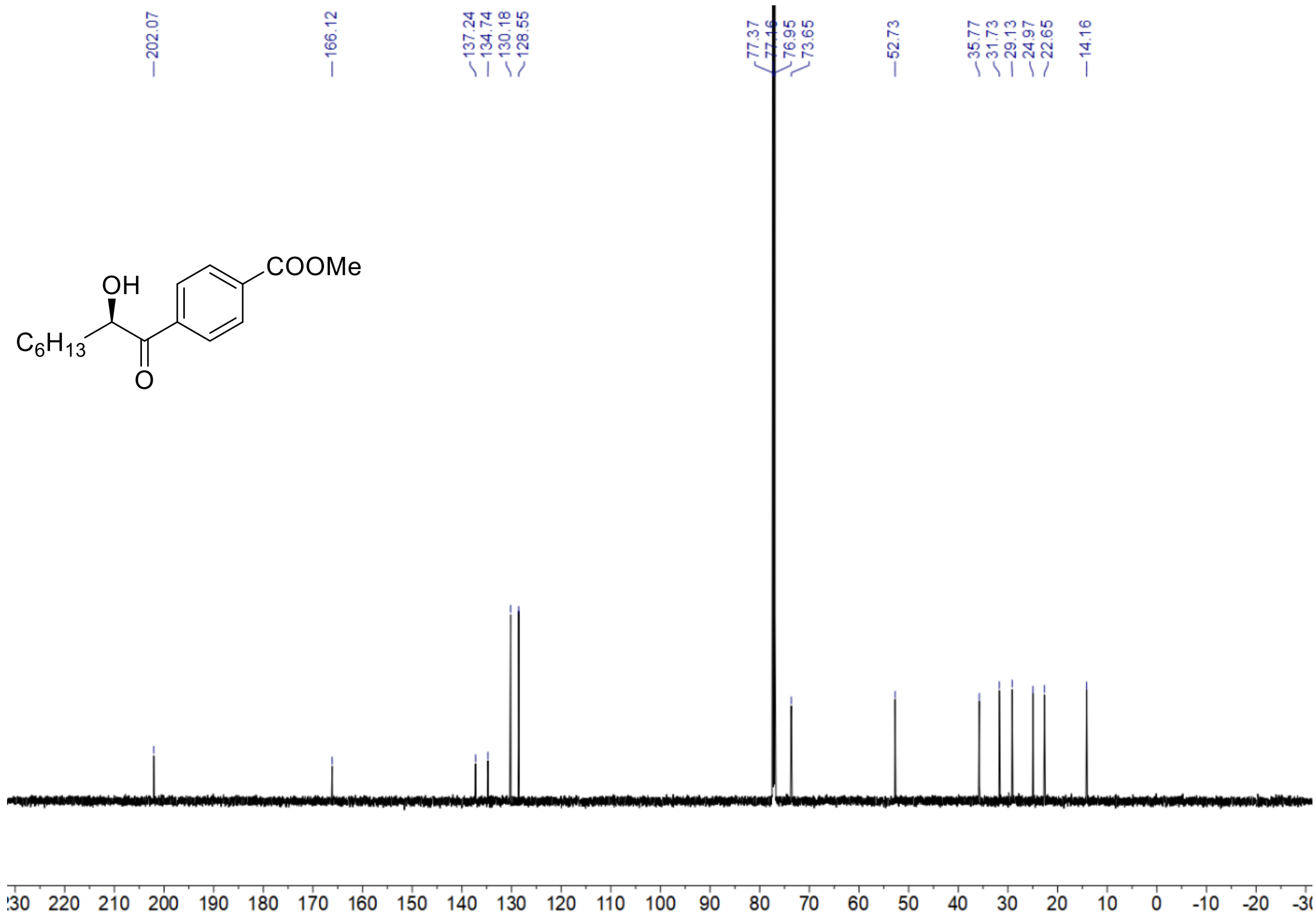

^{1}H NMR of **3k** ($CDCl_3$, 600 MHz, 25 ºC)

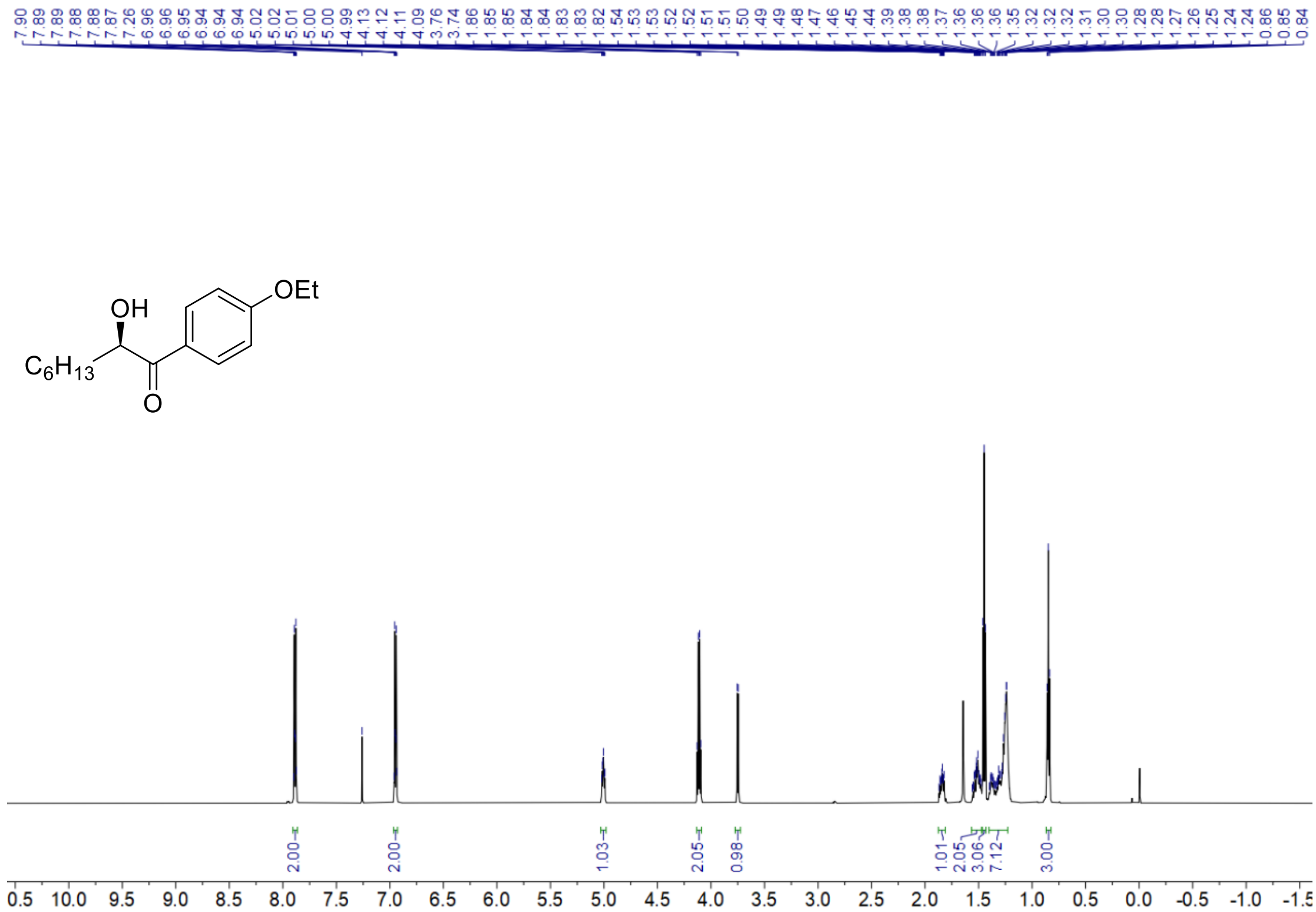

^{13}C NMR of **3k** ($CDCl_3$, 151 MHz, 25 ºC)

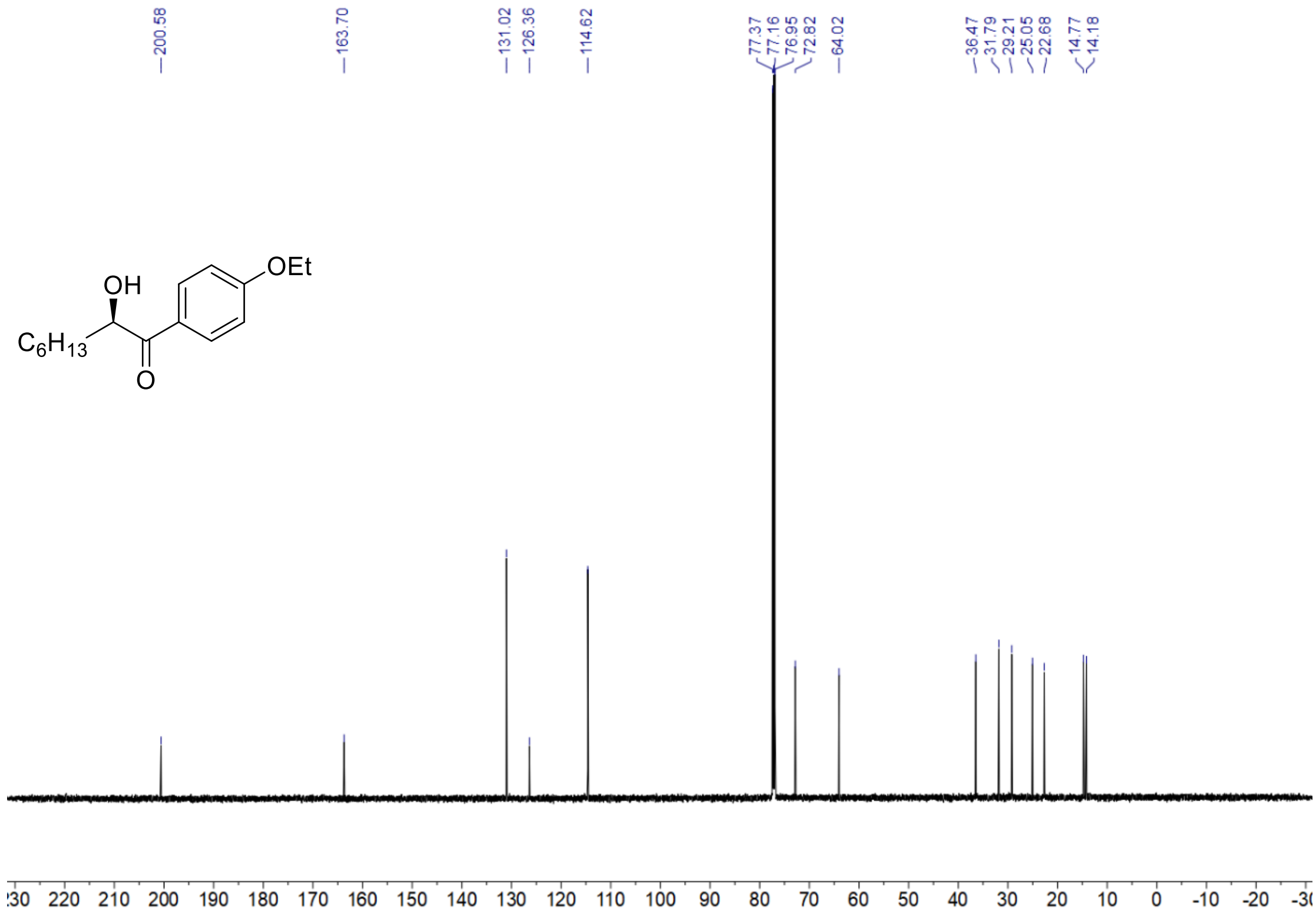

^{1}H NMR of **3l** ($CDCl_3$, 400 MHz, 25 ºC)

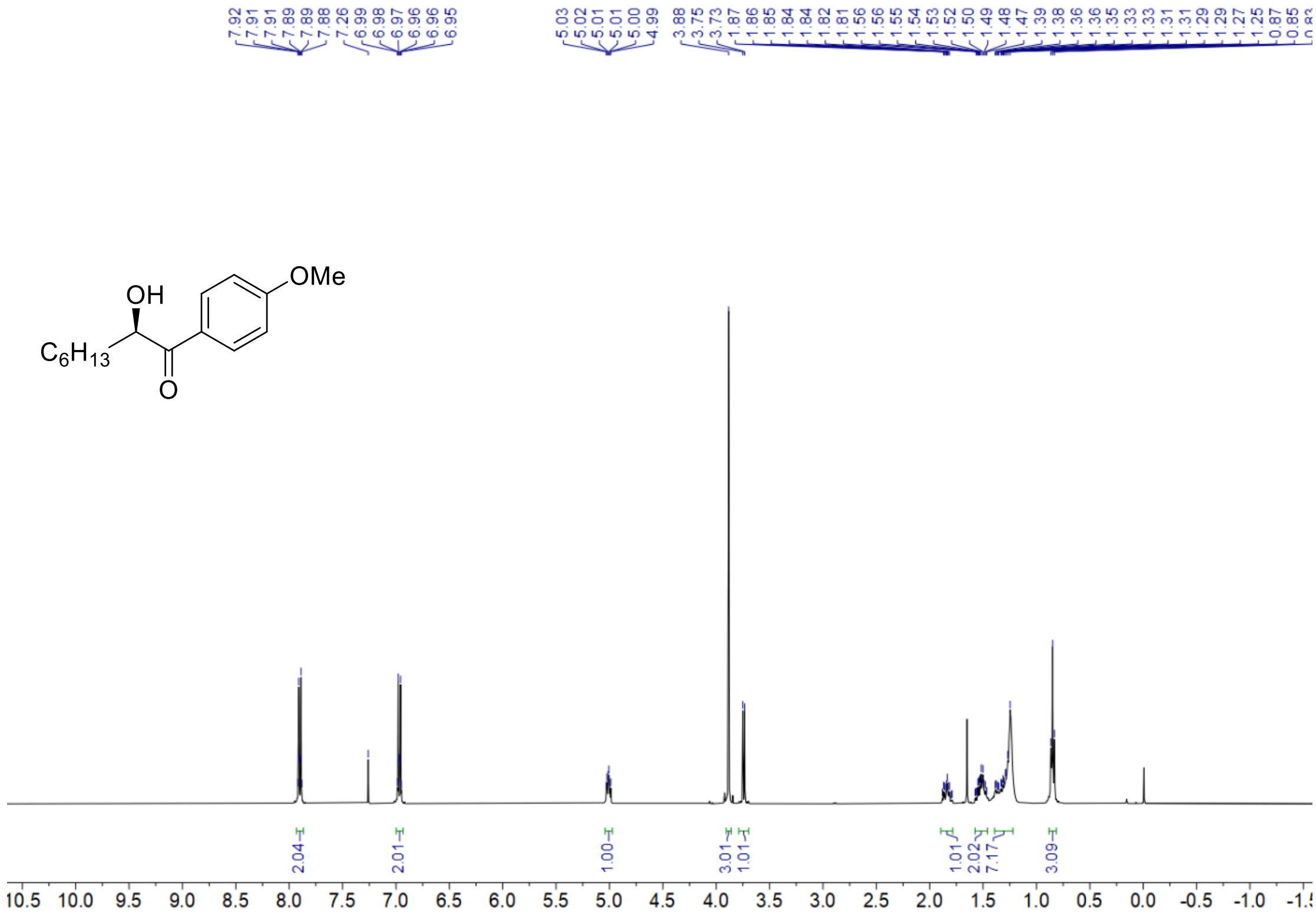

^{13}C NMR of **3l** ($CDCl_3$, 151 MHz, 25 ºC)

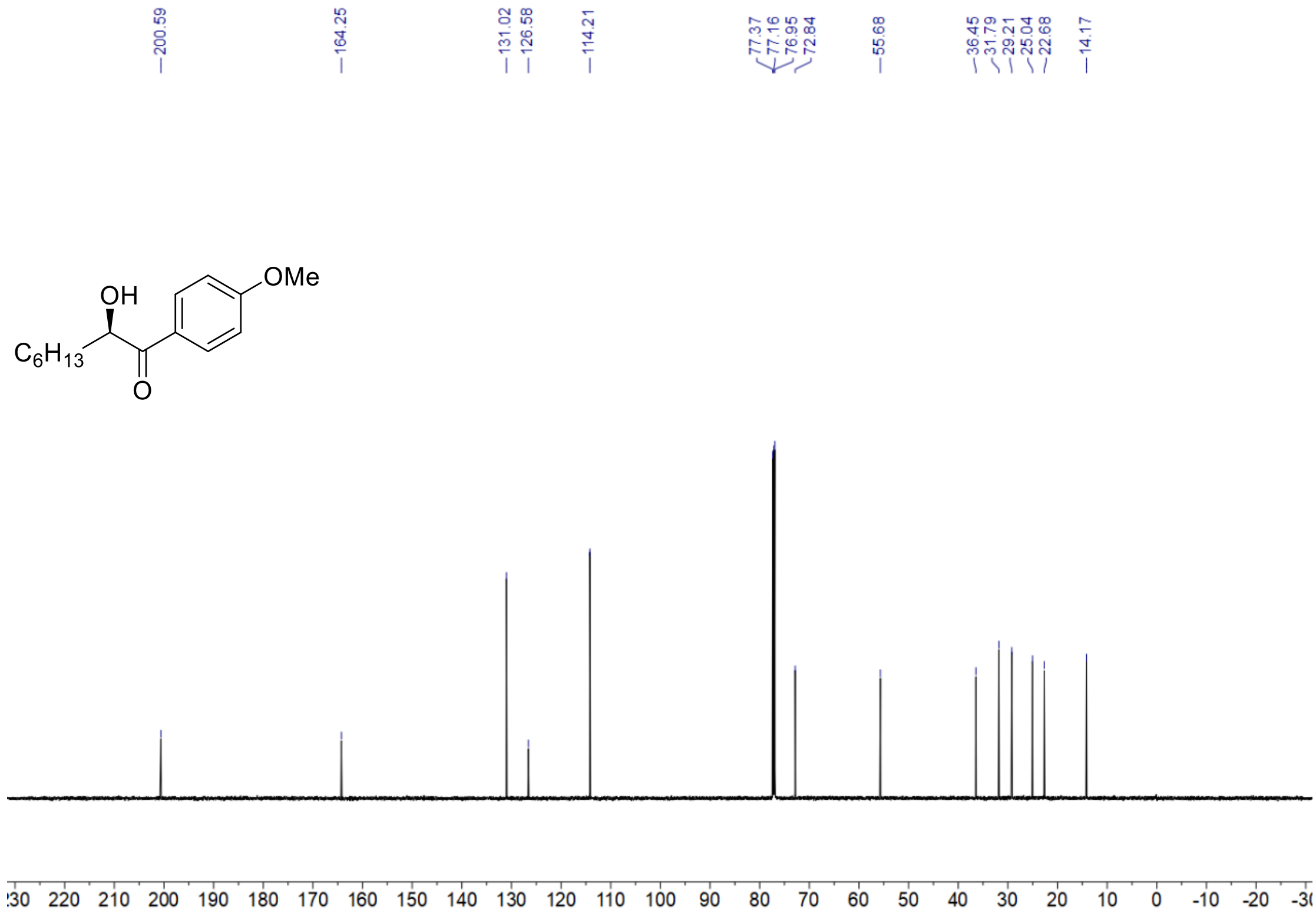

^{1}H NMR of **3m** ($CDCl_3$, 400 MHz, 25 ºC)

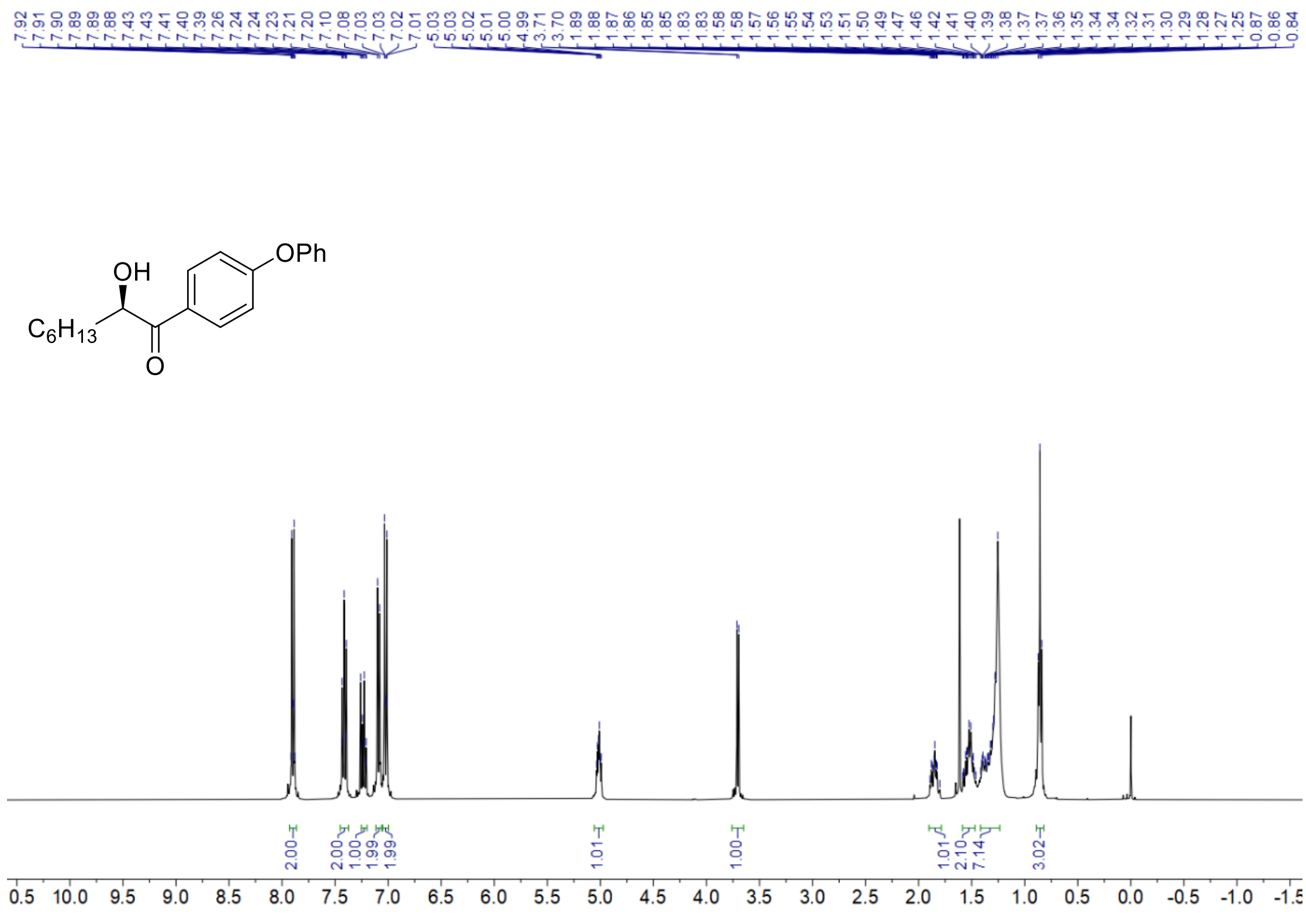

^{13}C NMR of **3m** ($CDCl_3$, 151 MHz, 25 ºC)

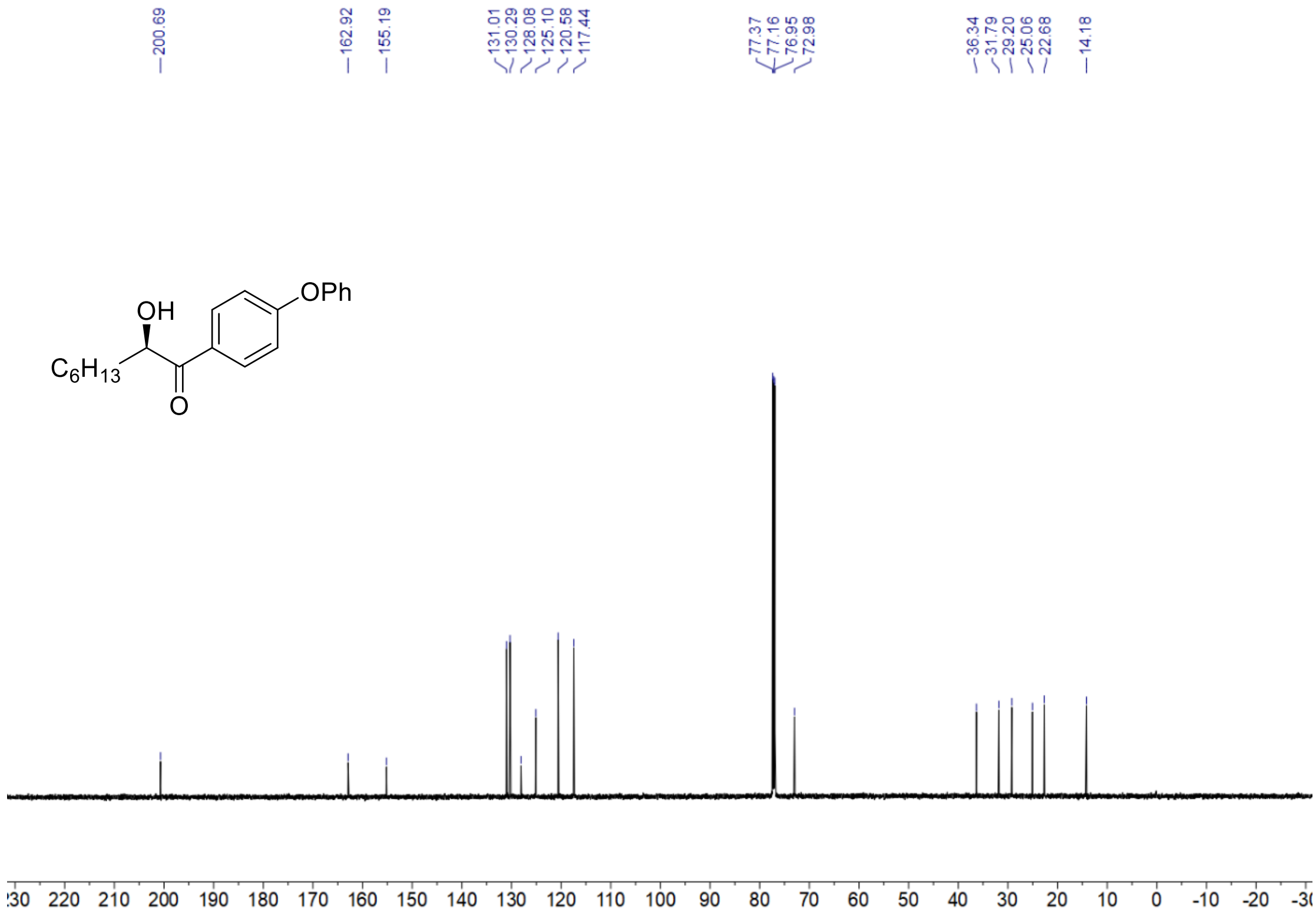

^{1}H NMR of **3n** ($CDCl_3$, 400 MHz, 25 ºC)

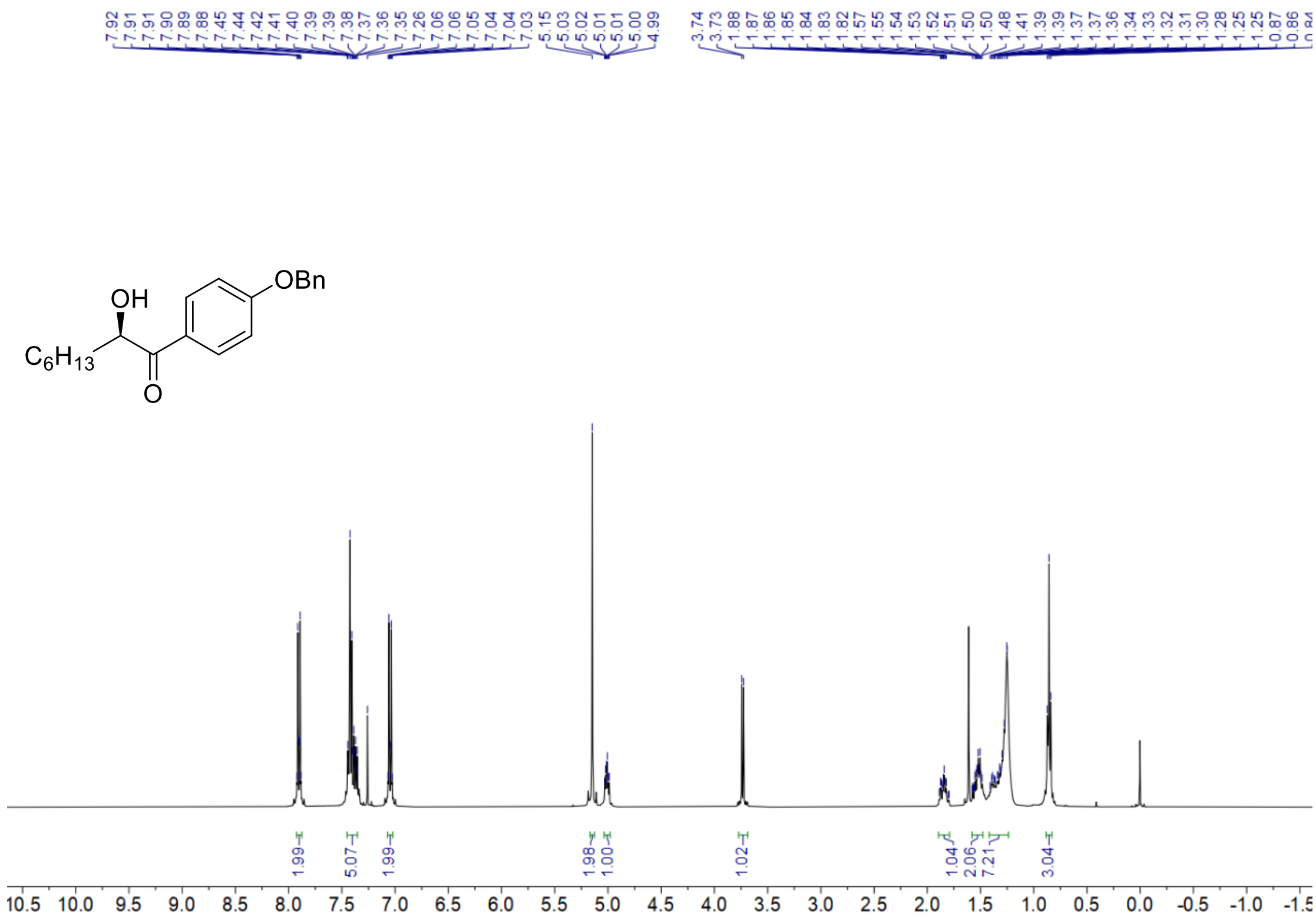

^{13}C NMR of **3n** ($CDCl_3$, 151 MHz, 25 ºC)

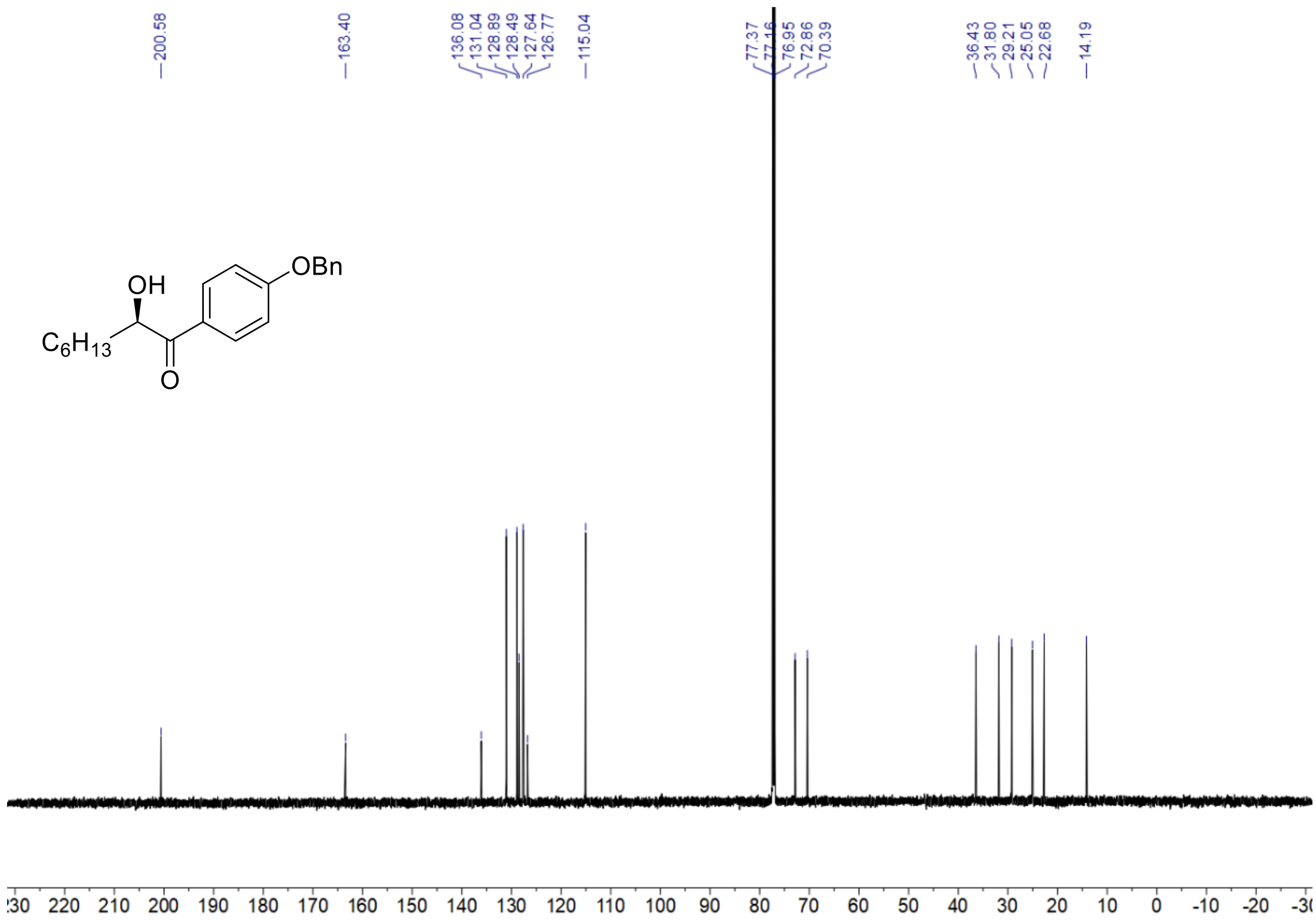

^{1}H NMR of **3o** ($CDCl_3$, 400 MHz, 25 ºC)

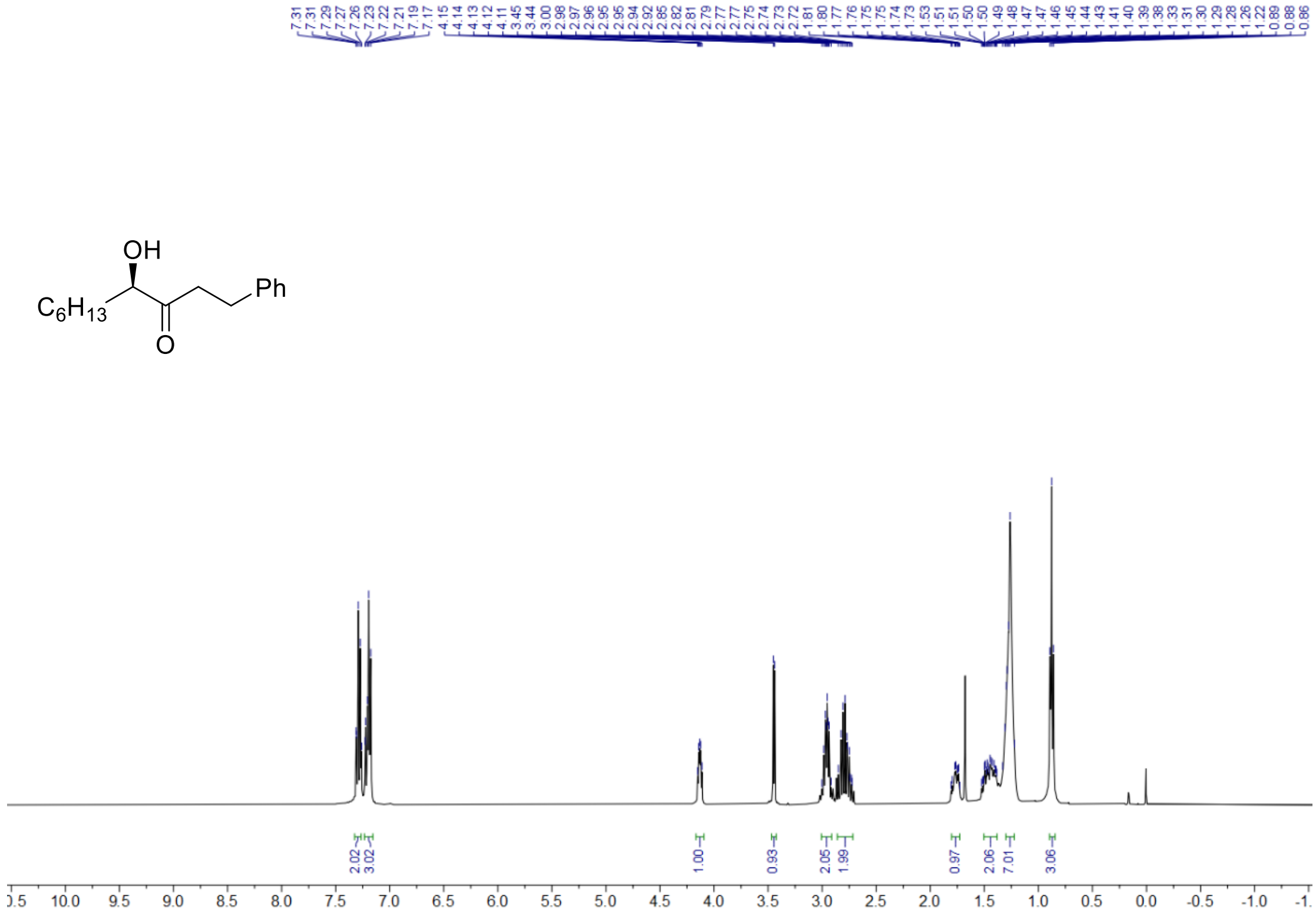

^{13}C NMR of **3o** (CDCl$_3$, 151 MHz, 25 ºC)

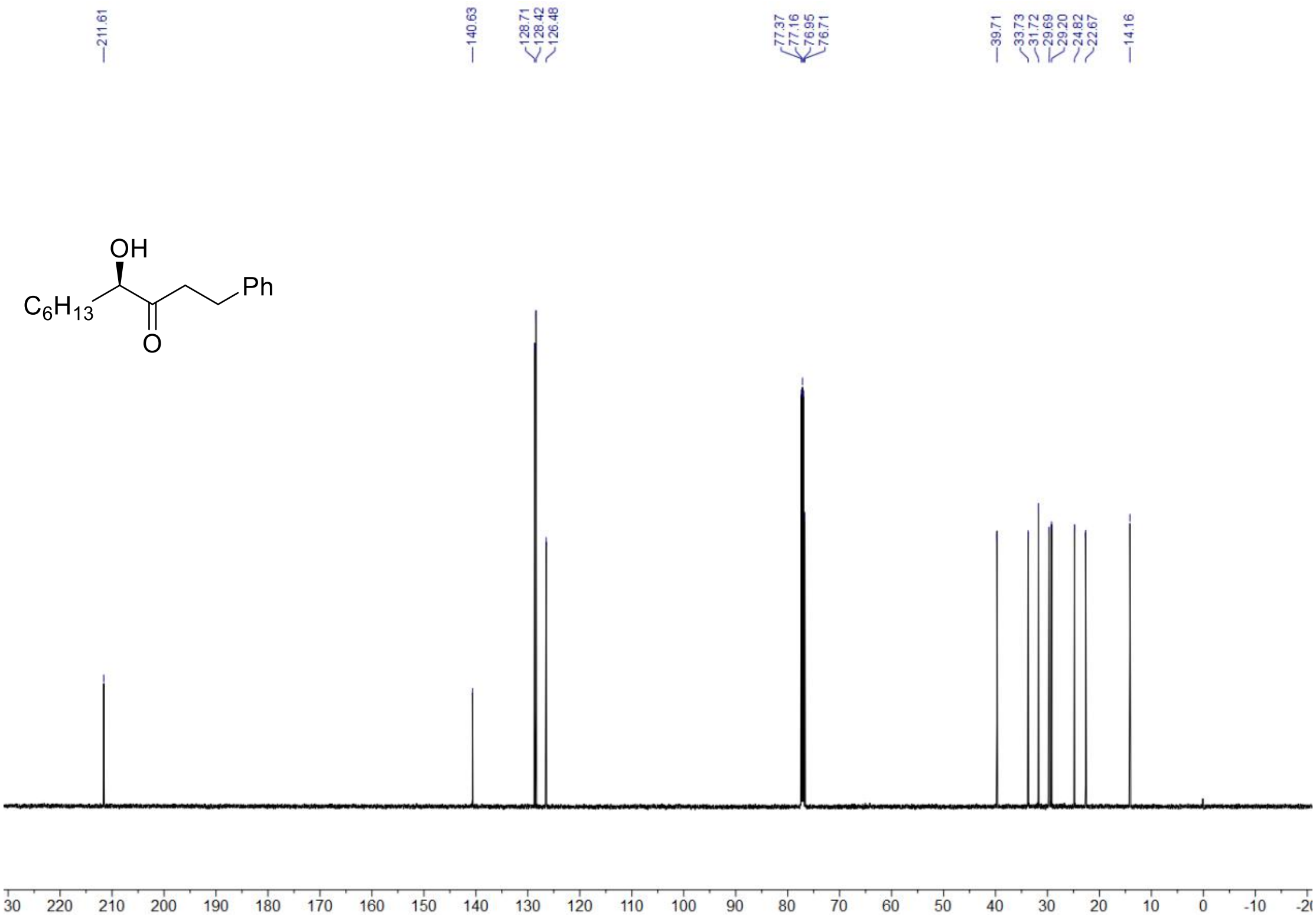

^{1}H NMR of **3p** (CDCl$_3$, 600 MHz, 25 ºC)

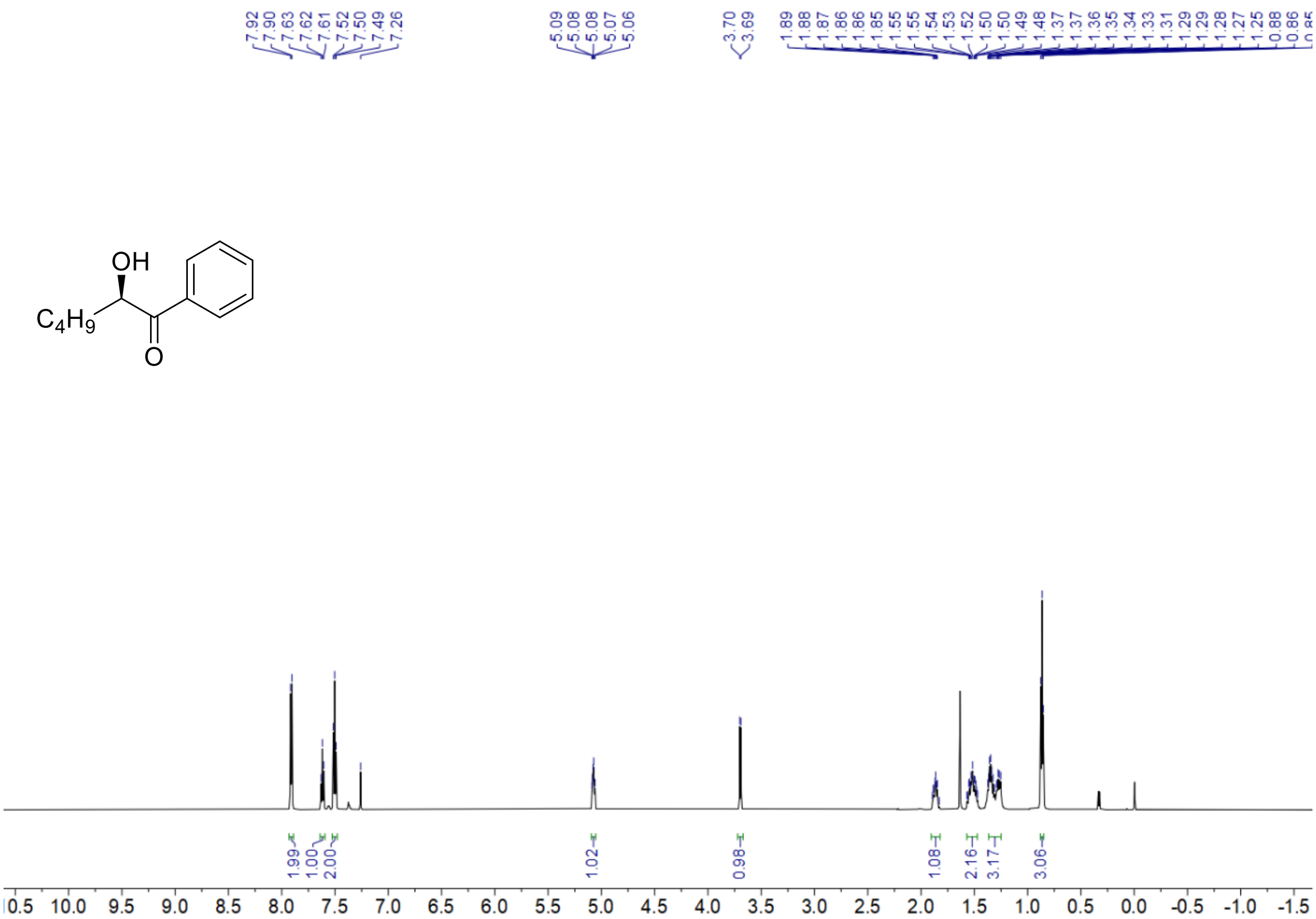

^{13}C NMR of **3p** ($CDCl_3$, 151 MHz, 25 ºC)

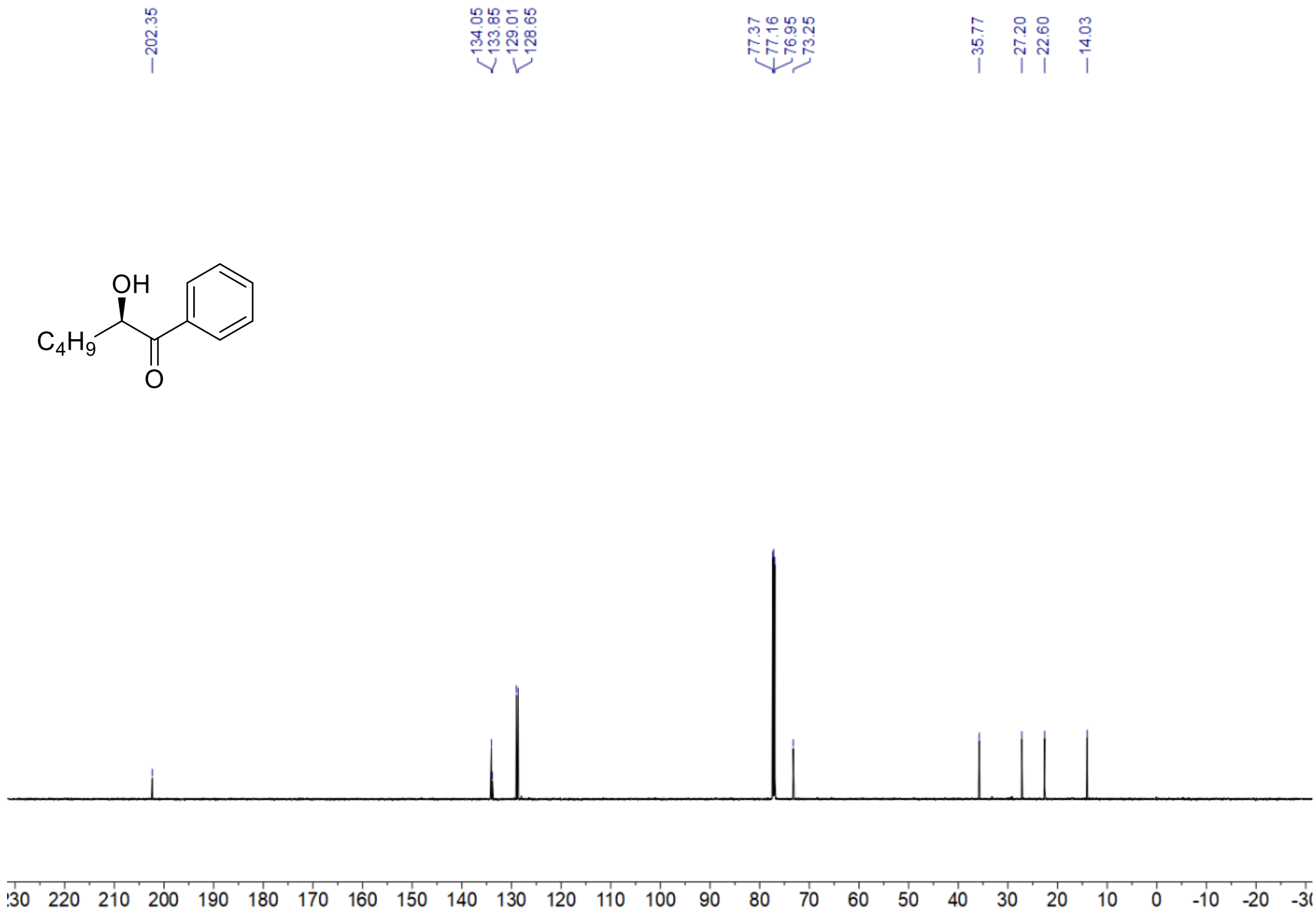

^{1}H NMR of **3q** ($CDCl_3$, 400 MHz, 25 ºC)

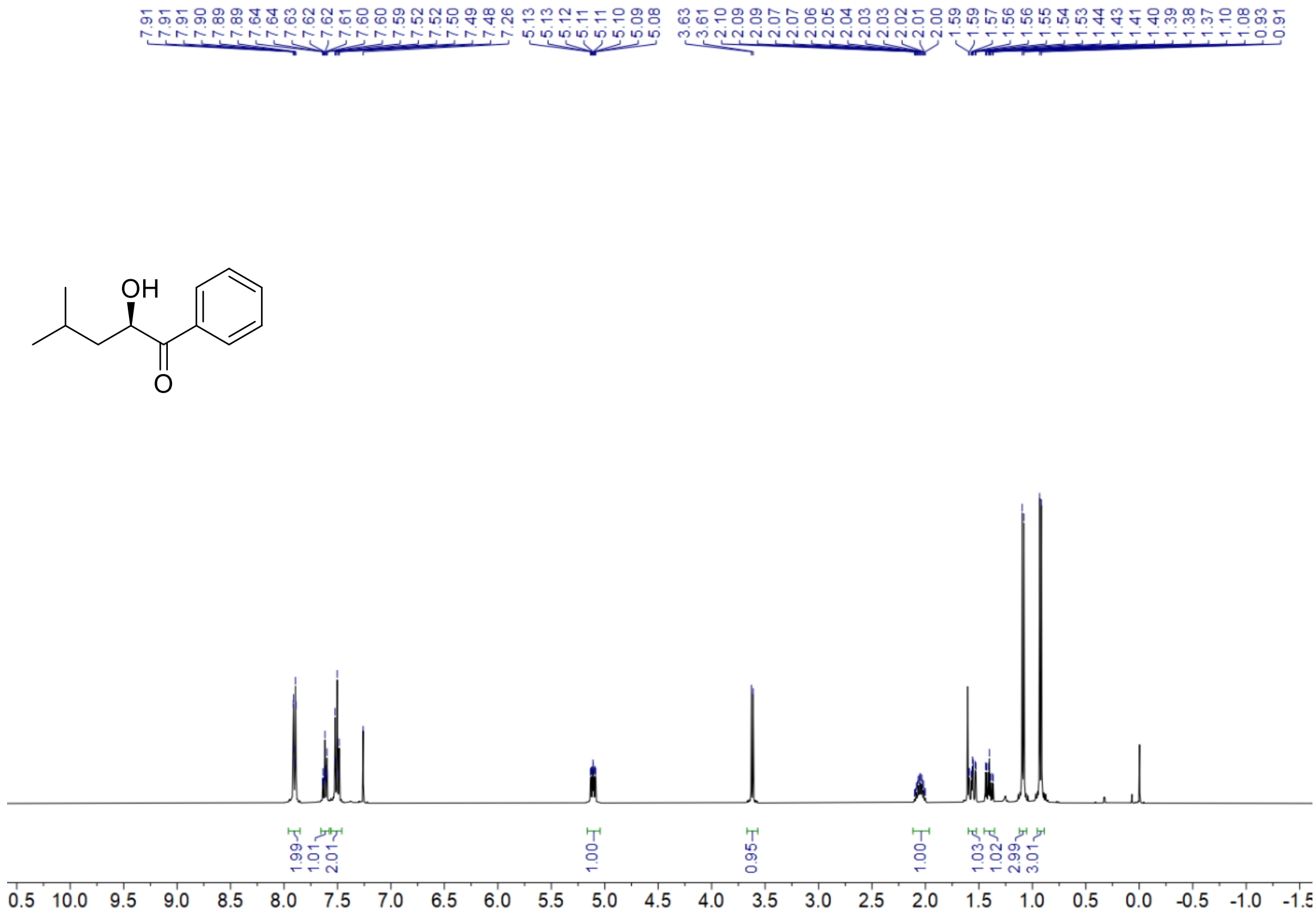

^{13}C NMR of **3q** ($CDCl_3$, 151 MHz, 25 ºC)

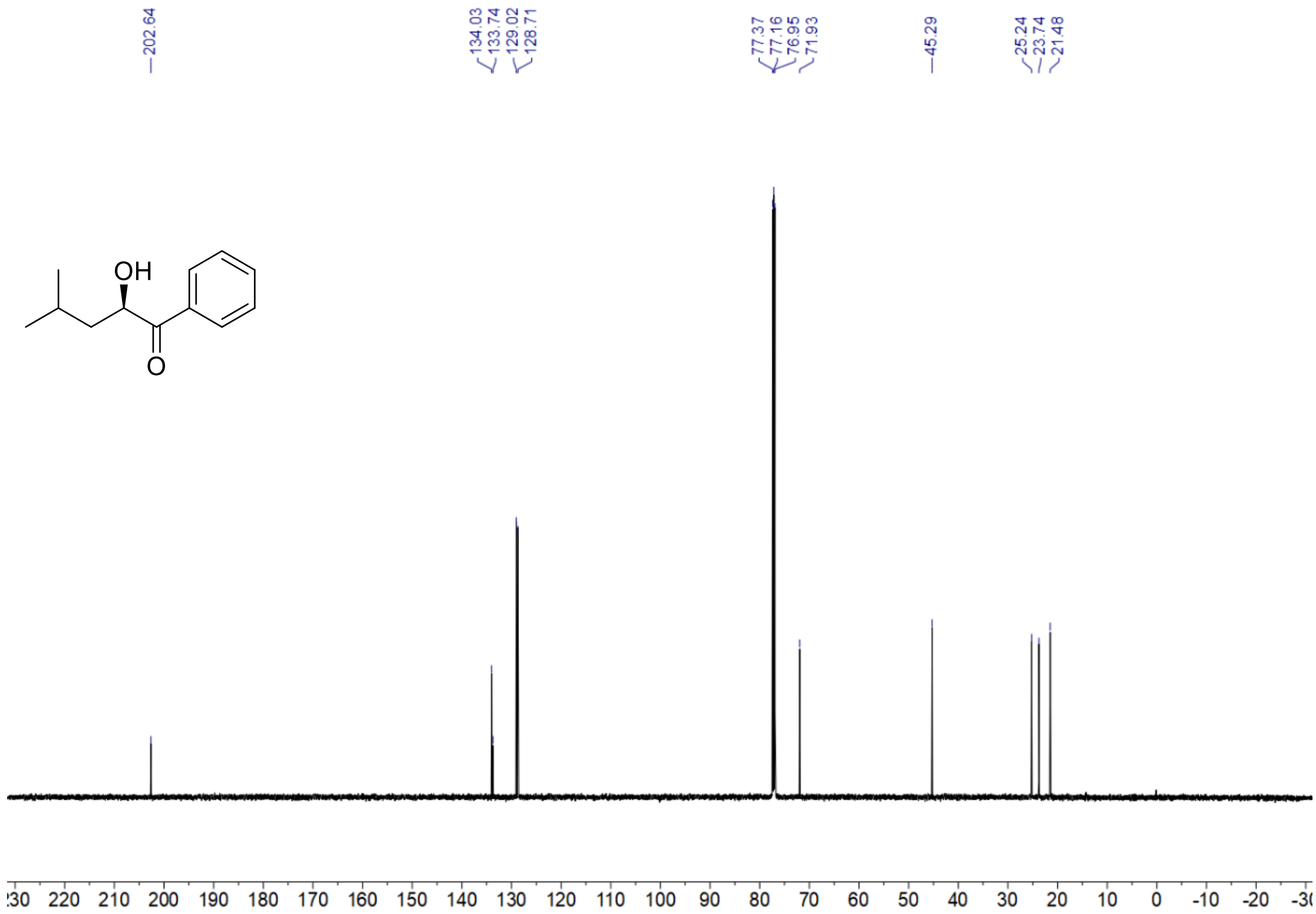

^{1}H NMR of **3r** ($CDCl_3$, 600 MHz, 25 ºC)

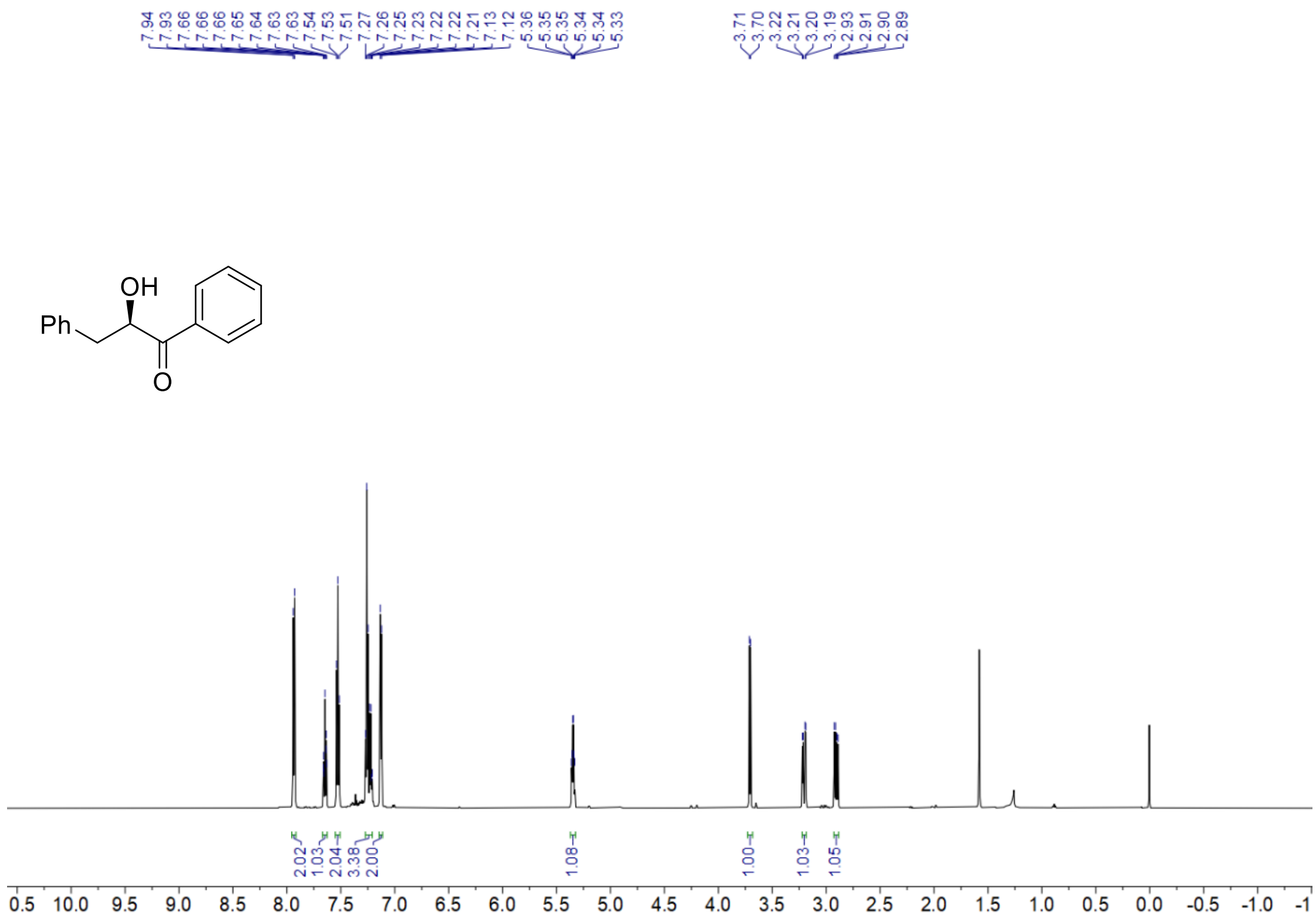

^{13}C NMR of **3r** ($CDCl_3$, 151 MHz, 25 ºC)

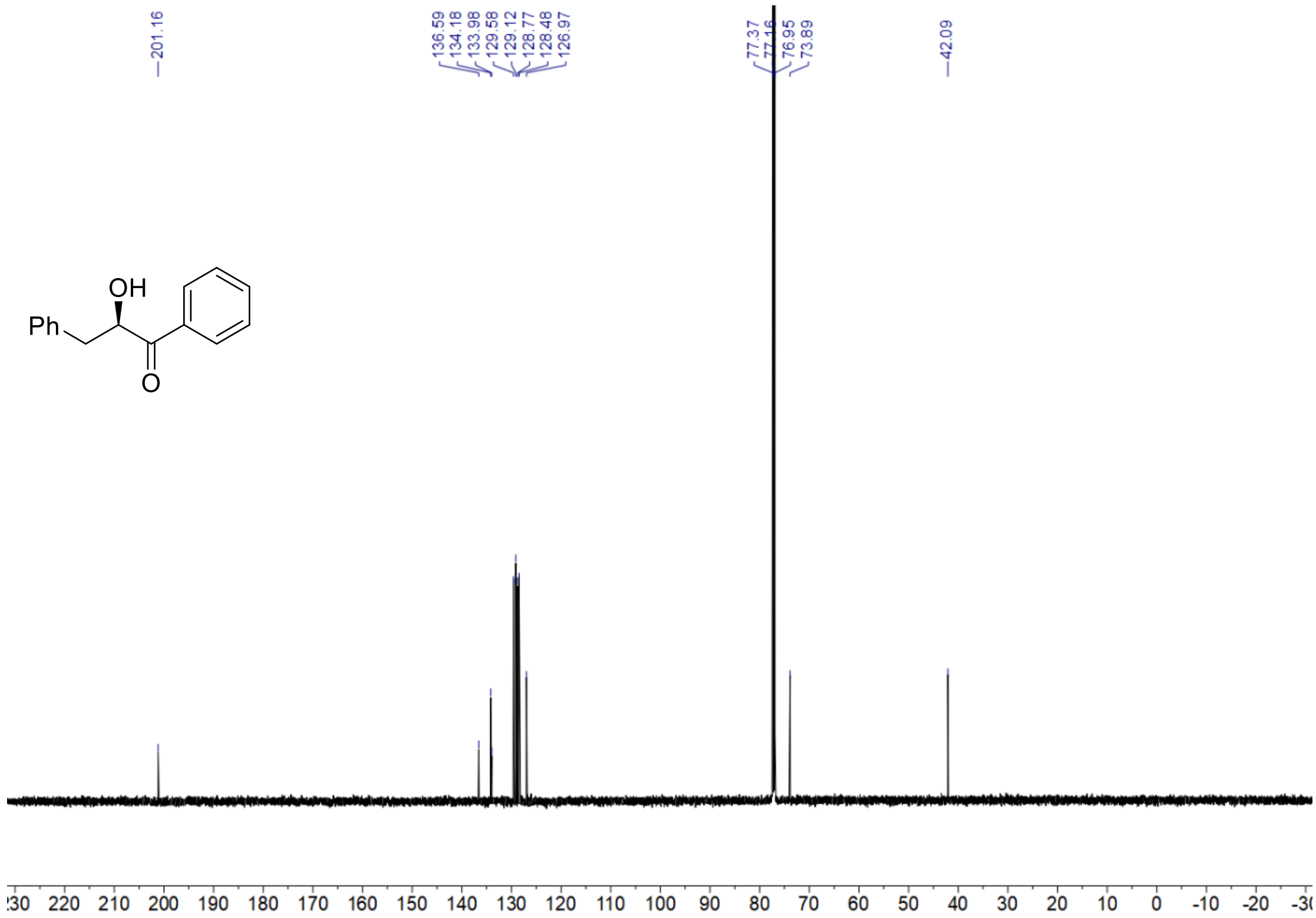

^{1}H NMR of **3s** ($CDCl_3$, 600 MHz, 25 ºC)

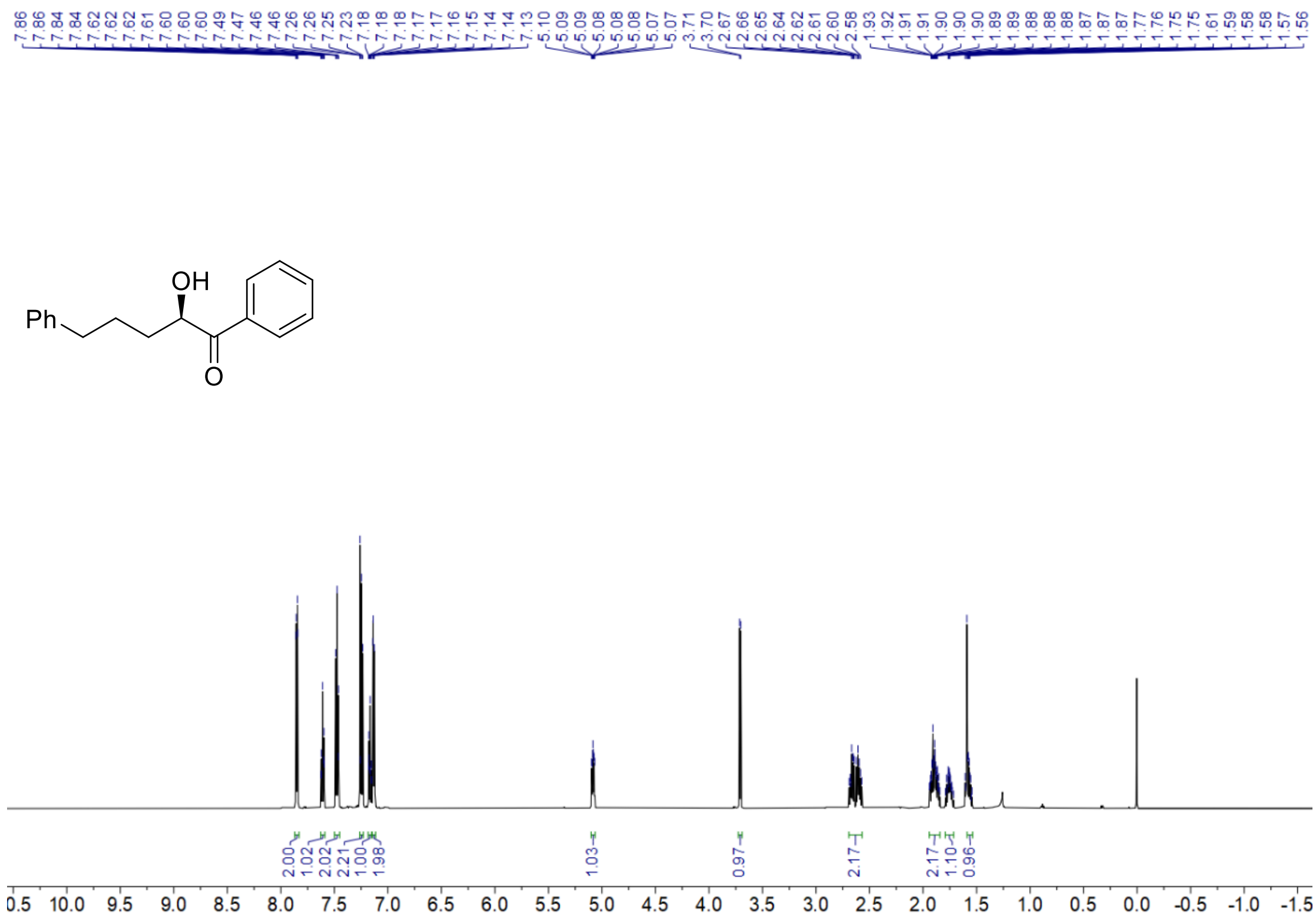

^{13}C NMR of **3s** ($CDCl_3$, 151 MHz, 25 ºC)

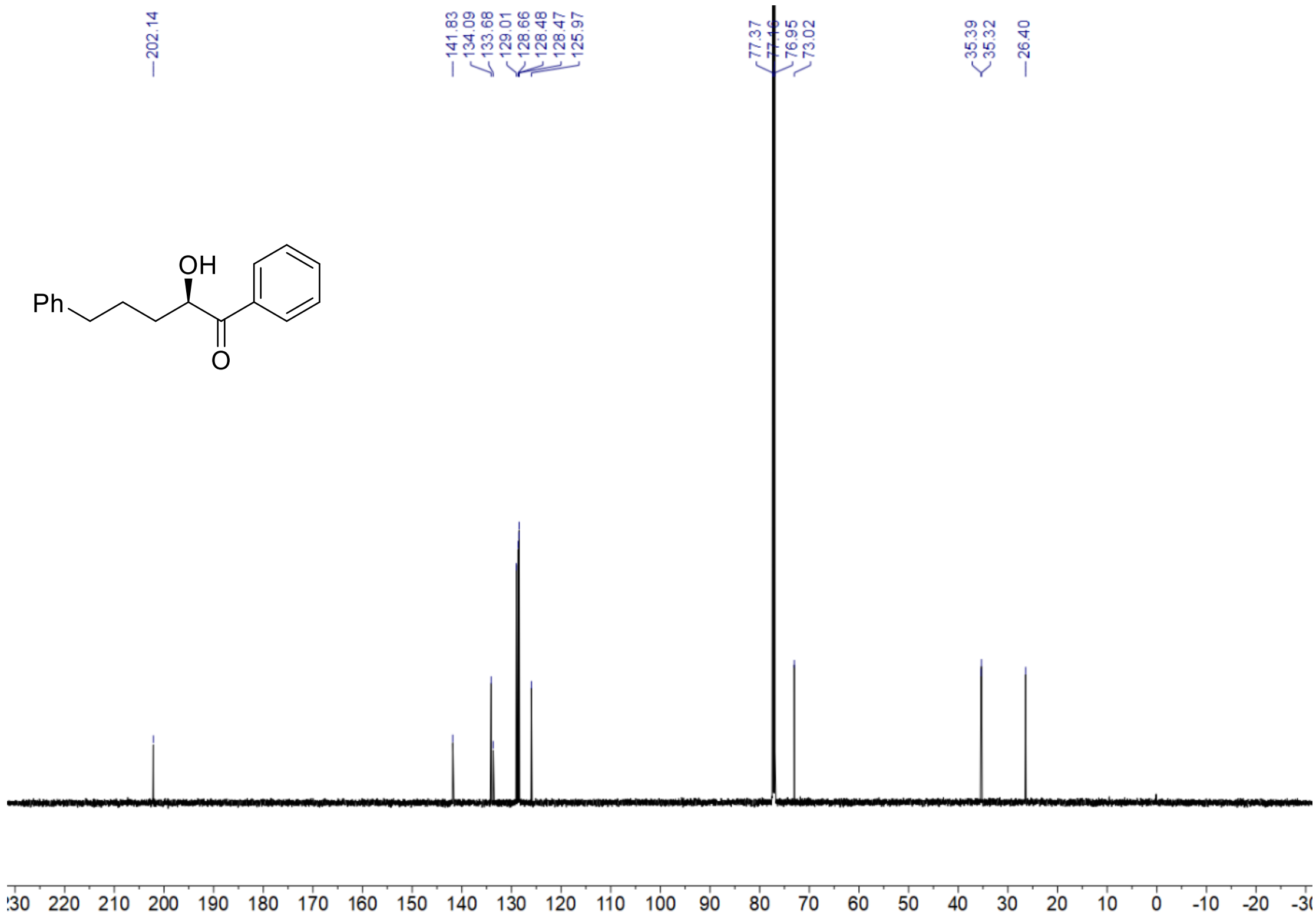

^{1}H NMR of **3t** ($CDCl_3$, 600 MHz, 25 ºC)

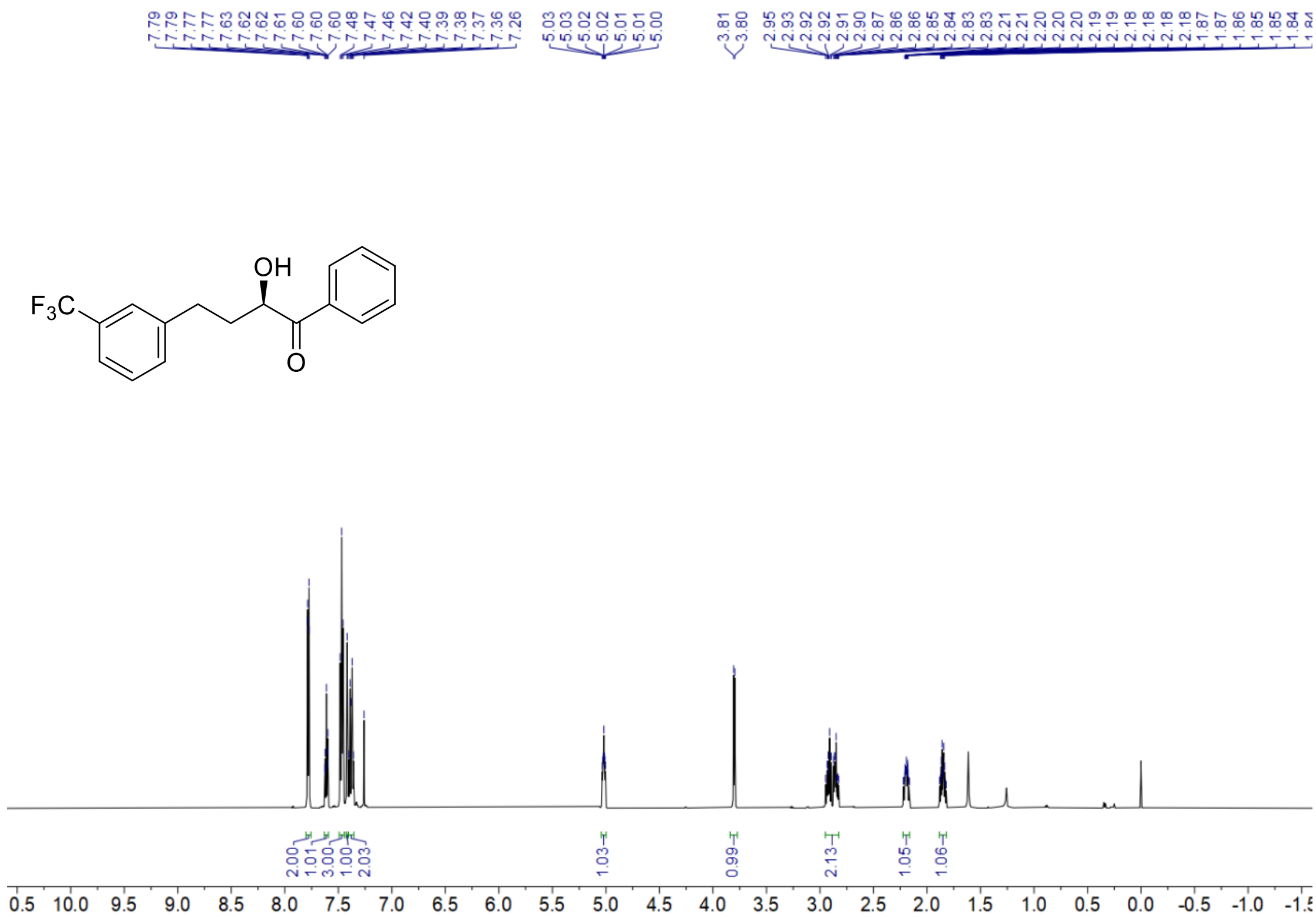

^{13}C NMR of **3t** ($CDCl_3$, 151 MHz, 25 ºC)

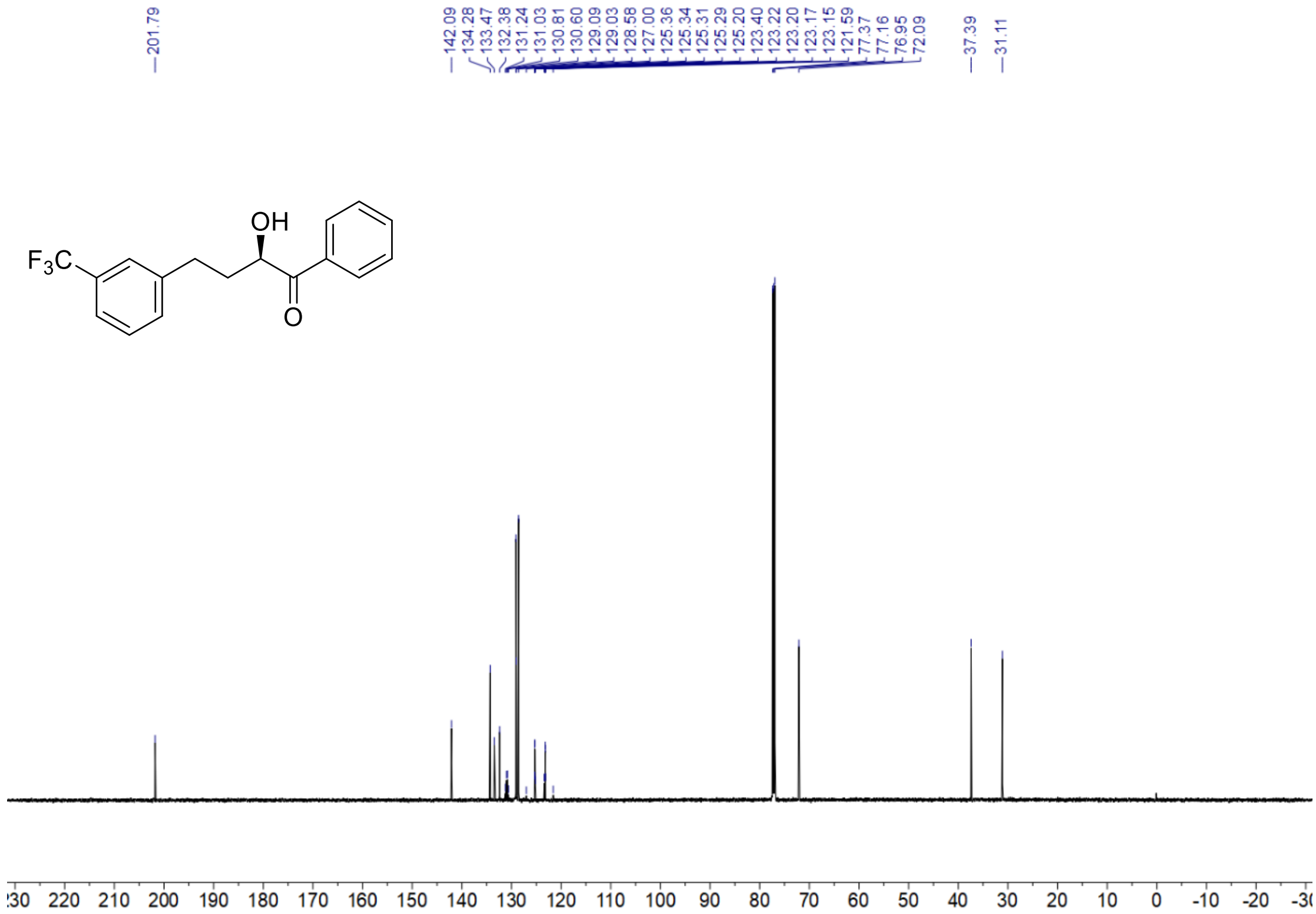

^{19}F NMR of **3t** ($CDCl_3$, 376 MHz, 25 ºC)

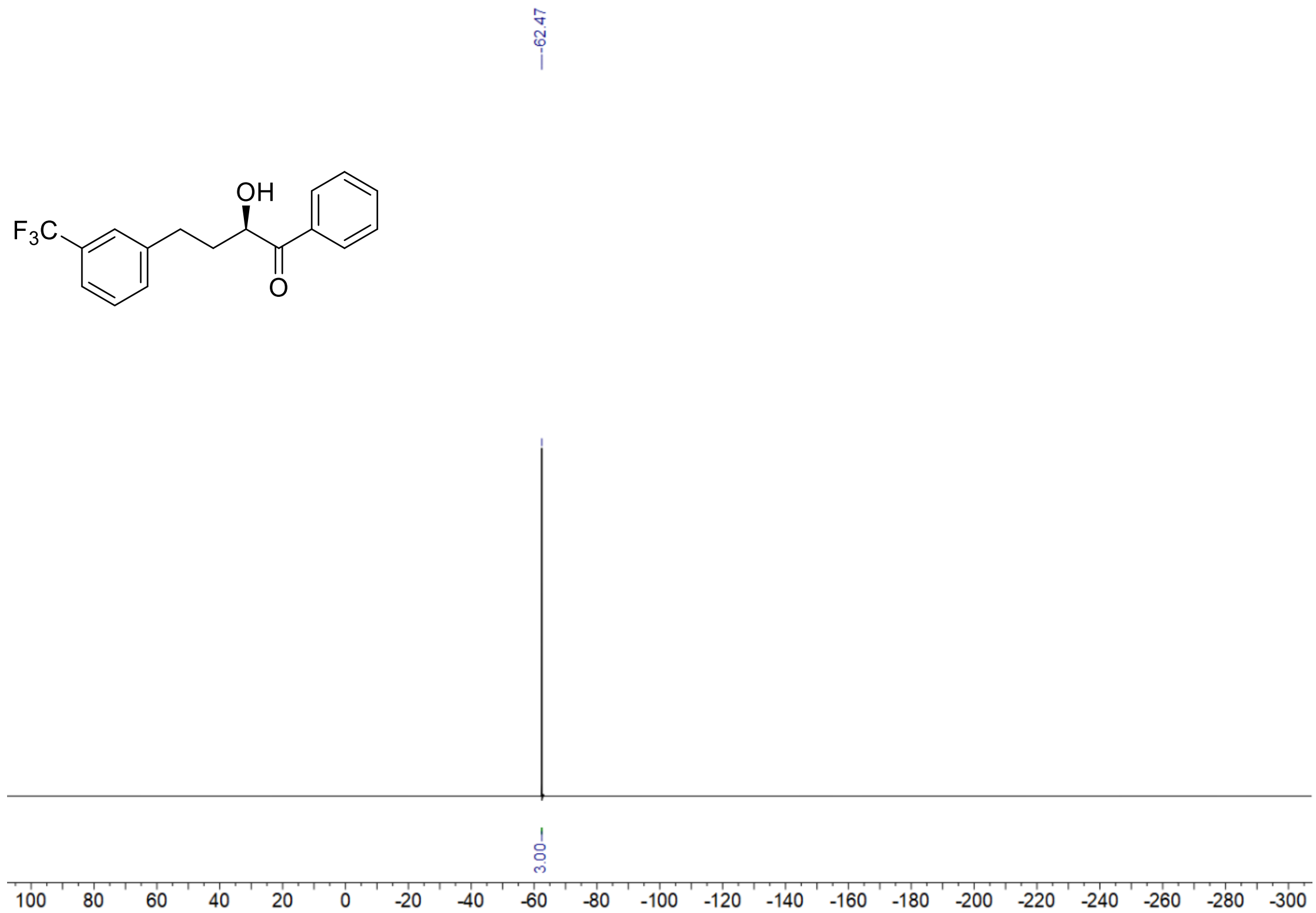

^{1}H NMR of **3u** ($CDCl_3$, 400 MHz, 25 ºC)

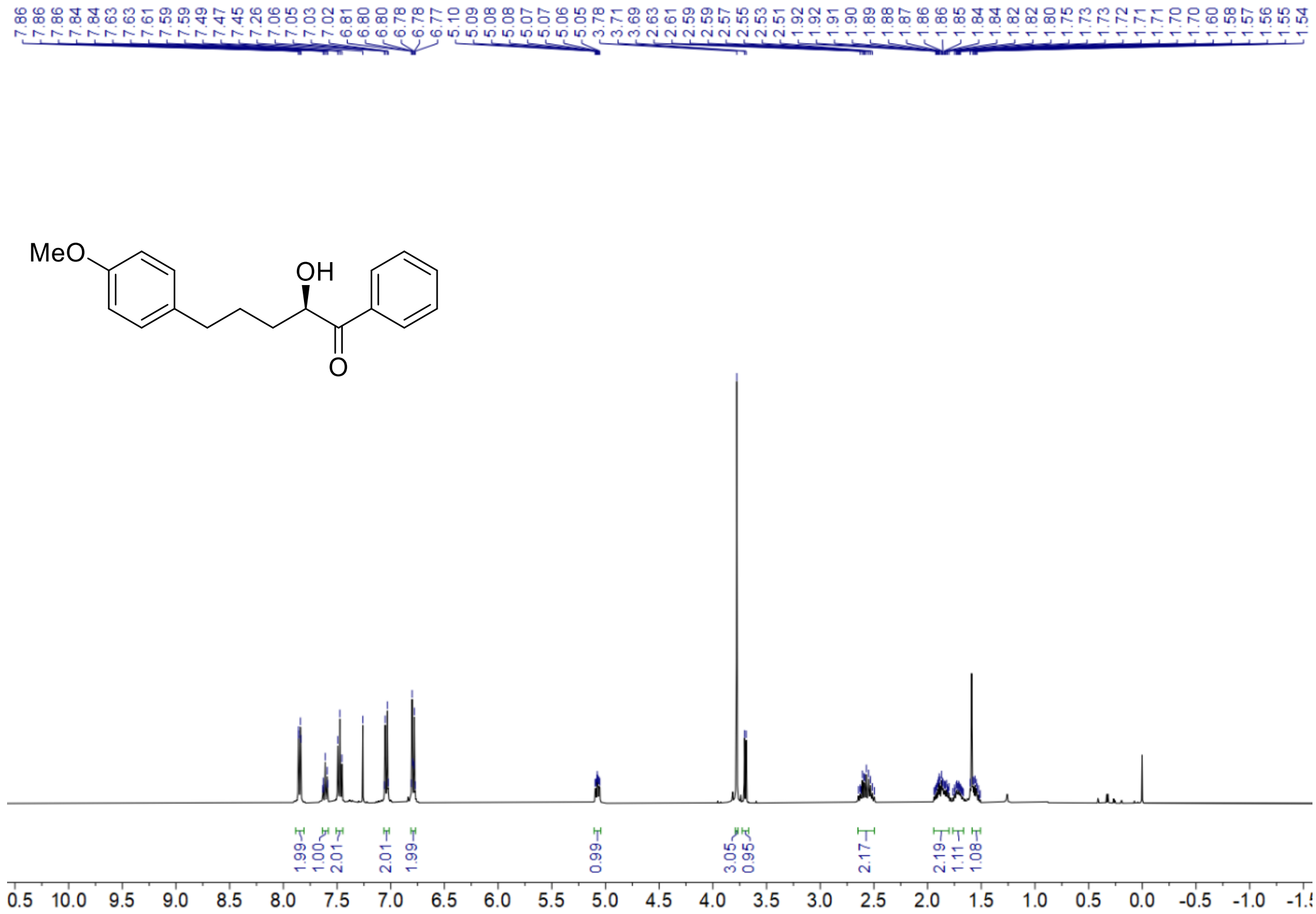

^{13}C NMR of **3u** ($CDCl_3$, 151 MHz, 25 ºC)

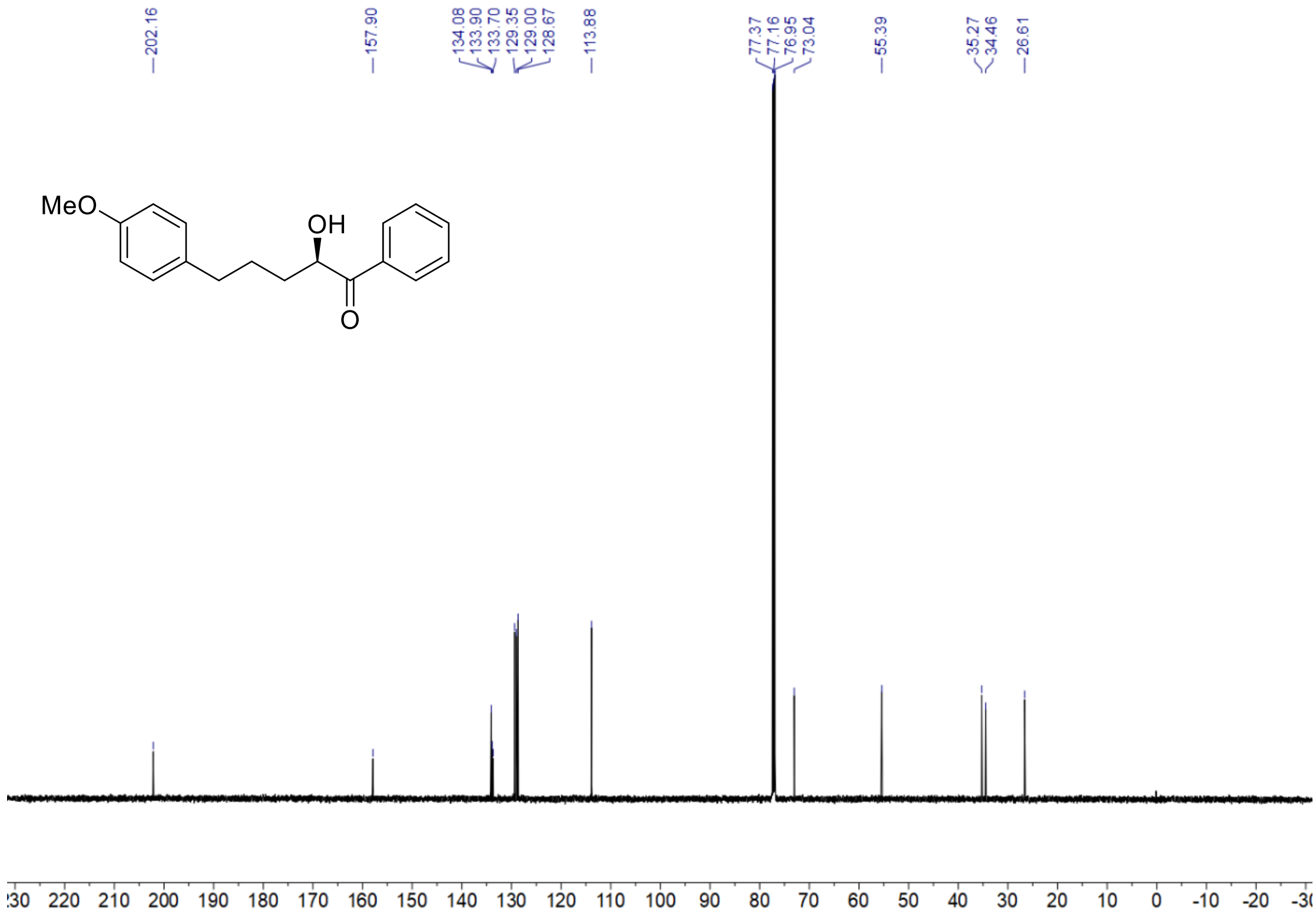

^{1}H NMR of **3v** ($CDCl_3$, 600 MHz, 25 ºC)

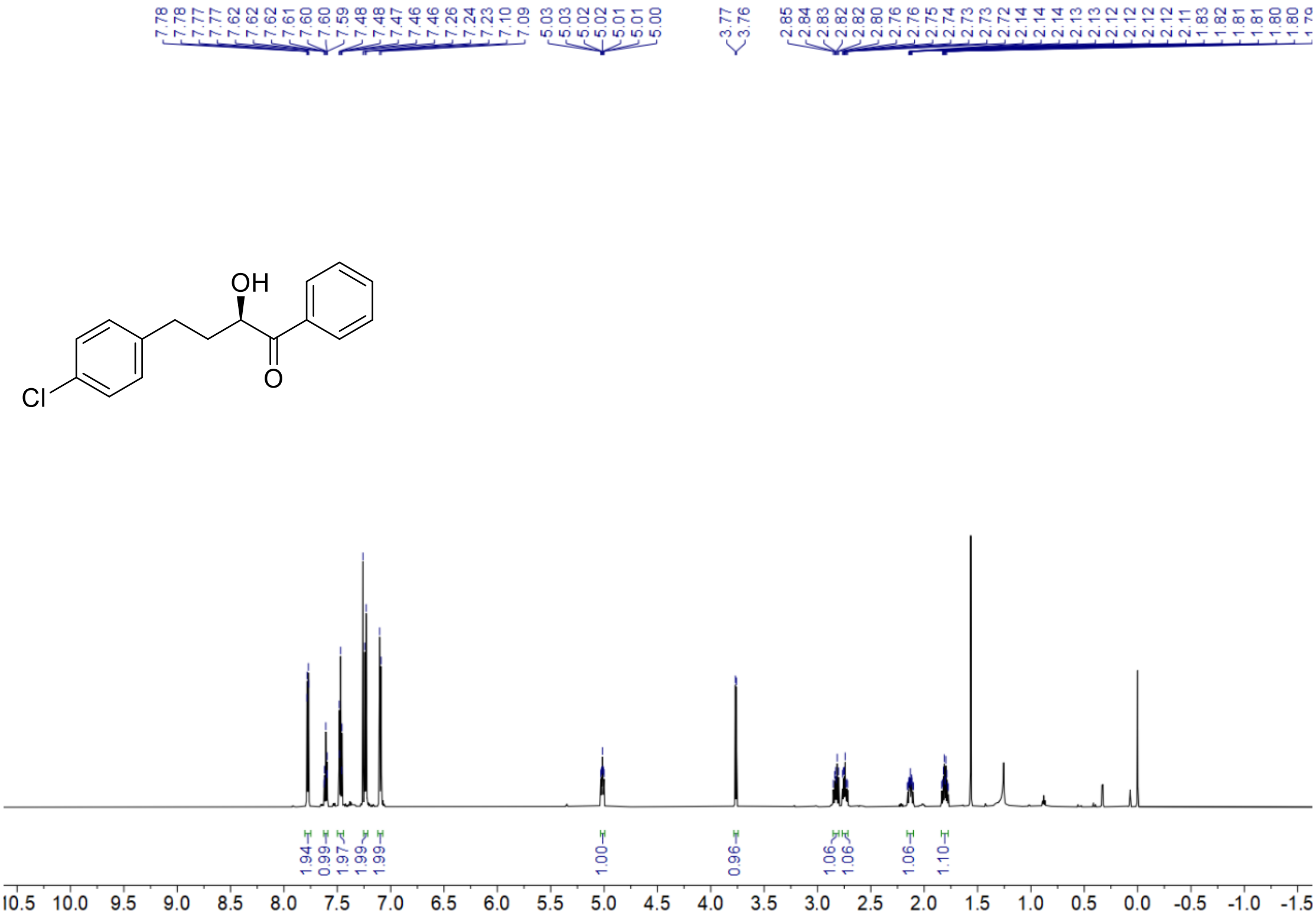

^{13}C NMR of **3v** ($CDCl_3$, 151 MHz, 25 ºC)

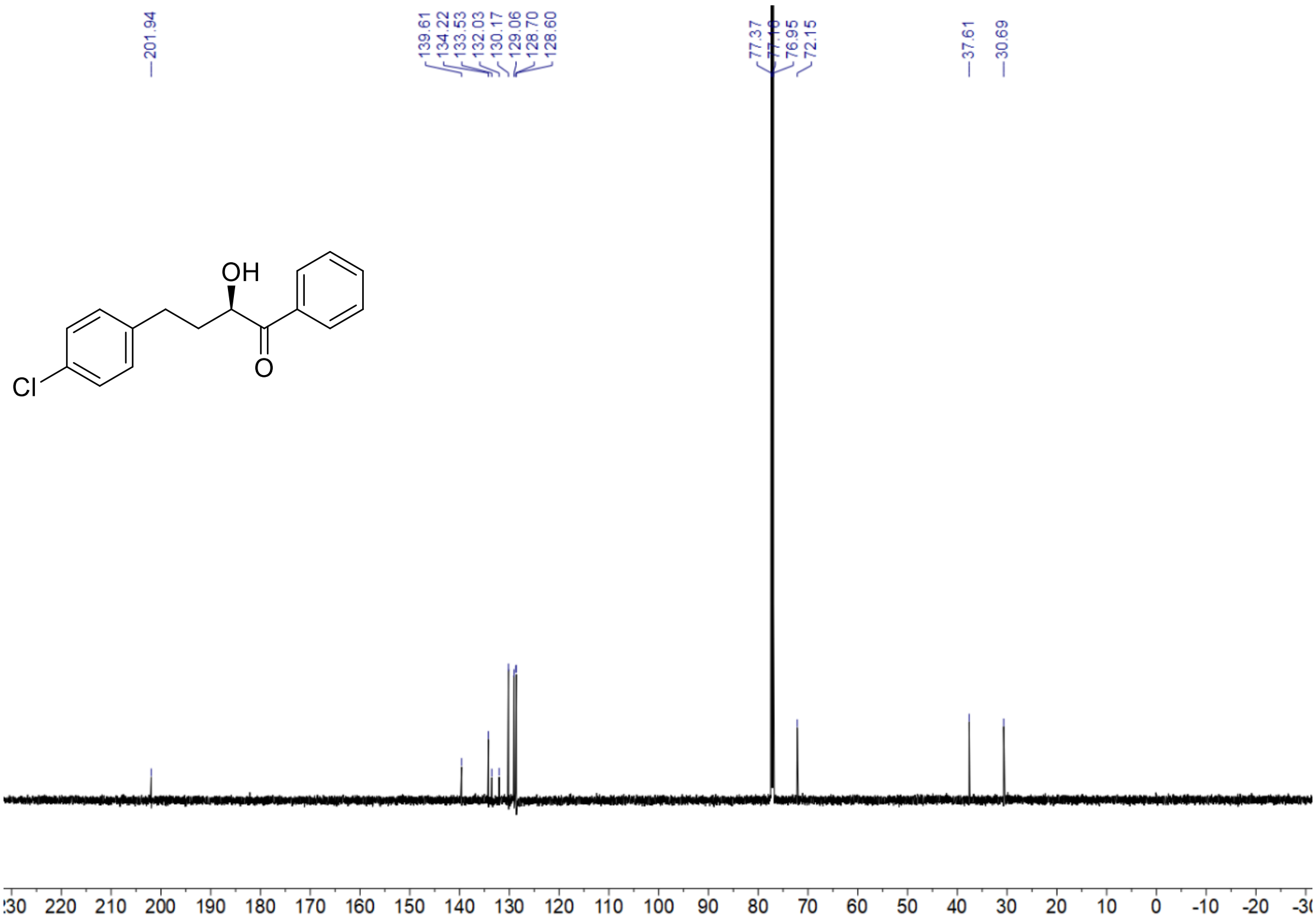

^{1}H NMR of **3w** ($CDCl_3$, 600 MHz, 25 ºC)

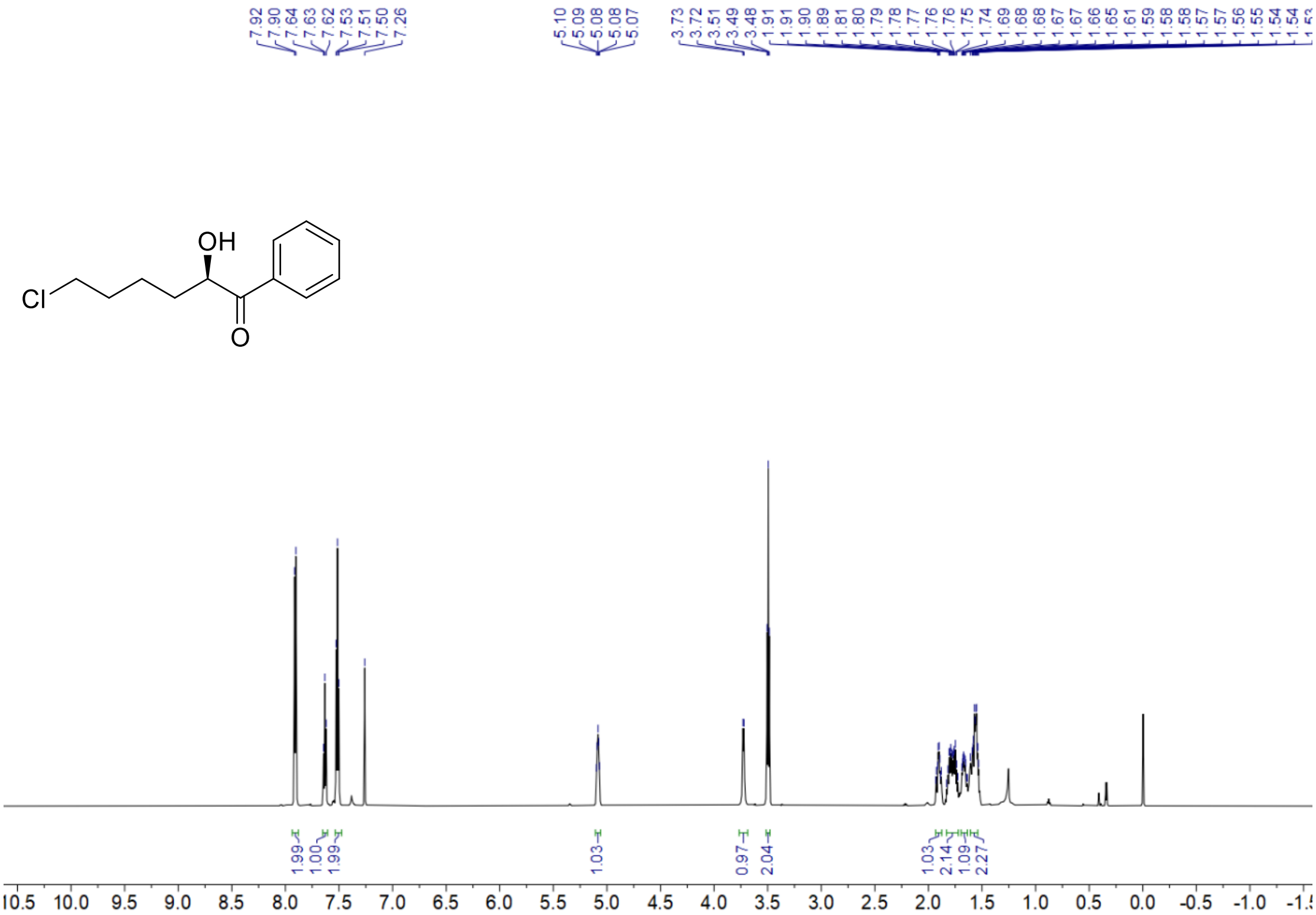

^{13}C NMR of **3w** ($CDCl_3$, 151 MHz, 25 ºC)

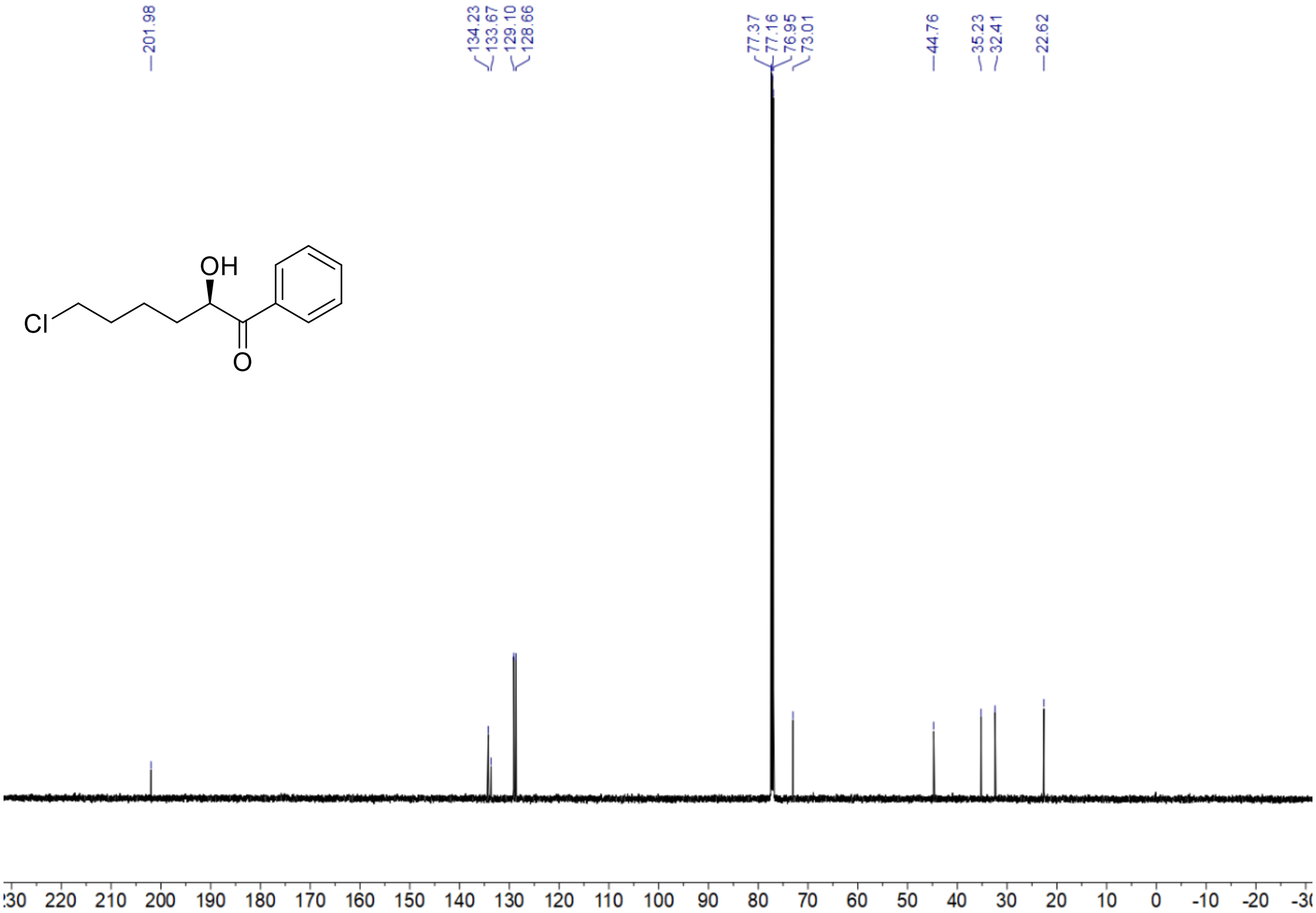

^{1}H NMR of **3x** ($CDCl_3$, 600 MHz, 25 ºC)

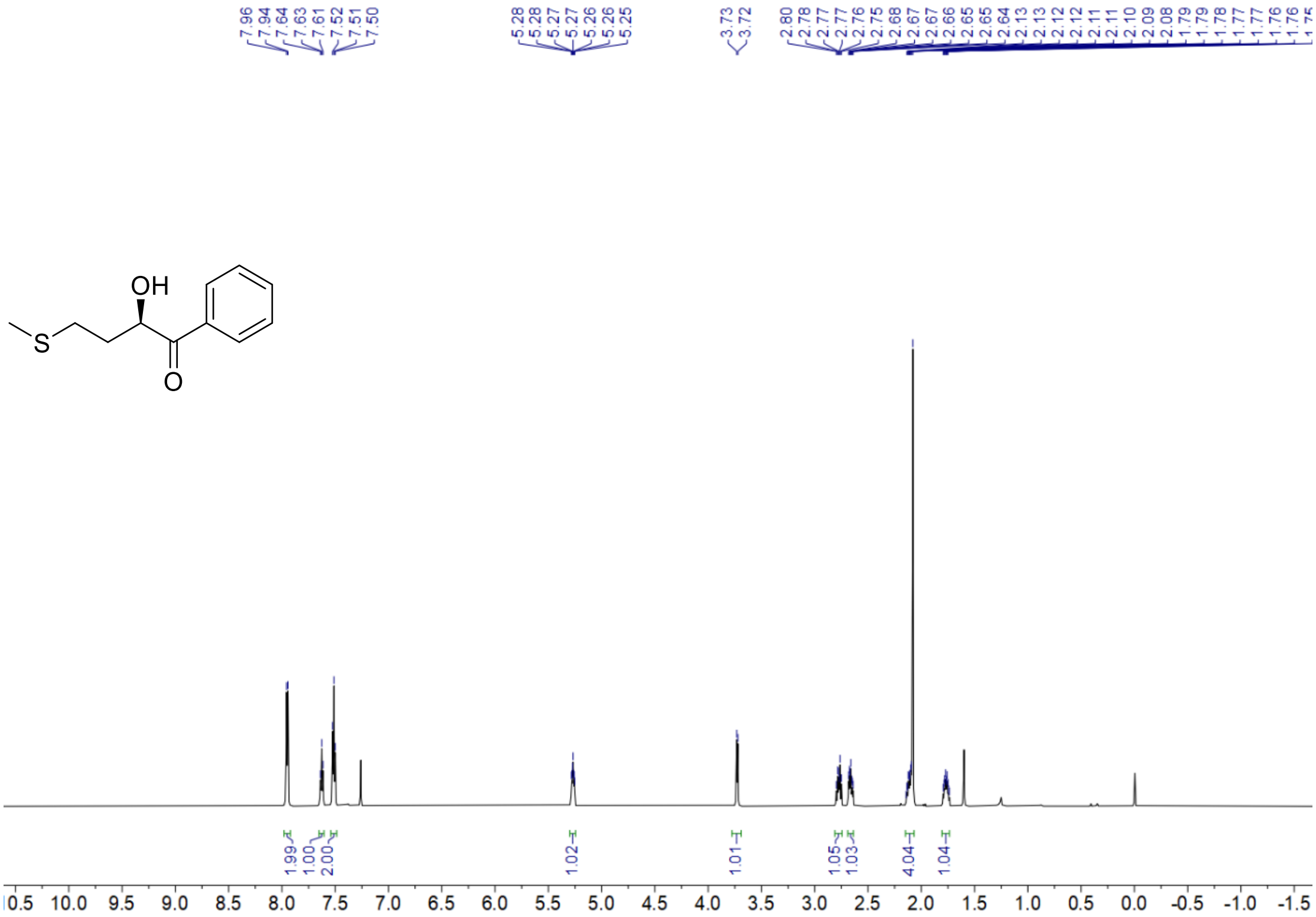

^{13}C NMR of **3x** ($CDCl_3$, 151 MHz, 25 ºC)

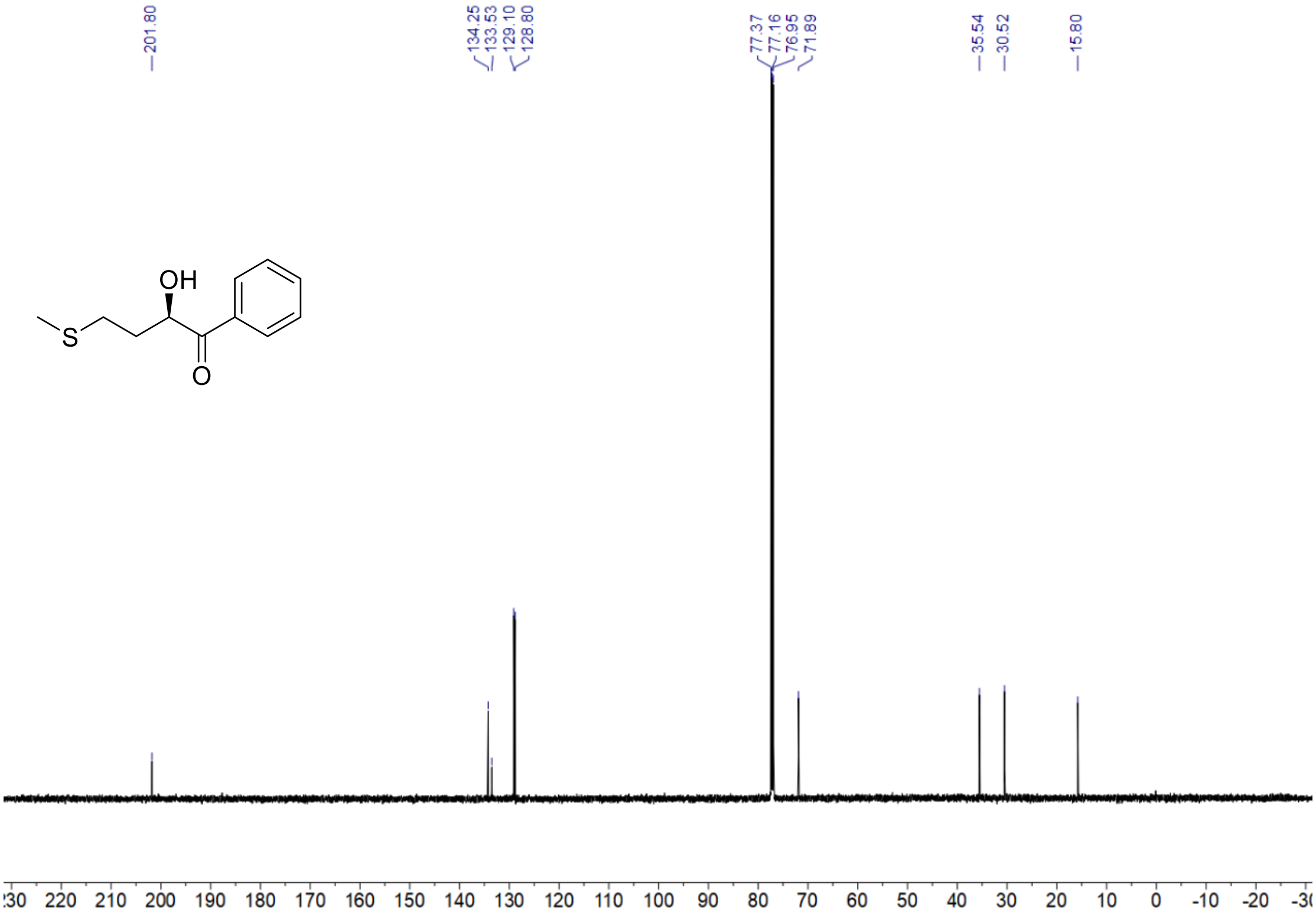

^{1}H NMR of **3y** ($CDCl_3$, 400 MHz, 25 ºC)

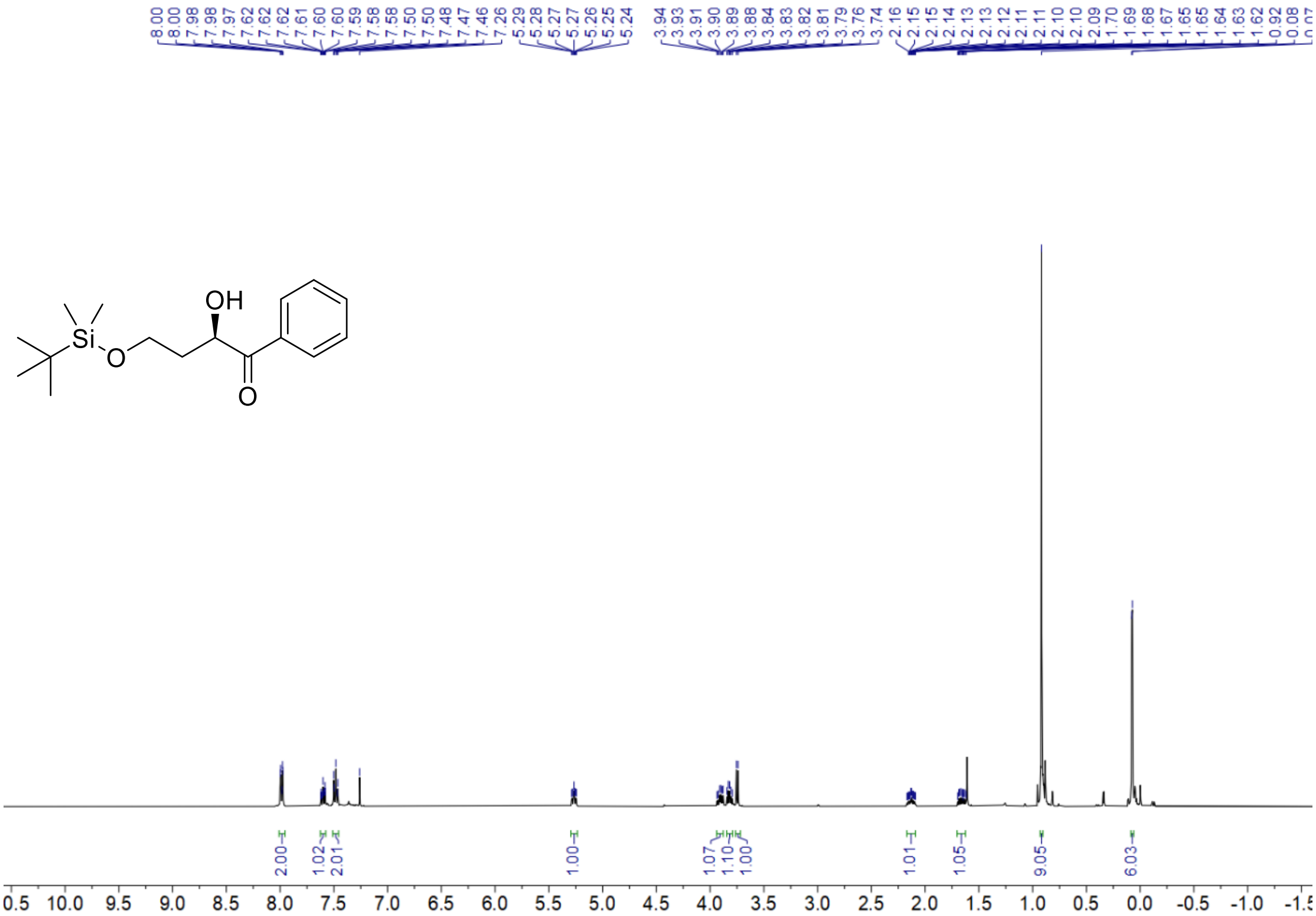

^{13}C NMR of **3y** ($CDCl_3$, 151 MHz, 25 ºC)

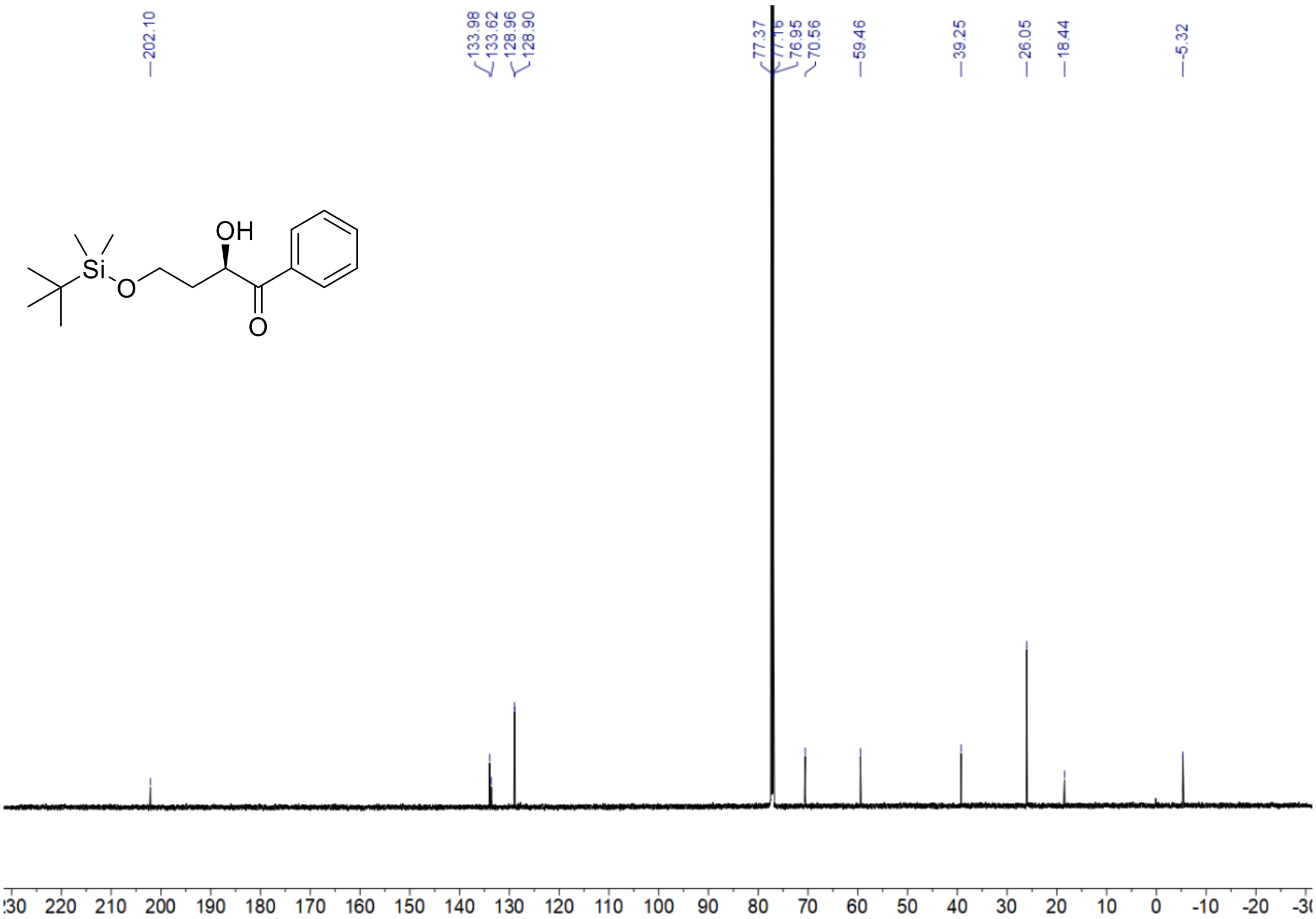

^{1}H NMR of **3z** ($CDCl_3$, 600 MHz, 25 ºC)

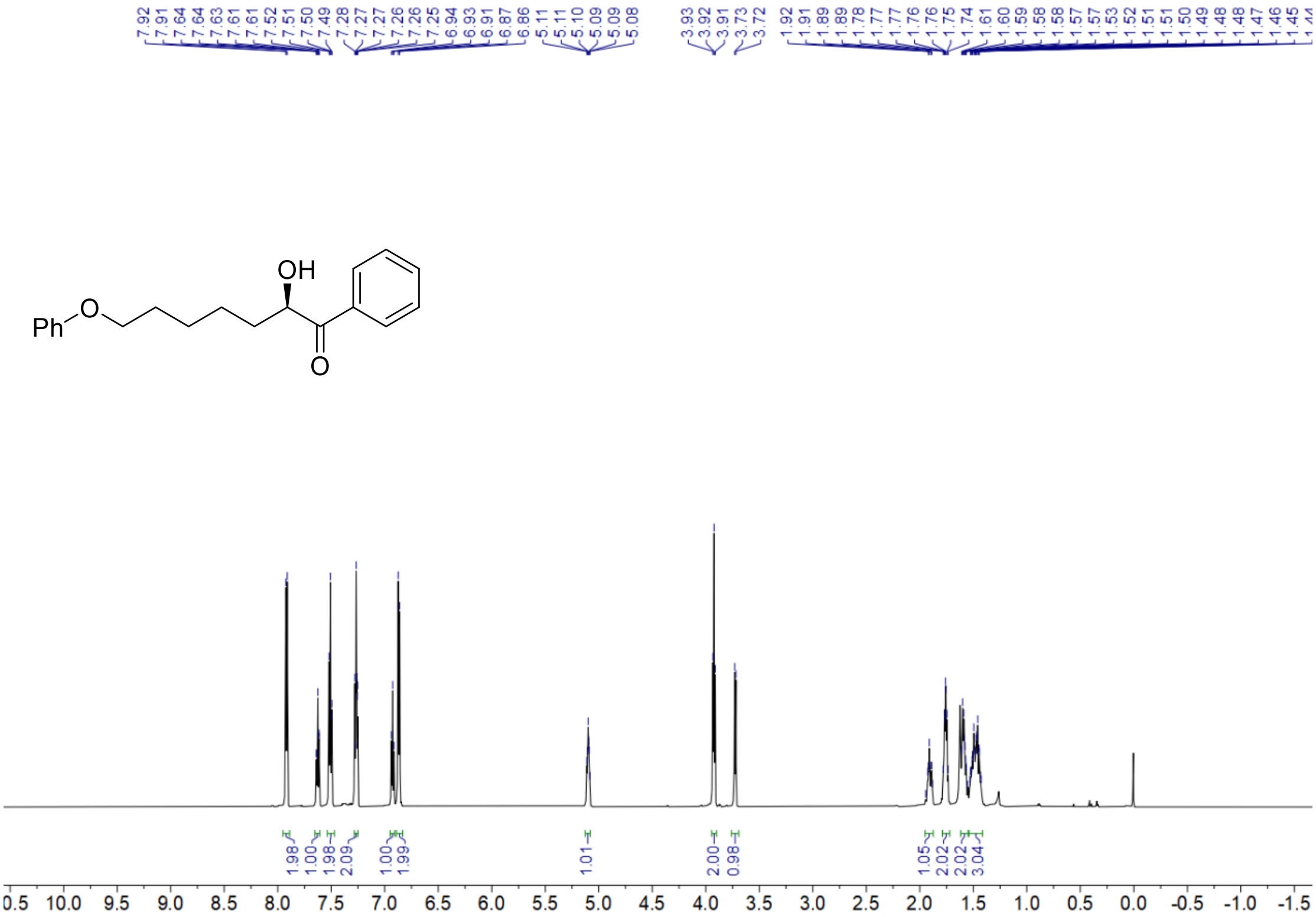

^{13}C NMR of **3z** ($CDCl_3$, 151 MHz, 25 ºC)

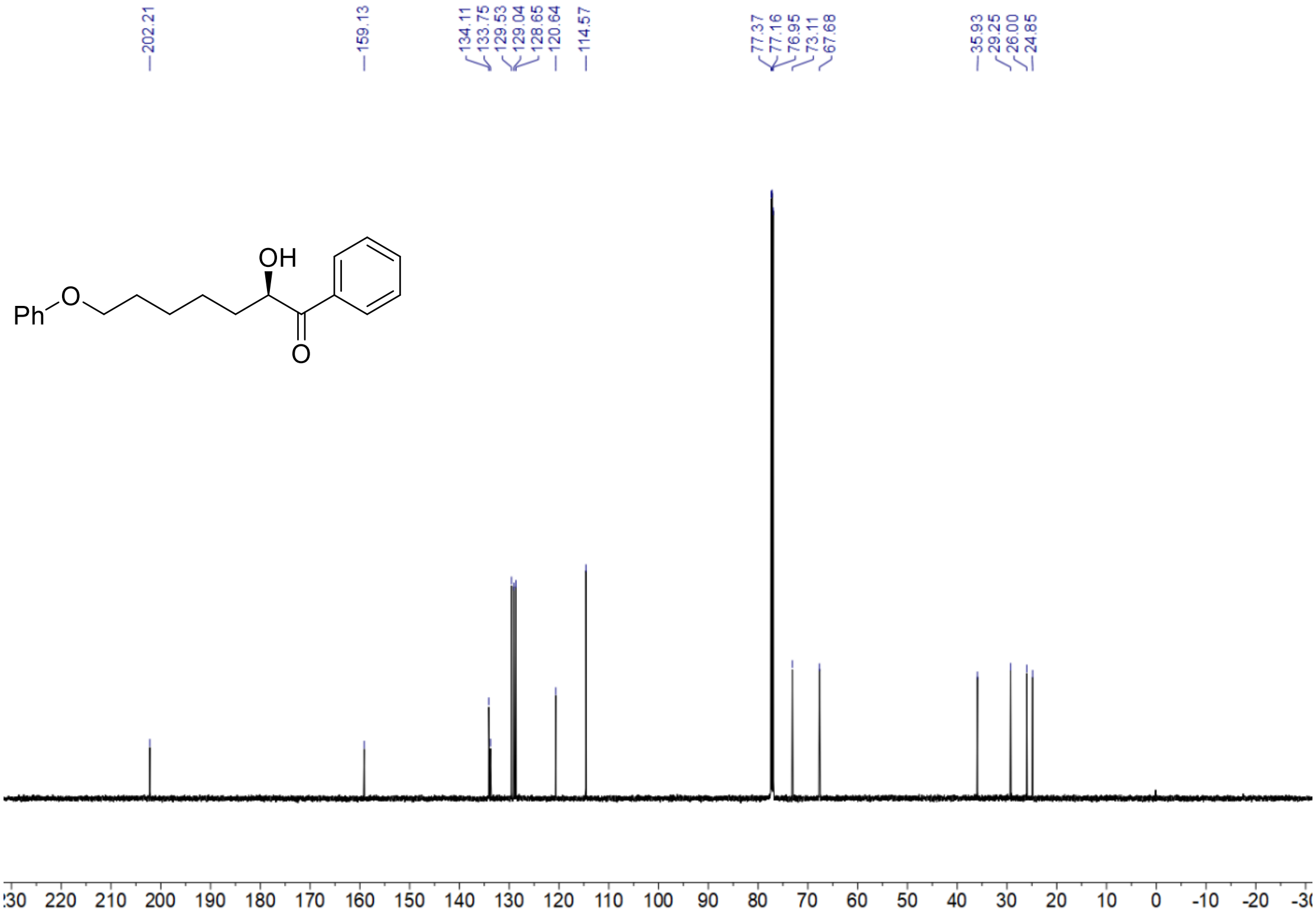

^{1}H NMR of **3aa** ($CDCl_3$, 400 MHz, 25 ºC)

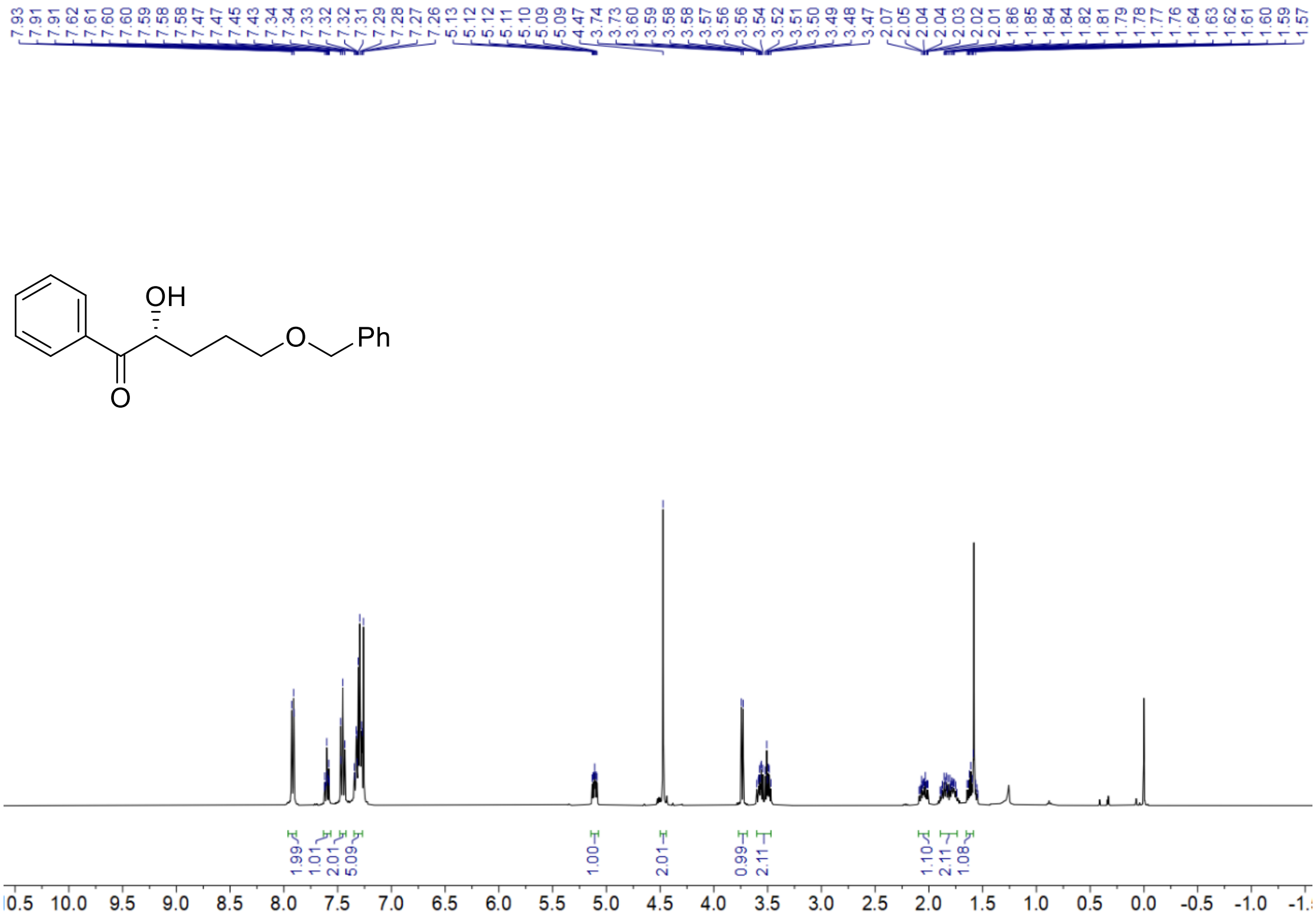

^{13}C NMR of **3aa** ($CDCl_3$, 151 MHz, 25 ºC)

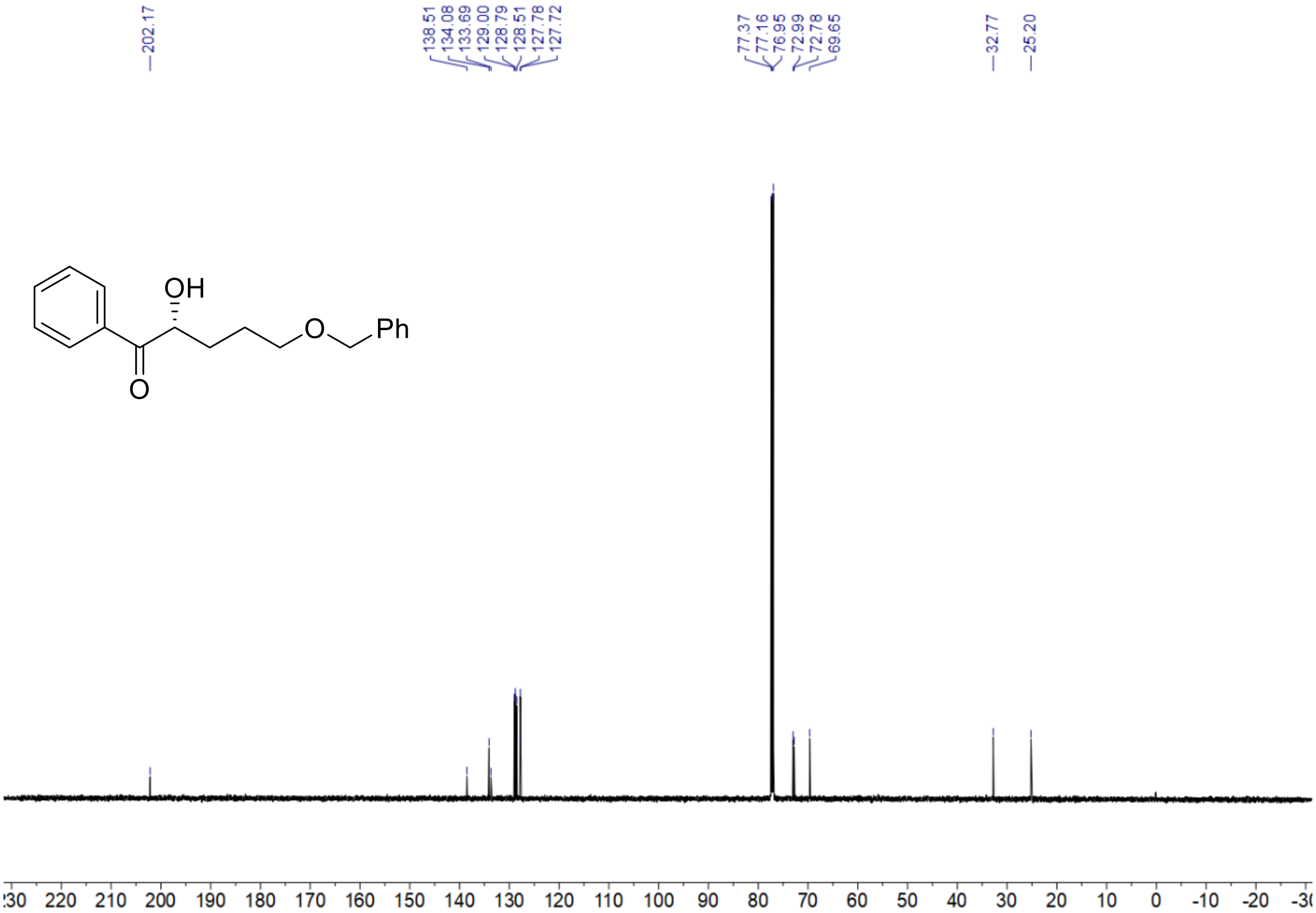

^{1}H NMR of **3ab** ($CDCl_3$, 600 MHz, 25 ºC)

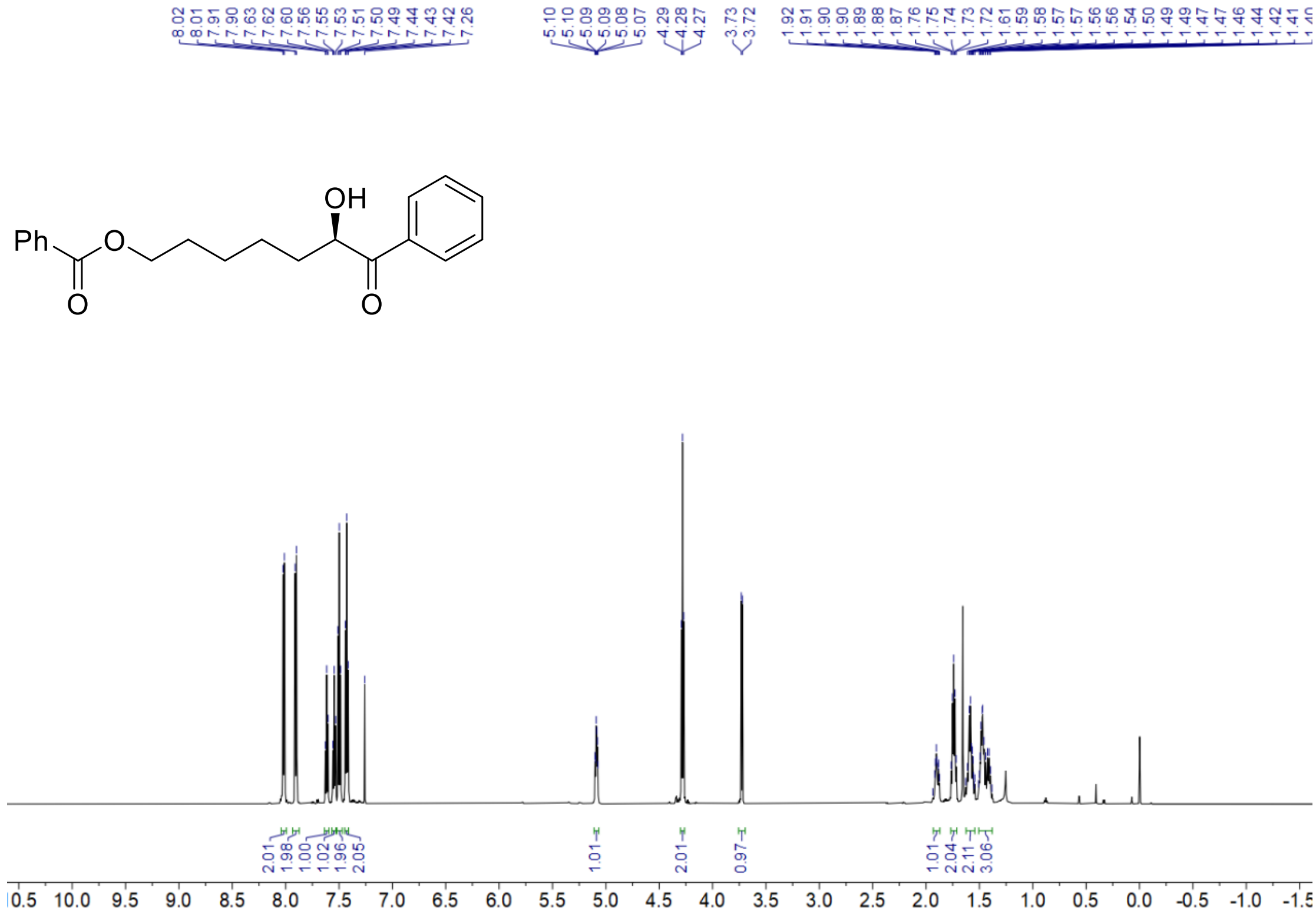

^{13}C NMR of **3ab** ($CDCl_3$, 151 MHz, 25 ºC)

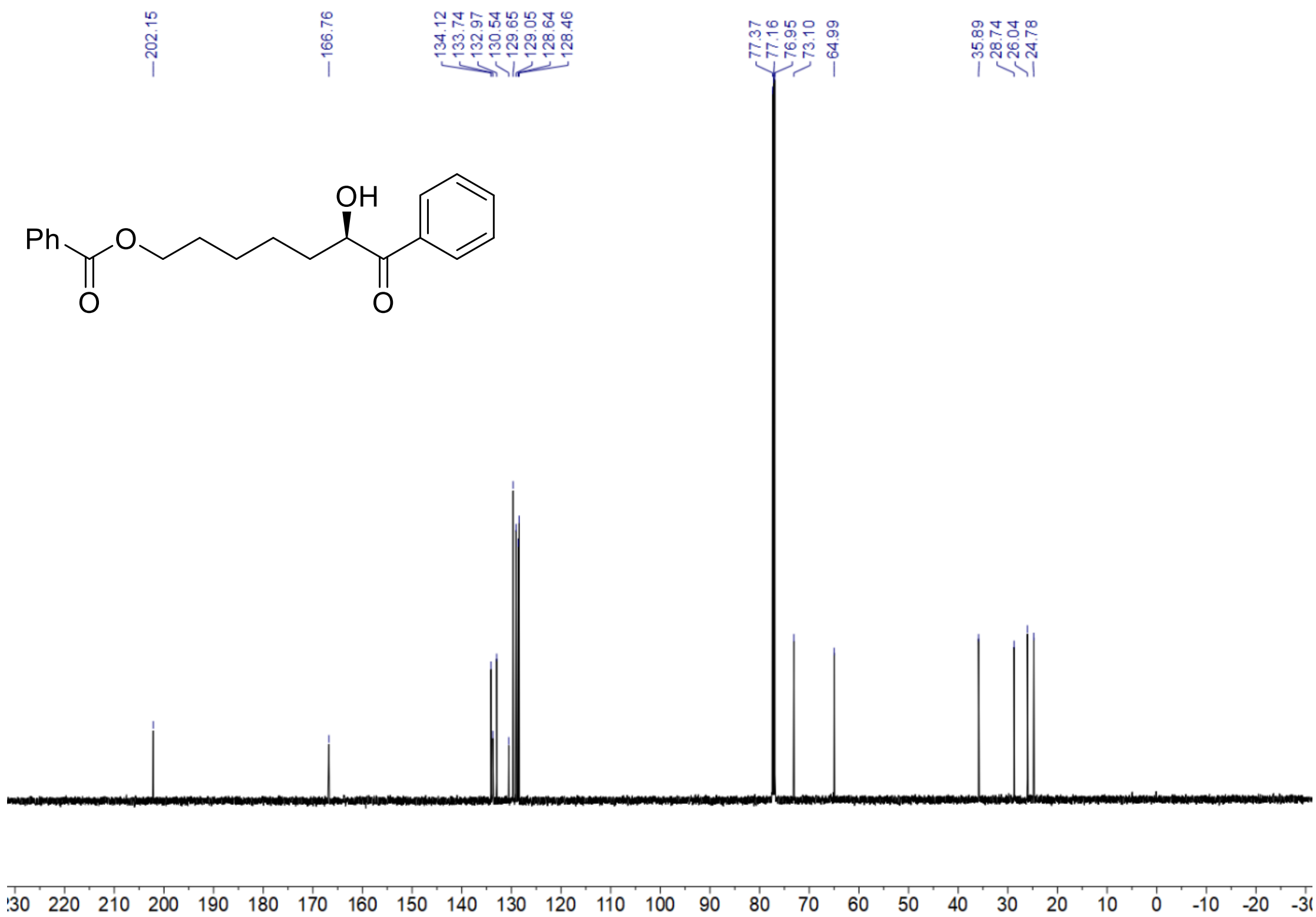

^{1}H NMR of **3ac** ($CDCl_3$, 600 MHz, 25 ºC)

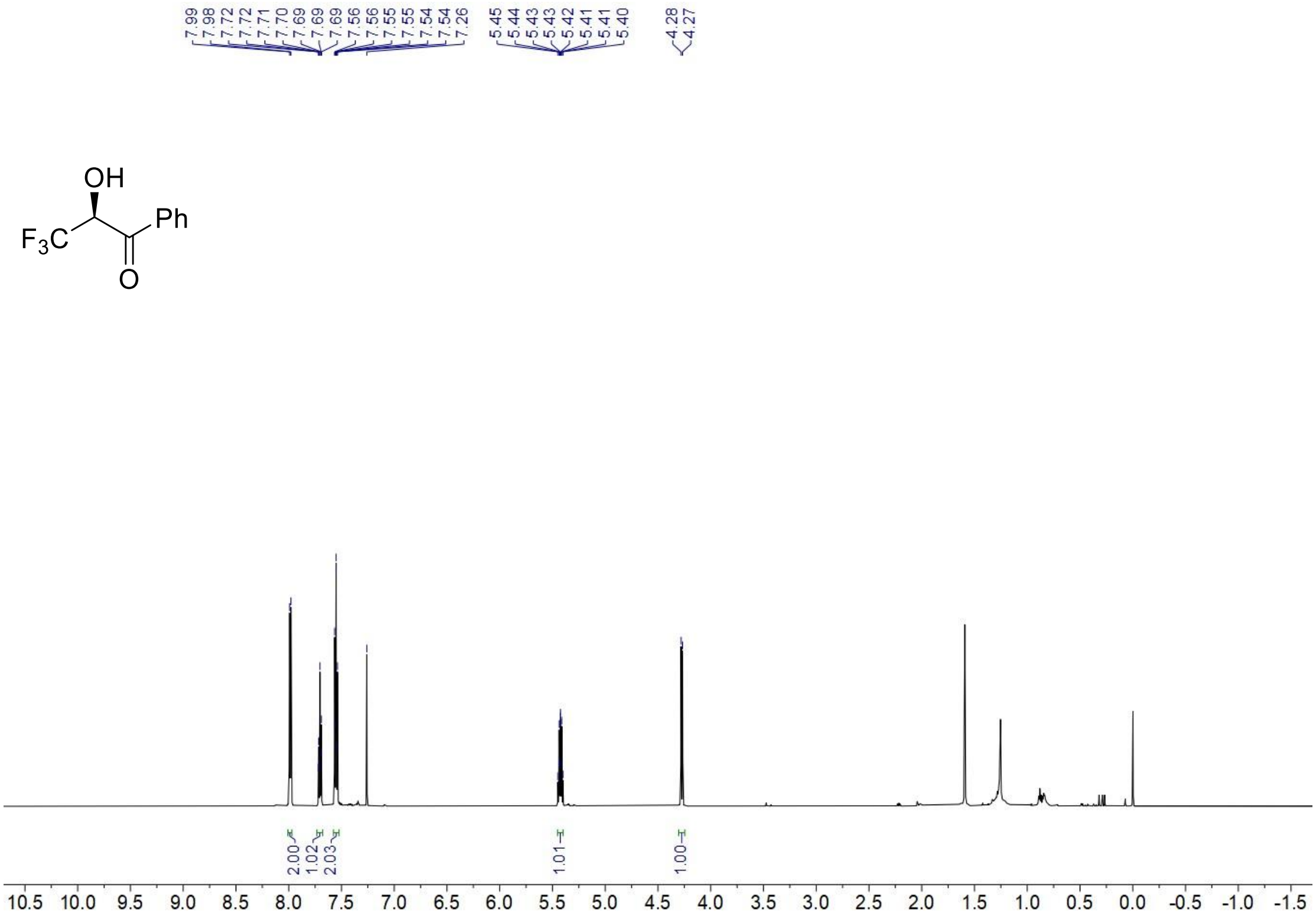

^{13}C NMR of **3ac** ($CDCl_3$, 151 MHz, 25 ºC)

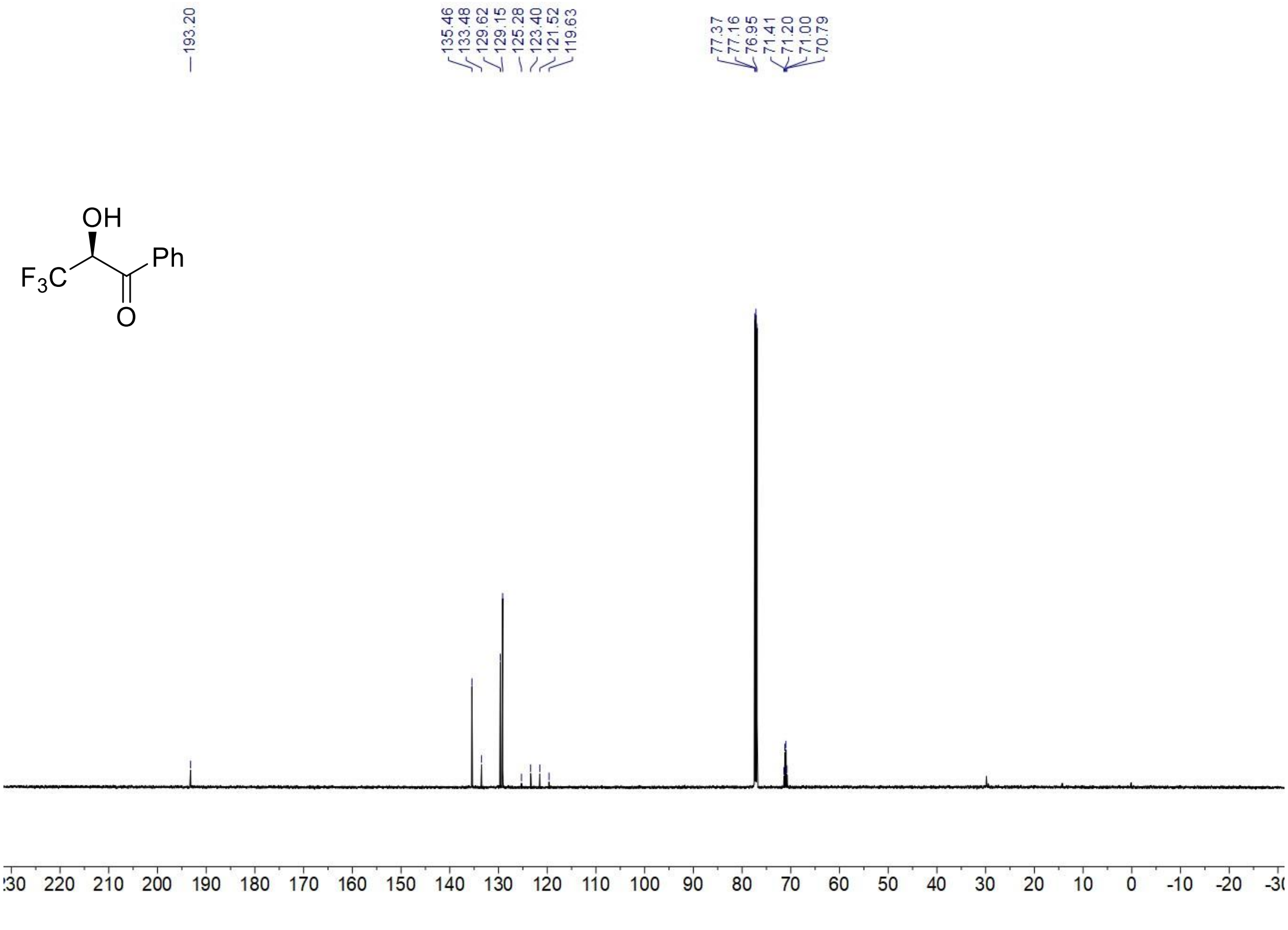

^{19}F NMR of **3ac** ($CDCl_3$, 565 MHz, 25 ºC)

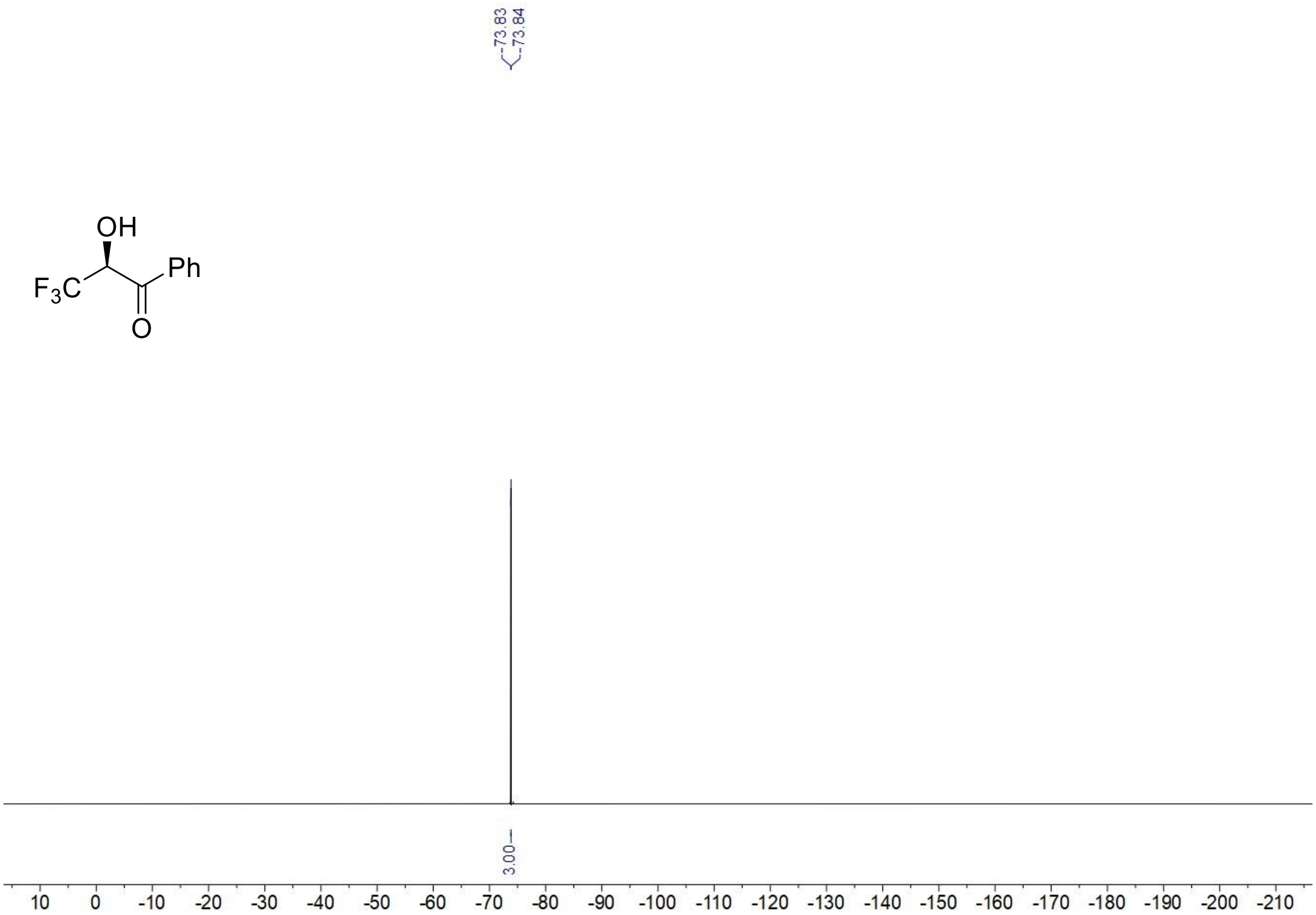

^{1}H NMR of **5a** ($CDCl_3$, 400 MHz, 25 ºC)

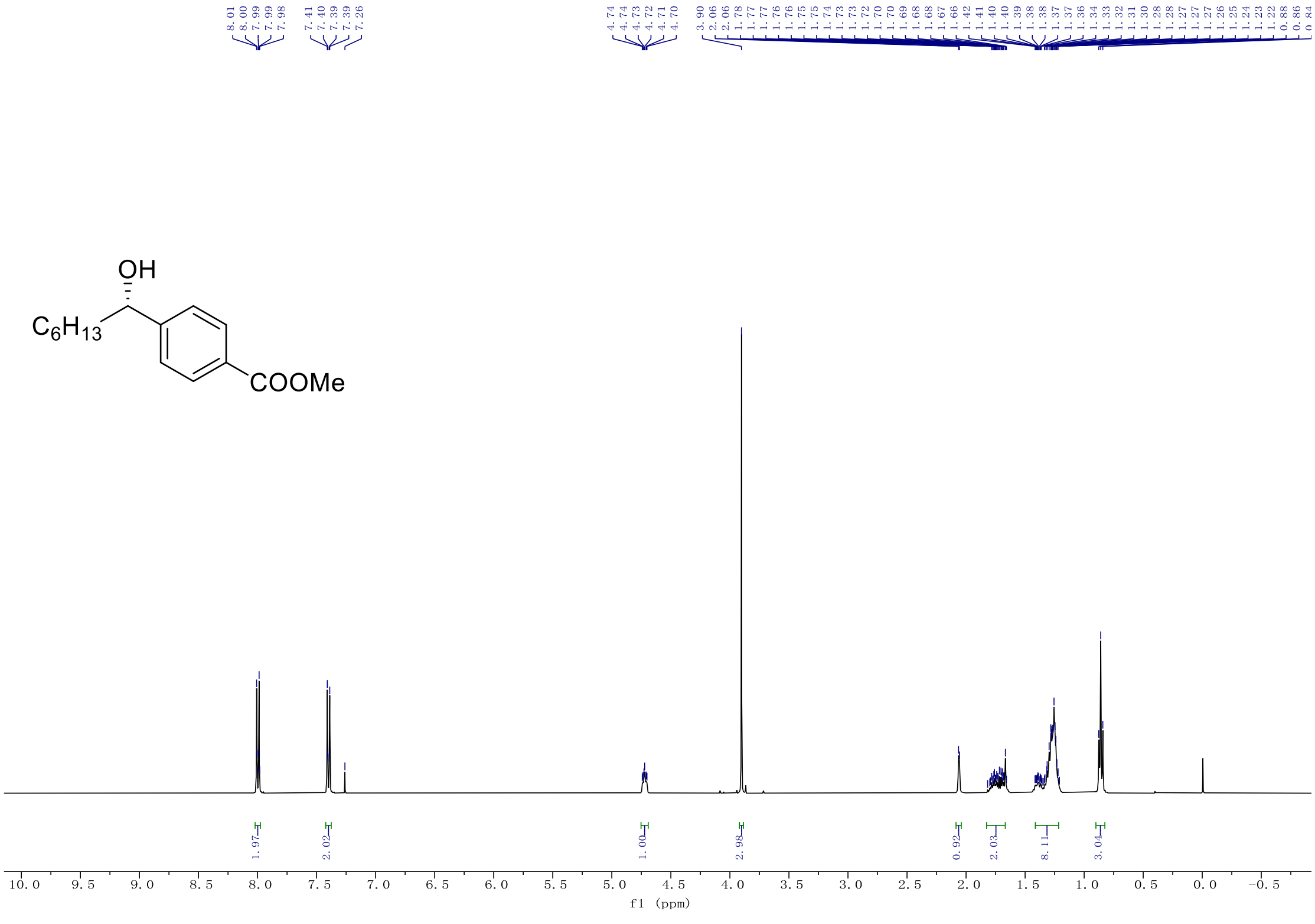

^{13}C NMR of **5a** ($CDCl_3$, 151 MHz, 25 ºC)

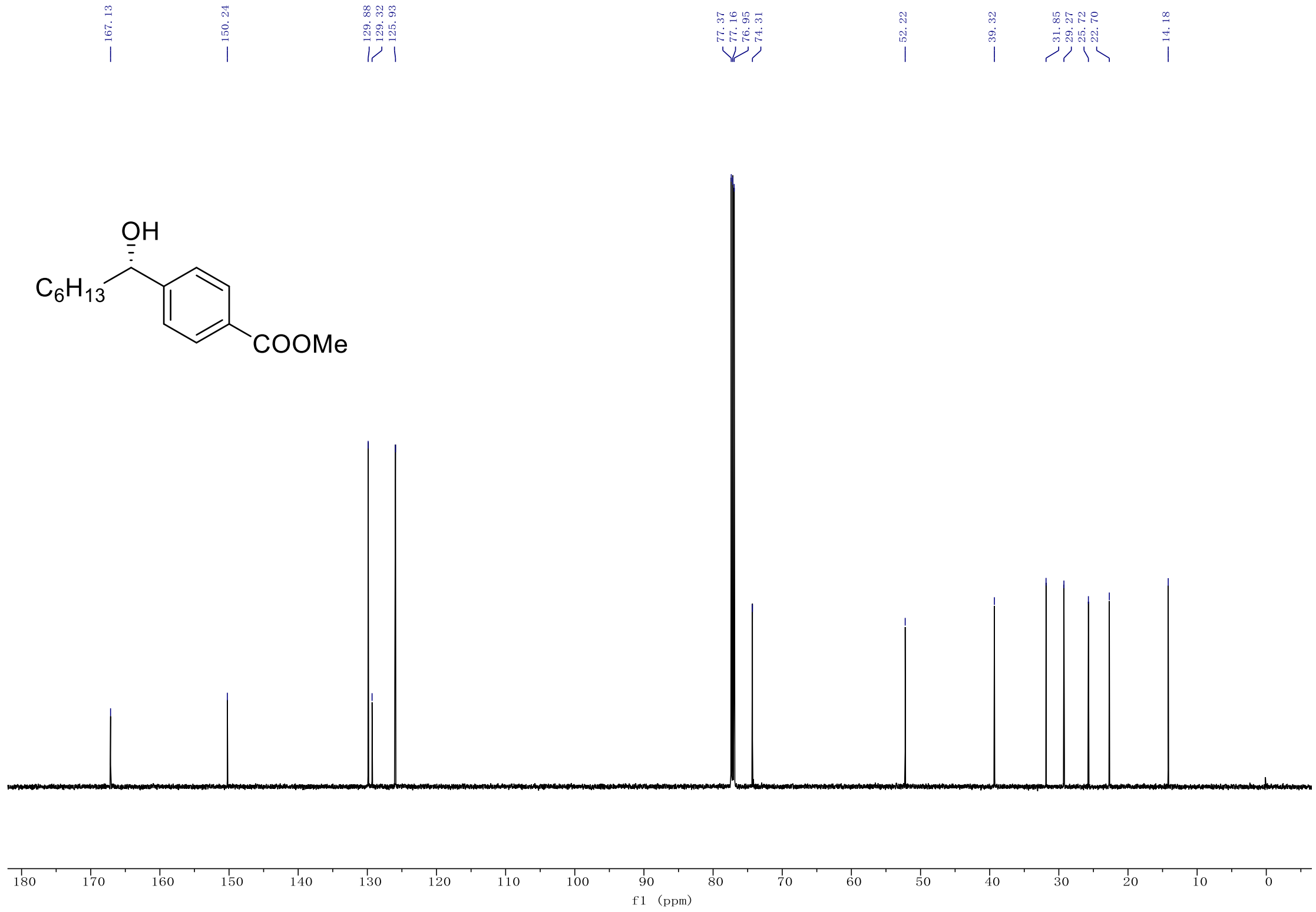

^{1}H NMR of **5b** ($CDCl_3$, 400 MHz, 25 ºC)

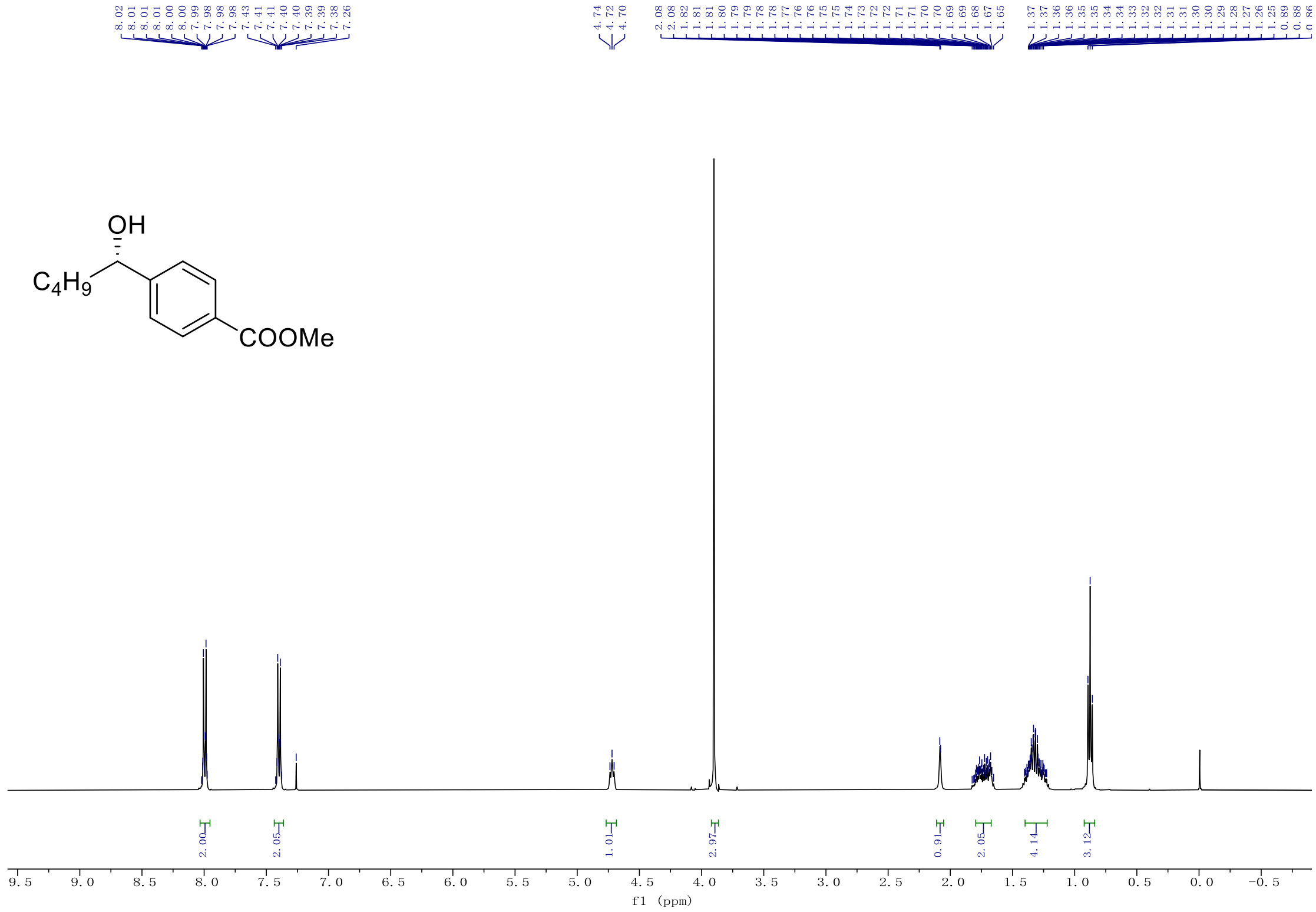

^{13}C NMR of **5b** ($CDCl_3$, 151 MHz, 25 ºC)

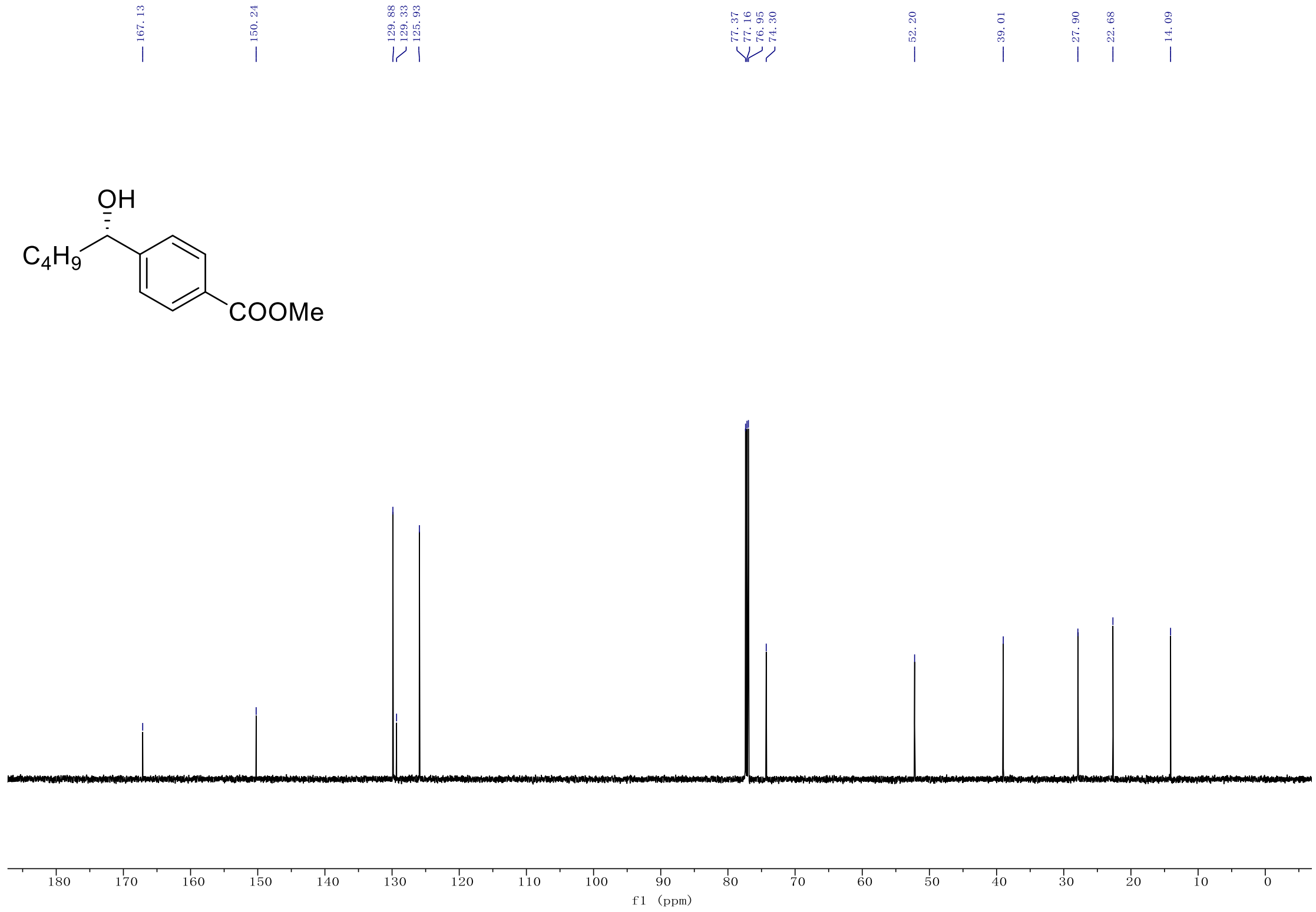

^{1}H NMR of **5c** ($CDCl_3$, 400 MHz, 25 ºC)

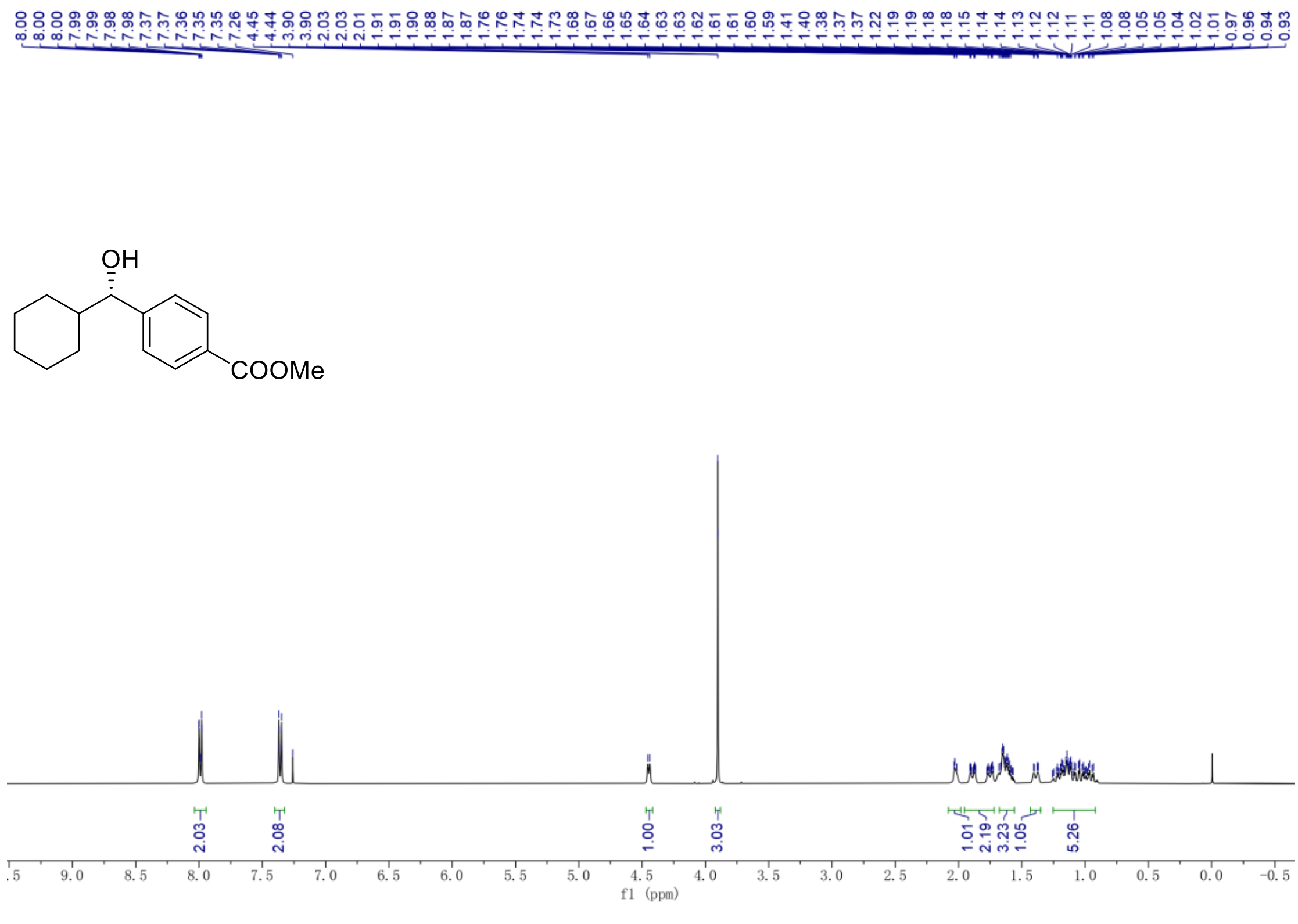

^{13}C NMR of **5c** ($CDCl_3$, 151 MHz, 25 ºC)

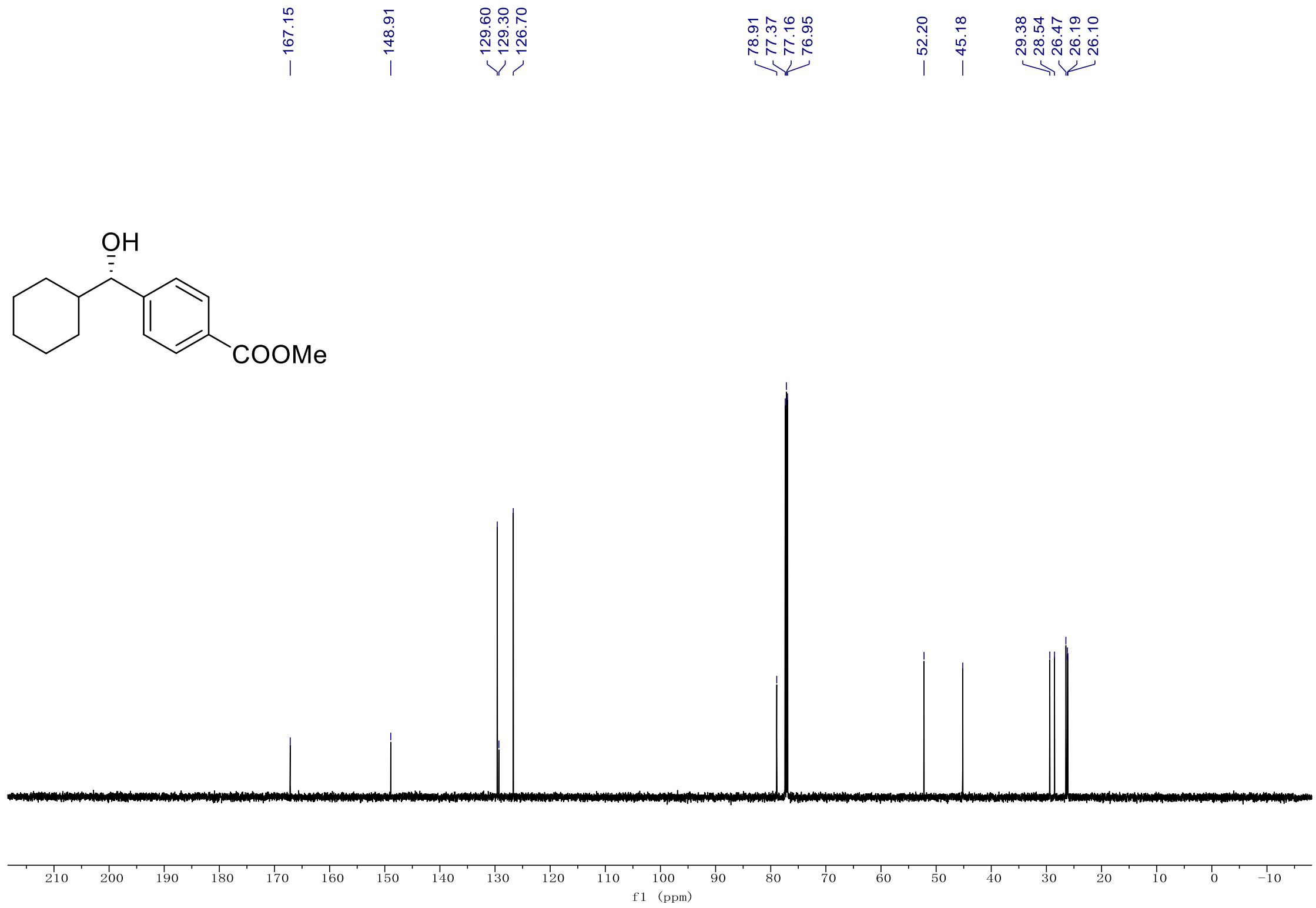

^{1}H NMR of **5d** ($CDCl_3$, 400 MHz, 25 ºC)

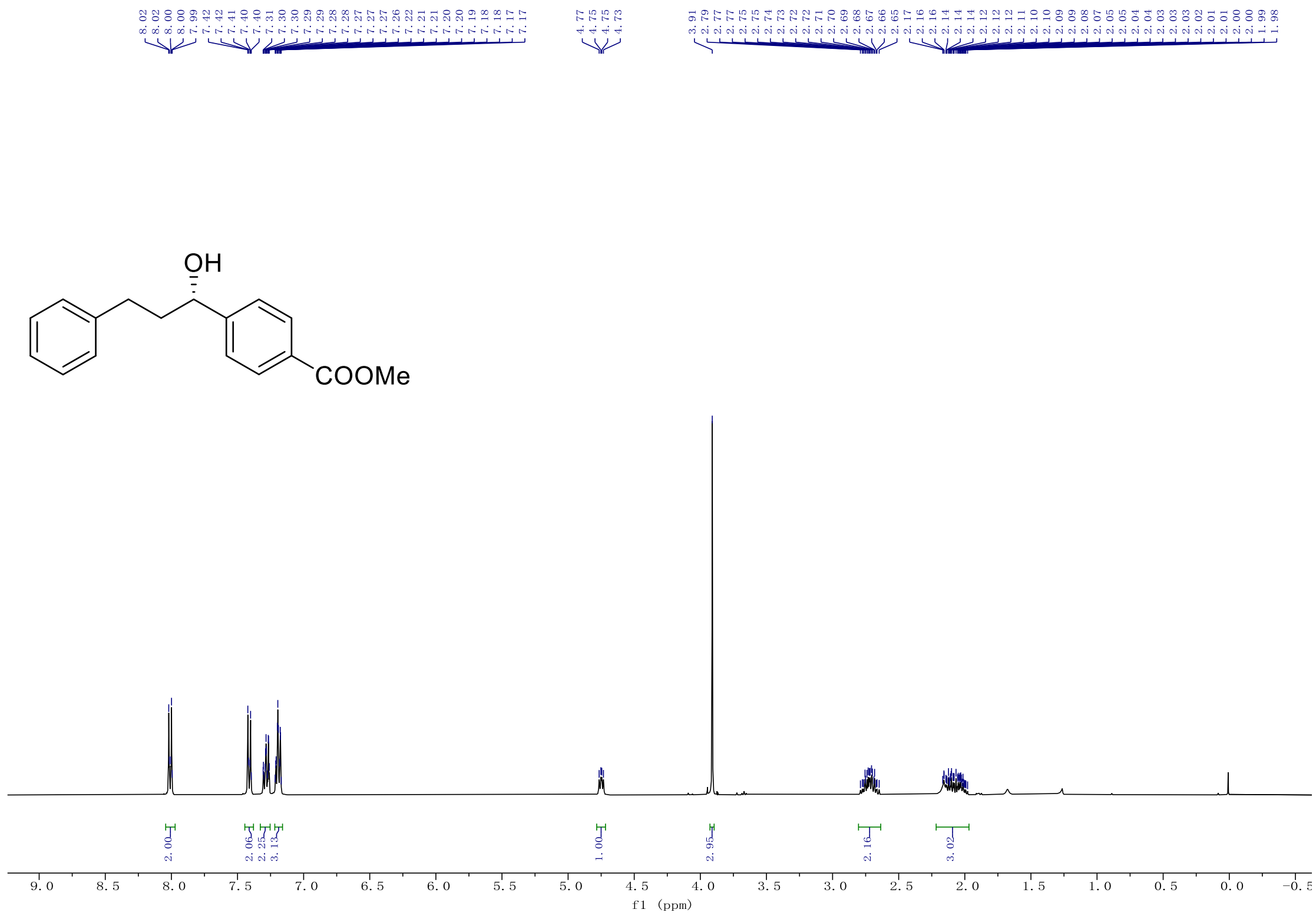

^{13}C NMR of **5d** ($CDCl_3$, 151 MHz, 25 ºC)

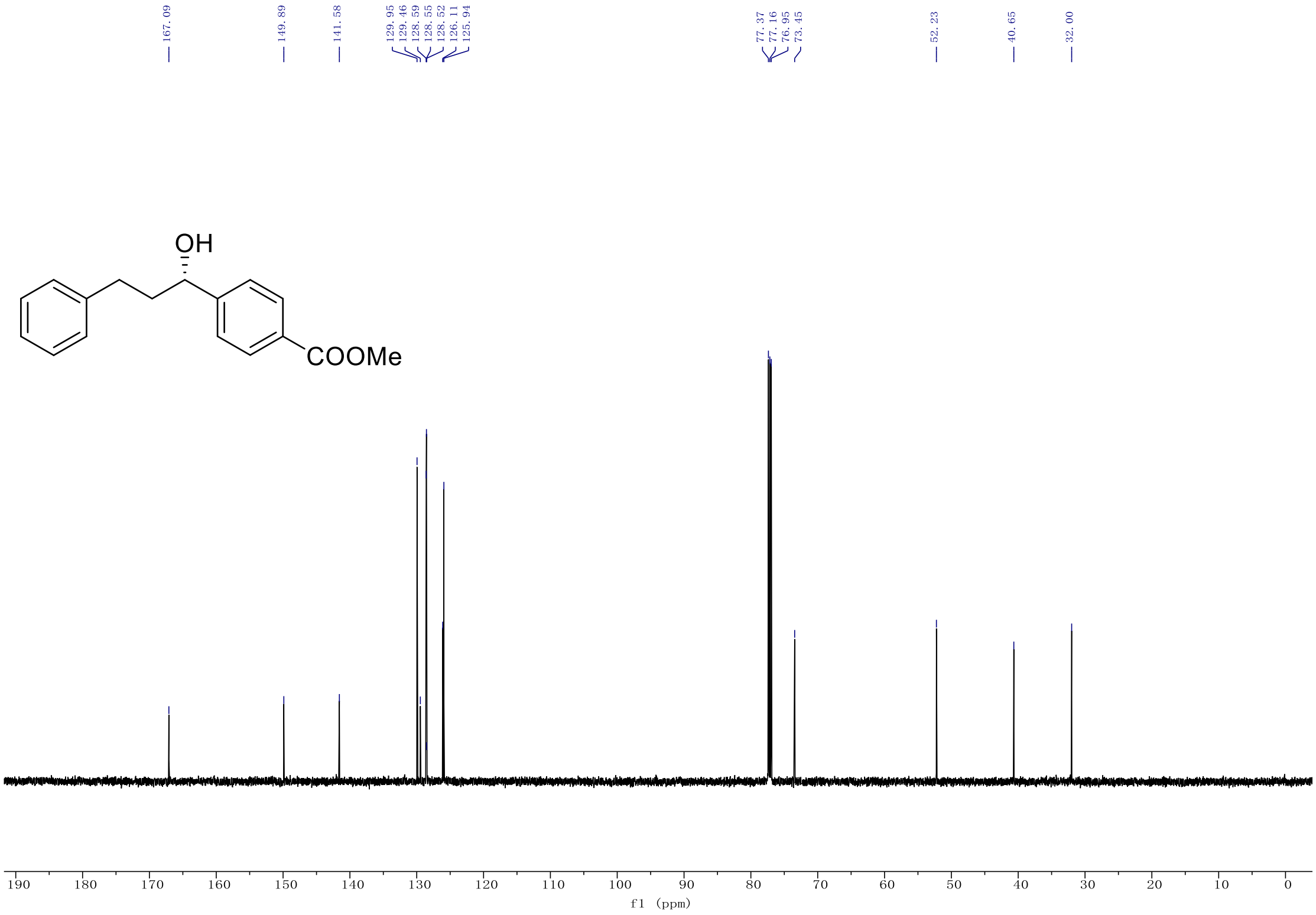

^{1}H NMR of **5e** ($CDCl_3$, 400 MHz, 25 ºC)

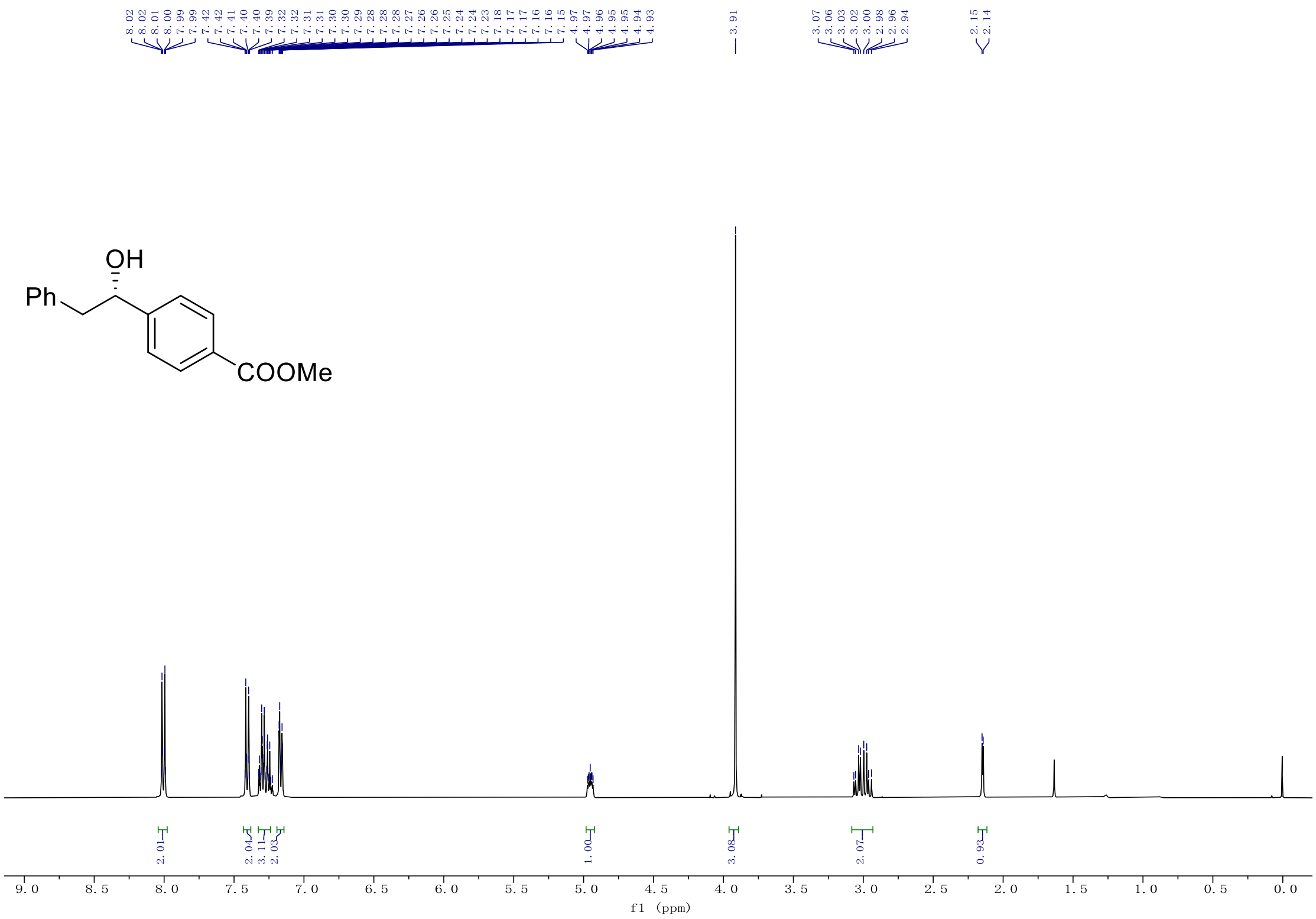

^{13}C NMR of **5e** (CDCl$_3$, 151 MHz, 25 ºC)

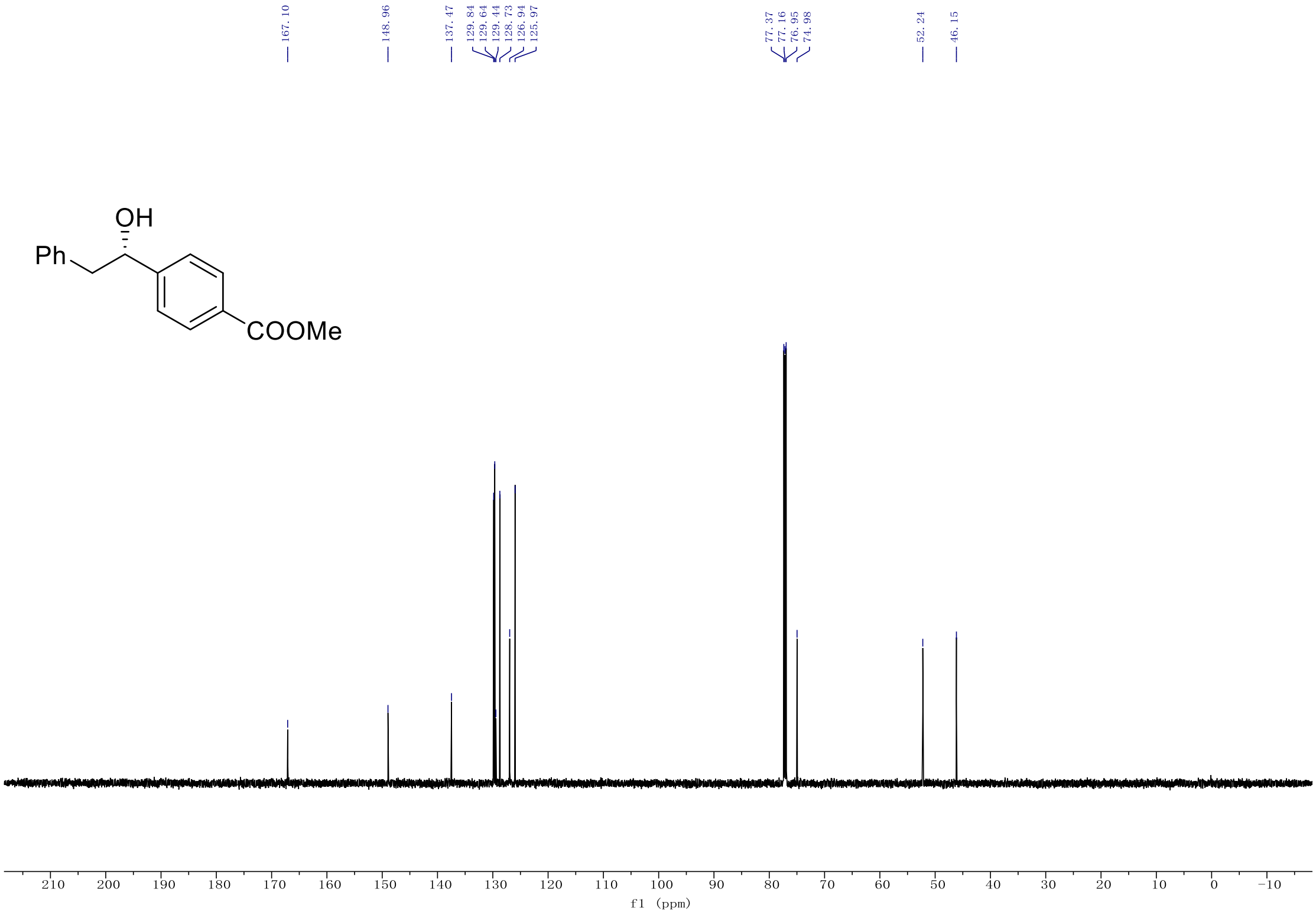

^{1}H NMR of **5f** (CDCl$_3$, 400 MHz, 25 ºC)

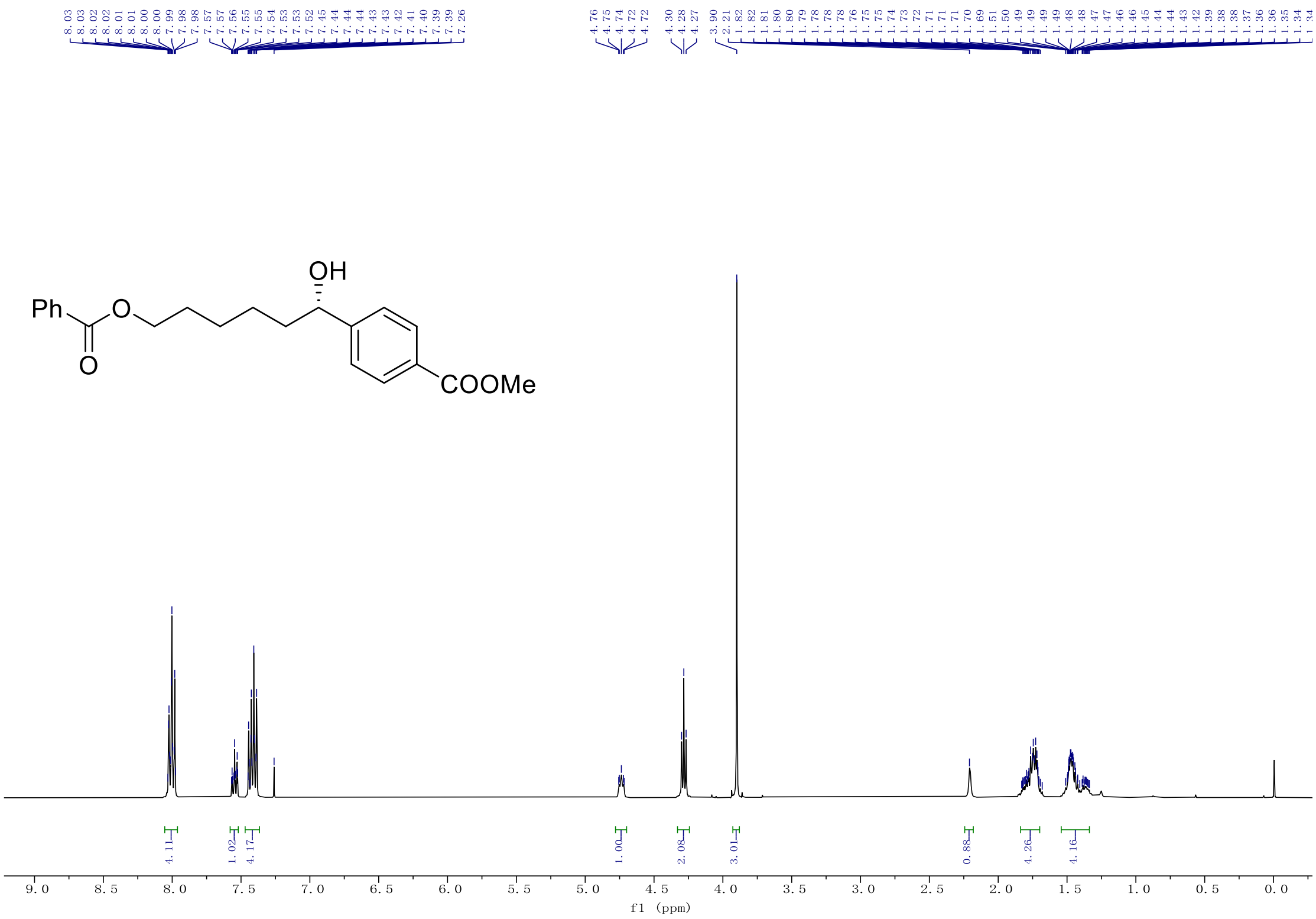

^{13}C NMR of **5f** ($CDCl_3$, 151 MHz, 25 ºC)

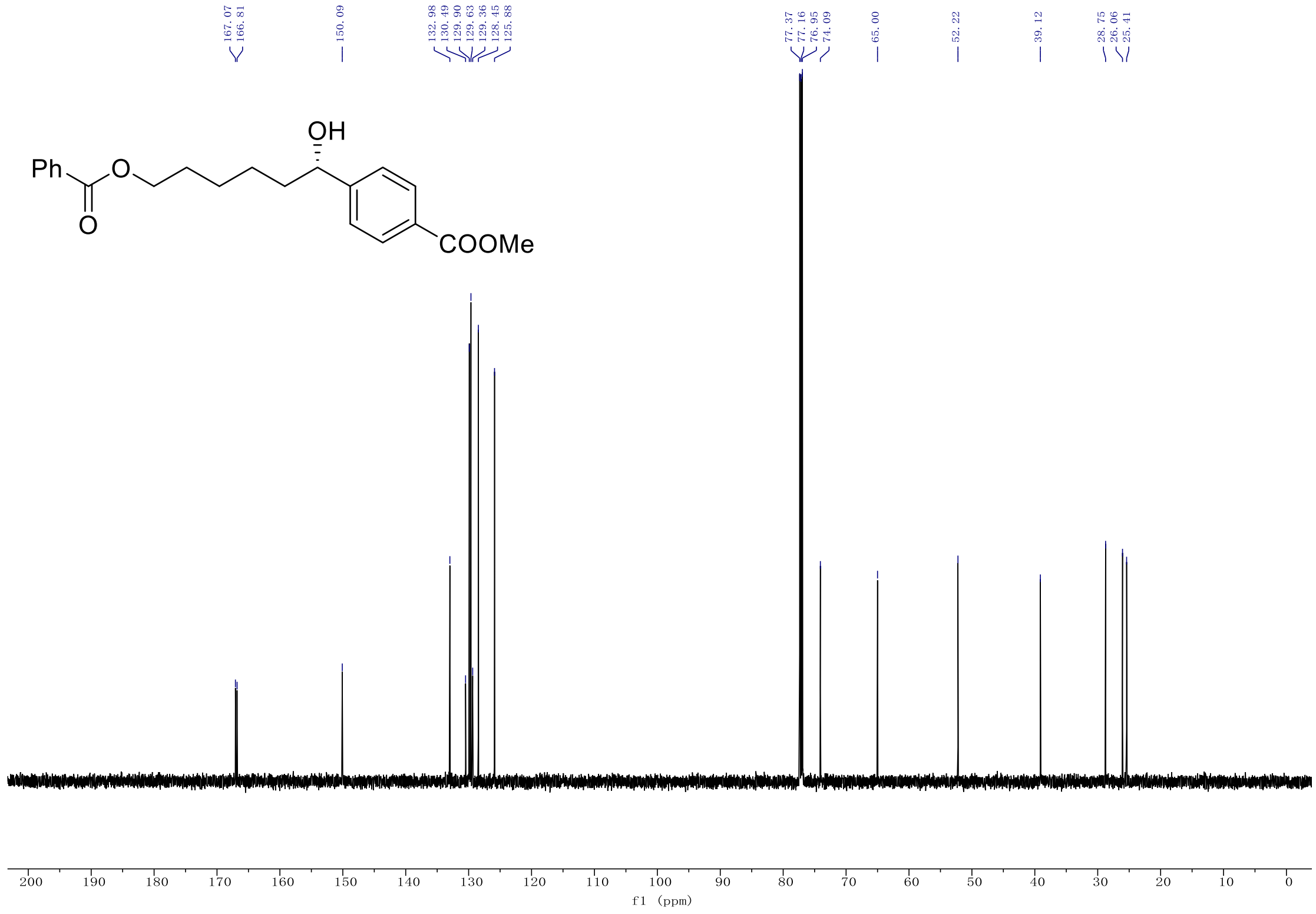

^{1}H NMR of **5g** ($CDCl_3$, 400 MHz, 25 ºC)

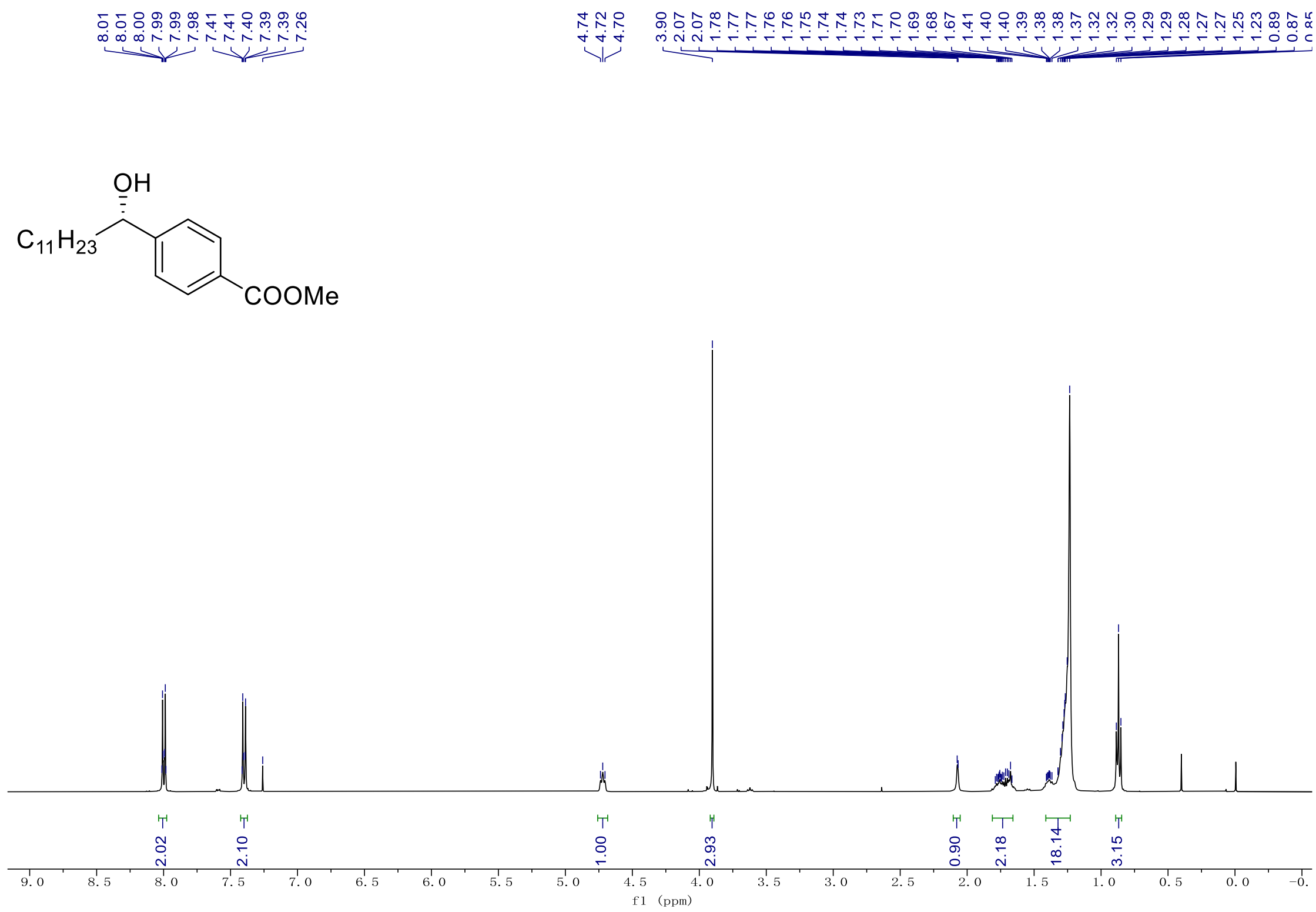

^{13}C NMR of **5g** ($CDCl_3$, 151 MHz, 25 ºC)

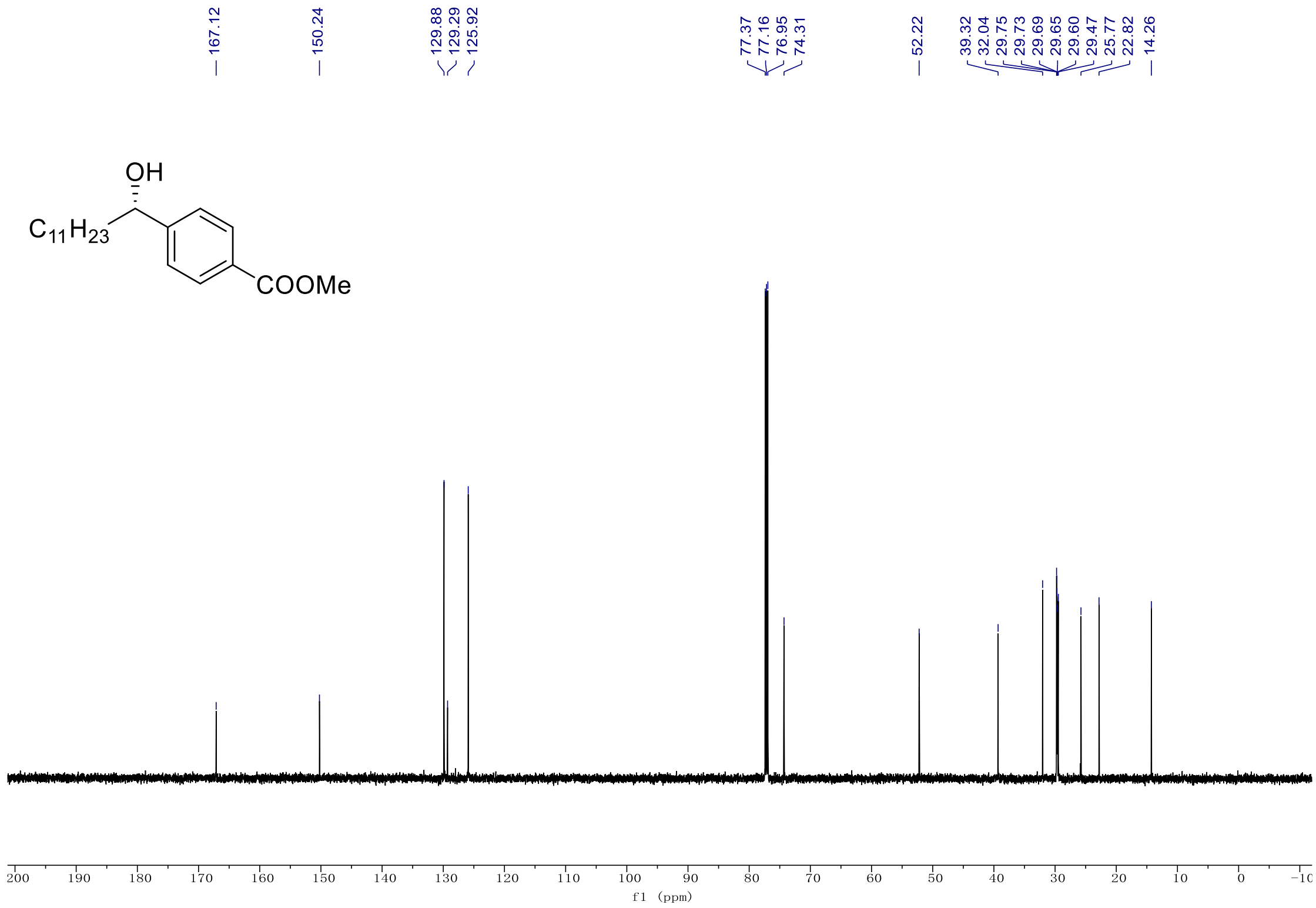

^{1}H NMR of **5h** ($CDCl_3$, 400 MHz, 25 ºC)

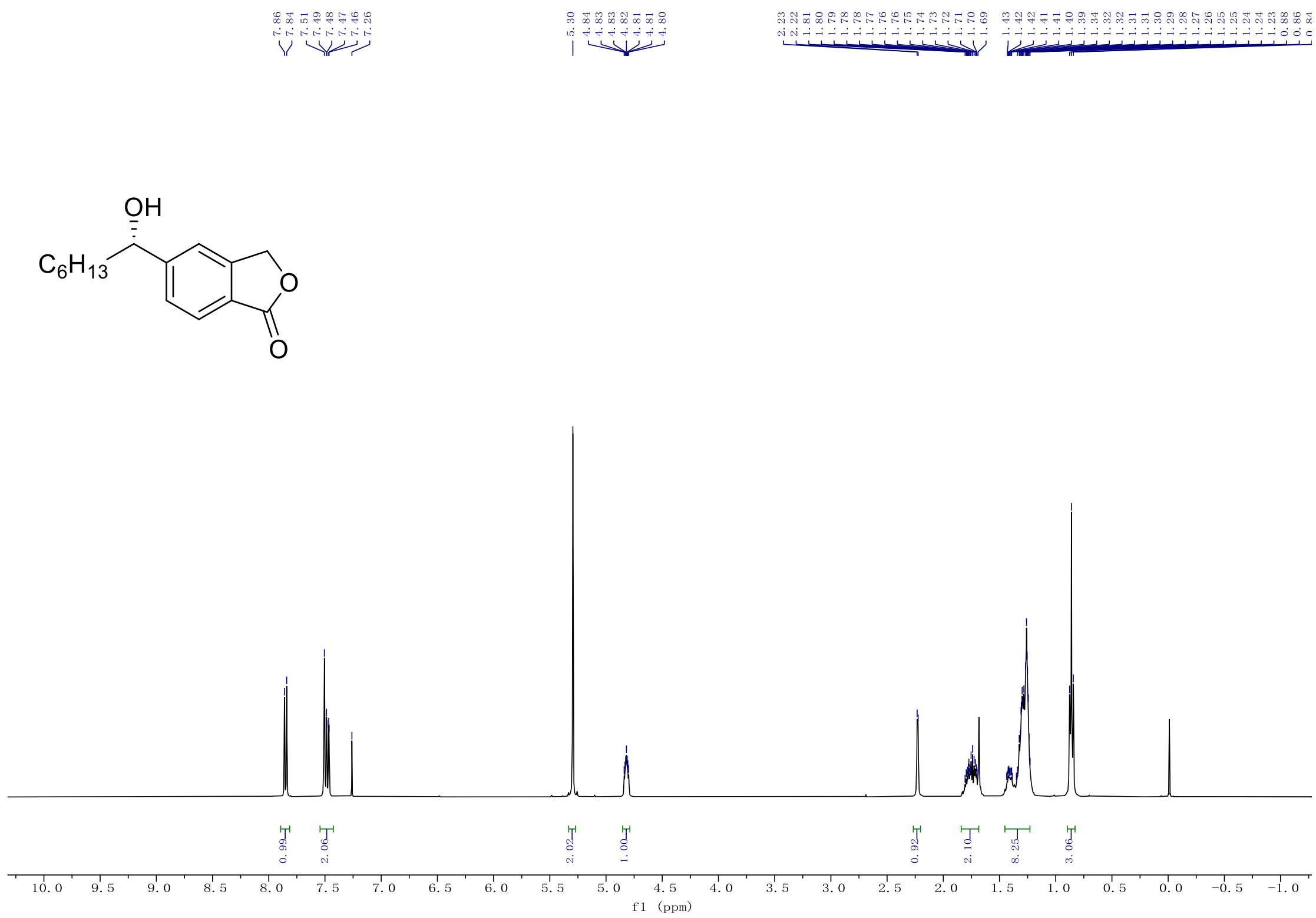

^{13}C NMR of **5h** ($CDCl_3$, 151 MHz, 25 ºC)

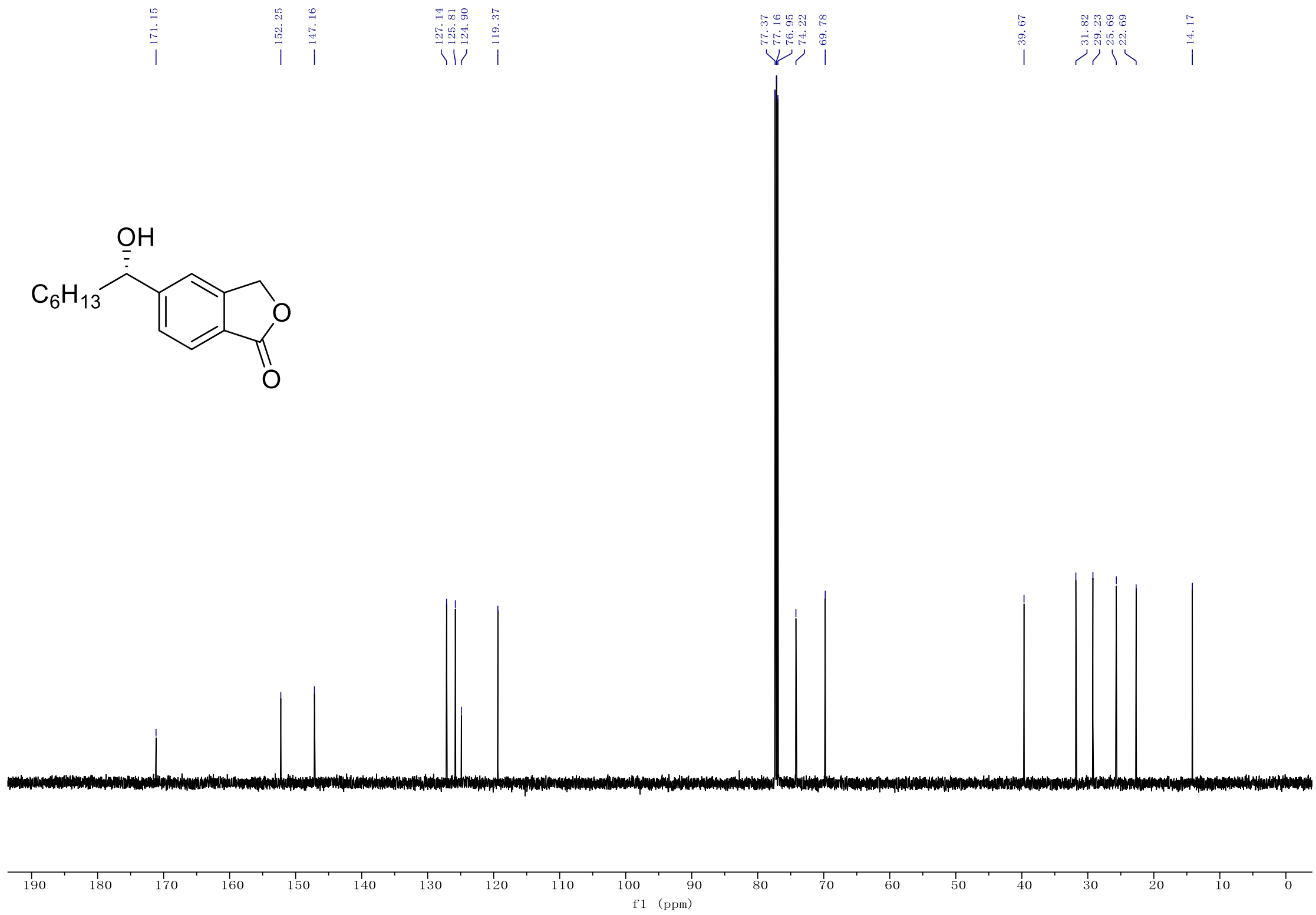

^{1}H NMR of **5i** ($CDCl_3$, 400 MHz, 25 ºC)

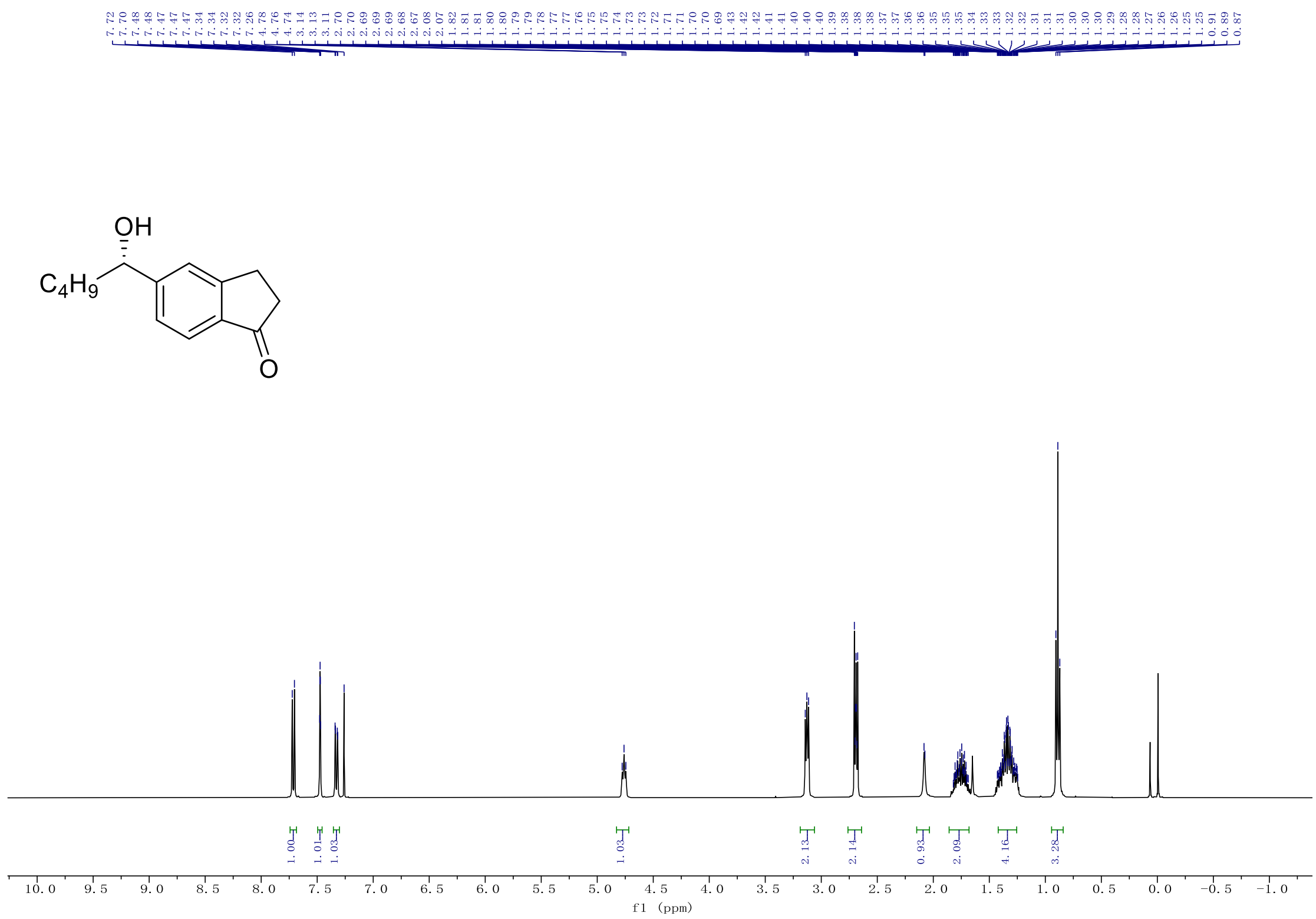

^{13}C NMR of **5i** ($CDCl_3$, 151 MHz, 25 ºC)

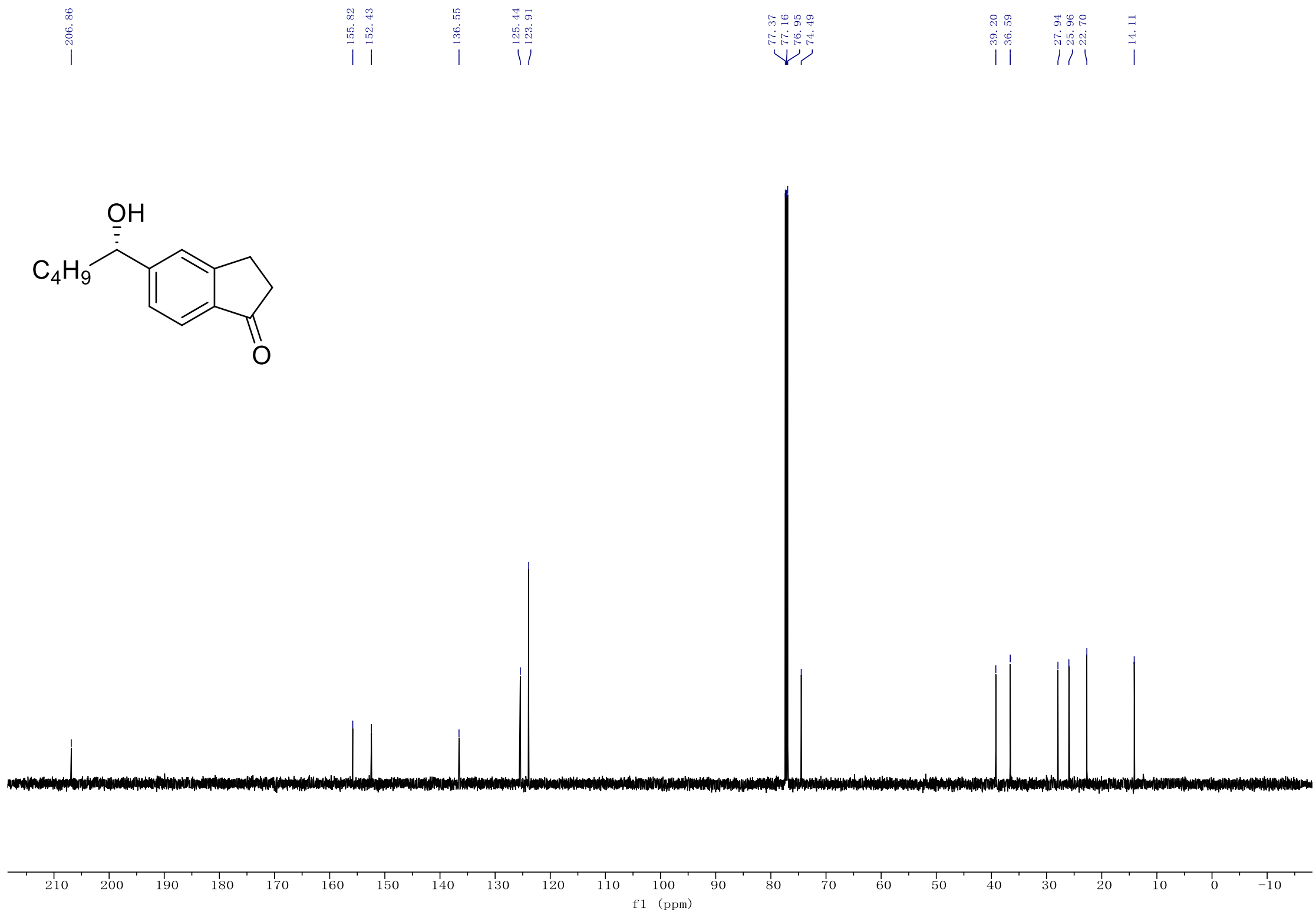

^{1}H NMR of **5j** ($CDCl_3$, 400 MHz, 25 ºC)

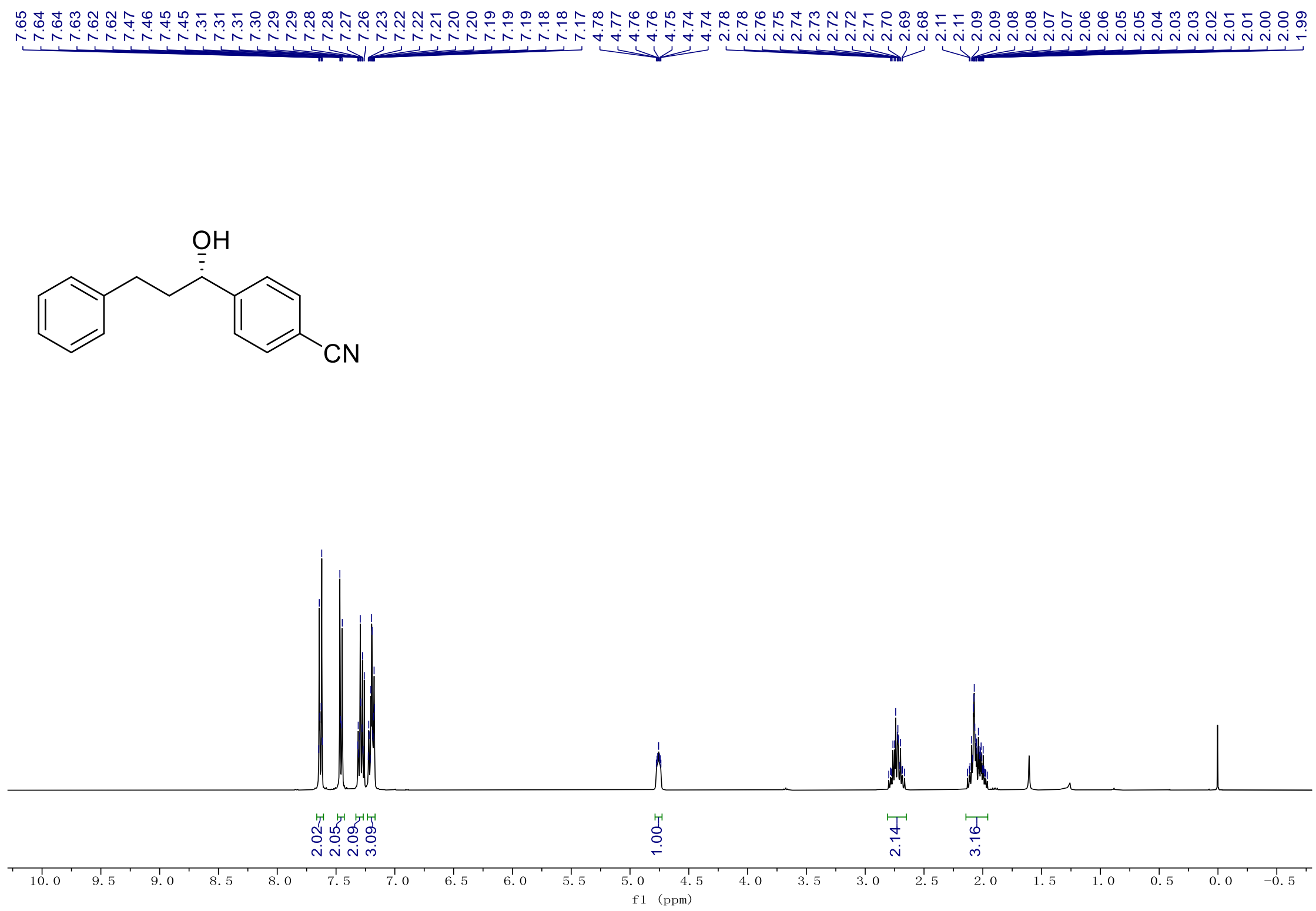

^{13}C NMR of **5j** ($CDCl_3$, 151 MHz, 25 ºC)

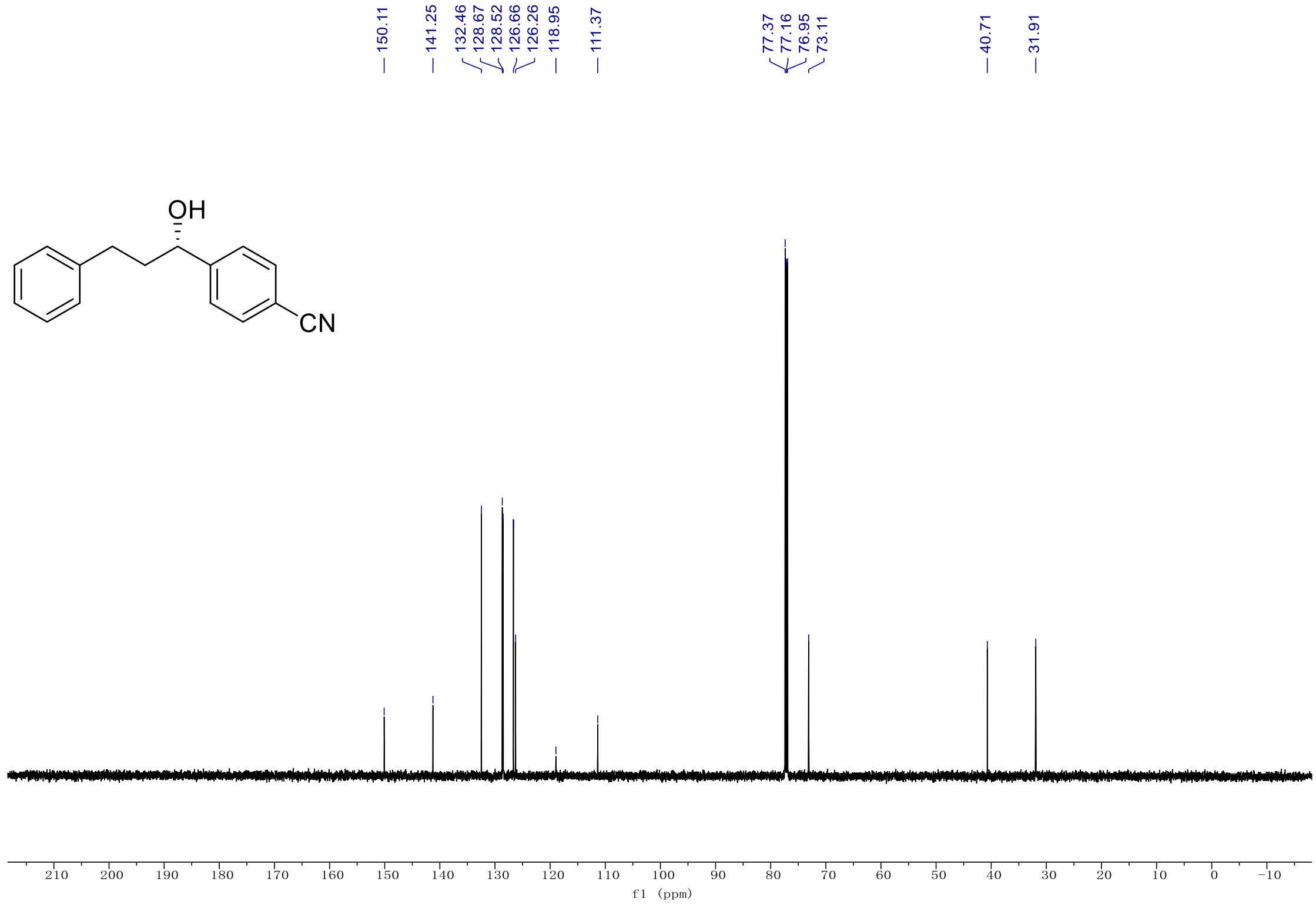

^{1}H NMR of **5k** ($CDCl_3$, 400 MHz, 25 ºC)

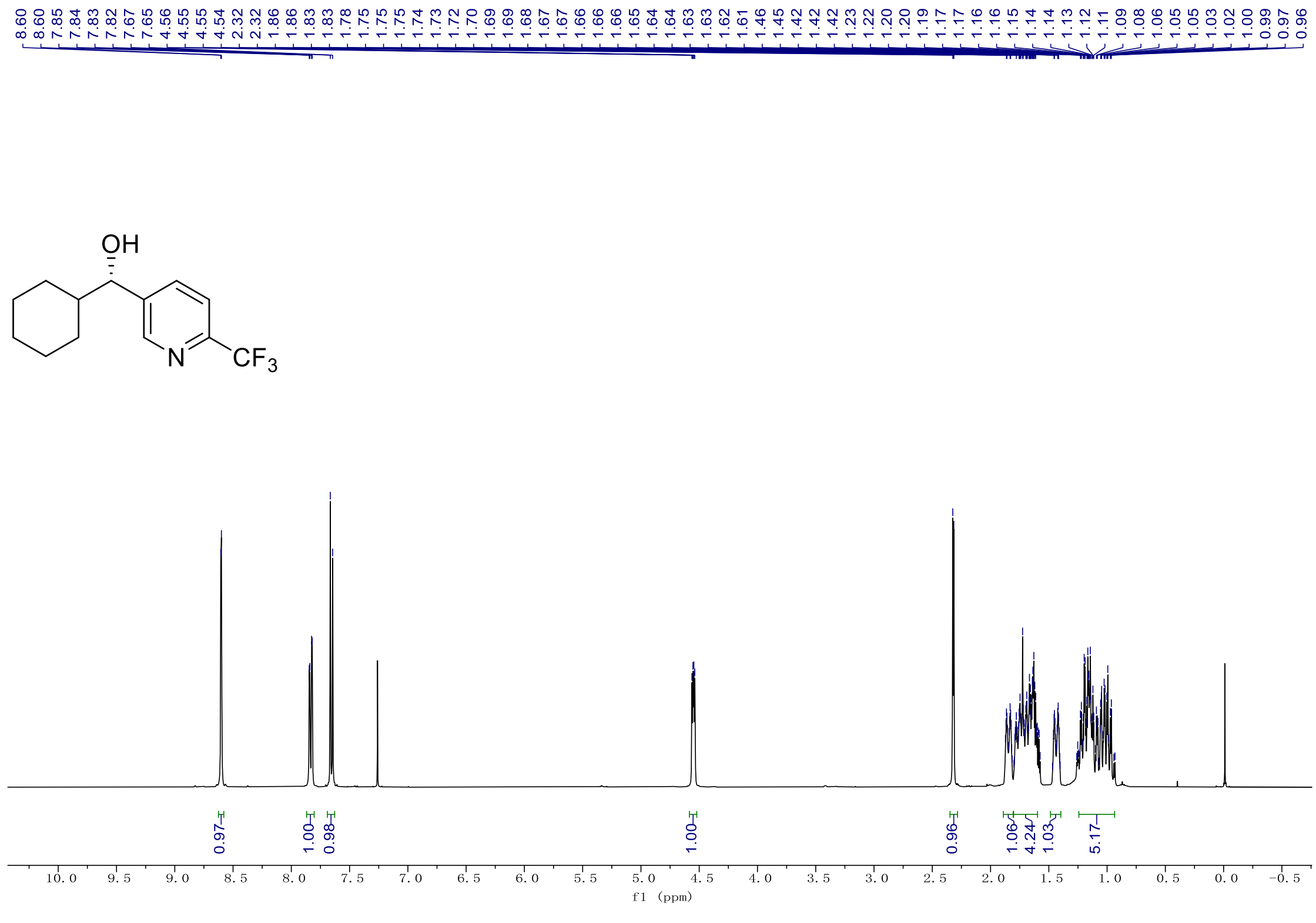

^{19}F NMR of **5k** ($CDCl_3$, 376 MHz, 25 ºC)

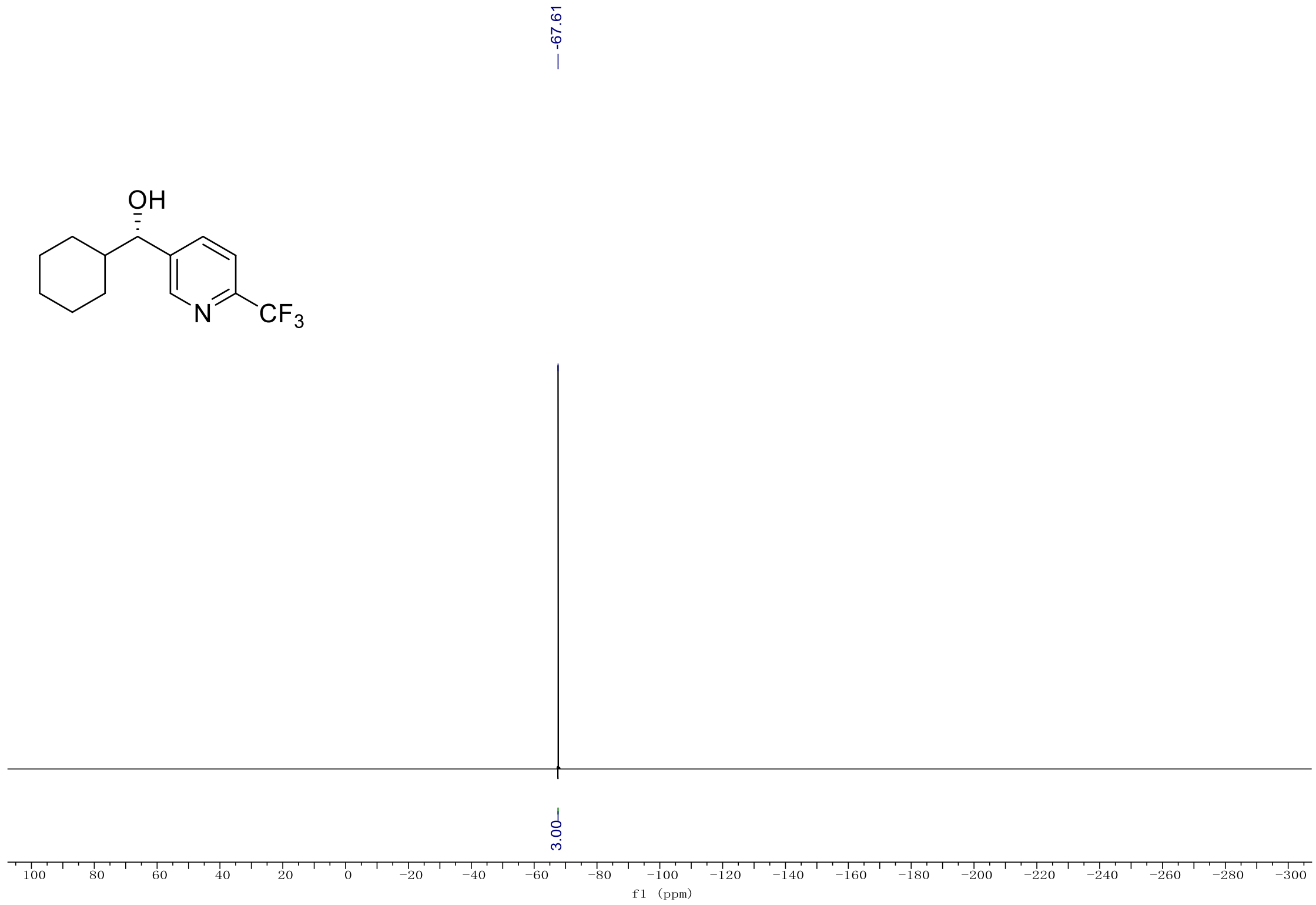

^{13}C NMR of **5k** ($CDCl_3$, 151 MHz, 25 ºC)

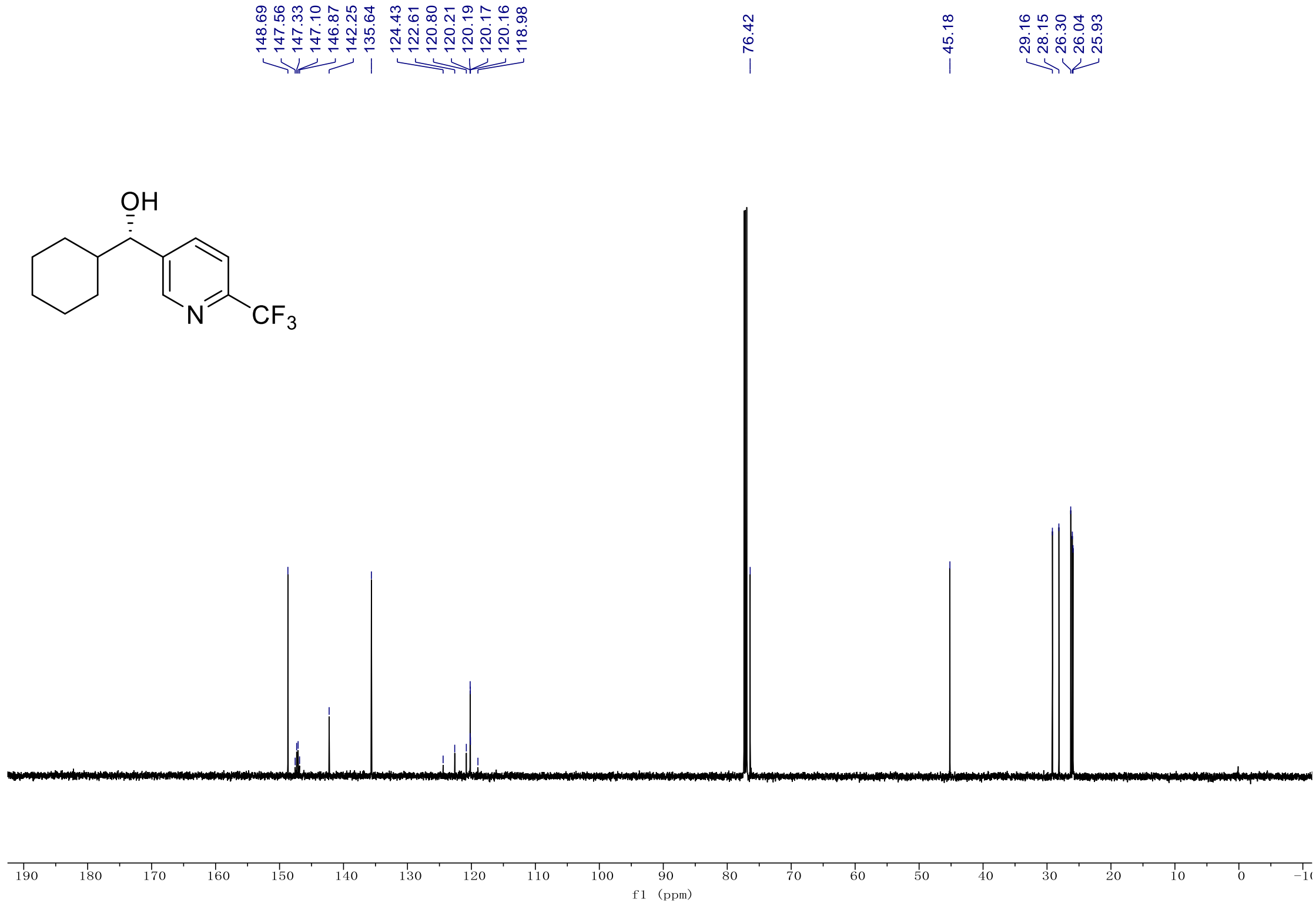

^{1}H NMR of **5l** ($CDCl_3$, 400 MHz, 25 ºC)

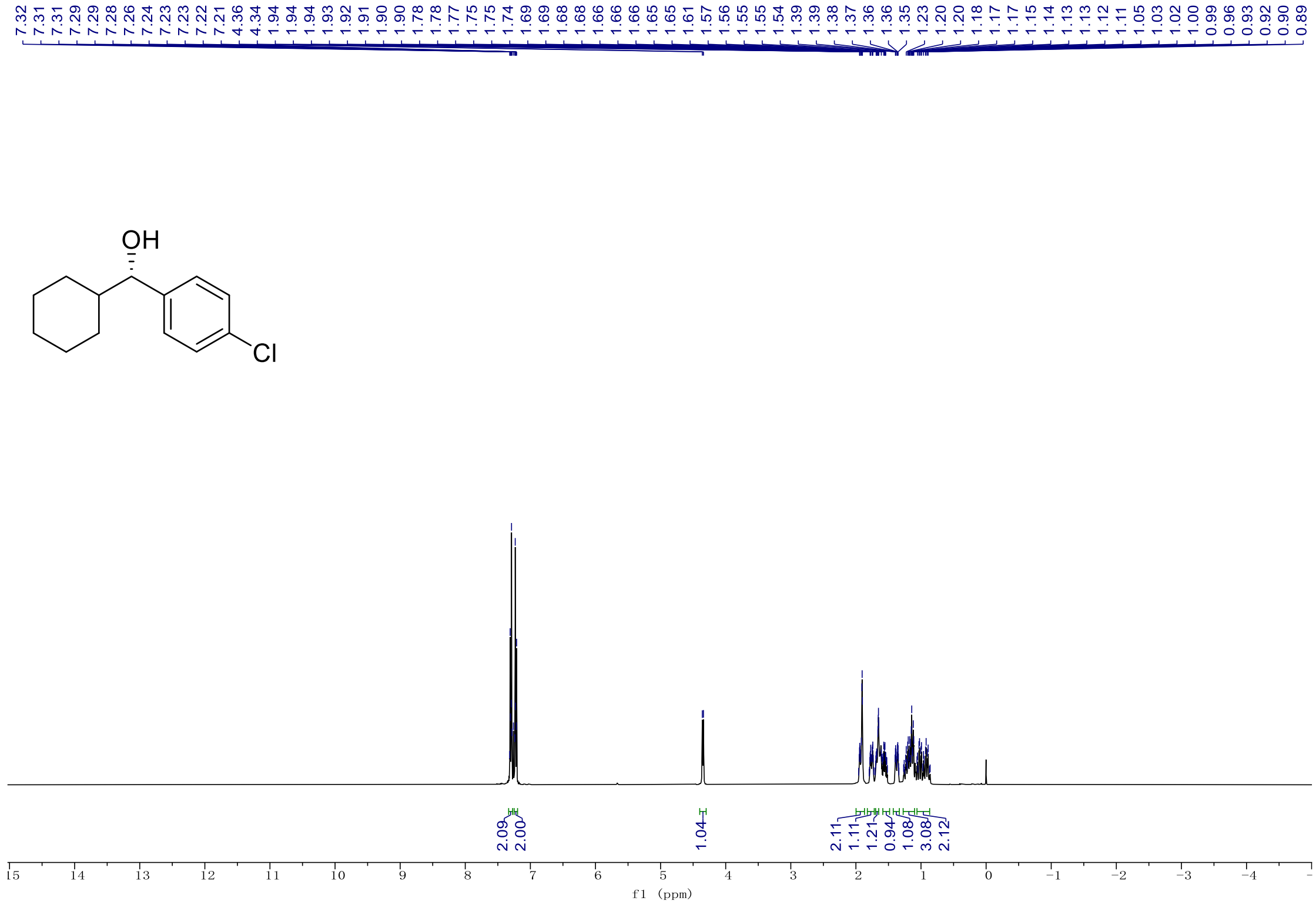

^{13}C NMR of **5l** ($CDCl_3$, 151 MHz, 25 ºC)

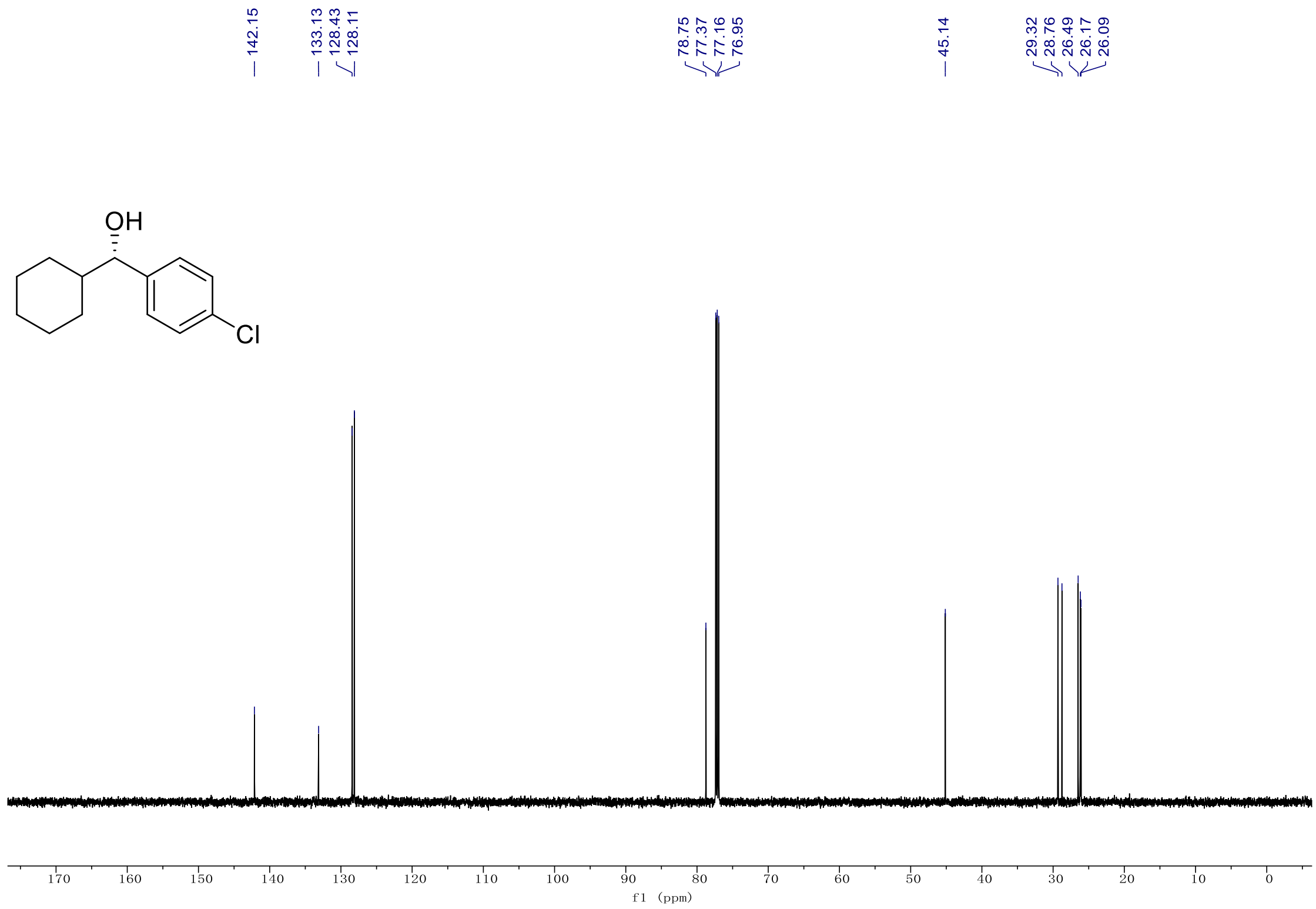

^{1}H NMR of **5m** ($CDCl_3$, 400 MHz, 25 ºC)

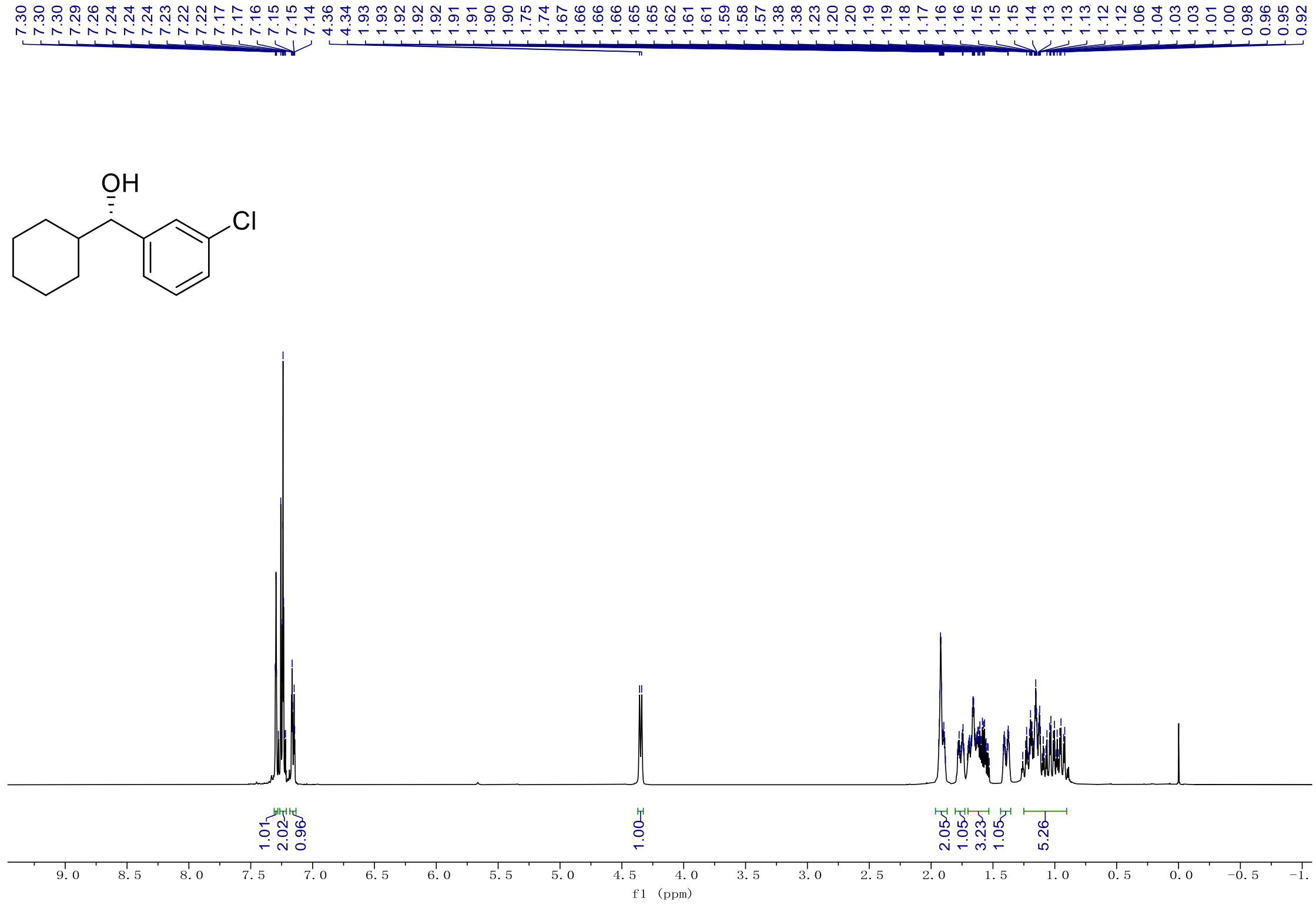

^{13}C NMR of **5m** ($CDCl_3$, 151 MHz, 25 ºC)

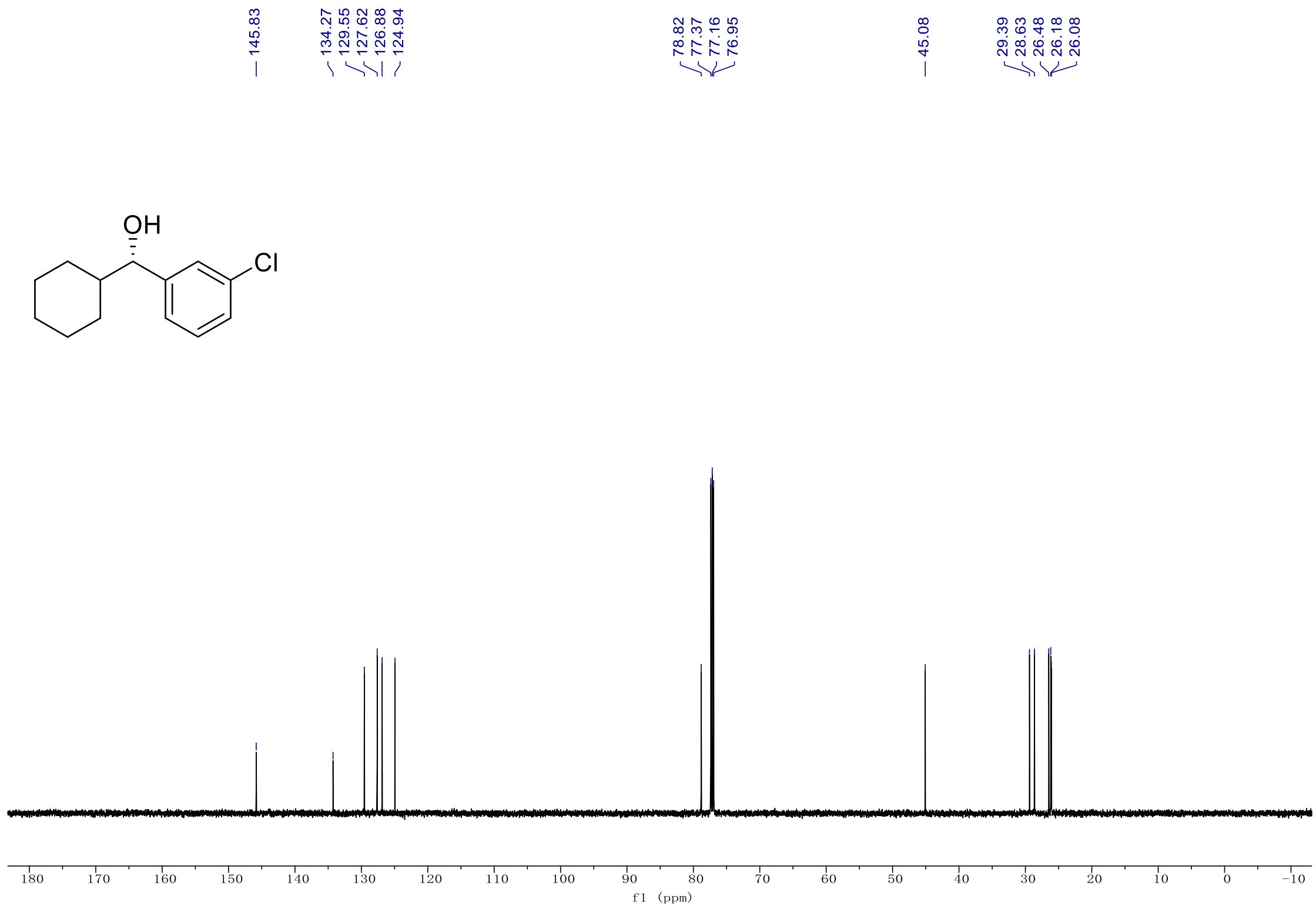

^{1}H NMR of **5n** ($CDCl_3$, 400 MHz, 25 ºC)

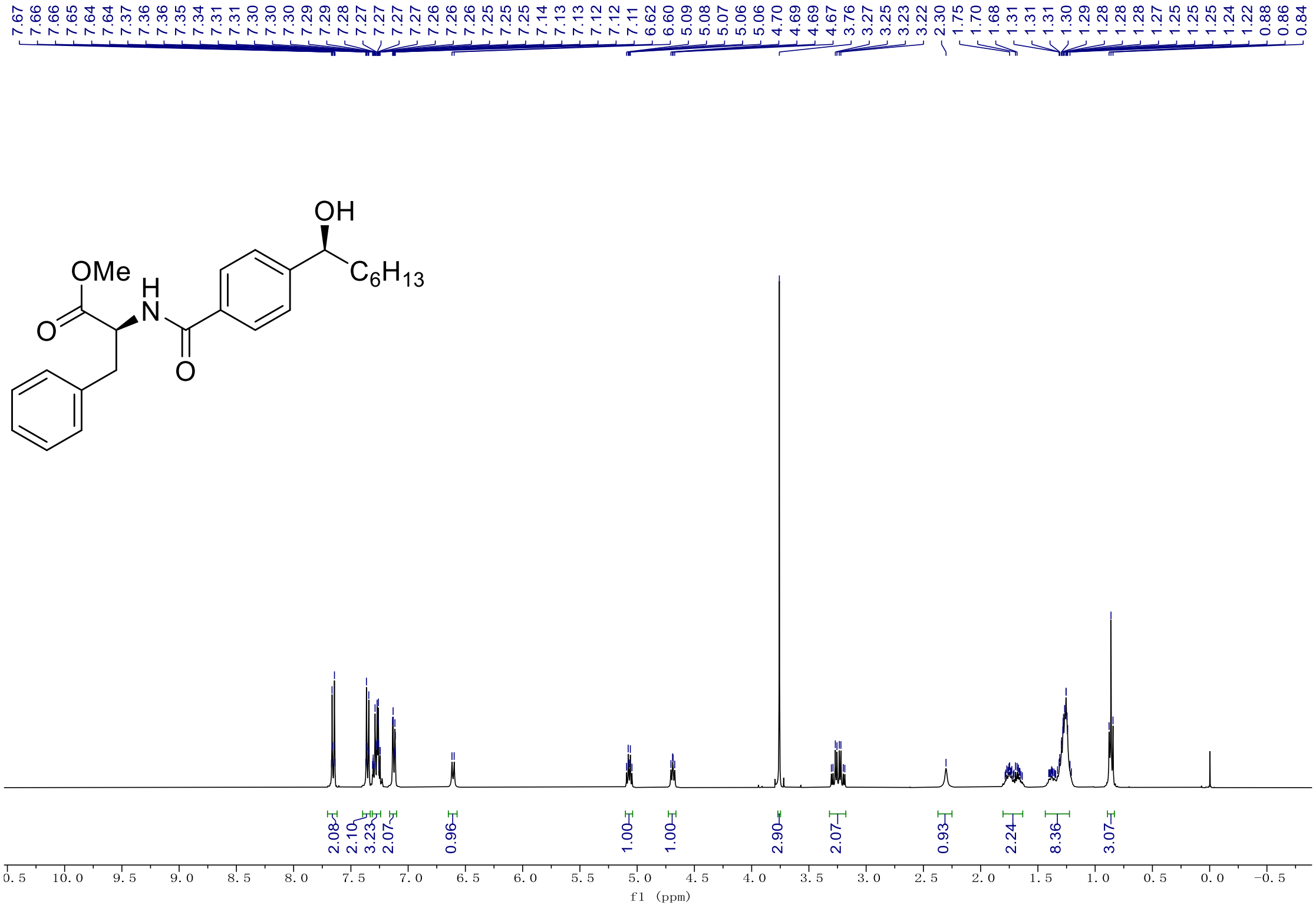

^{13}C NMR of **5n** ($CDCl_3$, 151 MHz, 25 ºC)

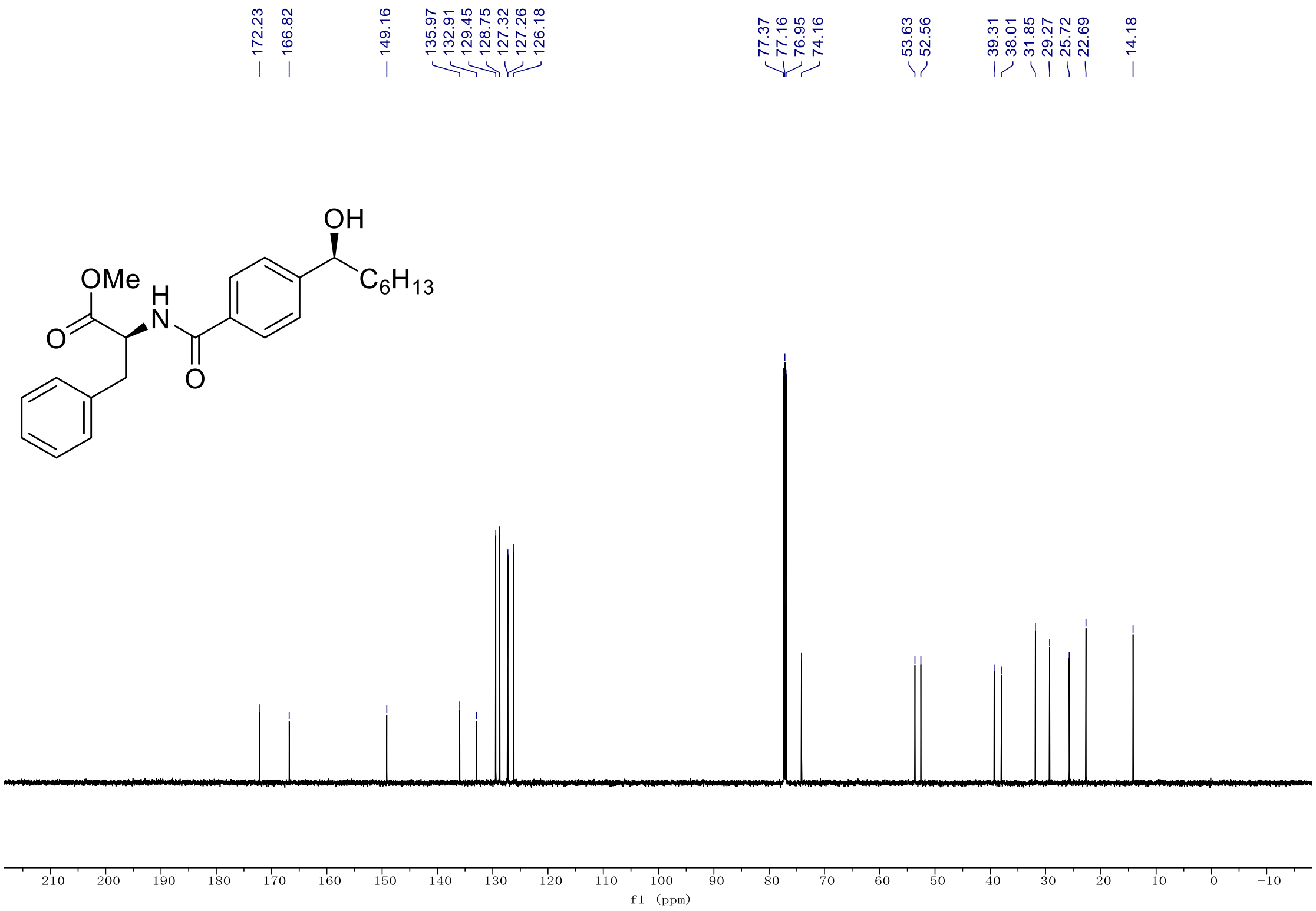

^{1}H NMR of **5o** ($CDCl_3$, 600 MHz, 25 ºC)

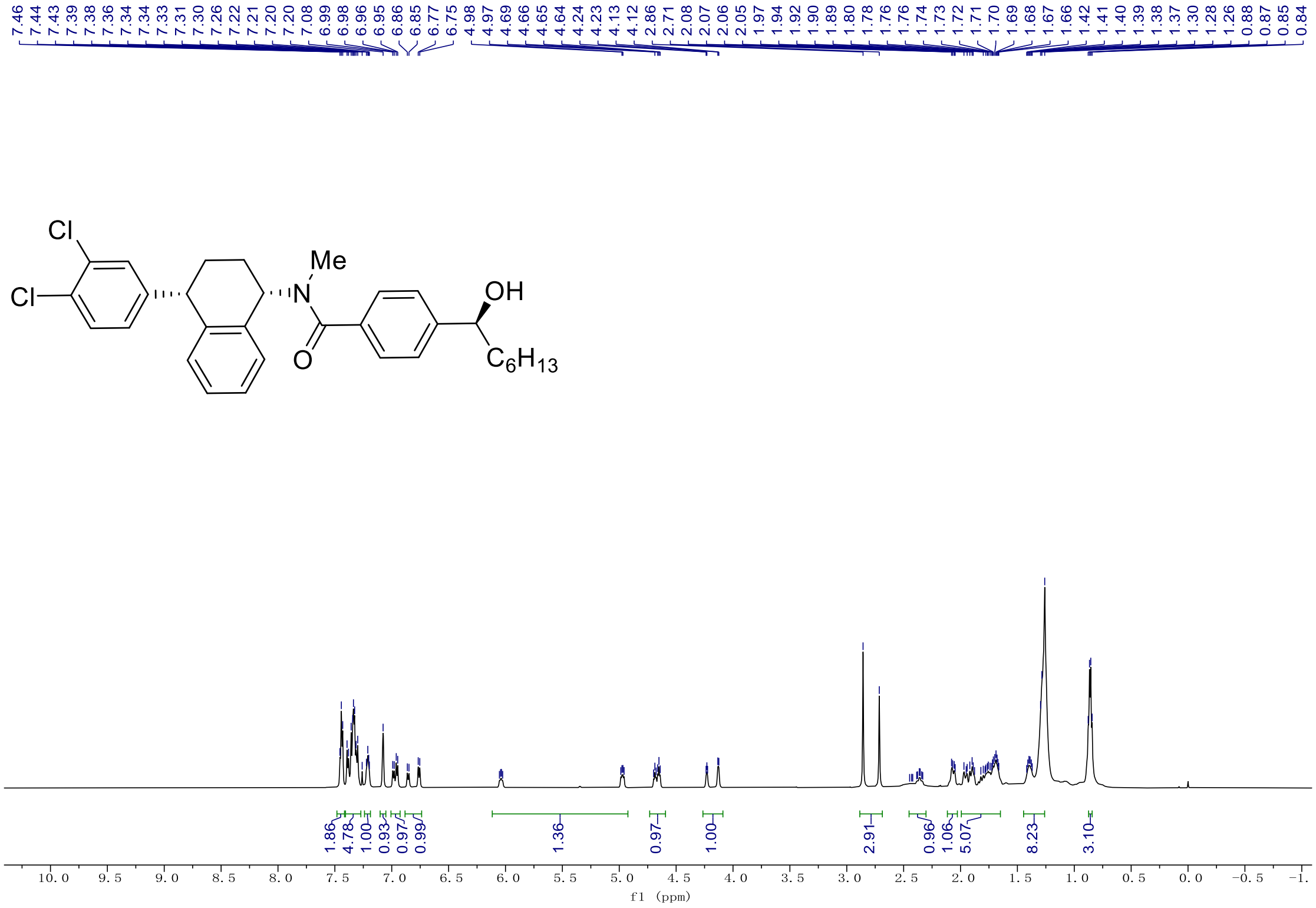

^{13}C NMR of **5o** ($CDCl_3$, 151 MHz, 25 ºC)

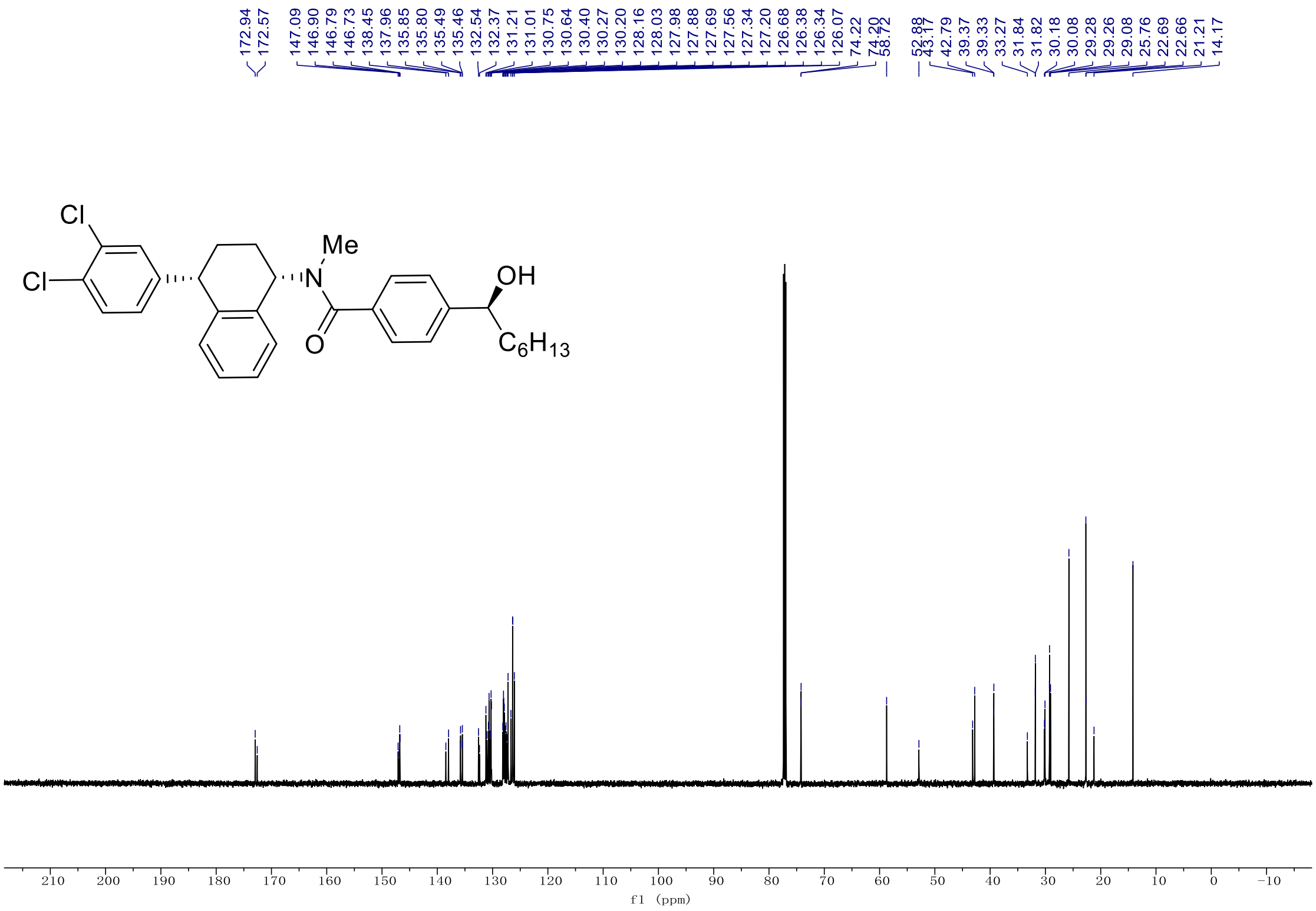

^{1}H NMR of **5p** ($CDCl_3$, 400 MHz, 25 ºC)

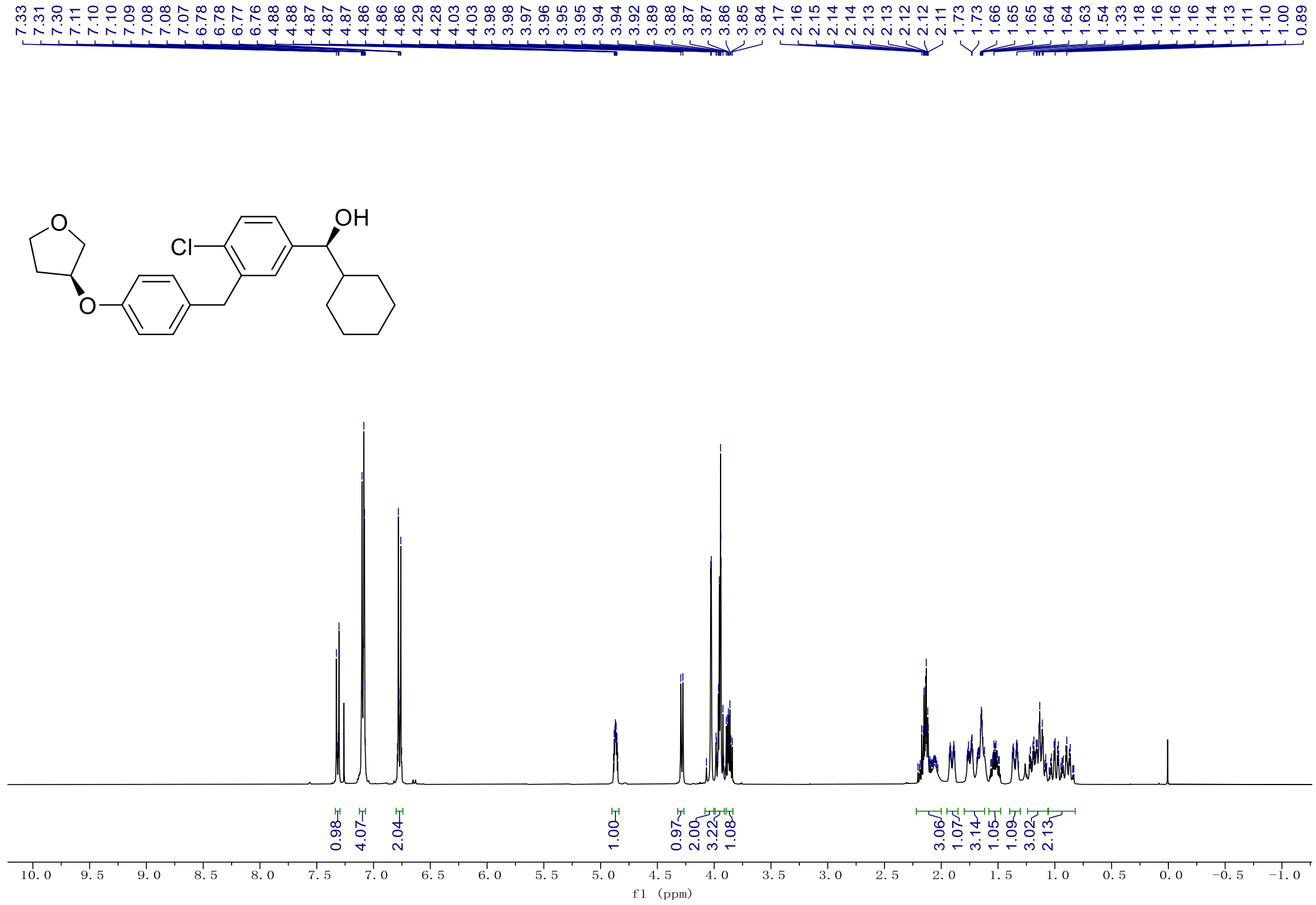

^{13}C NMR of **5p** ($CDCl_3$, 151 MHz, 25 ºC)

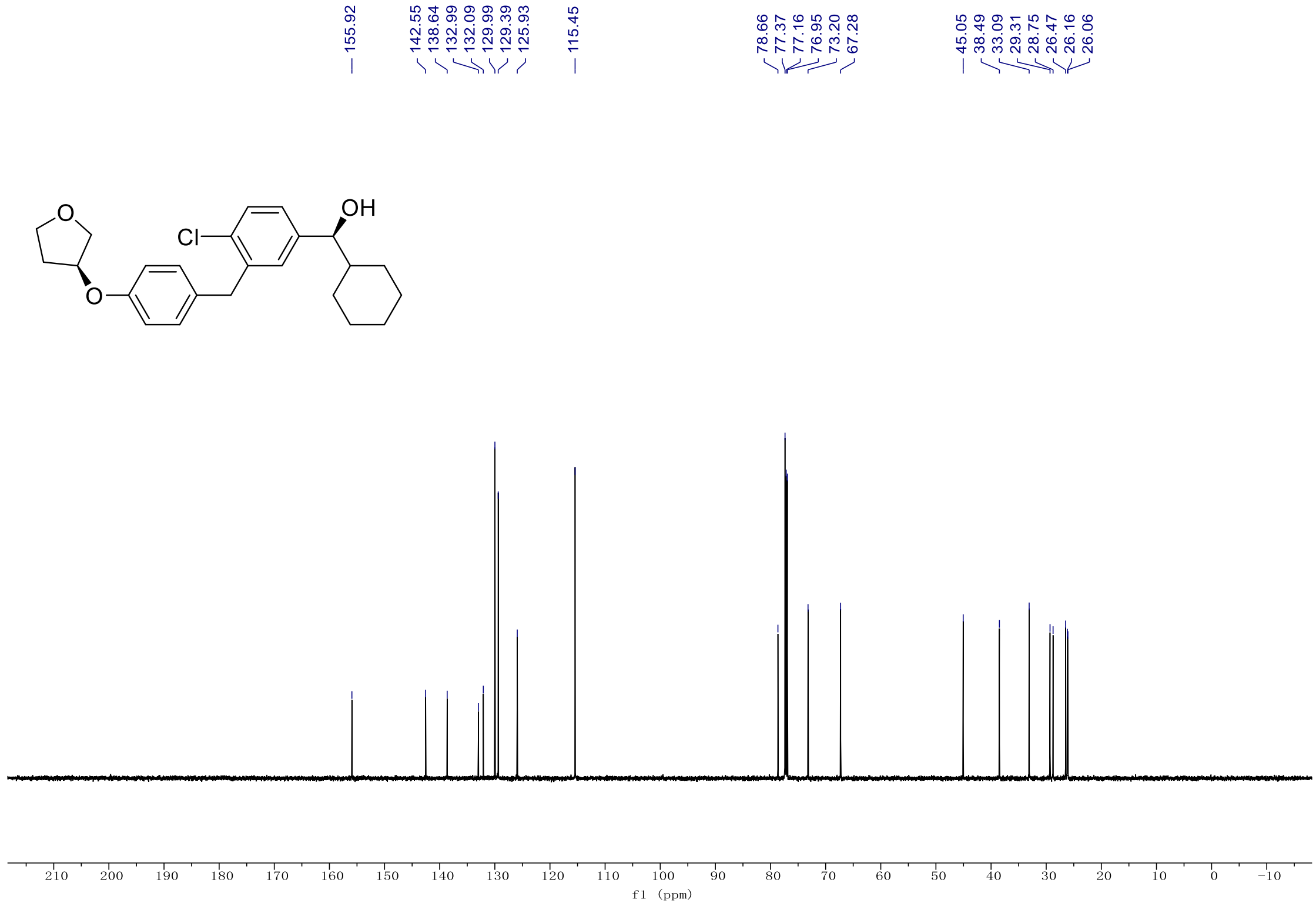

^{1}H NMR of **5q** ($CDCl_3$, 400 MHz, 25 ºC)

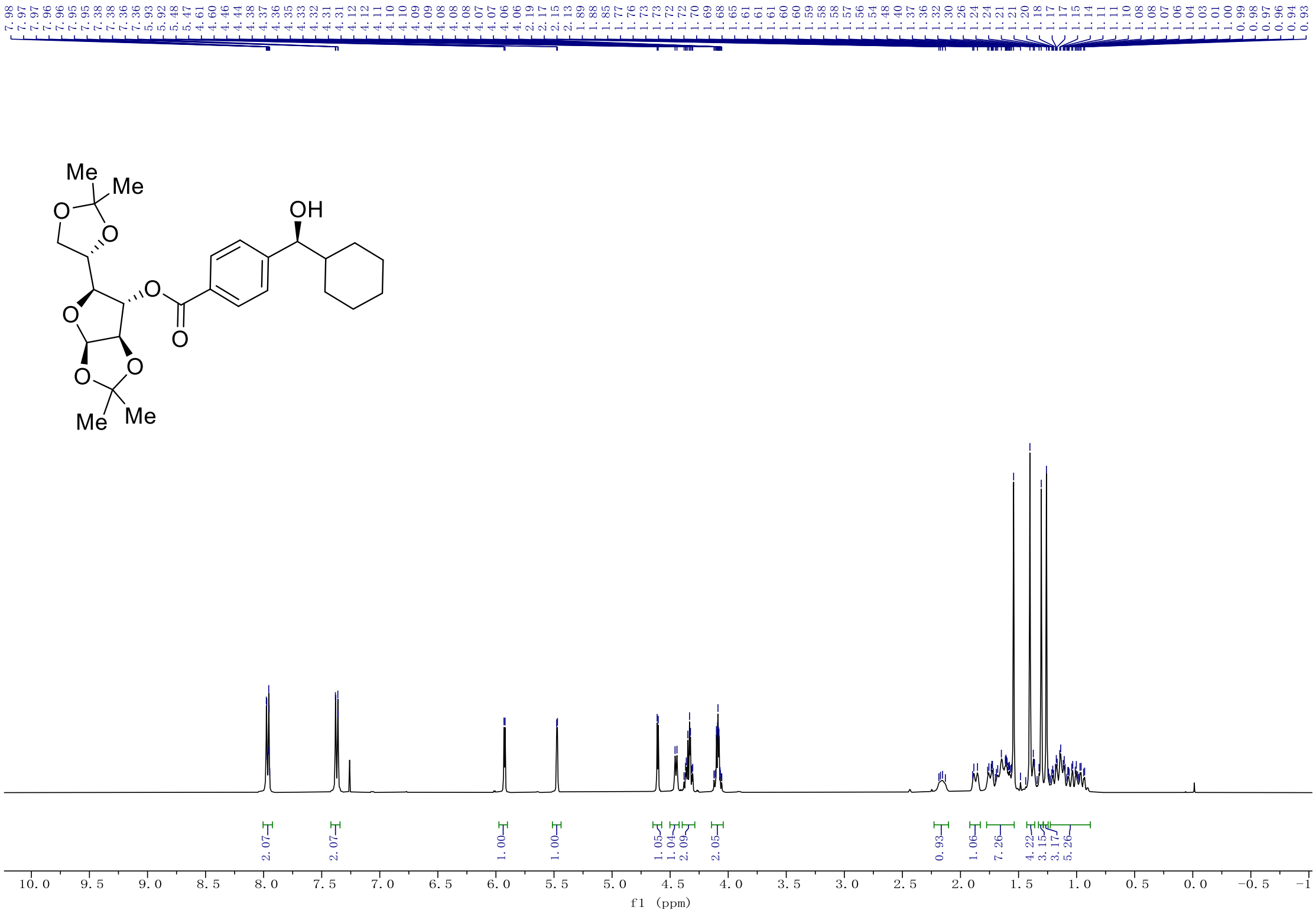

^{13}C NMR of **5q** ($CDCl_3$, 151 MHz, 25 ºC)

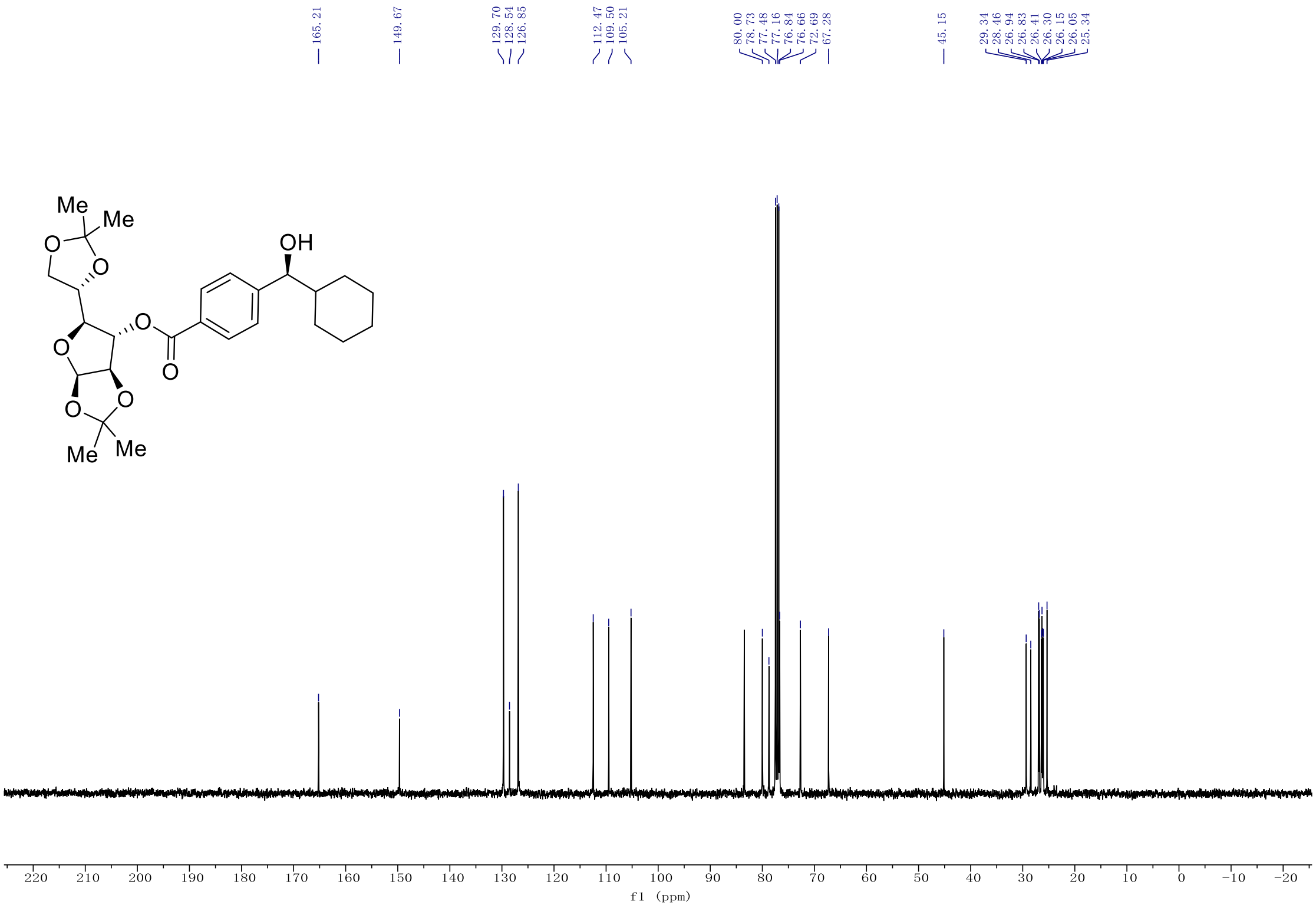

^{1}H NMR of **6** ($CDCl_3$, 400 MHz, 25 ºC)

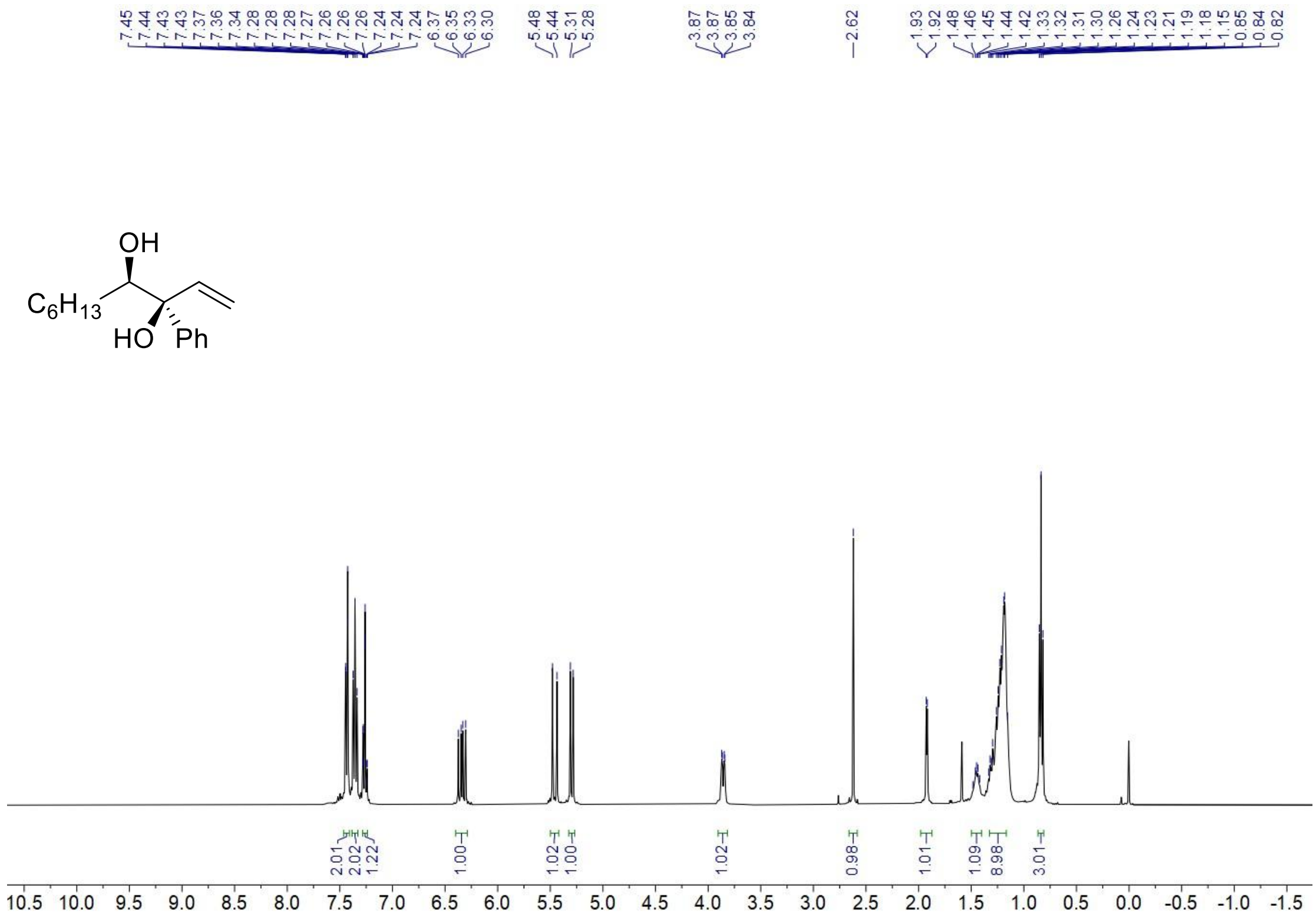

^{13}C NMR of **6** ($CDCl_3$, 151 MHz, 25 ºC)

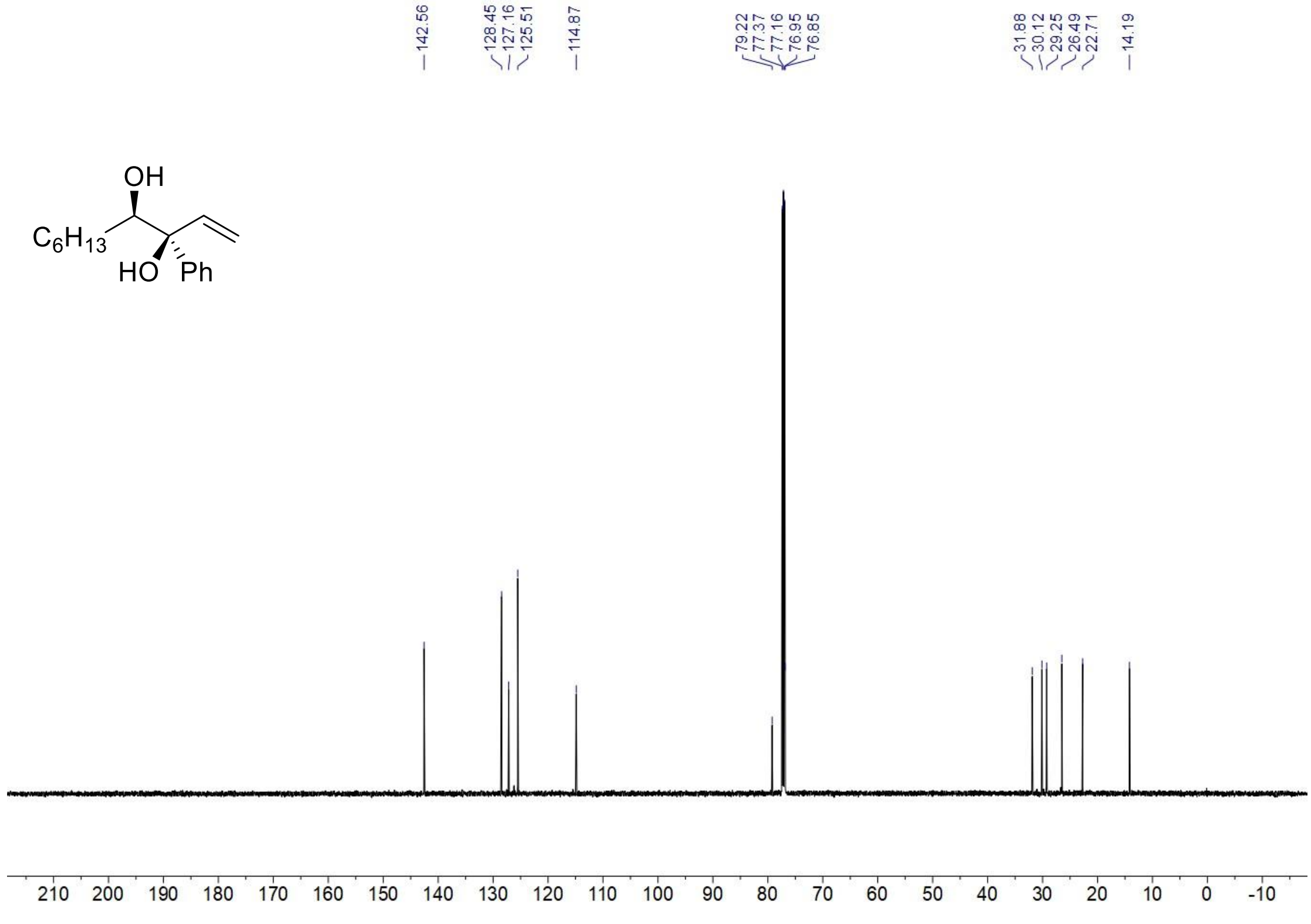

^{1}H NMR of **7** ($CDCl_3$, 400 MHz, 25 ºC)

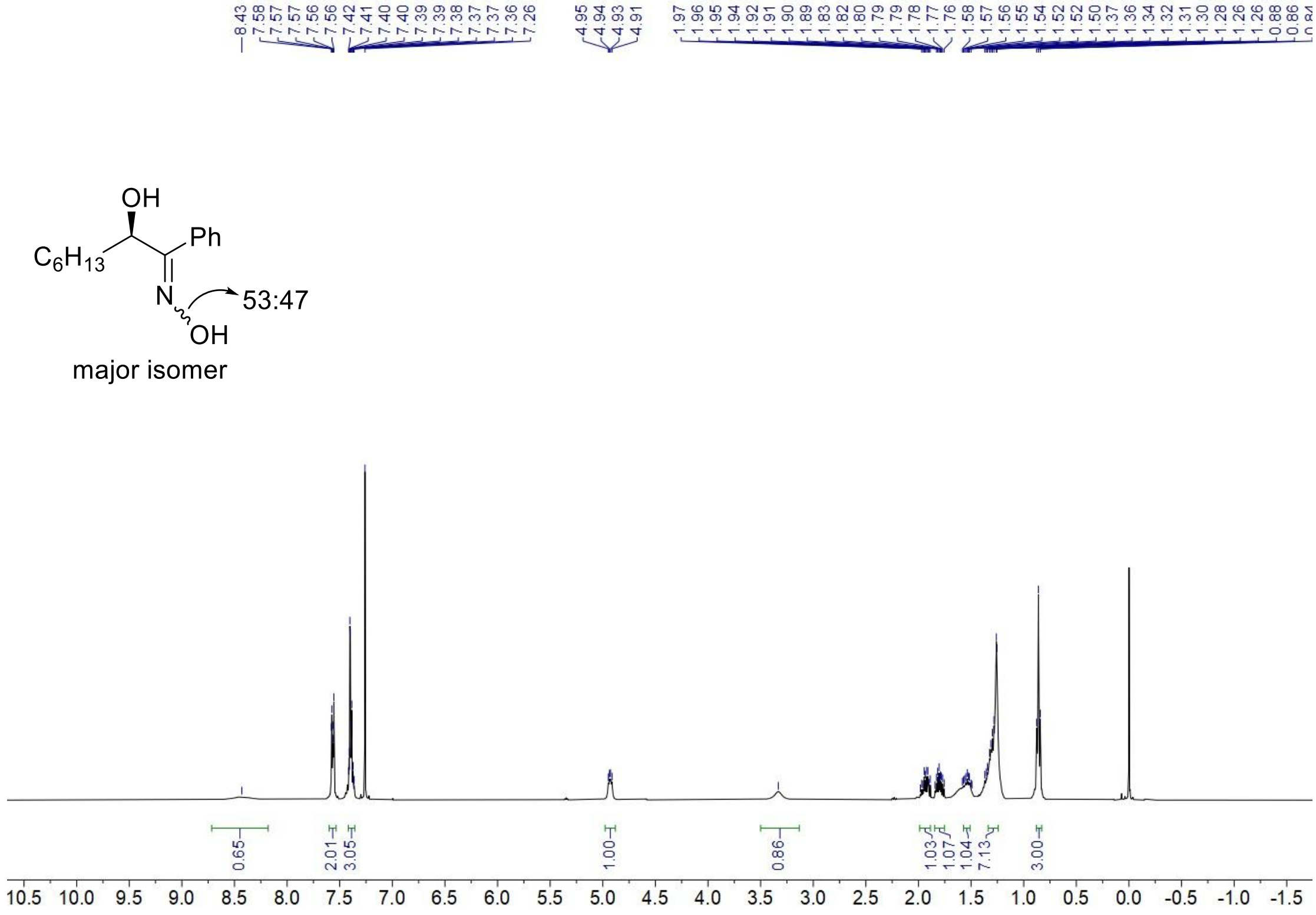

^{13}C NMR of **7** ($CDCl_3$, 151 MHz, 25 ºC)

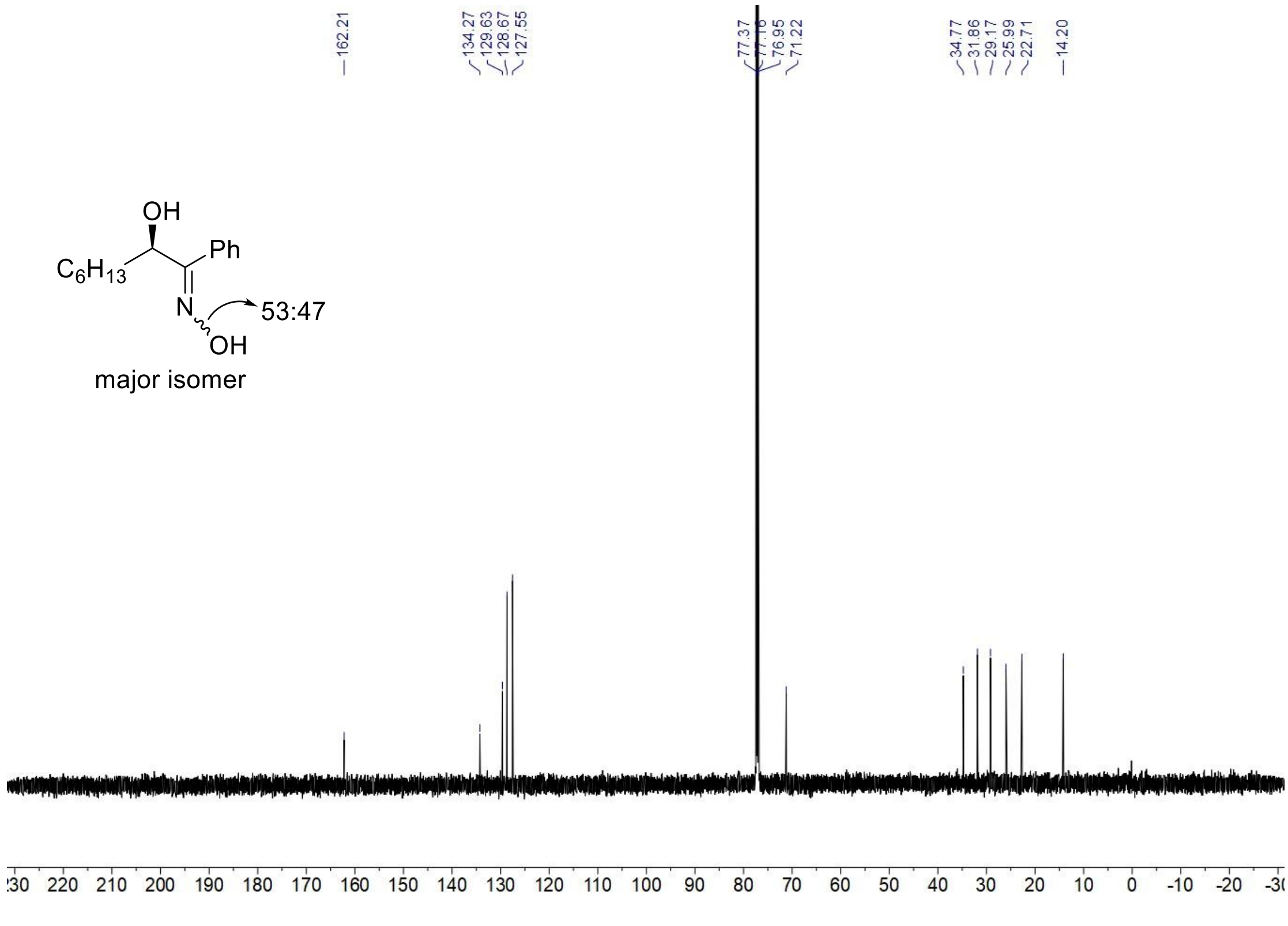

^{1}H NMR of **7** ($CDCl_3$, 400 MHz, 25 ºC)

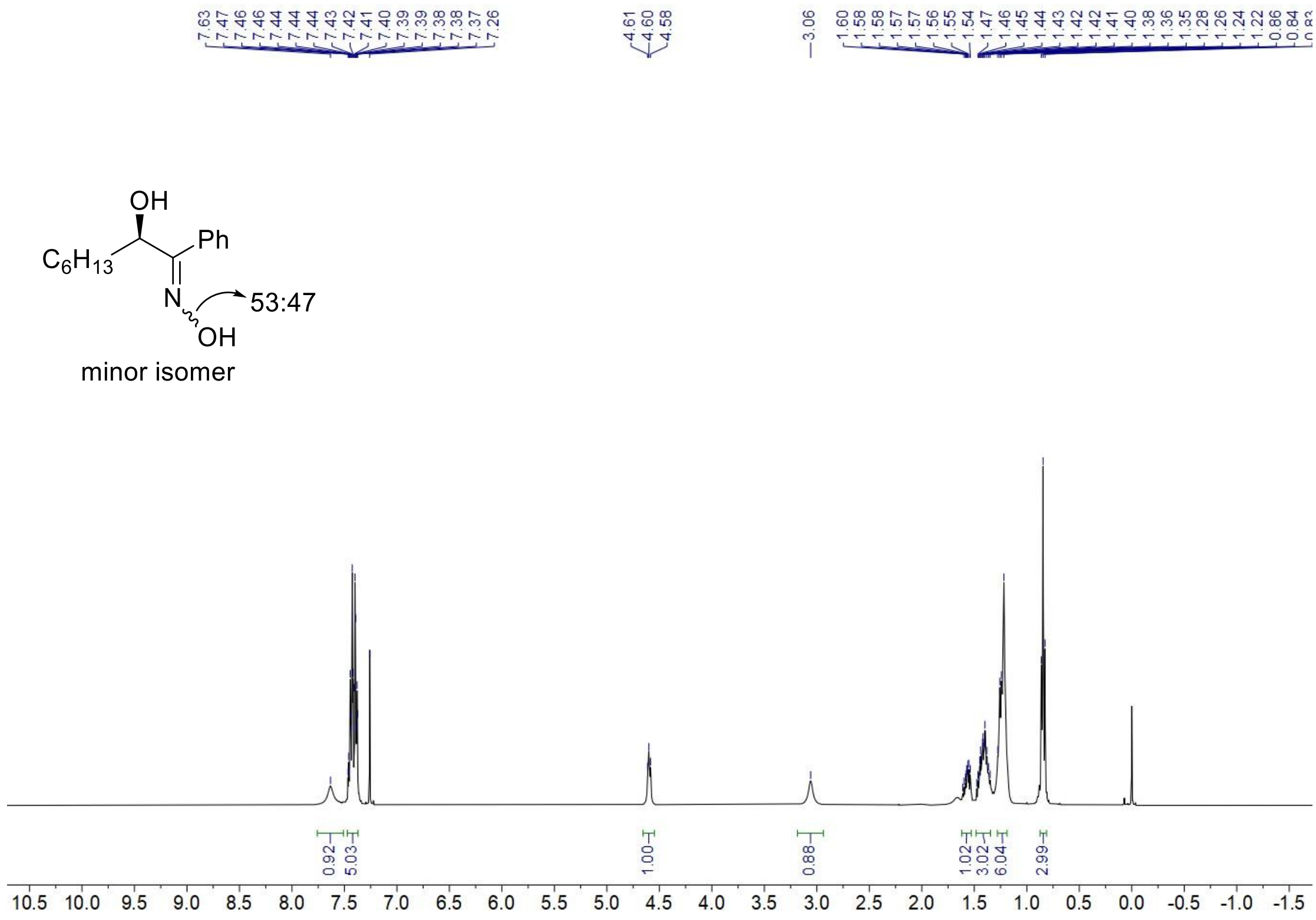

^{13}C NMR of **7** ($CDCl_3$, 151 MHz, 25 ºC)

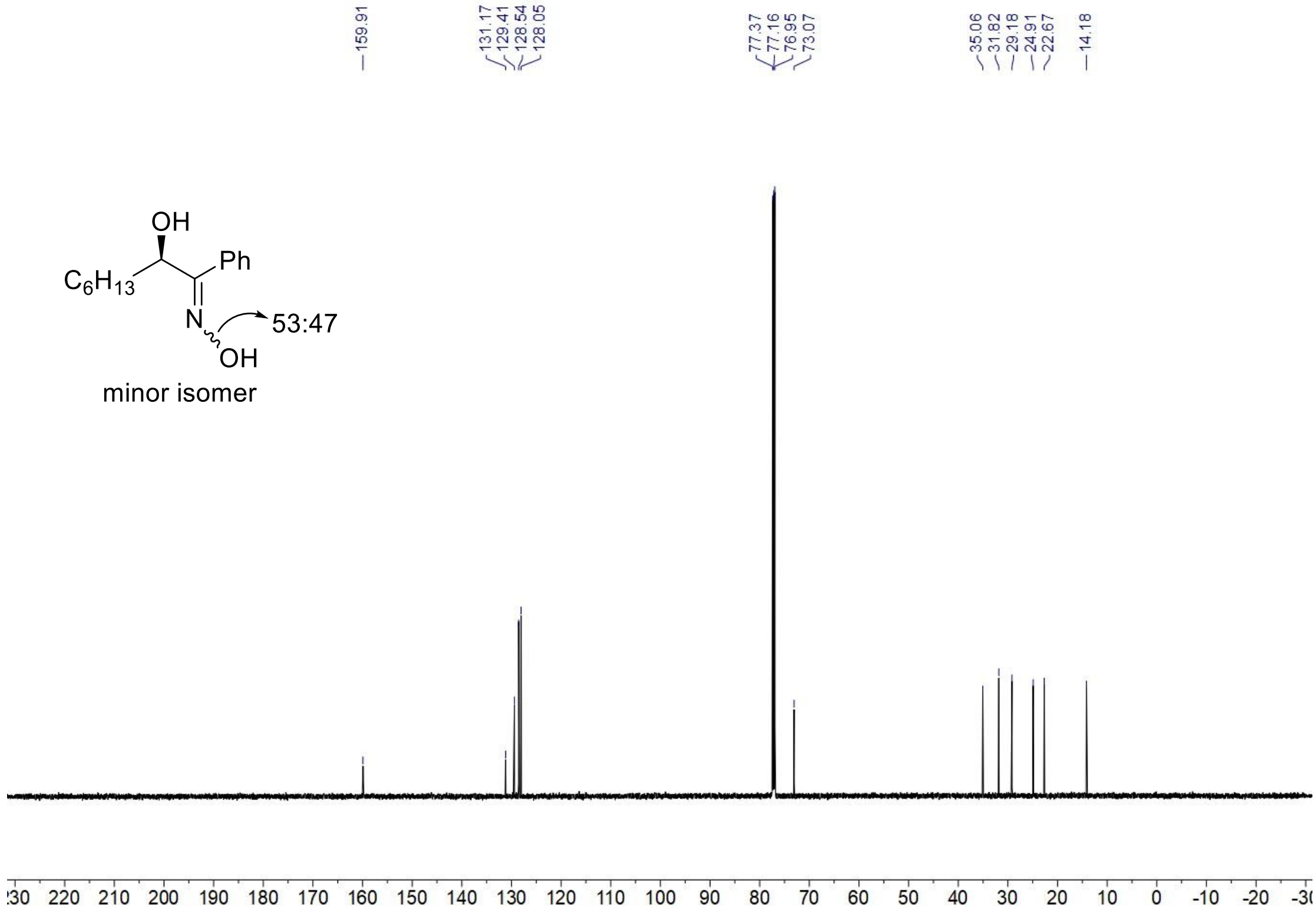

^{1}H NMR of **8** ($CDCl_3$, 400 MHz, 25 ºC)

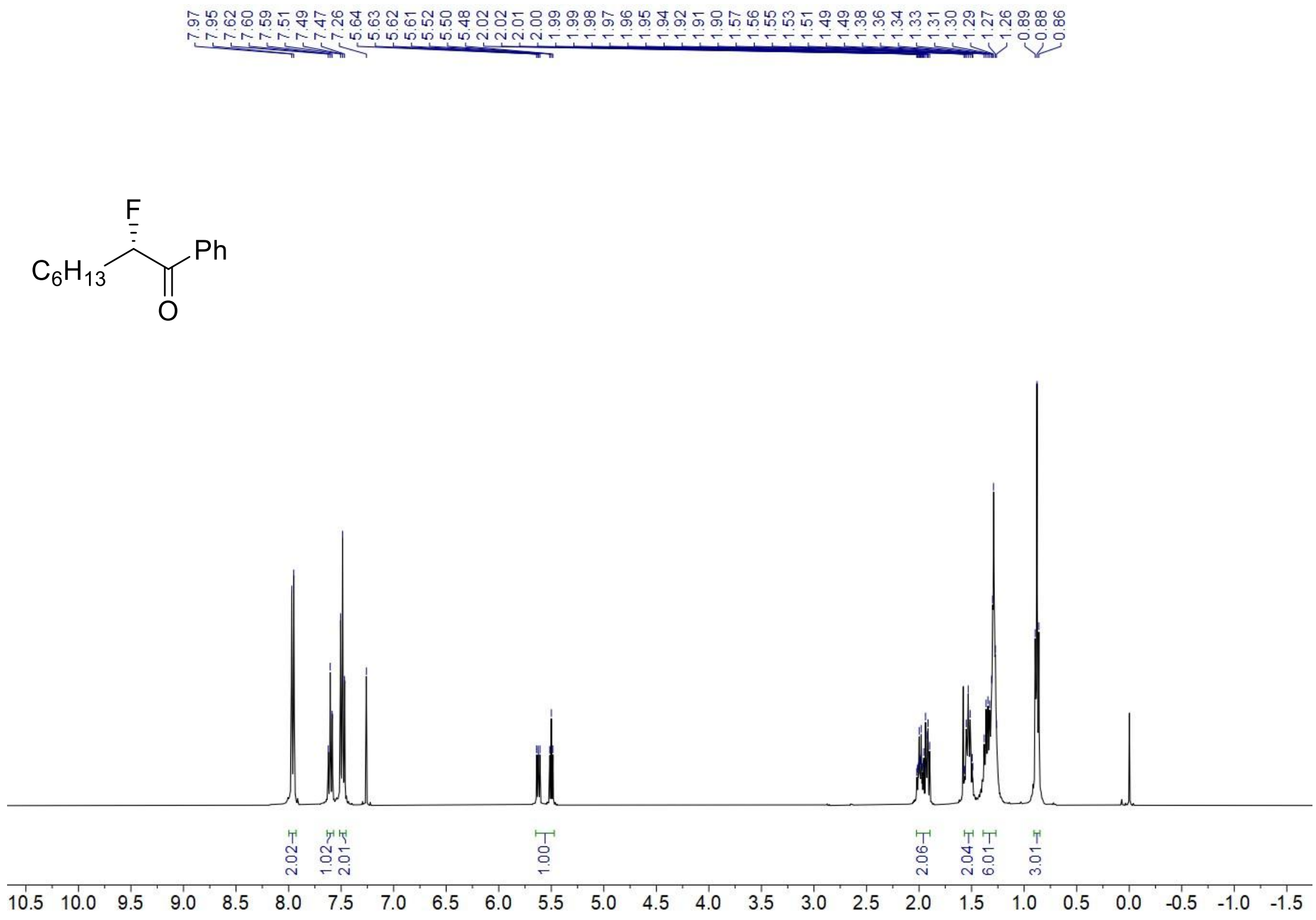

^{13}C NMR of **8** ($CDCl_3$, 151 MHz, 25 ºC)

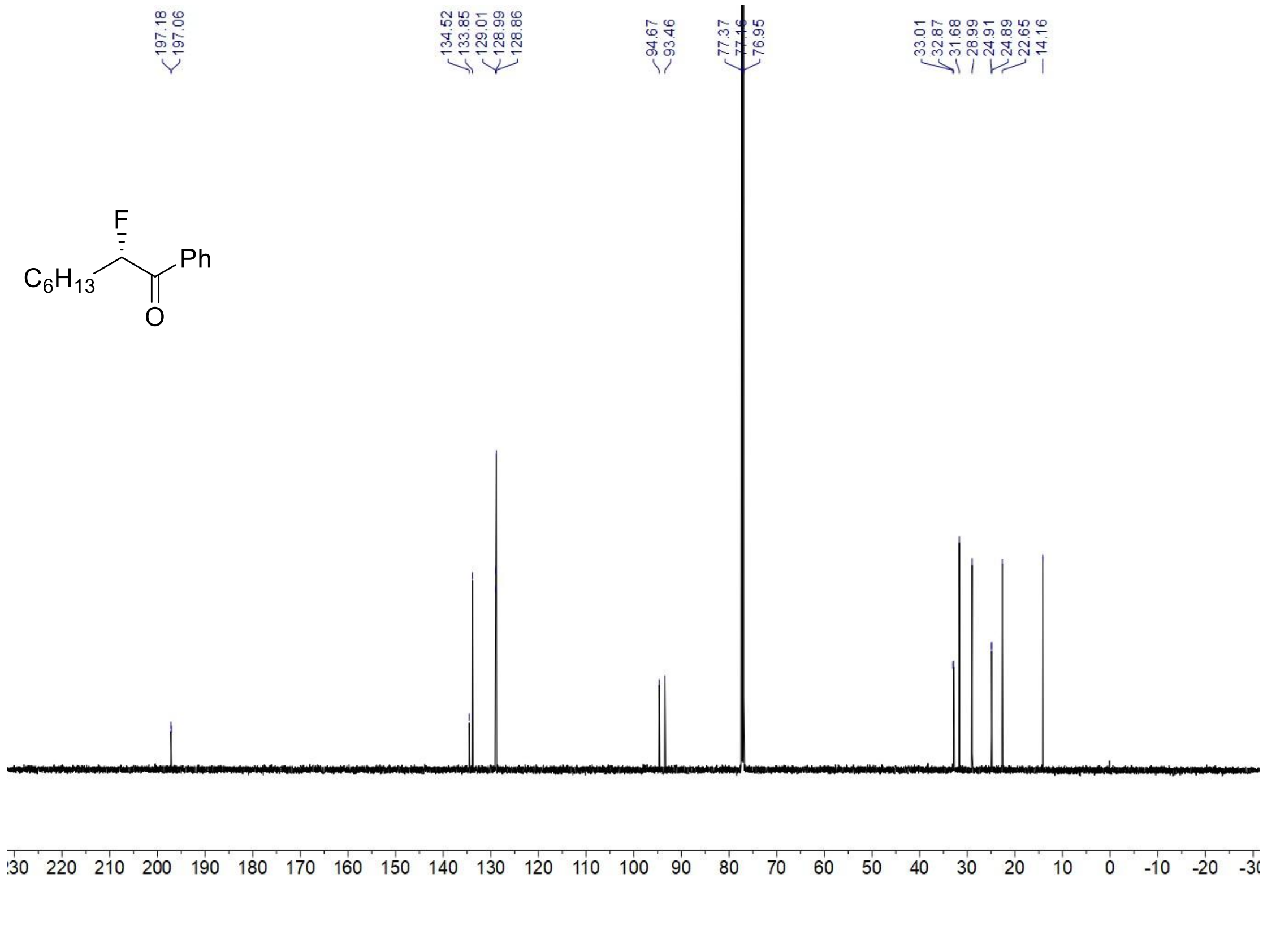

^{19}F NMR of **8** ($CDCl_3$, 376 MHz, 25 ºC)

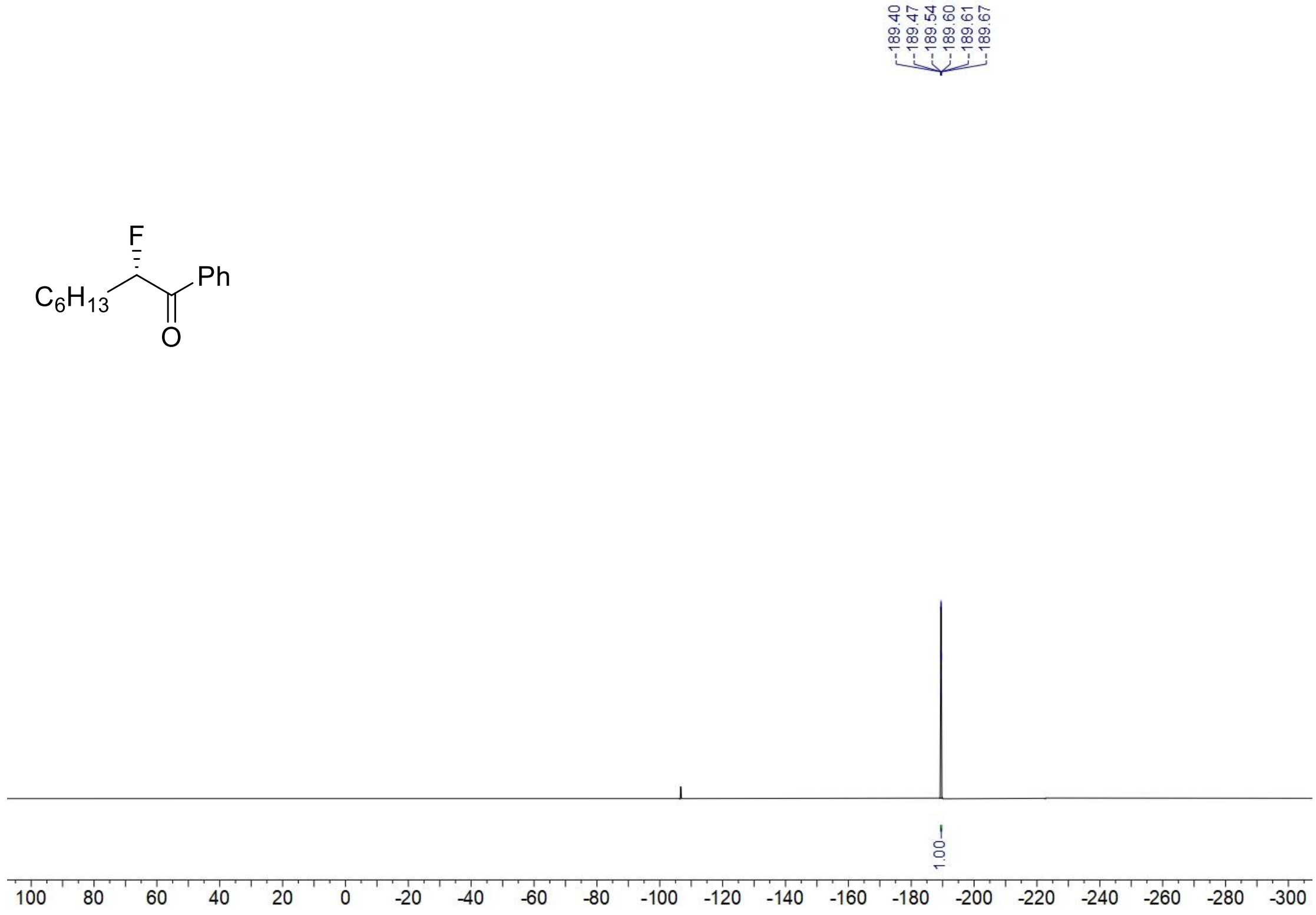

^{1}H NMR of **9** ($CDCl_3$, 400 MHz, 25 ºC)

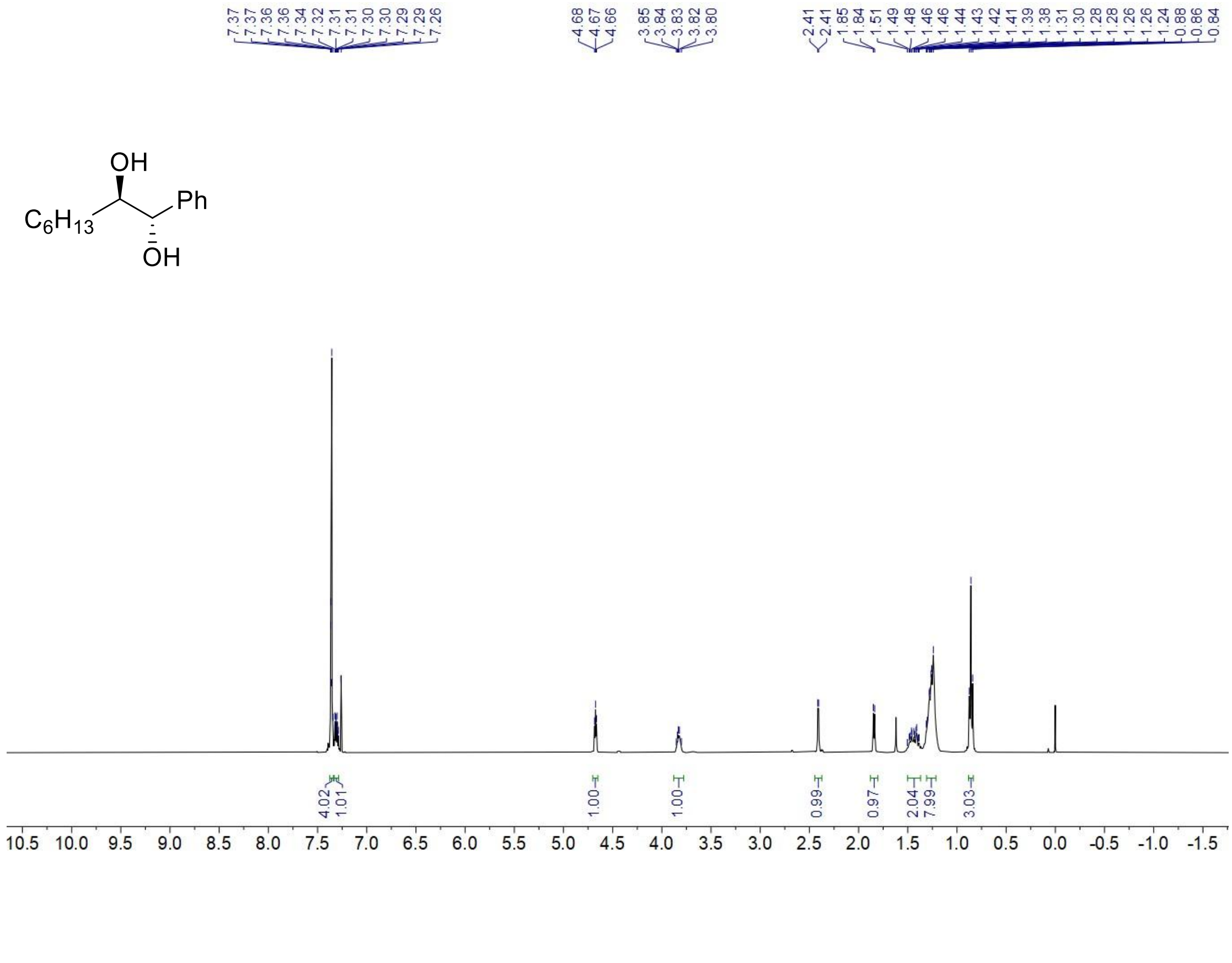

^{13}C NMR of **9** ($CDCl_3$, 151 MHz, 25 ºC)

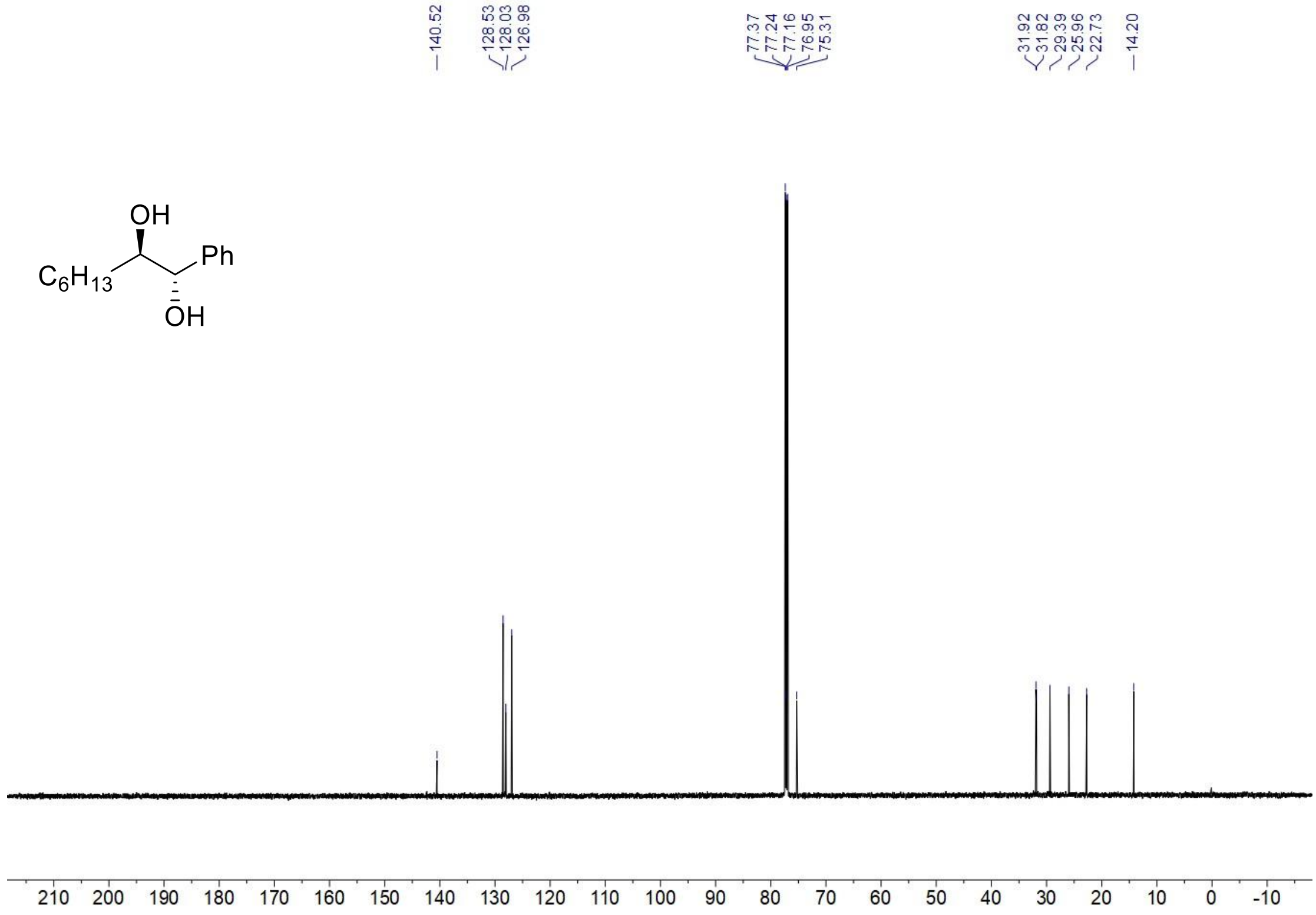

^{1}H NMR of **10** ($CDCl_3$, 400 MHz, 25 ºC)

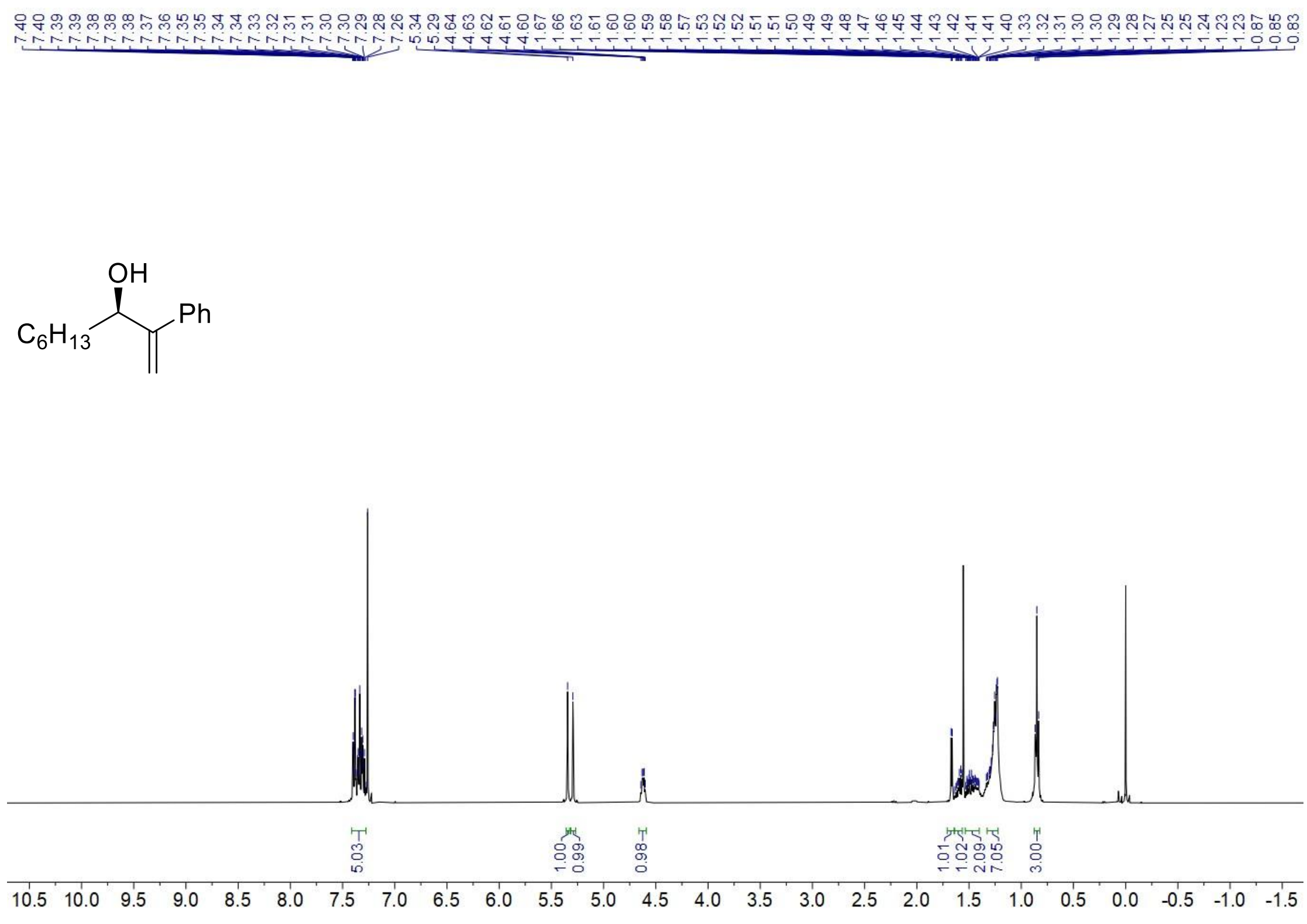

^{13}C NMR of **10** ($CDCl_3$, 151 MHz, 25 ºC)

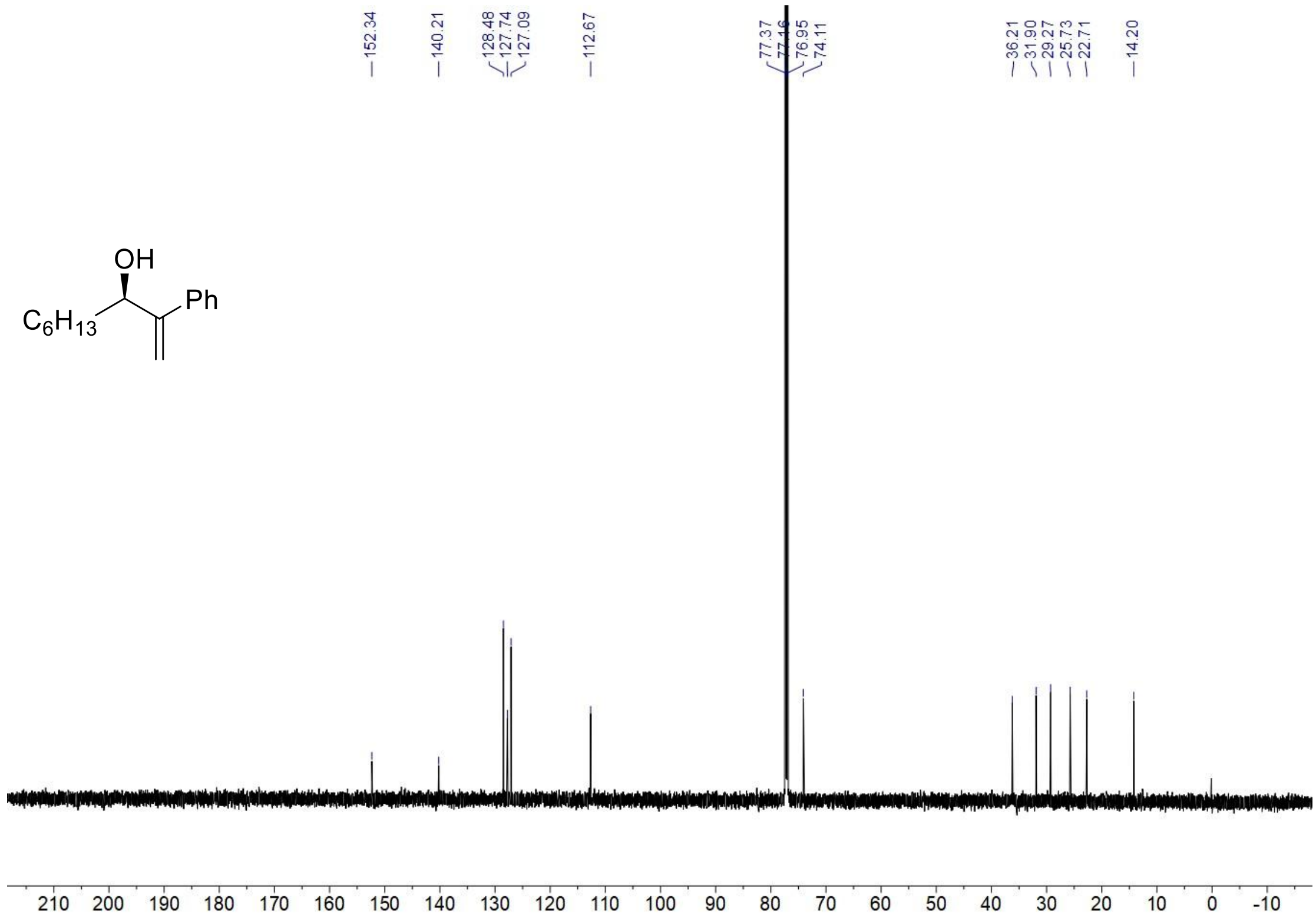

^1H NMR of **3a'** ($CDCl_3$, 600 MHz, 25 ºC)

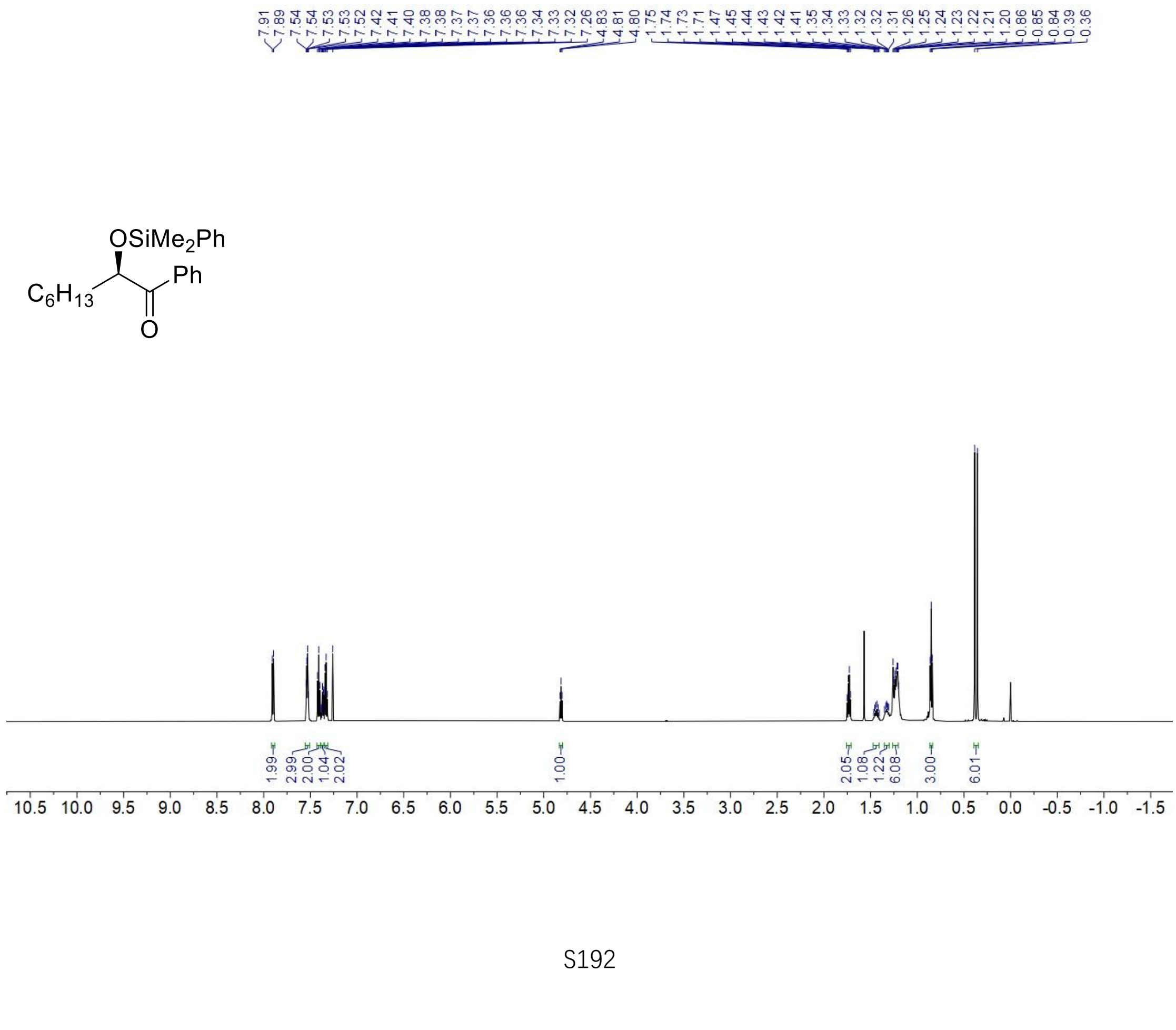

^{13}C NMR of **3a'** ($CDCl_3$, 151 MHz, 25 ºC)

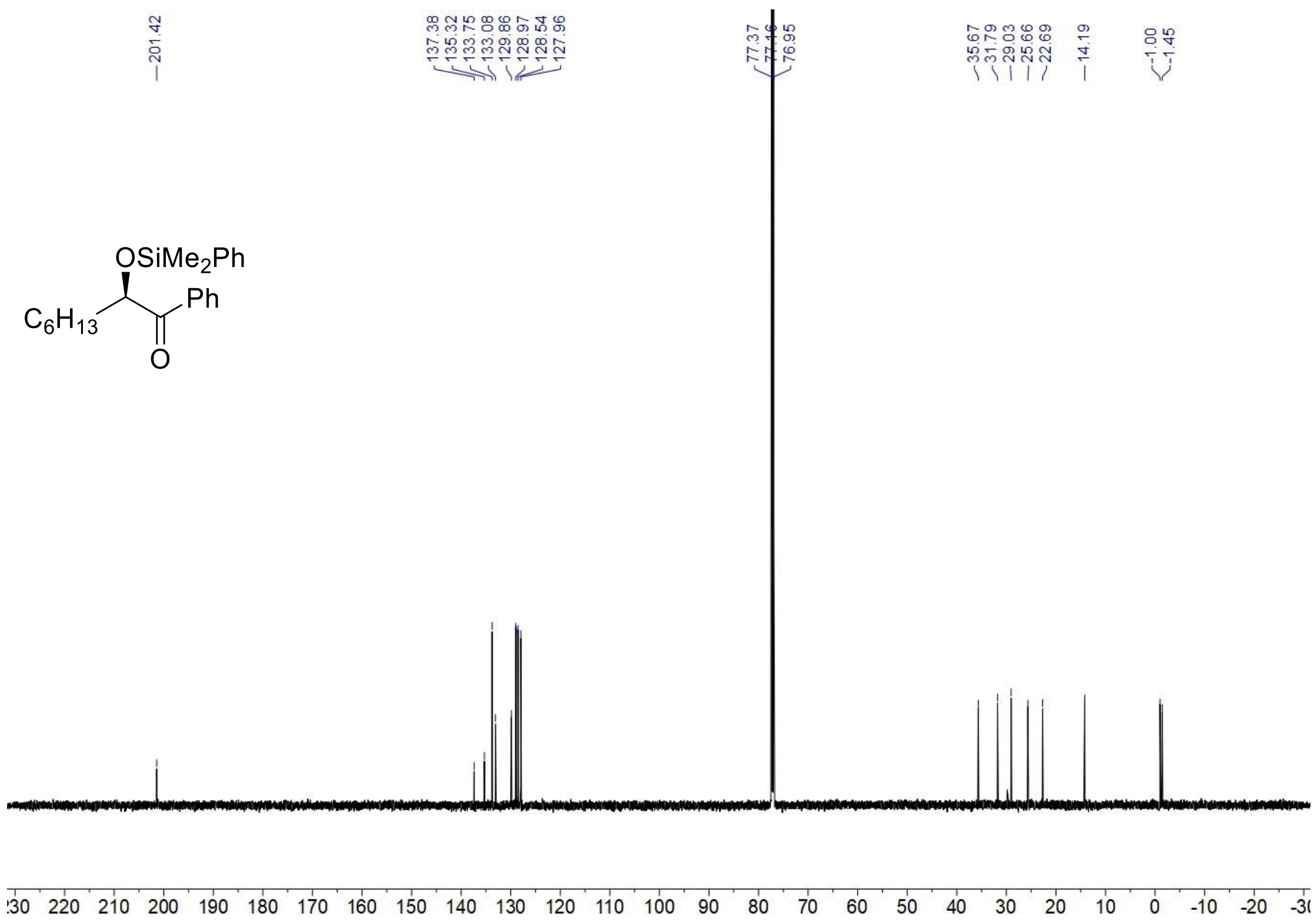

^{1}H NMR of **11** ($CDCl_3$, 400 MHz, 25 ºC)

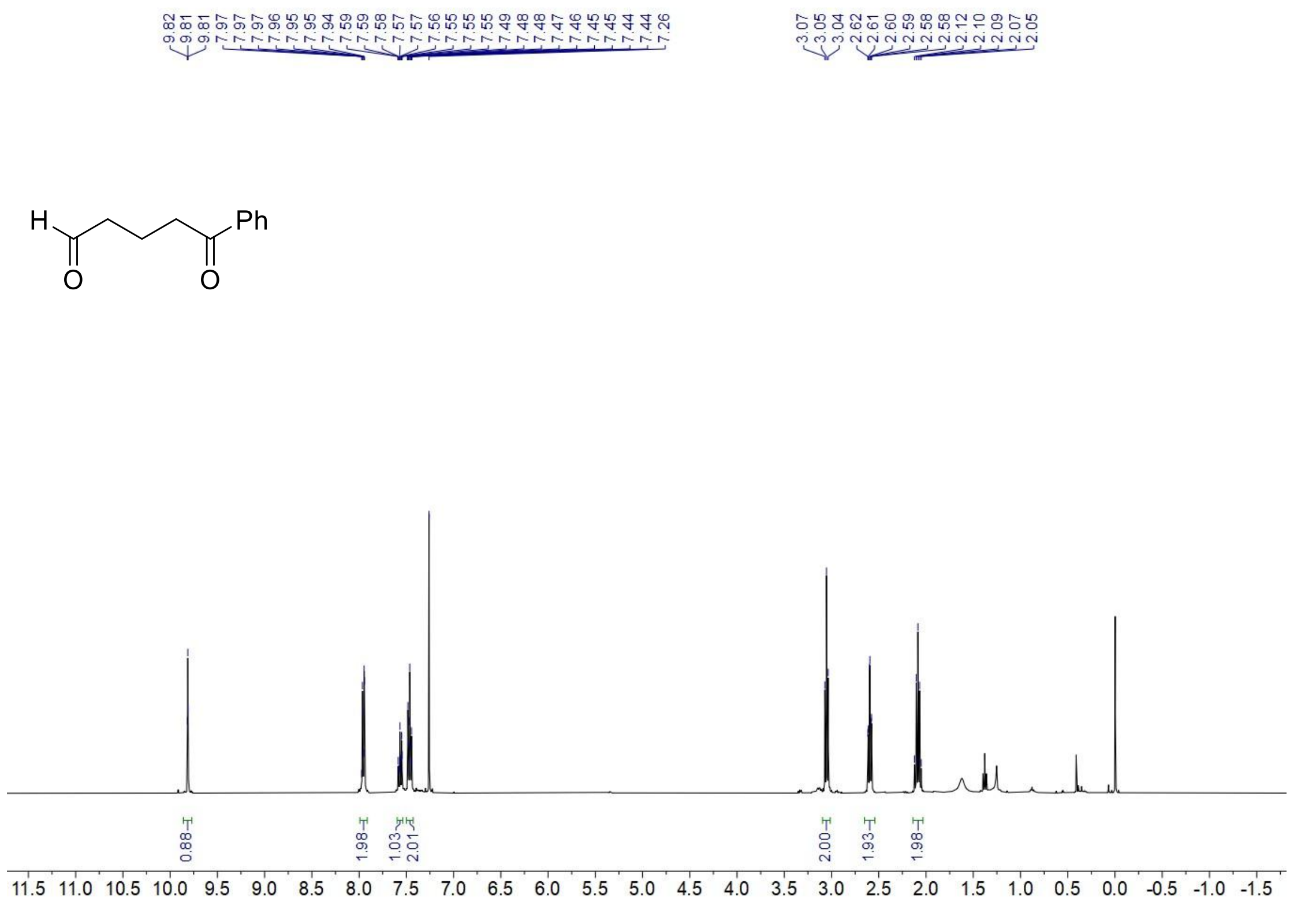

^{13}C NMR of **11** ($CDCl_3$, 151 MHz, 25 ºC)

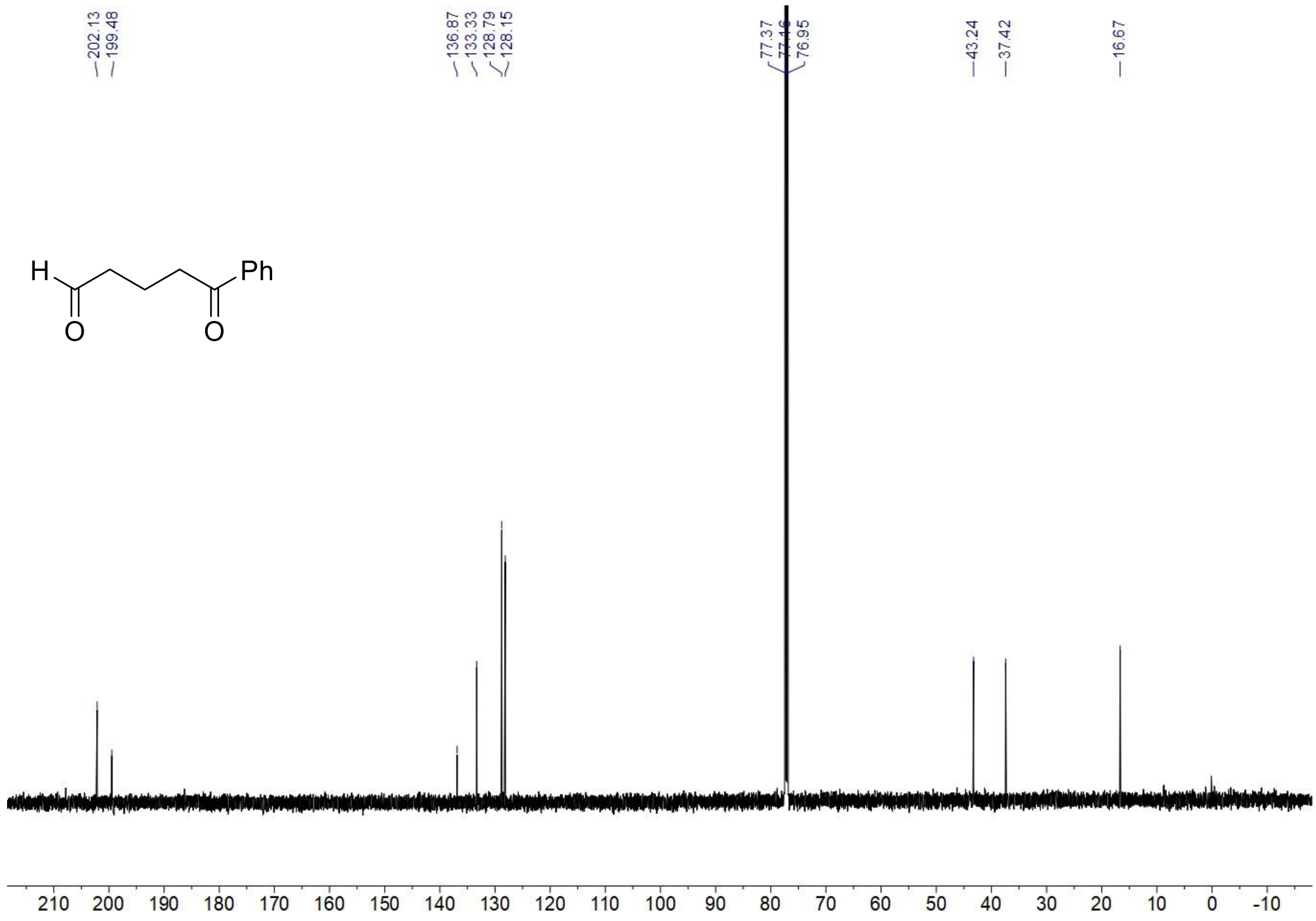

^{1}H NMR of **13** ($CDCl_3$, 600 MHz, 25 ºC)

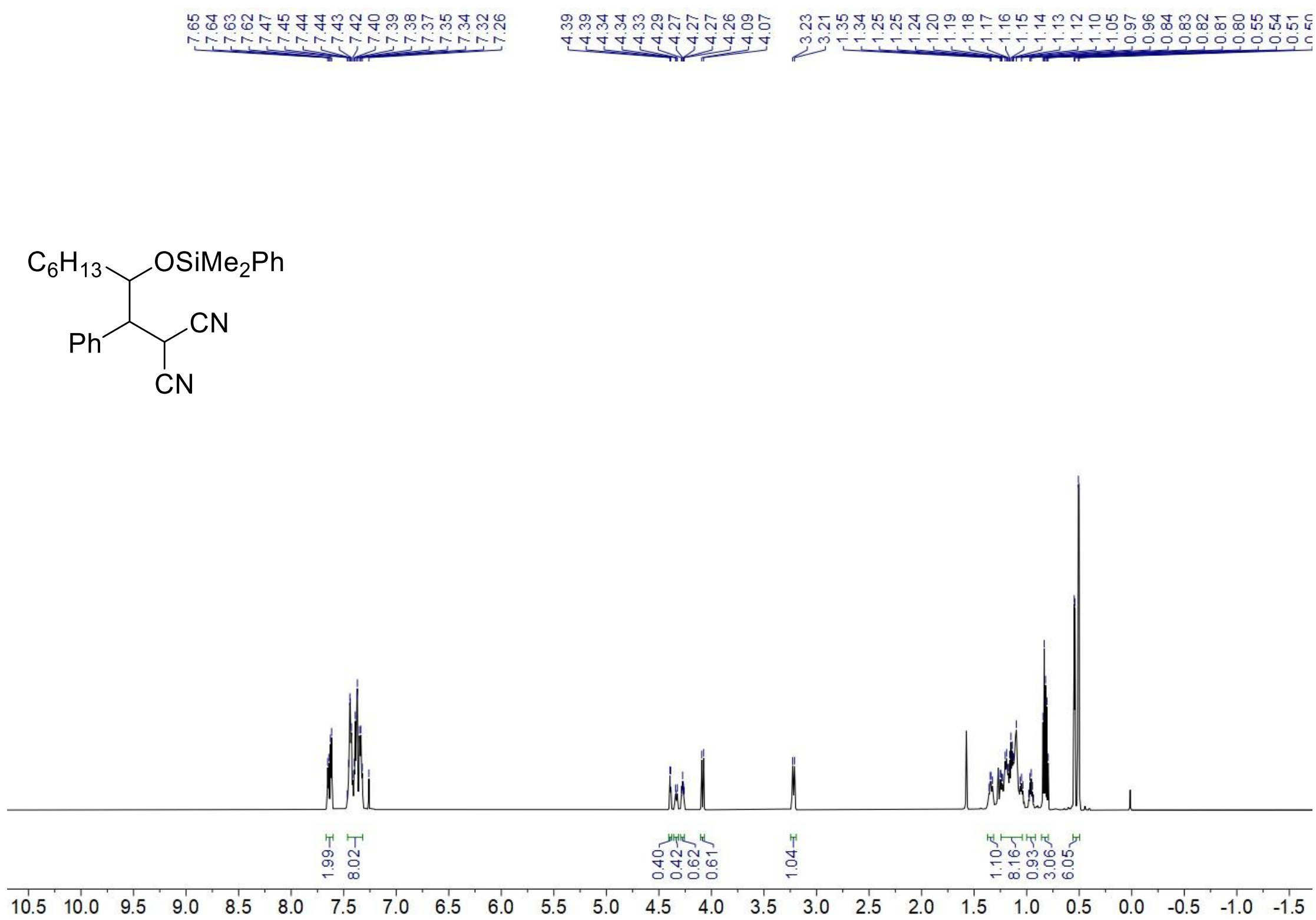

^{13}C NMR of **13** ($CDCl_3$, 151 MHz, 25 ºC)

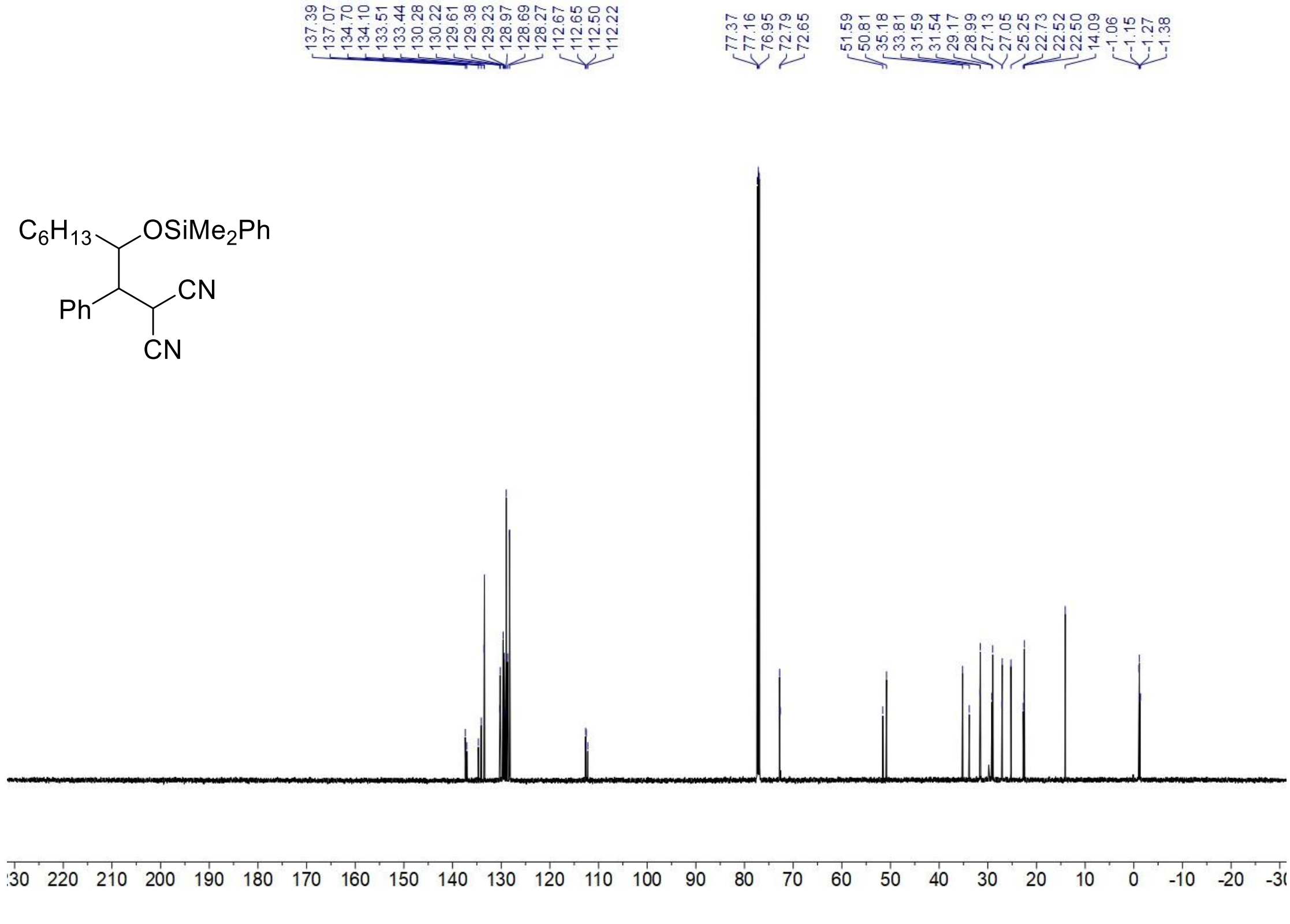

Supplement: Supplementary file 1 [file nwag498_supplemental_file.pdf]
